# Supplementary material for: Early Pregnancy Markers in the Serum of Ewes Identified via Proteomic and Metabolomic Analyses
Source: Int J Mol Sci. 2023 Sep 13;24(18):14054. doi: 10.3390/ijms241814054 (PMC10530974; doi:10.3390/ijms241814054)
Supplement: Supplementary file 1 [file ijms-24-14054-s001.zip › supplement tables/Table S2.docx]

| Accession_id | Protein name | Description | GO_term | KO_id | COG_id | Pfam_id | Subcellular Loc | N1 | N2 | N3 | P1 | P2 | P3 |
| --- | --- | --- | --- | --- | --- | --- | --- | --- | --- | --- | --- | --- | --- |
| A0A452FFB1 | CFH | A0A452FFB1_CAPHI Complement factor H OS=Capra hircus OX=9925 GN=CFH PE=4 SV=1 | BP:positive regulation of response to stimulus;BP:regulation of response to stimulus;BP:regulation of biological process;BP:response to external biotic stimulus;BP:interspecies interaction between organisms;BP:positive regulation of immune system process; | K23817;K23816;K23815;K04004 | ENOG410YBAR | PF00084.22 | extracellular | 150.2574768 | 871.9681396 | 1854.190796 | 181.529892 | 748.0119019 | 525.4960938 |
| A0A836CVL3 | JEQ12_006893 | A0A836CVL3_SHEEP Complement component C6 OS=Ovis aries OX=9940 GN=JEQ12_006893 PE=3 SV=1 | BP:positive regulation of response to stimulus;BP:regulation of response to stimulus;BP:embryo development;BP:embryo development ending in birth or egg hatching;BP:multicellular organism development;BP:cytolysis;BP:in utero embryonic development;BP:respon | K03998;K04000;K03995;K03996;K03997 | ENOG410YJ70 | PF01823.21;PF00084.22;PF00090.21;PF00057.20;PF19028.2 | cytoplasmic | 939.5975952 | 1043.491577 | 1091.929932 | 748.760437 | 1101.851563 | 1116.689209 |
| A0A836AAM7 | JEQ12_003140 | A0A836AAM7_SHEEP LRRCT domain-containing protein OS=Ovis aries OX=9940 GN=JEQ12_003140 PE=4 SV=1 | CC:cellular_component;CC:integral component of membrane;CC:cellular anatomical entity;CC:intrinsic component of membrane | K06261 | COG4886 | PF13855.8;PF12799.9 | extracellular | 26.73192024 | 31.98328781 | 17.38884735 | 39.3413353 | 35.80886459 | 20.06098938 |
| A0A836A9I6 | JEQ12_013894 | A0A836A9I6_SHEEP Ig-like domain-containing protein OS=Ovis aries OX=9940 GN=JEQ12_013894 PE=4 SV=1 | ------ | K06553 | ENOG4111RRF | PF07686.19;PF13927.8;PF00047.27;PF07679.18 | plasma membrane | 2792.546875 | 1499.83252 | 2373.68457 | 2788.981201 | 1293.260742 | 2624.576416 |
| W5NVB2 |  | W5NVB2_SHEEP SERPIN domain-containing protein OS=Ovis aries OX=9940 PE=4 SV=1 | CC:cellular_component;CC:extracellular space;CC:cellular anatomical entity | K04525 | COG4826 | PF00079.22 | cytoplasmic | 1007.871094 | 724.4764404 | 748.8165894 | 698.3317261 | 1188.9552 | 547.7418823 |
| A0A836A504 | JEQ12_003656 | A0A836A504_SHEEP Coagulation factor V OS=Ovis aries OX=9940 GN=JEQ12_003656 PE=3 SV=1 | BP:hemostasis;BP:blood coagulation;BP:circulatory system process;BP:biological regulation;BP:multicellular organismal process;BP:regulation of body fluid levels;BP:blood circulation;BP:coagulation;BP:biological_process;BP:system process;BP:regulation of b | K13624;K03899;K03902;K14735 | ENOG4111F6G | PF00754.27;PF07732.17;PF07731.16 | extracellular | 434.3057556 | 228.0440521 | 269.4511414 | 275.4198914 | 600.5560913 | 265.6899414 |
| A0A452EX53 | LOC106503728 | A0A452EX53_CAPHI Serum amyloid A protein OS=Capra hircus OX=9925 GN=LOC106503728 PE=3 SV=1 | BP:response to stimulus;BP:defense response;BP:acute-phase response;BP:response to stress;BP:biological_process;BP:inflammatory response;BP:acute inflammatory responseCC:protein-lipid complex;CC:high-density lipoprotein particle;CC:plasma lipoprotein part | K17310 | ENOG410YSBK | PF00277.20 | ER | 608.9703369 | 13.81392574 | 3.959215641 | 8.910122871 | 556.3913574 | 253.2473907 |
| A0A452G9E8 | ATRN | A0A452G9E8_CAPHI Attractin OS=Capra hircus OX=9925 GN=ATRN PE=4 SV=1 | BP:regulation of developmental growth;BP:ensheathment of neurons;BP:regulation of biological process;BP:myelination;BP:biological regulation;BP:developmental process;BP:regulation of developmental process;BP:cellular process;BP:biological_process;BP:axon | K23664;K24332;K05502 | ENOG410XRW4 | PF01344.27;PF13964.8;PF00059.23;PF01437.27;PF13854.8;PF13418.8;PF00431.22 | plasma membrane | 96.20744324 | 108.8383484 | 102.312355 | 105.9961014 | 96.448349 | 93.21163177 |
| W5NWX6 | LOC101117273 | W5NWX6_SHEEP Apolipoprotein C-III OS=Ovis aries OX=9940 GN=LOC101117273 PE=3 SV=1 | BP:regulation of metabolic process;BP:regulation of primary metabolic process;BP:negative regulation of lipid metabolic process;BP:regulation of triglyceride catabolic process;BP:negative regulation of molecular function;BP:negative regulation of biologic | K08759 | ENOG4111APE | PF05778.14;PF04691.14 | extracellular | 5.554385662 | 7.109713078 | 13.09944534 | 38.88830185 | 15.88189983 | 18.98579788 |
| P68116 | FGB | FIBB_SHEEP Fibrinogen beta chain (Fragment) OS=Ovis aries OX=9940 GN=FGB PE=1 SV=1 | BP:hemostasis;BP:blood coagulation;BP:response to external biotic stimulus;BP:interspecies interaction between organisms;BP:biological regulation;BP:regulation of biological quality;BP:multicellular organismal process;BP:regulation of body fluid levels;BP | K03904 | ------ | ------ | cytoplasmic | 68.93638611 | 24.13338089 | 123.2745438 | 62.6953125 | 90.5462265 | 42.5264473 |
| A0A836D7I1 | JEQ12_010126 | A0A836D7I1_SHEEP C1q domain-containing protein OS=Ovis aries OX=9940 GN=JEQ12_010126 PE=4 SV=1 | BP:synapse organization;BP:cell junction organization;BP:regulation of response to stimulus;BP:cell junction disassembly;BP:synapse disassembly;BP:regulation of biological process;BP:cellular component organization or biogenesis;BP:response to external bi | K03987;K03986;K23284;K03988 | ENOG4111MQB | PF00386.23;PF01391.20 | extracellular | 543.2513428 | 360.8677368 | 465.4699402 | 490.8668518 | 398.1327515 | 502.8553162 |
| Q7M371 |  | Q7M371_SHEEP Plasma proteinase inhibitor (Fragment) OS=Ovis aries OX=9940 PE=1 SV=1 | ------ | ------ | ------ | ------ | cytoplasmic | 27.96687889 | 0 | 72.46517181 | 48.44050217 | 120.9486237 | 37.4636879 |
| A0A452EN63 | FGB | A0A452EN63_CAPHI Fibrinogen beta chain OS=Capra hircus OX=9925 GN=FGB PE=4 SV=1 | BP:negative regulation of apoptotic signaling pathway;BP:positive regulation of heterotypic cell-cell adhesion;BP:regulation of extrinsic apoptotic signaling pathway;BP:negative regulation of extrinsic apoptotic signaling pathway;BP:regulation of secretio | K03905;K03904;K03903 | ENOG410ZYS4 | PF00147.20;PF08702.12 | ER | 22.69008064 | 22.84495354 | 40.85148621 | 34.79816437 | 25.2329464 | 37.66075516 |
| A0A836AM15 | JEQ12_000810 | A0A836AM15_SHEEP Threonine--tRNA ligase OS=Ovis aries OX=9940 GN=JEQ12_000810 PE=3 SV=1 | BP:threonyl-tRNA aminoacylation;BP:carboxylic acid metabolic process;BP:nucleic acid metabolic process;BP:cellular nitrogen compound metabolic process;BP:nitrogen compound metabolic process;BP:small molecule metabolic process;BP:ncRNA metabolic process;BP | K01868;K23867;K17420 | COG0441 | PF05782.13;PF00587.27;PF02824.23;PF07973.16;PF03129.22 | cytoplasmic | 660.256897 | 359.7340088 | 517.677063 | 421.757843 | 560.7421265 | 813.1502075 |
| A0A452FDR0 | BST1 | A0A452FDR0_CAPHI ADP-ribosyl cyclase/cyclic ADP-ribose hydrolase OS=Capra hircus OX=9925 GN=BST1 PE=3 SV=1 | BP:positive regulation of cell proliferation;BP:regulation of cell activation;BP:regulation of B cell activation;BP:positive regulation of cell activation;BP:regulation of B cell proliferation;BP:biological regulation;BP:positive regulation of mononuclear | K18152 | ENOG4111W33 | PF02267.19 | mitochondrial | 114.8716812 | 107.9577942 | 0 | 3.979661465 | 193.3970032 | 102.2054367 |
| A0A452ESV0 | MGAM | A0A452ESV0_CAPHI Maltase-glucoamylase OS=Capra hircus OX=9925 GN=MGAM PE=3 SV=1 | BP:organic substance metabolic process;BP:biological_process;BP:metabolic process;BP:primary metabolic process;BP:carbohydrate metabolic processCC:lytic vacuole;CC:intracellular organelle;CC:membrane-bounded organelle;CC:organelle;CC:intrinsic component o | K01203;K12316;K12047;K12317 | COG1501 | PF01055.28;PF16863.7;PF00088.20;PF13802.8 | lysosomal | 7.641057968 | 18.40047073 | 13.13722229 | 39.21939087 | 9.316045761 | 8.255054474 |
| A0A452E6R6 |  | A0A452E6R6_CAPHI Deleted in malignant brain tumors 1 protein OS=Capra hircus OX=9925 PE=3 SV=1 | ------ | K06545 | ENOG410XQVR | PF00530.20;PF00100.25;PF00431.22;PF15494.8 | peroxisomal | 101.4203262 | 47.76544189 | 24.50911331 | 10.49352551 | 82.45705414 | 31.89462662 |
| W5P4S0 | CP | W5P4S0_SHEEP Ceruloplasmin OS=Ovis aries OX=9940 GN=CP PE=3 SV=1 | BP:copper ion transport;BP:transition metal ion transport;BP:cation transport;BP:ion transport;BP:transport;BP:metal ion transport;BP:establishment of localization;BP:localization;BP:biological_processCC:cellular anatomical entity;CC:cellular_component;CC | K03899;K14735;K13624 | COG2132 | PF07731.16;PF07732.17;PF00394.24 | extracellular | 4501.459473 | 3237.055664 | 4063.401855 | 4072.012695 | 4640.338867 | 5264.03125 |
| A0A835ZJZ8 | JEQ12_012929 | A0A835ZJZ8_SHEEP Serglycin OS=Ovis aries OX=9940 GN=JEQ12_012929 PE=4 SV=1 | BP:negative regulation of biomineralization;BP:signal transduction;BP:regulation of bone mineralization;BP:negative regulation of bone mineralization;BP:regulation of biological process;BP:cellular component organization or biogenesis;BP:negative regulati | K06849 | ENOG4111DTK | PF04360.14 | extracellular | 381.5384216 | 247.9056549 | 287.6787109 | 435.9146729 | 205.9109955 | 203.6572723 |
| W5NQW9 | LOC101104482 | W5NQW9_SHEEP Alpha-1-macroglobulin-like OS=Ovis aries OX=9940 GN=LOC101104482 PE=3 SV=1 | CC:cellular_component;CC:cellular anatomical entity;CC:extracellular spaceMF:endopeptidase inhibitor activity;MF:endopeptidase regulator activity;MF:peptidase regulator activity;MF:molecular_function;MF:peptidase inhibitor activity;MF:serine-type endopept | K03910;K06530;K23589;K23593 | COG2373;ENOG410XRED | PF07678.16;PF07703.16;PF07677.16;PF00207.24;PF17791.3;PF17789.3;PF01835.21;PF05326.13 | extracellular | 3688.133789 | 1867.728638 | 1393.17395 | 2029.202515 | 1239.912598 | 1267.460205 |
| A0A452F8Z9 | USP32 | A0A452F8Z9_CAPHI Ubiquitinyl hydrolase 1 OS=Capra hircus OX=9925 GN=USP32 PE=4 SV=1 | ------ | K21343;K11835;K11847;K11837 | COG5560 | PF00443.31;PF06337.14;PF13423.8;PF13405.8;PF13202.8;PF00036.34;PF13499.8 | cytoplasmic | 282.4483948 | 96.03367615 | 335.8036194 | 324.5999146 | 168.8366394 | 0 |
| A0A836AKC1 | JEQ12_015006 | A0A836AKC1_SHEEP Complement subcomponent C1r OS=Ovis aries OX=9940 GN=JEQ12_015006 PE=4 SV=1 | BP:immune system process;BP:positive regulation of response to stimulus;BP:regulation of response to stimulus;BP:nitrogen compound metabolic process;BP:regulation of biological process;BP:zymogen activation;BP:organic substance metabolic process;BP:positi | K01331;K01330;K03993;K03992 | COG5640 | PF00089.28;PF00431.22;PF00084.22;PF14670.8;PF12662.9 | extracellular | 801.2759399 | 577.1011963 | 679.8959351 | 670.2039795 | 640.0230103 | 708.0150146 |
| A0A452FYF5 | THBS4 | A0A452FYF5_CAPHI Thrombospondin 4 OS=Capra hircus OX=9925 GN=THBS4 PE=3 SV=1 | BP:regulation of phosphate metabolic process;BP:regulation of metabolic process;BP:positive regulation of response to stimulus;BP:regulation of response to stimulus;BP:tissue remodeling;BP:behavior;BP:regulation of chemotaxis;BP:positive regulation of che | K16857;K04659 | ENOG410XQKE | PF05735.14;PF02412.20;PF11598.10;PF07645.17;PF12947.9;PF00008.29 | ER | 34.1622963 | 202.4964294 | 141.2134552 | 114.1227798 | 68.23097229 | 97.9887085 |
| A0A8C2PAV7 |  | A0A8C2PAV7_CAPHI Insulin-like growth factor-binding protein 4 OS=Capra hircus OX=9925 PE=4 SV=1 | BP:regulation of primary metabolic process;BP:regulation of metabolic process;BP:positive regulation of response to stimulus;BP:regulation of response to stimulus;BP:regulation of cellular component organization;BP:regulation of glucose metabolic process; | K23575;K23576;K23577;K23578;K10138 | ENOG4111HXV | PF00219.20 | mitochondrial | 5.723431587 | 9.099815369 | 10.45273495 | 6.79915905 | 10.43236446 | 8.392217636 |
| A0A452E5P2 | PRG4 | A0A452E5P2_CAPHI Proteoglycan 4 OS=Capra hircus OX=9925 GN=PRG4 PE=4 SV=1 | BP:negative regulation of metabolic process;BP:regulation of metabolic process;BP:negative regulation of gene expression;BP:regulation of biological process;BP:immune system process;BP:negative regulation of macromolecule metabolic process;BP:negative reg | K14648;K06251;K24286 | ENOG410XQ5D | PF00045.21;PF01033.19 | extracellular | 161.6507416 | 78.995224 | 113.4454422 | 133.0603485 | 130.2198486 | 96.45042419 |
| A0A835ZRY0 | JEQ12_007502 | A0A835ZRY0_SHEEP Ig-like domain-containing protein OS=Ovis aries OX=9940 GN=JEQ12_007502 PE=4 SV=1 | ------ | K06553 | ENOG41117U9;ENOG410Y9T8 | PF07686.19;PF13927.8;PF07679.18 | extracellular | 5860.874512 | 4463.284668 | 6817.218262 | 7018.262207 | 2928.549561 | 6883.441895 |
| W5PBY0 | C4BPA | W5PBY0_SHEEP Complement component 4 binding protein alpha OS=Ovis aries OX=9940 GN=C4BPA PE=4 SV=1 | BP:positive regulation of catabolic process;BP:regulation of catabolic process;BP:regulation of complement activation;BP:regulation of primary metabolic process;BP:regulation of metabolic process;BP:negative regulation of response to stimulus;BP:regulatio | K04002;K04012 | ENOG410XPJ1 | PF00084.22;PF18453.3 | extracellular | 8270.607422 | 5015.369629 | 8621.68457 | 8529.966797 | 9549.130859 | 8483.556641 |
| A0A8C2NQS3 |  | A0A8C2NQS3_CAPHI Ig-like domain-containing protein OS=Capra hircus OX=9925 PE=4 SV=1 | ------ | K06554;K06553;K06551 | ENOG41115Q6;ENOG410YQB2 | PF07686.19;PF07654.17;PF13927.8 | mitochondrial | 76.5656662 | 1477.360596 | 1292.087158 | 119.1861877 | 230.1182404 | 1782.408813 |
| A0A452EAV9 | C8B | A0A452EAV9_CAPHI Complement component C8 beta chain OS=Capra hircus OX=9925 GN=C8B PE=3 SV=1 | BP:positive regulation of response to stimulus;BP:regulation of response to stimulus;BP:regulation of biological process;BP:cytolysis;BP:response to external biotic stimulus;BP:interspecies interaction between organisms;BP:positive regulation of immune sy | K03998;K04000;K03995;K03996;K03997 | ENOG410Y2J1 | PF01823.21;PF00057.20;PF00090.21 | extracellular | 267.6236877 | 488.1818542 | 342.649292 | 377.3456421 | 292.8585815 | 275.502655 |
| A0A452EU19 | LOC102175560 | A0A452EU19_CAPHI Alpha-2-macroglobulin OS=Capra hircus OX=9925 GN=LOC102175560 PE=3 SV=1 | CC:extracellular space;CC:cellular_component;CC:cellular anatomical entityMF:endopeptidase inhibitor activity;MF:protein binding;MF:endopeptidase regulator activity;MF:peptidase regulator activity;MF:enzyme binding;MF:peptidase inhibitor activity;MF:molec | K03910;K06530;K23589;K23593 | ENOG410XQIV;ENOG410XRED | PF07678.16;PF00207.24;PF07703.16;PF07677.16;PF17789.3;PF17791.3;PF01835.21;PF02369.18 | extracellular | 74666.21094 | 84653.75 | 82570.55469 | 85862 | 74284.63281 | 91376.64844 |
| A0A452ELE7 | C2 | A0A452ELE7_CAPHI Complement C2 OS=Capra hircus OX=9925 GN=C2 PE=4 SV=1 | BP:regulation of immune system process;BP:regulation of transport;BP:positive regulation of response to stimulus;BP:regulation of response to stimulus;BP:humoral immune response;BP:regulation of biological process;BP:positive regulation of phagocytosis;BP | K01332;K17495;K01335 | COG5640 | PF00092.30;PF00089.28;PF00084.22;PF13519.8 | extracellular | 285.4934998 | 167.7982941 | 314.6237488 | 257.806488 | 244.6264191 | 222.5751343 |
| A0A452DY37 | B2M | A0A452DY37_CAPHI Beta-2-microglobulin OS=Capra hircus OX=9925 GN=B2M PE=3 SV=1 | BP:regulation of neuron projection development;BP:regulation of metabolic process;BP:protein homotetramerization;BP:regulation of transport;BP:positive regulation of response to stimulus;BP:regulation of response to stimulus;BP:negative regulation of epit | K08055;K06752 | ENOG410YTDG | PF07654.17 | extracellular | 778.2866821 | 1131.048218 | 1200.645996 | 1176.574707 | 1302.655884 | 1575.714355 |
| A0A452G9K5 | KRT1 | A0A452G9K5_CAPHI Keratin, type II cytoskeletal 1 OS=Capra hircus OX=9925 GN=KRT1 PE=3 SV=1 | BP:negative regulation of response to stimulus;BP:positive regulation of response to stimulus;BP:regulation of response to stimulus;BP:negative regulation of defense response;BP:establishment of skin barrier;BP:regulation of defense response;BP:negative r | K07605 | ENOG410YY6B | PF00038.23;PF16208.7;PF16210.7 | cytoplasmic | 321.9877625 | 970.5211792 | 645.2238159 | 714.2606812 | 285.85672 | 2157.914551 |
| A0A452FV98 | ITIH2 | A0A452FV98_CAPHI Inter-alpha-trypsin inhibitor heavy chain 2 OS=Capra hircus OX=9925 GN=ITIH2 PE=3 SV=1 | BP:glycosaminoglycan metabolic process;BP:mucopolysaccharide metabolic process;BP:metabolic process;BP:nitrogen compound metabolic process;BP:organic substance metabolic process;BP:hyaluronan metabolic process;BP:biological_process;BP:organonitrogen compo | K24513;K19014;K19015;K24515;K24514 | COG2304 | PF06668.14;PF08487.12;PF00092.30;PF13768.8;PF13519.8 | cytoplasmic | 5088.071289 | 7097.54834 | 6005.559082 | 4771.083496 | 4871.493164 | 4589.798828 |
| A0A836D7Y8 | JEQ12_001972 | A0A836D7Y8_SHEEP CN hydrolase domain-containing protein OS=Ovis aries OX=9940 GN=JEQ12_001972 PE=3 SV=1 | BP:nitrogen compound metabolic process;BP:metabolic process;BP:biological_processMF:hydrolase activity;MF:hydrolase activity, acting on carbon-nitroge;MF:hydrolase activity, acting on carbon-nitroge;MF:catalytic activity;MF:molecular_function | K08069;K01435 | COG0388 | PF19018.2;PF00795.24 | lysosomal | 62.04465103 | 67.95002747 | 112.3871307 | 37.40616226 | 35.66310883 | 44.24000931 |
| A0A836A057 | JEQ12_007518 | A0A836A057_SHEEP Ig-like domain-containing protein OS=Ovis aries OX=9940 GN=JEQ12_007518 PE=4 SV=1 | ------ | K06553 | ENOG410ZN0R | PF07686.19;PF13927.8 | extracellular | 10893.39453 | 6423.181152 | 10901.09082 | 5882.662598 | 8404.857422 | 3622.531982 |
| A0A452EQW7 | MDH1 | A0A452EQW7_CAPHI Malate dehydrogenase OS=Capra hircus OX=9925 GN=MDH1 PE=3 SV=1 | BP:carboxylic acid metabolic process;BP:tricarboxylic acid cycle;BP:organic substance metabolic process;BP:dicarboxylic acid metabolic process;BP:cellular process;BP:biological_process;BP:NADH metabolic process;BP:metabolic process;BP:oxoacid metabolic pr | K00025 | COG0039 | PF02866.20;PF00056.25 | cytoplasmic | 0 | 5.319169521 | 16.46225739 | 9.30722332 | 5.219877243 | 8.858073235 |
| A0A836D501 | JEQ12_014353 | A0A836D501_SHEEP IF rod domain-containing protein OS=Ovis aries OX=9940 GN=JEQ12_014353 PE=3 SV=1 | CC:cellular_component;CC:supramolecular fiber;CC:polymeric cytoskeletal fiber;CC:keratin filament;CC:supramolecular complex;CC:supramolecular polymer;CC:intermediate filament;CC:cellular anatomical entity | K07605 | ENOG410YY6B;ENOG410XUTQ | PF00038.23;PF16208.7;PF07926.14;PF10473.11 | cytoplasmic | 213.6162415 | 651.9467163 | 405.3700867 | 320.4953003 | 133.8460388 | 2161.688232 |
| P29701 | AHSG | FETUA_SHEEP Alpha-2-HS-glycoprotein OS=Ovis aries OX=9940 GN=AHSG PE=1 SV=1 | BP:regulation of primary metabolic process;BP:regulation of metabolic process;BP:regulation of transport;BP:regulation of response to stimulus;BP:ossification;BP:regulation of bone mineralization;BP:negative regulation of bone mineralization;BP:regulation | K23411;K23409 | ENOG4111RIP | PF00031.23 | extracellular | 30559.39453 | 36138.99609 | 28940.4082 | 27899.96289 | 27129.82031 | 27313.52734 |
| A0A836A6M4 | JEQ12_013685 | A0A836A6M4_SHEEP Vitellogenin domain-containing protein OS=Ovis aries OX=9940 GN=JEQ12_013685 PE=4 SV=1 | MF:lipid transporter activity;MF:transporter activity;MF:molecular_function | K14462 | ENOG411104F | PF01347.24;PF09172.13;PF06448.13;PF12491.10 | ER | 460.2190552 | 316.4032288 | 557.0734863 | 536.1131592 | 348.8440552 | 371.1195984 |
| A0A452FGV1 | SPP2 | A0A452FGV1_CAPHI Secreted phosphoprotein 24 OS=Capra hircus OX=9925 GN=SPP2 PE=3 SV=1 | BP:multicellular organismal process;BP:tissue remodeling;BP:biological_process;BP:bone remodelingCC:cellular_component;CC:extracellular region;CC:cellular anatomical entity | ------ | ENOG410Z1VS | PF07448.13;PF00666.19 | extracellular | 28.64264107 | 23.41467094 | 7.38090229 | 12.59438896 | 30.38786125 | 14.63209724 |
| A0A452F7K1 | LRRD1 | A0A452F7K1_CAPHI Leucine rich repeats and death domain containing 1 OS=Capra hircus OX=9925 GN=LRRD1 PE=4 SV=1 | BP:positive regulation of response to stimulus;BP:regulation of response to stimulus;BP:positive regulation of Ras protein signal transduction;BP:positive regulation of signaling;BP:signal transduction;BP:regulation of signaling;BP:positive regulation of | ------ | COG4886 | PF13855.8;PF12799.9 | cytoplasmic | 275.4075623 | 319.6201172 | 269.3374939 | 244.4050446 | 239.9572906 | 265.5733948 |
| A0A452E2C0 | TTR | A0A452E2C0_CAPHI Transthyretin OS=Capra hircus OX=9925 GN=TTR PE=3 SV=1 | BP:cellular metabolic process;BP:nucleobase-containing compound metabolic process;BP:cellular nitrogen compound metabolic process;BP:nitrogen compound metabolic process;BP:purine-containing compound metabolic process;BP:retinol metabolic process;BP:cellul | K20731 | COG2351 | PF00576.23 | cytoplasmic | 2014.499756 | 2077.381592 | 1888.643921 | 1999.817017 | 1452.721191 | 1594.736084 |
| A0A452FLL3 | LOC102182869 | A0A452FLL3_CAPHI Amine oxidase OS=Capra hircus OX=9925 GN=LOC102182869 PE=3 SV=1 | BP:nitrogen compound metabolic process;BP:regulation of oxidoreductase activity;BP:negative regulation of catalytic activity;BP:organic substance metabolic process;BP:biological regulation;BP:negative regulation of primary amine oxidase activity;BP:regula | K00276 | COG3733 | PF01179.22;PF02728.18;PF02727.18 | extracellular | 391.1045532 | 492.9851379 | 218.6258698 | 950.142395 | 405.1344604 | 413.6592712 |
| A0A6P3TS98 | JEQ12_003530 | A0A6P3TS98_SHEEP Uncharacterized protein OS=Ovis aries OX=9940 GN=JEQ12_003530 PE=4 SV=1 | BP:positive regulation of catabolic process;BP:regulation of catabolic process;BP:regulation of complement activation;BP:regulation of primary metabolic process;BP:regulation of metabolic process;BP:negative regulation of response to stimulus;BP:regulatio | K04002;K04012 | ENOG410XPJ1 | PF00084.22;PF18453.3 | plasma membrane | 531.0151367 | 248.7633057 | 1115.283813 | 1051.026855 | 762.6713867 | 1213.614502 |
| W5QAR2 | F13A1 | W5QAR2_SHEEP Coagulation factor XIII A chain OS=Ovis aries OX=9940 GN=F13A1 PE=3 SV=1 | BP:peptide cross-linking;BP:nitrogen compound metabolic process;BP:cellular macromolecule metabolic process;BP:protein activation cascade;BP:blood coagulation, fibrin clot formation;BP:organic substance metabolic process;BP:cellular protein metabolic proc | K05619;K05624;K05620;K05622;K03917 | ENOG410XQEZ | PF00927.24;PF00868.22;PF01841.21 | cytoplasmic | 34.96611023 | 76.43249512 | 70.8057251 | 94.44887543 | 34.24497223 | 62.31768036 |
| A0A452G129 | GC | A0A452G129_CAPHI Vitamin D-binding protein OS=Capra hircus OX=9925 GN=GC PE=4 SV=1 | BP:fat-soluble vitamin metabolic process;BP:organic cyclic compound metabolic process;BP:organic substance metabolic process;BP:small molecule metabolic process;BP:vitamin metabolic process;BP:lipid metabolic process;BP:biological_process;BP:metabolic pro | K16141;K12258;K16144 | ENOG4111PH6 | PF00273.22;PF09164.12 | extracellular | 17291.49023 | 19130.25391 | 18243.42969 | 20253.14258 | 15050.94727 | 16838.76367 |
| W5QH50 | HRG | W5QH50_SHEEP Histidine rich glycoprotein OS=Ovis aries OX=9940 GN=HRG PE=4 SV=1 | MF:endopeptidase inhibitor activity;MF:enzyme regulator activity;MF:peptidase regulator activity;MF:molecular_function;MF:peptidase inhibitor activity;MF:cysteine-type endopeptidase inhibitor activity;MF:molecular function regulator;MF:endopeptidase regul | K23411;K23410 | ENOG41117FH | PF00031.23 | extracellular | 1687.561157 | 4453.851074 | 2032.025269 | 1082.684692 | 976.8716431 | 2147.646973 |
| A0A452G3K3 | LOC102177641 | A0A452G3K3_CAPHI Spleen trypsin inhibitor I OS=Capra hircus OX=9925 GN=LOC102177641 PE=4 SV=1 | CC:cellular_component;CC:cellular anatomical entity;CC:extracellular spaceMF:endopeptidase inhibitor activity;MF:endopeptidase regulator activity;MF:peptidase regulator activity;MF:molecular_function;MF:peptidase inhibitor activity;MF:serine-type endopept | ------ | ENOG410ZW2A | PF00014.25 | extracellular | 59.840271 | 97.39820862 | 68.70879364 | 24.92626762 | 145.9650879 | 67.41255951 |
| H6WVW6 | SEPP1 | H6WVW6_CAPHI Selenoprotein P OS=Capra hircus OX=9925 GN=SEPP1 PE=2 SV=1 | BP:cellular process;BP:cellular metabolic process;BP:biological_process;BP:metabolic process;BP:selenium compound metabolic processCC:cellular_component;CC:extracellular region;CC:cellular anatomical entityMF:selenium binding;MF:binding;MF:molecular_funct | ------ | ENOG4111JK7 | PF04592.16;PF04593.16 | extracellular | 57.69314194 | 55.41395187 | 48.23661041 | 53.5067215 | 39.66489792 | 32.5174675 |
| A0A835ZZR0 | JEQ12_007500 | A0A835ZZR0_SHEEP Ig lambda chain V-I region BL2 OS=Ovis aries OX=9940 GN=JEQ12_007500 PE=4 SV=1 | ------ | K06553 | ------ | PF07686.19;PF13927.8;PF07679.18;PF00047.27 | extracellular | 1638.311646 | 817.7207642 | 2008.431763 | 1881.25647 | 592.9005127 | 1392.315796 |
| A0A452DYF3 | LOC102171351 | A0A452DYF3_CAPHI BPI1 domain-containing protein OS=Capra hircus OX=9925 GN=LOC102171351 PE=4 SV=1 | CC:intracellular membrane-bounded organelle;CC:secretory vesicle;CC:secretory granule;CC:cellular_component;CC:intracellular organelle;CC:intracellular vesicle;CC:cytoplasmic vesicle;CC:membrane-bounded organelle;CC:organelle;CC:cellular anatomical entity | ------ | ENOG4111BIC | PF01273.27 | cytoplasmic | 51594.48047 | 17503.36719 | 9181.925781 | 13647.39648 | 4018.351563 | 15028.73047 |
| A0A835ZVB8 | JEQ12_010483 | A0A835ZVB8_SHEEP Complement factor B OS=Ovis aries OX=9940 GN=JEQ12_010483 PE=4 SV=1 | BP:positive regulation of response to stimulus;BP:regulation of response to stimulus;BP:regulation of biological process;BP:response to external biotic stimulus;BP:interspecies interaction between organisms;BP:positive regulation of immune system process; | K01332;K24469;K17495;K01335 | COG5640 | PF00092.30;PF00089.28;PF00084.22;PF13519.8;PF09342.13 | extracellular | 2583.46582 | 2717.170898 | 3413.631104 | 2872.47583 | 3462.887451 | 3456.000244 |
| A0A452FW61 |  | A0A452FW61_CAPHI Ig-like domain-containing protein OS=Capra hircus OX=9925 PE=4 SV=1 | ------ | K06553 | ENOG410YNU3;ENOG4111C7P | PF07686.19;PF07679.18;PF13927.8 | plasma membrane | 0 | 297.2798157 | 246.7999268 | 0 | 84.01217651 | 0 |
| W5P0Q4 | LOC101102413 | W5P0Q4_SHEEP Haptoglobin OS=Ovis aries OX=9940 GN=LOC101102413 PE=4 SV=1 | BP:inflammatory response;BP:nitrogen compound metabolic process;BP:signal transduction;BP:cell surface receptor signaling pathway;BP:positive regulation of cell death;BP:regulation of cell death;BP:regulation of biological process;BP:immune system process | K01331;K01330;K16142;K03993;K03992 | COG5640 | PF00089.28 | ER | 0 | 0 | 0 | 0 | 337.7344971 | 0 |
| A0A836D3N6 | JEQ12_015335 | A0A836D3N6_SHEEP Paraoxonase OS=Ovis aries OX=9940 GN=JEQ12_015335 PE=3 SV=1 | BP:regulation of transport;BP:small molecule metabolic process;BP:small molecule catabolic process;BP:organic cyclic compound metabolic process;BP:organophosphate catabolic process;BP:positive regulation of molecular function;BP:positive regulation of bio | K01045 | ENOG4111QK7 | PF01731.22;PF08450.14 | extracellular | 670.0238037 | 1105.605347 | 308.9378967 | 451.1875 | 364.6246033 | 0 |
| A0A452DRJ1 | F12 | A0A452DRJ1_CAPHI Coagulation factor XII OS=Capra hircus OX=9925 GN=F12 PE=4 SV=1 | BP:hemostasis;BP:regulation of metabolic process;BP:negative regulation of response to stimulus;BP:blood coagulation;BP:regulation of response to stimulus;BP:regulation of defense response;BP:regulation of primary metabolic process;BP:positive regulation | K01328;K01343;K08648;K09631;K01348 | COG5640 | PF00089.28;PF00051.20;PF00040.21;PF00008.29;PF00039.20;PF13365.8 | extracellular | 228.9858246 | 321.7108765 | 330.2320862 | 262.7823486 | 299.7450867 | 250.10112 |
| A2P2G3 | VH | A2P2G3_SHEEP VH region (Fragment) OS=Ovis aries OX=9940 GN=VH PE=2 SV=1 | ------ | ------ | ENOG41114KX;ENOG410YNFT | PF07686.19 | extracellular | 158.1585846 | 57.85645294 | 164.9575653 | 105.0974579 | 76.90339661 | 190.1832733 |
| A0A452GAA0 | TFRC | A0A452GAA0_CAPHI Transferrin receptor protein 1 OS=Capra hircus OX=9925 GN=TFRC PE=3 SV=1 | BP:regulation of protein localization;BP:protein localization;BP:regulation of phosphate metabolic process;BP:regulation of primary metabolic process;BP:regulation of metabolic process;BP:positive regulation of response to stimulus;BP:regulation of respon | K14592;K23910;K01301;K06503 | COG2234 | PF04389.19;PF02225.24;PF04253.17 | Golgi apparatus | 38.92529297 | 28.71694183 | 27.14975357 | 34.16627121 | 27.74240875 | 31.27313995 |
| A0A835ZQF6 | JEQ12_008123 | A0A835ZQF6_SHEEP Immunoglobulin heavy constant mu OS=Ovis aries OX=9940 GN=JEQ12_008123 PE=4 SV=1 | CC:cellular_component;CC:integral component of membrane;CC:cellular anatomical entity;CC:intrinsic component of membrane | K06752;K06551 | ENOG41116WB;ENOG410ZM6W | PF07654.17;PF00047.27;PF08205.14;PF13927.8 | cytoplasmic | 70034.8125 | 51164.76563 | 89363.51563 | 83201.9375 | 37659.25391 | 81362.78125 |
| A0A452EEX6 | CNTLN | A0A452EEX6_CAPHI Centlein OS=Capra hircus OX=9925 GN=CNTLN PE=4 SV=1 | BP:protein localization;BP:cellular localization;BP:cellular macromolecule localization;BP:macromolecule localization;BP:cellular protein localization;BP:cellular process;BP:biological_process;BP:localization;BP:centriole-centriole cohesion;BP:protein loc | K16467 | ENOG410XRNE | ------ | cytoplasmic | 451.453186 | 314.4875183 | 433.6635742 | 478.7264709 | 430.155365 | 381.5512085 |
| A0A836D6U1 | JEQ12_013366 | A0A836D6U1_SHEEP Lipocln_cytosolic_FA-bd_dom domain-containing protein OS=Ovis aries OX=9940 GN=JEQ12_013366 PE=3 SV=1 | BP:positive regulation of response to stimulus;BP:regulation of response to stimulus;BP:regulation of biological process;BP:immune system process;BP:positive regulation of immune system process;BP:biological regulation;BP:positive regulation of biological | K03999;K01830 | ENOG4111Y9C | PF00061.25 | peroxisomal | 103.2168884 | 156.5244598 | 142.3999634 | 109.4476929 | 96.21097565 | 25.7408638 |
| A0A6P3E512 | JEQ12_001512 | A0A6P3E512_SHEEP Uncharacterized protein OS=Ovis aries OX=9940 GN=JEQ12_001512 PE=4 SV=1 | BP:negative regulation of metabolic process;BP:regulation of metabolic process;BP:negative regulation of cellular metabolic process;BP:regulation of cellular metabolic process;BP:regulation of biological process;BP:regulation of primary metabolic process; | K23411;K23410;K23409 | ENOG4111K2W | PF00031.23 | plasma membrane | 4828.796875 | 4355.482422 | 7797.494141 | 4635.864258 | 3812.138184 | 3633.829834 |
| A0A6P3EIG5 | JEQ12_008021 | A0A6P3EIG5_SHEEP Uncharacterized protein OS=Ovis aries OX=9940 GN=JEQ12_008021 PE=3 SV=1 | BP:regulation of metabolic process;BP:male gamete generation;BP:regulation of primary metabolic process;BP:negative regulation of macromolecule metabolic process;BP:negative regulation of molecular function;BP:negative regulation of biological process;BP: | K03913;K04525;K20734 | COG4826 | PF00079.22 | extracellular | 480.3740845 | 670.4329224 | 412.5675049 | 808.7367554 | 526.5732422 | 618.0740356 |
| A0A836AFF8 | JEQ12_017500 | A0A836AFF8_SHEEP Albumin OS=Ovis aries OX=9940 GN=JEQ12_017500 PE=4 SV=1 | BP:response to extracellular stimulus;BP:response to external stimulus;BP:regulation of biological process;BP:maintenance of mitochondrion location;BP:maintenance of organelle location;BP:regulation of cell death;BP:maintenance of location in cell;BP:cell | K16141;K16144;K12258 | ENOG410Z40H | PF00273.22 | cytoplasmic | 128525.8203 | 137040.6563 | 116130.8906 | 139708.7969 | 116442.1953 | 128402.5703 |
| W5PHP7 |  | W5PHP7_SHEEP SERPIN domain-containing protein OS=Ovis aries OX=9940 PE=3 SV=1 | CC:cellular_component;CC:extracellular space;CC:cellular anatomical entity | K04525 | COG4826 | PF00079.22 | mitochondrial | 3554.283691 | 3708.922119 | 2642.218018 | 3128.156006 | 6102.998047 | 4238 |
| A0A452DR78 | TGFBI | A0A452DR78_CAPHI Transforming growth factor-beta-induced protein ig-h3 OS=Capra hircus OX=9925 GN=TGFBI PE=4 SV=1 | BP:external encapsulating structure organization;BP:angiogenesis;BP:cellular component organization or biogenesis;BP:cellular component organization;BP:extracellular structure organization;BP:biological adhesion;BP:developmental process;BP:cell proliferat | K19020;K24790;K19519;K19013 | COG2335 | PF02469.24 | ER | 23.74349403 | 26.71210289 | 29.61108589 | 25.95355415 | 18.66939163 | 39.45336914 |
| A0A836A4R9 | JEQ12_003536 | A0A836A4R9_SHEEP Complement factor H OS=Ovis aries OX=9940 GN=JEQ12_003536 PE=4 SV=1 | BP:positive regulation of response to stimulus;BP:regulation of response to stimulus;BP:regulation of biological process;BP:response to external biotic stimulus;BP:interspecies interaction between organisms;BP:positive regulation of immune system process; | K23817;K23816;K23815;K04004 | ENOG410YBAR | PF00084.22 | extracellular | 3966.316406 | 5311.291016 | 5155.085449 | 4895.987305 | 4700.469727 | 5008.08252 |
| A0A835ZR66 | JEQ12_008131 | A0A835ZR66_SHEEP IGv domain-containing protein (Fragment) OS=Ovis aries OX=9940 GN=JEQ12_008131 PE=4 SV=1 | ------ | ------ | ENOG410YNFT | PF07686.19 | cytoplasmic | 6565.006836 | 5757.055664 | 8027.959961 | 5610.095703 | 3633.581787 | 7502.302734 |
| A0A452DMF8 | KRT5 | A0A452DMF8_CAPHI Keratin 5 OS=Capra hircus OX=9925 GN=KRT5 PE=3 SV=1 | BP:developmental process;BP:negative regulation of epithelial cell proliferation;BP:regulation of biological process;BP:cellular developmental process;BP:biological regulation;BP:epithelial cell differentiation;BP:negative regulation of biological process | K07605 | ENOG410YY6B | PF00038.23;PF16208.7 | mitochondrial | 15.5616436 | 50.02178192 | 15.52764988 | 22.55447006 | 10.00841331 | 194.2811737 |
| W5Q4Q3 |  | W5Q4Q3_SHEEP Serpin family D member 1 OS=Ovis aries OX=9940 PE=3 SV=1 | BP:negative regulation of metabolic process;BP:regulation of primary metabolic process;BP:regulation of metabolic process;BP:negative regulation of cellular metabolic process;BP:regulation of cellular metabolic process;BP:multicellular organismal process; | K03912;K04525 | COG4826 | PF00079.22 | extracellular | 1689.422852 | 2539.526367 | 1843.138184 | 1910.065552 | 2064.183838 | 1900.737427 |
| A0A452E7B7 | CDH13 | A0A452E7B7_CAPHI Cadherin 13 OS=Capra hircus OX=9925 GN=CDH13 PE=4 SV=1 | BP:positive regulation of nucleic acid-templated transcription;BP:regulation of primary metabolic process;BP:regulation of metabolic process;BP:positive regulation of epithelial cell proliferation;BP:regulation of transport;BP:positive regulation of respo | K06809;K06808;K06797;K06796;K06736 | ENOG410XQHI | PF00028.19;PF08758.13;PF17803.3 | cytoplasmic | 9.740315437 | 18.08529472 | 20.04319763 | 22.39122391 | 11.58109856 | 16.28710556 |
| A0A452G2X7 | PLP1 | A0A452G2X7_CAPHI Proteolipid protein 1 OS=Capra hircus OX=9925 GN=PLP1 PE=3 SV=1 | BP:primary metabolic process;BP:regulation of metabolic process;BP:regulation of transport;BP:cell development;BP:cell maturation;BP:central nervous system myelination;BP:small molecule metabolic process;BP:cell surface receptor signaling pathway;BP:small | K17271 | ENOG4110EPW | PF01275.21 | extracellular | 27.19818878 | 0 | 0 | 0 | 0 | 0 |
| A0A8C2SCX6 |  | A0A8C2SCX6_CAPHI C-X-C motif chemokine OS=Capra hircus OX=9925 PE=3 SV=1 | BP:antimicrobial humoral response;BP:movement of cell or subcellular component;BP:leukocyte migration;BP:humoral immune response;BP:cell chemotaxis;BP:antimicrobial humoral immune response mediated by antimicrobial peptide;BP:response to protozoan;BP:immu | K10029;K05506;K05505;K05407 | ENOG4111556;ENOG4111BTH | PF00048.22 | extracellular | 2524.139648 | 2270.824707 | 1928.626587 | 2657.415527 | 2310.375244 | 2711.914551 |
| A0A6P3ES03 | JEQ12_020025 | A0A6P3ES03_SHEEP Uncharacterized protein OS=Ovis aries OX=9940 GN=JEQ12_020025 PE=4 SV=1 | ------ | K16857;K04596;K15412;K17341;K04598 | ENOG410ZHSM | PF00090.21;PF18487.3;PF19028.2 | extracellular | 236.4985657 | 284.6439514 | 306.4217834 | 304.0619507 | 364.3538208 | 377.5193176 |
| A0A452EWN7 | MASP1 | A0A452EWN7_CAPHI Mannan binding lectin serine peptidase 1 OS=Capra hircus OX=9925 GN=MASP1 PE=4 SV=1 | BP:response to external biotic stimulus;BP:interspecies interaction between organisms;BP:innate immune response;BP:defense response;BP:response to stress;BP:biological_process;BP:immune response;BP:response to other organism;BP:response to biotic stimulus | K01331;K03992;K03993;K01330 | COG5640 | PF00089.28;PF00431.22;PF00084.22;PF14670.8;PF07645.17 | extracellular | 91.78432465 | 100.3634567 | 148.8013916 | 90.64081573 | 110.0526657 | 83.13848877 |
| A0A452F074 |  | A0A452F074_CAPHI Immunoglobulin heavy constant mu OS=Capra hircus OX=9925 PE=4 SV=1 | CC:cellular_component;CC:integral component of membrane;CC:cellular anatomical entity;CC:intrinsic component of membrane | K06554 | ENOG410YNB1 | PF07654.17;PF08205.14;PF00047.27;PF13927.8 | cytoplasmic | 196.1624298 | 173.9637756 | 295.9794922 | 214.1991119 | 172.6403961 | 230.8398132 |
| A0A836CX49 | JEQ12_003710 | A0A836CX49_SHEEP Spermidine synthase OS=Ovis aries OX=9940 GN=JEQ12_003710 PE=3 SV=1 | BP:response to stress;BP:immune system process;BP:positive regulation of response to stimulus;BP:polyamine biosynthetic process;BP:polyamine metabolic process;BP:cellular biogenic amine biosynthetic process;BP:cellular nitrogen compound metabolic process; | K00797;K01331;K03992;K03993;K01330 | COG5640 | PF01564.19;PF00089.28;PF00431.22;PF17284.4;PF00084.22;PF07645.17;PF13847.8 | cytoplasmic | 346.443512 | 148.9066772 | 244.60672 | 173.3174438 | 234.8684845 | 177.0970459 |
| A0A452DYV8 | AZGP1 | A0A452DYV8_CAPHI Alpha-2-glycoprotein 1, zinc-binding OS=Capra hircus OX=9925 GN=AZGP1 PE=3 SV=1 | BP:antigen processing and presentation via MHC class Ib;BP:antigen processing and presentation of peptide antigen via MHC class Ib;BP:antigen processing and presentation of endogenous peptide antigen via MHC class Ib;BP:immune system process;BP:antigen pr | K06751 | ENOG4111CJX | PF00129.20;PF07654.17;PF16497.7 | extracellular | 134.0286255 | 39.74762726 | 0 | 107.5945358 | 110.1342239 | 194.5165253 |
| A0A452FTD0 |  | A0A452FTD0_CAPHI Ig-like domain-containing protein OS=Capra hircus OX=9925 PE=4 SV=1 | ------ | K06553 | ENOG410YNU3;ENOG4111C7P | PF07686.19;PF13927.8 | extracellular | 865.6278687 | 1162.486328 | 2571.385742 | 2555.771729 | 1069.19873 | 4647.834473 |
| A0A836D8J7 | JEQ12_008673 | A0A836D8J7_SHEEP Complement C5 OS=Ovis aries OX=9940 GN=JEQ12_008673 PE=4 SV=1 | BP:regulation of metabolic process;BP:negative regulation of response to stimulus;BP:positive regulation of response to stimulus;BP:regulation of response to stimulus;BP:regulation of chemotaxis;BP:negative regulation of chemotaxis;BP:positive regulation | K03990;K03994;K03989 | ENOG410XRED;ENOG410ZFKB | PF07678.16;PF17790.3;PF07703.16;PF00207.24;PF17789.3;PF07677.16;PF01759.23;PF01835.21;PF17791.3;PF01821.20 | extracellular | 5143.12207 | 4464.490723 | 3871.385254 | 4176.698242 | 4578.803223 | 3768.286133 |
| A0A835ZZS3 | JEQ12_007515 | A0A835ZZS3_SHEEP Ig-like domain-containing protein OS=Ovis aries OX=9940 GN=JEQ12_007515 PE=4 SV=1 | ------ | K06553 | ENOG41117U9 | PF07686.19;PF13927.8 | plasma membrane | 301.0984497 | 309.9152527 | 408.5558167 | 677.4321289 | 318.7950439 | 0 |
| A0A452F0C8 | IL1RAP | A0A452F0C8_CAPHI Interleukin 1 receptor accessory protein OS=Capra hircus OX=9925 GN=IL1RAP PE=3 SV=1 | CC:integral component of membrane;CC:cellular_component;CC:intrinsic component of membrane;CC:cellular anatomical entityMF:signaling receptor activity;MF:molecular transducer activity;MF:transmembrane signaling receptor activity;MF:interleukin-1 receptor | K05174;K04723;K05170;K05171;K05173 | ENOG410ZRCQ | PF01582.22;PF18452.3;PF13927.8;PF00047.27;PF13895.8;PF13676.8 | cytoplasmic | 111.1787796 | 94.7750473 | 54.69782257 | 150.717453 | 142.8222809 | 67.67783356 |
| A0A452F2K7 | SOD3 | A0A452F2K7_CAPHI Superoxide dismutase [Cu-Zn] OS=Capra hircus OX=9925 GN=SOD3 PE=3 SV=1 | BP:response to hypoxia;BP:response to stress;BP:biological_process;BP:response to oxygen levels;BP:response to abiotic stimulus;BP:response to decreased oxygen levels;BP:response to stimulusCC:extracellular matrix;CC:intracellular organelle;CC:membrane-bo | K04565;K16627;K04569 | COG2032 | PF00080.22 | extracellular | 29.63969994 | 27.20710945 | 29.09514999 | 29.74841881 | 21.82738686 | 22.78728104 |
| W5QAB1 | HPX | W5QAB1_SHEEP Hemopexin OS=Ovis aries OX=9940 GN=HPX PE=4 SV=1 | BP:regulation of phosphate metabolic process;BP:regulation of primary metabolic process;BP:regulation of metabolic process;BP:positive regulation of response to stimulus;BP:regulation of response to stimulus;BP:porphyrin-containing compound metabolic proc | K01413;K06251;K01388;K18977 | ENOG410XQ5D | PF00045.21 | lysosomal | 37150.69531 | 30906.72461 | 26521.40625 | 31635.01367 | 36201.98828 | 36031.69141 |
| P02075 | HBB | HBB_SHEEP Hemoglobin subunit beta OS=Ovis aries OX=9940 GN=HBB PE=1 SV=2 | CC:macromolecular complex;CC:cellular_component;CC:hemoglobin complexMF:cation binding;MF:oxygen transporter activity;MF:oxygen binding;MF:heme binding;MF:tetrapyrrole binding;MF:organic cyclic compound binding;MF:ion binding;MF:small molecule binding;MF: | K13825;K13823 | COG1018 | PF00042.24 | cytoplasmic | 6542.296875 | 8859.810547 | 11020.55957 | 13242.68555 | 22623.45117 | 5544.255859 |
| A0A452FKG1 | F13B | A0A452FKG1_CAPHI Coagulation factor XIII B chain OS=Capra hircus OX=9925 GN=F13B PE=4 SV=1 | ------ | K23817;K03906;K23816;K04004 | ENOG410YE48 | PF00084.22;PF09014.12 | cytoplasmic | 69.31802368 | 130.8443909 | 157.1363983 | 264.695282 | 49.9463501 | 101.7931137 |
| A0A452EQV9 | CDH5 | A0A452EQV9_CAPHI Cadherin-5 OS=Capra hircus OX=9925 GN=CDH5 PE=4 SV=1 | BP:regulation of microtubule polymerization or depolymerization;BP:negative regulation of microtubule polymerization or depolymerization;BP:regulation of microtubule polymerization;BP:regulation of phosphate metabolic process;BP:negative regulation of mic | K06801;K06800;K06533;K06798;K06807 | ENOG410XQHI | PF00028.19;PF01049.19;PF16184.7 | plasma membrane | 50.09607697 | 74.632164 | 58.8816452 | 68.83386993 | 50.18723297 | 54.45168304 |
| A0A452DSW4 | LOC102168295 | A0A452DSW4_CAPHI Amine oxidase OS=Capra hircus OX=9925 GN=LOC102168295 PE=3 SV=1 | BP:nitrogen compound metabolic process;BP:regulation of oxidoreductase activity;BP:negative regulation of catalytic activity;BP:organic substance metabolic process;BP:biological regulation;BP:negative regulation of primary amine oxidase activity;BP:regula | K00276 | COG3733 | PF01179.22;PF02728.18;PF02727.18 | lysosomal | 158.9966125 | 155.2323456 | 221.738739 | 229.7113953 | 136.6186066 | 140.1273499 |
| D6PX64 | Crisp3 | D6PX64_SHEEP Cysteine-rich secretory protein 3 OS=Ovis aries OX=9940 GN=Crisp3 PE=2 SV=1 | CC:cellular_component;CC:extracellular region;CC:extracellular space;CC:cellular anatomical entity | K24834;K20412;K19919 | COG2340 | PF08562.12;PF00188.28 | extracellular | 91.57375336 | 181.982132 | 154.5076294 | 225.2631226 | 110.0033493 | 221.8651123 |
| A0A452EJM6 |  | A0A452EJM6_CAPHI CD5 antigen-like OS=Capra hircus OX=9925 PE=4 SV=1 | CC:membrane;CC:cellular_component;CC:cellular anatomical entityMF:cargo receptor activity;MF:scavenger receptor activity;MF:molecular_function | ------ | ENOG4110209 | PF00530.20;PF15494.8 | cytoplasmic | 0 | 0 | 12.60268879 | 12.54672146 | 0 | 0 |
| W5Q7J0 | APOB | W5Q7J0_SHEEP Apolipoprotein B OS=Ovis aries OX=9940 GN=APOB PE=4 SV=1 | MF:lipid transporter activity;MF:transporter activity;MF:molecular_function | K14462 | ENOG411104F | PF01347.24;PF09172.13;PF06448.13;PF12491.10 | cytoplasmic | 1389.692627 | 846.0601807 | 1047.380371 | 1011.05304 | 1062.526001 | 861.5307617 |
| A0A452DNI4 | MAN2B1 | A0A452DNI4_CAPHI Alpha-mannosidase OS=Capra hircus OX=9925 GN=MAN2B1 PE=3 SV=1 | BP:small molecule metabolic process;BP:mannose metabolic process;BP:organic substance metabolic process;BP:hexose metabolic process;BP:biological_process;BP:metabolic process;BP:primary metabolic process;BP:carbohydrate metabolic process;BP:monosaccharide | K01231;K12311;K12312 | ENOG410XQMZ | PF01074.24;PF07748.15;PF17677.3;PF09261.13 | ER | 9.679849625 | 19.82915878 | 22.46186066 | 23.30334473 | 30.26471329 | 28.73361588 |
| A0A452DL28 | F7 | A0A452DL28_CAPHI Coagulation factor VII OS=Capra hircus OX=9925 GN=F7 PE=4 SV=1 | BP:hemostasis;BP:positive regulation of response to external stimulus;BP:regulation of response to external stimulus;BP:positive regulation of cell communication;BP:positive regulation of response to stimulus;BP:regulation of response to stimulus;BP:nitro | K01321;K01320;K01314;K08664;K01344 | COG5640 | PF00089.28;PF00594.22;PF14670.8;PF09342.13;PF00008.29 | extracellular | 68.25015259 | 58.42828751 | 26.63693428 | 35.47730255 | 74.87023163 | 31.48459625 |
| W5QH45 | KNG1 | W5QH45_SHEEP Kininogen 1 OS=Ovis aries OX=9940 GN=KNG1 PE=4 SV=1 | BP:hemostasis;BP:regulation of primary metabolic process;BP:regulation of metabolic process;BP:negative regulation of response to stimulus;BP:blood coagulation;BP:regulation of response to stimulus;BP:negative regulation of cell adhesion;BP:circulatory sy | K03898;K13903 | ENOG4111ZQ8 | PF00031.23;PF00666.19 | plasma membrane | 5001.85498 | 5976.744141 | 5814.942871 | 5891.047852 | 6120.358887 | 6041.116699 |
| A0A835ZLM5 | JEQ12_013082 | A0A835ZLM5_SHEEP Angiotensinogen OS=Ovis aries OX=9940 GN=JEQ12_013082 PE=3 SV=1 | BP:regulation of tube size;BP:regulation of systemic arterial blood pressure;BP:regulation of systemic arterial blood pressure by hormone;BP:multicellular organismal process;BP:regulation of blood vessel diameter;BP:regulation of systemic arterial blood p | K03913;K04525;K09821 | COG4826 | PF00079.22 | extracellular | 1132.966431 | 904.2068481 | 1180.901489 | 937.8497314 | 1719.80188 | 1220.049561 |
| A0A835ZYK2 | JEQ12_008024 | A0A835ZYK2_SHEEP SERPIN domain-containing protein OS=Ovis aries OX=9940 GN=JEQ12_008024 PE=3 SV=1 | CC:cellular_component;CC:extracellular space;CC:cellular anatomical entity | K04525 | COG4826 | PF00079.22 | extracellular | 4128.327148 | 11264.82422 | 9463.325195 | 17751.58203 | 20321.2207 | 8072.82373 |
| W5P4C6 | F12 | W5P4C6_SHEEP Coagulation factor XII OS=Ovis aries OX=9940 GN=F12 PE=4 SV=1 | BP:hemostasis;BP:regulation of metabolic process;BP:negative regulation of response to stimulus;BP:blood coagulation;BP:regulation of response to stimulus;BP:regulation of defense response;BP:regulation of primary metabolic process;BP:positive regulation | K01328;K01343;K08648;K09631;K01348 | COG5640 | PF00089.28;PF00040.21;PF00051.20;PF00008.29;PF00039.20;PF13365.8 | extracellular | 145.0142517 | 211.2009277 | 236.9428558 | 185.7414703 | 173.0361633 | 148.9190979 |
| A0A452ELS1 | LOC102186111 | A0A452ELS1_CAPHI SERPIN domain-containing protein OS=Capra hircus OX=9925 GN=LOC102186111 PE=3 SV=1 | CC:cellular_component;CC:extracellular space;CC:cellular anatomical entity | K04525 | COG4826 | PF00079.22 | extracellular | 558.1110229 | 0 | 2369.379883 | 29.39265823 | 333.6769104 | 172.4077148 |
| W5PFF9 |  | W5PFF9_SHEEP Complement factor H OS=Ovis aries OX=9940 PE=4 SV=1 | BP:positive regulation of response to stimulus;BP:regulation of response to stimulus;BP:regulation of biological process;BP:response to external biotic stimulus;BP:interspecies interaction between organisms;BP:positive regulation of immune system process; | K23817;K23816;K23815;K04004 | ENOG410YBAR | PF00084.22 | extracellular | 259.6903076 | 410.9056396 | 1169.082642 | 319.4587402 | 444.5024414 | 568.4841919 |
| A0A8C2P6J0 |  | A0A8C2P6J0_CAPHI IGc1 domain-containing protein OS=Capra hircus OX=9925 PE=4 SV=1 | ------ | ------ | ENOG410YNB1 | PF07654.17 | mitochondrial | 49215.07813 | 50754.96875 | 77945.59375 | 91301.67188 | 43917.28516 | 74208.17969 |
| A0A8C2NQ70 |  | A0A8C2NQ70_CAPHI Ig-like domain-containing protein OS=Capra hircus OX=9925 PE=4 SV=1 | ------ | ------ | ENOG410YQB2;ENOG410ZN1U;ENOG41113FX;ENOG410YS8R | PF07686.19;PF13927.8;PF00047.27;PF07679.18 | cytoplasmic | 79.88445282 | 39.51933289 | 13.6809597 | 416.4079895 | 12.67566299 | 154.3048553 |
| A0A452FQE3 | ME1 | A0A452FQE3_CAPHI Malic enzyme OS=Capra hircus OX=9925 GN=ME1 PE=3 SV=1 | BP:regulation of metabolic process;BP:carboxylic acid metabolic process;BP:organic substance metabolic process;BP:biological regulation;BP:organic acid metabolic process;BP:regulation of NADP metabolic process;BP:cellular process;BP:biological_process;BP: | K00027;K00029 | COG0281 | PF03949.17;PF00390.21 | cytoplasmic | 0 | 16.38254929 | 4.280933857 | 9.783067703 | 5.737884998 | 1.032191038 |
| A0A6P7ED26 | JEQ12_019549 | A0A6P7ED26_SHEEP Carbonic anhydrase OS=Ovis aries OX=9940 GN=JEQ12_019549 PE=3 SV=1 | BP:regulation of peptide transport;BP:angiotensin-activated signaling pathway;BP:regulation of transport;BP:ion homeostasis;BP:signal transduction;BP:positive regulation of transmembrane transport;BP:regulation of oligopeptide transport;BP:homeostatic pro | K01672;K18245 | COG3338 | PF00194.23 | cytoplasmic | 40.07287216 | 85.74331665 | 36.90497589 | 13.41563511 | 67.23239136 | 44.19333267 |
| A0A835ZX73 | JEQ12_008126 | A0A835ZX73_SHEEP Immunoglobulin heavy constant mu OS=Ovis aries OX=9940 GN=JEQ12_008126 PE=4 SV=1 | ------ | K06751;K06554 | ENOG4111XUQ;ENOG410YNB1 | PF07654.17;PF00047.27;PF13927.8;PF08205.14 | cytoplasmic | 85316.5 | 32157.36133 | 47210.46484 | 61588.10547 | 33009.27734 | 43880.83594 |
| A0A286SF73 | CD14 | A0A286SF73_SHEEP Monocyte differentiation antigen CD14 OS=Ovis aries OX=9940 GN=CD14 PE=2 SV=1 | BP:regulation of metabolic process;BP:regulation of transport;BP:response to bacterial lipopeptide;BP:positive regulation of response to stimulus;BP:regulation of response to stimulus;BP:signal transduction;BP:cell surface receptor signaling pathway;BP:ce | K10159;K04391 | ENOG41118N2 | PF12799.9;PF13855.8 | extracellular | 131.0689087 | 177.5756226 | 134.7598572 | 102.7891617 | 127.3601761 | 138.8661041 |
| A0A835ZY90 | JEQ12_007512 | A0A835ZY90_SHEEP Ig-like domain-containing protein OS=Ovis aries OX=9940 GN=JEQ12_007512 PE=4 SV=1 | ------ | K06553 | ENOG41117U9;ENOG410Y9T8;ENOG410ZN0R | PF07686.19;PF07679.18;PF13927.8 | extracellular | 35.15403748 | 256.9711609 | 89.5701828 | 58.59912872 | 148.3885956 | 88.2961731 |
| A0A452E7A0 | PLG | A0A452E7A0_CAPHI Plasminogen OS=Capra hircus OX=9925 GN=PLG PE=3 SV=1 | BP:negative regulation of response to external stimulus;BP:regulation of response to external stimulus;BP:negative regulation of response to stimulus;BP:blood coagulation;BP:regulation of response to stimulus;BP:tissue remodeling;BP:regulation of wound he | K01315;K05460;K01313;K01343;K23441 | COG5640 | PF00051.20;PF00089.28;PF00024.28;PF13365.8 | extracellular | 0 | 0 | 269.973938 | 0 | 155.0331573 | 0 |
| A0A835ZYS5 | JEQ12_008129 | A0A835ZYS5_SHEEP Ig-like domain-containing protein (Fragment) OS=Ovis aries OX=9940 GN=JEQ12_008129 PE=4 SV=1 | ------ | ------ | ------ | PF07686.19 | cytoplasmic | 6.994520664 | 2.500243902 | 5.364285469 | 3.18772912 | 0 | 34.25283432 |
| A0A452DZU0 | LOC102174926 | A0A452DZU0_CAPHI SERPIN domain-containing protein OS=Capra hircus OX=9925 GN=LOC102174926 PE=3 SV=1 | CC:cellular_component;CC:extracellular space;CC:cellular anatomical entity | K04525 | COG4826 | PF00079.22 | mitochondrial | 7260.585938 | 6118.702148 | 4402.10498 | 5211.073242 | 7198.849609 | 5330.345215 |
| A0A836AQP3 | JEQ12_001804 | A0A836AQP3_SHEEP Signal recognition particle receptor subunit beta OS=Ovis aries OX=9940 GN=JEQ12_001804 PE=3 SV=1 | BP:antifungal humoral response;BP:cellular cation homeostasis;BP:antimicrobial humoral response;BP:antibacterial humoral response;BP:transition metal ion transport;BP:ion homeostasis;BP:metal ion homeostasis;BP:cellular chemical homeostasis;BP:iron ion tr | K17283;K12272;K14736;K06569 | ENOG410XQ36 | PF00405.19;PF09439.12;PF12974.9;PF00025.23;PF01926.25 | ER | 62540.83984 | 81140.84375 | 69372.53125 | 84129.10156 | 75989.13281 | 74178.67188 |
| A0A452F2R0 | FBLN1 | A0A452F2R0_CAPHI Fibulin-1 OS=Capra hircus OX=9925 GN=FBLN1 PE=3 SV=1 | BP:cellular process;BP:cellular component organization;BP:biological_process;BP:extracellular matrix organization;BP:extracellular structure organization;BP:external encapsulating structure organization;BP:cellular component organization or biogenesisCC:c | K17341;K06826;K17307 | ENOG410XP6K;ENOG410Y194 | PF07645.17;PF12662.9;PF12947.9;PF14670.8 | extracellular | 220.6966858 | 335.3442993 | 354.2227783 | 290.421814 | 139.2506409 | 338.6430664 |
| A0A452EJD6 | MYOC | A0A452EJD6_CAPHI Myocilin OS=Capra hircus OX=9925 GN=MYOC PE=4 SV=1 | BP:positive regulation of membrane depolarization;BP:positive regulation of cell junction assembly;BP:negative regulation of response to stimulus;BP:positive regulation of response to stimulus;BP:regulation of response to stimulus;BP:myelination in periph | K23027;K04594 | ENOG410YBJJ | PF02191.18 | ER | 12.6467371 | 18.0671711 | 38.93659973 | 7.112085342 | 4.565163612 | 18.36810684 |
| A0A836AC75 | JEQ12_016412 | A0A836AC75_SHEEP Complement C3 OS=Ovis aries OX=9940 GN=JEQ12_016412 PE=4 SV=1 | BP:regulation of phosphate metabolic process;BP:regulation of primary metabolic process;BP:regulation of metabolic process;BP:regulation of transport;BP:positive regulation of response to stimulus;BP:regulation of response to stimulus;BP:cell maturation;B | K03990;K03994;K03989 | ENOG410XRED | PF07678.16;PF01759.23;PF17790.3;PF07677.16;PF07703.16;PF00207.24;PF17789.3;PF01835.21;PF17791.3;PF01821.20 | cytoplasmic | 38812.99609 | 43409.28516 | 48763.30859 | 41275.14453 | 41044.25 | 43559.61719 |
| A0A835ZWE6 | JEQ12_003526 | A0A835ZWE6_SHEEP Ig-like domain-containing protein OS=Ovis aries OX=9940 GN=JEQ12_003526 PE=4 SV=1 | BP:protein localization;BP:detection of chemical stimulus;BP:enzyme linked receptor protein signaling pathway;BP:signal transduction;BP:detection of stimulus involved in sensory perception;BP:detection of chemical stimulus involved in sensory perception;B | K20395;K13073;K06719;K16851 | ENOG41128ZA | PF07686.19;PF13927.8 | Golgi apparatus | 2082.284424 | 42.26489258 | 61.80389786 | 30.47031784 | 44.16106796 | 42.25168228 |
| A0A452FLJ4 | LOC102168428 | A0A452FLJ4_CAPHI Serum amyloid A protein OS=Capra hircus OX=9925 GN=LOC102168428 PE=3 SV=1 | BP:response to stimulus;BP:defense response;BP:acute-phase response;BP:response to stress;BP:biological_process;BP:inflammatory response;BP:acute inflammatory responseCC:cellular_component;CC:high-density lipoprotein particle;CC:plasma lipoprotein particl | K17310 | ENOG410YSBK | PF00277.20 | Golgi apparatus | 27.5928936 | 15.42893219 | 21.44830894 | 21.22598839 | 27.84562874 | 32.88260269 |
| A0A452DTF9 | CLEC3B | A0A452DTF9_CAPHI C-type lectin domain family 3 member B OS=Capra hircus OX=9925 GN=CLEC3B PE=4 SV=1 | BP:bone mineralization;BP:response to organic substance;BP:response to endogenous stimulus;BP:regulation of primary metabolic process;BP:regulation of metabolic process;BP:positive regulation of cellular metabolic process;BP:positive regulation of nitroge | K17520;K17521;K17519;K10068;K03991 | ENOG4111IV3 | PF00059.23 | ER | 661.4837646 | 1133.343262 | 1337.018555 | 956.9655762 | 700.499939 | 809.6691284 |
| A0A835ZYS9 | JEQ12_002759 | A0A835ZYS9_SHEEP Keratin, type I cytoskeletal 14 OS=Ovis aries OX=9940 GN=JEQ12_002759 PE=3 SV=1 | CC:cellular_component;CC:supramolecular fiber;CC:polymeric cytoskeletal fiber;CC:supramolecular complex;CC:supramolecular polymer;CC:intermediate filament;CC:cellular anatomical entityMF:structural molecule activity;MF:molecular_function | K07604 | ENOG410Y9IV;ENOG410YK2A | PF00038.23 | cytoplasmic | 2.323388815 | 14.39910126 | 3.700288296 | 3.015169859 | 0 | 20.46571159 |
| A0A6P3EAG2 | JEQ12_017483 | A0A6P3EAG2_SHEEP Uncharacterized protein OS=Ovis aries OX=9940 GN=JEQ12_017483 PE=4 SV=1 | BP:regulation of respiratory burst;BP:positive regulation of metabolic process;BP:antimicrobial humoral response;BP:antibacterial humoral response;BP:macromolecular complex subunit organization;BP:cellular component assembly;BP:defense response to bacteri | ------ | ENOG410YX96 | PF15097.8 | ER | 20392.87891 | 7521.492676 | 8892.208008 | 9322.260742 | 5125.271484 | 10344.65527 |
| A0A452EKD8 |  | A0A452EKD8_CAPHI IGv domain-containing protein OS=Capra hircus OX=9925 PE=4 SV=1 | ------ | ------ | ENOG410YNFT | PF07686.19 | cytoplasmic | 85.96580505 | 66.97772217 | 98.75485229 | 73.23652649 | 38.57738495 | 145.7712097 |
| A0A836CQ93 | JEQ12_012211 | A0A836CQ93_SHEEP Insulin-like growth factor-binding protein complex acid labile subunit OS=Ovis aries OX=9940 GN=JEQ12_012211 PE=4 SV=1 | CC:insulin-like growth factor ternary complex;CC:growth factor complex;CC:macromolecular complex;CC:cellular_component;CC:insulin-like growth factor binding protein complex | K17256;K16351 | COG4886 | PF13855.8;PF12799.9;PF13306.8;PF01462.20 | extracellular | 579.8061523 | 256.9794312 | 275.6016846 | 371.5133667 | 391.5557556 | 250.1286774 |
| A0A6P7DGJ5 |  | A0A6P7DGJ5_SHEEP Alpha globin chain OS=Ovis aries OX=9940 PE=3 SV=1 | CC:macromolecular complex;CC:hemoglobin complex;CC:cellular_componentMF:cation binding;MF:oxygen transporter activity;MF:oxygen binding;MF:heme binding;MF:transition metal ion binding;MF:tetrapyrrole binding;MF:organic cyclic compound binding;MF:ion bindi | K13827;K13826;K13822 | COG1018 | PF00042.24 | cytoplasmic | 5046.95166 | 7104.066406 | 5981.000977 | 6986.15332 | 12132.75391 | 4865.493164 |
| A0A835ZZU0 | JEQ12_008389 | A0A835ZZU0_SHEEP Inter-alpha-trypsin inhibitor heavy chain H1 OS=Ovis aries OX=9940 GN=JEQ12_008389 PE=3 SV=1 | BP:glycosaminoglycan metabolic process;BP:mucopolysaccharide metabolic process;BP:metabolic process;BP:nitrogen compound metabolic process;BP:organic substance metabolic process;BP:hyaluronan metabolic process;BP:biological_process;BP:organonitrogen compo | K24513;K19014;K19015;K24515;K24514 | COG2304 | PF06668.14;PF08487.12;PF00092.30;PF13768.8;PF13519.8 | extracellular | 4629.45459 | 3574.688965 | 5422.345215 | 3754.880127 | 4036.610596 | 4510.591309 |
| A0A0M4KDI9 | ADIPOQ | A0A0M4KDI9_SHEEP Adiponectin (Fragment) OS=Ovis aries OX=9940 GN=ADIPOQ PE=4 SV=1 | BP:developmental process;BP:response to nitrogen compound;BP:cellular response to endogenous stimulus;BP:cell differentiation;BP:cellular response to chemical stimulus;BP:response to insulin;BP:cellular response to insulin stimulus;BP:fat cell differentia | K24214;K07296;K19479;K24218;K24213 | ENOG4111F5K | PF00386.23;PF01391.20 | cytoplasmic | 763.418457 | 1831.238159 | 1885.370972 | 1921.351807 | 1356.617676 | 1753.105469 |
| P32262 | SERPINC1 | ANT3_SHEEP Antithrombin-III OS=Ovis aries OX=9940 GN=SERPINC1 PE=2 SV=1 | BP:hemostasis;BP:regulation of primary metabolic process;BP:regulation of metabolic process;BP:regulation of response to external stimulus;BP:regulation of blood coagulation, intrinsic pathway;BP:blood coagulation;BP:regulation of response to stimulus;BP: | K03911;K13966;K13963 | COG4826 | PF00079.22 | extracellular | 4860.464355 | 9118.828125 | 6801.229492 | 7039.754395 | 7541.552246 | 6870.453613 |
| W5PZ55 | PPBP | W5PZ55_SHEEP C-X-C motif chemokine OS=Ovis aries OX=9940 GN=PPBP PE=3 SV=1 | BP:movement of cell or subcellular component;BP:leukocyte migration;BP:cell chemotaxis;BP:immune system process;BP:taxis;BP:cell migration;BP:leukocyte chemotaxis;BP:chemotaxis;BP:cellular process;BP:defense response;BP:response to stress;BP:biological_pr | K10029;K05505;K05506 | ENOG4111BTH | PF00048.22 | extracellular | 194.9921722 | 458.1928711 | 366.5940247 | 226.8877716 | 161.5410614 | 182.6078949 |
| W5PWE9 | ALB | W5PWE9_SHEEP Albumin OS=Ovis aries OX=9940 GN=ALB PE=4 SV=1 | BP:response to extracellular stimulus;BP:response to external stimulus;BP:regulation of biological process;BP:maintenance of mitochondrion location;BP:maintenance of organelle location;BP:regulation of cell death;BP:maintenance of location in cell;BP:cell | K16141;K16144;K12258 | ENOG410Z40H | PF00273.22 | extracellular | 12139.95117 | 16675.21484 | 9891.144531 | 19764.13086 | 18790.1543 | 18666.11133 |
| A0A836A7C4 | JEQ12_003887 | A0A836A7C4_SHEEP Antithrombin-III OS=Ovis aries OX=9940 GN=JEQ12_003887 PE=3 SV=1 | BP:hemostasis;BP:regulation of primary metabolic process;BP:regulation of metabolic process;BP:regulation of response to external stimulus;BP:regulation of blood coagulation, intrinsic pathway;BP:blood coagulation;BP:regulation of response to stimulus;BP: | K03911;K13966;K13963 | COG4826 | PF00079.22 | extracellular | 509.9993896 | 1402.508667 | 1419.687744 | 571.0419922 | 589.3538208 | 476.8652344 |
| W5QAA3 | LOC101114226 | W5QAA3_SHEEP Vascular cell adhesion protein 1-like OS=Ovis aries OX=9940 GN=LOC101114226 PE=4 SV=1 | BP:cell-cell adhesion;BP:cellular process;BP:biological_process;BP:cell adhesion;BP:biological adhesionCC:integral component of plasma membrane;CC:integral component of membrane;CC:cellular anatomical entity;CC:cellular_component;CC:intrinsic component of | K06255;K06527;K17341 | ENOG4111F4V | PF05790.17;PF07679.18;PF13927.8;PF00047.27;PF13895.8;PF07686.19;PF18452.3;PF03921.16;PF07654.17 | plasma membrane | 83.47685242 | 71.08020782 | 49.98809052 | 52.98067856 | 58.3053093 | 51.96310043 |
| W5PHI7 | LOC101116892 | W5PHI7_SHEEP Serpin A3-1 OS=Ovis aries OX=9940 GN=LOC101116892 PE=3 SV=1 | BP:negative regulation of metabolic process;BP:regulation of metabolic process;BP:negative regulation of cellular metabolic process;BP:regulation of cellular metabolic process;BP:negative regulation of nitrogen compound metabolic process;BP:regulation of | K04525 | COG4826 | PF00079.22 | cytoplasmic | 98.61006165 | 105.8048096 | 31.63551521 | 51.03590775 | 104.8440933 | 73.99155426 |
| A0A835ZZL2 | JEQ12_003209 | A0A835ZZL2_SHEEP SERPIN domain-containing protein OS=Ovis aries OX=9940 GN=JEQ12_003209 PE=3 SV=1 | BP:positive regulation of response to external stimulus;BP:regulation of response to external stimulus;BP:positive regulation of response to stimulus;BP:regulation of response to stimulus;BP:positive regulation of coagulation;BP:regulation of wound healin | K19614;K23430;K13966;K03983;K04525 | COG4826 | PF00079.22 | extracellular | 3061.047852 | 3248.457275 | 3065.031982 | 2686.162109 | 2820.224854 | 2969.866699 |
| A0A452EN11 | MST1 | A0A452EN11_CAPHI Macrophage stimulating 1 OS=Capra hircus OX=9925 GN=MST1 PE=3 SV=1 | BP:regulation of phosphate metabolic process;BP:regulation of primary metabolic process;BP:regulation of metabolic process;BP:negative regulation of cellular carbohydrate metabolic process;BP:regulation of response to stimulus;BP:regulation of chemotaxis; | K23441;K05460;K01313;K01343;K01315 | COG5640 | PF00051.20;PF00089.28;PF00024.28 | extracellular | 41.73766708 | 43.55369949 | 67.09049225 | 61.69752121 | 14.61117744 | 37.66471863 |
| A0A452FTY4 | LOC102168979 | A0A452FTY4_CAPHI Serum amyloid A protein OS=Capra hircus OX=9925 GN=LOC102168979 PE=3 SV=1 | BP:response to stimulus;BP:defense response;BP:acute-phase response;BP:response to stress;BP:biological_process;BP:inflammatory response;BP:acute inflammatory responseCC:cellular_component;CC:high-density lipoprotein particle;CC:plasma lipoprotein particl | K17310 | ENOG4111APX | PF00277.20 | Golgi apparatus | 431.7795105 | 455.3442383 | 318.3761597 | 183.0925598 | 444.3751526 | 331.5075378 |
| A0A452DVP7 | LCP1 | A0A452DVP7_CAPHI Lymphocyte cytosolic protein 1 OS=Capra hircus OX=9925 GN=LCP1 PE=4 SV=1 | BP:regulation of intracellular protein transport;BP:regulation of transport;BP:regulation of intracellular transport;BP:signal transduction;BP:cell activation involved in immune response;BP:positive regulation of biological process;BP:extracellular struct | K17336;K10381;K17275;K17276;K10382 | COG5069 | PF00307.33;PF00036.34;PF13499.8;PF13405.8;PF13833.8;PF13202.8 | cytoplasmic | 129.192749 | 122.3696518 | 134.9591217 | 119.9527817 | 66.99156952 | 95.43350983 |
| A0A452FI14 | APOA1 | A0A452FI14_CAPHI Apolipoprotein A-I OS=Capra hircus OX=9925 GN=APOA1 PE=3 SV=1 | BP:regulation of transport;BP:small molecule metabolic process;BP:negative regulation of production of molecular mediator of immune response;BP:small molecule biosynthetic process;BP:regulation of phospholipid transport;BP:positive regulation of stress fi | K08757 | ENOG410YGQ6 | PF01442.20;PF12732.9;PF07464.13;PF02601.17 | extracellular | 64771.1875 | 111919.2656 | 91152.77344 | 117938.0313 | 100302.5859 | 95298.19531 |
| D6PZY4 | fH | D6PZY4_SHEEP Factor H (Fragment) OS=Ovis aries OX=9940 GN=fH PE=2 SV=1 | BP:positive regulation of response to stimulus;BP:regulation of response to stimulus;BP:regulation of biological process;BP:response to external biotic stimulus;BP:interspecies interaction between organisms;BP:positive regulation of immune system process; | K23817;K23816;K24469;K04004 | ENOG410YBAR | PF00084.22 | cytoplasmic | 255.8329468 | 227.2181854 | 63.19930649 | 171.4235077 | 113.1763229 | 163.3882904 |
| A0A836CWA4 | JEQ12_008530 | A0A836CWA4_SHEEP Lactotransferrin OS=Ovis aries OX=9940 GN=JEQ12_008530 PE=3 SV=1 | BP:antifungal humoral response;BP:antimicrobial humoral response;BP:antibacterial humoral response;BP:negative regulation of response to stimulus;BP:regulation of response to stimulus;BP:iron ion homeostasis;BP:transition metal ion homeostasis;BP:inorgani | K17283;K14736;K06569 | ENOG410XQ36 | PF00405.19;PF12974.9 | extracellular | 19.06485748 | 7.874635696 | 3.501930475 | 16.29035187 | 10.38606358 | 8.871108055 |
| A0A452FQZ1 | APOF | A0A452FQZ1_CAPHI Apolipoprotein F OS=Capra hircus OX=9925 GN=APOF PE=4 SV=1 | BP:primary metabolic process;BP:lipid metabolic process;BP:sterol metabolic process;BP:alcohol metabolic process;BP:organic substance metabolic process;BP:organic cyclic compound metabolic process;BP:secondary alcohol metabolic process;BP:biological_proce | ------ | ENOG4112458 | PF15148.8 | extracellular | 119.5984344 | 55.31368637 | 55.97812271 | 55.07357407 | 68.0598526 | 41.01349258 |
| A0A452F089 | LOC102183974 | A0A452F089_CAPHI Lipocln_cytosolic_FA-bd_dom domain-containing protein OS=Capra hircus OX=9925 GN=LOC102183974 PE=3 SV=1 | BP:positive regulation of response to stimulus;BP:regulation of response to stimulus;BP:regulation of biological process;BP:immune system process;BP:positive regulation of immune system process;BP:biological regulation;BP:positive regulation of biological | K03999;K01830 | ENOG4111Y9C | PF00061.25 | peroxisomal | 510.989502 | 992.8342896 | 804.5748901 | 797.7989502 | 575.7939453 | 566.7272949 |
| A0A836CQZ4 | JEQ12_013161 | A0A836CQZ4_SHEEP Cytochrome P450 4V2 OS=Ovis aries OX=9940 GN=JEQ12_013161 PE=3 SV=1 | BP:hemostasis;BP:blood coagulation;BP:regulation of body fluid levels;BP:biological regulation;BP:multicellular organismal process;BP:coagulation;BP:biological_process;BP:regulation of biological qualityCC:membrane;CC:cellular anatomical entity;CC:cellula | K01323;K00490;K01324;K07427 | COG5640;COG2124 | PF00089.28;PF00067.24;PF00024.28;PF14295.8;PF13365.8;PF09342.13 | plasma membrane | 385.8143005 | 265.4691162 | 354.7956238 | 339.226532 | 371.5652771 | 371.7462463 |
| A0A452E053 |  | A0A452E053_CAPHI Ig-like domain-containing protein OS=Capra hircus OX=9925 PE=4 SV=1 | ------ | K06553 | ENOG41115Q6;ENOG41113FX;ENOG410YS8R;ENOG410ZN1U | PF07686.19;PF00047.27;PF13927.8 | cytoplasmic | 12921.23145 | 5352.258789 | 5078.674316 | 7482.416992 | 4483.836914 | 10445.45313 |
| A0A452G7E7 | SERPINF1 | A0A452G7E7_CAPHI Serpin family F member 1 OS=Capra hircus OX=9925 GN=SERPINF1 PE=3 SV=1 | BP:negative regulation of metabolic process;BP:regulation of metabolic process;BP:negative regulation of cell proliferation;BP:negative regulation of reproductive process;BP:negative regulation of cellular metabolic process;BP:negative regulation of epith | K19614;K03983;K04525 | COG4826 | PF00079.22 | extracellular | 1115.722046 | 1259.666016 | 1037.275024 | 1140.880493 | 1206.35144 | 1359.985962 |
| A0A3R5SS76 |  | A0A3R5SS76_SHEEP Actg1 OS=Ovis aries OX=9940 PE=2 SV=1 | BP:cell projection organization;BP:plasma membrane bounded cell projection morphogenesis;BP:cellular component morphogenesis;BP:cellular component organization or biogenesis;BP:anatomical structure morphogenesis;BP:cellular developmental process;BP:cellul | K12314;K12315;K05692;K12313 | COG5277 | PF00022.21 | cytoplasmic | 659.2559204 | 384.0026855 | 408.4752808 | 641.0116577 | 442.8925171 | 643.6513062 |
| W5P3R3 | PLG | W5P3R3_SHEEP Plasminogen OS=Ovis aries OX=9940 GN=PLG PE=3 SV=1 | BP:negative regulation of response to external stimulus;BP:regulation of response to external stimulus;BP:negative regulation of response to stimulus;BP:blood coagulation;BP:regulation of response to stimulus;BP:tissue remodeling;BP:regulation of wound he | K01315;K05460;K01313;K01343;K23441 | COG5640 | PF00051.20;PF00089.28;PF00024.28;PF13365.8;PF09342.13 | extracellular | 3787.822021 | 3974.922852 | 4531.241211 | 3864.048828 | 3873.602051 | 3846.292725 |
| A0A836A2H1 | JEQ12_006643 | A0A836A2H1_SHEEP SERPIN domain-containing protein OS=Ovis aries OX=9940 GN=JEQ12_006643 PE=3 SV=1 | BP:negative regulation of metabolic process;BP:negative regulation of response to external stimulus;BP:regulation of primary metabolic process;BP:regulation of metabolic process;BP:regulation of response to external stimulus;BP:negative regulation of resp | K19614;K04001;K03983;K04525 | COG4826 | PF00079.22 | extracellular | 8957.345703 | 7579.219238 | 7678.702148 | 7042.186035 | 8967.162109 | 8878.664063 |
| A0A452ET82 | PKM | A0A452ET82_CAPHI Pyruvate kinase OS=Capra hircus OX=9925 GN=PKM PE=3 SV=1 | BP:regulation of metabolic process;BP:positive regulation of sprouting angiogenesis;BP:regulation of sprouting angiogenesis;BP:regulation of primary metabolic process;BP:positive regulation of macromolecule metabolic process;BP:posttranscriptional regulat | K00873;K12406 | COG0469 | PF00224.23;PF02887.18 | cytoplasmic | 32.22901535 | 32.29312897 | 21.86283493 | 31.78339195 | 36.57936859 | 55.82175446 |
| W5PVL4 | MBL2 | W5PVL4_SHEEP Mannose-binding protein C OS=Ovis aries OX=9940 GN=MBL2 PE=4 SV=1 | BP:regulation of immune system process;BP:anatomical structure homeostasis;BP:regulation of transport;BP:positive regulation of response to stimulus;BP:regulation of response to stimulus;BP:homeostatic process;BP:positive regulation of phagocytosis;BP:imm | K03991;K10068 | ENOG4110NRD;ENOG410YQHR | PF00059.23;PF01391.20 | extracellular | 0 | 0 | 1326.721436 | 0 | 0 | 923.9841919 |
| A0A835ZV94 | JEQ12_003533 | A0A835ZV94_SHEEP Complement decay-accelerating factor OS=Ovis aries OX=9940 GN=JEQ12_003533 PE=4 SV=1 | CC:cellular_component;CC:integral component of membrane;CC:cellular anatomical entity;CC:intrinsic component of membrane | K24469;K04006;K04012 | ENOG410XPJ1 | PF00084.22 | plasma membrane | 1996.684326 | 1506.781738 | 1937.583008 | 1573.459961 | 1799.09314 | 1496.703003 |
| A0A835ZX46 | JEQ12_008023 | A0A835ZX46_SHEEP SERPIN domain-containing protein OS=Ovis aries OX=9940 GN=JEQ12_008023 PE=3 SV=1 | BP:negative regulation of metabolic process;BP:regulation of primary metabolic process;BP:regulation of metabolic process;BP:negative regulation of cellular metabolic process;BP:regulation of cellular metabolic process;BP:regulation of biological process; | K04525 | COG4826 | PF00079.22 | cytoplasmic | 5297.27002 | 2968.111084 | 2881.312256 | 4098.825684 | 4790.48877 | 6670.780762 |
| A0A452FZJ9 | ITIH3 | A0A452FZJ9_CAPHI Inter-alpha-trypsin inhibitor heavy chain 3 OS=Capra hircus OX=9925 GN=ITIH3 PE=3 SV=1 | BP:glycosaminoglycan metabolic process;BP:mucopolysaccharide metabolic process;BP:metabolic process;BP:nitrogen compound metabolic process;BP:organic substance metabolic process;BP:hyaluronan metabolic process;BP:biological_process;BP:organonitrogen compo | K24513;K19014;K19015;K24515;K24514 | COG2304 | PF06668.14;PF08487.12;PF00092.30;PF13768.8;PF13519.8 | extracellular | 5187.066895 | 4089.094482 | 5982.755859 | 3699.900146 | 5019.458984 | 4630.452148 |
| A2P2I0 | VH | A2P2I0_SHEEP VH region (Fragment) OS=Ovis aries OX=9940 GN=VH PE=2 SV=1 | ------ | ------ | ENOG410YNFT;ENOG41114KX | PF07686.19;PF13927.8 | extracellular | 419.9038696 | 544.5566406 | 856.3452759 | 896.4041138 | 210.9114227 | 180.6681366 |
| W5NXP3 | LOC101111083 | W5NXP3_SHEEP Serpin A3-6-like OS=Ovis aries OX=9940 GN=LOC101111083 PE=3 SV=1 | CC:cellular_component;CC:extracellular space;CC:cellular anatomical entity | K04525 | COG4826 | PF00079.22 | cytoplasmic | 35.18345642 | 1402.153198 | 5.164554119 | 4363.239258 | 485.0512695 | 0 |
| A0A835ZMV3 | JEQ12_010479 | A0A835ZMV3_SHEEP C4a anaphylatoxin OS=Ovis aries OX=9940 GN=JEQ12_010479 PE=4 SV=1 | BP:inflammatory response;BP:positive regulation of response to stimulus;BP:regulation of response to stimulus;BP:regulation of biological process;BP:humoral immune response;BP:response to external biotic stimulus;BP:interspecies interaction between organi | K03990;K03989 | ENOG410XRED | PF07678.16;PF00207.24;PF10494.11;PF07677.16;PF07703.16;PF01759.23;PF01835.21;PF17791.3;PF17789.3;PF01821.20 | cytoplasmic | 2204.483154 | 1608.247437 | 1101.026855 | 1548.068726 | 2250.550537 | 1451.272217 |
| A0A452DXI2 | LOC102174188 | A0A452DXI2_CAPHI C-type lectin domain-containing protein OS=Capra hircus OX=9925 GN=LOC102174188 PE=3 SV=1 | BP:cellular component organization or biogenesis;BP:cellular component organization;BP:extracellular structure organization;BP:cellular process;BP:biological_process;BP:extracellular matrix organization;BP:external encapsulating structure organizationCC:e | K03991;K10068 | ENOG410XPJ1 | PF09006.13;PF01391.20;PF00059.23 | extracellular | 99.62139893 | 15.61263275 | 50.75536728 | 52.37178802 | 30.58545876 | 45.62073517 |
| A0A6P3E3J1 | JEQ12_014774 | A0A6P3E3J1_SHEEP Uncharacterized protein OS=Ovis aries OX=9940 GN=JEQ12_014774 PE=4 SV=1 | CC:cellular_component;CC:extracellular region;CC:macromolecular complex;CC:collagen trimer;CC:cellular anatomical entity | K24218;K24213 | ENOG411198C | PF00386.23;PF01391.20 | extracellular | 1333.869019 | 1275.518066 | 1440.202637 | 1253.671875 | 969.9876709 | 904.9366455 |
| A0A452DP89 | ALDOB | A0A452DP89_CAPHI Fructose-bisphosphate aldolase OS=Capra hircus OX=9925 GN=ALDOB PE=3 SV=1 | BP:small molecule metabolic process;BP:cellular component organization or biogenesis;BP:NADH oxidation;BP:positive regulation of molecular function;BP:positive regulation of ATPase activity;BP:ADP metabolic process;BP:monocarboxylic acid metabolic process | K01623 | COG3588 | PF00274.21 | cytoplasmic | 19.34519577 | 65.14974976 | 98.30354309 | 77.21271515 | 33.29366684 | 47.09888077 |
| Q1KYZ7 | HBBA | Q1KYZ7_SHEEP Beta-A globin chain OS=Ovis aries OX=9940 GN=HBBA PE=3 SV=1 | CC:macromolecular complex;CC:cellular_component;CC:hemoglobin complexMF:cation binding;MF:oxygen transporter activity;MF:oxygen binding;MF:heme binding;MF:tetrapyrrole binding;MF:organic cyclic compound binding;MF:ion binding;MF:small molecule binding;MF: | K13825;K13823 | COG1018 | PF00042.24 | cytoplasmic | 1309.082642 | 1810.654785 | 0 | 58.79233933 | 20.58032608 | 1133.133179 |
| A0A452ES52 | SERPIND1 | A0A452ES52_CAPHI Serpin family D member 1 OS=Capra hircus OX=9925 GN=SERPIND1 PE=3 SV=1 | BP:negative regulation of metabolic process;BP:regulation of primary metabolic process;BP:regulation of metabolic process;BP:negative regulation of cellular metabolic process;BP:regulation of cellular metabolic process;BP:multicellular organismal process; | K23412;K03912;K23425;K04525;K13963 | COG4826 | PF00079.22 | cytoplasmic | 2033.060059 | 2405.709961 | 2260.509766 | 2506.641846 | 2245.358398 | 1959.158203 |
| A0A836A316 | JEQ12_004686 | A0A836A316_SHEEP Tissue factor pathway inhibitor OS=Ovis aries OX=9940 GN=JEQ12_004686 PE=4 SV=1 | MF:endopeptidase inhibitor activity;MF:enzyme regulator activity;MF:peptidase regulator activity;MF:molecular_function;MF:peptidase inhibitor activity;MF:serine-type endopeptidase inhibitor activity;MF:molecular function regulator;MF:endopeptidase regulat | K23421;K23087 | ENOG410ZW2A;ENOG410XQNP | PF00014.25 | extracellular | 443.8665771 | 248.5036621 | 144.5742188 | 202.9755859 | 614.4683228 | 317.291626 |
| W5QDP8 | FBLN1 | W5QDP8_SHEEP Fibulin-1 OS=Ovis aries OX=9940 GN=FBLN1 PE=3 SV=1 | BP:cellular process;BP:cellular component organization;BP:biological_process;BP:extracellular matrix organization;BP:extracellular structure organization;BP:external encapsulating structure organization;BP:cellular component organization or biogenesisCC:c | K17341;K06824;K06826;K17307 | ENOG410Y194;ENOG410XP6K | PF07645.17;PF12662.9;PF12947.9;PF14670.8 | cytoplasmic | 0 | 0 | 90.26138306 | 0 | 0 | 0 |
| A0A452FPN7 | GPX3 | A0A452FPN7_CAPHI Glutathione peroxidase 3 OS=Capra hircus OX=9925 GN=GPX3 PE=3 SV=1 | BP:cellular catabolic process;BP:hydrogen peroxide catabolic process;BP:cellular process;BP:reactive oxygen species metabolic process;BP:response to stress;BP:biological_process;BP:metabolic process;BP:catabolic process;BP:response to oxidative stress;BP: | K00432 | COG0386 | PF00255.21 | ER | 374.1856689 | 435.1409302 | 400.9828186 | 621.6022339 | 335.0950317 | 395.5214539 |
| W5NSA6 | LOC101122940 | W5NSA6_SHEEP Alpha-2-macroglobulin OS=Ovis aries OX=9940 GN=LOC101122940 PE=3 SV=1 | CC:cellular_component;CC:cellular anatomical entity;CC:extracellular spaceMF:endopeptidase inhibitor activity;MF:endopeptidase regulator activity;MF:peptidase regulator activity;MF:molecular_function;MF:peptidase inhibitor activity;MF:serine-type endopept | K03910;K06530;K23589;K23593 | ENOG410XQIV;ENOG410XRED | PF07678.16;PF07703.16;PF00207.24;PF07677.16;PF17791.3;PF17789.3;PF01835.21;PF05326.13;PF02369.18 | extracellular | 29802.46484 | 36723.30078 | 24706.33008 | 27596.1582 | 32537.63086 | 48349.80078 |
| A0A452E6D4 | C5 | A0A452E6D4_CAPHI Complement C5 OS=Capra hircus OX=9925 GN=C5 PE=4 SV=1 | BP:regulation of metabolic process;BP:negative regulation of response to stimulus;BP:positive regulation of response to stimulus;BP:regulation of response to stimulus;BP:regulation of chemotaxis;BP:negative regulation of chemotaxis;BP:positive regulation | K03990;K03994;K03989 | ENOG410XRED;ENOG410ZFKB | PF07678.16;PF17790.3;PF07703.16;PF07677.16;PF00207.24;PF01759.23;PF17789.3;PF01835.21;PF17791.3;PF01821.20 | extracellular | 118.6526108 | 0 | 133.9318848 | 82.29470062 | 272.1838379 | 0 |
| A0A835ZY78 | JEQ12_007497 | A0A835ZY78_SHEEP Ig-like domain-containing protein OS=Ovis aries OX=9940 GN=JEQ12_007497 PE=4 SV=1 | ------ | K06553 | ENOG41117U9;ENOG410ZN0R | PF07686.19;PF00047.27;PF13927.8 | extracellular | 2246.331055 | 2053.54248 | 8008.534668 | 2326.302246 | 1019.351074 | 3285.218018 |
| A0A452FH26 | EFEMP1 | A0A452FH26_CAPHI EGF containing fibulin extracellular matrix protein 1 OS=Capra hircus OX=9925 GN=EFEMP1 PE=4 SV=1 | BP:embryonic morphogenesis;BP:post-embryonic animal organ morphogenesis;BP:anatomical structure development;BP:regulation of nucleobase-containing compound metabolic process;BP:regulation of primary metabolic process;BP:regulation of metabolic process;BP: | K24335;K17340;K18262;K19866;K17342 | ENOG410YCRW | PF07645.17;PF12662.9;PF12947.9;PF14670.8;PF00008.29 | extracellular | 100.6420898 | 112.5489197 | 106.9451294 | 124.8215408 | 97.26796722 | 112.1701508 |
| A0A836D4P6 | JEQ12_014972 | A0A836D4P6_SHEEP Alpha-2-macroglobulin OS=Ovis aries OX=9940 GN=JEQ12_014972 PE=3 SV=1 | CC:cellular_component;CC:cellular anatomical entity;CC:extracellular spaceMF:endopeptidase inhibitor activity;MF:endopeptidase regulator activity;MF:peptidase regulator activity;MF:molecular_function;MF:peptidase inhibitor activity;MF:serine-type endopept | K03910;K06530;K23589;K23593 | ENOG410XQIV;ENOG410XRED | PF07678.16;PF07677.16;PF07703.16;PF00207.24;PF17791.3;PF17789.3;PF01835.21;PF02369.18 | extracellular | 659.0501709 | 719.6114502 | 950.0189819 | 739.5264893 | 671.7088013 | 703.086731 |
| A0A452G2F0 | IGFBP2 | A0A452G2F0_CAPHI Insulin-like growth factor-binding protein 2 OS=Capra hircus OX=9925 GN=IGFBP2 PE=4 SV=1 | BP:positive regulation of cell proliferation;BP:regulation of activated T cell proliferation;BP:regulation of cell activation;BP:positive regulation of cell activation;BP:regulation of response to stimulus;BP:regulation of T cell activation;BP:regulation | K23575;K23576;K23577;K23578;K10138 | ENOG4111GWQ | PF00086.20;PF00219.20 | extracellular | 37.36155319 | 167.7292938 | 129.5415192 | 93.82241058 | 25.6925354 | 116.0978775 |
| A0A6P3TM89 | LOC101113728 | A0A6P3TM89_SHEEP Clusterin OS=Ovis aries OX=9940 GN=LOC101113728 PE=3 SV=1 | BP:negative regulation of apoptotic signaling pathway;BP:positive regulation of apoptotic signaling pathway;BP:positive regulation of proteasomal ubiquitin-dependent protein catabolic process;BP:regulation of primary metabolic process;BP:regulation of pro | K17252 | ENOG410YYKB | PF01093.19 | extracellular | 3270.305176 | 2533.87085 | 1773.883789 | 2267.888428 | 2465.606934 | 2470.239746 |
| W5P627 | GSN | W5P627_SHEEP Gelsolin OS=Ovis aries OX=9940 GN=GSN PE=4 SV=1 | BP:regulation of organelle organization;BP:cell projection organization;BP:positive regulation of organelle organization;BP:regulation of protein depolymerization;BP:negative regulation of cellular component organization;BP:regulation of cellular componen | K05761;K05768;K08017 | ENOG410XR0A | PF00626.24 | mitochondrial | 4024.712158 | 7282.259277 | 4354.493164 | 6917.29541 | 3948.461914 | 5942.729004 |
| A0A836CXU1 | JEQ12_002712 | A0A836CXU1_SHEEP Amine oxidase OS=Ovis aries OX=9940 GN=JEQ12_002712 PE=3 SV=1 | BP:nitrogen compound metabolic process;BP:response to chemical;BP:regulation of oxidoreductase activity;BP:negative regulation of catalytic activity;BP:organic substance metabolic process;BP:biological regulation;BP:negative regulation of primary amine ox | K00276 | COG3733 | PF01179.22;PF02728.18;PF02727.18;PF01569.23 | lysosomal | 9257.03418 | 7429.427246 | 8702.447266 | 9402.168945 | 10158.16699 | 7812.080566 |
| A0A836D2B5 | JEQ12_017502 | A0A836D2B5_SHEEP Afamin OS=Ovis aries OX=9940 GN=JEQ12_017502 PE=4 SV=1 | BP:macromolecule localization;BP:protein localization;BP:protein stabilization;BP:vitamin transport;BP:nitrogen compound transport;BP:biological regulation;BP:organic substance transport;BP:regulation of biological quality;BP:transport;BP:biological_proce | K16141;K16144;K12258 | ENOG410Z40H | PF00273.22 | extracellular | 1557.676392 | 1348.66272 | 1426.636475 | 1352.824585 | 1599.694336 | 1764.999146 |
| A0A835ZZ97 | JEQ12_007053 | A0A835ZZ97_SHEEP Fibrinogen alpha chain OS=Ovis aries OX=9940 GN=JEQ12_007053 PE=4 SV=1 | BP:negative regulation of apoptotic signaling pathway;BP:positive regulation of heterotypic cell-cell adhesion;BP:regulation of extrinsic apoptotic signaling pathway;BP:negative regulation of extrinsic apoptotic signaling pathway;BP:regulation of secretio | K03903 | ENOG410ZYS4 | PF08702.12;PF12160.10 | extracellular | 834.5004883 | 504.0403748 | 573.3145752 | 683.2471924 | 555.9197998 | 660.479187 |
| W5Q0X5 | LOC101115576 | W5Q0X5_SHEEP Serpin A3-5 OS=Ovis aries OX=9940 GN=LOC101115576 PE=3 SV=1 | CC:cellular_component;CC:extracellular space;CC:cellular anatomical entity | K04525 | COG4826 | PF00079.22 | cytoplasmic | 4613.931152 | 4335.499512 | 3558.897705 | 4169.932129 | 6582.11377 | 3287.394043 |
| A0A452DR85 | LOC102181791 | A0A452DR85_CAPHI Ribosomal protein OS=Capra hircus OX=9925 GN=LOC102181791 PE=3 SV=1 | BP:protein localization;BP:regulation of metabolic process;BP:organic cyclic compound metabolic process;BP:organic cyclic compound catabolic process;BP:negative regulation of macromolecule metabolic process;BP:cellular macromolecule localization;BP:peptid | K02865 | COG0081 | PF00687.23 | nuclear | 50.54753876 | 55.73420715 | 52.11231232 | 13.88221264 | 47.14740753 | 61.38126373 |
| A0A836CWY9 | JEQ12_003532 | A0A836CWY9_SHEEP C4b-binding protein alpha chain OS=Ovis aries OX=9940 GN=JEQ12_003532 PE=4 SV=1 | ------ | K04002;K04007 | ENOG4111BC4 | PF00084.22;PF18453.3 | cytoplasmic | 3961.737549 | 6503.77002 | 6874.197754 | 4049.243164 | 2343.754395 | 3470.699951 |
| A0A452F0G8 | LTA4H | A0A452F0G8_CAPHI Leukotriene A(4) hydrolase OS=Capra hircus OX=9925 GN=LTA4H PE=3 SV=1 | BP:small molecule biosynthetic process;BP:carboxylic acid metabolic process;BP:organic acid biosynthetic process;BP:cellular catabolic process;BP:cellular nitrogen compound metabolic process;BP:nitrogen compound metabolic process;BP:cellular metabolic pro | K01260;K01254;K01257;K09605;K08776 | COG0308 | PF01433.22;PF09127.13;PF17900.3 | cytoplasmic | 15.46109486 | 17.06406593 | 15.97734261 | 9.303962708 | 5.697757244 | 13.37613869 |
| A0A6P3TKJ3 | C1S | A0A6P3TKJ3_SHEEP Complement C1s OS=Ovis aries OX=9940 GN=C1S PE=4 SV=1 | BP:positive regulation of response to stimulus;BP:regulation of response to stimulus;BP:regulation of biological process;BP:response to external biotic stimulus;BP:interspecies interaction between organisms;BP:positive regulation of immune system process; | K03992;K01331;K03993;K01330 | COG5640 | PF00089.28;PF00431.22;PF00084.22;PF14670.8;PF07645.17 | plasma membrane | 44.42019272 | 79.97535706 | 34.20221329 | 38.30969238 | 33.88740158 | 38.25882721 |
| A0A836CYF6 | JEQ12_003342 | A0A836CYF6_SHEEP C-C motif chemokine OS=Ovis aries OX=9940 GN=JEQ12_003342 PE=3 SV=1 | BP:cytokine-mediated signaling pathway;BP:positive regulation of response to stimulus;BP:regulation of response to stimulus;BP:signal transduction;BP:cell surface receptor signaling pathway;BP:cell chemotaxis;BP:cellular response to stimulus;BP:taxis;BP:r | K05408;K12499;K12964 | ENOG41116XA | PF00048.22 | extracellular | 317.3291626 | 54.95524216 | 93.59242249 | 62.70944595 | 130.4958801 | 122.472702 |
| A0A836A854 | JEQ12_016306 | A0A836A854_SHEEP Complement C3 OS=Ovis aries OX=9940 GN=JEQ12_016306 PE=4 SV=1 | BP:positive regulation of response to stimulus;BP:regulation of response to stimulus;BP:regulation of biological process;BP:response to external biotic stimulus;BP:interspecies interaction between organisms;BP:positive regulation of immune system process; | K03990;K03994;K03989;K11150 | ENOG410XRED | PF07678.16;PF00106.27;PF00207.24;PF07677.16;PF13561.8;PF17790.3;PF07703.16;PF17789.3;PF01759.23;PF17791.3;PF01835.21;PF08659.12;PF01821.20 | cytoplasmic | 584.2659302 | 284.4470215 | 645.4795532 | 830.4726563 | 748.4849243 | 718.2543945 |
| A0A6P3E7P3 | JEQ12_001284 | A0A6P3E7P3_SHEEP Vitamin K-dependent protein S OS=Ovis aries OX=9940 GN=JEQ12_001284 PE=4 SV=1 | BP:negative regulation of response to external stimulus;BP:regulation of response to external stimulus;BP:negative regulation of response to stimulus;BP:blood coagulation;BP:regulation of response to stimulus;BP:regulation of wound healing;BP:negative reg | K01314;K01344;K05464;K03908 | ENOG410ZTGU | PF00054.25;PF02210.26;PF14670.8;PF07645.17;PF00594.22;PF12662.9;PF00008.29;PF12947.9 | plasma membrane | 545.2723999 | 542.6138306 | 723.9596558 | 613.2762451 | 731.7700806 | 528.8865356 |
| A0A452EW49 | LOC102172488 | A0A452EW49_CAPHI Inhibitor of carbonic anhydrase OS=Capra hircus OX=9925 GN=LOC102172488 PE=3 SV=1 | CC:extracellular space;CC:cellular_component;CC:cellular anatomical entityMF:enzyme regulator activity;MF:molecular_function;MF:enzyme inhibitor activity;MF:molecular function regulator | K17283;K14736;K06569 | ENOG410XQ36 | PF00405.19;PF12974.9 | extracellular | 460.0645752 | 656.9365234 | 656.4203491 | 677.5081787 | 1113.737061 | 814.0130615 |
| W5PDE5 | LOC101120001 | W5PDE5_SHEEP Apolipoprotein R-like OS=Ovis aries OX=9940 GN=LOC101120001 PE=4 SV=1 | ------ | K04002;K04007 | ENOG4111BC4 | PF00084.22;PF18453.3 | plasma membrane | 4650.599121 | 2338.131592 | 3855.816162 | 2773.248291 | 4665.415527 | 3262.683838 |
| A0A452EJ90 | LCAT | A0A452EJ90_CAPHI Lecithin-cholesterol acyltransferase OS=Capra hircus OX=9925 GN=LCAT PE=3 SV=1 | BP:small molecule metabolic process;BP:cellular component organization or biogenesis;BP:macromolecular complex remodeling;BP:protein-lipid complex remodeling;BP:plasma lipoprotein particle remodeling;BP:secondary alcohol metabolic process;BP:glycerolipid | K00650;K06129 | ENOG410Y9CF | PF02450.17 | lysosomal | 568.6326904 | 343.3883362 | 253.3097229 | 442.6875916 | 362.4618225 | 249.5337219 |
| A0A452F6V7 | CFD | A0A452F6V7_CAPHI Complement factor D OS=Capra hircus OX=9925 GN=CFD PE=4 SV=1 | BP:positive regulation of response to stimulus;BP:regulation of response to stimulus;BP:signal transduction;BP:cell surface receptor signaling pathway;BP:regulation of biological process;BP:Notch signaling pathway;BP:response to external biotic stimulus;B | K01353;K08663;K23011;K01334 | COG5640 | PF00089.28;PF13365.8;PF09342.13 | ER | 29.29925156 | 38.48630142 | 36.4499054 | 42.26937103 | 40.41695404 | 21.53394699 |
| A0A836AE40 | JEQ12_015007 | A0A836AE40_SHEEP Complement C1s subcomponent OS=Ovis aries OX=9940 GN=JEQ12_015007 PE=4 SV=1 | BP:positive regulation of response to stimulus;BP:regulation of response to stimulus;BP:regulation of biological process;BP:response to external biotic stimulus;BP:interspecies interaction between organisms;BP:positive regulation of immune system process; | K03992;K01331;K03993;K01330 | COG5640 | PF00089.28;PF00431.22;PF00084.22;PF14670.8;PF07645.17 | mitochondrial | 358.182251 | 252.1020966 | 355.4702148 | 270.8310242 | 303.6140442 | 280.6075439 |
| A2P2H1 | VH | A2P2H1_SHEEP VH region (Fragment) OS=Ovis aries OX=9940 GN=VH PE=2 SV=1 | ------ | ------ | ENOG410YNFT;ENOG41114KX | PF07686.19;PF13927.8 | cytoplasmic | 424.8554688 | 253.1263428 | 442.1661377 | 495.2080688 | 287.4004822 | 377.6969299 |
| W5PF65 | TF | W5PF65_SHEEP Serotransferrin OS=Ovis aries OX=9940 GN=TF PE=3 SV=1 | BP:regulation of primary metabolic process;BP:regulation of metabolic process;BP:regulation of transport;BP:regulation of RNA biosynthetic process;BP:iron ion homeostasis;BP:iron ion transport;BP:signal transduction;BP:transition metal ion homeostasis;BP: | K17283;K14736;K06569 | ENOG410XQ36 | PF00405.19;PF12974.9 | extracellular | 14564.75488 | 6444.418945 | 5827.671387 | 8924.810547 | 8628.898438 | 5778.279297 |
| A0A452FJU4 | CHIA | A0A452FJU4_CAPHI Chitinase OS=Capra hircus OX=9925 GN=CHIA PE=3 SV=1 | BP:regulation of multicellular organismal process;BP:positive regulation of multicellular organismal process;BP:regulation of chemokine production;BP:nitrogen compound metabolic process;BP:multicellular organismal process;BP:cell death;BP:production of mo | K01183;K17523;K17526 | COG3325 | PF00704.30;PF01607.26 | extracellular | 9.839724541 | 85.16690063 | 54.8677597 | 85.05247498 | 38.63809204 | 26.7364502 |
| A0A452EBC3 | LOC106503930 | A0A452EBC3_CAPHI IFI27L1 OS=Capra hircus OX=9925 GN=LOC106503930 PE=3 SV=1 | CC:cellular_component;CC:integral component of membrane;CC:cellular anatomical entity;CC:intrinsic component of membrane | ------ | ENOG410Z2RX | PF06140.15 | extracellular | 70.15676117 | 38.20009232 | 44.43155289 | 13.56134129 | 31.03777123 | 84.44911194 |
| A0A452DXH4 |  | A0A452DXH4_CAPHI Ig-like domain-containing protein OS=Capra hircus OX=9925 PE=4 SV=1 | ------ | K06553 | ENOG410ZN0R | PF07686.19;PF13927.8 | cytoplasmic | 63.99285889 | 401.3685303 | 511.2609558 | 33.20666504 | 309.8774109 | 93.4841156 |
| A0A836CYT8 | JEQ12_002363 | A0A836CYT8_SHEEP Beta-2-glycoprotein 1 OS=Ovis aries OX=9940 GN=JEQ12_002363 PE=4 SV=1 | CC:cellular_component;CC:extracellular region;CC:cellular anatomical entityMF:sulfur compound binding;MF:glycosaminoglycan binding;MF:carbohydrate derivative binding;MF:molecular_function;MF:binding;MF:heparin binding | K06496;K24469;K17305;K17495 | ENOG410XPJ1 | PF00084.22;PF09014.12 | extracellular | 3199.932129 | 3119.305664 | 2295.968018 | 2636.300781 | 3384.230957 | 2858.508789 |
| A0A6P7DB31 | JEQ12_011511 | A0A6P7DB31_SHEEP Retinol-binding protein OS=Ovis aries OX=9940 GN=JEQ12_011511 PE=3 SV=1 | BP:organic hydroxy compound transport;BP:lipid transport;BP:organic substance transport;BP:transport;BP:retinol transport;BP:establishment of localization;BP:localization;BP:biological_process;BP:isoprenoid transport;BP:terpenoid transportCC:extracellular | K18271;K03098 | ENOG4111K1Y | PF00061.25;PF08212.14 | extracellular | 3816.391357 | 3634.123291 | 3827.654297 | 3221.159424 | 3573.693848 | 2898.678711 |
| A0A835ZZW7 | JEQ12_006507 | A0A835ZZW7_SHEEP Prothrombin OS=Ovis aries OX=9940 GN=JEQ12_006507 PE=3 SV=1 | BP:regulation of protein localization;BP:hemostasis;BP:regulation of primary metabolic process;BP:regulation of metabolic process;BP:regulation of transport;BP:negative regulation of response to stimulus;BP:blood coagulation;BP:regulation of sequestering | K01314;K01315;K01344;K23441;K01313 | COG5640 | PF00089.28;PF00051.20;PF09396.12;PF00594.22 | plasma membrane | 4577.922852 | 5439.860352 | 5856.723633 | 5216.650879 | 4880.892578 | 4309.291016 |
| W5NRI1 |  | W5NRI1_SHEEP A2M_N_2 domain-containing protein OS=Ovis aries OX=9940 PE=4 SV=1 | BP:regulation of phosphate metabolic process;BP:regulation of primary metabolic process;BP:regulation of metabolic process;BP:regulation of transport;BP:positive regulation of response to stimulus;BP:regulation of response to stimulus;BP:cell maturation;B | K03990;K03994;K03989 | ENOG410XRED | PF17790.3;PF07703.16;PF17789.3;PF01835.21;PF17791.3 | ER | 893.0322876 | 999.5515747 | 722.1914673 | 594.8428955 | 537.7103271 | 720.0899658 |
| A0A023ZTI7 |  | A0A023ZTI7_CAPHI Insulin-like growth factor II OS=Capra hircus OX=9925 PE=2 SV=1 | BP:regulation of MAPK cascade;BP:regulation of phosphate metabolic process;BP:regulation of primary metabolic process;BP:regulation of metabolic process;BP:positive regulation of response to stimulus;BP:regulation of response to stimulus;BP:ossification;B | K05459;K13769;K04526 | ENOG4111KP2 | PF08365.13;PF00049.20 | ER | 104.6542892 | 189.1529541 | 138.1978607 | 121.5290604 | 111.2298737 | 110.2280121 |
| A0A452EM11 | APOC3 | A0A452EM11_CAPHI Apolipoprotein C-III OS=Capra hircus OX=9925 GN=APOC3 PE=3 SV=1 | BP:regulation of metabolic process;BP:regulation of primary metabolic process;BP:negative regulation of lipid metabolic process;BP:regulation of triglyceride catabolic process;BP:negative regulation of molecular function;BP:negative regulation of biologic | K08759 | ENOG4111APE | PF05778.14;PF04691.14 | extracellular | 1586.959351 | 2372.651611 | 2141.740723 | 3332.619873 | 2146.284668 | 2343.088379 |
| A0A836A713 | JEQ12_013895 | A0A836A713_SHEEP Ig-like domain-containing protein OS=Ovis aries OX=9940 GN=JEQ12_013895 PE=4 SV=1 | ------ | K06553 | ENOG41115Q6 | PF07686.19;PF00047.27;PF13927.8 | cytoplasmic | 27.74681282 | 32.59410095 | 8.644072533 | 5.747300148 | 8.761331558 | 3.30337739 |
| A0A6P7EAS8 | VNN1 | A0A6P7EAS8_SHEEP Pantetheinase-like OS=Ovis aries OX=9940 GN=VNN1 PE=3 SV=1 | BP:negative regulation of apoptotic signaling pathway;BP:positive regulation of T cell differentiation in thymus;BP:regulation of apoptotic signaling pathway;BP:regulation of T cell differentiation in thymus;BP:negative regulation of response to stimulus; | K08069;K01435 | COG0388 | PF19018.2;PF00795.24 | lysosomal | 193.3851471 | 156.0358276 | 278.7547302 | 228.8139801 | 222.7823334 | 178.0008087 |
| W5PXI3 | AFM | W5PXI3_SHEEP Afamin OS=Ovis aries OX=9940 GN=AFM PE=4 SV=1 | BP:macromolecule localization;BP:protein localization;BP:protein stabilization;BP:vitamin transport;BP:nitrogen compound transport;BP:biological regulation;BP:organic substance transport;BP:regulation of biological quality;BP:transport;BP:biological_proce | K16141;K16144;K12258 | ENOG410Z40H | PF00273.22 | extracellular | 83.76862335 | 87.34895325 | 36.82792664 | 0 | 39.60240173 | 0 |
| A0A836D8K5 | JEQ12_008703 | A0A836D8K5_SHEEP Alpha-1-acid glycoprotein OS=Ovis aries OX=9940 GN=JEQ12_008703 PE=3 SV=1 | BP:regulation of immune system process;BP:response to stimulus;BP:defense response;BP:acute-phase response;BP:response to stress;BP:biological regulation;BP:biological_process;BP:inflammatory response;BP:acute inflammatory response;BP:regulation of biolog | K17308 | ENOG4111C49 | PF00061.25 | extracellular | 4929.527344 | 5105.226563 | 3879.035156 | 2913.279541 | 4458.430176 | 12657.35059 |
| A0A452DQP6 | ACTC1 | A0A452DQP6_CAPHI Actin alpha cardiac muscle 1 OS=Capra hircus OX=9925 GN=ACTC1 PE=3 SV=1 | BP:regulation of metabolic process;BP:muscle system process;BP:circulatory system process;BP:heart process;BP:cellular component organization or biogenesis;BP:positive regulation of macromolecule metabolic process;BP:positive regulation of biological proc | K12314;K12315;K05692;K10354;K12313 | COG5277 | PF00022.21 | cytoplasmic | 23.07053375 | 14.82402134 | 12.36722374 | 19.54545021 | 17.41463089 | 26.90364456 |
| W5NYF4 | PGLYRP2 | W5NYF4_SHEEP Peptidoglycan recognition protein 2 OS=Ovis aries OX=9940 GN=PGLYRP2 PE=3 SV=1 | BP:detection of biotic stimulus;BP:regulation of metabolic process;BP:negative regulation of response to stimulus;BP:regulation of response to stimulus;BP:regulation of defense response;BP:negative regulation of macromolecule metabolic process;BP:response | K01446 | ENOG4111PAY | PF01510.27 | extracellular | 78.01213837 | 89.37198639 | 102.058342 | 57.81374741 | 51.25344086 | 59.99463654 |
| A0A452G2G1 | S100A4 | A0A452G2G1_CAPHI Protein S100 OS=Capra hircus OX=9925 GN=S100A4 PE=3 SV=1 | BP:positive regulation of response to stimulus;BP:regulation of response to stimulus;BP:positive regulation of signaling;BP:regulation of biological process;BP:regulation of signaling;BP:positive regulation of cell communication;BP:regulation of cell comm | K23761;K23770;K23762;K23758;K23759 | ENOG4111DV3 | PF01023.21;PF13499.8 | cytoplasmic | 64.54231262 | 27.24791336 | 10.8924427 | 26.66461372 | 2.977428675 | 27.82111168 |
| A0A836A0U1 | JEQ12_002717 | A0A836A0U1_SHEEP Amine oxidase OS=Ovis aries OX=9940 GN=JEQ12_002717 PE=3 SV=1 | BP:nitrogen compound metabolic process;BP:regulation of oxidoreductase activity;BP:negative regulation of catalytic activity;BP:organic substance metabolic process;BP:biological regulation;BP:negative regulation of primary amine oxidase activity;BP:regula | K00276 | COG3733 | PF01179.22;PF02728.18;PF02727.18 | lysosomal | 2.13548255 | 18.33289146 | 10.36017609 | 4.558998585 | 7.040225506 | 31.10224915 |
| A0A452EME0 | KRT14 | A0A452EME0_CAPHI Keratin 14 OS=Capra hircus OX=9925 GN=KRT14 PE=3 SV=1 | CC:cellular_component;CC:supramolecular fiber;CC:polymeric cytoskeletal fiber;CC:supramolecular complex;CC:supramolecular polymer;CC:intermediate filament;CC:cellular anatomical entityMF:structural molecule activity;MF:molecular_function | K07604 | ENOG410Y9IV | PF00038.23 | cytoplasmic | 144.1783295 | 392.980072 | 103.1632614 | 104.7882767 | 60.01996613 | 877.0787964 |
| A0A8C2SEB7 | BAC7 | A0A8C2SEB7_CAPHI Antibacterial protein PR-39 OS=Capra hircus OX=9925 GN=BAC7.5 PE=3 SV=1 | BP:antimicrobial humoral response;BP:defense response to bacterium;BP:defense response to Gram-negative bacterium;BP:antimicrobial humoral immune response mediated by antimicrobial peptide;BP:cytolysis;BP:response to external biotic stimulus;BP:response t | K13916 | ENOG4111DSI;ENOG41119S0 | PF00666.19 | extracellular | 154.5432129 | 44.08430481 | 19.84528732 | 102.1854172 | 116.414917 | 68.76667023 |
| A0A452FR95 | CFB | A0A452FR95_CAPHI Complement factor B OS=Capra hircus OX=9925 GN=CFB PE=4 SV=1 | BP:positive regulation of response to stimulus;BP:regulation of response to stimulus;BP:regulation of biological process;BP:response to external biotic stimulus;BP:interspecies interaction between organisms;BP:positive regulation of immune system process; | K01332;K24469;K17495;K01335 | COG5640 | PF00089.28;PF00092.30;PF00084.22;PF13519.8 | extracellular | 2.481641293 | 4.155639648 | 8.145860672 | 1.065520644 | 12.1342144 | 21.44258881 |
| A0A835ZMI7 | JEQ12_010974 | A0A835ZMI7_SHEEP Serum amyloid A protein OS=Ovis aries OX=9940 GN=JEQ12_010974 PE=3 SV=1 | BP:response to stimulus;BP:defense response;BP:acute-phase response;BP:response to stress;BP:biological_process;BP:inflammatory response;BP:acute inflammatory responseCC:protein-lipid complex;CC:high-density lipoprotein particle;CC:plasma lipoprotein part | K17310 | ENOG410YSBK | PF00277.20 | ER | 72.13082123 | 0 | 0 | 4.670353413 | 1446.940308 | 391.949585 |
| A0A452E8F8 | HCFC2 | A0A452E8F8_CAPHI Host cell factor C2 OS=Capra hircus OX=9925 GN=HCFC2 PE=4 SV=1 | CC:nuclear body;CC:macromolecular complex;CC:catalytic complex;CC:cytosol;CC:membrane;CC:histone methyltransferase complex;CC:cellular_component;CC:methyltransferase complex;CC:transferase complex;CC:plasma membrane;CC:MLL1 complex;CC:cellular anatomical | K14966 | ENOG410Y5AC | PF13415.8;PF13854.8;PF01344.27;PF13418.8;PF13964.8;PF00041.23 | mitochondrial | 849.4747925 | 764.5675659 | 1172.519653 | 1330.47876 | 629.8916626 | 654.3209839 |
| A0A452E7M1 | KLF11 | A0A452E7M1_CAPHI Kruppel like factor 11 OS=Capra hircus OX=9925 GN=KLF11 PE=4 SV=1 | BP:negative regulation of metabolic process;BP:regulation of primary metabolic process;BP:regulation of metabolic process;BP:negative regulation of cellular biosynthetic process;BP:regulation of cellular biosynthetic process;BP:negative regulation of cell | K09208;K09209;K09206;K09207 | COG5048 | PF00096.28;PF13465.8;PF13894.8 | cytoplasmic | 0 | 0 | 0 | 130.6253052 | 0 | 0 |
| A0A452DKU9 | VCL | A0A452DKU9_CAPHI Vinculin OS=Capra hircus OX=9925 GN=VCL PE=3 SV=1 | BP:axon extension;BP:protein localization;BP:developmental growth;BP:developmental cell growth;BP:regulation of focal adhesion assembly;BP:cellular component organization or biogenesis;BP:cellular macromolecule localization;BP:regulation of endothelial ce | K05691;K05700 | ENOG410XSRU | PF01044.21 | cytoplasmic | 28.60057068 | 18.90829086 | 24.50277519 | 32.63731003 | 22.75959396 | 30.00502205 |
| A0A452FJF9 | TF | A0A452FJF9_CAPHI Serotransferrin OS=Capra hircus OX=9925 GN=TF PE=3 SV=1 | BP:regulation of primary metabolic process;BP:regulation of metabolic process;BP:regulation of transport;BP:regulation of RNA biosynthetic process;BP:iron ion homeostasis;BP:iron ion transport;BP:signal transduction;BP:transition metal ion homeostasis;BP: | K17283;K14736;K06569 | ENOG410XQ36 | PF00405.19;PF12974.9 | extracellular | 0 | 6642.846191 | 6373.050781 | 0 | 5602.231934 | 7003.818848 |
| A0A452FV45 | LUM | A0A452FV45_CAPHI Lumican OS=Capra hircus OX=9925 GN=LUM PE=3 SV=1 | BP:regulation of RNA metabolic process;BP:regulation of biosynthetic process;BP:positive regulation of metabolic process;BP:regulation of transforming growth factor beta1 production;BP:positive regulation of cellular biosynthetic process;BP:regulation of | K08121;K08122;K08123;K08124;K08125 | COG4886 | PF13855.8;PF12799.9;PF01462.20;PF14580.8 | extracellular | 257.0403442 | 483.9082031 | 412.3699951 | 440.2530212 | 197.9762421 | 472.3597107 |
| A0A452E4F5 | RNASE4 | A0A452E4F5_CAPHI Ribonuclease A family member 4 OS=Capra hircus OX=9925 GN=RNASE4 PE=3 SV=1 | CC:extracellular space;CC:cellular_component;CC:extracellular region;CC:cellular anatomical entityMF:hydrolase activity;MF:catalytic activity, acting on RNA;MF:nuclease activity;MF:endonuclease activity;MF:ribonuclease activity;MF:hydrolase activity, acti | K16632;K16631;K01168 | ENOG410Y4FD | PF00074.22 | ER | 23.10603905 | 21.94542694 | 33.94083786 | 13.7546587 | 31.1172905 | 24.90513992 |
| A0A836CVX0 | JEQ12_005411 | A0A836CVX0_SHEEP Apolipoprotein E OS=Ovis aries OX=9940 GN=JEQ12_005411 PE=3 SV=1 | BP:very-low-density lipoprotein particle clearance;BP:regulation of neuron apoptotic process;BP:negative regulation of neuron apoptotic process;BP:small molecule metabolic process;BP:small molecule catabolic process;BP:organic cyclic compound metabolic pr | K04524 | ENOG4111MYC | PF01442.20 | cytoplasmic | 1227.091187 | 755.1044312 | 742.661499 | 865.5828247 | 1203.47583 | 1152.159912 |
| A0A452GB68 | LOC102173987 | A0A452GB68_CAPHI High affinity immunoglobulin gamma Fc receptor I-like OS=Capra hircus OX=9925 GN=LOC102173987 PE=4 SV=1 | CC:cellular_component;CC:integral component of membrane;CC:cellular anatomical entity;CC:intrinsic component of membrane | K12560;K06498 | ENOG410YF57 | PF13895.8;PF13927.8;PF00047.27 | Golgi apparatus | 23.12470436 | 13.81404972 | 15.83441734 | 11.453372 | 3.254223347 | 16.82844925 |
| A0A836CU09 | JEQ12_007506 | A0A836CU09_SHEEP Ig-like domain-containing protein OS=Ovis aries OX=9940 GN=JEQ12_007506 PE=4 SV=1 | ------ | K06553 | ENOG41117U9;ENOG410Y9T8;ENOG410ZN0R | PF07686.19;PF07679.18 | extracellular | 934.0441284 | 949.0002441 | 462.8907776 | 685.8082275 | 722.4632568 | 5760.889648 |
| A0A836CWP3 | JEQ12_004558 | A0A836CWP3_SHEEP BPI1 domain-containing protein OS=Ovis aries OX=9940 GN=JEQ12_004558 PE=4 SV=1 | ------ | ------ | ENOG4111BIC | PF01273.27;PF11944.10 | extracellular | 7148.631348 | 1361.091309 | 399.9700012 | 624.7941284 | 278.9029541 | 1429.401611 |
| A0A836CYQ9 | JEQ12_004009 | A0A836CYQ9_SHEEP Coagulation factor XIII B chain OS=Ovis aries OX=9940 GN=JEQ12_004009 PE=4 SV=1 | ------ | K23817;K03906;K23816;K04004 | ENOG410YE48 | PF00084.22;PF09014.12 | extracellular | 375.9665833 | 490.5286865 | 432.9975281 | 445.2520752 | 413.2776794 | 347.9647217 |
| W5NPK5 | LOC443475 | W5NPK5_SHEEP Complement C3 OS=Ovis aries OX=9940 GN=LOC443475 PE=4 SV=1 | BP:regulation of phosphate metabolic process;BP:regulation of primary metabolic process;BP:regulation of metabolic process;BP:regulation of transport;BP:positive regulation of response to stimulus;BP:regulation of response to stimulus;BP:cell maturation;B | K03990;K03994;K03989 | ENOG410XRED | PF07678.16;PF07677.16 | cytoplasmic | 3726.44751 | 2327.556152 | 1744.159058 | 1814.801514 | 2373.563477 | 3392.665039 |
| A0A835ZX29 | JEQ12_008388 | A0A835ZX29_SHEEP Inter-alpha-trypsin inhibitor heavy chain H3 OS=Ovis aries OX=9940 GN=JEQ12_008388 PE=3 SV=1 | BP:glycosaminoglycan metabolic process;BP:mucopolysaccharide metabolic process;BP:metabolic process;BP:nitrogen compound metabolic process;BP:organic substance metabolic process;BP:hyaluronan metabolic process;BP:biological_process;BP:organonitrogen compo | K24513;K19014;K19015;K24515;K24514 | COG2304 | PF06668.14;PF08487.12;PF00092.30;PF13768.8;PF13519.8 | extracellular | 164.5293274 | 101.0803986 | 1.971909642 | 242.7931366 | 166.794342 | 0 |
| A0A452G0P6 | KRT10 | A0A452G0P6_CAPHI Keratin 10 OS=Capra hircus OX=9925 GN=KRT10 PE=3 SV=1 | CC:cellular_component;CC:supramolecular fiber;CC:polymeric cytoskeletal fiber;CC:supramolecular complex;CC:supramolecular polymer;CC:intermediate filament;CC:cellular anatomical entityMF:structural molecule activity;MF:molecular_function | K07604 | ENOG410YDXS | PF00038.23 | cytoplasmic | 311.238678 | 742.1697388 | 902.4124146 | 770.2853394 | 296.9194031 | 3401.049072 |
| A0A836ACY8 | JEQ12_000385 | A0A836ACY8_SHEEP PID domain-containing protein OS=Ovis aries OX=9940 GN=JEQ12_000385 PE=3 SV=1 | BP:positive regulation of response to stimulus;BP:regulation of response to stimulus;BP:interspecies interaction between organisms;BP:regulation of biological process;BP:cytolysis;BP:response to external biotic stimulus;BP:biological_process;BP:positive r | K03998;K03996;K03995;K20054;K03997 | ENOG410Y2J1;ENOG410XZ1H | PF01823.21;PF00640.25;PF00057.20 | nuclear | 93.46022797 | 44.22737503 | 191.808136 | 49.46747208 | 122.0491562 | 145.1218567 |
| A0A452EHT7 | ITIH4 | A0A452EHT7_CAPHI Inter-alpha-trypsin inhibitor heavy chain 4 OS=Capra hircus OX=9925 GN=ITIH4 PE=3 SV=1 | BP:glycosaminoglycan metabolic process;BP:mucopolysaccharide metabolic process;BP:metabolic process;BP:nitrogen compound metabolic process;BP:organic substance metabolic process;BP:hyaluronan metabolic process;BP:biological_process;BP:organonitrogen compo | K24513;K19014;K19015;K24515;K24514 | COG2304 | PF08487.12;PF00092.30;PF13768.8;PF13519.8;PF06668.14 | extracellular | 9678.773438 | 5690.632813 | 7703.362793 | 5816.315918 | 10982.48242 | 8067.253418 |
| A0A452FHV6 | PLTP | A0A452FHV6_CAPHI Phospholipid transfer protein OS=Capra hircus OX=9925 GN=PLTP PE=3 SV=1 | BP:fat-soluble vitamin metabolic process;BP:regulation of transport;BP:small molecule metabolic process;BP:small molecule biosynthetic process;BP:cilium movement;BP:organic cyclic compound biosynthetic process;BP:cellular component organization or biogene | K05399;K08761 | ENOG410Z88E | PF02886.19;PF01273.27 | ER | 202.5576324 | 126.7938461 | 35.79224777 | 72.32432556 | 20.81347084 | 45.25628662 |
| A0A452E5W4 | KLKB1 | A0A452E5W4_CAPHI Kallikrein B1 OS=Capra hircus OX=9925 GN=KLKB1 PE=4 SV=1 | BP:negative regulation of response to external stimulus;BP:inflammatory response;BP:regulation of response to external stimulus;BP:negative regulation of response to stimulus;BP:blood coagulation;BP:regulation of response to stimulus;BP:regulation of woun | K09628;K01323;K09634;K09633;K01324 | COG5640 | PF00089.28;PF00024.28;PF14295.8;PF13365.8 | extracellular | 180.7230835 | 99.73644257 | 205.0058289 | 149.5918579 | 196.4660187 | 207.2220764 |
| W5PDR5 | C8A | W5PDR5_SHEEP Complement C8 alpha chain OS=Ovis aries OX=9940 GN=C8A PE=3 SV=1 | BP:positive regulation of response to stimulus;BP:regulation of response to stimulus;BP:regulation of biological process;BP:cytolysis;BP:response to external biotic stimulus;BP:interspecies interaction between organisms;BP:positive regulation of immune sy | K03998;K04000;K03995;K03996;K03997 | ENOG410Y5MF | PF01823.21;PF00057.20;PF00090.21 | extracellular | 248.302597 | 414.9537048 | 274.4184265 | 317.4932251 | 193.8972473 | 188.0760193 |
| W5Q9D5 | VTN | W5Q9D5_SHEEP Vitronectin OS=Ovis aries OX=9940 GN=VTN PE=4 SV=1 | BP:regulation of cell adhesion;BP:cell differentiation;BP:cell-matrix adhesion;BP:glial cell differentiation;BP:regulation of biological process;BP:cellular component organization or biogenesis;BP:immune system process;BP:positive regulation of cell adhes | K07763;K06251;K08006;K24286;K01394 | ENOG410XQ5D | PF00045.21;PF01033.19 | extracellular | 411.7986755 | 203.0831146 | 518.4526367 | 72.39974976 | 35.96477127 | 130.4720459 |
| A0A452FXE6 | IGFBP3 | A0A452FXE6_CAPHI Insulin-like growth factor-binding protein 3 OS=Capra hircus OX=9925 GN=IGFBP3 PE=4 SV=1 | BP:regulation of phosphate metabolic process;BP:regulation of primary metabolic process;BP:regulation of metabolic process;BP:positive regulation of response to stimulus;BP:regulation of response to stimulus;BP:negative regulation of macromolecule metabol | K23575;K23576;K23577;K23579;K10138 | ENOG4111MF3 | PF00086.20;PF00219.20 | mitochondrial | 71.39404297 | 68.84345245 | 59.49097443 | 51.5247345 | 52.91701508 | 50.02700806 |
| A0A452DYR7 | CST3 | A0A452DYR7_CAPHI Cystatin C OS=Capra hircus OX=9925 GN=CST3 PE=4 SV=1 | BP:regulation of glycoprotein metabolic process;BP:regulation of catabolic process;BP:negative regulation of catabolic process;BP:negative regulation of metabolic process;BP:regulation of multicellular organismal process;BP:regulation of metabolic process | K13899;K13902;K13903 | ENOG4112CFJ | PF00031.23;PF16845.7 | mitochondrial | 186.3424835 | 208.2896881 | 180.6898804 | 170.2763214 | 173.299469 | 186.2562408 |
| A0A452G6Z7 | KIAA0100 | A0A452G6Z7_CAPHI KIAA0100 OS=Capra hircus OX=9925 GN=KIAA0100 PE=4 SV=1 | ------ | ------ | ENOG410XSYR | PF10351.11;PF10344.11;PF10347.11 | ER | 230.8115234 | 271.8056641 | 1016.879028 | 40.97438431 | 118.5905457 | 538.6534424 |
| A0A836CZY3 | JEQ12_017982 | A0A836CZY3_SHEEP RNAse_Pc domain-containing protein OS=Ovis aries OX=9940 GN=JEQ12_017982 PE=3 SV=1 | BP:negative regulation of cellular amide metabolic process;BP:posttranscriptional regulation of gene expression;BP:regulation of metabolic process;BP:negative regulation of cellular biosynthetic process;BP:regulation of protein metabolic process;BP:cell d | K16632;K16631;K01168 | ENOG410Y4FD | PF00074.22 | ER | 4.023369789 | 4.712497234 | 2.947215557 | 6.755691051 | 4.320073128 | 4.266537666 |
| W5P5I0 | CFI | W5P5I0_SHEEP Complement factor I OS=Ovis aries OX=9940 GN=CFI PE=4 SV=1 | BP:positive regulation of response to stimulus;BP:regulation of response to stimulus;BP:regulation of biological process;BP:positive regulation of immune system process;BP:regulation of immune system process;BP:positive regulation of biological process;BP | K01333;K09640;K09641;K09751;K09638 | COG5640 | PF00089.28;PF00057.20;PF00530.20 | cytoplasmic | 2517.38501 | 2364.436768 | 3056.585449 | 2710.234863 | 2759.412598 | 2560.327881 |
| A0A836CTG3 | JEQ12_008015 | A0A836CTG3_SHEEP Serpin A1 OS=Ovis aries OX=9940 GN=JEQ12_008015 PE=3 SV=1 | BP:hemostasis;BP:blood coagulation;BP:glucocorticoid metabolic process;BP:organic cyclic compound metabolic process;BP:organic substance metabolic process;BP:biological regulation;BP:regulation of biological quality;BP:multicellular organismal process;BP: | K03984;K04525 | COG4826 | PF00079.22 | ER | 33922.49609 | 43664.08203 | 32700.17578 | 41990.36719 | 40137.17578 | 38645.92578 |
| A0A452EEM4 | PGLYRP1 | A0A452EEM4_CAPHI Peptidoglycan-recognition protein OS=Capra hircus OX=9925 GN=PGLYRP1 PE=3 SV=1 | BP:detection of biotic stimulus;BP:regulation of metabolic process;BP:negative regulation of response to stimulus;BP:regulation of response to stimulus;BP:negative regulation of defense response;BP:regulation of defense response;BP:negative regulation of | K01446 | ENOG4111PQD | PF01510.27 | extracellular | 129.7678375 | 50.28770447 | 52.31240082 | 128.8640137 | 141.4698029 | 122.6978073 |
| A0A452EW91 | CILP2 | A0A452EW91_CAPHI Cartilage intermediate layer protein 2 OS=Capra hircus OX=9925 GN=CILP2 PE=4 SV=1 | CC:cellular_component;CC:extracellular region;CC:cellular anatomical entity | K00907;K22654;K24436;K16353 | ENOG410XXZR | PF13330.8;PF13927.8;PF13620.8;PF00090.21;PF13895.8;PF07679.18 | extracellular | 17.18451691 | 43.17960739 | 23.64767265 | 21.44539833 | 10.21117878 | 16.07321548 |
| A0A836CVH6 | JEQ12_010475 | A0A836CVH6_SHEEP Complement C4 gamma chain OS=Ovis aries OX=9940 GN=JEQ12_010475 PE=4 SV=1 | BP:inflammatory response;BP:positive regulation of response to stimulus;BP:regulation of response to stimulus;BP:regulation of biological process;BP:humoral immune response;BP:response to external biotic stimulus;BP:interspecies interaction between organi | K03990;K03989 | ENOG410XRED | PF07678.16;PF00207.24;PF07677.16;PF07703.16;PF01759.23;PF01835.21;PF17791.3;PF17789.3;PF01821.20 | ER | 11913.30859 | 11552.50586 | 7833.972656 | 9939.640625 | 15047.35156 | 9489.777344 |
| I1WXR3 | SERPINA1 | I1WXR3_SHEEP Alpha-1-antitrypsin transcript variant 1 OS=Ovis aries OX=9940 GN=SERPINA1 PE=2 SV=1 | BP:negative regulation of metabolic process;BP:regulation of metabolic process;BP:negative regulation of cellular metabolic process;BP:regulation of cellular metabolic process;BP:negative regulation of nitrogen compound metabolic process;BP:negative regul | K03984;K04525 | COG4826 | PF00079.22 | ER | 20.54847908 | 23.51205826 | 44.28222656 | 53.48075867 | 23.93252945 | 15.19974899 |
| A0A836CUA1 | JEQ12_006895 | A0A836CUA1_SHEEP Complement component C7 OS=Ovis aries OX=9940 GN=JEQ12_006895 PE=3 SV=1 | BP:positive regulation of response to stimulus;BP:regulation of response to stimulus;BP:regulation of biological process;BP:cytolysis;BP:response to external biotic stimulus;BP:interspecies interaction between organisms;BP:positive regulation of immune sy | K03998;K04000;K03995;K03996;K03997 | ENOG410YJ70 | PF01823.21;PF18434.3;PF00084.22;PF00057.20 | extracellular | 826.0314941 | 920.904541 | 790.8457642 | 722.0651245 | 702.4916992 | 767.6085205 |
| A0A452GAG0 | AMBP | A0A452GAG0_CAPHI Protein AMBP OS=Capra hircus OX=9925 GN=AMBP PE=3 SV=1 | BP:nitrogen compound metabolic process;BP:macromolecule metabolic process;BP:cellular protein metabolic process;BP:cellular macromolecule metabolic process;BP:organic substance metabolic process;BP:cellular process;BP:protein-chromophore linkage;BP:macrom | K23620;K23621;K23421;K03909;K23087 | ENOG410XQNP | PF00014.25;PF00061.25 | lysosomal | 3141.876709 | 2756.360596 | 3117.810791 | 2399.292969 | 2760.958252 | 2269.489502 |
| A0A8C2R1B8 |  | A0A8C2R1B8_CAPHI Serpin family A member 6 OS=Capra hircus OX=9925 PE=3 SV=1 | BP:negative regulation of metabolic process;BP:regulation of primary metabolic process;BP:regulation of metabolic process;BP:negative regulation of cellular metabolic process;BP:regulation of cellular metabolic process;BP:metabolic process;BP:glucocortico | K03984;K04525 | COG4826 | PF00079.22 | extracellular | 0 | 0 | 0 | 4.279446602 | 0 | 3.632297754 |
| A0A452E8A8 |  | A0A452E8A8_CAPHI Ig-like domain-containing protein OS=Capra hircus OX=9925 PE=4 SV=1 | ------ | K06553 | ENOG410Y9T8;ENOG410YNU3 | PF07686.19;PF07679.18 | extracellular | 1636.149902 | 1433.97998 | 473.8243408 | 793.4597168 | 699.8200684 | 1774.384521 |
| A0A452DX18 | C3 | A0A452DX18_CAPHI Complement C3 OS=Capra hircus OX=9925 GN=C3 PE=4 SV=1 | BP:regulation of phosphate metabolic process;BP:regulation of primary metabolic process;BP:regulation of metabolic process;BP:regulation of transport;BP:positive regulation of response to stimulus;BP:regulation of response to stimulus;BP:cell maturation;B | K03990;K03994;K03989 | ENOG410XRED | PF07678.16;PF17790.3;PF07677.16;PF07703.16;PF17789.3;PF01759.23;PF00207.24;PF01835.21;PF01821.20;PF17791.3 | ER | 1312.002319 | 1307.066528 | 1605.683838 | 1110.032593 | 630.1688232 | 852.961731 |
| P85295 | ALB | ALBU_CAPHI Albumin (Fragment) OS=Capra hircus OX=9925 GN=ALB PE=1 SV=2 | CC:extracellular space;CC:cellular_component;CC:cellular anatomical entityMF:anion binding;MF:cation binding;MF:organic cyclic compound binding;MF:heterocyclic compound binding;MF:lipid binding;MF:ion binding;MF:macrolide binding;MF:molecular_function;MF: | K16141 | ENOG410Z40H | ------ | cytoplasmic | 200.2854004 | 0 | 68.95519257 | 0 | 113.413414 | 133.0063477 |
| A0A836CTB6 | JEQ12_013017 | A0A836CTB6_SHEEP C-type lectin domain-containing protein OS=Ovis aries OX=9940 GN=JEQ12_013017 PE=4 SV=1 | BP:regulation of immune system process;BP:regulation of transport;BP:positive regulation of response to stimulus;BP:regulation of response to stimulus;BP:positive regulation of immune response;BP:symbiosis, encompassing mutualism through parasitism;BP:def | K03991;K10068 | ENOG4110NRD | PF00059.23;PF01391.20 | extracellular | 0 | 21.94350243 | 41.96352005 | 48.60848999 | 122.0027466 | 77.88394165 |
| A0A835ZMK0 | JEQ12_020330 | A0A835ZMK0_SHEEP Coagulation factor IX OS=Ovis aries OX=9940 GN=JEQ12_020330 PE=4 SV=1 | BP:hemostasis;BP:blood coagulation;BP:nitrogen compound metabolic process;BP:zymogen activation;BP:organic substance metabolic process;BP:biological regulation;BP:proteolysis;BP:regulation of biological quality;BP:multicellular organismal process;BP:regul | K01321;K01314;K01344;K12885;K01320 | COG5640 | PF00089.28;PF00076.24;PF14670.8;PF00008.29 | cytoplasmic | 446.5879822 | 379.8912659 | 363.8557739 | 394.4535522 | 460.0212097 | 318.5023804 |
| W5PD71 | LOC101115495 | W5PD71_SHEEP Pentaxin OS=Ovis aries OX=9940 GN=LOC101115495 PE=3 SV=1 | BP:regulation of metabolic process;BP:regulation of superoxide anion generation;BP:positive regulation of response to stimulus;BP:regulation of response to stimulus;BP:circulatory system process;BP:vascular process in circulatory system;BP:positive regula | K16143;K23267 | ENOG410YIJN | PF00354.19;PF13385.8 | lysosomal | 3323.932129 | 2553.669678 | 3393.861816 | 4949.53418 | 4237.138184 | 3425.246094 |
| A0A452G1N9 | SFTPB | A0A452G1N9_CAPHI Surfactant protein B OS=Capra hircus OX=9925 GN=SFTPB PE=4 SV=1 | BP:nitrogen compound metabolic process;BP:membrane lipid metabolic process;BP:organic substance metabolic process;BP:sphingolipid metabolic process;BP:multicellular organismal process;BP:lipid metabolic process;BP:respiratory gaseous exchange;BP:biologica | K12382 | ENOG410XSI5 | PF03489.19;PF02199.17;PF05184.17 | extracellular | 178.2563477 | 11.06967735 | 16.97952461 | 10.69830799 | 30.15173912 | 12.57551193 |
| A0A836D082 | JEQ12_004559 | A0A836D082_SHEEP BPI1 domain-containing protein OS=Ovis aries OX=9940 GN=JEQ12_004559 PE=4 SV=1 | BP:homeostatic process;BP:biological regulation;BP:biological_process;BP:regulation of liquid surface tension;BP:regulation of biological qualityMF:molecular_function;MF:lipid binding;MF:binding | ------ | ENOG4111BIC | PF01273.27 | extracellular | 3.768491268 | 56.33722687 | 0 | 29.30741882 | 0 | 91.68357849 |
| A0A836D9E9 | JEQ12_010128 | A0A836D9E9_SHEEP C1q domain-containing protein OS=Ovis aries OX=9940 GN=JEQ12_010128 PE=4 SV=1 | BP:synapse organization;BP:cell junction organization;BP:cell junction disassembly;BP:synapse disassembly;BP:regulation of biological process;BP:cellular component organization or biogenesis;BP:regulation of myeloid leukocyte differentiation;BP:negative r | K03987;K03986;K24213;K24212;K03988 | ENOG4111MQB | PF00386.23;PF01391.20 | extracellular | 1742.49939 | 1222.778198 | 1594.000122 | 1594.749268 | 1241.326416 | 1467.313232 |
| A0A836CVB7 | JEQ12_007520 | A0A836CVB7_SHEEP Ig-like domain-containing protein OS=Ovis aries OX=9940 GN=JEQ12_007520 PE=4 SV=1 | ------ | K06553 | ------ | PF07686.19;PF13927.8;PF07679.18 | extracellular | 713.9609985 | 1136.095825 | 1369.365479 | 556.3499146 | 475.8899536 | 2590.178955 |
| A0A452EX71 | CPB2 | A0A452EX71_CAPHI Carboxypeptidase B2 OS=Capra hircus OX=9925 GN=CPB2 PE=3 SV=1 | BP:negative regulation of response to external stimulus;BP:metabolic process;BP:regulation of response to external stimulus;BP:negative regulation of response to stimulus;BP:blood coagulation;BP:regulation of response to stimulus;BP:regulation of wound he | K01291;K08780;K08782;K01300;K23009 | COG2866 | PF00246.26;PF02244.18 | ER | 166.3027649 | 293.9972229 | 248.5724487 | 178.5421906 | 212.6272583 | 233.7812958 |
| A0A452DQJ9 | APOM | A0A452DQJ9_CAPHI Apolipoprotein M OS=Capra hircus OX=9925 GN=APOM PE=3 SV=1 | BP:cholesterol transport;BP:plasma lipoprotein particle assembly;BP:high-density lipoprotein particle remodeling;BP:negative regulation of plasma lipoprotein particle oxidation;BP:sterol transport;BP:lipid transport;BP:macromolecular complex subunit organ | ------ | ENOG4111Z0U | PF11032.10 | ER | 63.16686249 | 168.26297 | 172.9020996 | 171.4008942 | 76.98028564 | 159.1947174 |
| A0A836D2H0 | JEQ12_002340 | A0A836D2H0_SHEEP Coagulation factor X OS=Ovis aries OX=9940 GN=JEQ12_002340 PE=4 SV=1 | BP:hemostasis;BP:positive regulation of cell communication;BP:blood coagulation;BP:regulation of response to stimulus;BP:positive regulation of signaling;BP:regulation of biological process;BP:regulation of signaling;BP:regulation of cell communication;BP | K01314;K01321;K01344;K24467;K01320 | COG5640 | PF00089.28;PF00594.22;PF14670.8;PF00008.29 | extracellular | 401.1361084 | 435.012207 | 316.7023926 | 396.7189941 | 348.8853455 | 371.9696655 |
| A0A452E819 | KNG1 | A0A452E819_CAPHI Kininogen 1 OS=Capra hircus OX=9925 GN=KNG1 PE=4 SV=1 | BP:hemostasis;BP:regulation of primary metabolic process;BP:regulation of metabolic process;BP:negative regulation of response to stimulus;BP:blood coagulation;BP:regulation of response to stimulus;BP:negative regulation of cell adhesion;BP:circulatory sy | K03898;K13903 | ENOG4111ZQ8 | PF00031.23;PF00666.19 | extracellular | 3615.345703 | 4620.466309 | 3889.515137 | 3816.82251 | 4495.5 | 3782.391357 |
| W5NXM1 |  | W5NXM1_SHEEP A2M domain-containing protein OS=Ovis aries OX=9940 PE=4 SV=1 | BP:regulation of phosphate metabolic process;BP:regulation of primary metabolic process;BP:regulation of metabolic process;BP:regulation of transport;BP:positive regulation of response to stimulus;BP:regulation of response to stimulus;BP:cell maturation;B | K03990;K06530;K03910;K03989 | ENOG410XRED | PF00207.24 | cytoplasmic | 2055.034424 | 1756.035278 | 2837.533203 | 1990.495117 | 1163.744873 | 1377.039795 |
| A0A8C2SCG5 | BAC7 | A0A8C2SCG5_CAPHI Antibacterial protein PR-39-like OS=Capra hircus OX=9925 GN=BAC7.5 PE=3 SV=1 | BP:antimicrobial humoral response;BP:defense response to bacterium;BP:defense response to Gram-negative bacterium;BP:antimicrobial humoral immune response mediated by antimicrobial peptide;BP:cytolysis;BP:response to external biotic stimulus;BP:response t | K13916 | ENOG4111DSI;ENOG41119S0 | PF00666.19 | mitochondrial | 209.9976044 | 49.48556137 | 33.70651627 | 53.30778122 | 102.7455521 | 69.87039185 |
| A0A452EWI1 | APOB | A0A452EWI1_CAPHI Apolipoprotein B OS=Capra hircus OX=9925 GN=APOB PE=4 SV=1 | MF:lipid transporter activity;MF:transporter activity;MF:molecular_function | K14462 | ENOG411104F | PF01347.24;PF09172.13;PF06448.13;PF12491.10 | ER | 51.63901138 | 30.92551422 | 0 | 26.90599823 | 55.34194946 | 81.40631866 |
| A0A836AQC4 | JEQ12_001474 | A0A836AQC4_SHEEP Carboxypeptidase N subunit 2 OS=Ovis aries OX=9940 GN=JEQ12_001474 PE=4 SV=1 | CC:membrane;CC:extracellular space;CC:integral component of membrane;CC:external encapsulating structure;CC:cellular_component;CC:intrinsic component of membrane;CC:plasma membrane;CC:extracellular matrix;CC:cellular anatomical entityMF:hydrolase activity | K13023 | COG4886 | PF13855.8;PF13306.8;PF12799.9;PF14580.8;PF01462.20 | extracellular | 672.9272461 | 631.15802 | 643.2619019 | 539.444519 | 656.5819092 | 479.5545349 |
| A0A452FPJ0 | ITPRID1 | A0A452FPJ0_CAPHI ITPR interacting domain containing 1 OS=Capra hircus OX=9925 GN=ITPRID1 PE=4 SV=1 | MF:receptor binding;MF:molecular_function;MF:binding;MF:protein binding | ------ | ENOG410YK4C | PF14722.8;PF14723.8 | cytoplasmic | 699.8189697 | 649.4841309 | 1023.984253 | 1628.287109 | 799.8717041 | 1290.168457 |
| A0A452G4S7 | C8A | A0A452G4S7_CAPHI Complement C8 alpha chain OS=Capra hircus OX=9925 GN=C8A PE=3 SV=1 | BP:positive regulation of response to stimulus;BP:regulation of response to stimulus;BP:regulation of biological process;BP:cytolysis;BP:response to external biotic stimulus;BP:interspecies interaction between organisms;BP:positive regulation of immune sy | K03998;K04000;K03995;K03996;K03997 | ENOG410Y5MF | PF01823.21;PF00057.20 | extracellular | 495.5071106 | 899.699707 | 736.2716064 | 737.777771 | 537.1173706 | 538.7020874 |
| A0A452DNE9 | LOC102189464 | A0A452DNE9_CAPHI Apolipoprotein F OS=Capra hircus OX=9925 GN=LOC102189464 PE=4 SV=1 | ------ | ------ | ENOG41119MQ;ENOG4112458 | PF15148.8 | extracellular | 4733.907227 | 4880.913574 | 3763.243164 | 5353.867676 | 3977.274414 | 3276.880615 |
| A0A452EDL5 | PON1 | A0A452EDL5_CAPHI Paraoxonase OS=Capra hircus OX=9925 GN=PON1 PE=3 SV=1 | BP:regulation of transport;BP:small molecule metabolic process;BP:small molecule catabolic process;BP:organic cyclic compound metabolic process;BP:organophosphate catabolic process;BP:positive regulation of molecular function;BP:positive regulation of bio | K01045 | ENOG4111QK7 | PF01731.22;PF08450.14 | mitochondrial | 4184.854492 | 6154.716309 | 3519.813965 | 6467.609863 | 6193.276855 | 5754.962402 |
| A0A452DPJ3 |  | A0A452DPJ3_CAPHI Ig-like domain-containing protein OS=Capra hircus OX=9925 PE=4 SV=1 | ------ | K06553 | ENOG410ZN0R | PF07686.19;PF13927.8 | extracellular | 1015.081055 | 930.0616455 | 1116.49292 | 1713.358398 | 912.9835815 | 1172.280396 |
| A0A835ZPL0 | JEQ12_008022 | A0A835ZPL0_SHEEP SERPIN domain-containing protein OS=Ovis aries OX=9940 GN=JEQ12_008022 PE=3 SV=1 | CC:cellular_component;CC:extracellular space;CC:cellular anatomical entity | K04525 | COG4826 | PF00079.22 | extracellular | 49326.5625 | 50786.45313 | 42173.16797 | 48712.18359 | 51716.76563 | 49036.63672 |
| A0A452FIS2 | CD44 | A0A452FIS2_CAPHI CD44 antigen OS=Capra hircus OX=9925 GN=CD44 PE=4 SV=1 | BP:cellular process;BP:cell adhesion;BP:biological_process;BP:biological adhesionCC:cellular_component;CC:cell projection;CC:integral component of membrane;CC:actin-based cell projection;CC:cellular anatomical entity;CC:microvillus;CC:plasma membrane boun | K19012;K19018;K06256 | ENOG4111S6T | PF00193.19 | plasma membrane | 240.7784729 | 138.0110321 | 76.56664276 | 129.5020142 | 69.41236115 | 118.7511063 |
| A0A452G6B2 | HGFAC | A0A452G6B2_CAPHI HGF activator OS=Capra hircus OX=9925 GN=HGFAC PE=4 SV=1 | CC:cellular_component;CC:extracellular region;CC:cellular anatomical entityMF:serine-type endopeptidase activity;MF:endopeptidase activity;MF:hydrolase activity;MF:serine hydrolase activity;MF:catalytic activity, acting on a protein;MF:catalytic activity; | K01328;K01343;K08648;K09631;K01348 | COG5640 | PF00089.28;PF00051.20;PF00040.21;PF00008.29;PF00039.20 | extracellular | 66.21595001 | 169.0576324 | 216.3764191 | 95.9998703 | 130.7050323 | 134.6312256 |
| A0A452G0Q8 | LOC102190762 | A0A452G0Q8_CAPHI C1q domain-containing protein OS=Capra hircus OX=9925 GN=LOC102190762 PE=4 SV=1 | CC:cellular_component;CC:extracellular region;CC:macromolecular complex;CC:collagen trimer;CC:cellular anatomical entity | K19479;K24218 | ENOG411198C | PF00386.23;PF01391.20 | extracellular | 409.6259766 | 682.0753174 | 503.7188416 | 455.1750793 | 483.9482117 | 488.472229 |
| A0A452G8K0 | LOC102191308 | A0A452G8K0_CAPHI Protein HP-20 homolog OS=Capra hircus OX=9925 GN=LOC102191308 PE=4 SV=1 | CC:cellular_component;CC:extracellular region;CC:macromolecular complex;CC:collagen trimer;CC:cellular anatomical entity | K23455;K24213 | ENOG41114DJ | PF00386.23;PF01391.20 | extracellular | 448.4430542 | 592.7370605 | 429.5987244 | 428.7208862 | 435.4564209 | 342.5440674 |
| A0A835ZPM7 | JEQ12_008125 | A0A835ZPM7_SHEEP Immunoglobulin heavy constant mu OS=Ovis aries OX=9940 GN=JEQ12_008125 PE=4 SV=1 | ------ | K06751;K06752;K06554 | ENOG410YNB1 | PF07654.17;PF08205.14;PF00047.27;PF13927.8 | cytoplasmic | 73545.92969 | 66577.83594 | 106337.8828 | 80056.64844 | 81808.80469 | 104369.375 |
| W5QHZ5 |  | W5QHZ5_SHEEP Immunoglobulin kappa light chain-like OS=Ovis aries OX=9940 PE=4 SV=1 | ------ | K06554;K06551 | ENOG41115Q6 | PF07654.17;PF07686.19;PF13927.8;PF00047.27;PF07679.18;PF13895.8;PF08205.14 | plasma membrane | 18390.52344 | 8536.357422 | 9987.543945 | 11362.57813 | 13931.66699 | 17942.26367 |
| A0A6P7ET07 | JEQ12_005413 | A0A6P7ET07_SHEEP Apolipoprotein C-II OS=Ovis aries OX=9940 GN=JEQ12_005413 PE=3 SV=1 | BP:regulation of phosphate metabolic process;BP:regulation of metabolic process;BP:regulation of primary metabolic process;BP:positive regulation of phospholipid metabolic process;BP:positive regulation of lipid metabolic process;BP:positive regulation of | K22287 | ENOG4111AXD | PF05355.13 | ER | 264.3780212 | 221.8531647 | 177.4724121 | 289.1337585 | 272.047699 | 243.5986481 |
| A0A452E480 | LDHB | A0A452E480_CAPHI L-lactate dehydrogenase OS=Capra hircus OX=9925 GN=LDHB PE=3 SV=1 | BP:primary metabolic process;BP:metabolic process;BP:organic substance metabolic process;BP:organic acid metabolic process;BP:cellular metabolic process;BP:cellular process;BP:carboxylic acid metabolic process;BP:biological_process;BP:carbohydrate metabol | K00016 | COG0039 | PF00056.25;PF02866.20 | cytoplasmic | 101.977211 | 132.9384155 | 164.8293457 | 114.0512619 | 108.1432953 | 111.9317474 |
| A0A452FTX9 | LOC108636345 | A0A452FTX9_CAPHI Ig-like domain-containing protein OS=Capra hircus OX=9925 GN=LOC108636345 PE=4 SV=1 | CC:cellular_component;CC:integral component of membrane;CC:cellular anatomical entity;CC:intrinsic component of membrane | K20413;K20414 | ENOG410YSF7 | PF07686.19 | Golgi apparatus | 5.146457672 | 4.079332829 | 4.053558826 | 9.502317429 | 32.12520599 | 8.94907856 |
| A0A452FKE5 | LOC102189753 | A0A452FKE5_CAPHI Complement C4 gamma chain OS=Capra hircus OX=9925 GN=LOC102189753 PE=4 SV=1 | BP:inflammatory response;BP:positive regulation of response to stimulus;BP:regulation of response to stimulus;BP:regulation of biological process;BP:humoral immune response;BP:response to external biotic stimulus;BP:interspecies interaction between organi | K03990;K03989 | ENOG410XRED | PF07678.16;PF00207.24;PF07677.16;PF01759.23;PF07703.16;PF01835.21;PF17791.3;PF17789.3;PF01821.20 | ER | 3843.362305 | 3438.100098 | 1250.197144 | 3179.511719 | 4310.630859 | 2469.486328 |
| A0A836D5W7 | JEQ12_015044 | A0A836D5W7_SHEEP von Willebrand factor OS=Ovis aries OX=9940 GN=JEQ12_015044 PE=4 SV=1 | BP:hemostasis;BP:blood coagulation;BP:biological regulation;BP:biological adhesion;BP:cell-substrate adhesion;BP:multicellular organismal process;BP:regulation of body fluid levels;BP:cellular process;BP:cell activation;BP:coagulation;BP:biological_proces | K21125;K25030;K03900 | ENOG41100RZ | PF00094.27;PF00092.30;PF08742.13;PF16164.7;PF01826.19;PF00093.20 | plasma membrane | 96.14627075 | 74.31542969 | 48.07189178 | 86.30540466 | 51.0865097 | 56.68701553 |
| A0A835ZW29 | JEQ12_005133 | A0A835ZW29_SHEEP Haptoglobin OS=Ovis aries OX=9940 GN=JEQ12_005133 PE=4 SV=1 | BP:inflammatory response;BP:nitrogen compound metabolic process;BP:signal transduction;BP:cell surface receptor signaling pathway;BP:positive regulation of cell death;BP:regulation of cell death;BP:regulation of biological process;BP:immune system process | K01331;K01330;K16142;K03993;K03992 | COG5640 | PF00089.28 | extracellular | 45.7555809 | 17.16065598 | 12.95675755 | 20.8348999 | 15563.01855 | 506.0888367 |
| A0A6P3ECT1 | JEQ12_005949 | A0A6P3ECT1_SHEEP Uncharacterized protein OS=Ovis aries OX=9940 GN=JEQ12_005949 PE=3 SV=1 | BP:negative regulation of plasma lipoprotein particle oxidation;BP:regulation of plasma lipoprotein particle oxidation;BP:regulation of metabolic process;BP:regulation of transport;BP:small molecule metabolic process;BP:positive regulation of cholesterol | K08760 | ENOG41119VI | PF01442.20;PF07464.13;PF04286.14;PF07902.13 | extracellular | 3256.703125 | 9714.692383 | 6754.206543 | 6748.589355 | 5354.23584 | 7593.213379 |
| A0A836D582 | JEQ12_014354 | A0A836D582_SHEEP Keratin, type II cytoskeletal 1 OS=Ovis aries OX=9940 GN=JEQ12_014354 PE=3 SV=1 | CC:cellular_component;CC:supramolecular fiber;CC:polymeric cytoskeletal fiber;CC:keratin filament;CC:supramolecular complex;CC:supramolecular polymer;CC:intermediate filament;CC:cellular anatomical entity | K07605 | ENOG410YY6B;ENOG410XWGZ;ENOG410YNSE | PF00038.23;PF16208.7;PF07926.14 | cytoplasmic | 462.9654846 | 907.8076782 | 562.8771973 | 646.5847778 | 314.2290649 | 2432.034668 |
| A0A452EN59 | LOC102179590 | A0A452EN59_CAPHI C-C motif chemokine OS=Capra hircus OX=9925 GN=LOC102179590 PE=3 SV=1 | BP:cytokine-mediated signaling pathway;BP:positive regulation of response to stimulus;BP:regulation of response to stimulus;BP:signal transduction;BP:cell surface receptor signaling pathway;BP:cell chemotaxis;BP:cellular response to stimulus;BP:taxis;BP:r | K05408;K12499;K12964 | ENOG410YXJU | PF00048.22 | extracellular | 20.31988335 | 17.57653046 | 26.7485466 | 14.08754539 | 28.56189728 | 5.182430267 |
| A0A835ZWH6 | JEQ12_002713 | A0A835ZWH6_SHEEP Amine oxidase OS=Ovis aries OX=9940 GN=JEQ12_002713 PE=3 SV=1 | BP:nitrogen compound metabolic process;BP:regulation of oxidoreductase activity;BP:negative regulation of catalytic activity;BP:organic substance metabolic process;BP:biological regulation;BP:negative regulation of primary amine oxidase activity;BP:regula | K00276 | COG3733 | PF01179.22;PF02728.18;PF02727.18;PF09248.12 | cytoplasmic | 2524.400146 | 3078.620605 | 3213.888184 | 3573.145508 | 2423.082031 | 3398.400391 |
| A0A452EI54 |  | A0A452EI54_CAPHI Ig-like domain-containing protein OS=Capra hircus OX=9925 PE=4 SV=1 | ------ | K06553 | ENOG41115Q6;ENOG410ZN1U;ENOG410YS8R;ENOG410YQB2 | PF07686.19 | plasma membrane | 1093.806641 | 1439.710327 | 603.4076538 | 628.8826294 | 880.5337524 | 729.874939 |
| W5Q7I2 |  | W5Q7I2_SHEEP Ig-like domain-containing protein OS=Ovis aries OX=9940 PE=4 SV=1 | CC:cellular_component;CC:integral component of membrane;CC:cellular anatomical entity;CC:intrinsic component of membrane | K06751;K06554 | ENOG410YNB1 | PF07654.17;PF08205.14;PF13927.8 | cytoplasmic | 1484.663208 | 1221.64563 | 3134.364746 | 4193.810547 | 473.219635 | 1512.144897 |
| A0A452G077 | CCN2 | A0A452G077_CAPHI Cellular communication network factor 2 OS=Capra hircus OX=9925 GN=CCN2 PE=3 SV=1 | BP:regulation of metabolic process;BP:anatomical structure homeostasis;BP:positive regulation of response to stimulus;BP:regulation of response to stimulus;BP:cell-matrix adhesion;BP:cartilage condensation;BP:ossification;BP:signal transduction;BP:cell su | K06829;K23090;K06827;K23571;K22471 | ENOG4111F77 | PF19035.2;PF00007.24;PF00093.20;PF00219.20 | extracellular | 30.49913788 | 13.0915308 | 42.23157501 | 52.30340958 | 11.44798946 | 46.5775032 |
| A0A6P3E1Q0 | JEQ12_009821 | A0A6P3E1Q0_SHEEP Fibronectin OS=Ovis aries OX=9940 GN=JEQ12_009821 PE=4 SV=1 | BP:regulation of cell morphogenesis;BP:regulation of anatomical structure morphogenesis;BP:regulation of biological process;BP:biological regulation;BP:regulation of biological quality;BP:regulation of developmental process;BP:cellular process;BP:defense | K06252;K05717;K08132 | ENOG410Y2NH | PF00041.23;PF00039.20;PF16656.7;PF00040.21 | extracellular | 1608.969849 | 1932.706177 | 1938.441284 | 1620.514282 | 1506.452271 | 1334.212524 |
| A0A452E8D3 |  | A0A452E8D3_CAPHI Ig-like domain-containing protein OS=Capra hircus OX=9925 PE=4 SV=1 | ------ | K06553 | ENOG41117U9;ENOG410ZN0R | PF07686.19;PF13927.8 | extracellular | 17165.56836 | 9696.75 | 13159.63965 | 25536.86328 | 14226.96875 | 11243.13086 |
| A0A836CZR9 | JEQ12_005802 | A0A836CZR9_SHEEP Alpha-1B-glycoprotein OS=Ovis aries OX=9940 GN=JEQ12_005802 PE=4 SV=1 | CC:cellular_component;CC:extracellular region;CC:cellular anatomical entity | K14377 | ENOG4111984 | PF13895.8;PF13927.8;PF00047.27 | extracellular | 19730.03125 | 21724.44336 | 23548.80469 | 17843.10742 | 19410.27734 | 20937.36719 |
| A0A835ZR75 | JEQ12_007052 | A0A835ZR75_SHEEP Fibrinogen C-terminal domain-containing protein OS=Ovis aries OX=9940 GN=JEQ12_007052 PE=4 SV=1 | BP:protein polymerization;BP:cellular component assembly;BP:macromolecular complex subunit organization;BP:cellular process;BP:cell activation;BP:cellular component organization;BP:macromolecular complex assembly;BP:biological_process;BP:cellular macromol | K03905;K03904;K03903 | ENOG410ZYS4 | PF00147.20;PF08702.12 | cytoplasmic | 35.06989288 | 46.33681488 | 74.65081787 | 63.26461029 | 58.57187653 | 68.15127563 |
| A0A452DSP2 | CPN1 | A0A452DSP2_CAPHI Carboxypeptidase N subunit 1 OS=Capra hircus OX=9925 GN=CPN1 PE=3 SV=1 | BP:cellular nitrogen compound metabolic process;BP:nitrogen compound metabolic process;BP:organic substance metabolic process;BP:proteolysis;BP:cellular process;BP:biological_process;BP:metabolic process;BP:protein processing;BP:primary metabolic process; | K07752;K01292;K08638;K01294;K13022 | ENOG410XX0H | PF00246.26;PF13620.8;PF13715.8 | ER | 71.06639099 | 63.59771347 | 86.84618378 | 48.51966476 | 60.46389008 | 63.21781921 |
| A0A452EH87 |  | A0A452EH87_CAPHI Ig-like domain-containing protein OS=Capra hircus OX=9925 PE=4 SV=1 | ------ | ------ | ENOG410YNFT;ENOG41114KX | PF07686.19;PF13927.8 | cytoplasmic | 24.04226685 | 48.64477539 | 60.29033661 | 30.58493042 | 29.31184959 | 71.14752197 |
| A0A835ZVQ5 | JEQ12_004314 | A0A835ZVQ5_SHEEP SREBP regulating gene protein OS=Ovis aries OX=9940 GN=JEQ12_004314 PE=3 SV=1 | BP:positive regulation of response to stimulus;BP:regulation of response to stimulus;BP:positive regulation of signaling;BP:regulation of signaling;BP:positive regulation of cell communication;BP:regulation of cell communication;BP:regulation of biologica | ------ | ENOG4110XXW | PF10218.11 | cytoplasmic | 108.442482 | 9.032578468 | 20.42023468 | 444.6748352 | 7.519366264 | 5.068025112 |
| A0A452EW11 | C7 | A0A452EW11_CAPHI Complement C7 OS=Capra hircus OX=9925 GN=C7 PE=3 SV=1 | BP:positive regulation of response to stimulus;BP:regulation of response to stimulus;BP:regulation of biological process;BP:cytolysis;BP:response to external biotic stimulus;BP:interspecies interaction between organisms;BP:positive regulation of immune sy | K03998;K04000;K03995;K03996;K03997 | ENOG410YJ70 | PF01823.21;PF18434.3;PF00084.22;PF00057.20 | extracellular | 1198.259521 | 1336.353882 | 1173.934448 | 1113.208862 | 1121.023071 | 1120.488037 |
| Q7M323 | PLG | PLMN_CAPHI Plasminogen (Fragment) OS=Capra hircus OX=9925 GN=PLG PE=1 SV=1 | BP:negative regulation of response to external stimulus;BP:regulation of response to external stimulus;BP:negative regulation of response to stimulus;BP:blood coagulation;BP:regulation of response to stimulus;BP:tissue remodeling;BP:regulation of wound he | K01315;K05460;K01313;K05123;K23441 | COG5640 | PF00051.20 | cytoplasmic | 3.192322254 | 3.833625317 | 4.260319233 | 2.439355135 | 4.160329819 | 6.540345669 |
| A0A075W0S2 | BPIFA1 | A0A075W0S2_SHEEP BPI fold-containing family A member 1 OS=Ovis aries OX=9940 GN=BPIFA1 PE=2 SV=1 | BP:antimicrobial humoral response;BP:antibacterial humoral response;BP:regulation of transport;BP:homeostatic process;BP:defense response to bacterium;BP:regulation of liquid surface tension;BP:regulation of ion transmembrane transport;BP:immune response | ------ | ENOG41114MD | PF01273.27 | extracellular | 9.220627785 | 12.66334248 | 48.65524292 | 43.58595276 | 40.25283432 | 17.23285484 |
| A0A835ZJB9 | JEQ12_020579 | A0A835ZJB9_SHEEP SERPIN domain-containing protein OS=Ovis aries OX=9940 GN=JEQ12_020579 PE=3 SV=1 | BP:negative regulation of metabolic process;BP:regulation of primary metabolic process;BP:regulation of metabolic process;BP:negative regulation of cellular metabolic process;BP:regulation of cellular metabolic process;BP:regulation of biological process; | K04525;K20734 | COG4826 | PF00079.22 | extracellular | 524.4697876 | 419.4206238 | 267.5978088 | 469.8023682 | 348.6108704 | 356.0289307 |
| W5Q9A2 |  | W5Q9A2_SHEEP Alpha-2-glycoprotein 1, zinc-binding OS=Ovis aries OX=9940 PE=3 SV=1 | BP:antigen processing and presentation via MHC class Ib;BP:antigen processing and presentation of peptide antigen via MHC class Ib;BP:antigen processing and presentation of endogenous peptide antigen via MHC class Ib;BP:immune system process;BP:antigen pr | K06751 | ENOG4111CJX | PF00129.20;PF07654.17;PF16497.7 | ER | 558.9212036 | 1061.419312 | 1263.314331 | 1136.338745 | 1244.82373 | 1715.716187 |
| A0A836A8G3 | JEQ12_016456 | A0A836A8G3_SHEEP LRRCT domain-containing protein OS=Ovis aries OX=9940 GN=JEQ12_016456 PE=4 SV=1 | BP:regulation of cell proliferation;BP:regulation of anatomical structure morphogenesis;BP:cell differentiation;BP:regulation of response to stimulus;BP:positive regulation of cell proliferation;BP:regulation of vasculature development;BP:regulation of si | K13023 | COG4886 | PF13855.8;PF12799.9;PF14580.8 | extracellular | 1100.9104 | 1223.972412 | 1099.469727 | 1259.520264 | 1614.705688 | 1406.368896 |
| A0A8C2NV28 |  | A0A8C2NV28_CAPHI Complement C3 OS=Capra hircus OX=9925 PE=4 SV=1 | BP:regulation of phosphate metabolic process;BP:regulation of primary metabolic process;BP:regulation of metabolic process;BP:regulation of transport;BP:positive regulation of response to stimulus;BP:regulation of response to stimulus;BP:cell maturation;B | K03990;K03994;K03989 | ENOG410XRED | PF07678.16;PF07677.16;PF17789.3;PF01759.23;PF01835.21;PF07703.16;PF17790.3;PF17791.3;PF01821.20;PF00207.24 | cytoplasmic | 1188.172607 | 1410.786255 | 2175.727295 | 1085.14563 | 795.7252808 | 833.7015381 |
| A0A6P3E9Q2 | JEQ12_005177 | A0A6P3E9Q2_SHEEP Uncharacterized protein OS=Ovis aries OX=9940 GN=JEQ12_005177 PE=3 SV=1 | MF:aminopeptidase activity;MF:hydrolase activity;MF:transition metal ion binding;MF:cation binding;MF:catalytic activity, acting on a protein;MF:exopeptidase activity;MF:manganese ion binding;MF:ion binding;MF:catalytic activity;MF:molecular_function;MF:b | K01262;K14213 | COG0006 | PF00557.26;PF05195.18 | cytoplasmic | 61.35910034 | 87.31172943 | 76.23196411 | 71.00860596 | 39.31549454 | 68.56512451 |
| P68214 | FGA | FIBA_SHEEP Fibrinogen alpha chain (Fragment) OS=Ovis aries OX=9940 GN=FGA PE=1 SV=1 | ------ | K03903 | ------ | ------ | cytoplasmic | 36.16376114 | 12.8564167 | 20.13632774 | 67.75066376 | 16.27150536 | 19.86632919 |
| A0A452EGG7 | TREML1 | A0A452EGG7_CAPHI Triggering receptor expressed on myeloid cells like 1 OS=Capra hircus OX=9925 GN=TREML1 PE=4 SV=1 | CC:cellular_component;CC:integral component of membrane;CC:cellular anatomical entity;CC:intrinsic component of membrane | K13073;K14363 | ENOG411182M | ------ | extracellular | 84.16719055 | 56.93473816 | 48.76021576 | 29.17197227 | 42.4936409 | 59.30488205 |
| A0A6P3EIE1 | JEQ12_004008 | A0A6P3EIE1_SHEEP Uncharacterized protein OS=Ovis aries OX=9940 GN=JEQ12_004008 PE=4 SV=1 | ------ | K23817;K23816;K23815;K04004 | ENOG410ZN5E;ENOG4111AE7 | PF00084.22 | extracellular | 332.4685669 | 223.7606812 | 260.2907104 | 299.4155273 | 448.9213867 | 220.835083 |
| A0A452FZ76 | C1QA | A0A452FZ76_CAPHI Complement C1q subcomponent subunit A OS=Capra hircus OX=9925 GN=C1QA PE=4 SV=1 | BP:response to biotic stimulus;BP:synapse organization;BP:macrophage activation;BP:cell junction organization;BP:astrocyte activation;BP:cell maturation;BP:positive regulation of immune response;BP:leukocyte activation;BP:cell junction disassembly;BP:syna | K03987;K03986;K07296;K23284;K03988 | ENOG410YJJD | PF00386.23;PF01391.20 | extracellular | 1753.788696 | 1264.678589 | 1715.216431 | 1583.583618 | 1373.918213 | 1555.97644 |
| A0A6P7EHR7 | SHBG | A0A6P7EHR7_SHEEP Sex hormone binding globulin OS=Ovis aries OX=9940 GN=SHBG PE=4 SV=1 | ------ | K24337;K05464;K03908 | ENOG410YKMT | PF00054.25;PF02210.26 | ER | 326.2579956 | 484.7861938 | 310.3109131 | 343.3404541 | 255.9134064 | 247.6694336 |
| A0A836CY38 | JEQ12_004631 | A0A836CY38_SHEEP Lipopolysaccharide-binding protein OS=Ovis aries OX=9940 GN=JEQ12_004631 PE=3 SV=1 | BP:negative regulation of multicellular organismal process;BP:negative regulation of metabolic process;BP:inflammatory response;BP:regulation of metabolic process;BP:lipid transport;BP:regulation of cell activation;BP:negative regulation of cell activatio | K05399;K08761 | ENOG410Z88E | PF02886.19;PF01273.27 | plasma membrane | 212.8316803 | 56.19035339 | 81.61412048 | 70.32106018 | 368.4822388 | 161.1396637 |
| A0A452DZA7 |  | A0A452DZA7_CAPHI Ig-like domain-containing protein OS=Capra hircus OX=9925 PE=4 SV=1 | ------ | K06553 | ENOG410ZN0R | PF07686.19;PF13927.8 | cytoplasmic | 2992.079834 | 4137.248535 | 3543.002441 | 3648.545654 | 4468.047852 | 3763.766846 |
| A0A452G6C7 |  | A0A452G6C7_CAPHI Ig-like domain-containing protein OS=Capra hircus OX=9925 PE=4 SV=1 | ------ | K06553 | ENOG41117U9;ENOG410Y9T8;ENOG410ZN0R | PF07686.19;PF13927.8 | extracellular | 999.0374756 | 1114.333862 | 3629.505859 | 1587.504639 | 446.7173157 | 2069.500244 |
| A0A835ZRX6 | JEQ12_006902 | A0A835ZRX6_SHEEP Complement component C9 OS=Ovis aries OX=9940 GN=JEQ12_006902 PE=3 SV=1 | BP:cellular component assembly;BP:macromolecular complex subunit organization;BP:positive regulation of response to stimulus;BP:regulation of response to stimulus;BP:interspecies interaction between organisms;BP:regulation of biological process;BP:cellula | K03998;K04000;K03995;K03996;K03997 | ENOG410XT9A | PF01823.21;PF00057.20;PF19028.2 | extracellular | 2297.151611 | 2348.647461 | 2740.629395 | 2308.968018 | 3853.113281 | 2721.130859 |
| A0A452EPD2 | MBL2 | A0A452EPD2_CAPHI Mannose-binding protein C OS=Capra hircus OX=9925 GN=MBL2 PE=4 SV=1 | BP:regulation of immune system process;BP:anatomical structure homeostasis;BP:regulation of transport;BP:positive regulation of response to stimulus;BP:regulation of response to stimulus;BP:homeostatic process;BP:positive regulation of phagocytosis;BP:imm | K03991;K10068 | ENOG4110NRD;ENOG410YQHR | PF00059.23;PF01391.20 | extracellular | 1551.087524 | 1452.207764 | 2124.583252 | 1060.503296 | 1499.602783 | 1127.245728 |
| A0A452EP10 | KRT71 | A0A452EP10_CAPHI Keratin 71 OS=Capra hircus OX=9925 GN=KRT71 PE=3 SV=1 | BP:molting cycle process;BP:hair cycle process;BP:anatomical structure morphogenesis;BP:cellular component organization or biogenesis;BP:fibril organization;BP:cellular component organization;BP:developmental process;BP:multicellular organismal process;BP | K07605 | ENOG4111AD7 | PF00038.23;PF16208.7;PF10473.11 | cytoplasmic | 32.88703156 | 69.12284851 | 42.35677719 | 63.51064682 | 18.99702263 | 262.8684387 |
| A0A836CZD6 | JEQ12_005412 | A0A836CZD6_SHEEP Apolipoprotein C-IV OS=Ovis aries OX=9940 GN=JEQ12_005412 PE=3 SV=1 | BP:lipid transport;BP:homeostatic process;BP:triglyceride homeostasis;BP:chemical homeostasis;BP:regulation of biological process;BP:acylglycerol homeostasis;BP:establishment of localization;BP:positive regulation of lipid localization;BP:regulation of li | ------ | ENOG41118WT | PF15119.8 | cytoplasmic | 353.6253662 | 469.1695862 | 377.1028442 | 379.9964294 | 345.313385 | 474.5686951 |
| A0A452EHZ4 | ICOSLG | A0A452EHZ4_CAPHI Ig-like domain-containing protein OS=Capra hircus OX=9925 GN=ICOSLG PE=4 SV=1 | CC:cellular_component;CC:integral component of membrane;CC:cellular anatomical entity;CC:intrinsic component of membrane | K06712;K06746;K06710;K06551;K06745 | ENOG410Z67K | PF07686.19;PF13927.8;PF00047.27;PF08205.14 | peroxisomal | 53.39606094 | 33.16125488 | 23.93162155 | 20.83269691 | 27.11051369 | 23.17648315 |
| A0A452ELD4 | VTN | A0A452ELD4_CAPHI SMB domain-containing protein OS=Capra hircus OX=9925 GN=VTN PE=4 SV=1 | BP:regulation of cell adhesion;BP:cell differentiation;BP:cell-matrix adhesion;BP:glial cell differentiation;BP:regulation of biological process;BP:cellular component organization or biogenesis;BP:immune system process;BP:positive regulation of cell adhes | K07763;K06251;K08006;K24286;K01394 | ENOG410XQ5D | PF00045.21;PF01033.19 | extracellular | 2179.654785 | 1972.881958 | 2407.230957 | 450.2572632 | 212.2127686 | 1375.73999 |
| A0A835ZQ53 | JEQ12_011672 | A0A835ZQ53_SHEEP Hyaluronan-binding protein 2 OS=Ovis aries OX=9940 GN=JEQ12_011672 PE=4 SV=1 | BP:hemostasis;BP:positive regulation of response to external stimulus;BP:regulation of response to external stimulus;BP:blood coagulation;BP:regulation of response to stimulus;BP:regulation of cell migration;BP:regulation of biological process;BP:positive | K01328;K01343;K08648;K09631;K01348 | COG5640 | PF00089.28;PF00051.20;PF00008.29 | extracellular | 341.0248718 | 364.9952393 | 383.151123 | 352.8650208 | 335.2836914 | 268.4271545 |
| A0A836APE8 | JEQ12_001018 | A0A836APE8_SHEEP CD5 antigen-like OS=Ovis aries OX=9940 GN=JEQ12_001018 PE=4 SV=1 | BP:zymogen activation;BP:organic substance metabolic process;BP:proteolysis;BP:biological_process;BP:metabolic process;BP:protein processing;BP:primary metabolic process;BP:organonitrogen compound metabolic process;BP:protein maturation;BP:protein metabol | K06727 | ENOG4110209;ENOG410XQVR | PF00530.20;PF13895.8;PF13927.8;PF00047.27;PF15494.8;PF17736.3;PF07679.18;PF08205.14 | plasma membrane | 4703.676758 | 1958.002319 | 2227.141602 | 3461.024902 | 1624.624023 | 2654.651611 |
| A0A452FYY3 | APOA2 | A0A452FYY3_CAPHI Apolipoprotein A-II OS=Capra hircus OX=9925 GN=APOA2 PE=3 SV=1 | BP:regulation of metabolic process;BP:regulation of transport;BP:small molecule metabolic process;BP:cellular component organization or biogenesis;BP:positive regulation of macromolecule metabolic process;BP:response to external biotic stimulus;BP:respons | K08758 | ENOG4111552 | PF04711.15 | extracellular | 9064.642578 | 12549.35742 | 12910.1748 | 13410.85449 | 13137.48438 | 11304.54199 |
| A0A6P3YFZ8 | JEQ12_009414 | A0A6P3YFZ8_SHEEP Uncharacterized protein OS=Ovis aries OX=9940 GN=JEQ12_009414 PE=4 SV=1 | BP:hemostasis;BP:regulation of response to external stimulus;BP:negative regulation of response to stimulus;BP:blood coagulation;BP:regulation of response to stimulus;BP:regulation of wound healing;BP:liver development;BP:negative regulation of wound heal | K01314;K01320;K01321;K01344;K01313 | COG5640 | PF00089.28;PF00594.22;PF14670.8;PF00008.29 | extracellular | 183.4429169 | 170.6320801 | 175.7233429 | 139.7684937 | 138.5562134 | 119.7717667 |
| A0A452F1G2 | THBS1 | A0A452F1G2_CAPHI Thrombospondin 1 OS=Capra hircus OX=9925 GN=THBS1 PE=3 SV=1 | BP:negative regulation of multicellular organismal process;BP:negative regulation of blood vessel morphogenesis;BP:regulation of multicellular organismal process;BP:regulation of vasculature development;BP:negative regulation of vasculature development;BP | K16857;K04659 | ENOG410XQKE | PF05735.14;PF02412.20;PF00090.21;PF19028.2;PF00093.20;PF12662.9;PF12947.9;PF19030.2 | extracellular | 824.3667603 | 884.4478149 | 570.8272095 | 692.7198486 | 762.3678589 | 906.4098511 |
| A0A452F7Y5 | ALB | A0A452F7Y5_CAPHI Albumin OS=Capra hircus OX=9925 GN=ALB PE=4 SV=1 | BP:response to extracellular stimulus;BP:response to external stimulus;BP:regulation of biological process;BP:maintenance of mitochondrion location;BP:maintenance of organelle location;BP:regulation of cell death;BP:maintenance of location in cell;BP:cell | K16141;K16144;K12258 | ENOG410Z40H | PF00273.22 | extracellular | 163.4407959 | 191.8329468 | 117.3191452 | 156.5468445 | 103.0110474 | 128.391983 |
| A0A836CU14 | JEQ12_007521 | A0A836CU14_SHEEP Immunoglobulin lambda-1 light chain-like OS=Ovis aries OX=9940 GN=JEQ12_007521 PE=4 SV=1 | ------ | K06554;K06553 | ENOG410Y9T8 | PF07686.19;PF07654.17;PF13927.8;PF07679.18;PF00047.27;PF13895.8 | extracellular | 57648.57813 | 49143.87891 | 86755.32031 | 86477.02344 | 56840.95313 | 91910.41406 |
| A0A452EVP1 | APOD | A0A452EVP1_CAPHI Apolipoprotein D OS=Capra hircus OX=9925 GN=APOD PE=3 SV=1 | BP:regulation of intracellular protein transport;BP:negative regulation of lipoprotein oxidation;BP:regulation of lipoprotein oxidation;BP:regulation of metabolic process;BP:regulation of transport;BP:negative regulation of response to stimulus;BP:negativ | K18271;K03098 | COG3040 | PF00061.25;PF08212.14 | ER | 828.4569092 | 1621.289551 | 934.659668 | 1159.794434 | 867.5384521 | 972.6309204 |
