# Supplementary material for: Early Pregnancy Markers in the Serum of Ewes Identified via Proteomic and Metabolomic Analyses
Source: Int J Mol Sci. 2023 Sep 13;24(18):14054. doi: 10.3390/ijms241814054 (PMC10530974; doi:10.3390/ijms241814054)
Supplement: Supplementary file 1 [file ijms-24-14054-s001.zip › supplement tables/Table s1 List of metabolites-1345.docx]

| ID | Metabolite | Metab ID | Library ID | KEGG Compound ID | M/Z | Retention time | Mode | Adducts | Formula | Fragmentation Score | Theoretical Fragmentation Score | Mass Error | CAS ID | RSD | N1 | N2 | N3 | N4 | N5 | N6 | P1 | P2 | P3 | P4 | P5 | P6 | QC01 | QC02 | QC03 |
| --- | --- | --- | --- | --- | --- | --- | --- | --- | --- | --- | --- | --- | --- | --- | --- | --- | --- | --- | --- | --- | --- | --- | --- | --- | --- | --- | --- | --- | --- |
| neg_10 | Hippuric Acid | metab_5602 | HMDB0000714;MJDBOTE0000525 | C01586 | 178.0499 | 4.3363 | neg | M-H, M+Cl, M+FA-H, 2M-H | C9H9NO3 | 88.3 | 0 | -5.6802011 | 495-69-2 | 0.00598157 | 7.5217 | 7.7836 | 7.621 | 7.535 | 7.6962 | 7.7564 | 7.6305 | 7.5142 | 7.6826 | 7.3495 | 7.7451 | 7.7526 | 7.6506 | 7.6553 | 7.6549 |
| neg_1004 | Glycylphenylalanylleucylglycine | metab_5608 | HMDB0252827 | - | 429.1537 | 3.8367 | neg | M+K-2H | C19H28N4O5 | 0 | 50.6 | -2.2942357 | - | 0.00735226 | 4.9507 | 5.481 | 5.2463 | 5.2427 | 5.049 | 4.9742 | 5.4768 | 5.1015 | 4.8669 | 4.8535 | 4.972 | 5.1302 | 5.1865 | 5.1886 | 5.1928 |
| neg_1005 | Salicylamide glucuronide | metab_5609 | HMDB0257458 | - | 350.0301 | 3.8431 | neg | M+K-2H | C13H15NO8 | 0 | 67.8 | 5.39525169 | - | 0.02499687 | 4.749 | 4.9243 | 4.8587 | 4.9003 | 4.7801 | 4.7781 | 4.9493 | 4.8788 | 4.6893 | 4.7521 | 4.7224 | 4.7981 | 4.8407 | 4.8428 | 4.8603 |
| neg_1017 | L-quinate | metab_5622 | HMDB0304404 | C00296 | 190.05 | 3.8761 | neg | M-H | C7H11O6- | 0 | 45.8 | 8.93101 | 77-95-2 | 0.02985408 | 4.2738 | 3.8885 | 4.3035 | 4.5431 | 4.145 | 4.2915 | 3.9367 | 4.21 | 4.5906 | 4.2489 | 4.2848 | 4.5396 | 4.2913 | 4.2927 | 4.3143 |
| neg_1019 | (+)-8-epi-Altholactone | metab_5624 | - | - | 253.0461 | 3.8761 | neg | M+Na-2H | C13H12O4 | 56 | 0 | -9.0358608 | - | 0.03675531 | 4.0325 | 3.2988 | 4.0904 | 4.3487 | 3.8708 | 4.1843 | 3.5194 | 4.0136 | 4.4061 | 3.7689 | 3.8377 | 4.349 | 4.1576 | 4.157 | 4.1847 |
| neg_1027 | Pretyrosine | metab_5633 | HMDB0304809 | C00826 | 208.0606 | 3.8904 | neg | M-H2O-H | C10H13NO5 | 0 | 45.8 | -3.9804935 | 53078-86-7 | 0.02443081 | 4.9134 | 5.8095 | 5.5042 | 5.5903 | 5.2811 | 5.5191 | 5.4812 | 4.9616 | 4.9424 | 4.7099 | 5.7509 | 5.3594 | 5.3041 | 5.3062 | 5.3233 |
| neg_1029 | 5-Hydroxy-N-formylkynurenine | metab_5635 | PW_C002055;HMDB0004086 | C05648 | 251.0668 | 3.8982 | neg | M-H | C11H12N2O5 | 0 | 47.7 | -1.9844689 | - | 0.00332778 | 4.7387 | 5.1738 | 5.0016 | 4.8309 | 4.8369 | 5.0969 | 5.3289 | 3.4365 | 3.4505 | 4.3765 | 5.071 | 4.7836 | 4.7097 | 4.7084 | 4.7113 |
| neg_1036 | Glycyl-Histidine | metab_5643 | HMDB0028843 | - | 249.0375 | 3.9211 | neg | M+K-2H | C8H12N4O3 | 0 | 60.5 | -9.6629036 | - | 0.06946542 | 3.6473 | 2.9472 | 2.897 | 3.633 | 2.8891 | 3.2523 | 3.6781 | 2.9702 | 3.7005 | 2.92 | 3.738 | 4.099 | 3.8812 | 3.8299 | 3.8299 |
| neg_1044 | 2-[4-(sulfooxy)phenyl]acetic acid | metab_5651 | HMDB0132500 | - | 230.9963 | 3.9536 | neg | M-H | C8H8O6S | 0 | 54.4 | -2.6231565 | - | 0.00545789 | 4.6619 | 4.8306 | 4.5968 | 4.7232 | 5.1386 | 5.1772 | 4.678 | 4.6184 | 5.1357 | 4.4818 | 5.0865 | 5.0633 | 4.966 | 4.9681 | 4.9634 |
| neg_1048 | 4-Hydroxy-6-Methyl-2-Pyrone | metab_5655 | - | C02752 | 125.0232 | 3.9769 | neg | M-H | C6H6O3 | 90.4 | 0 | -9.965167 | 675-10-5 | 0.02390226 | 4.5246 | 4.4328 | 4.5045 | 4.4602 | 4.611 | 4.6064 | 4.5375 | 4.5235 | 4.6443 | 4.6261 | 4.5394 | 4.5452 | 4.449 | 4.4677 | 4.4506 |
| neg_1058 | Caffeic acid 4-O-sulfate | metab_5664 | HMDB0041708 | - | 258.9914 | 4.0219 | neg | M-H | C9H8O7S | 0 | 43.5 | -1.613815 | 151481-51-5 | 0.00296311 | 4.2778 | 4.4739 | 4.2322 | 4.2824 | 4.4137 | 4.3202 | 4.3412 | 4.1879 | 4.3309 | 3.8234 | 4.4127 | 4.2698 | 4.3413 | 4.3433 | 4.3437 |
| neg_1060 | Ethyl (+/-)-3-hydroxybutyrate | metab_5667 | - | - | 309.155 | 4.0297 | neg | 2M+FA-H | C6H12O3 | 0 | 54.3 | -1.7882994 | - | 0.01963571 | 4.3632 | 4.6771 | 4.6472 | 4.6046 | 4.6949 | 4.6695 | 4.605 | 4.3631 | 4.6234 | 4.7503 | 4.5013 | 4.7189 | 4.7848 | 4.8002 | 4.7863 |
| neg_1063 | Indoxyl Sulfate | metab_5670 | HMDB0000682 | - | 212.0015 | 4.048 | neg | M-H | C8H7NO4S | 69.4 | 0 | -3.6377297 | 487-94-5 | 0.01628716 | 6.0779 | 6.4027 | 6.4634 | 6.4075 | 6.5217 | 5.8194 | 6.3073 | 6.2604 | 6.4126 | 6.0077 | 6.2844 | 6.3485 | 6.2141 | 6.227 | 6.2157 |
| neg_1066 | Gabaculine | metab_5673 | HMDB0251519;HMDB0001891 | C12110 | 138.0548 | 4.0708 | neg | M-H | C7H9NO2 | 31.5 | 0 | -8.8096454 | 99-05-8;87980-11-8 | 0.02677195 | 4.0664 | 4.5307 | 4.2909 | 4.3193 | 4.2022 | 4.1046 | 4.2453 | 4.2028 | 4.09 | 4.0624 | 4.2008 | 4.0888 | 4.3367 | 4.3585 | 4.3551 |
| neg_1075 | 3-Hydroxymethylantipyrine | metab_5681 | HMDB0013840 | - | 249.0876 | 4.1216 | neg | M+FA-H | C11H12N2O2 | 0 | 56.6 | -2.4521601 | 18125-49-0 | 0.01175564 | 4.8605 | 5.2084 | 4.8576 | 4.6812 | 5.1746 | 5.2575 | 4.8578 | 4.8091 | 5.107 | 4.4561 | 5.4696 | 5.046 | 4.8951 | 4.9046 | 4.8967 |
| neg_1102 | 6-Hydroxymelatonin | metab_5712 | PW_C002052;HMDB0004081 | C05643 | 293.114 | 4.2718 | neg | M+FA-H | C13H16N2O3 | 0 | 34.3 | -1.2255741 | 2208-41-5 | 0.02714712 | 4.993 | 5.2154 | 5.1323 | 4.8466 | 5.0847 | 5.0523 | 5.1789 | 5.1499 | 4.9563 | 5.0048 | 4.9116 | 5.1553 | 5.1084 | 5.0886 | 5.1099 |
| neg_1103 | Fucosyl-GM1 | metab_5713 | HMDB0252509 | - | 732.8338 | 4.2924 | neg | M-2H | C63H109N3O35 | 0 | 68.6 | -1.3461429 | - | 0.03895808 | 5.133 | 5.8411 | 5.3378 | 5.2246 | 5.4369 | 5.0838 | 5.0259 | 5.1917 | 5.2011 | 5.3808 | 5.1751 | 4.9156 | 5.5594 | 5.5597 | 5.5299 |
| neg_1104 | Phaseoloside D | metab_5714 | HMDB0303708;HMDB0039410 | C08967 | 689.3178 | 4.3023 | neg | M-2H | C65H104O31 | 0 | 45.9 | -4.3482915 | 30937-16-7 | 0.03734501 | 5.5626 | 5.4965 | 5.6127 | 5.0348 | 5.5071 | 5.1816 | 5.1745 | 5.3764 | 5.1689 | 5.0707 | 5.3144 | 5.0544 | 5.1596 | 5.132 | 5.1316 |
| neg_1109 | Beclamide | metab_5719 | HMDB0248921 | - | 178.0427 | 4.3285 | neg | M-H2O-H | C10H12ClNO | 0 | 62.8 | -0.9508078 | - | 0.01781937 | 4.7271 | 5.0188 | 4.8572 | 4.8061 | 4.6819 | 4.9177 | 4.8447 | 4.6985 | 4.8383 | 4.5914 | 4.8704 | 4.9526 | 4.7345 | 4.7482 | 4.7477 |
| neg_1116 | Desmethyl Bosentan | metab_5726 | HMDB0251051 | - | 574.1136 | 4.3363 | neg | M+K-2H | C26H27N5O6S | 0 | 44 | -5.9463848 | - | 0.02721961 | 5.0595 | 5.5294 | 5.2628 | 4.9357 | 5.4447 | 5.4848 | 5.2118 | 4.9193 | 5.3641 | 3.7517 | 5.4633 | 5.4578 | 5.3125 | 5.3146 | 5.293 |
| neg_1120 | 5-(3',4',5'-Trihydroxyphenyl)-gamma-valerolactone-4'-O-sulphate | metab_5731 | HMDB0059987 | - | 324.9984 | 4.3441 | neg | M+Na-2H | C11H12O8S | 0 | 55.2 | -4.9763575 | - | 0.02120359 | 5.0938 | 5.162 | 5.1197 | 5.1324 | 5.0645 | 5.0904 | 5.1396 | 5.1252 | 5.0213 | 5.0696 | 5.136 | 5.0678 | 5.0755 | 5.0775 | 5.0923 |
| neg_1132 | 1-(2-Amino-3-hydroxyphenyl)-ethanone sulfate | metab_5743 | HMDB0304931 | - | 230.0123 | 4.4138 | neg | M-H | C8H9NO5S | 0 | 59.9 | -2.6113878 | - | 0.01896132 | 4.4754 | 3.9307 | 4.8245 | 4.9344 | 4.2354 | 4.2671 | 4.8565 | 5.029 | 4.0975 | 4.3152 | 4.5571 | 4.7411 | 4.3394 | 4.3543 | 4.341 |
| neg_114 | Allantoin | metab_5750 | HMDB0000462;MJDBOTE0001088 | C01551 | 157.0356 | 0.9895 | neg | M-H, M+Cl | C4H6N4O3 | 54.4 | 0 | -6.9993583 | 97-59-6 | 0.0045859 | 5.2201 | 5.7112 | 5.6495 | 5.4878 | 5.7923 | 5.6968 | 5.6219 | 5.485 | 5.6264 | 5.5475 | 5.6647 | 5.7771 | 5.6868 | 5.6888 | 5.6908 |
| neg_1143 | 2-Hydroxyhexanoic Acid | metab_5754 | HMDB0001624 | - | 131.0701 | 4.5659 | neg | M-H | C6H12O3 | 35.1 | 0 | -9.5074869 | 6064-63-7 | 0.00329307 | 4.3935 | 4.1381 | 4.271 | 4.31 | 4.0969 | 4.0028 | 4.3903 | 4.2472 | 4.0028 | 4.3852 | 4.1882 | 4.1369 | 4.2383 | 4.2412 | 4.2399 |
| neg_1154 | N-Acetylglucosamine 6-sulfate | metab_5766 | HMDB0000814 | C04132 | 322.024 | 4.6594 | neg | M+Na-2H | C8H15NO9S | 0 | 51 | 8.47772158 | 10356-99-7 | 0.00979837 | 5.0613 | 5.0717 | 5.302 | 5.2722 | 4.8158 | 4.9165 | 5.1679 | 5.3292 | 4.8546 | 4.9953 | 5.1262 | 5.0613 | 4.9663 | 4.9739 | 4.9735 |
| neg_1157 | Mometasone | metab_5769 | HMDB0014902 | C07816 | 407.1219 | 4.6648 | neg | M-H2O-H | C22H28Cl2O4 | 0 | 62.3 | 7.58366802 | 105102-22-5 | 0.14231715 | 4.4238 | 4.4605 | 4.9748 | 4.8294 | 3.5581 | 3.7027 | 4.6396 | 4.9894 | 3.6383 | 4.1263 | 4.556 | 4.39 | 4.0898 | 4.0919 | 3.9786 |
| neg_1158 | Histidylcysteine | metab_5770 | HMDB0028882 | - | 239.0588 | 4.6722 | neg | M-H2O-H | C9H14N4O3S | 0 | 34.6 | -7.8419908 | 116833-93-3 | 0.00514012 | 4.9958 | 4.9388 | 4.9694 | 5.1043 | 5.0239 | 5.0093 | 4.9679 | 4.8921 | 4.9195 | 4.9276 | 5.0604 | 5.0086 | 4.9221 | 4.9265 | 4.9236 |
| neg_1163 | 3-(4'-methylthio)butylmalate | metab_5776 | HMDB0304107 | C17219 | 233.0484 | 4.7354 | neg | M-H | C9H14O5S-2 | 0 | 41.9 | -2.1737461 | - | 0.01986813 | 3.987 | 4.1967 | 4.1157 | 4.013 | 4.1796 | 4.0766 | 4.349 | 4.0525 | 4.4552 | 3.8913 | 4.0751 | 4.29 | 3.8386 | 3.824 | 3.8234 |
| neg_1167 | 2-Methylindoline | metab_5780 | - | - | 132.0806 | 4.7738 | neg | M-H | C9H11N | 30.1 | 0 | -9.4414162 | - | 0.02479023 | 2.8723 | 4.4518 | 3.9369 | 3.9728 | 3.4434 | 3.7466 | 3.5431 | 2.9606 | 2.9063 | 2.9367 | 3.8429 | 3.4284 | 3.6951 | 3.7137 | 3.7141 |
| neg_1174 | 3-methylsulfinylpropyl-glucosinolate | metab_5787 | HMDB0304141 | - | 458.9733 | 4.814 | neg | M+K-2H | C11H20NO10S3- | 0 | 45.8 | -0.6144307 | - | 0.02480426 | 5.1319 | 5.3519 | 5.3685 | 5.5242 | 5.0124 | 4.9958 | 5.4239 | 5.4285 | 5.2177 | 5.0904 | 5.4449 | 5.3804 | 5.1876 | 5.1697 | 5.1893 |
| neg_1180 | 4-Hydroxybenzoic Acid | metab_5793 | HMDB0000500;PW_C000382;HMDB0304180;MJDBOTE0001079 | C00156 | 137.0232 | 4.8448 | neg | M-H | C7H6O3 | 54.8 | 0 | -8.8795612 | 99-96-7 | 0.00568076 | 4.4396 | 4.4705 | 4.4328 | 4.6214 | 4.576 | 4.8795 | 4.5609 | 4.4978 | 4.5906 | 4.6087 | 4.4719 | 4.5345 | 4.6252 | 4.6297 | 4.6292 |
| neg_1182 | 3,4-Dihydro-9-hydroxy-[1]benzothieno[2,3-f]-1,4-thiazepin-5(2H)-one | metab_5795 | HMDB0253770 | - | 271.9843 | 4.8448 | neg | M+Na-2H | C11H9NO2S2 | 0 | 68.9 | 8.51638047 | - | 0.00335385 | 6.1449 | 6.1875 | 6.2417 | 6.2967 | 6.1455 | 6.1391 | 6.2464 | 6.2639 | 6.1749 | 6.2061 | 6.2623 | 6.2325 | 6.1649 | 6.1669 | 6.1641 |
| neg_1188 | Phthalic Acid | metab_5800 | HMDB0002107;MJDBOTE0000627 | C01606 | 165.0183 | 4.9197 | neg | M-H | C8H6O4 | 56.9 | 0 | -6.2281545 | 88-99-3 | 0.00264341 | 5.7552 | 5.7143 | 5.768 | 5.7881 | 5.7722 | 5.8042 | 5.7458 | 5.7427 | 5.7419 | 5.8579 | 5.7243 | 5.7274 | 5.7622 | 5.7645 | 5.7633 |
| neg_1194 | N-Acetylleucine | metab_5807 | HMDB0011756 | C02710 | 172.097 | 4.964 | neg | M-H | C8H15NO3 | 69.5 | 0 | -5.5596616 | 1188-21-2 | 0.0323944 | 4.6027 | 4.8892 | 4.7085 | 4.6483 | 4.7255 | 4.8464 | 4.7836 | 4.5769 | 4.6977 | 4.7121 | 4.7497 | 4.7354 | 4.7207 | 4.7456 | 4.745 |
| neg_120 | 2-Keto-glutaramic acid | metab_5814 | HMDB0001552 | C00940 | 144.029 | 1.0678 | neg | M-H2O-H, M-H | C5H7NO4 | 0 | 37.2 | -8.1734553 | 18465-19-5 | 0.0547368 | 2.783 | 3.2854 | 3.5243 | 3.7167 | 3.3001 | 2.8981 | 3.182 | 2.8287 | 3.193 | 3.2031 | 3.5563 | 2.8282 | 3.8625 | 3.9046 | 3.904 |
| neg_1201 | Tris(2-chloroethyl) phosphate | metab_5816 | HMDB0259276 | C14445 | 318.9233 | 5.0137 | neg | M+Cl | C6H12Cl3O4P | 0 | 31.4 | 0.20656173 | - | 0.0108093 | 3.9975 | 4.8211 | 4.4617 | 4.5527 | 4.4321 | 4.6086 | 4.5496 | 3.1355 | 3.0003 | 3.2557 | 4.6437 | 4.3537 | 4.3814 | 4.3741 | 4.3829 |
| neg_1206 | 2'-Fluoro-2',3'-dideoxyinosine | metab_5820 | HMDB0245537 | - | 235.0619 | 5.064 | neg | M-H2O-H | C10H11FN4O3 | 0 | 59 | -6.8156627 | - | 0.00415742 | 4.2029 | 5.2251 | 4.8517 | 4.6572 | 4.8683 | 5.4508 | 5.1841 | 2.764 | 2.778 | 2.8084 | 5.475 | 4.8948 | 4.7241 | 4.7276 | 4.7266 |
| neg_1218 | 6-Hydroxyhexanoic Acid | metab_5831 | HMDB0012843 | C06103 | 131.0701 | 5.1413 | neg | M-H | C6H12O3 | 62.2 | 0 | -9.5005142 | 1191-25-9 | 0.00797072 | 5.4434 | 5.3444 | 5.5217 | 5.5674 | 5.51 | 5.4317 | 5.2846 | 5.4122 | 5.6778 | 4.9597 | 5.3862 | 5.4988 | 5.3562 | 5.3628 | 5.3577 |
| neg_1221 | Dehydrocyanaropicrin | metab_5835 | HMDB0035029 | - | 381.0756 | 5.1536 | neg | M+K-2H | C19H20O6 | 0 | 62.5 | 2.85390339 | 35821-02-4 | 0.01545637 | 4.6645 | 3.0154 | 4.1591 | 3.8616 | 4.0413 | 3.7868 | 4.6343 | 4.2554 | 3.3532 | 4.6199 | 3.0363 | 3.6198 | 4.4024 | 4.4147 | 4.404 |
| neg_1228 | Isoleucyl-Tryptophan | metab_5842 | HMDB0028918 | - | 316.1661 | 5.1851 | neg | M-H | C17H23N3O3 | 0 | 47.3 | -1.7758198 | - | 0.01736242 | 4.5487 | 4.9356 | 4.5548 | 5.1018 | 4.4456 | 4.2797 | 4.3013 | 4.1266 | 4.4116 | 4.723 | 4.593 | 4.7674 | 4.6975 | 4.6846 | 4.6843 |
| neg_1246 | N-Acetyl-D-phenylalanine | metab_5860 | - | C05620 | 206.0815 | 5.3415 | neg | M-H | C11H13NO3 | 46.9 | 0 | -3.7598885 | 10172-89-1 | 0.00190068 | 3.9685 | 4.7049 | 4.3519 | 4.4335 | 4.2785 | 4.5222 | 4.464 | 4.1851 | 4.2466 | 3.737 | 4.5786 | 4.4753 | 4.3872 | 4.3876 | 4.3888 |
| neg_1248 | Phenylpyruvic Acid | metab_5862 | HMDB0304081;HMDB0000205;PW_C000131;MJDBOTE0000608 | C00166 | 163.039 | 5.3514 | neg | M-H | C9H8O3 | 85.5 | 0 | -6.5021391 | 156-06-9 | 0.02552959 | 4.1552 | 4.6209 | 4.1699 | 4.3412 | 4.4566 | 4.4118 | 4.5059 | 3.981 | 4.2596 | 4.2424 | 4.3439 | 4.3217 | 4.2205 | 4.24 | 4.2396 |
| neg_1257 | Nalidixic acid | metab_5871 | HMDB0014917 | C05079 | 231.0769 | 5.4251 | neg | M-H | C12H12N2O3 | 39.4 | 0 | -2.7477185 | 389-08-2 | 0.00234256 | 4.6047 | 5.6989 | 5.2697 | 5.3893 | 5.0696 | 5.3224 | 5.3833 | 4.5793 | 4.4932 | 4.1703 | 5.4845 | 4.9627 | 5.2436 | 5.2456 | 5.2446 |
| neg_1282 | Isoferulic acid 3-sulfate | metab_5896 | HMDB0041748 | - | 273.0071 | 5.5409 | neg | M-H | C10H10O7S | 0 | 53.5 | -1.1106319 | - | 0.00339366 | 4.0524 | 4.2545 | 3.2993 | 3.9917 | 3.943 | 3.9478 | 4.0354 | 3.9781 | 3.9936 | 3.3223 | 4.1988 | 3.73 | 4.3416 | 4.3436 | 4.3408 |
| neg_1289 | N1-trans-Feruloylagmatine | metab_5900 | HMDB0037107 | C18325 | 287.1496 | 5.5701 | neg | M-H2O-H | C15H22N4O3 | 0 | 30.1 | -5.6660206 | - | 0.02190216 | 3.8625 | 4.027 | 2.8274 | 2.9486 | 4.3448 | 3.5571 | 4.0047 | 2.806 | 4.0328 | 3.252 | 3.9674 | 4.1452 | 4.4869 | 4.489 | 4.5042 |
| neg_1298 | P-Toluenesulfonic acid | metab_5905 | HMDB0059933 | C06677 | 217.0169 | 5.5885 | neg | M+FA-H | C7H8O3S | 0 | 34 | -4.1117771 | 104-15-4 | 0.00506986 | 5.7119 | 5.825 | 5.5956 | 5.4858 | 6.2271 | 6.046 | 5.685 | 5.5211 | 6.2778 | 5.0896 | 6.1465 | 6.1722 | 6.1745 | 6.1788 | 6.176 |
| neg_1313 | Cynaratriol | metab_5919 | HMDB0034983 | - | 317.1138 | 5.6822 | neg | M+Cl | C15H22O5 | 0 | 57.9 | -8.3730007 | 70894-20-1 | 0.01226747 | 4.6104 | 4.0805 | 4.6444 | 4.5128 | 3.5716 | 3.6971 | 4.5615 | 4.5143 | 3.9105 | 4.5892 | 3.5354 | 4.149 | 4.5015 | 4.5034 | 4.5115 |
| neg_1327 | Cinnamoylglycine | metab_5934 | HMDB0011621 | - | 204.0658 | 5.7563 | neg | M-H | C11H11NO3 | 72.8 | 0 | -3.86471 | 16534-24-0 | 0.00274708 | 6.288 | 6.1192 | 6.2044 | 6.1643 | 6.1146 | 6.0763 | 6.1122 | 5.9489 | 6.2592 | 6.0741 | 5.9125 | 6.0872 | 5.932 | 5.9341 | 5.9341 |
| neg_134 | Aspartyl-Glutamate | metab_5946 | HMDB0028752 | - | 243.0617 | 1.1308 | neg | M-H2O-H, M+Na-2H | C9H14N2O7 | 0 | 49.3 | -2.0825659 | - | 0.00924619 | 5.0776 | 5.0927 | 5.1335 | 5.1439 | 5.1494 | 5.0527 | 5.1174 | 5.2254 | 5.1128 | 5.2493 | 5.1055 | 5.1815 | 5.0856 | 5.0798 | 5.0876 |
| neg_1347 | Cubicin | metab_5953 | HMDB0250576 | - | 808.8403 | 5.8008 | neg | M-2H | C72H101N17O26 | 0 | 92.4 | -9.4514099 | - | 0.00312486 | 7.8401 | 7.3355 | 7.3029 | 7.1473 | 7.4181 | 7.1658 | 7.2602 | 7.6036 | 7.3409 | 7.0633 | 7.5366 | 7.3966 | 7.0007 | 7.002 | 6.9993 |
| neg_1357 | Dihydrophaseic acid | metab_5964 | HMDB0038660;LMPR0103050005 | C15971 | 317.1138 | 5.8312 | neg | M+Cl | C15H22O5 | 0 | 44.3 | -8.2558692 | 41756-77-8 | 0.03854469 | 4.7166 | 4.342 | 4.6254 | 4.7082 | 3.7889 | 3.9358 | 4.6521 | 4.6753 | 4.1715 | 4.738 | 3.2716 | 4.1405 | 4.6948 | 4.6661 | 4.6961 |
| neg_1370 | 4-Allylpyrocatechol sulfate | metab_5976 | HMDB0304934 | - | 229.017 | 5.8629 | neg | M-H | C9H10O5S | 0 | 54.5 | -2.6696995 | - | 0.0007899 | 5.2143 | 5.5987 | 5.1592 | 5.3239 | 5.5281 | 5.4956 | 5.2696 | 5.0646 | 5.723 | 5.0533 | 5.4802 | 5.552 | 5.397 | 5.3963 | 5.3968 |
| neg_1371 | Lacosamide-glucuronide | metab_5977 | HMDB0060829 | - | 473.1446 | 5.8629 | neg | M+FA-H | C18H24N2O10 | 0 | 69.3 | 7.64749686 | - | 0.00539806 | 5.3392 | 5.8144 | 5.4287 | 5.2789 | 5.7769 | 5.755 | 5.4936 | 5.434 | 5.6615 | 4.9646 | 5.5254 | 5.6414 | 5.6619 | 5.664 | 5.6593 |
| neg_1373 | Momordicasaponin I | metab_5979 | HMDB0036265 | - | 835.3532 | 5.8689 | neg | M-2H | C76H120O40 | 0 | 74 | -8.7842888 | 96552-95-3 | 0.01463918 | 6.3336 | 6.115 | 6.1885 | 6.0633 | 6.1249 | 5.9961 | 6.1275 | 6.2529 | 6.172 | 6.1409 | 6.0823 | 6.1302 | 5.7552 | 5.7578 | 5.7456 |
| neg_1377 | Folic acid | metab_5983 | PW_C000076;HMDB0000121 | C00504 | 422.1199 | 5.8762 | neg | M-H2O-H | C19H19N7O6 | 0 | 31.8 | -4.371264 | 59-30-3 | 0.02123486 | 4.0773 | 3.4754 | 3.4884 | 3.4978 | 4.109 | 4.1967 | 3.9813 | 3.8825 | 3.8352 | 3.7042 | 3.7246 | 4.1787 | 4.5495 | 4.5515 | 4.5345 |
| neg_1382 | 2-Phenylethanol glucuronide | metab_5989 | HMDB0010350 | C03033 | 297.0975 | 5.8836 | neg | M-H | C14H18O7 | 0 | 87.6 | -1.4765151 | - | 0.01991169 | 4.2836 | 4.1239 | 4.5998 | 4.6987 | 3.7377 | 3.7506 | 5.1213 | 4.6859 | 3.8425 | 4.1262 | 3.7711 | 3.8485 | 4.2219 | 4.2375 | 4.2235 |
| neg_1383 | M-Coumaric acid | metab_5990 | HMDB0001713 | C12621 | 327.0869 | 5.8836 | neg | 2M-H | C9H8O3 | 0 | 40.8 | -1.5050191 | 14755-02-3 | 0.03591374 | 4.9828 | 5.119 | 5.0688 | 4.9277 | 5.0865 | 4.8102 | 5.0702 | 5.1284 | 5.1672 | 4.7734 | 5.0048 | 5.1915 | 4.88 | 4.9075 | 4.8815 |
| neg_1389 | Phenylalanylarginine | metab_5996 | HMDB0028989 | - | 342.1552 | 5.8975 | neg | M+Na-2H | C15H23N5O3 | 0 | 54.3 | 1.43544793 | 1238-09-1 | 0.03107855 | 4.7442 | 4.325 | 4.1874 | 4.4963 | 3.8942 | 3.7572 | 3.9166 | 4.1184 | 3.732 | 4.979 | 3.7473 | 3.8068 | 5.2588 | 5.2825 | 5.26 |
| neg_1392 | Hymenoxon | metab_6000 | HMDB0253305 | C09482 | 317.1138 | 5.9019 | neg | M+Cl | C15H22O5 | 0 | 67.7 | -8.2433384 | 57377-32-9 | 0.02904192 | 4.5639 | 3.8143 | 4.335 | 4.3918 | 3.026 | 3.7569 | 4.3338 | 4.4567 | 3.7041 | 4.6249 | 3.0418 | 3.732 | 4.4525 | 4.4545 | 4.4315 |
| neg_1412 | 3-Allylphenol sulfate | metab_6022 | HMDB0304933 | - | 213.022 | 5.9427 | neg | M-H | C9H10O4S | 0 | 61.1 | -3.3196795 | - | 0.00954547 | 5.5602 | 5.9408 | 5.7475 | 5.9179 | 5.9628 | 5.9034 | 5.8046 | 5.702 | 6.0789 | 5.7388 | 6.0211 | 5.967 | 5.7574 | 5.7652 | 5.759 |
| neg_1413 | Cytochlor | metab_6023 | HMDB0246767 | - | 297.9998 | 5.9427 | neg | M+K-2H | C9H12ClN3O4 | 0 | 68 | -1.6240643 | - | 0.05074599 | 4.0054 | 4.3656 | 4.176 | 4.3502 | 4.4351 | 4.3172 | 4.2363 | 4.1084 | 4.5755 | 4.199 | 4.4706 | 4.4055 | 4.158 | 4.1212 | 4.1196 |
| neg_1418 | (S)-9-Hydroxy-10-undecenoic acid | metab_6028 | LMFA01050436;HMDB0032662 | - | 245.1389 | 5.9505 | neg | M+FA-H | C11H20O3 | 0 | 42.4 | -2.5050186 | - | 0.00325389 | 4.305 | 4.2285 | 4.1888 | 4.3143 | 4.7189 | 4.3354 | 4.4944 | 4.2411 | 4.7424 | 4.2072 | 4.623 | 4.5161 | 4.6632 | 4.6653 | 4.6626 |
| neg_1429 | 3-Hydroxybenzoic Acid | metab_6040 | HMDB0002466 | C00587 | 137.0232 | 5.9648 | neg | M-H | C7H6O3 | 72.2 | 0 | -8.6479991 | 99-06-9 | 0.00745369 | 4.9314 | 4.9811 | 4.9886 | 4.9301 | 5.2162 | 5.2959 | 4.9824 | 4.9573 | 5.189 | 5.0745 | 5.2078 | 5.2264 | 5.1885 | 5.1947 | 5.1901 |
| neg_1432 | Ecgonine Ethyl Ester | metab_6044 | HMDB0251685 | - | 258.1344 | 5.9648 | neg | M+FA-H | C11H19NO3 | 0 | 52.6 | -1.4527799 | - | 0.02602069 | 4.7987 | 3.426 | 3.6872 | 4.2698 | 3.2649 | 1.0903 | 3.8823 | 3.906 | 1.0673 | 4.4569 | 2.8541 | 3.3657 | 4.2008 | 4.1816 | 4.1812 |
| neg_1435 | BUTHIDAZOLE | metab_6047 | HMDB0249461 | C19112 | 293.0487 | 5.9648 | neg | M+K-2H | C10H16N4O2S | 0 | 47.4 | 2.88730045 | 55511-98-3 | 0.01669813 | 4.6566 | 4.7929 | 4.7293 | 4.6504 | 4.8691 | 4.6521 | 4.5145 | 4.6604 | 4.7546 | 4.54 | 4.8874 | 4.89 | 4.8346 | 4.8471 | 4.8346 |
| neg_1445 | Allylisopropylacetamide | metab_6054 | HMDB0248168 | - | 186.1127 | 5.9766 | neg | M+FA-H | C8H15NO | 0 | 51.9 | -6.3262415 | - | 0.00880629 | 4.6619 | 4.2452 | 4.5031 | 4.6173 | 4.0554 | 3.9891 | 4.6072 | 4.4649 | 4.0014 | 4.7948 | 4.0503 | 4.1869 | 4.1793 | 4.1865 | 4.1808 |
| neg_1448 | (1R,2R,3S,1'R)-Nepetalinic acid | metab_6057 | HMDB0034971 | - | 245.1025 | 5.9766 | neg | M+FA-H | C10H16O4 | 0 | 37.9 | -2.6137452 | 32603-11-5 | 0.0136546 | 3.2565 | 3.5603 | 3.2866 | 3.1566 | 3.538 | 3.4654 | 2.8328 | 3.8056 | 3.2688 | 2.9799 | 3.3572 | 3.084 | 4.9711 | 4.982 | 4.9726 |
| neg_1454 | Prostaglandin F-main urinary metabolite | metab_6064 | HMDB0256371;HMDB0258925 | - | 387.2019 | 5.9766 | neg | M+FA-H | C18H30O6 | 0 | 42.1 | -1.5155726 | - | 0.00950554 | 3.716 | 4.2102 | 3.9119 | 3.8305 | 4.9047 | 4.8797 | 4.0926 | 4.0052 | 4.8062 | 3.687 | 4.8338 | 4.8908 | 5.0033 | 5.0111 | 5.0049 |
| neg_1458 | Ketoprofen glucuronide | metab_6068 | HMDB0010334 | - | 411.1079 | 5.9844 | neg | M-H2O-H | C22H22O9 | 0 | 34.9 | -1.5104648 | 76690-94-3 | 0.03516256 | 4.7172 | 4.9741 | 5.1569 | 5.1404 | 4.618 | 4.3884 | 5.04 | 4.9873 | 4.67 | 4.9254 | 4.7253 | 5.0232 | 4.9329 | 4.907 | 4.9063 |
| neg_1462 | 5-(3',4'-Dihydroxyphenyl)-gamma-valerolactone 4'-sulfate | metab_6073 | HMDB0240473 | - | 269.0122 | 5.9923 | neg | M-H2O-H | C11H12O7S | 0 | 37.5 | -1.1657297 | - | 0.01656272 | 4.0704 | 4.1639 | 4.2187 | 4.4761 | 4.1748 | 4.1662 | 4.0088 | 4.2911 | 4.4475 | 4.2529 | 4.3288 | 4.2959 | 4.3391 | 4.3409 | 4.3523 |
| neg_1469 | N-Acetylalliin | metab_6078 | HMDB0242184 | - | 264.0542 | 6.0001 | neg | M+FA-H | C8H13NO4S | 0 | 40 | -2.536697 | - | 0.00288357 | 3.9926 | 4.4624 | 4.2876 | 4.2376 | 4.1014 | 4.615 | 4.4072 | 3.5841 | 3.5981 | 3.6285 | 4.626 | 4.0523 | 4.0955 | 4.0975 | 4.0952 |
| neg_1471 | Arginylthreonine | metab_6081 | HMDB0028719 | - | 310.1292 | 6.0001 | neg | M+Cl | C10H21N5O4 | 0 | 42.3 | 1.73284503 | 106326-78-7 | 0.02051866 | 3.6572 | 3.755 | 3.7077 | 3.8954 | 3.9458 | 3.4529 | 3.2916 | 3.8697 | 3.8433 | 3.9745 | 3.2942 | 3.8749 | 4.2896 | 4.2916 | 4.3058 |
| neg_1475 | 3-Indoleacetic Acid | metab_6084 | HMDB0000197 | C00954 | 174.0551 | 6.0079 | neg | M-H | C10H9NO2 | 66.7 | 0 | -5.5198765 | 87-51-4 | 0.0932379 | 3.4416 | 3.5869 | 3.2739 | 3.5062 | 3.657 | 3.5196 | 3.9527 | 3.5071 | 3.5109 | 3.4481 | 3.7745 | 3.9208 | 3.7726 | 3.7644 | 3.6967 |
| neg_1479 | Taurochenodeoxycholate-3-sulfate | metab_6088 | HMDB0002486;LMST05020029 | - | 288.6192 | 6.0079 | neg | M-2H | C26H45NO9S2 | 0 | 68.2 | -1.0348502 | 67030-59-5 | 0.09093767 | 4.9157 | 4.6187 | 4.1297 | 4.4598 | 4.544 | 4.2234 | 4.5186 | 4.242 | 4.0411 | 4.5532 | 4.6828 | 4.6109 | 4.0203 | 4.0236 | 4.0887 |
| neg_1480 | Menthyl pyrrolidone carboxylate | metab_6090 | HMDB0032368 | C03962 | 317.1964 | 6.0079 | neg | M-H | C16H30O6 | 0 | 32.7 | -1.8624 | 52528-10-6 | 0.02646307 | 3.9048 | 4.0793 | 3.8717 | 4.0376 | 4.1948 | 4.102 | 4.1696 | 3.9292 | 4.1853 | 4.3599 | 3.867 | 4.1189 | 4.3181 | 4.3385 | 4.3379 |
| neg_1483 | Merodesmosine | metab_6093 | HMDB0030407 | - | 439.2 | 6.0079 | neg | M+K-2H | C18H34N4O6 | 0 | 64.2 | 8.85061881 | 17096-97-8 | 0.01701846 | 5.2796 | 5.2789 | 5.4041 | 5.3485 | 5.0612 | 4.9573 | 5.2704 | 4.9852 | 5.0184 | 5.1295 | 5.1509 | 5.233 | 4.7999 | 4.7878 | 4.8013 |
| neg_1487 | Ferulic acid | metab_6097 | HMDB0000954 | C01494 | 193.0498 | 6.0158 | neg | M-H | C10H10O4 | 0 | 61.5 | -4.2459993 | 537-98-4 | 0.02244267 | 4.3037 | 4.0941 | 4.6348 | 4.62 | 3.9968 | 4.555 | 4.5517 | 4.563 | 4.3109 | 4.191 | 4.4205 | 4.0249 | 4.2243 | 4.2262 | 4.2083 |
| neg_1497 | Baptifoline | metab_6107 | HMDB0033481 | C10755 | 295.1215 | 6.0236 | neg | M+Cl | C15H20N2O2 | 0 | 40.2 | -1.4840059 | 732-50-3 | 0.08773504 | 4.4515 | 3.6509 | 4.1331 | 4.6574 | 3.6264 | 3.5866 | 3.9466 | 4.0604 | 3.5351 | 3.5833 | 3.8754 | 4.0062 | 3.6157 | 3.6229 | 3.6835 |
| neg_1499 | 17a,21-Dihydroxy-5b-pregnane-3,11,20-trione | metab_6109 | HMDB0006758;PW_C002911 | C05469 | 407.2069 | 6.0236 | neg | M+FA-H | C21H30O5 | 0 | 60 | -1.7441501 | 68-54-2 | 0.01111337 | 3.8214 | 4.981 | 4.8133 | 5.0802 | 3.8791 | 3.2583 | 4.1704 | 4.8526 | 4.8757 | 4.8235 | 4.1156 | 3.7845 | 5.0557 | 5.0476 | 5.0471 |
| neg_1502 | Hexyl salicylic acid | metab_6113 | HMDB0061943 | - | 267.1237 | 6.0315 | neg | M+FA-H | C13H18O3 | 0 | 47.3 | -0.4927346 | - | 0.01927699 | 4.3683 | 4.5957 | 4.5135 | 4.6595 | 4.5559 | 4.468 | 4.6435 | 4.4699 | 4.5451 | 4.3043 | 4.5532 | 4.6514 | 4.5664 | 4.5813 | 4.5807 |
| neg_1510 | 2-Propylglutaric Acid | metab_6120 | HMDB0060684;PW_C040548 | C16658 | 173.081 | 6.0393 | neg | M-H | C8H14O4 | 81.6 | 0 | -5.6068755 | 32806-62-5 | 0.00668572 | 4.7226 | 4.7453 | 4.7408 | 4.766 | 4.7121 | 4.6856 | 4.8649 | 4.7623 | 4.7524 | 4.7487 | 4.8162 | 4.8528 | 4.6351 | 4.6342 | 4.6396 |
| neg_1511 | Indole-5,6-quinone | metab_6121 | HMDB0006779;PW_C002926 | C05579 | 192.0307 | 6.0393 | neg | M+FA-H | C8H5NO2 | 0 | 51.9 | 3.09623361 | 582-59-2 | 0.0561775 | 2.9557 | 3.937 | 3.4618 | 2.9957 | 3.6742 | 4.376 | 4.1573 | 2.965 | 2.9789 | 3.0094 | 4.5098 | 3.3823 | 3.7156 | 3.7498 | 3.7635 |
| neg_1514 | N-OCTANOYL-L-HOMOSERINE LACTONE | metab_6124 | HMDB0255213 | C21199 | 272.1502 | 6.0393 | neg | M+FA-H | C12H21NO3 | 0 | 55.3 | -0.7082613 | 147852-84-4 | 0.00572964 | 5.5883 | 5.1611 | 5.3743 | 5.5309 | 5.0671 | 4.9053 | 4.997 | 5.2538 | 4.9329 | 5.4428 | 4.9621 | 5.1962 | 5.2588 | 5.2547 | 5.2543 |
| neg_1520 | Pyrazosulfuron-ethyl | metab_6131 | HMDB0256958 | C18444 | 459.0928 | 6.0393 | neg | M+FA-H | C14H18N6O7S | 0 | 56.8 | -2.7205881 | 93697-74-6 | 0.00355136 | 5.0546 | 4.966 | 5.1745 | 5.0432 | 5.0206 | 5.0185 | 5.0307 | 5.1258 | 4.9998 | 5.3024 | 4.9807 | 4.9469 | 4.9912 | 4.9916 | 4.9887 |
| neg_1536 | Histidylmethionine | metab_6147 | HMDB0028891 | - | 321.0818 | 6.0628 | neg | M+Cl | C11H18N4O3S | 0 | 61.1 | 8.55713153 | 11/1/88 | 0.0197667 | 4.0113 | 4.6204 | 4.5499 | 4.404 | 4.3763 | 4.3704 | 4.527 | 3.8644 | 4.0285 | 3.6437 | 4.6505 | 4.5877 | 4.4054 | 4.4075 | 4.3916 |
| neg_1548 | Betavulgarin | metab_6159 | HMDB0303334;LMPK12050361 | C10201 | 357.0612 | 6.0706 | neg | M+FA-H | C17H12O6 | 0 | 40 | -1.3642451 | 51068-94-1 | 0.00461093 | 5.2704 | 5.256 | 5.3154 | 5.3274 | 5.2851 | 5.3246 | 5.3154 | 5.381 | 5.3002 | 5.417 | 5.2851 | 5.3011 | 5.3 | 5.304 | 5.3016 |
| neg_1550 | Gamma-CEHC glucuronide | metab_6162 | HMDB0242175 | - | 421.15 | 6.0706 | neg | M-H2O-H | C21H28O10 | 0 | 55 | -1.0345246 | - | 0.00495351 | 5.0656 | 5.4387 | 5.2372 | 5.3012 | 5.548 | 5.5368 | 5.2802 | 5.1536 | 5.4529 | 5.0327 | 5.5724 | 5.5909 | 5.3732 | 5.3764 | 5.3724 |
| neg_1563 | MG(14:1(9Z)/0:0/0:0) | metab_6175 | HMDB0011562 | - | 345.2277 | 6.0863 | neg | M+FA-H | C17H32O4 | 0 | 49.7 | -1.7644964 | - | 0.00750409 | 3.978 | 4.6253 | 4.4808 | 4.3157 | 4.6312 | 4.3263 | 4.7084 | 4.2266 | 4.5823 | 4.6719 | 4.5775 | 4.6817 | 4.7927 | 4.7985 | 4.7981 |
| neg_1574 | Ipratropium bromide | metab_6184 | HMDB0249401;HMDB0014476 | C07052 | 353.1997 | 6.0941 | neg | M+Na-2H | C20H30NO3+ | 0 | 50.5 | 7.28431899 | 60205-81-4 | 0.01012379 | 4.5058 | 5.0709 | 5.0528 | 4.6702 | 4.6382 | 5.0272 | 4.9808 | 4.6225 | 4.9343 | 4.3954 | 4.9409 | 4.8186 | 4.6984 | 4.7063 | 4.7058 |
| neg_1580 | Ancymidol | metab_6191 | HMDB0248390 | C18774 | 255.1137 | 6.102 | neg | M-H | C15H16N2O2 | 0 | 46.3 | -0.7438347 | 12771-68-5 | 0.03606917 | 3.8094 | 0.345 | 4.0988 | 3.8882 | 0.3478 | 2.3164 | 3.9492 | 4.2395 | 3.4166 | 4.1907 | 0.3566 | 2.8427 | 4.1842 | 4.2148 | 4.1943 |
| neg_1583 | TXB2 | metab_6194 | LMFA03030002;HMDB0003252 | C05963 | 369.2277 | 6.102 | neg | M-H | C20H34O6 | 45 | 0 | -1.5169158 | 54397-85-2 | 0.00755383 | 5.5457 | 5.2633 | 5.2939 | 5.3441 | 5.2111 | 5.1206 | 5.4143 | 5.6106 | 5.1589 | 4.8779 | 4.5971 | 5.705 | 5.5564 | 5.5627 | 5.558 |
| neg_1588 | 3-Carboxy-4-methyl-5-ethyl-2-furanpropionic acid | metab_6199 | HMDB0244411 | - | 225.0762 | 6.1098 | neg | M-H | C11H14O5 | 0 | 34.9 | -2.9633674 | - | 0.01571432 | 4.2104 | 4.5361 | 4.4019 | 4.701 | 4.9465 | 4.7253 | 4.6827 | 4.4108 | 5.059 | 4.4998 | 4.9116 | 4.9619 | 4.875 | 4.8875 | 4.8766 |
| neg_1594 | Ergocornine | metab_6204 | HMDB0251906 | C09162 | 582.2715 | 6.1098 | neg | M+Na-2H | C31H39N5O5 | 0 | 67.9 | 2.98640027 | 564-36-3 | 0.01413142 | 5.4948 | 5.6531 | 4.8172 | 4.7652 | 5.9136 | 5.8419 | 5.3142 | 4.9154 | 5.7502 | 5.897 | 5.6675 | 5.5841 | 5.4034 | 5.4147 | 5.4049 |
| neg_1605 | 3-Hydroxyhept-4-enoylcarnitine | metab_6216 | HMDB0241685 | - | 286.1657 | 6.1255 | neg | M-H | C14H25NO5 | 0 | 38.5 | -1.1182471 | - | 0.03126138 | 4.2553 | 2.964 | 3.6534 | 4.1882 | 3.045 | 2.9948 | 3.5731 | 3.9047 | 2.9696 | 3.9984 | 2.985 | 3.4722 | 4.3948 | 4.3715 | 4.3715 |
| neg_1606 | O-Demethylmetoprolol | metab_6217 | HMDB0255850 | - | 298.1655 | 6.1255 | neg | M+FA-H | C14H23NO3 | 0 | 49.8 | -2.0529325 | - | 0.01071413 | 5.1167 | 4.4904 | 4.8336 | 4.7852 | 4.7244 | 4.3695 | 4.1666 | 4.9867 | 4.6515 | 4.9279 | 4.7046 | 4.9684 | 5.2274 | 5.2239 | 5.2181 |
| neg_1612 | 1,4-Dihydroxy-2-naphthoic acid | metab_6223 | HMDB0244212 | C03657 | 203.0342 | 6.1333 | neg | M-H | C11H8O4 | 68.5 | 0 | -3.9080021 | 31519-22-9 | 0.00613055 | 5.0013 | 5.0847 | 4.9689 | 5.1527 | 5.1665 | 5.052 | 5.1272 | 5.035 | 5.3025 | 4.9831 | 5.194 | 5.2889 | 5.5643 | 5.5695 | 5.5659 |
| neg_1617 | 6-keto PGE1 | metab_6227 | HMDB0004241;LMFA03010012 | C05962 | 367.2153 | 6.1333 | neg | M-H | C20H32O6 | 65.2 | 0 | 7.2329785 | 67786-53-2 | 0.01585715 | 5.8455 | 6.1344 | 6.2458 | 6.4454 | 5.8225 | 6.2342 | 6.1754 | 6.0213 | 6.1639 | 5.6747 | 6.2192 | 6.2114 | 6.0005 | 6.0131 | 6.002 |
| neg_1621 | 3-Methylsuberic acid | metab_6232 | LMFA01030981;HMDB0059783 | - | 169.086 | 6.1411 | neg | M-H2O-H | C9H16O4 | 0 | 31 | -5.3025473 | - | 0.00132267 | 4.522 | 4.4176 | 4.4472 | 4.4311 | 4.3776 | 4.3936 | 4.4955 | 4.6217 | 4.3928 | 4.2806 | 4.2339 | 4.7182 | 4.599 | 4.5984 | 4.5979 |
| neg_1624 | Diethyl Phthalate | metab_6234 | HMDB0094660 | C14175 | 221.0812 | 6.1411 | neg | M-H | C12H14O4 | 60.3 | 0 | -3.3655769 | 84-66-2 | 0.00318005 | 5.0513 | 5.0559 | 5.0831 | 5.2305 | 5.2874 | 5.1117 | 5.1234 | 5.1007 | 5.0274 | 5.0782 | 5.2109 | 5.2643 | 4.9158 | 4.9179 | 4.9152 |
| neg_1626 | B-Octylglucoside | metab_6236 | HMDB0245753 | - | 273.1705 | 6.1411 | neg | M-H2O-H | C14H28O6 | 0 | 39.5 | -0.9079614 | - | 0.0030489 | 5.0104 | 5.0198 | 5.1026 | 5.1047 | 5.161 | 5.1407 | 5.1031 | 4.9689 | 5.3511 | 5.0073 | 5.2627 | 5.2203 | 5.1263 | 5.1283 | 5.1288 |
| neg_1627 | 2-Propyl-2,4-pentadienoic acid | metab_6237 | HMDB0060682;PW_C040569 | C16656 | 325.1653 | 6.1411 | neg | 2M+FA-H | C8H12O2 | 0 | 40 | -1.4414599 | 72010-18-5 | 0.00839465 | 4.3136 | 4.632 | 4.3477 | 4.4576 | 4.7265 | 4.2638 | 4.4455 | 4.1338 | 4.6651 | 4.1367 | 4.6238 | 4.6798 | 4.448 | 4.4412 | 4.4424 |
| neg_1628 | Kukoamine B | metab_6238 | HMDB0060525 | C17616 | 511.2903 | 6.1411 | neg | M-H2O-H | C28H42N4O6 | 0 | 53.3 | -4.3148928 | - | 0.01555705 | 5.4753 | 5.0352 | 5.2726 | 5.2338 | 5.2078 | 5.3971 | 5.6481 | 5.3666 | 5.3262 | 4.7824 | 5.3168 | 5.3214 | 5.2094 | 5.1983 | 5.1973 |
| neg_1638 | Pinometostat | metab_6249 | HMDB0256563 | - | 583.3118 | 6.149 | neg | M+Na-2H | C30H42N8O3 | 0 | 53.7 | -1.4896218 | - | 0.07442342 | 3.8413 | 5.22 | 4.9477 | 4.5315 | 4.9595 | 5.1798 | 4.9269 | 4.5344 | 5.2793 | 4.0744 | 4.9622 | 4.7334 | 4.8493 | 4.9049 | 4.8507 |
| neg_1646 | 9'-Carboxy-gamma-chromanol | metab_6258 | HMDB0012868 | - | 421.2594 | 6.1568 | neg | M+FA-H | C23H36O4 | 0 | 62.8 | -0.3219717 | 1215088-64-4 | 0.03095619 | 3.8396 | 4.4046 | 4.5618 | 3.9124 | 4.8597 | 4.9379 | 4.4006 | 4.1298 | 5.1544 | 3.8504 | 5.0514 | 4.5634 | 5.2093 | 5.2114 | 5.1869 |
| neg_1649 | Senkyunolide N | metab_6261 | HMDB0302272 | - | 207.1019 | 6.1646 | neg | M-H2O-H | C12H18O4 | 0 | 49.2 | -3.3872915 | - | 0.01187669 | 4.5252 | 4.5848 | 4.6224 | 4.6538 | 4.6046 | 4.6386 | 4.6584 | 4.6614 | 4.5928 | 4.6986 | 4.5821 | 4.6681 | 4.6531 | 4.6551 | 4.6453 |
| neg_1650 | (Z)-4-Hydroxy-6-dodecenoic acid lactone | metab_6263 | LMFA07040067;HMDB0032331 | C03107 | 241.144 | 6.1646 | neg | M+FA-H | C12H20O2 | 0 | 37.9 | -2.7007091 | 18679-18-0 | 0.01054989 | 4.8805 | 4.797 | 4.7227 | 4.8882 | 4.746 | 4.6963 | 5.0713 | 4.6551 | 4.7399 | 4.9874 | 4.7664 | 4.8383 | 4.8673 | 4.8694 | 4.8606 |
| neg_1654 | N-Docosahexaenoyl Glutamine | metab_6267 | HMDB0242012;LMFA08020129 | - | 493.2437 | 6.1646 | neg | M+K-2H | C27H40N2O4 | 0 | 36.9 | -8.0777883 | - | 0.00615181 | 5.4913 | 5.9561 | 5.8791 | 5.8619 | 6.0487 | 6.1152 | 6.2394 | 5.7811 | 5.8993 | 5.5289 | 6.0716 | 6.1037 | 5.8037 | 5.8057 | 5.8004 |
| neg_1660 | Hygromycin B | metab_6274 | HMDB0034229 | C01925 | 572.2345 | 6.1725 | neg | M+FA-H | C20H37N3O13 | 0 | 44.9 | 6.95827959 | 31282-04-9 | 0.01003279 | 4.8343 | 4.4209 | 4.4962 | 4.7429 | 4.3876 | 4.3759 | 4.8037 | 4.2276 | 4.4574 | 4.6819 | 4.5378 | 4.4941 | 5.0486 | 5.0413 | 5.0408 |
| neg_1670 | 1-Arachidonoylglycerol | metab_6284 | HMDB0243830;LMGL01010032;HMDB0011578 | C13857 | 423.2748 | 6.1803 | neg | M+FA-H | C23H38O4 | 0 | 71.2 | -1.1651493 | 124511-15-5 | 0.00685189 | 3.3931 | 5.3016 | 5.124 | 5.0292 | 5.0242 | 5.4887 | 5.0515 | 4.672 | 5.4218 | 4.1933 | 5.6581 | 4.6789 | 5.3509 | 5.3562 | 5.3559 |
| neg_1674 | Cuminaldehyde | metab_6288 | HMDB0002214 | C06577 | 193.0862 | 6.1881 | neg | M+FA-H | C10H12O | 0 | 31.7 | -5.7630553 | 122-03-2 | 0.00832951 | 5.0845 | 5.0358 | 5.1267 | 5.1219 | 5.0472 | 5.1157 | 5.0977 | 5.1235 | 5.0724 | 5.182 | 5.0867 | 5.1153 | 5.0922 | 5.0943 | 5.0992 |
| neg_1677 | 2-cis,4-trans-xanthoxin | metab_6291 | HMDB0304066 | C13453 | 249.1491 | 6.1881 | neg | M-H | C15H22O3 | 0 | 38.8 | -2.0333705 | 7/7/66 | 0.00908285 | 3.6832 | 3.3256 | 3.4093 | 3.5646 | 3.7873 | 4.0065 | 3.3588 | 3.4875 | 3.9693 | 3.67 | 3.8086 | 3.9947 | 4.5059 | 4.5133 | 4.5074 |
| neg_1678 | 2-Hydroxy-5-octenoylcarnitine | metab_6292 | HMDB0241703 | - | 300.1812 | 6.1881 | neg | M-H | C15H27NO5 | 0 | 41.6 | -1.5513326 | - | 0.02277126 | 5.3208 | 4.1905 | 4.7965 | 5.2437 | 3.924 | 3.1322 | 4.0867 | 4.8386 | 3.9433 | 5.0857 | 4.1566 | 4.4347 | 5.2734 | 5.2567 | 5.2561 |
| neg_1686 | 4-Hydroxyphenylacetylglutamic acid | metab_6299 | HMDB0006061 | C05595 | 262.0744 | 6.2038 | neg | M-H2O-H | C13H15NO6 | 0 | 34.1 | 8.24404422 | 1029120-37-3 | 0.1091891 | 4.3047 | 4.0008 | 4.2327 | 4.1088 | 4.0038 | 4.1459 | 4.1355 | 4.186 | 4.0809 | 4.4175 | 4.0768 | 4.1339 | 4.0215 | 4.032 | 4.1062 |
| neg_1689 | Indan-1-ol | metab_6302 | HMDB0059601 | C01710 | 179.0704 | 6.2116 | neg | M+FA-H | C9H10O | 0 | 49.6 | -7.0016788 | - | 0.00985357 | 4.439 | 4.6727 | 4.6482 | 4.6638 | 4.6561 | 4.5182 | 4.7067 | 4.5059 | 4.807 | 4.5321 | 4.6441 | 4.7629 | 5.0311 | 5.0387 | 5.0382 |
| neg_1690 | Isokobusone | metab_6304 | HMDB0036791 | C16977 | 267.1599 | 6.2116 | neg | M+FA-H | C14H22O2 | 0 | 52.8 | -1.3691528 | 24173-72-6 | 0.00447871 | 4.7546 | 4.8569 | 4.7406 | 4.8444 | 5.052 | 4.8427 | 4.9553 | 4.6339 | 5.0114 | 4.8583 | 5.0036 | 5.0534 | 4.8856 | 4.8892 | 4.8887 |
| neg_1691 | DG(8:0/8:0/0:0) | metab_6305 | HMDB0116368;PW_C062137 | C00165 | 389.2547 | 6.2116 | neg | M+FA-H | C19H36O5 | 0 | 55.6 | 0.75005698 | - | 0.06184963 | 4.2751 | 4.7516 | 4.7284 | 4.724 | 4.5494 | 4.789 | 4.8248 | 4.4229 | 4.7912 | 4.2964 | 4.768 | 4.7809 | 4.8348 | 4.7898 | 4.8393 |
| neg_1692 | Pindolol | metab_6306 | PW_C009401;HMDB0015095;MJDBOTE0000319 | C07445 | 495.2955 | 6.2116 | neg | 2M-H | C14H20N2O2 | 0 | 60.2 | -4.3523514 | 13523-86-9 | 0.00418856 | 6.3115 | 6.1693 | 6.3702 | 6.0159 | 5.9238 | 6.374 | 6.4594 | 6.2742 | 6.2268 | 5.951 | 6.3093 | 5.8638 | 5.976 | 5.9731 | 5.9727 |
| neg_1705 | Coutaric acid | metab_6321 | HMDB0029225 | C01327 | 386.146 | 6.2195 | neg | M+K-2H | C18H27N3O4 | 0 | 44.8 | -7.8051405 | - | 0.02588178 | 4.6845 | 4.7418 | 4.8604 | 4.6022 | 4.5963 | 4.2687 | 4.6625 | 4.6405 | 4.7703 | 4.4975 | 4.5506 | 4.8394 | 5.0615 | 5.0422 | 5.0422 |
| neg_1727 | PE-NMe(18:0/18:3(6Z,9Z,12Z)) | metab_6343 | PW_C059213;HMDB0113090 | C01241 | 800.5439 | 6.2351 | neg | M+FA-H | C42H78NO8P | 0 | 90.3 | -1.0243429 | - | 0.08917298 | 3.5568 | 5.0839 | 4.8308 | 5.262 | 4.86 | 4.8628 | 4.8637 | 5.229 | 4.8003 | 5.0111 | 4.908 | 4.7694 | 5.5637 | 5.5112 | 5.4895 |
| neg_1729 | Tuberose lactone | metab_6345 | HMDB0303314 | - | 239.1283 | 6.2429 | neg | M+FA-H | C12H18O2 | 0 | 37.2 | -2.7646205 | - | 0.00668441 | 5.5923 | 5.5909 | 5.6366 | 5.6499 | 5.6278 | 5.642 | 5.642 | 5.6495 | 5.6323 | 5.701 | 5.6033 | 5.6308 | 5.6175 | 5.6228 | 5.6223 |
| neg_1731 | 2-Hydroxyoctadecanoic acid | metab_6348 | HMDB0062549;HMDB0242145 | C03045 | 345.2642 | 6.2429 | neg | M+FA-H | C18H36O3 | 0 | 56.7 | -1.6030388 | 26531-80-6 | 0.07429728 | 4.4154 | 5.2406 | 4.8724 | 4.8931 | 4.9645 | 5.0977 | 5.0059 | 4.5518 | 5.2568 | 4.7191 | 4.8875 | 4.9397 | 4.9795 | 4.9464 | 4.915 |
| neg_1743 | 9-F1-phytoprostane | metab_6360 | HMDB0304641 | - | 327.2173 | 6.2586 | neg | M-H | C18H32O5 | 0 | 79.8 | -1.1746413 | - | 0.00438077 | 5.4096 | 5.5821 | 5.5167 | 5.484 | 5.5979 | 5.4412 | 5.6214 | 5.3551 | 5.5684 | 5.5329 | 5.597 | 5.4989 | 5.9009 | 5.8979 | 5.8974 |
| neg_1765 | 6alpha-hydroxy-castasterone | metab_6378 | HMDB0304235 | C15803 | 447.3513 | 6.2903 | neg | M-H2O-H | C28H50O5 | 0 | 32 | 7.1322209 | - | 0.00529932 | 6.0492 | 6.0096 | 6.0335 | 6.0967 | 6.0222 | 6.0481 | 5.9958 | 6.0302 | 5.9885 | 6.1216 | 6.03 | 6.0154 | 6.0714 | 6.0735 | 6.076 |
| neg_1769 | Myristoleic acid | metab_6382 | HMDB0002000;LMFA01030051 | C08322 | 271.1912 | 6.2981 | neg | M+FA-H | C14H26O2 | 0 | 71.8 | -1.4128421 | 544-64-9 | 0.00404041 | 5.2073 | 5.0513 | 5.0066 | 5.4686 | 4.7641 | 4.6024 | 5.3632 | 4.8414 | 4.7919 | 5.3409 | 4.8331 | 4.8805 | 5.2259 | 5.2239 | 5.2274 |
| neg_1794 | Armillarin | metab_6408 | HMDB0039380 | - | 459.205 | 6.3295 | neg | M+FA-H | C24H30O6 | 0 | 36.6 | 6.09944023 | 83329-14-0 | 0.0239729 | 5.0458 | 5.1231 | 5.2093 | 5.1132 | 4.8989 | 5.2138 | 4.9823 | 4.9248 | 5.1527 | 4.7744 | 5.1351 | 5.0318 | 5.1588 | 5.142 | 5.1613 |
| neg_1797 | Dinoterb | metab_6411 | - | C18671 | 239.0668 | 6.3373 | neg | M-H | C10H12N2O5 | 58.4 | 0 | -2.3234436 | 1420-07-1 | 0.01579663 | 4.7226 | 4.7464 | 4.758 | 4.7643 | 4.766 | 4.7722 | 4.7579 | 4.7645 | 4.7176 | 4.7822 | 4.7608 | 4.7575 | 4.7536 | 4.7425 | 4.755 |
| neg_1801 | PC(16:0/18:3(6Z,9Z,12Z)) | metab_6417 | PW_C004023;HMDB0007974;LMGP01010598 | C00157 | 800.544 | 6.3373 | neg | M+FA-H | C42H78NO8P | 0 | 97.6 | -1.000533 | 203393-39-9 | 0.03357863 | 4.6946 | 5.4195 | 5.0341 | 5.4981 | 5.4473 | 5.2763 | 5.2984 | 5.5223 | 5.1791 | 5.3702 | 5.4142 | 5.2446 | 6.0023 | 5.9799 | 5.9753 |
| neg_1808 | Gamma-Linolenic acid | metab_6424 | PW_C001772;LMFA01030141;HMDB0003073 | C06426 | 323.2219 | 6.3457 | neg | M+FA-H | C18H30O2 | 0 | 51.4 | -3.0679782 | 506-26-3 | 0.00721828 | 4.3478 | 4.6394 | 4.4784 | 4.5659 | 4.5213 | 4.2887 | 4.5625 | 4.1235 | 4.4659 | 4.4724 | 4.3605 | 4.3161 | 4.8209 | 4.8157 | 4.8152 |
| neg_1813 | Adrenic Acid | metab_6430 | LMFA04000050;PW_C001506;LMFA01030178;HMDB0002226 | C16527 | 377.2696 | 6.3536 | neg | M+FA-H | C22H36O2 | 68.1 | 0 | -0.3269256 | 28874-58-0 | 0.00725236 | 4.772 | 4.9086 | 5.0194 | 4.9281 | 4.8326 | 4.9016 | 5.0352 | 4.7061 | 4.9668 | 4.6269 | 5.0603 | 4.8167 | 4.7538 | 4.7557 | 4.7495 |
| neg_1821 | 9(S)-HODE | metab_6437 | HMDB0004670 | C14767 | 295.2274 | 6.3692 | neg | M-H | C18H32O3 | 49 | 0 | -1.651772 | 73543-67-6 | 0.03851916 | 5.0298 | 5.1302 | 4.9934 | 5.0552 | 4.7537 | 4.9247 | 5.2878 | 5.1318 | 4.7423 | 5.1943 | 4.7023 | 4.9307 | 4.9684 | 4.9401 | 4.9395 |
| neg_183 | (2R,4S,5R)-2,4,5,6-Tetrahydroxyhexanal | metab_6445 | HMDB0242540 | - | 185.0422 | 3.5882 | neg | M-H, M+Na-2H | C6H12O5 | 0 | 31.2 | -5.9110265 | - | 0.00055573 | 5.0439 | 4.9519 | 5.029 | 4.956 | 5.1112 | 5.1089 | 4.9054 | 4.9111 | 5.1649 | 5.0335 | 5.1193 | 5.1601 | 5.1894 | 5.1897 | 5.1892 |
| neg_1830 | Palmitoleic acid | metab_6446 | HMDB0003229;HMDB0012328;LMFA01030056 | C08362 | 299.2223 | 6.3927 | neg | M+FA-H | C16H30O2 | 0 | 63.3 | -1.9567876 | 373-49-9;10030-73-6 | 0.00879048 | 5.284 | 5.1829 | 5.2239 | 5.4122 | 4.7487 | 4.6881 | 5.4563 | 5.1744 | 4.9426 | 5.4847 | 4.8673 | 4.9119 | 5.2062 | 5.1999 | 5.1993 |
| neg_1831 | Deoxycholic Acid | metab_6447 | HMDB0000626;LMST04010040;PW_C000487;MJDBOTE0000359 | C04483 | 391.285 | 6.3927 | neg | M-H | C24H40O4 | 92.9 | 0 | -1.0793902 | 83-44-3 | 0.00562007 | 5.4506 | 5.8147 | 5.8815 | 5.5753 | 5.8798 | 6.0094 | 6.0317 | 5.7614 | 6.0776 | 5.586 | 5.6314 | 6.1176 | 5.8018 | 5.8038 | 5.799 |
| neg_1837 | Stearidonic acid | metab_6451 | LMFA01030357;PW_C002804;HMDB0006547 | C16300 | 321.2064 | 6.4005 | neg | M+FA-H | C18H28O2 | 0 | 40.9 | -2.5489732 | 20290-75-9 | 0.00973298 | 4.0242 | 3.8111 | 3.7617 | 4.086 | 3.1215 | 3.065 | 4.1698 | 3.7757 | 3.0399 | 4.2891 | 3.0552 | 3.0511 | 4.6527 | 4.6457 | 4.6452 |
| neg_1839 | 25-Acetylvulgaroside | metab_6453 | HMDB0041365 | - | 459.2728 | 6.4005 | neg | M-H2O-H | C27H42O7 | 0 | 75 | -5.007912 | 172616-88-5 | 0.00471168 | 4.7752 | 5.0469 | 5.0822 | 4.6595 | 5.073 | 5.1963 | 5.2467 | 5.0225 | 5.2648 | 4.6693 | 4.9058 | 5.2932 | 5.0159 | 5.0179 | 5.02 |
| neg_1862 | Deoxycorticosterone acetate | metab_6478 | HMDB0251003 | C14554 | 353.2117 | 6.4403 | neg | M-H2O-H | C23H32O4 | 0 | 56 | -1.3655196 | 56-47-3 | 0.00237975 | 5.1608 | 5.1438 | 5.0423 | 4.8428 | 5.6534 | 5.1643 | 4.7672 | 5.5238 | 5.4335 | 4.8798 | 4.7741 | 5.0264 | 5.6688 | 5.6708 | 5.6695 |
| neg_1864 | Cyclohexaneundecanoic acid | metab_6480 | HMDB0030997 | C12100 | 313.2381 | 6.4481 | neg | M+FA-H | C17H32O2 | 0 | 64.8 | -1.3744012 | 4277-62-7 | 0.00415902 | 6.4396 | 6.2938 | 6.3336 | 6.5408 | 5.9175 | 5.8696 | 6.5546 | 6.2581 | 6.0524 | 6.5341 | 6.0736 | 6.081 | 6.239 | 6.2424 | 6.2418 |
| neg_1866 | Xi-Dihydro-5-octyl-2(3H)-furanone | metab_6481 | HMDB0031683 | - | 441.3216 | 6.4481 | neg | 2M+FA-H | C12H22O2 | 0 | 66.2 | -1.4889782 | 57084-18-1 | 0.05354269 | 4.6489 | 4.9171 | 4.7592 | 4.6847 | 4.7703 | 4.7187 | 4.8782 | 4.5161 | 4.8092 | 4.5925 | 4.8676 | 4.7538 | 4.9013 | 4.8614 | 4.9034 |
| neg_1873 | PE(18:2/0:0) | metab_6488 | - | - | 476.2776 | 6.4638 | neg | M-H | C23H44NO7P | 96.3 | 0 | -1.3389124 | - | 0.00402944 | 5.8672 | 6.1904 | 6.141 | 5.9646 | 6.0461 | 6.1931 | 5.9781 | 5.8538 | 6.0944 | 5.8981 | 5.9837 | 5.9651 | 6.036 | 6.0332 | 6.0328 |
| neg_1875 | 4-Decan-4-Ylbenzenesulfonic Acid | metab_6490 | - | - | 297.1525 | 6.4716 | neg | M-H | C16H26O3S | 74.6 | 0 | -1.6213136 | - | 0.01358612 | 5.4768 | 5.471 | 5.4455 | 5.5814 | 5.4761 | 5.5359 | 5.6629 | 5.4043 | 5.4817 | 5.6463 | 5.6016 | 5.6367 | 5.7004 | 5.7025 | 5.7115 |
| neg_1877 | 1-Oleoylglycerophosphoserine | metab_6492 | HMDB0061694 | - | 544.2657 | 6.4716 | neg | M+Na-2H | C24H46NO9P | 0 | 71.6 | 0.09714646 | - | 0.01890516 | 4.6 | 4.9556 | 4.8282 | 4.8524 | 4.895 | 5.0358 | 4.5689 | 4.5886 | 4.8397 | 4.5057 | 4.665 | 4.6396 | 4.7931 | 4.8077 | 4.8072 |
| neg_1880 | Sterculic acid | metab_6496 | LMFA01140018;HMDB0258495;HMDB0304490 | C08366 | 339.2535 | 6.4795 | neg | M+FA-H | C19H34O2 | 0 | 66.9 | -2.1209699 | 738-87-4 | 0.01242133 | 4.688 | 4.752 | 4.7788 | 4.7608 | 4.5409 | 4.3182 | 4.834 | 4.4845 | 4.744 | 4.6658 | 4.5875 | 4.5955 | 4.7339 | 4.736 | 4.7257 |
| neg_1884 | LysoPC(22:5(7Z,10Z,13Z,16Z,19Z)/0:0) | metab_6500 | HMDB0010403 | C04230 | 614.3458 | 6.4795 | neg | M+FA-H | C30H52NO7P | 0 | 87.4 | -1.0251864 | - | 0.00207917 | 6.3014 | 6.243 | 6.2808 | 6.1887 | 6.3662 | 6.2144 | 6.365 | 6.1667 | 6.1596 | 6.1988 | 6.3707 | 6.3977 | 6.1742 | 6.1729 | 6.1725 |
| neg_1889 | Arachidonic acid | metab_6505 | HMDB0001043;PW_C000821;LMFA01030001 | C00219 | 303.2325 | 6.4951 | neg | M-H | C20H32O2 | 0 | 43.8 | -1.4903389 | 506-32-1 | 0.0155418 | 5.2826 | 5.1952 | 5.2242 | 5.2562 | 5.3476 | 5.3961 | 5.3558 | 5.2465 | 5.242 | 5.4491 | 5.3304 | 5.2479 | 5.226 | 5.2381 | 5.2374 |
| neg_1891 | DG(2:0/20:3(6,8,11)-OH(5)/0:0) | metab_6508 | HMDB0297051 | - | 483.2957 | 6.503 | neg | M+FA-H | C25H42O6 | 0 | 38.4 | -1.5381328 | - | 0.0054275 | 5.4158 | 5.2856 | 5.218 | 5.2731 | 5.5661 | 5.6311 | 5.4519 | 5.439 | 5.4441 | 5.6828 | 5.5489 | 5.249 | 5.4416 | 5.4444 | 5.4397 |
| neg_1898 | Linoleic Acid | metab_6515 | HMDB0006270;HMDB0000673;LMFA01030120;PW_C000530;HMDB0247706;MJDBOTE0000548 | C01595 | 279.2325 | 6.5193 | neg | M-H | C18H32O2 | 72 | 0 | -1.5806316 | 506-21-8;60-33-3 | 0.00477606 | 5.2226 | 5.3465 | 5.3823 | 5.2615 | 5.4267 | 5.5255 | 5.3086 | 5.2413 | 5.438 | 5.3685 | 5.3875 | 5.3111 | 5.3936 | 5.3956 | 5.3977 |
| neg_1910 | MEDICA 16 | metab_6530 | HMDB0254402 | - | 341.2691 | 6.5585 | neg | M-H | C20H38O4 | 85.5 | 0 | -1.7954405 | - | 0.01041607 | 5.6633 | 5.729 | 5.7285 | 5.6747 | 5.4485 | 5.3871 | 5.7572 | 5.4672 | 5.5609 | 5.5263 | 5.5591 | 5.5189 | 5.5512 | 5.5594 | 5.5587 |
| neg_1913 | 3-hydroxypentadecanoic acid | metab_6533 | HMDB0061657 | - | 257.2118 | 6.5663 | neg | M-H | C15H30O3 | 0 | 44.5 | -1.6266347 | - | 0.01757557 | 5.126 | 5.0682 | 4.9777 | 5.2189 | 4.8638 | 4.9783 | 5.083 | 4.9518 | 4.9876 | 5.3314 | 4.9341 | 4.9399 | 4.9578 | 4.9599 | 4.9719 |
| neg_1915 | PC(16:1(9E)/0:0) | metab_6535 | HMDB0256155;LMGP01050021 | - | 492.309 | 6.5663 | neg | M-H | C24H48NO7P | 0 | 65.2 | -1.0667498 | - | 0.00540312 | 4.9738 | 5.4059 | 5.3008 | 5.3182 | 5.1422 | 5.2276 | 5.2534 | 5.1224 | 5.2491 | 5.2157 | 4.944 | 5.0065 | 5.2491 | 5.246 | 5.2506 |
| neg_1916 | 4-(undecan-5-yl)benzene-1-Sulfonic Acid | metab_6536 | - | - | 311.1683 | 6.5741 | neg | M-H | C17H28O3S | 75.3 | 0 | -1.13801 | - | 0.01008329 | 5.9558 | 5.9571 | 5.9127 | 6.0343 | 5.956 | 6.0203 | 6.121 | 5.8519 | 5.9387 | 6.1212 | 6.0558 | 6.1207 | 6.187 | 6.1948 | 6.1943 |
| neg_1918 | Gymnodimine | metab_6538 | HMDB0041430 | C20025 | 552.3302 | 6.5741 | neg | M+FA-H | C32H45NO4 | 0 | 59.4 | -5.6613184 | 173792-58-0 | 0.00460159 | 5.7101 | 6.0355 | 5.9533 | 5.9223 | 5.8518 | 5.8505 | 5.9786 | 5.8189 | 5.8749 | 5.86 | 5.7861 | 5.8249 | 5.9027 | 5.9047 | 5.9007 |
| neg_1920 | PE-NMe(20:2(11Z,14Z)/18:0) | metab_6541 | PW_C059466;HMDB0113342 | C01241 | 830.5912 | 6.5741 | neg | M+FA-H | C44H84NO8P | 0 | 89.2 | -0.5793341 | - | 0.06982559 | 6.6504 | 5.7133 | 6.7588 | 6.7161 | 6.6485 | 6.7253 | 6.6727 | 6.7248 | 6.7188 | 6.8291 | 6.6979 | 6.7659 | 6.7679 | 6.7172 | 6.7155 |
| neg_1925 | LysoPA(0:0/18:2(9Z,12Z)) | metab_6546 | HMDB0007852 | - | 433.2354 | 6.582 | neg | M-H | C21H39O7P | 0 | 51.2 | -1.5206087 | - | 0.00901606 | 5.512 | 5.567 | 5.6085 | 5.3912 | 5.5971 | 5.6555 | 5.437 | 5.2811 | 5.7122 | 5.3669 | 5.4525 | 5.5605 | 5.5284 | 5.5304 | 5.5359 |
| neg_1927 | 15-Hexadecanolide | metab_6548 | HMDB0031711 | - | 299.2223 | 6.5898 | neg | M+FA-H | C16H30O2 | 0 | 47.1 | -1.8340869 | 69297-56-9 | 0.01959672 | 4.844 | 5.1794 | 5.0131 | 5.1417 | 4.3706 | 4.7118 | 4.9079 | 4.6384 | 4.4241 | 5.0689 | 4.9878 | 4.4849 | 4.857 | 4.8721 | 4.8716 |
| neg_1937 | (R)-Sulcatol | metab_6558 | LMFA05000566;HMDB0030030 | C07288 | 255.2324 | 6.6139 | neg | 2M-H | C8H16O | 0 | 32.9 | -2.016905 | 6/2/30 | 0.01178394 | 5.4952 | 5.3892 | 5.395 | 5.4727 | 5.419 | 5.4751 | 5.3983 | 5.4255 | 5.4293 | 5.4524 | 5.4085 | 5.3177 | 5.4917 | 5.4937 | 5.5014 |
| neg_1942 | Trans-16-Octadecenoic acid | metab_6564 | HMDB0304769;LMFA01030081 | - | 281.2481 | 6.6296 | neg | M-H | C18H34O2 | 0 | 94.1 | -1.7293159 | - | 0.01216081 | 5.2922 | 5.3662 | 5.4453 | 5.4151 | 5.398 | 5.4578 | 5.367 | 5.3945 | 5.4252 | 5.4999 | 5.3437 | 5.2694 | 5.4219 | 5.4242 | 5.432 |
| neg_1953 | DG(8:0/i-13:0/0:0) | metab_6576 | PW_C064418;HMDB0092909 | C00165 | 395.316 | 6.6531 | neg | M-H2O-H | C24H46O5 | 0 | 51.1 | -1.6910069 | - | 0.02712656 | 5.0905 | 5.2868 | 5.0493 | 4.9772 | 5.0819 | 4.8082 | 5.0193 | 4.5266 | 4.9488 | 4.939 | 4.9976 | 4.9451 | 5.0967 | 5.0769 | 5.0982 |
| neg_1955 | 13,14-Dihydro-15-keto-PGE2 | metab_6578 | LMFA03010031;HMDB0002776 | C04671 | 351.2206 | 6.6609 | neg | M-H | C20H32O5 | 77.2 | 0 | 8.13399088 | 363-23-5 | 0.00824165 | 6.2981 | 6.4835 | 6.4565 | 6.4098 | 6.1292 | 6.5256 | 6.3331 | 6.1969 | 6.3131 | 6.3922 | 6.4154 | 6.4069 | 6.3339 | 6.3286 | 6.3355 |
| neg_196 | 4-Carboxynevirapine | metab_6583 | PW_C040609;HMDB0060759 | - | 333.04 | 3.8367 | neg | M+Na-2H, M+K-2H | C15H12N4O3 | 0 | 56.8 | 1.70252971 | - | 0.01744036 | 5.1044 | 5.2706 | 5.1708 | 5.211 | 5.1351 | 5.1427 | 5.3155 | 5.1884 | 5.0724 | 5.1115 | 5.1422 | 5.1408 | 5.1783 | 5.1918 | 5.1912 |
| neg_1963 | DG(10:0/12:0/0:0) | metab_6587 | HMDB0092963;PW_C043342 | C00165 | 409.3318 | 6.6922 | neg | M-H2O-H | C25H48O5 | 0 | 33.6 | -1.3281093 | - | 0.02660219 | 4.3324 | 4.7652 | 4.5933 | 4.5386 | 4.5912 | 3.3941 | 4.5095 | 4.3021 | 4.5077 | 4.2969 | 4.413 | 4.4524 | 4.6337 | 4.6349 | 4.6142 |
| neg_1965 | Palmitaldehyde | metab_6589 | HMDB0001551;PW_C001193;LMFA06000088 | C00517 | 285.2431 | 6.7078 | neg | M+FA-H | C16H32O | 0 | 47.8 | -1.6675423 | 629-80-1 | 0.01312239 | 5.3813 | 5.3026 | 5.1599 | 5.3817 | 5.0117 | 4.9088 | 5.3071 | 5.1435 | 4.9696 | 5.2409 | 5.0502 | 5.0074 | 5.1278 | 5.138 | 5.1375 |
| neg_1970 | Prostaglandin D1 Alcohol | metab_6593 | - | - | 339.2534 | 6.7313 | neg | M-H | C20H36O4 | 54.9 | 0 | -2.0936448 | - | 0.0228569 | 3.7688 | 4.2703 | 3.6605 | 3.7357 | 3.9339 | 3.7659 | 3.792 | 3.5282 | 3.8501 | 3.6189 | 3.5575 | 3.5534 | 4.2678 | 4.2697 | 4.2858 |
| neg_1972 | LysoPE(20:1(11Z)/0:0) | metab_6595 | HMDB0011512 | - | 552.3301 | 6.747 | neg | M+FA-H | C25H50NO7P | 0 | 80.2 | -1.116373 | - | 0.04244101 | 4.5319 | 5.1006 | 4.8976 | 4.775 | 4.7891 | 4.8212 | 4.9111 | 4.5917 | 4.8957 | 4.8733 | 4.56 | 4.5895 | 4.9358 | 4.9043 | 4.9042 |
| neg_1975 | (Z)-6-Nonen-1-ol | metab_6598 | HMDB0040581 | - | 283.2638 | 6.7548 | neg | 2M-H | C9H18O | 0 | 56.1 | -1.7162148 | 35854-86-5 | 0.00939329 | 4.7599 | 4.584 | 4.7397 | 4.8185 | 4.8768 | 4.949 | 4.7849 | 4.7971 | 4.8499 | 5.0104 | 4.9429 | 4.561 | 4.9484 | 4.9558 | 4.9552 |
| neg_198 | Milbemycin A3 | metab_6603 | HMDB0254721;HMDB0254720;HMDB0242623;HMDB0254730 | - | 527.2969 | 6.1803 | neg | M-H, M+Na-2H | C31H44O7 | 0 | 61.3 | -8.6165498 | - | 0.016987 | 4.0621 | 6.2958 | 6.0779 | 5.65 | 6.5527 | 6.5875 | 5.4437 | 5.6123 | 6.5579 | 5.9258 | 6.4828 | 6.1782 | 6.3192 | 6.3068 | 6.3062 |
| neg_1981 | (+/-)-Menthyl acetate | metab_6604 | HMDB0041264 | C09870 | 441.3214 | 6.7946 | neg | 2M+FA-H | C12H22O2 | 0 | 45.7 | -1.8292312 | 89-48-5 | 0.00461649 | 4.9879 | 5.519 | 5.2467 | 5.2578 | 5.1877 | 5.3825 | 5.3903 | 4.9618 | 5.3991 | 5.1138 | 5.2205 | 5.1686 | 5.545 | 5.547 | 5.543 |
| neg_1985 | AVOCADENE ACETATE | metab_6608 | LMFA05000715 | - | 327.2535 | 6.8102 | neg | M-H | C19H36O4 | 56.7 | 0 | -1.7006099 | - | 0.03530301 | 4.7066 | 4.7699 | 4.66 | 5.0542 | 3.9255 | 4.0892 | 4.8892 | 4.6599 | 4.3367 | 4.9031 | 4.295 | 4.226 | 4.802 | 4.829 | 4.8036 |
| neg_1986 | Chalepin acetate | metab_6609 | HMDB0030666 | C09308 | 355.1576 | 6.8102 | neg | M-H | C21H24O5 | 0 | 31.6 | 7.10543489 | 14882-94-1 | 0.01677233 | 5.523 | 5.1523 | 5.1316 | 5.5221 | 4.637 | 4.6061 | 5.3498 | 5.252 | 4.6928 | 5.4046 | 4.6558 | 4.8552 | 5.3431 | 5.3533 | 5.3573 |
| neg_1987 | 12-HETE | metab_6610 | HMDB0062287;HMDB0006111;HMDB0244455;LMFA03060063;LMFA03060064;LMFA03060088 | C14777 | 365.236 | 6.8102 | neg | M+FA-H | C20H32O3 | 0 | 54.6 | 8.22142914 | 54397-83-0 | 0.00548968 | 5.8226 | 6.0741 | 5.7627 | 5.9553 | 5.5335 | 5.7955 | 5.9316 | 5.6571 | 5.6414 | 5.9084 | 5.8526 | 5.6681 | 5.9095 | 5.9063 | 5.911 |
| neg_1988 | N-Oleoyl Histidine | metab_6611 | HMDB0241960;LMFA08020133 | - | 464.3138 | 6.8102 | neg | M+FA-H | C24H41N3O3 | 0 | 32.1 | 1.80064138 | - | 0.01286884 | 5.7917 | 5.4478 | 5.6777 | 5.7247 | 5.528 | 5.5348 | 5.7268 | 5.7036 | 5.4515 | 5.7203 | 5.3425 | 5.5823 | 5.7946 | 5.7857 | 5.7961 |
| neg_1997 | Capric acid | metab_6621 | LMFA01010010;HMDB0000511;PW_C000391 | C01571 | 209.094 | 6.818 | neg | M+K-2H | C10H20O2 | 0 | 48.7 | -5.1870159 | 334-48-5 | 0.00463727 | 5.3676 | 5.3321 | 5.2967 | 5.4813 | 5.1818 | 5.2043 | 5.4088 | 5.3883 | 5.174 | 5.4731 | 5.2141 | 5.2341 | 5.4042 | 5.4018 | 5.4058 |
| neg_1998 | N-oleoyl taurine | metab_6622 | LMFA08020081;HMDB0242113 | - | 388.2522 | 6.818 | neg | M-H | C20H39NO4S | 41.1 | 0 | -1.3462873 | - | 0.00767297 | 6.0302 | 5.0211 | 5.6756 | 5.9673 | 5.5646 | 5.4495 | 5.8827 | 5.7689 | 4.9693 | 5.9217 | 5.5656 | 5.4446 | 5.5933 | 5.5885 | 5.5949 |
| neg_2000 | LysoPC(P-16:0/0:0) | metab_6627 | HMDB0010407 | - | 524.335 | 6.8337 | neg | M+FA-H | C24H50NO6P | 0 | 81.3 | -1.7252133 | - | 0.0067323 | 5.2913 | 5.3421 | 5.4019 | 5.3715 | 5.3512 | 5.4783 | 5.4197 | 5.4094 | 5.2833 | 5.3228 | 5.3913 | 5.5287 | 5.5317 | 5.5338 | 5.5281 |
| neg_2051 | Demethylphylloquinone | metab_6681 | HMDB0004649 | C13309 | 473.2818 | 7.1365 | neg | M+K-2H | C30H44O2 | 0 | 72.2 | -2.2410072 | 34625-86-0 | 0.02112939 | 5.8985 | 5.8873 | 5.9158 | 5.9835 | 5.7543 | 5.8568 | 5.9303 | 5.84 | 5.8218 | 6.0166 | 5.8603 | 5.8904 | 5.9962 | 5.9807 | 5.9802 |
| neg_212 | 3-(4-Hydroxyphenyl)lactate | metab_6757 | HMDB0000755 | C03672 | 181.0497 | 3.9134 | neg | M-H2O-H, M-H | C9H10O4 | 56.2 | 0 | -5.2308887 | 306-23-0 | 0.00544732 | 5.4614 | 5.433 | 5.4296 | 5.5296 | 5.4492 | 5.4534 | 5.4379 | 5.4098 | 5.5128 | 5.2899 | 5.6107 | 5.743 | 5.6436 | 5.648 | 5.6475 |
| neg_2133 | 1-Hydroxy-2,5-dioxopyrrolidine-3-sulfonic acid | metab_6772 | HMDB0243897 | - | 175.9677 | 7.5649 | neg | M-H2O-H | C4H5NO6S | 0 | 43.3 | 9.13700549 | - | 0.00774752 | 5.046 | 5.026 | 5.0694 | 5.0681 | 5.0742 | 5.0824 | 5.0277 | 5.0287 | 5.0645 | 5.1218 | 5.0234 | 5.0335 | 5.0664 | 5.0609 | 5.0604 |
| neg_218 | L-Tryptophan | metab_6823 | PW_C000741;HMDB0000929;MJDBOTE0000426 | C00078 | 203.0817 | 3.8367 | neg | M-H, 2M-H | C11H12N2O2 | 75.9 | 0 | -4.2318816 | 73-22-3 | 0.00055999 | 6.1833 | 6.4946 | 6.3615 | 6.374 | 6.2624 | 6.2281 | 6.4879 | 6.269 | 6.1225 | 6.1958 | 6.208 | 6.2766 | 6.3217 | 6.3217 | 6.3213 |
| neg_223 | Glutamylleucine | metab_6879 | HMDB0028823 | - | 259.1295 | 4.136 | neg | M-H, M+Na-2H | C11H20N2O5 | 0 | 34.8 | -1.6199595 | 5969-52-8 | 0.01071901 | 5.5103 | 5.6524 | 5.5709 | 5.3264 | 5.4678 | 5.2605 | 5.5315 | 5.387 | 5.2269 | 5.3672 | 5.2587 | 5.4473 | 5.4309 | 5.4396 | 5.4326 |
| neg_226 | 6-Hydroxy-5-methoxyindole glucuronide | metab_6912 | HMDB0010362 | - | 338.0877 | 4.2183 | neg | M-H, M+Na-2H | C15H17NO8 | 0 | 65.6 | -1.2119151 | 77463-72-0 | 0.01797177 | 5.5367 | 5.0362 | 5.925 | 5.949 | 4.9748 | 5.1034 | 5.8739 | 6.1419 | 4.8713 | 5.5246 | 5.5491 | 5.6967 | 5.3718 | 5.386 | 5.3734 |
| neg_228 | Indole-3-carboxilic acid-O-sulphate | metab_6934 | HMDB0060002 | - | 239.9966 | 4.2361 | neg | M-H, M+Na-2H | C9H7NO5S | 0 | 58.9 | -2.4793846 | - | 0.01624261 | 3.9028 | 4.3707 | 4.242 | 3.7574 | 5.7888 | 5.8202 | 4.0621 | 3.9127 | 5.7602 | 3.3193 | 5.7325 | 5.8706 | 5.7521 | 5.7646 | 5.7642 |
| neg_232 | 3-Indole carboxylic acid glucuronide | metab_6979 | HMDB0013189 | - | 336.072 | 4.3363 | neg | M-H, M+Na-2H | C15H15NO8 | 0 | 70.8 | -1.4698766 | - | 0.00316358 | 4.9106 | 5.217 | 5.4415 | 5.0398 | 6.5053 | 6.5356 | 5.2591 | 4.9961 | 6.4424 | 4.4936 | 6.5291 | 6.594 | 5.9607 | 5.9635 | 5.9623 |
| neg_236 | Ketoleucine | metab_7023 | PW_C000547;HMDB0000695 | C00233 | 129.0545 | 4.3736 | neg | M-H, M+FA-H | C6H10O3 | 67.6 | 0 | -9.6647973 | 816-66-0 | 0.0030208 | 6.6406 | 6.4696 | 6.577 | 6.5127 | 6.4439 | 6.497 | 6.6607 | 6.4194 | 6.3184 | 6.3789 | 6.4529 | 6.3666 | 6.377 | 6.379 | 6.3765 |
| neg_24 | Ribitol | metab_7066 | HMDB0000508 | C00474 | 197.0658 | 0.8958 | neg | M-H2O-H, M-H, M+FA-H | C5H12O5 | 57.3 | 0 | -5.5551691 | 488-81-3 | 0.0097931 | 6.9069 | 6.8843 | 6.8771 | 6.8569 | 6.8643 | 6.9107 | 6.9964 | 6.8596 | 6.9027 | 6.982 | 6.8996 | 6.9549 | 6.855 | 6.857 | 6.8631 |
| neg_241 | Pimelic Acid | metab_7078 | HMDB0000857;LMFA01170051 | C02656 | 159.0652 | 4.5555 | neg | M-H, M+Na-2H | C7H12O4 | 41.3 | 0 | -6.775525 | 111-16-0 | 0.01297111 | 5.2848 | 5.5012 | 5.286 | 5.2522 | 5.4809 | 5.4738 | 5.3848 | 5.1966 | 5.534 | 5.2354 | 5.6298 | 5.5947 | 5.4703 | 5.4797 | 5.4804 |
| neg_2414 | LysoPE(0:0/22:0) | metab_7083 | HMDB0011490 | - | 582.3771 | 7.5336 | neg | M+FA-H | C27H56NO7P | 0 | 85.4 | -0.9989574 | - | 0.02389085 | 5.1432 | 5.2155 | 5.3014 | 5.3036 | 5.1094 | 5.2118 | 5.2683 | 5.1743 | 5.2071 | 5.3198 | 5.2609 | 5.2152 | 5.154 | 5.1364 | 5.1358 |
| neg_243 | 5-Hydroxy-6-methoxyindole glucuronide | metab_7100 | HMDB0010363 | - | 338.0877 | 4.61 | neg | M-H, M+Na-2H | C15H17NO8 | 0 | 57.8 | -1.213985 | 77463-71-9 | 0.014125 | 4.787 | 5.2685 | 5.0959 | 4.8449 | 5.8531 | 5.8066 | 5.254 | 4.8353 | 5.7129 | 4.4825 | 6.0106 | 6.0541 | 5.4897 | 5.4992 | 5.4878 |
| neg_244 | P-coumaroyltriacetic acid lactone | metab_7111 | HMDB0304447 | C12087 | 308.0084 | 4.3285 | neg | M+Na-2H, M+K-2H | C15H11O5- | 0 | 66.5 | -3.1807954 | - | 0.01186882 | 5.7881 | 5.9379 | 5.8657 | 5.8091 | 5.8694 | 5.9442 | 5.8718 | 5.7916 | 5.8802 | 5.7332 | 5.9087 | 5.8916 | 5.8743 | 5.8839 | 5.8759 |
| neg_248 | 2-Pyrocatechuic Acid | metab_7155 | HMDB0000397;MJDBOTE0000462 | C00196 | 153.0182 | 4.6535 | neg | M-H2O-H, M-H | C7H6O4 | 95.7 | 0 | -7.3121263 | 303-38-8 | 0.00886931 | 5.0006 | 5.3889 | 5.0745 | 5.1221 | 5.6357 | 5.5223 | 4.9765 | 4.856 | 5.5793 | 4.9716 | 5.5478 | 5.7134 | 5.7618 | 5.7687 | 5.7682 |
| neg_2497 | PE-NMe(18:0/22:4(7Z,10Z,13Z,16Z)) | metab_7174 | HMDB0113104;PW_C059227 | C01241 | 854.591 | 7.1365 | neg | M+FA-H | C46H84NO8P | 0 | 90.1 | -0.860517 | - | 0.0359541 | 4.8027 | 6.6375 | 6.9111 | 6.449 | 6.4838 | 6.6763 | 6.6333 | 6.5855 | 6.6498 | 6.7778 | 6.5694 | 6.7105 | 6.6231 | 6.5941 | 6.5992 |
| neg_2522 | Citreoviridin | metab_7203 | HMDB0250305 | C16766 | 383.1889 | 6.9877 | neg | M-H2O-H | C23H30O6 | 0 | 55.9 | 6.31455302 | 25425-12-1 | 0.03485068 | 5.8366 | 5.489 | 5.4267 | 5.866 | 4.703 | 4.7192 | 5.6669 | 5.3635 | 5.0147 | 5.8124 | 4.6645 | 5.0676 | 5.5248 | 5.5524 | 5.5284 |
| neg_2530 | 3-Oxo-12,18-ursadien-28-oic acid | metab_7212 | HMDB0037065 | - | 489.2767 | 6.9798 | neg | M+K-2H | C30H44O3 | 0 | 65.8 | -1.9959739 | 273223-69-1 | 0.01358436 | 5.5686 | 5.519 | 5.4578 | 5.5575 | 5.0596 | 5.135 | 5.443 | 5.3507 | 5.1336 | 5.6621 | 5.0246 | 4.7157 | 5.3085 | 5.2986 | 5.2982 |
| neg_2550 | Neuromedin N (1-4) | metab_7231 | HMDB0013021 | - | 485.2819 | 6.8884 | neg | M-H2O-H | C26H40N4O6 | 0 | 57.8 | 9.87309084 | - | 0.00245081 | 6.3657 | 6.4539 | 6.356 | 6.522 | 6.0968 | 6.1501 | 6.4181 | 6.4239 | 6.1153 | 6.5576 | 6.145 | 6.2575 | 6.9272 | 6.9257 | 6.9252 |
| neg_2553 | Thromboxane | metab_7234 | HMDB0003208;LMFA03030015;HMDB0001452 | C02198 | 341.3055 | 6.8727 | neg | M+FA-H | C20H40O | 0 | 36.5 | -1.9717632 | 57576-52-0;66719-58-2 | 0.00935458 | 3.3417 | 3.728 | 3.7573 | 4.2357 | 3.6334 | 3.6913 | 3.3903 | 3.3617 | 3.3757 | 3.4062 | 3.391 | 3.387 | 5.0671 | 5.0689 | 5.0748 |
| neg_2555 | Avenestergenin A1 | metab_7236 | HMDB0035264 | - | 618.3769 | 6.8493 | neg | M-H2O-H | C38H55NO7 | 0 | 79.8 | -4.8727088 | 90578-35-1 | 0.18824841 | 4.4724 | 5.1725 | 4.7217 | 5.2309 | 5.4426 | 5.3088 | 5.1097 | 4.9118 | 5.186 | 4.4506 | 5.0787 | 5.2741 | 4.7307 | 4.7314 | 4.5798 |
| neg_2556 | Pristanal | metab_7237 | LMPR0104010012;HMDB0001958;PW_C001309 | - | 327.29 | 6.8337 | neg | M+FA-H | C19H38O | 0 | 37.9 | -1.7420839 | 105373-75-9 | 0.00532283 | 5.4452 | 5.6454 | 5.5326 | 5.7618 | 5.3103 | 5.2228 | 5.5869 | 5.3245 | 5.3639 | 5.6419 | 5.4364 | 5.3725 | 5.7565 | 5.7572 | 5.7608 |
| neg_2557 | PE-NMe(18:0/20:3(8Z,11Z,14Z)) | metab_7238 | PW_C059220;HMDB0113097 | C01241 | 828.5755 | 6.818 | neg | M+FA-H | C44H82NO8P | 0 | 75.7 | -0.6741152 | - | 0.03204664 | 6.1857 | 6.4535 | 6.2433 | 6.402 | 6.4106 | 6.4588 | 6.4363 | 6.5257 | 6.438 | 6.5318 | 6.4586 | 6.4276 | 6.7062 | 6.7052 | 6.6814 |
| neg_2558 | Tetracosanedioic acid | metab_7239 | HMDB0302983;LMFA01170039 | - | 397.3316 | 6.818 | neg | M-H | C24H46O4 | 0 | 67.5 | -1.9554974 | - | 0.03405864 | 5.4691 | 5.735 | 5.5605 | 5.7133 | 5.3878 | 5.183 | 5.6364 | 5.2702 | 5.4826 | 5.4263 | 5.2581 | 5.2538 | 5.5492 | 5.5236 | 5.5498 |
| neg_2559 | Rubschisandrin | metab_7240 | HMDB0257360 | C10621 | 381.1733 | 6.818 | neg | M-H2O-H | C23H28O6 | 0 | 36.6 | 6.36818409 | 82467-51-4 | 0.02097118 | 5.8595 | 5.6234 | 5.5673 | 6.0395 | 4.8581 | 4.7911 | 5.7282 | 5.6749 | 4.8301 | 5.8277 | 4.7837 | 5.1846 | 5.729 | 5.732 | 5.7459 |
| neg_2561 | Terephthalic Acid | metab_7243 | HMDB0002428;MJDBOTE0000641 | C06337 | 165.0182 | 6.818 | neg | M-H | C8H6O4 | 81.8 | 0 | -6.7841905 | 100-21-0 | 0.01500687 | 5.298 | 5.1738 | 5.1834 | 5.319 | 4.9189 | 4.9509 | 5.3515 | 5.2186 | 4.9906 | 5.2803 | 4.9657 | 5.0318 | 5.2258 | 5.2305 | 5.2387 |
| neg_2574 | NORETHINDRONE ACETATE | metab_7257 | HMDB0255731 | C08152 | 339.1993 | 6.8102 | neg | M-H | C22H28O3 | 32.2 | 0 | 8.00563148 | 51-98-9 | 0.01803394 | 6.3413 | 6.2061 | 6.179 | 6.473 | 6.0554 | 6.1023 | 6.3618 | 6.1757 | 6.0647 | 6.4222 | 6.0851 | 6.2107 | 6.4274 | 6.4295 | 6.4418 |
| neg_2578 | 2-Hydroxystearic acid | metab_7261 | HMDB0062549;HMDB0242145 | C03045 | 299.2586 | 6.8024 | neg | M-H | C18H36O3 | 0 | 59.8 | -1.836751 | 26531-80-6 | 0.00217024 | 6.0588 | 5.9758 | 5.9789 | 6.2549 | 5.7036 | 5.6879 | 6.0795 | 5.9351 | 5.6859 | 5.918 | 5.7125 | 5.777 | 5.9288 | 5.9287 | 5.9304 |
| neg_2590 | Erucic acid | metab_7275 | HMDB0002068 | C08316 | 383.3162 | 6.747 | neg | M+FA-H | C22H42O2 | 0 | 72.1 | -1.5485326 | 112-86-7 | 0.01774844 | 4.6934 | 5.077 | 4.8787 | 4.8272 | 4.7227 | 4.8032 | 5.1527 | 4.5028 | 4.9746 | 4.5623 | 4.6855 | 4.6259 | 4.8527 | 4.8681 | 4.8612 |
| neg_2594 | (2R,3S)-2-Octyl-3-pentyloxane | metab_7279 | HMDB0242702 | - | 313.2744 | 6.7156 | neg | M+FA-H | C18H36O | 0 | 30.1 | -1.6444255 | - | 0.01390158 | 4.7164 | 4.6224 | 4.5894 | 5.2302 | 4.546 | 4.5751 | 4.2312 | 3.8901 | 4.3466 | 4.6105 | 4.3897 | 4.596 | 5.3584 | 5.3485 | 5.3595 |
| neg_2599 | Muricatacin | metab_7283 | HMDB0038685;LMFA05000682 | - | 283.2274 | 6.7078 | neg | M-H | C17H32O3 | 0 | 56.7 | -1.4884583 | 134698-86-5 | 0.00394983 | 4.5607 | 4.9968 | 4.5553 | 4.8442 | 3.6628 | 4.1602 | 4.8458 | 4.3124 | 4.356 | 5.0927 | 4.5077 | 4.3171 | 4.9799 | 4.9772 | 4.9768 |
| neg_2607 | Docosanedioic acid | metab_7293 | HMDB0242127;HMDB0304342;LMFA01170037 | C19625 | 369.3004 | 6.6844 | neg | M-H | C22H42O4 | 0 | 83.6 | -1.7861296 | - | 0.00586726 | 5.6484 | 5.8722 | 5.8311 | 5.7458 | 5.6448 | 5.6244 | 5.7949 | 5.5275 | 5.816 | 5.6109 | 5.6403 | 5.5719 | 5.5819 | 5.5777 | 5.5773 |
| neg_2608 | Dodecylbenzenesulfonic Acid | metab_7294 | HMDB0059915;HMDB0031031 | - | 325.1838 | 6.6844 | neg | M-H | C18H30O3S | 86.3 | 0 | -1.4806896 | - | 0.00594088 | 5.959 | 5.8722 | 5.8676 | 5.9828 | 5.9115 | 5.9527 | 6.0566 | 5.8215 | 5.9012 | 6.0855 | 5.9681 | 6.0466 | 6.1443 | 6.1464 | 6.1494 |
| neg_2610 | LysoPC(15:0/0:0) | metab_7297 | HMDB0010381;HMDB0010375 | C04230 | 480.3088 | 6.6765 | neg | M-H | C23H48NO7P | 0 | 72 | -1.552642 | - | 0.00461474 | 6.9282 | 6.7895 | 6.7971 | 6.8225 | 6.7705 | 6.7978 | 6.8223 | 6.8187 | 6.806 | 6.7927 | 6.7899 | 6.7789 | 6.7699 | 6.7717 | 6.7739 |
| neg_2618 | 16-Methylheptadecanoic acid | metab_7305 | HMDB0031066 | C20356 | 321.2195 | 6.6531 | neg | M+K-2H | C18H36O2 | 0 | 52.9 | -2.3434396 | 2724-58-5 | 0.00201132 | 5.098 | 5.0788 | 5.1598 | 5.2546 | 5.1424 | 5.103 | 5.1906 | 5.1245 | 5.1614 | 5.2386 | 5.08 | 5.2366 | 5.8091 | 5.8093 | 5.8107 |
| neg_2619 | 8,11-eicosadiynoic acid | metab_7306 | LMFA01030688 | - | 303.2325 | 6.6531 | neg | M-H | C20H32O2 | 54.6 | 0 | -1.4649253 | - | 0.01160191 | 5.367 | 5.1085 | 5.199 | 5.2844 | 5.2288 | 5.1403 | 5.304 | 5.1346 | 5.0569 | 5.2399 | 5.0905 | 5.3179 | 5.2145 | 5.2067 | 5.2161 |
| neg_2627 | 8-Oxohexadecanoic acid | metab_7313 | HMDB0030972 | - | 269.2119 | 6.6452 | neg | M-H | C16H30O3 | 0 | 36.3 | -1.2188193 | 2777-52-8 | 0.00291019 | 5.0149 | 5.2954 | 5.0594 | 5.2704 | 4.302 | 4.9113 | 5.1964 | 4.8574 | 4.7602 | 5.448 | 5.0564 | 4.7243 | 5.2786 | 5.279 | 5.281 |
| neg_2628 | LysoPE(18:0/0:0) | metab_7314 | HMDB0011130 | - | 462.299 | 6.6374 | neg | M-H2O-H | C23H48NO7P | 0 | 57.3 | 0.08823127 | 69747-55-3 | 0.02829511 | 5.4129 | 4.9653 | 5.3072 | 5.2823 | 5.3092 | 5.033 | 5.3438 | 5.1509 | 5.206 | 4.8362 | 4.6981 | 5.644 | 5.3437 | 5.3458 | 5.3233 |
| neg_2629 | Momordol | metab_7315 | HMDB0029804 | - | 439.3425 | 6.6374 | neg | M-H | C26H48O5 | 0 | 60.5 | -1.014685 | 189156-42-1 | 0.00068463 | 5.5451 | 5.5673 | 5.5887 | 5.5389 | 5.5513 | 5.252 | 5.6518 | 5.5057 | 5.491 | 5.5063 | 5.4106 | 5.5586 | 5.765 | 5.7656 | 5.7651 |
| neg_2631 | LysoPE(P-16:0/0:0) | metab_7318 | HMDB0011152 | - | 436.283 | 6.6296 | neg | M-H | C21H44NO6P | 0 | 41.2 | -0.8596713 | 174062-72-7 | 0.03553607 | 5.3603 | 5.0836 | 5.3637 | 5.329 | 5.2279 | 5.2014 | 5.3551 | 5.294 | 5.1771 | 5.2219 | 5.0154 | 5.3097 | 5.3479 | 5.3307 | 5.3171 |
| neg_2636 | PE-NMe(22:2(13Z,16Z)/16:1(9Z)) | metab_7323 | PW_C059696;HMDB0113567 | C01241 | 828.5752 | 6.6139 | neg | M+FA-H | C44H82NO8P | 0 | 81.2 | -1.0669614 | - | 0.17427443 | 5.4527 | 6.0848 | 5.8391 | 6.0306 | 6.021 | 6.01 | 6.2306 | 6.2139 | 6.0815 | 6.1166 | 6.1928 | 6.0618 | 5.6509 | 5.5012 | 5.5657 |
| neg_2637 | 11Z-Eicosenoic acid | metab_7324 | LMFA01030085;HMDB0002231 | C16526 | 355.2849 | 6.6139 | neg | M+FA-H | C20H38O2 | 0 | 73.6 | -1.680934 | 5561-99-9 | 0.00114246 | 4.9692 | 5.1731 | 5.0304 | 4.9726 | 4.8876 | 5.0187 | 5.1814 | 4.7673 | 4.9954 | 4.7802 | 4.9578 | 4.9135 | 5.0001 | 5.001 | 5.001 |
| neg_2654 | 11-Oxahexadecanolide | metab_7342 | HMDB0303921 | - | 255.1961 | 6.582 | neg | M-H | C15H28O3 | 0 | 51.5 | -1.654785 | - | 0.01352159 | 3.8228 | 4.055 | 4.2633 | 4.1914 | 3.0223 | 3.873 | 5.0235 | 3.5191 | 3.9205 | 4.3967 | 3.4029 | 3.9627 | 4.9834 | 4.9938 | 4.9933 |
| neg_2660 | 5alpha-Cyprinol | metab_7349 | HMDB0247015 | C16890 | 451.3423 | 6.5741 | neg | M-H | C27H48O5 | 0 | 31 | -1.2453549 | 2952-70-7 | 0.00995044 | 4.9693 | 5.0236 | 5.0051 | 5.0307 | 4.9324 | 4.7165 | 5.0262 | 4.8449 | 4.9015 | 4.7772 | 4.8754 | 4.9443 | 5.06 | 5.0601 | 5.0675 |
| neg_2665 | 5,7-Megastigmadien-9-ol glucoside | metab_7354 | HMDB0041044 | - | 337.2051 | 6.5663 | neg | M-H2O-H | C19H32O6 | 0 | 53.9 | 8.58518868 | 146610-77-7 | 0.01166945 | 5.6852 | 5.9461 | 5.9564 | 5.6806 | 5.5475 | 5.9867 | 5.7617 | 5.6432 | 5.7927 | 5.8132 | 5.9279 | 5.8041 | 5.8012 | 5.8111 | 5.8079 |
| neg_2678 | 1-arachidonoyl-2-hydroxy-sn-glycero-3-phosphate | metab_7368 | - | - | 457.2355 | 6.5428 | neg | M-H | C23H39O7P | 68.9 | 0 | -1.1909546 | - | 0.01526515 | 5.4924 | 5.4011 | 5.3505 | 5.2507 | 5.5183 | 5.4925 | 5.4057 | 5.2513 | 5.4863 | 5.342 | 5.4079 | 5.5023 | 5.2667 | 5.256 | 5.2682 |
| neg_2687 | 2-Dodecoxyethyl Hydrogen Sulfate | metab_7378 | - | - | 309.1737 | 6.5271 | neg | M-H | C14H30O5S | 35.2 | 0 | -1.4526673 | - | 0.01319015 | 4.673 | 4.7394 | 4.7656 | 4.7942 | 4.9367 | 4.7561 | 4.6872 | 4.6783 | 4.6365 | 4.7584 | 4.7369 | 4.8234 | 4.8538 | 4.8642 | 4.8549 |
| neg_2694 | PC(17:2(9Z,12Z)/0:0) | metab_7386 | HMDB0256157;LMGP01050127 | - | 504.3091 | 6.5114 | neg | M-H | C25H48NO7P | 0 | 81 | -0.8692231 | - | 0.0022419 | 6.4974 | 6.5628 | 6.5682 | 6.4929 | 6.5907 | 6.5861 | 6.5549 | 6.4495 | 6.5618 | 6.4779 | 6.5255 | 6.5872 | 6.5429 | 6.5427 | 6.5445 |
| neg_2697 | 2-Hydroxymyristic Acid | metab_7388 | HMDB0002261;LMFA01050484 | - | 243.196 | 6.5114 | neg | M-H | C14H28O3 | 70.6 | 0 | -2.1653084 | 2507-55-3 | 0.01314006 | 5.2449 | 5.1931 | 5.2824 | 5.5452 | 5.0029 | 5.0908 | 5.2814 | 5.2942 | 5.0712 | 5.5347 | 5.0754 | 4.9981 | 5.1038 | 5.0942 | 5.0938 |
| neg_27 | Benzenebutanoic acid, alpha-(acetylamino)-2-amino-gamma-oxo- | metab_7391 | HMDB0245586 | - | 271.0697 | 3.8367 | neg | M+Na-2H, M+K-2H, M-H | C12H14N2O4 | 0 | 49.4 | -1.387502 | - | 0.01603672 | 5.5857 | 5.7729 | 5.7454 | 5.7006 | 5.6183 | 5.6159 | 5.8048 | 5.6699 | 5.522 | 5.5995 | 5.578 | 5.6439 | 5.6808 | 5.6829 | 5.6938 |
| neg_2701 | PE(20:3/0:0) | metab_7393 | - | - | 502.2936 | 6.4951 | neg | M-H | C25H46NO7P | 83.5 | 0 | -0.6846548 | - | 0.02380905 | 4.1265 | 4.8833 | 4.9108 | 4.771 | 4.5594 | 4.7865 | 4.6141 | 4.7084 | 4.8924 | 4.7301 | 4.5507 | 4.2031 | 4.9977 | 5.0159 | 5.0155 |
| neg_2707 | Dihydrotestosterone | metab_7398 | HMDB0002961;PW_C001745;HMDB0243561;MJDBOTE0001000 | C03917 | 335.2216 | 6.4873 | neg | M+FA-H | C19H30O2 | 0 | 75.6 | -4.1005245 | 521-18-6 | 0.01309017 | 5.3535 | 5.0719 | 5.1167 | 5.3941 | 4.6971 | 4.4475 | 5.3763 | 5.0835 | 4.8228 | 5.4343 | 4.8079 | 4.8195 | 5.5753 | 5.5663 | 5.5768 |
| neg_2709 | Gossypol | metab_7399 | LMPR0103330002;HMDB0040723 | C07667 | 517.186 | 6.4795 | neg | M-H | C30H30O8 | 83.5 | 0 | -1.4693157 | 303-45-7;90141-22-3 | 0.02102367 | 5.6711 | 4.9328 | 5.0637 | 5.1816 | 5.1132 | 4.929 | 4.6314 | 5.3539 | 5.4043 | 5.2408 | 4.9505 | 5.0819 | 5.2495 | 5.2344 | 5.2332 |
| neg_2719 | Supinine | metab_7410 | HMDB0302362 | C10403 | 264.1602 | 6.4716 | neg | M-H2O-H | C15H25NO4 | 0 | 40.9 | -1.0725129 | 551-58-6 | 0.01392292 | 4.4269 | 4.4579 | 4.4834 | 4.5543 | 4.3195 | 4.531 | 4.432 | 4.5181 | 4.4355 | 4.5485 | 4.5246 | 4.4504 | 4.5004 | 4.4907 | 4.5018 |
| neg_2722 | Ganodermic acid P2 | metab_7414 | HMDB0035292 | - | 605.3205 | 6.4638 | neg | M+Cl | C34H50O7 | 0 | 83 | -7.9632185 | 112430-69-0 | 0.00845759 | 6.0985 | 5.9598 | 5.8755 | 5.9366 | 6.0522 | 6.0093 | 6.0339 | 5.9398 | 5.916 | 5.9994 | 5.9939 | 6.0389 | 5.8753 | 5.8819 | 5.8815 |
| neg_2725 | Norethindrone oxime | metab_7417 | HMDB0255732 | - | 358.2019 | 6.4638 | neg | M+FA-H | C20H27NO2 | 0 | 70.7 | -1.4165803 | - | 0.00863544 | 4.0949 | 4.7502 | 4.4718 | 4.3269 | 4.5529 | 4.6535 | 4.6342 | 4.2161 | 4.9427 | 4.4348 | 4.7028 | 4.7555 | 5.2036 | 5.198 | 5.2051 |
| neg_2726 | 12(S)-HETrE | metab_7418 | HMDB0062747 | - | 321.2429 | 6.4638 | neg | M-H | C20H34O3 | 0 | 84.8 | -1.7873375 | - | 0.00193247 | 5.4069 | 5.3971 | 5.1917 | 5.6728 | 4.926 | 5.2624 | 5.4233 | 5.576 | 5.1256 | 5.1627 | 4.5146 | 5.4022 | 5.2736 | 5.2724 | 5.272 |
| neg_2727 | 4-(2,6,6-Trimethyl-1-cyclohexen-1-yl)-2-butanone | metab_7419 | HMDB0032913 | C03527 | 239.1647 | 6.4638 | neg | M+FA-H | C13H22O | 0 | 41.4 | -3.0032606 | 17283-81-7 | 0.01169693 | 5.3701 | 5.0873 | 5.3238 | 5.4764 | 4.2965 | 4.7752 | 5.0957 | 5.2748 | 4.4337 | 5.439 | 4.6256 | 4.6428 | 5.0107 | 5.0027 | 5.0122 |
| neg_2728 | Lucidenic acid M | metab_7420 | HMDB0035973 | C10029 | 443.2803 | 6.456 | neg | M-H2O-H | C27H42O6 | 0 | 53.9 | -0.0761356 | 110241-33-3 | 0.0320724 | 4.104 | 4.178 | 4.3554 | 3.8479 | 4.0189 | 4.0526 | 4.2619 | 4.1252 | 4.2914 | 3.9804 | 4.0768 | 4.2718 | 4.3913 | 4.3943 | 4.3686 |
| neg_2734 | Dodecyl Hydrogen Sulfate | metab_7425 | - | - | 265.1476 | 6.4481 | neg | M-H | C12H26O4S | 86.6 | 0 | -1.0341036 | - | 0.00988048 | 5.6319 | 5.5221 | 5.4465 | 5.5508 | 5.5338 | 5.5689 | 5.4974 | 5.4352 | 5.4406 | 5.5371 | 5.4552 | 5.486 | 5.5732 | 5.5667 | 5.5748 |
| neg_2744 | Andrographolide | metab_7434 | HMDB0248395;MJDBOTE0001465 | C20214 | 387.1557 | 6.4324 | neg | M+K-2H | C20H30O5 | 0 | 63.4 | -6.2830525 | 5508-58-7 | 0.01404581 | 4.8968 | 4.9413 | 4.9496 | 4.9423 | 4.9381 | 4.9904 | 4.9394 | 4.9097 | 4.8967 | 5.0362 | 4.9166 | 4.9383 | 4.961 | 4.9513 | 4.9626 |
| neg_2753 | SM(d18:2(4E,14Z)/PGF2alpha) | metab_7444 | HMDB0290571 | - | 843.5499 | 6.4162 | neg | M+FA-H | C43H79N2O9P | 0 | 87.9 | -0.7368436 | - | 0.05753609 | 5.3363 | 5.7396 | 5.7236 | 5.7991 | 5.3698 | 5.7267 | 5.7881 | 5.7632 | 5.6518 | 5.857 | 5.7145 | 5.7088 | 5.7284 | 5.6808 | 5.6932 |
| neg_2755 | P-Menthane-3,8-diol | metab_7446 | HMDB0036145;HMDB0035595;LMPR0102090063 | C02904 | 343.2848 | 6.4162 | neg | 2M-H | C10H20O2 | 0 | 57.5 | -1.6392123 | 42822-86-6;91739-72-9;565-50-4 | 0.01401304 | 5.2146 | 5.5715 | 5.5475 | 5.5951 | 5.2631 | 5.27 | 5.6462 | 5.1638 | 5.2826 | 5.2899 | 5.49 | 5.3875 | 5.5459 | 5.5356 | 5.5352 |
| neg_2769 | PE(22:1(13Z)/15:0) | metab_7460 | HMDB0009516;PW_C005564 | C00350 | 804.5757 | 6.3927 | neg | M+FA-H | C42H82NO8P | 0 | 93.3 | -0.384992 | - | 0.06961246 | 6.0645 | 6.0929 | 6.6601 | 6.7255 | 6.5731 | 6.7057 | 6.7757 | 6.5405 | 6.5571 | 6.7528 | 6.7832 | 6.6368 | 6.3883 | 6.334 | 6.3402 |
| neg_2771 | Chenodeoxycholic Acid | metab_7463 | LMST04010032;HMDB0000518;PW_C000397;MJDBOTE0001132 | C02528 | 437.2902 | 6.3927 | neg | M+FA-H | C24H40O4 | 80.7 | 0 | -1.6401734 | 474-25-9 | 0.0293941 | 4.9482 | 5.1269 | 5.1345 | 4.8141 | 5.1525 | 5.2807 | 5.3198 | 5.1036 | 5.3461 | 4.715 | 5.0336 | 5.4014 | 5.0792 | 5.1017 | 5.0804 |
| neg_2779 | 2-hydroxyhexadecanoic acid | metab_7469 | HMDB0031057 | - | 271.2276 | 6.3849 | neg | M-H | C16H32O3 | 38.5 | 0 | -1.0379274 | 16452-52-1 | 0.03360007 | 5.3441 | 5.3604 | 5.1541 | 5.3773 | 4.5829 | 4.7377 | 5.4559 | 5.317 | 4.6581 | 5.3511 | 4.534 | 4.841 | 5.1315 | 5.1332 | 5.1069 |
| neg_2782 | 9,10-Epoxyoctadecenoic acid | metab_7473 | HMDB0004701 | C14825 | 341.2327 | 6.377 | neg | M+FA-H | C18H32O3 | 0 | 63.2 | -2.2276257 | - | 0.02866617 | 3.5772 | 3.8469 | 3.7332 | 3.7936 | 3.6962 | 3.1035 | 3.9005 | 3.3308 | 3.1986 | 3.1087 | 3.6905 | 3.1828 | 4.4386 | 4.4171 | 4.439 |
| neg_2785 | Beta-Ionone | metab_7476 | HMDB0036565 | C12287 | 237.1491 | 6.377 | neg | M+FA-H | C13H20O | 0 | 52.3 | -2.8583563 | 79-77-6 | 0.05254146 | 4.812 | 4.4837 | 4.8327 | 5.0001 | 3.953 | 4.4218 | 4.5295 | 4.8112 | 4.1148 | 4.9579 | 4.22 | 4.305 | 4.5 | 4.5024 | 4.4611 |
| neg_2786 | PE-NMe2(16:0/18:2(9Z,12Z)) | metab_7477 | PW_C059959;HMDB0113943 | C04308 | 788.5442 | 6.3692 | neg | M+FA-H | C41H78NO8P | 0 | 95.2 | -0.6592826 | - | 0.05923997 | 5.2761 | 5.7966 | 5.3176 | 5.8399 | 5.5186 | 5.3443 | 5.8548 | 5.9291 | 5.4575 | 5.3863 | 5.9435 | 5.4858 | 5.5819 | 5.5402 | 5.5361 |
| neg_2787 | LysoPC(20:5(5Z,8Z,11Z,14Z,17Z)/0:0) | metab_7478 | HMDB0010397 | C04230 | 586.3145 | 6.3692 | neg | M+FA-H | C28H48NO7P | 0 | 84.8 | -1.0439839 | - | 0.03684751 | 5.5105 | 5.7357 | 5.5033 | 5.4457 | 5.6795 | 5.661 | 5.6597 | 5.5103 | 5.5549 | 5.4949 | 5.3428 | 5.615 | 5.5796 | 5.5817 | 5.5527 |
| neg_2788 | (R)-pantoate | metab_7479 | HMDB0303992;HMDB0240389 | C00522 | 440.1928 | 6.3692 | neg | 3M-H | C6H11O4- | 0 | 46.5 | 6.49968421 | 1112-33-0;470-29-1 | 0.04080552 | 3.2482 | 4.1621 | 3.6995 | 3.4108 | 3.3812 | 3.3074 | 3.6046 | 3.6155 | 3.6736 | 3.3127 | 3.5152 | 4.0408 | 4.8162 | 4.8215 | 4.7882 |
| neg_2793 | Trans-and cis-2,4,8-Trimethyl-3,7-nona-dien-2-ol | metab_7485 | - | - | 227.1646 | 6.3692 | neg | M+FA-H | C12H22O | 0 | 40.7 | -3.4747425 | - | 0.15343008 | 4.2804 | 3.8972 | 4.2061 | 4.3935 | 2.9435 | 3.0242 | 3.7067 | 4.1031 | 3.2616 | 4.7043 | 3.668 | 3.2658 | 3.829 | 3.853 | 3.7221 |
| neg_2799 | PE-NMe(22:5(7Z,10Z,13Z,16Z,19Z)/18:0) | metab_7488 | PW_C059784;HMDB0113651 | C01241 | 852.5755 | 6.3536 | neg | M+FA-H | C46H82NO8P | 0 | 89.8 | -0.6144707 | - | 0.22416874 | 5.8937 | 6.575 | 6.7308 | 6.6757 | 6.5376 | 6.6385 | 6.7159 | 6.6694 | 6.618 | 6.7878 | 6.5689 | 6.6531 | 6.7418 | 6.5608 | 6.7457 |
| neg_2804 | 4-Octylphenol | metab_7495 | HMDB0246557 | C14132 | 251.1648 | 6.3536 | neg | M+FA-H | C14H22O | 0 | 48.6 | -2.369489 | 1806-26-4 | 0.01002046 | 3.9252 | 4.5177 | 4.3807 | 4.6074 | 4.2712 | 4.1615 | 4.541 | 4.3018 | 4.5808 | 4.5265 | 4.372 | 4.4972 | 5.0866 | 5.0887 | 5.0803 |
| neg_2807 | 5-Kete | metab_7498 | HMDB0246835;HMDB0010217;PW_C006229 | C14732 | 317.2117 | 6.3457 | neg | M-H | C20H30O3 | 40.2 | 0 | -1.5580584 | 126432-17-5 | 0.00603354 | 5.0526 | 4.7796 | 4.3239 | 5.0767 | 4.5437 | 4.7593 | 5.0697 | 5.0232 | 4.5531 | 4.6634 | 4.2671 | 4.8827 | 4.8093 | 4.8086 | 4.8044 |
| neg_2817 | 3-Dehydroteasterone | metab_7508 | LMST01030131;HMDB0041527 | C15792 | 491.3374 | 6.3373 | neg | M+FA-H | C28H46O4 | 0 | 91.3 | -0.9390327 | 124853-28-7 | 0.0349442 | 4.9338 | 5.2036 | 4.9474 | 5.0193 | 5.1886 | 4.8806 | 5.1667 | 4.6603 | 5.1923 | 4.6257 | 5.0409 | 4.7801 | 4.6447 | 4.6469 | 4.6718 |
| neg_2831 | Prostaglandin F3a | metab_7520 | HMDB0002122 | C06476 | 333.2059 | 6.3295 | neg | M-H2O-H | C20H32O5 | 0 | 75.1 | -3.3788826 | 745-64-2 | 0.00777025 | 5.6572 | 5.5971 | 5.623 | 5.8606 | 5.4399 | 5.2248 | 5.9753 | 5.5046 | 5.4625 | 5.8382 | 5.4198 | 5.4712 | 5.5786 | 5.5807 | 5.5741 |
| neg_2838 | 3-Methylhexanoylcarnitine | metab_7527 | HMDB0241038 | - | 254.1758 | 6.3216 | neg | M-H2O-H | C14H27NO4 | 0 | 34.8 | -1.4970405 | - | 0.01534005 | 4.5846 | 3.3561 | 4.6546 | 5.1997 | 3.3612 | 3.5732 | 4.4862 | 4.8562 | 3.3617 | 5.1578 | 3.377 | 3.5873 | 4.4402 | 4.4296 | 4.4419 |
| neg_2842 | Lithocholic acid glucuronide | metab_7531 | HMDB0254126;HMDB0002513 | C03033 | 551.322 | 6.3138 | neg | M-H | C30H48O9 | 0 | 30.9 | -0.9464726 | 75239-91-7 | 0.02158191 | 5.5555 | 5.3668 | 5.3014 | 5.2215 | 5.1115 | 5.1015 | 5.1348 | 5.2558 | 5.4197 | 5.0304 | 5.2068 | 5.5046 | 4.9241 | 4.9266 | 4.9091 |
| neg_2844 | Avocadene 4-acetate | metab_7533 | HMDB0031045;LMFA05000642 | - | 373.259 | 6.3138 | neg | M+FA-H | C19H36O4 | 0 | 76.3 | -1.6983047 | 59499-18-2 | 0.03613531 | 4.6723 | 5.2706 | 5.0951 | 5.0959 | 5.136 | 4.9551 | 5.2541 | 4.3926 | 5.0559 | 4.9298 | 5.0296 | 5.044 | 5.2651 | 5.2382 | 5.2382 |
| neg_2856 | 2-[3-(sulfooxy)phenyl]acetic acid | metab_7545 | HMDB0125163 | - | 277.0036 | 6.2981 | neg | M+FA-H | C8H8O6S | 0 | 47.5 | 5.13345711 | - | 0.00566438 | 3.7022 | 4.284 | 3.5245 | 3.9455 | 4.3955 | 4.5142 | 4.0183 | 3.8759 | 4.7364 | 3.9791 | 4.3366 | 4.3038 | 5.1793 | 5.1814 | 5.1842 |
| neg_2858 | (2E,4E)-Hexa-2,4-dienedioylcarnitine | metab_7547 | HMDB0241673 | - | 266.1031 | 6.2981 | neg | M-H2O-H | C13H19NO6 | 0 | 31.4 | -1.1299273 | - | 0.00582294 | 4.9444 | 4.9647 | 5.0031 | 4.9514 | 4.8986 | 4.9053 | 4.9565 | 4.9444 | 4.8795 | 5.0134 | 4.9206 | 4.9647 | 4.932 | 4.9278 | 4.9274 |
| neg_2863 | Deoxycholylglycine | metab_7552 | HMDB0000631;HMDB0242414;LMST05030006 | C05464 | 448.3062 | 6.2903 | neg | M-H | C26H43NO5 | 0 | 77.3 | -1.4024038 | 360-65-6 | 0.0017954 | 5.7863 | 6.7651 | 6.9205 | 6.5911 | 7.2255 | 7.1814 | 6.1993 | 6.8732 | 7.0986 | 6.5273 | 7.0322 | 7.0694 | 6.8857 | 6.8863 | 6.8872 |
| neg_2864 | Henagliflozin | metab_7553 | HMDB0253086 | - | 435.1015 | 6.2903 | neg | M-H2O-H | C22H24ClFO7 | 0 | 43.5 | -0.3159537 | - | 0.01611772 | 3.4084 | 4.5123 | 3.4499 | 3.4592 | 3.4419 | 3.4676 | 3.4571 | 3.5747 | 4.8909 | 5.4873 | 3.5661 | 3.9561 | 4.8123 | 4.8008 | 4.7997 |
| neg_2867 | P,p'-DDA | metab_7555 | HMDB0249252 | C06640 | 279.0006 | 6.2903 | neg | M-H | C14H10Cl2O2 | 67.4 | 0 | 7.39555051 | - | 0.02081371 | 3.0888 | 3.5634 | 3.1634 | 3.9078 | 4.0269 | 4.2136 | 3.6742 | 3.1089 | 4.4638 | 3.7435 | 4.1723 | 4.069 | 4.9926 | 4.9773 | 4.9768 |
| neg_2879 | 1-(4-O-beta-D-glucopyranosyl-3-methoxyphenyl)-3,5-dihydroxydecane | metab_7568 | HMDB0303085 | - | 475.267 | 6.2749 | neg | M+Na-2H | C25H42O7 | 0 | 56.5 | -1.5696359 | - | 0.00577487 | 4.9608 | 5.7407 | 5.712 | 5.256 | 5.6851 | 5.9256 | 5.8313 | 5.3856 | 5.9312 | 5.1871 | 5.6367 | 5.6866 | 6.0182 | 6.0218 | 6.0169 |
| neg_2880 | Ecdysone | metab_7570 | HMDB0251684;LMST01010210 | C00477 | 445.2953 | 6.2749 | neg | M-H2O-H | C27H44O6 | 0 | 45.9 | -1.3172184 | 3604-87-3 | 0.00536431 | 4.8385 | 4.6699 | 4.7404 | 4.0161 | 4.9816 | 4.8749 | 4.4604 | 4.3866 | 4.8316 | 4.4863 | 4.6939 | 4.8511 | 4.8557 | 4.8603 | 4.8574 |
| neg_2884 | 13,14-Dihydro PGF-1a | metab_7573 | HMDB0005076 | - | 357.2642 | 6.2749 | neg | M-H | C20H38O5 | 0 | 39 | -1.3687999 | - | 0.01590217 | 4.9027 | 5.311 | 5.2616 | 5.2586 | 5.2142 | 5.2186 | 5.3518 | 4.8846 | 5.2812 | 4.9579 | 5.2807 | 5.261 | 5.4867 | 5.4753 | 5.4743 |
| neg_2902 | Atractylenolide III | metab_7593 | HMDB0248708;MJDBOTE0001286 | C17887 | 283.11 | 6.2586 | neg | M+Cl | C15H20O3 | 0 | 44.3 | -2.4131496 | - | 0.03533579 | 4.8055 | 4.7761 | 4.7599 | 4.7994 | 4.7286 | 4.8714 | 4.6531 | 4.7087 | 4.7992 | 4.9328 | 4.8392 | 4.9053 | 4.6019 | 4.629 | 4.6285 |
| neg_2909 | Ethenzamide | metab_7599 | HMDB0245110 | - | 210.0764 | 6.2508 | neg | M+FA-H | C9H11NO2 | 0 | 33.7 | -4.4469452 | - | 0.00916588 | 4.6312 | 4.6558 | 4.6296 | 4.6612 | 4.6334 | 4.6565 | 4.7325 | 4.6931 | 4.6537 | 4.7512 | 4.6493 | 4.6658 | 4.6452 | 4.6528 | 4.6468 |
| neg_2911 | Dioscoretine | metab_7602 | HMDB0038588 | - | 240.1601 | 6.2508 | neg | M-H | C13H23NO3 | 0 | 42.2 | -1.7672829 | 128637-87-6 | 0.02813475 | 5.3275 | 4.7521 | 5.4874 | 5.5921 | 3.885 | 4.4851 | 5.1589 | 5.4667 | 4.2556 | 5.7417 | 4.2526 | 4.5739 | 4.8449 | 4.8469 | 4.8668 |
| neg_2921 | 9-Oxo-nonanoic acid | metab_7612 | HMDB0094711;LMFA01060160 | C16322 | 343.2121 | 6.2429 | neg | 2M-H | C9H16O3 | 0 | 53.8 | -1.4993121 | - | 0.00332282 | 4.8725 | 5.0538 | 4.9525 | 4.7916 | 5.0439 | 4.9239 | 5.0176 | 4.8304 | 4.9813 | 4.9055 | 4.9182 | 4.9784 | 5.1288 | 5.1317 | 5.1304 |
| neg_2923 | Ipomeatetrahydrofuran | metab_7614 | HMDB0040904 | - | 301.2016 | 6.2429 | neg | M+FA-H | C15H28O3 | 0 | 56.6 | -1.8777363 | 92448-62-9 | 0.01143199 | 4.9083 | 4.8515 | 4.8891 | 4.9762 | 4.855 | 4.6845 | 5.1632 | 4.8471 | 4.9714 | 5.1323 | 4.8876 | 4.8305 | 4.8805 | 4.8728 | 4.8821 |
| neg_2926 | 4-Hydroxydebrisoquine | metab_7617 | HMDB0006468 | - | 236.1048 | 6.2429 | neg | M+FA-H | C10H13N3O | 0 | 58.7 | 3.79186381 | 59333-79-8 | 0.01227074 | 5.0837 | 5.0136 | 5.0884 | 5.082 | 5.0398 | 5.1223 | 5.1086 | 5.1192 | 5.0343 | 5.1779 | 5.0631 | 5.0826 | 5.024 | 5.0339 | 5.0256 |
| neg_2927 | Procarbazine | metab_7618 | HMDB0015299 | C07402 | 220.1462 | 6.2429 | neg | M-H | C12H19N3O | 0 | 35.3 | 2.9152624 | 671-16-9 | 0.01841291 | 4.5172 | 4.4121 | 4.4855 | 4.4838 | 4.4502 | 4.5263 | 4.5072 | 4.53 | 4.4793 | 4.5763 | 4.4733 | 4.4924 | 4.4826 | 4.4695 | 4.4842 |
| neg_2928 | 4-Methylumbelliferyl acetate | metab_7619 | HMDB0032989 | C03837 | 217.0499 | 6.2429 | neg | M-H | C12H10O4 | 0 | 66.9 | -3.2575374 | 5/9/47 | 0.02640514 | 2.791 | 3.0997 | 2.8325 | 2.921 | 3.9359 | 3.8614 | 2.8397 | 3.032 | 3.8952 | 2.8555 | 3.634 | 3.7626 | 4.0829 | 4.1033 | 4.0843 |
| neg_2938 | 2,4,5,7alpha-Tetrahydro-1,4,4,7a-tetramethyl-1H-inden-2-ol | metab_7630 | HMDB0036684 | - | 237.1493 | 6.2351 | neg | M+FA-H | C13H20O | 0 | 44.2 | -1.4472208 | 99901-23-2 | 0.00884058 | 4.8583 | 4.8107 | 4.8868 | 4.8725 | 4.8484 | 4.8618 | 4.9129 | 4.9149 | 4.8486 | 4.9464 | 4.86 | 4.8894 | 4.8483 | 4.8557 | 4.85 |
| neg_2961 | Metoprolol | metab_7655 | HMDB0001932;PW_C001291;MJDBOTE0001765 | C07202 | 312.1812 | 6.2195 | neg | M+FA-H | C15H25NO3 | 0 | 36.9 | -1.5124897 | 37350-58-6;51384-51-1 | 0.02955113 | 6.0822 | 4.8599 | 5.3153 | 5.5813 | 4.5171 | 4.499 | 4.7686 | 5.68 | 4.9517 | 6.0024 | 4.7456 | 4.9997 | 5.4773 | 5.4553 | 5.4553 |
| neg_2978 | 5-Nonyltetrahydro-2-oxo-3-furancarboxylic acid | metab_7671 | HMDB0030993 | - | 255.1598 | 6.2116 | neg | M-H | C14H24O4 | 0 | 54.3 | -1.5444309 | - | 0.01372894 | 5.0717 | 4.9827 | 5.0139 | 5.0863 | 5.1047 | 4.8778 | 5.0725 | 4.8829 | 5.0184 | 5.2268 | 4.975 | 5.1048 | 5.043 | 5.0539 | 5.0445 |
| neg_2986 | 1,11-Undecanedicarboxylic acid | metab_7679 | HMDB0002327 | - | 243.1597 | 6.2038 | neg | M-H | C13H24O4 | 74.4 | 0 | -2.0459633 | 505-52-2 | 0.00937955 | 5.1994 | 4.9535 | 4.9792 | 5.4269 | 5.0374 | 4.8826 | 5.1134 | 4.897 | 5.0024 | 5.0789 | 5.0095 | 5.095 | 5.0748 | 5.0821 | 5.0816 |
| neg_2992 | (1R,2R,4R,5S)-(+)-p-Menthane-2,5-diol | metab_7686 | HMDB0301947 | - | 173.1173 | 6.1959 | neg | M-H | C9H18O3 | 0 | 32.7 | -5.6266149 | - | 0.00675361 | 4.7358 | 4.6816 | 4.7507 | 4.7991 | 4.7455 | 4.7592 | 4.7612 | 4.7534 | 4.8006 | 4.8339 | 4.7334 | 4.8041 | 4.7809 | 4.7829 | 4.7771 |
| neg_3004 | Lithocholic acid glycine conjugate | metab_7701 | LMST05030009;HMDB0000698;PW_C000550 | C15557 | 478.3164 | 6.1803 | neg | M+FA-H | C26H43NO4 | 0 | 47.5 | -2.3059049 | 474-74-8 | 0.01773788 | 4.4808 | 4.7338 | 4.7496 | 4.544 | 5.4336 | 4.9077 | 4.6196 | 4.7702 | 5.6481 | 4.7535 | 4.9151 | 5.4813 | 5.1367 | 5.1237 | 5.1232 |
| neg_3013 | 4-Heptyloxyphenol | metab_7711 | HMDB0246434 | C14236 | 253.1441 | 6.1803 | neg | M+FA-H | C13H20O2 | 0 | 48 | -1.9285606 | 13037-86-0 | 0.0057381 | 5.2154 | 5.2148 | 5.2192 | 5.2226 | 5.2038 | 5.2363 | 5.2642 | 5.2145 | 5.2397 | 5.3081 | 5.2065 | 5.2191 | 5.2152 | 5.2173 | 5.2124 |
| neg_3014 | Atractylodin | metab_7712 | HMDB0248710 | C16918 | 227.071 | 6.1803 | neg | M+FA-H | C13H10O | 0 | 40.9 | -2.0516817 | 55290-63-6 | 0.01093818 | 3.5183 | 3.9031 | 4.8864 | 3.6066 | 5.0019 | 4.9135 | 3.8298 | 3.0467 | 4.8686 | 3.4951 | 4.7632 | 4.8878 | 4.3514 | 4.3599 | 4.3594 |
| neg_3023 | Probenecid | metab_7722 | HMDB0015166 | C07372 | 284.0959 | 6.1725 | neg | M-H | C13H19NO4S | 70.3 | 0 | -1.0299921 | 57-66-9 | 0.00890344 | 4.4688 | 4.8336 | 4.7955 | 4.621 | 4.2975 | 3.6969 | 4.9107 | 4.6394 | 4.1743 | 4.454 | 4.3545 | 5.0275 | 4.2847 | 4.2917 | 4.2911 |
| neg_3024 | 4'-(Nitrosomethylamino)-1-(3-pyridyl)-1-butanone | metab_7723 | HMDB0246622;HMDB0011603 | C16453 | 252.1 | 6.1725 | neg | M+FA-H | C10H13N3O2 | 0 | 45.4 | 4.76711856 | 64091-91-4 | 0.01573353 | 4.7914 | 4.8663 | 4.8562 | 4.8397 | 4.8906 | 4.9075 | 4.8643 | 4.8815 | 4.8783 | 4.9889 | 4.8784 | 4.8854 | 4.8628 | 4.8763 | 4.8684 |
| neg_3025 | Exo,exo-1,8-Epoxy-p-menthane-2,6-diol | metab_7724 | HMDB0041376 | C17620 | 185.1174 | 6.1725 | neg | M-H | C10H18O3 | 0 | 46.2 | -4.838862 | 38223-98-2 | 0.00397724 | 4.8381 | 5.1181 | 5.0289 | 5.0964 | 5.2699 | 5.081 | 5.2514 | 4.8951 | 5.332 | 4.9336 | 5.2345 | 5.4362 | 5.1534 | 5.1566 | 5.1561 |
| neg_3030 | Aceteugenol | metab_7729 | HMDB0034122 | C14567 | 251.0927 | 6.1646 | neg | M+FA-H | C12H14O3 | 0 | 44.8 | 0.84127994 | 93-28-7 | 0.0108106 | 4.5865 | 4.6885 | 4.6957 | 4.6761 | 4.6251 | 4.6962 | 4.6782 | 4.6836 | 4.7 | 4.6952 | 4.6271 | 4.6472 | 4.6779 | 4.6864 | 4.6858 |
| neg_3046 | Pregnenolone sulfate | metab_7745 | HMDB0060382;HMDB0000774;LMST05020014 | C18044 | 395.1893 | 6.149 | neg | M-H | C21H32O5S | 72.2 | 0 | -1.1761012 | 1247-64-9 | 0.00449857 | 5.5377 | 5.6609 | 5.7281 | 5.7278 | 5.4677 | 5.4253 | 5.5257 | 5.5753 | 5.5355 | 5.5637 | 5.4683 | 5.67 | 5.3031 | 5.3051 | 5.307 |
| neg_3047 | 11b-PGF2a | metab_7746 | HMDB0010199 | C05959 | 353.2329 | 6.149 | neg | M-H | C20H34O5 | 0 | 65.8 | -1.3955655 | - | 0.01253216 | 5.29 | 5.0237 | 4.9435 | 5.1736 | 4.9804 | 4.8301 | 5.1434 | 5.1905 | 4.9376 | 4.7782 | 4.6328 | 5.3177 | 5.0907 | 5.1004 | 5.1 |
| neg_3055 | Ichangin 4-glucoside | metab_7755 | HMDB0039340 | - | 631.2403 | 6.1411 | neg | M-H2O-H | C32H42O14 | 0 | 62.3 | 1.11876978 | - | 0.04106743 | 4.8103 | 4.2451 | 5.0632 | 4.9235 | 3.6113 | 4.0678 | 4.8763 | 5.115 | 4.3381 | 4.9725 | 4.0586 | 4.4269 | 5.0354 | 5.0669 | 5.0664 |
| neg_3061 | 3,4-Methylenedioxymethamphetamine | metab_7762 | HMDB0041931;HMDB0254382 | C07577 | 238.108 | 6.1411 | neg | M+FA-H | C11H15NO2 | 0 | 33 | -2.5605886 | 42542-10-9;4764-17-4 | 0.05370016 | 4.1445 | 4.527 | 4.3151 | 4.4282 | 4.1358 | 4.315 | 4.4505 | 4.4471 | 4.3552 | 4.0452 | 4.4505 | 4.4682 | 3.9656 | 4.0058 | 4.0074 |
| neg_3067 | Corticosterone-21-hemisuccinate | metab_7768 | HMDB0250465 | - | 491.2282 | 6.1333 | neg | M+FA-H | C25H34O7 | 0 | 52.5 | -0.9995754 | - | 0.01282186 | 3.4454 | 4.8421 | 5.4017 | 4.8145 | 5.1326 | 4.9727 | 5.3861 | 4.4148 | 4.9871 | 4.1897 | 5.381 | 5.3638 | 4.7512 | 4.7615 | 4.7527 |
| neg_3069 | 3'-Hydroxystanozolol | metab_7770 | HMDB0006001 | - | 381.1947 | 6.1333 | neg | M+K-2H | C21H32N2O2 | 0 | 32.3 | -0.9583796 | 125709-39-9 | 0.01700757 | 4.7793 | 5.2241 | 5.0641 | 5.0835 | 4.7792 | 4.7522 | 5.3094 | 4.4256 | 4.6285 | 5.2571 | 4.9635 | 4.6765 | 4.7609 | 4.7744 | 4.7731 |
| neg_307 | PS(15:0/22:0) | metab_7771 | PW_C060732;LMGP03010156;HMDB0112334 | C02737 | 826.5599 | 7.5336 | neg | M-H, M+Na-2H | C43H84NO10P | 0 | 35.8 | 2.46616532 | - | 0.15154175 | 5.94 | 6.1267 | 6.031 | 5.6288 | 5.9164 | 6.2688 | 6.0865 | 6.105 | 6.1712 | 6.3167 | 6.2625 | 6.2215 | 4.5453 | 4.4357 | 4.4353 |
| neg_3071 | (S)-10,16-Dihydroxyhexadecanoic acid | metab_7772 | HMDB0037798 | C08285 | 287.2224 | 6.1333 | neg | M-H | C16H32O4 | 0 | 70.8 | -1.255013 | 69232-67-3 | 0.01011472 | 4.198 | 4.3591 | 4.2121 | 4.1637 | 3.9429 | 3.9087 | 4.5914 | 4.444 | 4.033 | 4.1423 | 3.9964 | 4.2614 | 5.1053 | 5.1119 | 5.1037 |
| neg_3084 | 8-Hydroxy-4(6)-lactarene-5,14-diol | metab_7786 | HMDB0035780 | - | 299.186 | 6.1255 | neg | M+FA-H | C15H26O3 | 0 | 56.5 | -1.5549282 | - | 0.02911562 | 4.4625 | 4.5602 | 4.395 | 4.4428 | 4.5549 | 4.3697 | 4.7128 | 4.3871 | 4.517 | 4.5939 | 4.584 | 4.555 | 4.6376 | 4.6567 | 4.633 |
| neg_3088 | 2-Amino-4-oxo-6-(1',2'-dioxoprolyl)-7,8-dihydroxypteridine | metab_7790 | HMDB0001410 | C05255 | 593.1296 | 6.1177 | neg | 2M+Hac-H | C9H9N5O5 | 0 | 72.7 | -9.3082874 | - | 0.00512107 | 5.1801 | 5.1581 | 5.204 | 5.2233 | 5.0972 | 5.2137 | 5.1887 | 5.2301 | 5.2083 | 5.2498 | 5.2969 | 5.236 | 5.1977 | 5.1949 | 5.1993 |
| neg_309 | PS(15:0/24:1(15Z)) | metab_7792 | PW_C060740;HMDB0112341 | C02737 | 852.5752 | 7.5179 | neg | M-H, M+Na-2H | C45H86NO10P | 0 | 32.3 | 1.9089908 | - | 0.29651076 | 5.3942 | 5.4888 | 5.4298 | 5.3957 | 5.5373 | 5.7651 | 5.637 | 5.6313 | 5.6816 | 5.7036 | 5.6576 | 5.734 | 4.4185 | 4.2043 | 4.2141 |
| neg_3095 | 4-Oxo-4-(3-Oxodecan-2-Ylamino)Butanoic Acid | metab_7797 | - | - | 270.1709 | 6.1177 | neg | M-H | C14H25NO4 | 52.1 | 0 | -0.8427721 | - | 0.02539128 | 3.0906 | 3.396 | 3.399 | 3.3889 | 3.653 | 2.8758 | 3.7755 | 2.8367 | 2.8507 | 3.8264 | 3.1636 | 3.2812 | 4.2158 | 4.1971 | 4.1965 |
| neg_31 | 3-Phenyllactic Acid | metab_7801 | HMDB0000779;MJDBOTE0000652 | C01479 | 165.0547 | 5.0437 | neg | M+Cl, M+FA-H, M-H | C9H10O3 | 30.4 | 0 | -6.4213415 | 828-01-3 | 0.01056378 | 6.3703 | 6.5374 | 6.5066 | 6.4601 | 6.3397 | 6.2853 | 6.0452 | 6.105 | 6.7365 | 6.2705 | 6.5776 | 6.3387 | 6.147 | 6.1556 | 6.1486 |
| neg_3102 | 4-ene-Valproic acid | metab_7805 | HMDB0013897;PW_C008639;LMFA01030982 | C16648 | 343.2121 | 6.1098 | neg | 2M+Hac-H | C8H14O2 | 0 | 35.1 | -1.9331236 | 1575-72-0 | 0.00641174 | 4.3822 | 4.72 | 4.5471 | 4.3745 | 4.8298 | 4.6554 | 4.7231 | 4.4895 | 4.828 | 4.6101 | 4.7582 | 4.7388 | 4.9798 | 4.9818 | 4.9853 |
| neg_3120 | Phenprobamate | metab_7824 | HMDB0256418 | - | 224.0923 | 6.102 | neg | M+FA-H | C10H13NO2 | 0 | 35.9 | -3.0369596 | - | 0.01901755 | 4.8046 | 5.1228 | 4.8478 | 4.909 | 5.1055 | 5.1222 | 5.3165 | 4.8086 | 5.1031 | 4.6848 | 5.26 | 5.4236 | 4.7808 | 4.7966 | 4.7848 |
| neg_3134 | 6-Dehydrotestosterone glucuronide | metab_7838 | HMDB0010337 | C00178 | 507.2228 | 6.0863 | neg | M+FA-H | C25H34O8 | 0 | 69.3 | -1.6915995 | - | 0.00726458 | 5.9014 | 6.0463 | 6.0203 | 5.797 | 5.8289 | 6.1493 | 6.0006 | 5.8021 | 5.3655 | 5.44 | 5.6248 | 6.0064 | 5.558 | 5.5528 | 5.5524 |
| neg_3141 | (+)-Lysergic acid | metab_7845 | HMDB0242256 | - | 313.119 | 6.0863 | neg | M+FA-H | C16H16N2O2 | 0 | 51.9 | -1.2664983 | - | 0.00731703 | 5.6325 | 5.395 | 5.7639 | 5.579 | 5.2957 | 5.4794 | 5.8992 | 5.7368 | 5.3205 | 5.7325 | 5.2901 | 5.3809 | 5.8352 | 5.8413 | 5.8368 |
| neg_3143 | N-Hydroxy-L-proline | metab_7847 | HMDB0243901 | - | 152.0342 | 6.0863 | neg | M+Na-2H | C5H9NO3 | 0 | 39.7 | 9.87887848 | - | 0.01006032 | 4.4954 | 4.4415 | 4.5112 | 4.5362 | 4.5025 | 4.5277 | 4.5588 | 4.5377 | 4.4944 | 4.6387 | 4.4846 | 4.5262 | 4.5319 | 4.5401 | 4.5335 |
| neg_3151 | Nitrolinoleic acid | metab_7856 | HMDB0255653;HMDB0005049 | C13800 | 370.2229 | 6.0785 | neg | M+FA-H | C18H31NO4 | 0 | 62.9 | -1.9270405 | 774603-04-2 | 0.03922974 | 4.0461 | 3.8486 | 3.8816 | 3.9986 | 3.7615 | 3.7344 | 3.6254 | 4.2716 | 3.877 | 3.0809 | 3.0657 | 4.391 | 4.3119 | 4.283 | 4.3137 |
| neg_3168 | 3,5-Dichloro-2,6-dihydroxybenzoic acid | metab_7873 | HMDB0242164 | - | 220.9407 | 6.0706 | neg | M-H | C7H4Cl2O4 | 0 | 35.6 | -2.8901367 | - | 0.01605012 | 4.3628 | 4.5117 | 4.5873 | 4.659 | 4.6095 | 4.5323 | 4.721 | 4.6644 | 4.7274 | 4.382 | 4.6824 | 4.8495 | 4.6046 | 4.5927 | 4.5924 |
| neg_3172 | L-alpha-amino-epsilon-keto-pimelate | metab_7877 | HMDB0304407 | - | 435.129 | 6.0628 | neg | 2M+Hac-H | C7H10NO5- | 0 | 59.7 | 8.88236907 | - | 0.00745339 | 4.3201 | 4.7929 | 4.6717 | 4.7005 | 4.6371 | 4.6861 | 4.5099 | 4.5402 | 4.7961 | 4.2892 | 4.7615 | 4.679 | 4.6593 | 4.6546 | 4.6608 |
| neg_3173 | Corynoxeine | metab_7878 | HMDB0250480 | - | 417.155 | 6.0628 | neg | M+Cl | C22H26N2O4 | 0 | 56.8 | -9.6157553 | - | 0.0125236 | 4.1108 | 4.8844 | 4.8436 | 4.6979 | 4.5397 | 4.3779 | 4.6766 | 4.5273 | 4.6285 | 4.3892 | 4.7403 | 5.0558 | 4.5869 | 4.5951 | 4.5849 |
| neg_3174 | 3a,11b,21-Trihydroxy-20-oxo-5b-pregnan-18-al | metab_7879 | LMST02030197;PW_C002906;HMDB0006753 | C05474 | 409.2258 | 6.0628 | neg | M+FA-H | C21H32O5 | 0 | 46.4 | 7.19839038 | - | 0.0349242 | 4.6276 | 5.557 | 5.0998 | 5.0727 | 5.3825 | 4.7826 | 5.4817 | 4.7931 | 5.2751 | 4.2048 | 5.682 | 5.5095 | 5.061 | 5.0466 | 5.0769 |
| neg_3178 | (+)-gamma-Hydroxy-L-homoarginine | metab_7883 | HMDB0301838 | C08286 | 239.092 | 6.0628 | neg | M+Cl | C7H16N4O3 | 0 | 65.9 | 1.56016789 | 1616-99-5 | 0.03763563 | 4.4812 | 4.4377 | 4.5027 | 4.4897 | 4.5314 | 4.5512 | 4.5482 | 4.6074 | 4.5652 | 4.5543 | 4.5046 | 4.5516 | 4.4505 | 4.4526 | 4.4795 |
| neg_3180 | 3-Cresotinic acid | metab_7886 | HMDB0002390 | C14088 | 151.039 | 6.0628 | neg | M-H | C8H8O3 | 65.3 | 0 | -7.3100049 | 83-40-9 | 0.00638905 | 4.0371 | 4.2295 | 4.1352 | 4.179 | 4.8742 | 4.822 | 4.2602 | 4.0885 | 4.8637 | 4.1588 | 4.8265 | 4.9781 | 4.5792 | 4.5808 | 4.5846 |
| neg_3183 | Cinncassiol C3 | metab_7889 | HMDB0036859 | C17641 | 427.1969 | 6.055 | neg | M+FA-H | C20H30O7 | 0 | 43.3 | -1.2273311 | 64979-94-8 | 0.01314282 | 5.0346 | 5.6415 | 5.3064 | 5.3489 | 5.7559 | 5.7261 | 5.3731 | 5.1246 | 5.7313 | 5.005 | 5.783 | 5.8251 | 5.6022 | 5.6041 | 5.5934 |
| neg_3189 | 2-Mercaptobenzothiazole | metab_7895 | HMDB0030524 | C14437 | 165.978 | 6.055 | neg | M-H | C7H5NS2 | 82 | 0 | -6.1786806 | 149-30-4 | 0.01077146 | 3.9448 | 2.9683 | 4.1545 | 2.9907 | 3.836 | 3.4998 | 4.6474 | 4.5542 | 3.89 | 4.3394 | 3.4843 | 4.214 | 3.8619 | 3.8642 | 3.8552 |
| neg_3192 | Ptaquiloside | metab_7899 | HMDB0242690 | C19515 | 397.1868 | 6.0471 | neg | M-H | C20H30O8 | 0 | 43.2 | -0.0309168 | 87625-62-5 | 0.00394827 | 4.6274 | 5.0483 | 4.9043 | 4.8583 | 5.2549 | 5.0908 | 4.9074 | 4.7794 | 5.0637 | 4.7721 | 5.1827 | 5.2622 | 5.0022 | 5.0003 | 5.0037 |
| neg_3197 | Precocene II | metab_7904 | HMDB0256742 | C09018 | 265.1078 | 6.0471 | neg | M+FA-H | C13H16O3 | 0 | 39.4 | -1.7585919 | 644-06-4 | 0.00351492 | 4.4402 | 4.4341 | 4.5206 | 4.4795 | 4.4449 | 4.4559 | 4.4444 | 4.4245 | 4.4059 | 4.6522 | 4.4374 | 4.4475 | 4.408 | 4.41 | 4.4109 |
| neg_3209 | (-)-Abscisic acid | metab_7917 | HMDB0243505;LMPR0103050008 | C11060 | 285.1129 | 6.0393 | neg | M+Na-2H | C15H20O4 | 0 | 65.9 | 7.76746794 | - | 0.00744718 | 5.2894 | 5.4628 | 5.3202 | 5.1512 | 5.435 | 5.1697 | 5.289 | 5.1585 | 5.317 | 5.2467 | 5.395 | 5.4092 | 5.395 | 5.3955 | 5.4008 |
| neg_3215 | 5-(3-Pyridyl)-2-hydroxytetrahydrofuran | metab_7924 | HMDB0062407 | C19578 | 210.0765 | 6.0393 | neg | M+FA-H | C9H11NO2 | 0 | 37.1 | -4.3007581 | 53798-73-5 | 0.01327778 | 4.1479 | 4.2907 | 4.1999 | 4.0555 | 4.3575 | 4.3188 | 4.4928 | 4.2297 | 4.226 | 4.1386 | 4.5194 | 4.5854 | 4.0203 | 4.0224 | 4.0311 |
| neg_3216 | L-Dopa | metab_7925 | HMDB0000181;PW_C000117 | C00355 | 196.0607 | 6.0393 | neg | M-H | C9H11NO4 | 0 | 45.5 | -3.9789953 | 59-92-7 | 0.02279926 | 4.2409 | 4.8531 | 4.5345 | 4.6917 | 4.933 | 4.7563 | 4.6542 | 4.247 | 4.7695 | 4.7272 | 4.8219 | 4.6362 | 4.8976 | 4.8997 | 4.9156 |
| neg_3242 | 10-Hydroxymyristic acid methyl ester | metab_7951 | HMDB0031061 | - | 303.2172 | 6.0158 | neg | M+FA-H | C15H30O3 | 0 | 57.4 | -1.8765468 | - | 0.01342698 | 3.7534 | 3.8304 | 3.7076 | 3.5281 | 4.0431 | 3.7835 | 3.6598 | 2.9491 | 4.2929 | 3.3146 | 3.5844 | 4.049 | 4.4902 | 4.481 | 4.4918 |
| neg_3246 | Coumaryl acetate | metab_7955 | HMDB0304297 | C20465 | 237.0763 | 6.0158 | neg | M+FA-H | C11H12O3 | 0 | 30.7 | -2.8243438 | - | 0.01352697 | 3.2483 | 2.9033 | 2.7405 | 2.9099 | 3.288 | 2.7527 | 2.7928 | 2.7038 | 3.0542 | 2.7482 | 2.7331 | 2.729 | 3.914 | 3.9031 | 3.9124 |
| neg_3248 | L-2,3-Dihydrodipicolinate | metab_7957 | HMDB0012247;HMDB0303995 | C03340 | 168.0292 | 6.0158 | neg | M-H | C7H7NO4 | 0 | 43.6 | -5.9791334 | 16052-12-3 | 0.00510814 | 4.6432 | 4.5612 | 4.6793 | 4.6802 | 4.5802 | 4.6215 | 4.6009 | 4.6758 | 4.5923 | 4.7941 | 4.5879 | 4.6192 | 4.6084 | 4.6048 | 4.6043 |
| neg_3261 | Phenol A | metab_7972 | HMDB0256407 | - | 241.1076 | 6.0079 | neg | M+FA-H | C11H16O3 | 0 | 34.8 | -2.6907974 | - | 0.04802525 | 4.1255 | 4.1754 | 4.1186 | 4.152 | 4.243 | 4.0679 | 4.1523 | 4.124 | 4.1691 | 4.2265 | 4.1947 | 4.1203 | 4.2698 | 4.3068 | 4.3062 |
| neg_3263 | 2-Aminopropiophenone | metab_7974 | HMDB0245024 | - | 130.065 | 6.0079 | neg | M-H2O-H | C9H11NO | 0 | 35.8 | -8.2797269 | - | 0.0115615 | 4.0067 | 4.1763 | 4.0352 | 4.1511 | 4.2822 | 4.0134 | 4.4854 | 4.1212 | 4.2687 | 3.9562 | 4.3695 | 4.5234 | 4.3142 | 4.3064 | 4.3158 |
| neg_3269 | 2-Naphthalenesulfonic acid | metab_7979 | HMDB0255446 | C16202 | 253.0172 | 6.0001 | neg | M+FA-H | C10H8O3S | 0 | 39.8 | -2.1314831 | 120-18-3 | 0.0218649 | 4.8211 | 4.991 | 4.9131 | 5.1285 | 5.073 | 4.9735 | 4.9953 | 4.9949 | 5.236 | 4.9119 | 5.0463 | 5.0395 | 5.0011 | 5.0179 | 5.0174 |
| neg_3278 | Huperzine b | metab_7988 | HMDB0253233 | C09866 | 293.1061 | 5.9923 | neg | M+K-2H | C16H20N2O | 0 | 54.7 | -0.3260489 | 103548-82-9 | 0.16849256 | 4.8444 | 3.6965 | 4.3352 | 4.7512 | 3.8297 | 4.0655 | 3.9227 | 4.6451 | 4.1443 | 4.773 | 4.0568 | 4.5242 | 3.9067 | 3.7726 | 3.907 |
| neg_3292 | 2-(3-Carboxy-3-(methylammonio)propyl)-L-histidine | metab_8001 | HMDB0011654 | C04692 | 270.1344 | 5.9766 | neg | M-H | C11H19N4O4+ | 0 | 35.8 | 3.98752854 | - | 0.00881882 | 4.7923 | 4.5124 | 4.5591 | 4.6426 | 4.3061 | 4.2836 | 4.758 | 4.3273 | 4.1839 | 5.0143 | 4.0565 | 4.4718 | 4.8517 | 4.8588 | 4.853 |
| neg_3294 | S-Phenyl-L-cysteine | metab_8003 | HMDB0257425 | - | 242.0486 | 5.9766 | neg | M+FA-H | C9H11NO2S | 0 | 47.1 | -3.2635123 | - | 0.17493033 | 3.9008 | 2.5605 | 3.9032 | 3.9314 | 4.015 | 3.3774 | 4.2719 | 4.0532 | 3.8644 | 3.4114 | 4.1076 | 4.3533 | 3.4231 | 3.2827 | 3.3155 |
| neg_3299 | Cis-3-Hexenyl pyruvate | metab_8008 | HMDB0038276 | - | 151.0753 | 5.9766 | neg | M-H2O-H | C9H14O3 | 0 | 39.4 | -6.8754192 | 68133-76-6 | 0.01139667 | 4.524 | 4.957 | 4.8017 | 4.8963 | 5.1554 | 5.2016 | 4.7739 | 4.5842 | 5.2747 | 4.819 | 5.0304 | 5.0086 | 4.9949 | 5.0041 | 4.9965 |
| neg_3304 | Cyclohexanecarboxylic acid | metab_8014 | HMDB0031342 | C09822 | 301.1652 | 5.9688 | neg | 2M+FA-H | C7H12O2 | 0 | 45.2 | -1.9515008 | 98-89-5 | 0.00473687 | 4.1501 | 4.2173 | 4.03 | 3.78 | 4.2982 | 4.1707 | 4.1274 | 3.7986 | 4.1834 | 4.1244 | 4.3435 | 4.3234 | 4.4043 | 4.4063 | 4.4022 |
| neg_3308 | Bemcentinib | metab_8018 | HMDB0244307 | - | 541.2647 | 5.9648 | neg | M+Cl | C30H34N8 | 0 | 33.7 | 9.23578562 | - | 0.02495003 | 4.6193 | 5.1263 | 5.017 | 5.1772 | 4.7017 | 4.7427 | 4.8464 | 4.7562 | 4.7789 | 4.757 | 4.6143 | 4.5713 | 4.5468 | 4.5487 | 4.5663 |
| neg_331 | CDP-DG(20:4(6E,8Z,11Z,13E)-2OH(5S,15S)/a-13:0) | metab_8020 | HMDB0292812 | - | 990.4524 | 5.3514 | neg | M-2H, M-H | C45H75N3O17P2 | 0 | 43.6 | 2.47897989 | - | 0.05722162 | 5.6748 | 4.9581 | 5.5072 | 4.9398 | 4.958 | 5.0708 | 5.3634 | 5.394 | 5.0738 | 5.3759 | 5.4291 | 5.3091 | 4.9096 | 4.8607 | 4.8955 |
| neg_3312 | (2s)-2-[6-(Sulfooxy)naphthalen-2-yl]propanoic acid | metab_8023 | HMDB0242129 | - | 277.0203 | 5.9648 | neg | M-H2O-H | C13H12O6S | 0 | 56.9 | 9.12563201 | - | 0.01070898 | 4.8901 | 4.9055 | 4.9936 | 4.9743 | 5.4187 | 5.4414 | 4.9806 | 5.0507 | 5.5267 | 4.8591 | 5.3785 | 5.3593 | 5.353 | 5.3617 | 5.3545 |
| neg_3316 | Sulcatone | metab_8027 | HMDB0035915 | C07287 | 171.1017 | 5.9648 | neg | M+FA-H | C8H14O | 0 | 30.7 | -7.9116754 | 110-93-0 | 0.01972415 | 4.7372 | 4.4235 | 4.5283 | 4.4614 | 4.4805 | 4.5718 | 4.8255 | 4.2918 | 4.2295 | 4.5209 | 4.55 | 4.65 | 4.6116 | 4.6135 | 4.5977 |
| neg_3321 | Phaseolic acid | metab_8033 | LMFA01060200;HMDB0031897 | C10483 | 243.1233 | 5.9584 | neg | M-H2O-H | C12H22O6 | 0 | 54.7 | -1.7837258 | 53755-04-7;29533-15-1 | 0.00906135 | 4.6617 | 4.531 | 4.5241 | 4.5307 | 4.7109 | 4.507 | 4.5825 | 4.4863 | 4.6851 | 4.6572 | 4.639 | 4.7251 | 4.7414 | 4.7485 | 4.7481 |
| neg_3325 | Esmolol | metab_8037 | PW_C008734;HMDB0014333 | C06980 | 340.176 | 5.9505 | neg | M+FA-H | C16H25NO4 | 0 | 38.3 | -2.0695454 | 103598-03-4 | 0.01939193 | 3.0249 | 3.6788 | 3.7046 | 3.0757 | 4.3586 | 3.9774 | 3.0735 | 3.616 | 4.1877 | 3.6678 | 4.1117 | 4.18 | 4.7925 | 4.8077 | 4.794 |
| neg_3327 | 10-(2,3-Dihydroxypropoxy)-10-oxodecanoic acid | metab_8039 | HMDB0257660 | - | 275.1497 | 5.9505 | neg | M-H | C13H24O6 | 0 | 40.5 | -1.0551244 | - | 0.0109856 | 3.5376 | 2.9106 | 3.0823 | 3.3093 | 3.1889 | 3.4105 | 2.9309 | 3.2736 | 2.9163 | 3.5397 | 3.1753 | 2.9275 | 4.3901 | 4.3989 | 4.3914 |
| neg_3330 | (2S)-2-(Diaminomethylideneamino)-3-phenylpropanoic acid | metab_8043 | HMDB0248298 | - | 206.0927 | 5.9505 | neg | M-H | C10H13N3O2 | 0 | 49 | -3.7336734 | - | 0.01397787 | 4.1756 | 4.7786 | 4.3889 | 4.1836 | 4.3052 | 4.7465 | 4.7799 | 2.5648 | 2.5787 | 3.4383 | 4.7738 | 4.1737 | 4.3441 | 4.3548 | 4.3544 |
| neg_3339 | Abscisic acid | metab_8052 | HMDB0243505;HMDB0036093;HMDB0035140 | C06082 | 301.0828 | 5.9348 | neg | M+K-2H | C15H20O4 | 0 | 32.1 | -7.2638935 | 7773-56-0;21293-29-8 | 0.00940155 | 2.7667 | 4.4149 | 3.9399 | 3.6076 | 4.0112 | 4.832 | 4.434 | 2.6554 | 2.5723 | 2.5698 | 4.9354 | 4.1873 | 4.3642 | 4.3684 | 4.3602 |
| neg_3342 | Cinnamyl alcohol | metab_8056 | HMDB0029697;HMDB0029698 | C02394 | 179.0704 | 5.9348 | neg | M+FA-H | C9H10O | 0 | 61.5 | -7.1375804 | 4407-36-7;104-54-1 | 0.02659567 | 4.1566 | 4.1952 | 4.0729 | 4.0173 | 4.4094 | 4.2284 | 4.078 | 4.0847 | 4.4048 | 3.9941 | 4.4011 | 4.4531 | 4.2413 | 4.2618 | 4.2426 |
| neg_3347 | THIARABINE | metab_8061 | HMDB0258974 | - | 240.0428 | 5.9284 | neg | M-H2O-H | C9H13N3O4S | 0 | 51.6 | -7.9622962 | - | 0.0068273 | 5.0535 | 5.5852 | 5.3961 | 5.5843 | 5.1332 | 5.4016 | 5.2861 | 5.116 | 5.1032 | 5.0039 | 5.5489 | 5.1481 | 5.2645 | 5.2667 | 5.2704 |
| neg_335 | Suberic Acid | metab_8064 | HMDB0000893;LMFA01170001;MJDBOTE0000630 | C08278 | 173.0809 | 5.4459 | neg | M-H, M+Na-2H | C8H14O4 | 83.2 | 0 | -5.753831 | 505-48-6 | 0.00494202 | 5.2953 | 5.3956 | 5.2189 | 5.2066 | 5.4555 | 5.324 | 5.3425 | 5.166 | 5.3917 | 5.2077 | 5.4721 | 5.4438 | 5.5986 | 5.5951 | 5.5946 |
| neg_3365 | Nitrazepam | metab_8080 | HMDB0015534 | C07487 | 280.0725 | 5.9074 | neg | M-H | C15H11N3O3 | 0 | 33 | -1.0743452 | 146-22-5 | 0.01855384 | 3.8829 | 4.8715 | 4.4177 | 4.2581 | 4.1802 | 4.7632 | 4.7713 | 3.9029 | 3.9169 | 3.9474 | 5.0867 | 4.4554 | 4.1709 | 4.1856 | 4.1727 |
| neg_3381 | Vulgarin | metab_8098 | HMDB0036130 | C09600 | 285.1128 | 5.894 | neg | M+Na-2H | C15H20O4 | 0 | 71.4 | 7.62650432 | 3162-56-9 | 0.0146408 | 5.3111 | 5.3924 | 5.3951 | 5.2714 | 5.2488 | 5.0902 | 5.4953 | 5.574 | 5.2002 | 5.1108 | 5.2803 | 5.3781 | 5.2036 | 5.2151 | 5.2048 |
| neg_3382 | Apigenidin | metab_8099 | HMDB0303074 | C08574 | 254.0567 | 5.894 | neg | M-H | C15H11O4+ | 0 | 39.8 | -6.8691854 | - | 0.03413842 | 4.5031 | 5.6183 | 5.128 | 5.0426 | 5.1601 | 5.5856 | 5.5388 | 1.8731 | 1.8869 | 2.8563 | 5.8902 | 5.2916 | 5.0344 | 5.0605 | 5.0358 |
| neg_3386 | 15-Deacetylneosolaniol | metab_8102 | HMDB0036157 | C02951 | 321.1352 | 5.8836 | neg | M-H2O-H | C17H24O7 | 0 | 43.3 | 2.40336793 | 76348-84-0 | 0.01303782 | 4.5552 | 3.2763 | 4.5794 | 3.5591 | 3.9566 | 4.5767 | 5.0581 | 3.268 | 3.2819 | 4.4626 | 4.2986 | 4.2623 | 4.5549 | 4.5657 | 4.5632 |
| neg_339 | Indolelactic acid | metab_8106 | HMDB0000671 | C02043 | 204.0658 | 5.514 | neg | M-H2O-H, M-H | C11H11NO3 | 83.4 | 0 | -3.8571739 | 7417-65-4 | 0.00470821 | 4.8076 | 4.911 | 4.8441 | 5.1805 | 4.6815 | 4.823 | 4.8864 | 4.8505 | 4.8965 | 4.6075 | 4.9394 | 5.2821 | 5.6546 | 5.652 | 5.6561 |
| neg_3391 | Dhesn | metab_8108 | HMDB0251109 | - | 530.2787 | 5.8762 | neg | M-H2O-H | C30H39N5O5 | 0 | 32.9 | 2.66583681 | - | 0.01488396 | 4.7019 | 5.3131 | 3.3182 | 4.1325 | 5.2314 | 5.4586 | 5.2188 | 3.2968 | 5.0222 | 5.1999 | 5.6011 | 4.5206 | 4.9863 | 4.9981 | 4.9878 |
| neg_3395 | 4-Vinylcyclohexene dioxide | metab_8111 | HMDB0246593 | C19311 | 185.081 | 5.8762 | neg | M+FA-H | C8H12O2 | 0 | 39.1 | -6.4551779 | - | 0.00935707 | 4.3975 | 4.4202 | 4.3052 | 4.3312 | 4.3765 | 4.3798 | 4.3896 | 4.298 | 4.3801 | 4.4277 | 4.3416 | 4.4132 | 4.4069 | 4.4089 | 4.4011 |
| neg_3413 | Methylphenobarbital | metab_8131 | HMDB0014987 | C07829 | 281.0676 | 5.8362 | neg | M+Cl | C13H14N2O3 | 0 | 40.4 | -8.93959 | 115-38-8 | 0.04392612 | 2.7727 | 4.9332 | 4.0843 | 4.1064 | 4.1022 | 4.769 | 4.3652 | 2.7928 | 2.8067 | 2.8372 | 5.0311 | 3.985 | 4.0742 | 4.0416 | 4.0759 |
| neg_3415 | L-arginino-succinate | metab_8133 | HMDB0304399 | - | 270.098 | 5.8312 | neg | M-H2O-H | C10H17N4O6- | 0 | 36.5 | 3.55372133 | - | 0.02752081 | 2.9574 | 3.4254 | 3.232 | 3.0082 | 4.5757 | 3.7958 | 3.0061 | 3.3087 | 4.3758 | 3.0219 | 4.2565 | 4.4584 | 4.1972 | 4.1772 | 4.1988 |
| neg_3416 | (3S,5R,6R,7E)-3,5,6-Trihydroxy-7-megastigmen-9-one | metab_8134 | HMDB0038736 | - | 287.1496 | 5.8233 | neg | M+FA-H | C13H22O4 | 0 | 57.1 | -1.6016765 | - | 0.03775705 | 4.7469 | 4.3416 | 4.5343 | 4.899 | 4.5536 | 4.6441 | 5.5948 | 4.418 | 4.7412 | 4.865 | 4.8572 | 4.6902 | 4.6935 | 4.6855 | 4.7168 |
| neg_3425 | Imperatorin | metab_8144 | HMDB0034376;MJDBOTE0000939 | C09269 | 315.0871 | 5.8008 | neg | M+FA-H | C16H14O4 | 0 | 55.9 | -1.0374451 | 482-44-0 | 0.0199056 | 4.7857 | 4.9369 | 4.896 | 4.7404 | 4.639 | 4.2218 | 4.8751 | 4.9011 | 4.4695 | 4.4402 | 4.707 | 4.8722 | 4.2807 | 4.2827 | 4.2965 |
| neg_3428 | 3-[4-(sulfooxy)phenyl]propanoic acid | metab_8147 | HMDB0125171 | - | 245.012 | 5.7889 | neg | M-H | C9H10O6S | 0 | 60.2 | -2.0256613 | - | 0.01019013 | 5.0863 | 4.9636 | 4.8431 | 4.6679 | 5.4168 | 5.4532 | 5.1991 | 4.9858 | 5.434 | 4.9433 | 5.2212 | 5.1128 | 5.2568 | 5.2654 | 5.2594 |
| neg_3429 | 2-Methoxy-4-methylphenol sulfate | metab_8148 | HMDB0304925 | - | 239.0014 | 5.784 | neg | M+Na-2H | C8H10O5S | 0 | 51.2 | 8.59424562 | - | 0.00302968 | 4.3245 | 4.6629 | 4.2286 | 4.3338 | 4.8208 | 4.6383 | 4.347 | 4.427 | 4.8524 | 4.1359 | 4.7794 | 4.7782 | 4.8149 | 4.817 | 4.8145 |
| neg_3442 | Cloethocarb | metab_8160 | HMDB0250338 | C18951 | 240.0427 | 5.7681 | neg | M-H2O-H | C11H14ClNO4 | 0 | 39.8 | -2.354187 | 51487-69-5 | 0.03040273 | 4.5112 | 4.4591 | 4.3658 | 4.2215 | 4.2802 | 4.2698 | 4.3047 | 4.2296 | 4.4126 | 4.0965 | 4.0473 | 4.2762 | 4.0185 | 4.0266 | 4.0006 |
| neg_3444 | 4-Hydroxy-3-methoxy-cinnamoylglycine | metab_8162 | HMDB0059747 | C02564 | 272.0538 | 5.7563 | neg | M+Na-2H | C12H13NO5 | 0 | 35.8 | -1.1314514 | - | 0.00071653 | 4.8212 | 4.6652 | 4.7951 | 4.7862 | 4.6621 | 4.7053 | 4.7065 | 4.3311 | 4.8368 | 4.6533 | 4.4086 | 4.6582 | 4.4892 | 4.4898 | 4.4893 |
| neg_3445 | Oxonal | metab_8163 | HMDB0255997 | - | 193.0498 | 5.7563 | neg | M+FA-H | C9H8O2 | 0 | 36.4 | -5.8084435 | - | 0.02406484 | 4.3679 | 4.5068 | 4.4654 | 4.384 | 4.4854 | 4.9035 | 4.6776 | 4.277 | 4.2926 | 4.2963 | 4.9513 | 4.5273 | 4.5248 | 4.5433 | 4.5426 |
| neg_3449 | 2-(4-Methoxyphenyl)ethyl hydrogen sulfate | metab_8166 | HMDB0304915 | - | 231.0327 | 5.7331 | neg | M-H | C9H12O5S | 0 | 56.8 | -2.6485853 | - | 0.00845162 | 5.9199 | 6.2099 | 5.9801 | 5.9476 | 6.1141 | 6.1455 | 5.9407 | 5.9011 | 6.1417 | 5.8342 | 6.1357 | 6.1597 | 6.0088 | 6.0154 | 6.015 |
| neg_3452 | 1,4-Cyclohexanedicarboxylic Acid | metab_8170 | - | - | 171.0653 | 5.7217 | neg | M-H | C8H12O4 | 66.9 | 0 | -5.8244555 | - | 0.01011553 | 5.3686 | 5.3097 | 5.3453 | 5.3306 | 5.2933 | 5.3236 | 5.3176 | 5.4068 | 5.3225 | 5.4055 | 5.2941 | 5.3026 | 5.3756 | 5.3835 | 5.383 |
| neg_3465 | Pyridoxine 5'-phosphate | metab_8181 | HMDB0001319;PW_C001017 | C00627 | 230.0201 | 5.6251 | neg | M-H2O-H | C8H12NO6P | 0 | 34.1 | -9.177712 | 447-05-2 | 0.01246477 | 4.6116 | 3.0162 | 4.1961 | 3.0386 | 4.3248 | 3.9558 | 4.9697 | 3.0079 | 3.0218 | 3.5702 | 3.964 | 4.1024 | 4.8371 | 4.8468 | 4.8463 |
| neg_3470 | Geranyl acetate | metab_8185 | HMDB0035157 | C09861 | 241.144 | 5.6054 | neg | M+FA-H | C12H20O2 | 0 | 33.7 | -2.901187 | 105-87-3 | 0.02236821 | 4.0145 | 4.038 | 3.7806 | 3.308 | 3.9243 | 3.8012 | 4.1207 | 3.594 | 3.6213 | 3.1493 | 4.0794 | 4.0124 | 4.2008 | 4.2181 | 4.2175 |
| neg_3483 | Isoproterenol | metab_8193 | PW_C009493;HMDB0015197 | C07056 | 256.1186 | 5.5701 | neg | M+FA-H | C11H17NO3 | 0 | 39.2 | -2.1177939 | 7683-59-2 | 0.01152697 | 4.7375 | 4.6254 | 4.4077 | 4.5922 | 4.0151 | 3.4187 | 4.8916 | 4.4881 | 3.9399 | 4.5939 | 4.1086 | 4.2042 | 4.8294 | 4.8216 | 4.831 |
| neg_3489 | Phenethylamine glucuronide | metab_8198 | HMDB0010323 | C03033 | 278.1029 | 5.5409 | neg | M-H2O-H | C14H19NO6 | 0 | 54.5 | -1.5645931 | - | 0.0034523 | 4.0986 | 4.1828 | 3.2405 | 3.9591 | 3.4939 | 3.7029 | 3.4156 | 4.0217 | 3.7576 | 3.8082 | 4.0465 | 3.9241 | 4.8504 | 4.8526 | 4.8498 |
| neg_3497 | Indole-3-acetaldehyde | metab_8206 | HMDB0001190 | C00637 | 158.06 | 5.5105 | neg | M-H | C10H9NO | 67.8 | 0 | -7.0357195 | 2591-98-2 | 0.02248883 | 2.7969 | 3.0941 | 2.9955 | 3.3191 | 2.7284 | 2.7541 | 3.0271 | 3.0785 | 2.9787 | 2.7593 | 3.4956 | 3.7871 | 4.0403 | 4.0577 | 4.0569 |
| neg_3513 | Tyrosol | metab_8221 | HMDB0004284;MJDBOTE0001325 | C06044 | 183.0654 | 5.4414 | neg | M+FA-H | C8H10O2 | 0 | 49.7 | -6.6487654 | 501-94-0 | 0.00290917 | 5.5726 | 5.6208 | 5.3706 | 5.4603 | 5.6623 | 5.0956 | 4.5806 | 5.5101 | 5.5614 | 5.6627 | 5.4224 | 5.3984 | 5.4049 | 5.403 | 5.4025 |
| neg_3520 | Phenylalanyltryptophan | metab_8229 | HMDB0029006 | - | 350.1505 | 5.4116 | neg | M-H | C20H21N3O3 | 0 | 74.8 | -1.4059794 | 24587-41-5 | 0.00451701 | 4.3313 | 4.511 | 4.3038 | 4.7368 | 4.5985 | 4.5978 | 4.7914 | 4.5275 | 4.3166 | 4.6695 | 4.5848 | 4.6925 | 4.8135 | 4.8172 | 4.8141 |
| neg_3527 | 16-Ketoestradiol | metab_8234 | HMDB0000406;LMST02010047 | C14383 | 617.3144 | 5.3514 | neg | 2M+FA-H | C18H22O3 | 0 | 55.8 | 4.23100521 | 566-75-6 | 0.01803968 | 5.0113 | 5.4841 | 5.1833 | 5.2527 | 4.9826 | 4.7939 | 4.6926 | 4.6676 | 4.9153 | 5.2879 | 4.9913 | 5.1567 | 5.0289 | 5.0308 | 5.0163 |
| neg_3531 | Ala-Thr-Trp-Leu-Pro-Pro-Arg | metab_8239 | HMDB0249908 | - | 876.41 | 5.3306 | neg | M+K-2H | C40H61N11O9 | 0 | 67.7 | -4.7204312 | - | 0.12092953 | 5.6332 | 4.7278 | 5.0134 | 5.0069 | 4.7481 | 4.7807 | 4.7118 | 4.9233 | 4.8116 | 5.2862 | 5.1683 | 5.1753 | 4.752 | 4.6636 | 4.6639 |
| neg_3544 | Methotrexate | metab_8253 | HMDB0014703;PW_C009057;HMDB0248287;MJDBOTE0000857 | C01937 | 489.1395 | 5.2008 | neg | M+Cl | C20H22N8O5 | 0 | 37.6 | -2.7029739 | 59-05-2 | 0.01846861 | 4.902 | 5.2301 | 5.0791 | 5.0487 | 5.0836 | 4.9923 | 5.1712 | 4.9566 | 5.0415 | 4.7591 | 5.1449 | 5.1556 | 4.9674 | 4.9694 | 4.9545 |
| neg_3547 | 6-Hydroxy-4-methoxy-3-(3-methyl-2-butenyl)-2-(2-phenylethenyl)benzoic acid | metab_8256 | HMDB0039439 | C10264 | 359.1243 | 5.1851 | neg | M+Na-2H | C21H22O4 | 0 | 60.4 | -6.3352177 | 87402-83-3 | 0.03372281 | 2.9606 | 4.3907 | 3.7556 | 3.8238 | 3.5951 | 3.0198 | 3.6299 | 3.6123 | 2.9946 | 3.0251 | 3.1166 | 3.0059 | 4.2038 | 4.1791 | 4.1782 |
| neg_3549 | 3-Methyladipic Acid | metab_8258 | HMDB0000555;MJDBOTE0000616 | - | 159.0652 | 5.1851 | neg | M-H | C7H12O4 | 38.6 | 0 | -6.750062 | 1/3/58 | 0.00520931 | 5.1348 | 5.4142 | 5.1342 | 5.1461 | 5.5088 | 5.3405 | 5.2791 | 5.0204 | 5.3478 | 5.0735 | 5.3878 | 5.4945 | 5.2309 | 5.2279 | 5.2324 |
| neg_3551 | 1,2,3-Benzotriazine | metab_8261 | HMDB0249031 | - | 130.0398 | 5.1801 | neg | M-H | C7H5N3 | 0 | 35 | -9.4466671 | - | 0.02516283 | 3.8515 | 4.4243 | 4.2156 | 3.755 | 4.3352 | 5.3524 | 4.7655 | 2.3742 | 2.3881 | 2.9543 | 5.3974 | 4.4622 | 4.4276 | 4.4298 | 4.4474 |
| neg_3555 | Acetyl-DL-Leucine | metab_8265 | HMDB0011756 | C02710 | 172.0969 | 5.159 | neg | M-H | C8H15NO3 | 74.9 | 0 | -5.7296973 | 1188-21-2 | 0.03024514 | 4.6929 | 5.0144 | 4.805 | 4.8027 | 4.7793 | 4.8839 | 4.866 | 4.6028 | 4.684 | 4.697 | 4.8574 | 4.8385 | 4.7748 | 4.7768 | 4.7983 |
| neg_3559 | 2-Carboxy-5,7-dimethyl-4-octanolide | metab_8269 | HMDB0030986 | - | 259.1183 | 5.1344 | neg | M+FA-H | C11H18O4 | 0 | 32.7 | -1.8801516 | - | 0.01747777 | 3.9155 | 3.246 | 3.9114 | 4.3956 | 3.9794 | 3.9298 | 4.8651 | 3.8895 | 3.9256 | 4.083 | 4.1978 | 4.0653 | 3.9106 | 3.9127 | 3.8986 |
| neg_3565 | Vanilloyl glucose | metab_8275 | HMDB0302470 | C20470 | 351.0691 | 5.0994 | neg | M+Na-2H | C14H18O9 | 0 | 45.8 | -1.8481655 | - | 0.0082143 | 4.3429 | 4.2783 | 4.812 | 4.8157 | 4.2093 | 4.0409 | 4.7847 | 4.8594 | 4.0703 | 4.2791 | 4.3711 | 4.6204 | 4.4667 | 4.4622 | 4.4597 |
| neg_3566 | P-Cresol glucuronide | metab_8276 | HMDB0011686 | - | 283.0819 | 5.0994 | neg | M-H | C13H16O7 | 0 | 58.6 | -1.5057792 | - | 0.0073256 | 5.8929 | 5.855 | 6.3156 | 6.3242 | 5.7754 | 5.6555 | 6.2514 | 6.3476 | 5.7828 | 5.8749 | 5.9689 | 6.1524 | 5.946 | 5.9522 | 5.9501 |
| neg_3569 | Salicyluric acid | metab_8278 | HMDB0000840 | C07588 | 194.045 | 5.0792 | neg | M-H | C9H9NO4 | 65.1 | 0 | -4.3821726 | 487-54-7 | 0.03619208 | 4.2847 | 4.477 | 4.1292 | 4.1773 | 4.901 | 4.6926 | 4.3845 | 4.0837 | 4.8177 | 4.1469 | 4.8767 | 5.0075 | 4.9121 | 4.9398 | 4.9393 |
| neg_3571 | 5-Hydroxynorvaline-betaxanthin | metab_8281 | HMDB0304638 | C14133 | 360.9348 | 5.064 | neg | M+FA-H | C14H8Cl4 | 0 | 43.9 | -4.4487354 | - | 0.06995984 | 3.463 | 4.7059 | 3.8878 | 3.5138 | 4.3217 | 4.9671 | 4.7253 | 3.4831 | 3.4971 | 3.5275 | 5.01 | 4.1538 | 4.0792 | 4.1192 | 4.1397 |
| neg_3595 | P-Anisic acid | metab_8304 | HMDB0001101 | C02519 | 151.0389 | 4.9197 | neg | M-H | C8H8O3 | 45.5 | 0 | -7.4643463 | 100-09-4 | 0.02659369 | 4.1664 | 4.2838 | 4.2075 | 4.2139 | 4.4736 | 4.3942 | 4.2574 | 4.0818 | 4.3968 | 4.1792 | 4.444 | 4.4765 | 4.357 | 4.3774 | 4.3769 |
| neg_3604 | 4-Vinylphenol sulfate | metab_8313 | HMDB0062775 | - | 245.0121 | 4.8296 | neg | M+FA-H | C8H8O4S | 0 | 54.5 | -2.3817678 | 131351-90-1 | 0.0159269 | 4.1882 | 4.733 | 4.2232 | 4.4535 | 4.7423 | 4.7528 | 4.4471 | 3.7468 | 4.7484 | 4.3164 | 4.6526 | 4.4281 | 4.5314 | 4.5425 | 4.53 |
| neg_3607 | Alpha-D-glucuronate 1-phosphate | metab_8316 | HMDB0304535 | - | 600.9821 | 4.814 | neg | 2M+Hac-H | C6H8O10P-3 | 0 | 37.9 | -5.1970156 | - | 0.00781939 | 5.0535 | 5.382 | 5.4883 | 5.6279 | 4.9623 | 4.8588 | 5.4646 | 5.4892 | 5.1038 | 4.988 | 5.4613 | 5.3815 | 5.1339 | 5.1315 | 5.1272 |
| neg_3612 | P-Tolyl Sulfate | metab_8322 | - | - | 187.0061 | 4.814 | neg | M-H | C7H8O4S | 74.2 | 0 | -5.0283949 | - | 0.00596138 | 7.7888 | 7.9271 | 7.9652 | 8.0906 | 7.7616 | 7.7465 | 7.9992 | 8.0385 | 7.8619 | 7.8153 | 8.0321 | 7.9749 | 7.8146 | 7.8193 | 7.8189 |
| neg_3634 | Myricetin | metab_8346 | LMPK12110001;HMDB0002755;MJDBOTE0000381 | C10107 | 339.014 | 4.6594 | neg | M+Na-2H | C15H10O8 | 0 | 63.4 | 5.66816658 | 529-44-2 | 0.01778047 | 4.7095 | 4.7032 | 4.9061 | 4.8983 | 4.4459 | 4.5066 | 4.7805 | 4.9307 | 4.4915 | 4.554 | 4.7332 | 4.6936 | 4.5977 | 4.6117 | 4.5991 |
| neg_3656 | O-methoxycatechol-O-sulphate | metab_8370 | HMDB0060013 | - | 203.0011 | 4.5135 | neg | M-H | C7H8O5S | 0 | 50.8 | -4.089843 | - | 0.01442653 | 5.528 | 5.7847 | 5.6321 | 5.7775 | 6.2114 | 6.1718 | 5.7606 | 5.6955 | 6.257 | 5.3826 | 6.2821 | 6.373 | 6.0183 | 6.0298 | 6.0199 |
| neg_3668 | Xi-2,3-Dihydro-3,5-dihydroxy-6-methyl-4H-pyran-4-one | metab_8381 | HMDB0036380 | - | 203.0553 | 4.403 | neg | M+FA-H | C7H10O4 | 0 | 43.4 | -5.243701 | - | 0.0096765 | 2.5959 | 3.2157 | 2.6315 | 2.6797 | 5.1857 | 5.0277 | 2.6386 | 2.6101 | 5.0742 | 2.9742 | 4.8389 | 4.8508 | 4.6428 | 4.6508 | 4.6444 |
| neg_3670 | Niridazole | metab_8384 | HMDB0255625 | C19268 | 259.0132 | 4.3951 | neg | M+FA-H | C6H6N4O3S | 0 | 32.7 | -5.022968 | 61-57-4 | 0.02120844 | 4.9733 | 4.6522 | 4.9046 | 4.8809 | 4.6272 | 4.4833 | 4.8329 | 4.7882 | 4.5094 | 4.799 | 4.5067 | 4.4789 | 4.6637 | 4.6804 | 4.6655 |
| neg_3675 | N-Acetylserotonin | metab_8389 | PW_C000956;HMDB0001238 | C00978 | 263.1033 | 4.3584 | neg | M+FA-H | C12H14N2O2 | 0 | 32.8 | -1.8901614 | 1210-83-9 | 0.03700121 | 4.3667 | 4.6837 | 4.7364 | 4.6046 | 4.1395 | 4.5321 | 4.3982 | 4.9028 | 4.347 | 4.2203 | 4.8972 | 4.2017 | 4.3566 | 4.3851 | 4.3845 |
| neg_3676 | Indoxylsulfuric acid | metab_8390 | HMDB0000682 | - | 212.0015 | 4.3662 | neg | M-H | C8H7NO4S | 71.4 | 0 | -3.7515128 | 487-94-5 | 0.00444091 | 5.2341 | 5.7288 | 5.6473 | 5.6727 | 5.6105 | 5.0531 | 5.5234 | 5.4669 | 5.5621 | 5.3406 | 5.5469 | 5.5061 | 5.3553 | 5.3589 | 5.3584 |
| neg_3681 | 1-Piperideine-2-carboxylic acid | metab_8396 | HMDB0001084 | C04092 | 148.0392 | 4.3441 | neg | M+Na-2H | C6H9NO2 | 0 | 34.9 | 9.74726339 | 2756-89-0 | 0.01187479 | 4.593 | 4.8698 | 4.6872 | 4.6371 | 4.7846 | 4.8257 | 4.6657 | 4.5617 | 4.713 | 4.3216 | 4.7944 | 4.7939 | 4.72 | 4.7281 | 4.7186 |
| neg_3682 | 4-Methyl-1,4-dihydropyridine-3,5-dicarbaldehyde | metab_8397 | HMDB0254370 | - | 132.0442 | 4.3441 | neg | M-H2O-H | C8H9NO2 | 0 | 32.6 | -8.3210388 | - | 0.00749683 | 4.794 | 5.0373 | 4.8987 | 4.7933 | 4.9473 | 5.0059 | 4.8846 | 4.7688 | 4.9363 | 4.5916 | 5.0236 | 5.0261 | 4.9078 | 4.9137 | 4.9132 |
| neg_3687 | Kaltostat | metab_8401 | HMDB0253761;HMDB0029940 | C01768 | 379.0905 | 4.3363 | neg | M-H2O-H | C14H22O13 | 0 | 61.4 | 5.78998993 | 9005-32-7 | 0.00213374 | 6.0106 | 6.3753 | 6.143 | 6.0107 | 6.2481 | 6.3094 | 6.1482 | 5.9689 | 6.2087 | 5.6572 | 6.2916 | 6.3064 | 6.2015 | 6.2002 | 6.1997 |
| neg_3688 | Cyclic-hpmpc | metab_8402 | HMDB0250198 | - | 297.9997 | 4.3363 | neg | M+K-2H | C8H12N3O5P | 0 | 39.7 | -1.2366465 | - | 0.01053042 | 4.8244 | 5.0091 | 4.9158 | 4.8487 | 4.8858 | 5.0032 | 4.9001 | 4.7953 | 4.9049 | 4.6571 | 4.9684 | 4.8988 | 4.9168 | 4.9253 | 4.9183 |
| neg_3690 | Ecgonine | metab_8405 | HMDB0006548 | C10858 | 230.1027 | 4.3363 | neg | M+FA-H | C9H15NO3 | 0 | 37.9 | -3.5520188 | 481-37-8 | 0.00498857 | 3.0396 | 2.7883 | 2.8925 | 2.8107 | 2.7934 | 2.8191 | 3.6814 | 2.78 | 3.2821 | 3.3805 | 2.8093 | 3.641 | 4.3338 | 4.3381 | 4.3354 |
| neg_3698 | N-Arachidonoyl Glycine | metab_8413 | HMDB0241918;LMFA08020003;HMDB0005096 | - | 338.0876 | 4.3221 | neg | M-H2O-H | C15H19NO9 | 0 | 33.1 | -1.5548888 | 179113-91-8 | 0.01310579 | 5.3296 | 4.5982 | 5.7158 | 5.7405 | 4.6041 | 4.8854 | 5.6065 | 5.9645 | 4.4914 | 5.2786 | 5.3991 | 5.4502 | 5.0918 | 5.0829 | 5.0935 |
| neg_3710 | Ferulic acid 4-O-sulfate | metab_8428 | HMDB0029200 | - | 273.0071 | 4.2451 | neg | M-H | C10H10O7S | 0 | 43.8 | -1.1785988 | 151481-53-7 | 0.01559846 | 4.6871 | 4.8851 | 4.4521 | 4.6157 | 4.8302 | 4.8626 | 4.7709 | 4.5979 | 4.7125 | 4.2985 | 4.9499 | 4.8455 | 4.6478 | 4.6597 | 4.6595 |
| neg_3717 | 5-Ethoxy-4,5-dihydro-2(3H)furanone | metab_8435 | HMDB0031214 | - | 151.039 | 4.2302 | neg | M+Na-2H | C6H10O3 | 0 | 35.1 | 9.95599277 | 932-85-4 | 0.02207013 | 4.1242 | 4.2913 | 4.2184 | 4.2985 | 4.3925 | 4.3489 | 4.2561 | 4.125 | 4.443 | 4.2502 | 4.383 | 4.359 | 4.3039 | 4.3212 | 4.3201 |
| neg_3723 | Trans-4-Hydroxycyclohexylacetic acid | metab_8441 | HMDB0000909 | - | 203.0917 | 4.206 | neg | M+FA-H | C8H14O3 | 0 | 30.9 | -5.1902527 | 68592-23-4 | 0.01149939 | 3.784 | 3.9498 | 3.6853 | 3.5434 | 4.1071 | 3.8074 | 4.0937 | 3.4191 | 4.1158 | 3.4079 | 4.1723 | 4.0466 | 4.4559 | 4.4476 | 4.447 |
| neg_3734 | Phenyl glucuronide | metab_8453 | HMDB0060014 | - | 269.0663 | 4.1216 | neg | M-H | C12H14O7 | 47.9 | 0 | -1.3870624 | - | 0.00802582 | 3.9549 | 4.9777 | 5.3974 | 4.5963 | 4.9187 | 4.7061 | 4.5343 | 3.9749 | 4.6226 | 4.2595 | 4.8483 | 5.0565 | 4.5775 | 4.5838 | 4.5833 |
| neg_3747 | Fructose 1-phosphate | metab_8467 | HMDB0060467;HMDB0001076;PW_C000847 | C01094 | 296.9793 | 4.048 | neg | M+K-2H | C6H13O9P | 0 | 63.8 | 3.91543964 | 15978-08-2 | 0.02069293 | 4.6603 | 5.0124 | 5.0793 | 5.0242 | 5.1442 | 4.4345 | 4.9528 | 4.8774 | 5.0383 | 4.5884 | 4.9022 | 4.9622 | 4.8261 | 4.8419 | 4.8416 |
| neg_3751 | 9H-Pyrido[3,4-b]indole-3-carboxylic acid | metab_8471 | HMDB0249637 | - | 247.0277 | 4.0297 | neg | M+Cl | C12H8N2O2 | 0 | 39.9 | -1.4796582 | - | 0.01095399 | 3.865 | 4.3667 | 4.0495 | 3.8453 | 4.6742 | 4.5147 | 4.0287 | 3.7165 | 4.5766 | 3.286 | 4.5571 | 4.4256 | 4.6555 | 4.6633 | 4.6547 |
| neg_3761 | 2-Hydroxypyridine | metab_8482 | HMDB0013751 | C02502 | 235.0718 | 3.9838 | neg | 2M+FA-H | C5H5NO | 0 | 49 | -3.2089998 | 142-08-5 | 0.02034514 | 4.3713 | 5.1602 | 4.715 | 4.5061 | 5.0331 | 4.9329 | 4.7605 | 4.4072 | 4.8639 | 4.125 | 5.1112 | 4.8256 | 4.697 | 4.7128 | 4.6983 |
| neg_3762 | Gamma-Glutamylleucine | metab_8483 | HMDB0011171 | - | 259.1295 | 3.9769 | neg | M-H | C11H20N2O5 | 0 | 60.6 | -1.5582884 | 2566-39-4 | 0.01818661 | 5.0453 | 5.1462 | 5.08 | 4.9475 | 5.0885 | 4.9265 | 5.0383 | 5.0043 | 4.8917 | 4.9548 | 4.7808 | 5.074 | 4.9696 | 4.9827 | 4.9687 |
| neg_3765 | N-Acetyl-L-Tyrosine | metab_8486 | HMDB0244966;HMDB0000866;MJDBOTE0000672 | C01657 | 222.0764 | 3.9581 | neg | M-H | C11H13NO4 | 94.9 | 0 | -3.3238769 | 537-55-3 | 0.01516594 | 3.743 | 4.4864 | 4.0602 | 4.2796 | 3.9912 | 4.0843 | 4.2495 | 3.5389 | 3.8425 | 3.172 | 4.3046 | 4.236 | 4.001 | 4.0032 | 4.0133 |
| neg_3770 | Pyrrolodiazepine | metab_8492 | HMDB0257006 | - | 130.0398 | 3.9472 | neg | M-H | C7H5N3 | 0 | 31.8 | -9.475473 | - | 0.01885758 | 3.2678 | 3.9638 | 3.8458 | 2.7828 | 3.6817 | 4.805 | 4.4832 | 2.7521 | 2.7661 | 2.7965 | 4.7845 | 2.7773 | 3.7827 | 3.7974 | 3.784 |
| neg_3774 | 5-Methylpyrogallol sulfate | metab_8495 | HMDB0304924 | - | 218.9962 | 3.928 | neg | M-H | C7H8O6S | 0 | 30.4 | -3.0064944 | - | 0.01287084 | 4.1385 | 4.4624 | 4.0451 | 4.1949 | 4.6638 | 4.6318 | 4.1056 | 4.1605 | 4.8044 | 3.2411 | 4.7283 | 4.5549 | 4.4528 | 4.4547 | 4.4633 |
| neg_3780 | Phenylacetic Acid | metab_8501 | HMDB0000209;PW_C000135 | C07086 | 135.0439 | 3.9134 | neg | M-H | C8H8O2 | 61.1 | 0 | -9.0926727 | 103-82-2 | 0.02422804 | 3.8938 | 3.8776 | 3.7242 | 4.0001 | 3.7089 | 3.842 | 3.8606 | 3.8983 | 3.8992 | 3.7581 | 4.0555 | 4.1729 | 4.0296 | 4.0486 | 4.0316 |
| neg_3784 | Isovalerylglycine | metab_8504 | HMDB0000678;MJDBOTE0001176 | - | 158.0812 | 3.906 | neg | M-H | C7H13NO3 | 36 | 0 | -6.9127708 | 16284-60-9 | 0.01031181 | 5.4062 | 5.5568 | 5.5415 | 5.5277 | 5.6992 | 5.7079 | 5.5242 | 5.489 | 5.6981 | 5.4845 | 5.6224 | 5.8226 | 5.8507 | 5.8587 | 5.8582 |
| neg_3789 | 1,1-Dimethylbiguanide | metab_8508 | HMDB0001921 | C07151 | 164.0706 | 3.8982 | neg | M+Cl | C4H11N5 | 0 | 35.1 | -2.044727 | 1115-70-4 | 0.03268886 | 3.7818 | 4.19 | 4.0089 | 4.0319 | 3.8849 | 4.0424 | 4.0057 | 3.7682 | 3.5893 | 3.7035 | 4.1271 | 4.0906 | 3.9176 | 3.9428 | 3.9421 |
| neg_3791 | Lamivudine sulfoxide | metab_8511 | PW_C040661;HMDB0060599 | - | 281.9936 | 3.8904 | neg | M+K-2H | C8H11N3O4S | 0 | 39.1 | -8.1521235 | - | 0.01077935 | 5.0771 | 4.9175 | 5.0854 | 5.073 | 5.0847 | 5.0773 | 5.0022 | 5.0921 | 5.0356 | 5.1719 | 5.0738 | 5.04 | 5.0297 | 5.0319 | 5.0387 |
| neg_3796 | L-Threo-3-Phenylserine | metab_8516 | HMDB0002184 | C03290 | 162.055 | 3.8904 | neg | M-H2O-H | C9H11NO3 | 0 | 58.3 | -5.9525324 | 6254-48-4 | 0.00963521 | 5.4055 | 5.3442 | 5.4745 | 5.4641 | 5.4056 | 5.458 | 5.3602 | 5.4479 | 5.3987 | 5.5003 | 5.4128 | 5.4311 | 5.4102 | 5.4122 | 5.4183 |
| neg_3810 | Paracetamol sulfate | metab_8533 | HMDB0059911;PW_C040297 | - | 230.0122 | 3.8707 | neg | M-H | C8H9NO5S | 0 | 42.8 | -2.6874516 | 10066-90-7 | 0.02115646 | 4.6365 | 4.6049 | 4.8855 | 4.8052 | 4.8327 | 4.8971 | 4.7926 | 4.6689 | 4.9505 | 4.4334 | 5.114 | 5.0014 | 4.712 | 4.704 | 4.7223 |
| neg_3815 | Tiglylglycine | metab_8537 | HMDB0000959 | - | 156.0655 | 3.8589 | neg | M-H | C7H11NO3 | 65 | 0 | -7.1750923 | 35842-45-6 | 0.01453203 | 4.7964 | 4.4277 | 4.7152 | 4.8068 | 4.6213 | 4.4986 | 4.5145 | 4.6652 | 4.7062 | 4.8469 | 4.3838 | 4.537 | 4.6228 | 4.6152 | 4.6278 |
| neg_3819 | 6-Aminopenicillanic acid | metab_8541 | HMDB0060618 | C02954 | 491.1242 | 3.8431 | neg | 2M+Hac-H | C8H12N2O3S | 0 | 60.8 | -7.7808114 | 551-16-6 | 0.01690341 | 4.4476 | 4.9336 | 4.6832 | 4.733 | 4.465 | 4.4486 | 4.9653 | 4.5908 | 4.2218 | 4.2542 | 4.4493 | 4.6132 | 4.6263 | 4.6374 | 4.6402 |
| neg_3828 | Ethyl glucuronide | metab_8551 | HMDB0010325 | - | 203.0554 | 3.8327 | neg | M-H2O-H | C8H14O7 | 0 | 60.7 | -3.4079879 | 17685-04-0 | 0.03743827 | 3.9819 | 4.5847 | 4.001 | 4.1935 | 4.2806 | 3.9286 | 4.2813 | 3.4616 | 4.0681 | 3.6973 | 4.4137 | 4.0852 | 4.231 | 4.2442 | 4.2633 |
| neg_3829 | 3-Methoxy-4-Hydroxyphenylglycol sulfate | metab_8552 | HMDB0003332 | - | 245.012 | 3.8268 | neg | M-H2O-H | C9H12O7S | 0 | 46.1 | -2.0178265 | 71324-20-4 | 0.03429066 | 3.9975 | 4.517 | 3.8115 | 4.1537 | 4.2319 | 4.4048 | 4.2997 | 4.0202 | 4.2968 | 3.8815 | 4.3415 | 4.1227 | 4.1596 | 4.1617 | 4.1862 |
| neg_383 | 3-Phenyl-1-(2,4,6-trihydroxyphenyl)propan-1-one | metab_8553 | - | - | 257.0815 | 5.9688 | neg | M-H, M+FA-H | C15H14O4 | 62.1 | 0 | -1.580066 | - | 0.01537714 | 4.9304 | 4.8793 | 4.8147 | 4.6451 | 4.777 | 4.5006 | 4.9094 | 4.6808 | 4.6884 | 4.7006 | 4.8853 | 4.946 | 4.7507 | 4.7629 | 4.7523 |
| neg_3845 | Adipic Acid | metab_8569 | HMDB0000448;LMFA01170048 | C06104 | 145.0495 | 3.771 | neg | M-H | C6H10O4 | 85.3 | 0 | -7.8966029 | 124-04-9 | 0.00272148 | 5.1074 | 5.0304 | 5.0544 | 4.9284 | 5.1903 | 5.0896 | 5.0562 | 4.9988 | 5.0857 | 4.9602 | 5.1465 | 5.197 | 5.0859 | 5.087 | 5.0883 |
| neg_3847 | Gynocardin | metab_8571 | HMDB0029913 | C08331 | 324.0719 | 3.7572 | neg | M+Na-2H | C12H17NO8 | 0 | 54.8 | 6.07379984 | 14332-17-3 | 0.02626715 | 5.3231 | 5.0035 | 5.4071 | 4.7314 | 5.1548 | 4.9548 | 5.1948 | 5.3167 | 5.2937 | 5.3144 | 4.8651 | 4.722 | 5.1214 | 5.1023 | 5.1014 |
| neg_3859 | PYRIDOPYRIMIDINE | metab_8582 | HMDB0256973 | - | 130.0398 | 3.7371 | neg | M-H | C7H5N3 | 0 | 44.2 | -9.4428663 | - | 0.05591063 | 3.0862 | 3.8906 | 3.7725 | 2.4639 | 3.5149 | 4.6898 | 4.4337 | 2.4066 | 2.4205 | 2.4509 | 4.6755 | 2.4317 | 3.76 | 3.7179 | 3.7613 |
| neg_386 | 2-(p-Acetamidophenyl)-2-ethylglutarimide | metab_8583 | HMDB0247904 | - | 255.1136 | 5.9074 | neg | M-H2O-H, M-H | C15H18N2O3 | 0 | 61.4 | -1.1944388 | - | 0.01023638 | 4.5626 | 4.1715 | 4.3586 | 4.4125 | 3.0014 | 2.8736 | 4.3315 | 4.3257 | 3.6358 | 4.4583 | 0.1176 | 3.6737 | 4.472 | 4.474 | 4.4805 |
| neg_3867 | 4-Hydroxyphenylacetic acid sulfate | metab_8590 | HMDB0304906 | - | 230.9963 | 3.657 | neg | M-H | C8H8O6S | 0 | 41.6 | -2.6421238 | - | 0.01193476 | 4.7159 | 4.698 | 4.2822 | 4.6889 | 4.7799 | 5.0448 | 4.3743 | 4.3151 | 4.7834 | 3.9868 | 4.9163 | 4.7127 | 4.7097 | 4.719 | 4.7182 |
| neg_3868 | Dihydroxyphenylacetic acid sulfate | metab_8591 | HMDB0304904 | - | 246.9913 | 3.6506 | neg | M-H | C8H8O7S | 0 | 31.4 | -1.8888489 | - | 0.00893583 | 4.1282 | 4.3476 | 3.882 | 3.982 | 4.647 | 4.7198 | 4.1204 | 4.3243 | 4.5424 | 3.1913 | 4.7599 | 4.6727 | 4.1869 | 4.1811 | 4.1884 |
| neg_3876 | Gly-Ile | metab_8600 | HMDB0028844 | - | 187.1079 | 3.5951 | neg | M-H | C8H16N2O3 | 37 | 0 | -4.834527 | - | 0.01130928 | 3.3694 | 3.2058 | 3.5541 | 3.2283 | 2.5497 | 2.4526 | 2.442 | 3.4348 | 3.2236 | 3.2631 | 2.9524 | 3.132 | 4.7289 | 4.7207 | 4.7201 |
| neg_3877 | Indole carboxylic acid sulfate | metab_8601 | HMDB0304912 | - | 239.9967 | 3.5882 | neg | M-H | C9H7NO5S | 0 | 52.6 | -2.3446671 | - | 0.00572811 | 4.0073 | 4.3436 | 4.384 | 4.037 | 5.4918 | 5.6498 | 4.3297 | 4.0311 | 5.4603 | 3.5589 | 5.406 | 5.5696 | 5.0942 | 5.0902 | 5.0897 |
| neg_3881 | 1,2-Diacylglycerol-LD-PE-pool | metab_8606 | HMDB0062269 | - | 117.0544 | 3.5818 | neg | M-H2O-H | C5H12O4 | 0 | 30.5 | -9.5982137 | 115-77-5 | 0.00436018 | 5.9483 | 5.8987 | 5.9631 | 5.9108 | 6.0635 | 6.0503 | 5.8596 | 5.857 | 6.0826 | 5.9003 | 6.0058 | 6.1136 | 6.0883 | 6.0903 | 6.0865 |
| neg_3887 | Glutaric Acid | metab_8611 | HMDB0000661 | C00489 | 131.0337 | 3.5612 | neg | M-H | C5H8O4 | 83.7 | 0 | -9.4237336 | 110-94-1 | 0.01052137 | 5.2505 | 5.1772 | 5.2658 | 5.3919 | 5.4378 | 5.3284 | 5.1887 | 5.2261 | 5.3475 | 5.2323 | 5.2648 | 5.3664 | 5.3091 | 5.3112 | 5.3179 |
| neg_3889 | Aminohippuric acid | metab_8613 | HMDB0001867 | D06890 | 193.061 | 3.5548 | neg | M-H | C9H10N2O3 | 52.5 | 0 | -4.5906808 | 61-78-9 | 0.01056553 | 4.6357 | 4.7141 | 4.4477 | 4.396 | 4.5292 | 4.1822 | 4.5907 | 4.71 | 4.4965 | 4.5217 | 4.0925 | 4.4429 | 4.2942 | 4.3011 | 4.2925 |
| neg_3902 | 2,5-Dihydroxybenzenesulfonic Acid | metab_8629 | HMDB0245498 | - | 188.9854 | 3.4893 | neg | M-H | C6H6O5S | 0 | 39.1 | -4.8342258 | - | 0.0068289 | 6.4478 | 6.8414 | 6.5975 | 6.6802 | 6.9898 | 6.9477 | 6.7072 | 6.5452 | 7.0253 | 6.4336 | 7.0485 | 7.0458 | 6.8309 | 6.8363 | 6.8358 |
| neg_3903 | Mannitol | metab_8630 | HMDB0000765 | C00392 | 203.0553 | 3.4843 | neg | M+Na-2H | C6H14O6 | 0 | 35.1 | 8.83219124 | 69-65-8 | 0.02054541 | 3.9063 | 4.1514 | 4.0033 | 3.948 | 4.1004 | 4.2866 | 4.5754 | 3.6851 | 4.4416 | 4.0418 | 4.2551 | 4.1309 | 4.0815 | 4.0836 | 4.0671 |
| neg_3917 | 2,3-Dihydro-1H-pyrrole-2-carboxylic acid | metab_8643 | HMDB0244902 | - | 225.0874 | 3.4032 | neg | 2M-H | C5H7NO2 | 0 | 32.1 | -2.9368256 | - | 0.01036207 | 5.0192 | 5.1675 | 5.0741 | 5.1532 | 5.1072 | 5.13 | 5.1382 | 5.0493 | 5.0993 | 5.0042 | 5.0633 | 5.2719 | 4.9422 | 4.9443 | 4.9508 |
| neg_3918 | Sulfuric acid 4-methoxyphenyl ester | metab_8644 | HMDB0242192 | - | 203.0012 | 3.4032 | neg | M-H | C7H8O5S | 0 | 46.4 | -4.0043652 | - | 0.02716494 | 2.4808 | 3.7497 | 2.5222 | 2.5315 | 4.8167 | 4.7166 | 2.7712 | 2.5168 | 4.6375 | 2.5452 | 4.6091 | 4.7713 | 4.0558 | 4.074 | 4.0781 |
| neg_392 | 2-Hydroxycampholonic acid | metab_8646 | HMDB0301821 | - | 199.0968 | 6.0001 | neg | M-H, M+Cl | C10H16O4 | 0 | 58.9 | -3.9495448 | - | 0.00568285 | 5.006 | 4.9143 | 4.9106 | 5.0108 | 4.9779 | 4.8775 | 4.9407 | 4.9003 | 4.9948 | 5.0983 | 4.9648 | 5.0746 | 4.9102 | 4.915 | 4.9117 |
| neg_3926 | 2-Hydroxyfelbamate | metab_8649 | PW_C040524;HMDB0060670 | C16582 | 235.0718 | 3.375 | neg | M-H2O-H | C11H14N2O5 | 0 | 45.2 | -2.3140835 | - | 0.0101854 | 4.5624 | 4.45 | 4.4191 | 4.2431 | 4.2654 | 3.2196 | 4.4926 | 4.0621 | 3.8454 | 4.0655 | 4.2977 | 3.8332 | 4.3451 | 4.3472 | 4.3387 |
| neg_3935 | 5'-Deoxyadenosine | metab_8659 | HMDB0001983 | C05198 | 296.0995 | 3.3468 | neg | M+FA-H | C10H13N5O3 | 0 | 33.7 | -2.1850972 | 4754-39-6 | 0.0150425 | 4.8955 | 5.0018 | 4.9205 | 4.8785 | 4.9464 | 4.8614 | 4.8778 | 4.9933 | 5.1046 | 4.8892 | 5.0159 | 5.0164 | 4.6953 | 4.6843 | 4.6838 |
| neg_3937 | L-Phenylalanine | metab_8661 | HMDB0000159;HMDB0250791;PW_C000104;MJDBAA00000196 | C00079 | 164.0706 | 3.3468 | neg | M-H | C9H11NO2 | 94.3 | 0 | -6.5019133 | 63-91-2 | 0.01606786 | 6.0218 | 6.0822 | 6.0296 | 5.9455 | 6.0026 | 6.0188 | 6.0827 | 6.0146 | 5.9567 | 6.026 | 5.9157 | 6.0986 | 6.0413 | 6.0331 | 6.047 |
| neg_3947 | 7,4'-Dihydroxy-8-methylflavan | metab_8672 | HMDB0247289;LMPK12020230 | C09650 | 277.0859 | 3.3279 | neg | M+Na-2H | C16H16O3 | 0 | 44.9 | 4.86187422 | 75412-98-5 | 0.00387273 | 4.467 | 4.6645 | 4.6306 | 4.3 | 4.6101 | 4.4147 | 4.6073 | 4.4627 | 4.2981 | 4.4388 | 4.474 | 4.6503 | 4.7405 | 4.7437 | 4.7414 |
| neg_3953 | L-Agaridoxin | metab_8679 | HMDB0029445 | - | 275.0646 | 3.2941 | neg | M+Na-2H | C11H14N2O5 | 0 | 30.3 | -1.1988996 | - | 0.0080728 | 4.7656 | 4.6642 | 4.4876 | 4.3419 | 4.6193 | 4.2366 | 4.6559 | 4.5461 | 4.4895 | 4.454 | 4.2301 | 4.7082 | 4.6524 | 4.6587 | 4.6582 |
| neg_3954 | Hydroxyphenylacetylglycine | metab_8680 | HMDB0000735 | C05596 | 190.0501 | 3.2897 | neg | M-H2O-H | C10H11NO4 | 0 | 36.8 | -4.2398488 | 28116-23-6 | 0.01004684 | 5.2084 | 5.0699 | 4.9772 | 4.8189 | 5.0888 | 4.7808 | 5.0635 | 4.983 | 4.9124 | 4.9474 | 4.8193 | 5.1538 | 5.0852 | 5.0935 | 5.0869 |
| neg_3958 | 4-Hydroxy-N-phenyl-3,5-bis(1-pyrrolidinylmethyl)benzamide | metab_8684 | HMDB0247889 | - | 416.1781 | 3.271 | neg | M+K-2H | C23H29N3O2 | 0 | 51.6 | 9.31527256 | - | 0.02184074 | 5.529 | 5.1778 | 5.6286 | 5.1579 | 5.2793 | 5.1332 | 5.1645 | 5.2245 | 5.376 | 5.3322 | 5.2645 | 5.4279 | 5.0271 | 5.0442 | 5.0288 |
| neg_3960 | Xanthosine | metab_8685 | PW_C000203;HMDB0000299;MJDBOTE0001063 | C01762 | 283.068 | 3.266 | neg | M-H | C10H12N4O6 | 83.7 | 0 | -1.4287867 | 146-80-5 | 0.02502234 | 4.969 | 5.027 | 5.1405 | 5.0452 | 5.2512 | 5.1901 | 5.0013 | 4.9792 | 5.1212 | 4.9416 | 5.0295 | 5.2559 | 4.872 | 4.8912 | 4.8906 |
| neg_3969 | 2,6-diamino-4-hydroxy-5-formamidopyrimidine | metab_8694 | HMDB0304046;HMDB0011657 | C04744 | 150.041 | 3.1504 | neg | M-H2O-H | C5H7N5O2 | 0 | 48 | -6.8872631 | 77440-13-2 | 0.04247297 | 4.4899 | 4.3725 | 4.3718 | 4.2105 | 4.2605 | 3.9683 | 4.4438 | 4.2919 | 4.3957 | 4.308 | 4.3856 | 4.2597 | 3.799 | 3.801 | 3.8315 |
| neg_3972 | Glucose pyruvate lactate | metab_8698 | HMDB0252788 | - | 335.0605 | 3.1291 | neg | M-H2O-H | C12H18O12 | 0 | 71.3 | -4.2185637 | - | 0.00530844 | 6.094 | 5.9743 | 6.0552 | 5.6284 | 5.9304 | 5.8903 | 6.0053 | 5.928 | 5.9915 | 5.9973 | 5.9318 | 5.9479 | 5.6341 | 5.6343 | 5.6382 |
| neg_3973 | Inosine | metab_8699 | PW_C000127;HMDB0000195;HMDB0247301;MJDBOTE0000386 | C00294 | 267.0731 | 3.1291 | neg | M-H | C10H12N4O5 | 99 | 0 | -1.4087165 | 58-63-9 | 0.00757732 | 7.3863 | 7.2737 | 7.3525 | 6.9352 | 7.2181 | 7.1679 | 7.312 | 7.2271 | 7.291 | 7.2897 | 7.2282 | 7.2279 | 6.9217 | 6.9277 | 6.9272 |
| neg_3975 | [3-(2-Acetamidoethyl)-5-methoxy-1H-indol-6-yl]oxy hydrogen sulfate | metab_8701 | HMDB0257903 | - | 365.0402 | 3.1202 | neg | M+Na-2H | C13H16N2O7S | 0 | 59.7 | -6.7001165 | - | 0.03703317 | 5.0015 | 4.9403 | 5.0372 | 4.5674 | 4.949 | 4.827 | 5.0334 | 4.9082 | 4.948 | 5.0015 | 4.9437 | 4.9287 | 4.4933 | 4.466 | 4.4655 |
| neg_3976 | Rhamnose | metab_8702 | HMDB0000849;MJDBOTE0001343 | C00507 | 163.0601 | 3.1147 | neg | M-H | C6H12O5 | 76.9 | 0 | -6.5968562 | 3615-41-6 | 0.01092683 | 3.8023 | 3.8626 | 3.7583 | 4.0319 | 3.9086 | 3.9159 | 3.8529 | 3.7746 | 4.0323 | 4.056 | 3.8659 | 3.9066 | 4.4279 | 4.4364 | 4.4359 |
| neg_3984 | Deoxyuridine | metab_8711 | HMDB0245528;PW_C000007;HMDB0000012;MJDBOTE0000510 | C00526 | 273.0725 | 2.7744 | neg | M+FA-H | C9H12N2O5 | 45.9 | 0 | -1.345533 | 951-78-0 | 0.02214811 | 4.8184 | 4.7905 | 4.5029 | 4.3464 | 4.7761 | 4.5673 | 4.6249 | 4.7283 | 4.779 | 4.5971 | 4.6727 | 4.7718 | 4.616 | 4.5998 | 4.617 |
| neg_4 | Azelaic Acid | metab_8728 | HMDB0000784;LMFA01170054;MJDBOTE0000344 | C08261 | 187.0967 | 5.9427 | neg | M-H, M+Na-2H, M+Cl, M-H2O-H | C9H16O4 | 48.3 | 0 | -4.7652355 | 123-99-9 | 0.01667918 | 6.0745 | 6.215 | 6.0686 | 5.9484 | 6.3524 | 6.1417 | 6.13 | 6.0247 | 6.3209 | 5.9895 | 6.3989 | 6.3419 | 6.3081 | 6.3213 | 6.3097 |
| neg_4029 | Uridine | metab_8761 | PW_C000202;HMDB0000296;MJDBOTE0000788 | C00299 | 243.0616 | 2.2136 | neg | M-H | C9H12N2O6 | 65.4 | 0 | -2.5049159 | 58-96-8 | 0.01066213 | 5.3855 | 5.3903 | 5.303 | 5.2334 | 5.5482 | 5.3448 | 5.378 | 5.2694 | 5.3748 | 5.3677 | 5.3465 | 5.5299 | 5.254 | 5.256 | 5.2628 |
| neg_4038 | Tricarballylic Acid | metab_8771 | HMDB0031193;MJDBOTE0001362 | C19806 | 175.0238 | 1.9811 | neg | M-H | C6H8O6 | 66.5 | 0 | -5.9678845 | 99-14-9 | 0.01443078 | 3.9275 | 3.9257 | 4.0094 | 3.8938 | 4.5832 | 4.3779 | 4.0039 | 3.9437 | 4.5534 | 4.1216 | 4.1782 | 4.0508 | 4.3232 | 4.3244 | 4.3346 |
| neg_4039 | Xanthine | metab_8772 | HMDB0000292;PW_C000198;MJDBOTE0000403 | C00385 | 151.025 | 1.9659 | neg | M-H | C5H4N4O2 | 33.9 | 0 | -7.4039099 | 69-89-6 | 0.00788226 | 4.9941 | 5.1024 | 5.049 | 5.2177 | 5.3802 | 5.1933 | 5.1902 | 4.8367 | 5.1778 | 5.1034 | 4.9739 | 5.2341 | 4.7628 | 4.7578 | 4.7644 |
| neg_4074 | 2-Cyano-2-(3,4-dihydroxy-benzyl)-thioacetamide | metab_8810 | HMDB0250056 | - | 258.9963 | 1.3186 | neg | M+K-2H | C10H10N2O2S | 0 | 64.4 | 6.11642307 | - | 0.0026038 | 5.5614 | 5.319 | 5.4223 | 5.3147 | 5.0074 | 5.1593 | 5.5963 | 5.4412 | 5.3476 | 5.4836 | 5.2471 | 5.4191 | 5.2452 | 5.2473 | 5.2454 |
| neg_409 | 2-Methoxy-4-vinylphenol | metab_8826 | HMDB0013744 | C17883 | 195.0655 | 6.055 | neg | M-H, M+FA-H | C9H10O2 | 0 | 51.9 | -5.2634666 | 7786-61-0 | 0.00249455 | 4.955 | 4.7719 | 5.4772 | 5.399 | 4.5046 | 5.2627 | 5.4274 | 5.6295 | 4.9055 | 4.5985 | 5.2522 | 4.706 | 4.9581 | 4.9565 | 4.956 |
| neg_410 | Bisdethiobis(methylthio)gliotoxin | metab_8838 | HMDB0249258 | - | 401.0872 | 6.0471 | neg | M-H2O-H, M+FA-H | C15H20N2O4S2 | 0 | 50.1 | 7.04513643 | - | 0.00346304 | 6.0869 | 6.0766 | 6.1816 | 6.1166 | 6.0847 | 6.1131 | 6.1081 | 6.1765 | 6.0672 | 6.2789 | 6.0635 | 6.0596 | 6.0801 | 6.0818 | 6.0831 |
| neg_4126 | Ascorbic acid 2-sulfate | metab_8866 | HMDB0060649 | - | 254.9812 | 1.0835 | neg | M-H | C6H8O9S | 0 | 49.2 | -1.7963007 | 37627-95-5 | 0.00949309 | 4.7172 | 4.9941 | 4.8895 | 5.0115 | 4.4527 | 4.5165 | 4.9638 | 5.0156 | 4.1854 | 4.9439 | 4.7623 | 4.7446 | 5.0937 | 5.0995 | 5.1017 |
| neg_4130 | Formiminoglutamic acid | metab_8871 | HMDB0255140;HMDB0000854;PW_C000680 | C00439 | 173.0558 | 1.0757 | neg | M-H | C6H10N2O4 | 0 | 35.1 | -5.6289784 | 816-90-0 | 0.02535493 | 3.7204 | 4.8049 | 4.4642 | 4.6899 | 4.3554 | 4.6382 | 4.2152 | 3.8162 | 3.8747 | 3.0394 | 4.8941 | 4.322 | 4.3751 | 4.3945 | 4.394 |
| neg_4142 | N2-Acetyl-L-ornithine | metab_8884 | HMDB0003357 | C00437 | 173.0922 | 1.0288 | neg | M-H | C7H14N2O3 | 74.7 | 0 | -5.6930093 | 8/9/05 | 0.02046631 | 5.016 | 5.4734 | 5.2468 | 5.3284 | 5.3544 | 5.1994 | 5.6433 | 5.1288 | 5.4782 | 5.4466 | 5.2968 | 5.4519 | 5.1303 | 5.1276 | 5.144 |
| neg_4189 | Citrulline | metab_8934 | PW_C000723;HMDB0000904;MJDBOTE0000481 | C00327 | 174.0874 | 0.9739 | neg | M-H | C6H13N3O3 | 89.7 | 0 | -5.6886582 | 372-75-8 | 0.00440033 | 5.5363 | 5.5968 | 5.4747 | 5.6049 | 5.2841 | 5.1943 | 5.7304 | 5.4879 | 5.2497 | 5.6268 | 5.1376 | 5.4507 | 5.2269 | 5.2297 | 5.2306 |
| neg_4212 | L-Glutamine | metab_8961 | PW_C000500;HMDB0003423;HMDB0000641;MJDBOTE0000775 | C00064 | 145.0607 | 0.9114 | neg | M-H | C5H10N2O3 | 84.6 | 0 | -7.8791269 | 56-85-9;5959-95-5 | 0.00397151 | 5.6771 | 5.6291 | 5.572 | 5.5414 | 5.664 | 5.7076 | 5.6647 | 5.5964 | 5.5483 | 5.6825 | 5.739 | 5.7659 | 5.6309 | 5.629 | 5.6325 |
| neg_4216 | L-Histidine | metab_8964 | PW_C000116;HMDB0000177;MJDBOTE0000427 | C00135 | 154.0611 | 0.8958 | neg | M-H | C6H9N3O2 | 64.9 | 0 | -7.2474024 | 71-00-1 | 0.01736312 | 5.4805 | 5.5015 | 5.4467 | 5.388 | 5.4818 | 5.4496 | 5.5398 | 5.317 | 5.3451 | 5.3713 | 5.4717 | 5.6596 | 5.3824 | 5.3844 | 5.3963 |
| neg_422 | Gemfibrozil 1-O-beta-Glucuronide | metab_8968 | HMDB0247652 | - | 425.1814 | 6.0863 | neg | M-H, M+Na-2H | C21H30O9 | 0 | 58 | -0.6260355 | - | 0.00769986 | 5.1314 | 5.6991 | 5.3919 | 5.5188 | 5.9351 | 5.8555 | 5.5497 | 5.3128 | 5.7603 | 5.1811 | 5.9368 | 6.0328 | 5.59 | 5.5844 | 5.584 |
| neg_434 | (2E)-Oct-2-enedioylcarnitine | metab_9099 | HMDB0241710 | - | 314.1606 | 6.1177 | neg | M-H, M+Na-2H | C15H25NO6 | 0 | 44.9 | -1.0344489 | - | 0.01243317 | 4.2765 | 5.2318 | 5.186 | 5.1539 | 5.4048 | 5.0598 | 4.8353 | 4.837 | 5.0253 | 5.493 | 5.1297 | 5.1017 | 5.9609 | 5.9518 | 5.9513 |
| neg_438 | Lucidone B | metab_9141 | HMDB0035834 | - | 399.2206 | 6.1255 | neg | M-H, M+Cl | C24H32O5 | 0 | 31.7 | 7.1774487 | 97653-93-5 | 0.00642637 | 6.5368 | 5.7521 | 6.4424 | 5.983 | 5.7693 | 6.4416 | 6.515 | 6.4648 | 6.5693 | 6.0969 | 6.2671 | 5.8628 | 6.0541 | 6.0562 | 6.0507 |
| neg_44 | Glucoobtusifolin | metab_9163 | HMDB0029617 | C10381 | 491.1189 | 6.0236 | neg | M-H, M+Cl, M+FA-H | C22H22O10 | 0 | 44.1 | -1.2964169 | 120163-18-0 | 0.00340774 | 5.677 | 5.6278 | 5.7295 | 5.6663 | 5.6102 | 5.6369 | 5.6531 | 5.7305 | 5.6087 | 5.8774 | 5.5579 | 5.5943 | 5.6213 | 5.6234 | 5.6205 |
| neg_441 | Norecasantalic acid | metab_9175 | HMDB0036817 | - | 225.1126 | 6.0785 | neg | M-H, M+FA-H | C11H16O2 | 0 | 31.1 | -3.4829973 | 59300-52-6 | 0.0016156 | 4.7109 | 4.8244 | 4.6455 | 4.838 | 4.8924 | 4.771 | 4.82 | 4.7909 | 4.8791 | 4.7619 | 4.8736 | 4.9331 | 4.7909 | 4.79 | 4.7895 |
| neg_446 | Sativic acid | metab_9230 | HMDB0302836;LMFA02000148 | - | 347.2434 | 6.1568 | neg | M-H, M+K-2H | C18H36O6 | 0 | 63.3 | -1.5602232 | - | 0.02149153 | 3.6476 | 3.9795 | 4.1005 | 4.522 | 4.2281 | 3.8397 | 4.4833 | 3.927 | 4.3877 | 2.4702 | 3.9878 | 4.5102 | 4.9008 | 4.9026 | 4.8855 |
| neg_4478 | Deoxyribose | metab_9250 | HMDB0245099;HMDB0003224 | C01801 | 133.0494 | 0.0182 | neg | M-H | C5H10O4 | 49.9 | 0 | -9.2821888 | 533-67-5 | 0.00880696 | 5.0277 | 5.0243 | 5.0538 | 5.0839 | 5.0586 | 5.0833 | 5.0784 | 5.0729 | 5.0466 | 5.1429 | 5.0711 | 5.0762 | 5.064 | 5.0709 | 5.0704 |
| neg_45 | Decanedioic acid | metab_9262 | LMFA01170006;HMDB0000792 | C08277 | 201.1124 | 6.0393 | neg | M-H, M+Na-2H, M-H2O-H | C10H18O4 | 41.8 | 0 | -3.9216667 | 111-20-6 | 0.00788718 | 5.9379 | 5.9745 | 5.9019 | 5.9516 | 6.0508 | 5.8332 | 5.9407 | 5.769 | 5.9853 | 5.7717 | 6.0575 | 6.0593 | 6.0695 | 6.0756 | 6.0751 |
| neg_452 | 10-Hydroxydecanoic acid | metab_9265 | HMDB0244272 | C02774 | 375.2748 | 6.2038 | neg | 2M-H, 2M+Hac-H | C10H20O3 | 0 | 49 | -1.2141186 | 1679-53-4 | 0.05590409 | 5.3152 | 5.9412 | 5.713 | 5.558 | 5.9852 | 5.7997 | 5.7151 | 5.3884 | 5.9846 | 5.2593 | 5.9108 | 5.7341 | 5.9995 | 5.9584 | 5.9578 |
| neg_456 | 2,3-dinor Prostaglandin E1 | metab_9269 | - | - | 325.2015 | 6.2116 | neg | M-H2O-H, M-H | C18H30O5 | 65.2 | 0 | -1.6632578 | - | 0.00342397 | 4.5516 | 4.7935 | 4.5897 | 4.6183 | 4.8225 | 4.6224 | 4.7341 | 4.4093 | 4.7893 | 4.752 | 4.6636 | 4.7019 | 5.036 | 5.0368 | 5.034 |
| neg_460 | 10-hydroxy-11S,12S-epoxy-5Z,8Z,14Z-eicosatrienoic acid | metab_9274 | HMDB0062479;LMFA03090003 | C14810 | 381.231 | 6.2195 | neg | M-H, M+FA-H | C20H32O4 | 0 | 56.4 | 8.15286548 | - | 0.00570064 | 5.5124 | 5.7641 | 5.6641 | 5.5712 | 5.4475 | 5.7391 | 5.7893 | 5.4424 | 5.6856 | 5.2355 | 5.6663 | 5.6181 | 5.6862 | 5.6882 | 5.6833 |
| neg_464 | L-Monomenthyl glutarate | metab_9278 | HMDB0303264 | - | 269.1755 | 6.2351 | neg | M-H, 2M+FA-H | C15H26O4 | 0 | 57.8 | -1.2686207 | - | 0.0141975 | 5.0292 | 5.1609 | 5.0451 | 5.0938 | 5.0805 | 4.9419 | 5.1359 | 4.9521 | 5.1497 | 5.0686 | 5.1104 | 5.1016 | 5.011 | 5.0224 | 5.0128 |
| neg_465 | Tetradecanedioic acid | metab_9279 | HMDB0000872;LMFA01170018 | C11002 | 257.1754 | 6.2351 | neg | M-H, M+Na-2H | C14H26O4 | 67.7 | 0 | -1.5620826 | 821-38-5 | 0.00681572 | 5.8756 | 5.8936 | 5.8501 | 6.0209 | 5.8607 | 5.6631 | 5.9094 | 5.6184 | 5.8458 | 5.8789 | 5.7474 | 5.7656 | 5.8214 | 5.8271 | 5.823 |
| neg_466 | Tanacetol A | metab_9280 | HMDB0035722 | - | 293.1755 | 6.2429 | neg | M-H, M+Cl | C17H26O4 | 0 | 51.4 | -1.0949925 | 86778-06-5 | 0.00457901 | 5.7918 | 5.7611 | 5.7903 | 5.8083 | 5.7898 | 5.8323 | 5.8158 | 5.8272 | 5.7929 | 5.8782 | 5.7948 | 5.801 | 5.7663 | 5.7691 | 5.7701 |
| neg_478 | Xi-8-Hydroxyhexadecanedioic acid | metab_9293 | HMDB0037831 | - | 283.1911 | 6.2903 | neg | M-H2O-H, M+K-2H | C16H30O5 | 0 | 40.3 | -1.3834094 | - | 0.00571319 | 5.3611 | 5.3475 | 5.3847 | 5.6025 | 5.1994 | 5.0122 | 5.5503 | 5.3111 | 5.2513 | 5.7321 | 5.1999 | 5.264 | 5.3675 | 5.3723 | 5.369 |
| neg_483 | Glyuranolide | metab_9299 | HMDB0038745 | - | 511.302 | 6.2903 | neg | M-H, M+Na-2H | C31H44O6 | 0 | 35.6 | -8.7183371 | 123914-44-3 | 0.01365306 | 4.041 | 5.7196 | 5.8521 | 5.468 | 6.2104 | 6.1822 | 4.5991 | 5.8574 | 6.0866 | 5.334 | 6.0408 | 6.0426 | 5.8654 | 5.8674 | 5.8563 |
| neg_494 | Prostaglandin F1a | metab_9311 | HMDB0002685 | C06475 | 337.2381 | 6.3536 | neg | M-H2O-H, M-H | C20H36O5 | 0 | 66.4 | -0.8833989 | 745-62-0 | 0.00928064 | 4.9135 | 5.1198 | 5.0602 | 5.0142 | 4.9228 | 4.9715 | 5.1739 | 4.7933 | 5.0005 | 5.0272 | 4.9688 | 5.0031 | 5.2042 | 5.1975 | 5.1971 |
| neg_500 | Ruscogenin | metab_9318 | HMDB0257364;LMST01080038 | C08909 | 429.3002 | 6.3927 | neg | M-H, M+FA-H | C27H42O4 | 0 | 91.9 | -1.9266343 | 472-11-7 | 0.02076681 | 5.1032 | 4.5639 | 4.5779 | 4.7192 | 4.4427 | 4.2888 | 4.6176 | 4.7989 | 4.4486 | 4.7432 | 4.5148 | 4.7538 | 4.9319 | 4.9339 | 4.9173 |
| neg_503 | LysoPC(18:3(9Z,12Z,15Z)/0:0) | metab_9321 | HMDB0010388 | - | 562.3144 | 6.4083 | neg | M+Cl, M+FA-H | C26H48NO7P | 0 | 81.5 | -1.1991865 | - | 0.00703978 | 5.7144 | 6.1174 | 5.9216 | 5.9237 | 5.9588 | 5.956 | 5.927 | 5.7216 | 6.0074 | 5.8162 | 5.8453 | 5.9009 | 6.0275 | 6.0295 | 6.0235 |
| neg_506 | LysoPC(22:6(4Z,7Z,10Z,13Z,16Z,19Z)/0:0) | metab_9324 | HMDB0010404 | C04230 | 612.3302 | 6.424 | neg | M+Cl, M+FA-H | C30H50NO7P | 0 | 83.8 | -0.814834 | 162440-05-3 | 0.01121851 | 5.8927 | 5.8337 | 5.7744 | 5.681 | 5.976 | 5.8086 | 5.9238 | 5.7278 | 5.693 | 5.7032 | 6.0239 | 6.0588 | 5.7013 | 5.7034 | 5.6941 |
| neg_51 | 3a,7b,12a-Trihydroxyoxocholanyl-Glycine | metab_9328 | LMST05030007;HMDB0000331 | - | 464.301 | 6.1803 | neg | M-H, 2M-H, M-H2O-H | C26H43NO6 | 0 | 47.3 | -1.6970641 | 68753-51-5 | 0.00569134 | 5.7594 | 7.4825 | 7.2566 | 6.9064 | 7.8124 | 7.8198 | 6.7427 | 6.8632 | 7.7879 | 7.1413 | 7.6956 | 7.3611 | 7.4751 | 7.4799 | 7.4767 |
| neg_514 | LysoPC(20:4(5Z,8Z,11Z,14Z)/0:0) | metab_9333 | HMDB0010395 | C04230 | 588.3303 | 6.4638 | neg | M+Cl, M+FA-H | C28H50NO7P | 0 | 78.9 | -0.7170626 | 60701-99-7 | 0.00294062 | 6.9885 | 6.8955 | 6.8364 | 6.8451 | 7.0379 | 6.956 | 6.995 | 6.8638 | 6.8531 | 6.9275 | 6.9547 | 7.0299 | 6.8347 | 6.8328 | 6.8323 |
| neg_519 | 1-Linoleoyl-sn-Glycero-3-Phosphocholine | metab_9338 | HMDB0010386;LMGP01050137 | C04100 | 564.3301 | 6.5114 | neg | M+Cl, M+FA-H | C26H50NO7P | 68 | 0 | -1.1834147 | 22252-07-9 | 0.00551835 | 7.1107 | 7.174 | 7.1781 | 7.0923 | 7.1923 | 7.2026 | 7.1601 | 7.044 | 7.1655 | 7.0797 | 7.1353 | 7.182 | 7.1532 | 7.1493 | 7.1489 |
| neg_525 | LysoPC(20:3(5Z,8Z,11Z)/0:0) | metab_9345 | HMDB0010393 | - | 590.3459 | 6.5585 | neg | M+Cl, M+FA-H | C28H52NO7P | 0 | 84.3 | -0.7315925 | 1199257-41-4 | 0.00506176 | 5.8122 | 6.2149 | 6.2043 | 6.1973 | 6.1523 | 6.2693 | 6.0986 | 6.1677 | 6.197 | 6.1354 | 5.8812 | 6.0424 | 6.3583 | 6.3621 | 6.3583 |
| neg_53 | 3-Dehydrocholic Acid | metab_9350 | HMDB0000502;HMDB0304121 | - | 405.2641 | 6.1646 | neg | M-H, M+Cl, M+FA-H | C24H38O5 | 64 | 0 | -1.4462313 | 2304-89-4 | 0.0121966 | 5.0595 | 5.3472 | 5.3925 | 5.6683 | 5.0535 | 5.5587 | 5.8587 | 5.9191 | 5.322 | 4.7957 | 5.3971 | 5.5093 | 6.3259 | 6.3358 | 6.3275 |
| neg_530 | 5alpha-Pregnan-20alpha-ol-3-one | metab_9351 | HMDB0060408 | C18041 | 339.2324 | 6.5585 | neg | M+Na-2H, M+FA-H | C21H34O2 | 0 | 43 | 5.81734048 | 516-59-6 | 0.01418465 | 5.5935 | 5.6222 | 5.6546 | 5.3268 | 5.7358 | 5.4651 | 5.1481 | 6.0987 | 5.5984 | 5.1326 | 5.4193 | 5.5709 | 6.2776 | 6.2886 | 6.2881 |
| neg_54 | (6E,8R,10Z)-8-hydroxy-3-oxohexadecadienoic acid | metab_9361 | HMDB0062365 | - | 281.1754 | 6.2508 | neg | M-H, M+Na-2H, M-H2O-H | C16H26O4 | 0 | 44 | -1.4992093 | 2190485-67-5 | 0.05793276 | 4.7086 | 4.5733 | 4.7582 | 4.8763 | 4.6483 | 4.5503 | 4.7985 | 4.6982 | 4.5315 | 5.0837 | 4.5481 | 4.6836 | 4.6587 | 4.703 | 4.703 |
| neg_542 | 1-Palmitoylphosphatidylcholine | metab_9364 | HMDB0010382;HMDB0256091 | C04230 | 540.33 | 6.6687 | neg | M+Cl, M+FA-H | C24H50NO7P | 0 | 67 | -1.3104286 | 17364-16-8 | 0.00326698 | 7.4711 | 7.3238 | 7.3345 | 7.3584 | 7.317 | 7.345 | 7.3614 | 7.3536 | 7.3398 | 7.3106 | 7.3328 | 7.3108 | 7.303 | 7.3018 | 7.3046 |
| neg_553 | L-Menthyl acetoacetate | metab_9376 | HMDB0032369 | - | 221.1541 | 6.2429 | neg | M-H2O-H, M-H | C14H24O3 | 0 | 53.1 | -2.4207148 | 59557-05-0 | 0.00721396 | 4.9503 | 4.9248 | 4.956 | 4.9821 | 4.971 | 4.9345 | 4.9673 | 4.9543 | 4.9509 | 5.0549 | 4.9226 | 4.9486 | 4.9407 | 4.9467 | 4.9422 |
| neg_56 | 16-Hydroxy-10-oxohexadecanoic acid | metab_9383 | HMDB0041287 | - | 285.2068 | 6.3457 | neg | M-H, M+Na-2H, M-H2O-H | C16H30O4 | 0 | 56.5 | -1.2965001 | 53833-25-3 | 0.00699373 | 6.0648 | 5.9462 | 5.9787 | 6.2359 | 5.5593 | 5.5137 | 6.2613 | 6.0241 | 5.6654 | 6.3578 | 5.6451 | 5.7117 | 5.9165 | 5.9189 | 5.9129 |
| neg_57 | 12(R)-HETE | metab_9394 | PW_C040453 | C14822 | 319.2273 | 6.4083 | neg | M-H, M+Cl, M-H2O-H | C20H32O3 | 72.2 | 0 | -1.6569471 | - | 0.00547717 | 6.7244 | 6.3925 | 6.1034 | 6.7467 | 5.9352 | 6.2153 | 6.5297 | 6.6099 | 6.0311 | 6.0978 | 5.5245 | 6.4005 | 6.4265 | 6.4226 | 6.4221 |
| neg_58 | 9(S)-HpODE | metab_9405 | HMDB0006940 | C14827 | 311.2224 | 6.3849 | neg | M-H, M+Cl, M-H2O-H | C18H32O4 | 61.8 | 0 | -1.1395794 | 29774-12-7 | 0.00353962 | 6.4231 | 6.5098 | 6.6185 | 6.7052 | 6.2797 | 6.0527 | 6.6754 | 6.4045 | 6.3741 | 6.6587 | 6.3037 | 6.3241 | 6.3363 | 6.3384 | 6.3354 |
| neg_7 | Allocholic acid | metab_9536 | LMST04010092;HMDB0000505 | C17737 | 407.2798 | 6.2749 | neg | M-H, 2M-H, M+FA-H, M+Cl | C24H40O5 | 55.2 | 0 | -1.3328032 | 2464-18-8 | 0.00235373 | 6.4116 | 7.0678 | 7.0579 | 6.6218 | 7.082 | 7.311 | 7.1861 | 6.7799 | 7.3337 | 6.5855 | 7.0119 | 7.0815 | 7.3779 | 7.3799 | 7.3788 |
| neg_769 | (+)-threo-2-Amino-3,4-dihydroxybutanoic acid | metab_9609 | HMDB0029389;LMFA01050439 | - | 116.034 | 1.1386 | neg | M-H2O-H | C4H9NO4 | 0 | 39.5 | -9.5787183 | 21768-44-5 | 0.01202567 | 5.0556 | 4.623 | 4.8208 | 5.0918 | 4.6356 | 4.6297 | 4.8782 | 4.6894 | 4.6134 | 5.2819 | 4.5958 | 4.6772 | 4.775 | 4.7769 | 4.7848 |
| neg_778 | Uric Acid | metab_9619 | HMDB0000289;PW_C000195;MJDBOTE0001097 | C00366 | 167.02 | 1.1699 | neg | M-H | C5H4N4O3 | 89 | 0 | -6.2840809 | 69-93-2 | 0.01222582 | 5.4711 | 5.4932 | 5.362 | 5.5882 | 5.599 | 5.4862 | 5.425 | 5.5051 | 5.8502 | 5.6654 | 5.2629 | 5.7912 | 6.5152 | 6.5173 | 6.5252 |
| neg_784 | Citric Acid | metab_9625 | HMDB0000094;PW_C000063;MJDBNL00000139 | C00158 | 191.0188 | 1.2014 | neg | M-H | C6H8O7 | 91.4 | 0 | -4.6558771 | 77-92-9 | 0.00256077 | 5.3179 | 6.145 | 6.3311 | 6.2148 | 6.1827 | 6.3019 | 6.2305 | 5.4552 | 5.7067 | 5.2536 | 6.4335 | 6.0756 | 6.2266 | 6.2263 | 6.2283 |
| neg_795 | Trans-Aconitic Acid | metab_9637 | HMDB0000958;MJDBOTE0001106 | C02341 | 173.0081 | 1.3341 | neg | M-H | C6H6O6 | 40.4 | 0 | -5.9129815 | 4023-65-8 | 0.00605256 | 3.6708 | 4.4969 | 4.3554 | 4.1916 | 4.7611 | 4.464 | 4.4254 | 3.4024 | 4.4517 | 2.6102 | 4.6782 | 4.2776 | 4.8883 | 4.8927 | 4.893 |
| neg_8 | P-CHLOROPHENYLALANINE | metab_9642 | HMDB0033261 | - | 198.0319 | 3.8825 | neg | M-H, M+Cl, M+FA-H, 2M-H | C9H10ClNO2 | 67.5 | 0 | -4.2190538 | 101365-03-1 | 0.01573413 | 7.3409 | 7.2704 | 7.4043 | 7.3934 | 7.3629 | 7.39 | 7.3056 | 7.3783 | 7.3348 | 7.433 | 7.3364 | 7.3492 | 7.3348 | 7.3367 | 7.3474 |
| neg_815 | Pseudouridine | metab_9660 | HMDB0000767 | C02067 | 279.0385 | 1.8587 | neg | M+Cl | C9H12N2O6 | 69.7 | 0 | -1.8319795 | 1445-07-4 | 0.00396973 | 5.6701 | 5.7238 | 5.5953 | 5.4725 | 5.8377 | 5.6229 | 5.6877 | 5.5052 | 5.6639 | 5.5702 | 5.6372 | 5.8512 | 5.5425 | 5.5445 | 5.5411 |
| neg_817 | L-Glutamic gamma-semialdehyde | metab_9662 | HMDB0002104;PW_C001415 | C01165 | 130.0497 | 1.9047 | neg | M-H | C5H9NO3 | 0 | 50.6 | -9.5695336 | 496-92-4 | 0.0107387 | 5.1388 | 5.2399 | 5.2778 | 5.2585 | 5.487 | 5.5108 | 5.2689 | 5.2383 | 5.4578 | 5.2976 | 5.2952 | 5.5464 | 5.3857 | 5.3873 | 5.3944 |
| neg_819 | Threonic acid | metab_9664 | HMDB0000943 | C01620 | 117.018 | 1.9659 | neg | M-H2O-H | C4H8O5 | 0 | 32.7 | -9.5787712 | 7306-96-9 | 0.00269762 | 5.9768 | 6.2967 | 6.0728 | 6.2562 | 6.0241 | 6.107 | 6.0511 | 6.0181 | 6.0911 | 6.0071 | 6.1346 | 6.1669 | 6.0414 | 6.0406 | 6.0429 |
| neg_829 | Norleucine | metab_9675 | HMDB0001645;HMDB0251526 | C01933 | 130.0861 | 2.2421 | neg | M-H | C6H13NO2 | 31.8 | 0 | -9.5152473 | 327-57-1 | 0.01261511 | 5.5132 | 5.5598 | 5.4951 | 5.4271 | 5.424 | 5.3837 | 5.528 | 5.3628 | 5.3334 | 5.4615 | 5.2996 | 5.4988 | 5.4214 | 5.4311 | 5.4307 |
| neg_830 | L-Tyrosine | metab_9677 | HMDB0000158;PW_C000103;MJDBOTE0000356 | C00082 | 180.0656 | 2.2421 | neg | M-H | C9H11NO3 | 78.8 | 0 | -5.3938423 | 60-18-4 | 0.0049397 | 5.7202 | 5.8913 | 5.8206 | 5.824 | 5.8042 | 5.7897 | 5.9141 | 5.7 | 5.6802 | 5.8094 | 5.8047 | 5.8532 | 5.7668 | 5.7678 | 5.7637 |
| neg_862 | Parachlorophenol | metab_9712 | HMDB0246398 | C02124 | 126.9959 | 2.734 | neg | M-H | C6H5ClO | 31 | 0 | 2.17012322 | 106-48-9 | 0.03390049 | 3.4338 | 3.2888 | 3.8774 | 2.7191 | 4.6519 | 4.437 | 4.1328 | 3.0566 | 4.4507 | 3.8436 | 4.1645 | 4.3495 | 3.742 | 3.7681 | 3.7437 |
| neg_868 | 3-Sulfobenzoic acid | metab_9718 | HMDB0245984 | - | 246.9913 | 3.0377 | neg | M+FA-H | C7H6O5S | 0 | 69 | -2.5875646 | - | 0.01523953 | 2.6714 | 3.5865 | 2.8225 | 2.7221 | 3.5889 | 2.7838 | 3.1239 | 2.6914 | 3.3424 | 2.7358 | 3.2694 | 3.4857 | 4.4744 | 4.4639 | 4.4763 |
| neg_874 | Guanosine | metab_9725 | HMDB0000133;PW_C000087;MJDBOTE0000372 | C00387 | 282.084 | 3.1504 | neg | M-H | C10H13N5O5 | 95.2 | 0 | -1.4753485 | 118-00-3 | 0.00322576 | 6.2927 | 6.1595 | 6.1712 | 6.0417 | 6.0785 | 5.9107 | 6.2288 | 6.0958 | 6.1903 | 6.1603 | 6.1899 | 6.0975 | 5.7903 | 5.7924 | 5.793 |
| neg_876 | 3-O-Methyl-L-DOPA | metab_9727 | - | - | 210.0764 | 3.226 | neg | M-H | C10H13NO4 | 32.7 | 0 | -3.5859862 | - | 0.01323937 | 4.5287 | 3.9733 | 3.7532 | 4.0275 | 3.8597 | 4.0016 | 4.0353 | 4.0814 | 4.1351 | 3.9855 | 4.0282 | 4.0978 | 4.2093 | 4.2116 | 4.2202 |
| neg_882 | Isopropylmaleic acid | metab_9734 | HMDB0012241 | C02631 | 203.0553 | 3.271 | neg | M+FA-H | C7H10O4 | 0 | 47.7 | -5.190219 | 44976-69-4 | 0.01584764 | 4.2337 | 4.3678 | 4.4044 | 4.354 | 4.5096 | 4.405 | 4.7364 | 4.1447 | 4.6327 | 4.2256 | 4.5246 | 4.3805 | 4.2303 | 4.2324 | 4.2431 |
| neg_883 | 2-Ethylhydracrylic acid | metab_9735 | HMDB0000396 | - | 281.1237 | 3.271 | neg | 2M+FA-H | C5H10O3 | 0 | 32.2 | -1.9236008 | 4374-62-3 | 0.03520941 | 4.2422 | 5.0828 | 4.8703 | 4.8007 | 4.9866 | 4.9619 | 4.8972 | 4.3042 | 4.9773 | 4.7772 | 4.8742 | 4.9768 | 5.0265 | 5.0535 | 5.0531 |
| neg_893 | 3-Hydroxyindolin-2-one-sulfate | metab_9745 | HMDB0304928 | - | 227.9966 | 3.3085 | neg | M-H | C8H7NO5S | 0 | 66.6 | -2.8174595 | - | 0.00409835 | 5.3854 | 5.4332 | 5.4629 | 5.4931 | 5.3786 | 5.416 | 5.4381 | 5.4605 | 5.38 | 5.2101 | 5.2313 | 5.4158 | 5.2316 | 5.2351 | 5.2331 |
| neg_894 | Gamma-Glutamylvaline | metab_9746 | HMDB0011172 | - | 245.1138 | 3.3189 | neg | M-H | C10H18N2O5 | 0 | 65.8 | -2.0925444 | 2746-34-1 | 0.00913182 | 5.217 | 5.3144 | 5.3003 | 5.1574 | 5.2203 | 5.1442 | 5.2701 | 5.2109 | 5.0683 | 5.2008 | 5.0351 | 5.2097 | 5.2031 | 5.2102 | 5.2097 |
| neg_903 | 1-(1,2,3,4,5-Pentahydroxypent-1-yl)-1,2,3,4-tetrahydro-beta-carboline-3-carboxylate | metab_9757 | HMDB0012492 | - | 365.1347 | 3.3437 | neg | M-H | C17H22N2O7 | 0 | 46.9 | -2.1018394 | - | 0.01981121 | 4.8613 | 4.7825 | 4.8907 | 4.7985 | 4.7848 | 4.6954 | 4.7839 | 4.9295 | 4.739 | 4.9031 | 4.6406 | 4.89 | 4.8135 | 4.799 | 4.7985 |
| neg_904 | 3-Methoxytyrosine | metab_9758 | HMDB0001434;HMDB0060747 | - | 232.0585 | 3.3468 | neg | M+Na-2H | C10H13NO4 | 0 | 38.5 | -2.8464607 | 7636-26-2 | 0.00971629 | 5.3978 | 5.4281 | 5.3958 | 5.2904 | 5.3365 | 5.3763 | 5.4254 | 5.3865 | 5.3083 | 5.3891 | 5.2782 | 5.4427 | 5.3888 | 5.3908 | 5.3969 |
| neg_906 | Carbohydrazide | metab_9760 | HMDB0249636 | - | 125.0232 | 3.3571 | neg | M+Cl | CH6N4O | 0 | 37.7 | -4.3687672 | - | 0.01919672 | 4.5069 | 4.797 | 4.7133 | 4.8548 | 4.6306 | 4.6378 | 4.596 | 4.6791 | 4.6915 | 4.6445 | 4.7222 | 4.6753 | 4.6857 | 4.6879 | 4.701 |
| neg_920 | Cyclopentenyl cytosine | metab_9773 | HMDB0250668 | - | 284.0884 | 3.4032 | neg | M+FA-H | C10H13N3O4 | 0 | 30.2 | -1.784405 | - | 0.00411448 | 5.0796 | 4.5051 | 4.817 | 5.0277 | 4.804 | 4.9553 | 5.166 | 5.2968 | 5.4083 | 4.8816 | 5.1539 | 5.0829 | 4.8591 | 4.8562 | 4.8558 |
| neg_925 | N-(2-Furoyl)glycine | metab_9777 | HMDB0000439 | - | 168.0292 | 3.437 | neg | M-H | C7H7NO4 | 54.7 | 0 | -5.964516 | 5657-19-2 | 0.01140629 | 2.4958 | 3.649 | 3.4791 | 3.3633 | 4.545 | 4.6136 | 3.5354 | 3.2317 | 4.4923 | 3.1459 | 4.4914 | 4.5589 | 4.0628 | 4.0721 | 4.0704 |
| neg_933 | 7-Deaza-2'-deoxyguanosine | metab_9785 | HMDB0247243 | - | 265.0937 | 3.4803 | neg | M-H | C11H14N4O4 | 0 | 64.7 | -1.993453 | - | 0.02410021 | 3.0864 | 3.8827 | 3.6704 | 3.6538 | 3.696 | 3.5983 | 3.1351 | 3.4654 | 3.616 | 3.2677 | 4.0546 | 3.6518 | 4.1205 | 4.1392 | 4.122 |
| neg_941 | Pantothenic Acid | metab_9793 | HMDB0000210;HMDB0250782;PW_C000136;MJDBOTE0000845 | C00864 | 218.1027 | 3.5153 | neg | M-H | C9H17NO5 | 82.2 | 0 | -3.27599 | 79-83-4 | 0.0054349 | 5.1212 | 5.2862 | 5.3515 | 5.1511 | 5.0488 | 5.1709 | 5.1897 | 5.1487 | 5.2095 | 4.9657 | 5.3049 | 5.2546 | 5.2562 | 5.2532 | 5.2579 |
| neg_951 | D-Digitoxose | metab_9804 | HMDB0251279 | C21045 | 295.1393 | 3.5744 | neg | 2M-H | C6H12O4 | 0 | 49.3 | -1.8043468 | - | 0.01092167 | 4.5034 | 5.0502 | 4.8471 | 4.7676 | 5.106 | 5.1182 | 4.8348 | 4.5407 | 5.0565 | 4.8479 | 4.9808 | 5.2536 | 5.0665 | 5.0685 | 5.0755 |
| neg_970 | Gabazine | metab_9823 | HMDB0252568 | C13796 | 332.1247 | 3.7316 | neg | M+FA-H | C15H17N3O3 | 0 | 50.4 | -1.6975235 | - | 0.0118163 | 5.2164 | 4.6823 | 4.9931 | 4.6826 | 4.9774 | 4.9052 | 4.9349 | 4.9288 | 4.6967 | 4.8919 | 4.8286 | 5.0331 | 4.8093 | 4.8012 | 4.8108 |
| neg_977 | 3-deoxy-D-arabino-heptulosonate-7-phosphate | metab_9830 | HMDB0304124 | C04691 | 287.0162 | 3.7371 | neg | M-H | C7H13O10P | 0 | 57.7 | -4.1743947 | - | 0.0187164 | 3.6704 | 4.688 | 4.529 | 4.7691 | 4.5772 | 3.8101 | 3.6425 | 4.2895 | 4.3823 | 3.6583 | 4.2318 | 4.2005 | 4.062 | 4.0487 | 4.0636 |
| neg_987 | Alpha-Hydroxyhippuric Acid | metab_9841 | HMDB0002404 | - | 194.045 | 3.7769 | neg | M-H | C9H9NO4 | 50.8 | 0 | -4.3239105 | 16555-77-4 | 0.00692925 | 4.3645 | 4.2883 | 4.2516 | 4.3133 | 4.4955 | 4.954 | 4.4327 | 4.2726 | 4.5045 | 4.0722 | 4.4847 | 4.5826 | 4.8317 | 4.8267 | 4.8262 |
| neg_988 | Acetaminophen | metab_9842 | PW_C001233;HMDB0001859 | C06804 | 150.0549 | 3.7833 | neg | M-H | C8H9NO2 | 0 | 30.5 | -7.5957149 | 103-90-2 | 0.00785463 | 3.3989 | 3.5471 | 3.563 | 3.4884 | 3.6111 | 3.9766 | 3.6055 | 3.4924 | 3.7031 | 3.4963 | 3.5469 | 3.6654 | 3.9092 | 3.9159 | 3.9137 |
| neg_995 | Glycyl-Phenylalanine | metab_9849 | HMDB0028848 | - | 221.0925 | 3.8074 | neg | M-H | C11H14N2O3 | 0 | 40.4 | -3.0951061 | - | 0.06132351 | 4.724 | 4.5598 | 4.612 | 4.6117 | 4.1145 | 4.3508 | 4.717 | 4.6679 | 4.4924 | 4.5226 | 4.5617 | 4.5195 | 4.2241 | 4.2715 | 4.2707 |
| neg_998 | L-Rhamnulose | metab_9852 | HMDB0010207 | C00861 | 185.042 | 3.8178 | neg | M+Na-2H | C6H12O5 | 0 | 36.3 | -6.7795792 | 470-21-3 | 0.02971117 | 4.3115 | 3.7752 | 4.3992 | 4.0118 | 4.2011 | 4.3394 | 3.4665 | 4.0375 | 4.4135 | 2.9844 | 4.2824 | 4.1647 | 4.1727 | 4.1956 | 4.1949 |
| neg_1071 | 3-Methyl-2-Oxovaleric Acid | metab_9854 | PW_C000373;HMDB0000491 | C00671 | 129.0545 | 4.1127 | neg | M-H | C6H10O3 | 77.6 | 0 | -9.691902 | 1460-34-0;24809-08-3 | 0.03820573 | 6.4866 | 6.4233 | 6.453 | 6.4697 | 6.3468 | 6.2273 | 6.3493 | 6.3328 | 6.3257 | 6.2178 | 6.1927 | 6.2915 | 6.2994 | 6.3286 | 6.3283 |
| neg_3441 | 4-Hydroxycinnamic acid | metab_9855 | HMDB0002035;HMDB0030677;HMDB0003654 | C00811 | 327.087 | 5.7681 | neg | 2M-H | C9H8O3 | 0 | 36.1 | -1.380561 | 4501-31-9;7400-08-0;501-98-4;501-98-4;20649-40-5 | 0.03637848 | 5.0207 | 5.1893 | 5.0533 | 5.1033 | 4.9515 | 4.8193 | 5.2302 | 5.2239 | 4.8723 | 4.6797 | 5.1117 | 5.1097 | 4.9959 | 4.9692 | 4.9685 |
| neg_1444 | 4-Hydroxycoumarin | metab_9856 | HMDB0003654 | C20414 | 161.0234 | 5.9766 | neg | M-H | C9H6O3 | 70.5 | 0 | -6.5503747 | 20649-40-5;1076-38-6 | 0.03599489 | 4.4279 | 4.6292 | 4.6877 | 4.4219 | 4.5252 | 4.4618 | 4.5976 | 4.594 | 4.4041 | 4.4461 | 4.6426 | 4.5501 | 4.2966 | 4.3243 | 4.2984 |
| neg_2796 | 9,10-Dihydroxystearic acid | metab_9857 | LMFA02000142;HMDB0302281;HMDB0303981 | C19622 | 315.2535 | 6.3614 | neg | M-H | C18H36O4 | 0 | 50.2 | -1.8964025 | 120-87-6 | 0.00123585 | 5.4967 | 5.5525 | 5.4455 | 5.6085 | 5.3999 | 5.2794 | 5.6583 | 5.3999 | 5.3941 | 5.4191 | 5.4124 | 5.4285 | 5.8666 | 5.8677 | 5.8672 |
| neg_1770 | 9,10-Epoxyoctadecanoic acid | metab_9858 | HMDB0247617;LMFA02000326;HMDB0061650 | C19620 | 313.238 | 6.2981 | neg | M-H | C18H34O4 | 0 | 34.9 | -1.2682566 | 154966-80-0 | 0.00180803 | 5.5925 | 5.6087 | 5.5916 | 5.6333 | 5.6921 | 5.5623 | 5.741 | 5.5866 | 5.5341 | 5.582 | 5.6171 | 5.6759 | 5.8863 | 5.8853 | 5.8848 |
| neg_3856 | Acrylic acid | metab_9859 | LMFA01030193;PW_C012827;HMDB0031647 | C00511 | 203.0553 | 3.7371 | neg | 2M+Hac-H | C3H4O2 | 0 | 33.6 | -5.9538983 | 79-10-7;9003-01-4 | 0.05671203 | 3.615 | 4.2222 | 3.7815 | 3.7126 | 4.3876 | 4.0401 | 3.6657 | 3.6774 | 4.1105 | 2.9865 | 4.0865 | 4.0774 | 4.0356 | 4.0788 | 4.0791 |
| neg_1868 | Androstan-3alpha,17beta-diol | metab_9860 | LMST02020052;HMDB0060437 | C03852 | 337.238 | 6.456 | neg | M+FA-H | C19H32O2 | 0 | 85.7 | -1.4183772 | - | 0.04364846 | 4.3227 | 4.6159 | 4.4701 | 4.564 | 4.3766 | 4.2021 | 4.5565 | 4.2832 | 4.5141 | 4.2714 | 4.3052 | 4.3421 | 4.6462 | 4.6792 | 4.6473 |
| neg_396 | Artemisin | metab_9861 | LMPR0103190003;HMDB0248621 | C09538 | 283.0972 | 6.0079 | neg | M+Na-2H, 2M+Hac-H | C15H18O4 | 0 | 50.3 | 7.62907687 | 481-05-0 | 0.0039702 | 5.074 | 4.9685 | 4.851 | 4.8303 | 4.7807 | 4.618 | 5.003 | 4.9326 | 4.7774 | 5.1988 | 4.8884 | 4.9542 | 4.826 | 4.828 | 4.8294 |
| neg_766 | Ascorbic Acid | metab_9862 | PW_C000031;HMDB0000044;MJDBOTE0001568 | C01041 | 175.0238 | 1.123 | neg | M-H | C6H8O6 | 62.3 | 0 | -5.7166287 | 50-81-7 | 0.00940529 | 4.9853 | 4.2981 | 4.9949 | 4.4435 | 4.7827 | 5.0429 | 5.1148 | 5.1099 | 4.9429 | 5.4671 | 4.4579 | 4.472 | 5.0226 | 5.0307 | 5.0257 |
| neg_1684 | Brassinolide | metab_9863 | HMDB0041130;HMDB0034081;LMST01140001 | C08814 | 479.3366 | 6.1959 | neg | M-H | C28H48O6 | 0 | 39.4 | -2.433202 | 72962-43-7;78821-43-9 | 0.03196745 | 5.1252 | 5.0742 | 5.2044 | 5.1404 | 5.0405 | 5.0427 | 5.4214 | 5.0976 | 5.2218 | 4.9093 | 5.1666 | 4.9068 | 4.8419 | 4.8668 | 4.8655 |
| neg_3523 | Caffeic Acid | metab_9864 | HMDB0001964;HMDB0003501 | C01197 | 179.034 | 5.381 | neg | M-H | C9H8O4 | 38.6 | 0 | -5.204107 | 501-16-6;331-39-5;4361-87-9 | 0.03587732 | 3.8706 | 3.8098 | 3.6116 | 3.7659 | 4.3529 | 4.1831 | 3.8822 | 3.824 | 4.1821 | 3.4663 | 4.3625 | 4.4138 | 3.9265 | 3.9313 | 3.9554 |
| neg_603 | D-Apiose | metab_9865 | HMDB0029941 | C01488;C21040 | 171.0265 | 0.751 | neg | M+Na-2H | C5H10O5 | 0 | 34.2 | -6.8693928 | 639-97-4 | 0.01070303 | 4.4174 | 4.4023 | 4.3783 | 4.4507 | 4.5319 | 4.4059 | 4.4503 | 4.5767 | 4.4744 | 4.5522 | 4.4362 | 4.4424 | 4.4573 | 4.4495 | 4.4491 |
| neg_4214 | D-Galactose | metab_9866 | HMDB0250761;PW_C000093;HMDB0003449;HMDB0000143;MJDBOTE0001042 | C00124 | 225.061 | 0.9036 | neg | M+FA-H | C6H12O6 | 61.2 | 0 | -3.5580736 | 3646-73-9;7296-64-2;59-23-4;10257-28-0;59-23-4 | 0.00840192 | 6.0461 | 6.0474 | 6.0678 | 6.065 | 6.0198 | 6.0436 | 6.0018 | 6.054 | 5.956 | 5.9338 | 5.9364 | 6.0459 | 6.0605 | 6.0625 | 6.0676 |
| neg_2533 | Dehydrovomifoliol | metab_9867 | HMDB0036819;HMDB0302822;LMPR0103050009 | C02533 | 443.2467 | 6.9642 | neg | 2M-H | C13H18O3 | 0 | 41.1 | 6.20793815 | 15764-81-5 | 0.08526057 | 4.7927 | 4.8027 | 4.6547 | 4.5876 | 4.4087 | 4.6901 | 4.6106 | 4.538 | 4.897 | 4.8615 | 4.7072 | 4.6888 | 4.3787 | 4.443 | 4.3821 |
| neg_50 | Dodecanedioic Acid | metab_9868 | LMFA01170009;HMDB0000933;HMDB0000623;MJDBOTE0000650 | C16308 | 229.1439 | 6.1568 | neg | M-H, M+Na-2H, M-H2O-H | C12H22O4 | 48.7 | 0 | -2.5732106 | 6402-36-4;693-23-2 | 0.00548175 | 5.9088 | 5.9882 | 5.8276 | 5.9241 | 6.0735 | 5.8176 | 5.949 | 5.7097 | 6.0531 | 5.7693 | 6.0782 | 6.1142 | 5.9832 | 5.9879 | 5.9848 |
| neg_2653 | Hexadecanedioic acid | metab_9869 | HMDB0000712;HMDB0000672;LMFA01170022;HMDB0304377 | C19615 | 267.1962 | 6.582 | neg | M-H2O-H | C16H30O4 | 0 | 65.8 | -1.2314178 | 505-54-4;42150-38-9 | 0.04173103 | 4.396 | 4.1152 | 4.2611 | 4.1957 | 3.4133 | 3.8177 | 4.3624 | 4.1517 | 3.3576 | 4.3345 | 3.5065 | 3.6463 | 4.2309 | 4.2671 | 4.2504 |
| neg_888 | Kynurenine | metab_9870 | HMDB0000684;MJDBOTE0001164 | C00328 | 207.0767 | 3.2897 | neg | M-H | C10H12N2O3 | 84.4 | 0 | -3.7856698 | 2922-83-0;343-65-7 | 0.01172648 | 5.3766 | 5.2853 | 5.1745 | 5.0616 | 5.2832 | 5.045 | 5.2798 | 5.1619 | 5.1025 | 5.0873 | 5.0571 | 5.3621 | 5.2624 | 5.2715 | 5.2711 |
| neg_4121 | L-Glutamic Acid | metab_9871 | HMDB0000148;HMDB0003339;HMDB0060475;PW_C000095;MJDBOTE0000649 | C00025 | 146.0447 | 1.0995 | neg | M-H | C5H9NO4 | 82.7 | 0 | -7.9164028 | 6893-26-1;56-86-0 | 0.01483031 | 5.011 | 4.9393 | 4.9978 | 5.0094 | 5.1303 | 5.0602 | 4.9905 | 4.9684 | 4.9848 | 4.9601 | 4.9705 | 5.2539 | 4.9849 | 4.9746 | 4.9865 |
| neg_1501 | Lawsone | metab_9872 | HMDB0253997;HMDB0030773 | C10368 | 219.0292 | 6.0315 | neg | M+FA-H | C10H6O3 | 0 | 80.9 | -4.2226944 | 481-39-0;83-72-7 | 0.02892155 | 3.8498 | 3.7918 | 3.872 | 3.7883 | 3.5395 | 3.6968 | 3.6993 | 3.8109 | 3.3899 | 3.6331 | 3.7945 | 3.548 | 4.1815 | 4.2031 | 4.1816 |
| neg_255 | LysoPC(18:0/0:0) | metab_9873 | HMDB0010384;HMDB0011149 | C04230;C04317 | 568.3613 | 7.1985 | neg | M+Cl, M+FA-H | C26H54NO7P | 0 | 90.8 | -1.357287 | 74430-89-0;19420-57-6 | 0.00529209 | 7.4853 | 7.361 | 7.4248 | 7.4367 | 7.3337 | 7.3649 | 7.4347 | 7.3353 | 7.327 | 7.3991 | 7.4161 | 7.4353 | 7.4311 | 7.4284 | 7.4265 |
| neg_121 | Malic Acid | metab_9874 | PW_C000592;HMDB0000156;MJDBOTE0000694 | C00149 | 133.013 | 1.0835 | neg | M-H2O-H, M-H | C4H6O5 | 91.6 | 0 | -9.2630649 | 617-48-1;6915-15-7;97-67-6 | 0.01280916 | 5.3548 | 5.4592 | 5.4135 | 5.5189 | 5.6725 | 5.7059 | 5.319 | 5.3788 | 5.7225 | 4.9857 | 5.6053 | 5.5495 | 5.58 | 5.582 | 5.5904 |
| neg_1441 | Mycophenolic acid | metab_9875 | HMDB0015159;HMDB0014826;PW_C009457 | C20380 | 365.1248 | 5.9688 | neg | M+FA-H | C17H20O6 | 0 | 31.1 | 1.88829924 | 128794-94-5;24280-93-1 | 0.01625708 | 4.3676 | 2.9364 | 4.3111 | 2.9588 | 3.4973 | 4.3779 | 4.8693 | 2.928 | 2.942 | 4.3186 | 3.865 | 3.9182 | 4.2902 | 4.3032 | 4.2921 |
| neg_2735 | N-Choloylglycine | metab_9876 | HMDB0000138;HMDB0255104;HMDB0000148 | C01921 | 500.2779 | 6.4403 | neg | M+Cl | C26H43NO6 | 0 | 59.9 | -1.1958378 | 56-86-0;475-31-0 | 0.00181005 | 5.8444 | 5.9376 | 5.8726 | 5.8296 | 5.8921 | 5.9565 | 5.8423 | 5.782 | 5.8779 | 5.9073 | 5.777 | 5.7808 | 5.8711 | 5.872 | 5.8726 |
| neg_2700 | Octadec-9-enoic Acid | metab_9877 | HMDB0062703;HMDB0000573;HMDB0000207 | C00712 | 327.2536 | 6.503 | neg | M+FA-H | C18H34O2 | 0 | 79.4 | -1.6737062 | 2027-47-6;112-80-1;112-79-8 | 0.00498778 | 5.165 | 5.1267 | 5.1615 | 5.2051 | 4.8405 | 4.7922 | 5.3338 | 4.9748 | 4.9736 | 5.094 | 5.023 | 4.9002 | 5.1148 | 5.112 | 5.1163 |
| neg_3858 | Phenol sulphate | metab_9878 | PW_C040399;HMDB0060015 | C02180;C00850 | 172.9904 | 3.7371 | neg | M-H | C6H6O4S | 0 | 57.9 | -5.8127522 | 937-34-8 | 0.0071209 | 5.7817 | 6.6158 | 6.4328 | 6.5142 | 6.7572 | 6.6829 | 6.3571 | 6.1159 | 6.6976 | 6.1153 | 6.7233 | 6.7891 | 6.3586 | 6.3606 | 6.3647 |
| neg_934 | Phenylacetylglutamine | metab_9879 | HMDB0062645;HMDB0006344;PW_C002728;MJDBOTE0000584 | C04148 | 309.1089 | 3.4843 | neg | M+FA-H | C13H16N2O4 | 0 | 58.7 | -1.1469664 | 28047-15-6 | 0.0048605 | 4.8369 | 5.2113 | 5.0298 | 4.8249 | 4.9473 | 4.8633 | 5.0369 | 4.8486 | 4.9246 | 4.7756 | 4.9382 | 5.0376 | 4.9444 | 4.9482 | 4.948 |
| neg_2849 | Plastoquinone | metab_9880 | HMDB0303698;HMDB0030130 | C16694 | 249.1128 | 6.306 | neg | M+FA-H | C13H16O2 | 0 | 43.3 | -2.2742749 | 112055-76-2;4299-57-4 | 0.01489075 | 4.6744 | 4.7018 | 4.7413 | 4.7405 | 4.6808 | 4.6971 | 4.7358 | 4.6935 | 4.7259 | 4.7547 | 4.6764 | 4.7123 | 4.6928 | 4.6948 | 4.7048 |
| pos_1 | Glycocholic Acid | metab_0 | PW_C000089;HMDB0000138;HMDB0000331;LMST05030001;MJDBOTE0000466 | C01921 | 430.2963 | 6.2602 | pos | M+H-2H2O, 2M+Na, 2M+H, 2M+NH4, M+Na, M+NH4, M+K, M+2Na-H, M+H, M+H-H2O | C26H43NO6 | 86.4 | 0 | 2.31489858 | 475-31-0;68753-51-5 | 0.00490874 | 6.027 | 7.5861 | 7.2694 | 6.915 | 7.8076 | 7.8506 | 6.7452 | 6.8479 | 7.8554 | 7.1035 | 7.7362 | 7.4259 | 7.5527 | 7.5569 | 7.5542 |
| pos_10 | Peregrine | metab_1 | HMDB0256309 | - | 486.2838 | 6.125 | pos | M+K, M+2Na-H, M+Na, M+NH4, M+H-H2O | C26H41NO6 | 0 | 36.4 | 2.66832493 | - | 0.00667854 | 3.6014 | 5.3049 | 4.5202 | 4.246 | 5.2477 | 5.7162 | 4.5753 | 4.7288 | 5.5249 | 4.492 | 5.7211 | 5.056 | 5.8681 | 5.8686 | 5.8734 |
| pos_101 | 3-(2-Methylperoxyethyl)-1H-indole | metab_13 | HMDB0258019 | - | 233.1291 | 5.2017 | pos | M+H-H2O, M+ACN+H, M+ACN+Na | C11H13NO2 | 0 | 53.1 | 3.29154193 | - | 0.00200046 | 3.7011 | 7.0362 | 4.2449 | 4.2192 | 4.1833 | 4.5332 | 2.8189 | 3.7212 | 4.9208 | 4.3164 | 3.608 | 4.3679 | 5.7402 | 5.7388 | 5.7405 |
| pos_1020 | Tiglic acid | metab_24 | HMDB0001470;LMFA01020030 | C08279 | 101.0606 | 0.6583 | pos | M+H | C5H8O2 | 0 | 49.6 | 8.43876583 | 80-59-1 | 0.01841951 | 5.2633 | 5.2613 | 5.2998 | 5.298 | 5.2781 | 5.3371 | 5.3368 | 5.3352 | 5.3262 | 5.365 | 5.3202 | 5.3192 | 5.3143 | 5.3141 | 5.328 |
| pos_1043 | Isoputreanine | metab_49 | HMDB0006009 | - | 161.1289 | 0.7703 | pos | M+H | C7H16N2O2 | 0 | 49.2 | 3.08881096 | 66165-33-1 | 0.01417509 | 5.8095 | 5.6789 | 5.8185 | 5.6468 | 5.6178 | 5.339 | 5.7677 | 5.5193 | 5.913 | 5.8963 | 5.6102 | 5.4967 | 5.3775 | 5.3773 | 5.388 |
| pos_1083 | Carboxyethyllysine | metab_90 | HMDB0249659 | - | 183.111 | 0.8023 | pos | M+H-2H2O | C9H18N2O4 | 0 | 41.4 | -8.1968348 | - | 0.00702652 | 5.2283 | 5.1642 | 5.2843 | 5.1176 | 4.9778 | 4.9708 | 5.1729 | 5.0503 | 5.3931 | 5.3019 | 5.0368 | 5.1858 | 4.8653 | 4.8651 | 4.8704 |
| pos_1089 | 2-Amino-4-[carbamimidoyl(methyl)amino]butanoic acid | metab_96 | HMDB0255374 | - | 219.0834 | 0.8103 | pos | M+2Na-H | C6H14N4O2 | 0 | 65.7 | 3.45404304 | - | 0.00680981 | 5.9336 | 5.9357 | 5.8615 | 5.8714 | 5.8482 | 5.9029 | 5.9286 | 5.8914 | 5.8682 | 5.9 | 5.8633 | 5.9844 | 5.9628 | 5.9626 | 5.9678 |
| pos_1098 | Tebipenem | metab_106 | HMDB0258760 | C21522 | 348.0802 | 0.8183 | pos | M+H-2H2O | C16H21N3O4S2 | 0 | 40.2 | -8.6205524 | 161715-21-5 | 0.01060755 | 5.2213 | 5.1663 | 5.1495 | 5.1873 | 5.053 | 5.1879 | 5.1416 | 5.0655 | 5.2353 | 5.0296 | 5.191 | 5.3099 | 5.0436 | 5.0474 | 5.0527 |
| pos_11 | Trifolirhizin | metab_107 | HMDB0303946;LMPK12070033;HMDB0036630;MJDBOTE0000264 | C10538 | 469.1117 | 6.0604 | pos | M+H-H2O, M+NH4, M+Na, M+H, M+K | C22H22O10 | 0 | 52.3 | 2.67408625 | 6807-83-6 | 0.00609737 | 6.683 | 6.7061 | 6.7226 | 6.7218 | 6.6468 | 6.7177 | 6.6746 | 6.7359 | 6.6774 | 6.7562 | 6.6185 | 6.65 | 6.6123 | 6.6158 | 6.6174 |
| pos_110 | 4-(2-chloroanilino)-4-oxobutanoic acid | metab_108 | - | - | 228.0428 | 5.6498 | pos | M+H, M+Na, M+2Na-H | C10H10ClNO3 | 43.3 | 0 | 2.7654592 | - | 0.0159505 | 4.3846 | 5.3887 | 5.0571 | 5.3231 | 4.8601 | 5.1328 | 4.777 | 4.7207 | 4.7871 | 3.8123 | 5.2846 | 4.7097 | 4.9711 | 4.9599 | 4.9726 |
| pos_1128 | Creatinine | metab_138 | HMDB0000562;MJDBOTE0000819 | C00791 | 227.1257 | 1.0007 | pos | 2M+H | C4H7N3O | 70.9 | 0 | 2.71577788 | 60-27-5 | 0.0051334 | 5.3989 | 5.2956 | 5.2287 | 5.401 | 5.0037 | 5.16 | 5.1548 | 5.4725 | 5.3711 | 5.4257 | 5.3915 | 5.3368 | 5.7112 | 5.714 | 5.7157 |
| pos_1130 | L-Carnitine | metab_141 | PW_C000044;HMDB0000062;MJDBOTE0000806 | C00318 | 162.113 | 1.0087 | pos | M+H | C7H15NO3 | 79.8 | 0 | 3.17491677 | 541-15-1 | 0.00281136 | 7.3053 | 7.1609 | 7.2329 | 7.0682 | 7.2354 | 7.5087 | 7.2723 | 7.3266 | 7.2856 | 7.0928 | 7.4361 | 7.4248 | 7.2148 | 7.2124 | 7.214 |
| pos_1132 | Proline betaine | metab_143 | HMDB0004827 | C10172 | 144.1024 | 1.0167 | pos | M+H | C7H13NO2 | 0 | 38.8 | 3.3561361 | 471-87-4 | 0.00668321 | 5.1999 | 5.4941 | 5.4112 | 5.2746 | 5.3353 | 5.3162 | 5.3116 | 5.0218 | 5.409 | 5.3963 | 5.2515 | 5.2315 | 5.2906 | 5.2864 | 5.292 |
| pos_1147 | Prolylhydroxyproline | metab_158 | HMDB0256781;HMDB0006695 | - | 229.1189 | 1.0959 | pos | M+H | C10H16N2O4 | 93.5 | 0 | 2.51022804 | 18684-24-7 | 0.02206162 | 4.4797 | 4.882 | 4.3417 | 4.3873 | 4.826 | 4.5302 | 4.5905 | 4.9821 | 4.7506 | 4.853 | 4.5 | 4.7006 | 4.8583 | 4.8581 | 4.8747 |
| pos_117 | 2-Hydroxydecanedioic acid | metab_182 | HMDB0000424 | - | 459.2211 | 6.0684 | pos | M+H-2H2O, 2M+NH4, 2M+Na | C10H18O5 | 0 | 39.8 | 2.39483943 | 103963-71-9 | 0.01564615 | 4.0899 | 3.8854 | 4.0006 | 5.2905 | 3.993 | 4.0221 | 4.0871 | 3.775 | 3.9623 | 3.8769 | 3.9522 | 4.0471 | 4.7104 | 4.7101 | 4.6985 |
| pos_1174 | Carboxyphosphamide | metab_187 | PW_C040481;HMDB0060449 | C07646 | 315.0046 | 1.2872 | pos | M+Na | C7H15Cl2N2O4P | 0 | 33.2 | 2.60697311 | 22788-18-7 | 0.01262348 | 5.9625 | 5.8316 | 5.8249 | 5.7458 | 5.5372 | 5.6658 | 5.9304 | 5.8382 | 5.8486 | 5.8084 | 5.6948 | 5.8761 | 5.7488 | 5.7555 | 5.7597 |
| pos_1185 | G-Nitro-l-arginine methyl ester | metab_198 | HMDB0252563 | - | 198.0979 | 1.4556 | pos | M+H-2H2O | C7H15N5O4 | 0 | 42.4 | -2.84701 | - | 0.00570995 | 5.3598 | 5.3833 | 5.4169 | 5.4972 | 5.3796 | 5.4392 | 5.2614 | 5.4719 | 5.3847 | 5.3351 | 5.318 | 5.2841 | 5.5086 | 5.5134 | 5.51 |
| pos_1190 | Glutamic acid diethyl ester | metab_204 | HMDB0252807 | - | 245.1502 | 1.5995 | pos | M+ACN+H | C9H17NO4 | 0 | 38.2 | 3.23051551 | - | 0.01069129 | 5.5483 | 5.5766 | 5.7025 | 5.7091 | 5.4245 | 5.5505 | 5.7351 | 5.805 | 5.4745 | 5.8516 | 5.5394 | 5.6905 | 5.8778 | 5.8849 | 5.8865 |
| pos_1205 | 4-Acetamido-2-aminobutanoic acid | metab_219 | HMDB0031411 | C06442 | 125.0716 | 1.7755 | pos | M+H-2H2O | C6H12N2O3 | 0 | 33 | 3.9946429 | 1190-46-1 | 0.01905177 | 4.8267 | 4.8151 | 4.8884 | 4.8745 | 4.8411 | 4.886 | 4.9797 | 4.9586 | 4.9589 | 4.9665 | 4.9081 | 4.9148 | 4.915 | 4.9013 | 4.9163 |
| pos_1215 | Ethyl acrylate | metab_230 | HMDB0033978 | C19238 | 101.0605 | 1.8875 | pos | M+H | C5H8O2 | 0 | 39.7 | 8.32310748 | 140-88-5 | 0.01100615 | 4.571 | 4.5403 | 4.5971 | 4.6001 | 4.5948 | 4.6177 | 4.6212 | 4.6111 | 4.6332 | 4.6602 | 4.5646 | 4.5465 | 4.6256 | 4.6255 | 4.6338 |
| pos_1216 | 2-Cyclohexen-1-one | metab_231 | HMDB0245089 | C02395 | 97.0657 | 1.8955 | pos | M+H | C6H8O | 0 | 31 | 9.0955384 | 930-68-7 | 0.01147611 | 5.8394 | 5.8131 | 5.8471 | 5.8392 | 5.7931 | 5.8398 | 5.8368 | 5.8336 | 5.829 | 5.8763 | 5.7998 | 5.7975 | 5.8776 | 5.8774 | 5.8688 |
| pos_1225 | L-Isoleucine | metab_241 | PW_C000112;HMDB0000172;HMDB0000557;MJDBOTE0001077 | C00407 | 132.1025 | 1.9753 | pos | M+H | C6H13NO2 | 67.2 | 0 | 4.27680885 | 1509-34-8;73-32-5 | 0.00532774 | 7.2454 | 7.2302 | 7.1835 | 7.0553 | 7.1302 | 7.029 | 7.2835 | 7.0458 | 7.0226 | 7.1231 | 6.9856 | 7.2336 | 7.1997 | 7.1994 | 7.1955 |
| pos_1244 | 4-Oxo-L-proline | metab_262 | HMDB0304793;HMDB0246561 | C01877 | 147.0769 | 2.1111 | pos | M+NH4 | C5H7NO3 | 0 | 41.5 | 4.08762869 | 4347-18-6 | 0.00940899 | 5.3035 | 5.3388 | 5.156 | 5.1859 | 5.23 | 5.3532 | 5.1954 | 5.2213 | 5.278 | 5.2057 | 5.3991 | 5.3091 | 5.3201 | 5.3199 | 5.3271 |
| pos_1249 | L-Canaline | metab_267 | HMDB0012251 | C08270 | 176.1036 | 2.1511 | pos | M+ACN+H | C4H10N2O3 | 0 | 31.6 | 4.4578293 | 496-93-5 | 0.0145801 | 4.973 | 5.145 | 4.8781 | 5.0998 | 4.6456 | 4.6979 | 5.0913 | 4.9348 | 4.8366 | 4.9529 | 4.6235 | 4.8095 | 4.7554 | 4.7452 | 4.7568 |
| pos_1262 | (3R,4R)-3-Amino-1-hydroxy-4-methylpyrrolidin-2-one | metab_282 | HMDB0243603 | - | 169.0361 | 2.455 | pos | M+K | C5H10N2O2 | 0 | 45.2 | -9.6928664 | - | 0.03081776 | 4.3537 | 4.5167 | 4.5247 | 4.5426 | 4.5114 | 4.6239 | 4.5799 | 4.5831 | 4.6304 | 4.6594 | 4.5926 | 4.6044 | 4.7119 | 4.7346 | 4.736 |
| pos_127 | Glycochenodeoxycholic Acid | metab_290 | LMST05030008;HMDB0242392;HMDB0000637;MJDBOTE0001174 | C05466 | 414.3014 | 6.4029 | pos | M+H-2H2O, M+Na, M+H-H2O | C26H43NO5 | 31.5 | 0 | 2.56350109 | 640-79-9 | 0.00826181 | 5.8704 | 6.8697 | 6.9996 | 6.6643 | 7.2138 | 7.2212 | 6.2868 | 6.9715 | 7.1744 | 6.5538 | 7.0961 | 7.0963 | 6.948 | 6.9478 | 6.9541 |
| pos_1270 | 5-[(1-Iminoethyl)amino]-2-aminopentanoic acid | metab_291 | HMDB0246210 | - | 174.1245 | 2.5111 | pos | M+H | C7H15N3O2 | 0 | 41.4 | 4.32060816 | - | 0.00715499 | 4.567 | 5.7067 | 5.3448 | 5.5858 | 4.9659 | 5.1942 | 4.9042 | 5.1519 | 4.5875 | 4.3067 | 5.136 | 4.8333 | 4.9639 | 4.9637 | 4.9691 |
| pos_1277 | Propionylcarnitine | metab_298 | PW_C000658;LMFA07070105;HMDB0000824 | C03017 | 218.1394 | 2.5751 | pos | M+H | C10H19NO4 | 88.1 | 0 | 3.26283911 | 20064-19-1;17298-37-2 | 0.00682944 | 6.3934 | 6.4221 | 6.3691 | 6.0943 | 6.7242 | 7.0442 | 6.4432 | 6.3487 | 6.7569 | 6.2163 | 6.8877 | 6.9695 | 6.5707 | 6.5704 | 6.5757 |
| pos_1281 | D-Glucamine | metab_303 | HMDB0246693 | - | 146.0817 | 2.5911 | pos | M+H-2H2O | C6H15NO5 | 0 | 51.3 | 2.79372218 | - | 0.00800057 | 5.1841 | 5.2097 | 5.1517 | 5.207 | 5.1911 | 5.2022 | 5.0665 | 5.1072 | 5.2053 | 5.0692 | 5.146 | 5.2623 | 5.2348 | 5.2282 | 5.2298 |
| pos_1283 | 5-Hydroxy-L-tryptophan | metab_305 | PW_C000358;HMDB0000472 | C00643 | 221.0927 | 2.5991 | pos | M+H | C11H12N2O3 | 0 | 77.6 | 2.68766281 | 9/8/50 | 0.01502628 | 4.8426 | 4.9093 | 4.8167 | 4.7303 | 4.8421 | 4.7103 | 4.9995 | 4.756 | 4.5823 | 4.6475 | 4.754 | 4.7915 | 4.771 | 4.7834 | 4.7807 |
| pos_1289 | L-Valine, N-(2-hydroxy-3-butenyl)- | metab_311 | HMDB0253927 | - | 229.1553 | 2.6791 | pos | M+ACN+H | C9H17NO3 | 0 | 49.4 | 3.59759298 | - | 0.00380966 | 6.0422 | 5.9704 | 5.9525 | 5.9021 | 5.8036 | 5.762 | 6.0262 | 6.1058 | 5.8301 | 6.001 | 5.9483 | 6.0728 | 6.1607 | 6.1588 | 6.1621 |
| pos_1292 | Endothion | metab_314 | HMDB0251802 | C18982 | 313.0527 | 2.7031 | pos | M+CH3OH+H | C9H13O6PS | 0 | 46.5 | 7.83655424 | 4/3/78 | 0.06306044 | 3.962 | 4.1364 | 3.915 | 3.3167 | 3.667 | 3.627 | 3.8427 | 3.798 | 4.5464 | 3.7252 | 3.6877 | 3.8661 | 3.6281 | 3.615 | 3.5748 |
| pos_1297 | 5-Keto-D-gluconate | metab_319 | HMDB0011731 | C01062 | 212.0747 | 2.7671 | pos | M+NH4 | C6H10O7 | 0 | 44.8 | -9.3262901 | 3470-36-8 | 0.01248525 | 5.0861 | 5.2153 | 5.1589 | 4.9541 | 5.1991 | 5.2601 | 5.2953 | 4.8464 | 5.3112 | 4.7672 | 5.2271 | 5.3714 | 4.8764 | 4.8864 | 4.8778 |
| pos_1300 | 1,2,5,6-Tetrahydro-4H-pyrrolo[3,2,1-ij]quinolin-4-one | metab_324 | HMDB0037113 | C18487 | 174.0919 | 2.7751 | pos | M+H | C11H11NO | 0 | 52.6 | 3.33520061 | 57369-32-1 | 0.02210268 | 3.3718 | 3.7533 | 3.3155 | 3.482 | 4.1765 | 4.1362 | 3.4165 | 3.4689 | 4.1581 | 3.5565 | 4.0745 | 4.1285 | 3.9258 | 3.9256 | 3.9422 |
| pos_1301 | Dimethyl Phthalate | metab_325 | HMDB0251388;MJDBOTE0001757 | C11233 | 195.0658 | 2.7751 | pos | M+H | C10H10O4 | 67.6 | 0 | 3.17447364 | 131-11-3 | 0.00863497 | 4.5902 | 4.5578 | 4.4846 | 4.5092 | 4.3729 | 4.4509 | 4.4451 | 4.5303 | 4.4859 | 4.5222 | 4.4906 | 4.5298 | 4.5833 | 4.5904 | 4.585 |
| pos_1305 | Methyldopa | metab_328 | HMDB0251525;HMDB0011754;MJDBOTE0001405 | C07194 | 212.0923 | 2.7831 | pos | M+H | C10H13NO4 | 37.5 | 0 | 2.70520963 | 555-30-6 | 0.00286072 | 4.8343 | 4.2639 | 4.2294 | 4.3798 | 4.1797 | 4.2841 | 4.281 | 4.4329 | 4.4674 | 4.3161 | 4.3504 | 4.3614 | 4.5369 | 4.5367 | 4.5347 |
| pos_1306 | Pro-Pro-Pro | metab_329 | HMDB0256689 | - | 310.1768 | 2.7831 | pos | M+H | C15H23N3O4 | 0 | 54 | 2.24076932 | - | 0.03494825 | 5.2982 | 4.6375 | 5.0073 | 5.0235 | 4.2716 | 4.4368 | 4.7761 | 5.0887 | 4.993 | 4.7387 | 4.601 | 4.9331 | 4.6215 | 4.6212 | 4.5948 |
| pos_1317 | Hex-3-enedioylcarnitine | metab_340 | HMDB0241671 | - | 288.1446 | 2.8071 | pos | M+H | C13H21NO6 | 0 | 68 | 1.64952962 | - | 0.02174945 | 5.0794 | 4.1534 | 4.2777 | 4.1664 | 4.2232 | 4.3235 | 4.2932 | 4.7234 | 4.393 | 4.5511 | 4.4559 | 4.8088 | 4.4701 | 4.4857 | 4.4873 |
| pos_1323 | (E)-Casimiroedine | metab_347 | HMDB0030274 | C10577 | 418.1942 | 2.8151 | pos | M+H | C21H27N3O6 | 0 | 47.5 | -7.3477815 | 2/1/53 | 0.03305338 | 5.4323 | 5.0191 | 5.4613 | 5.0591 | 5.1095 | 4.9314 | 5.0571 | 5.081 | 5.2731 | 5.197 | 5.1476 | 5.3711 | 4.8959 | 4.9219 | 4.8989 |
| pos_1324 | Validamycin A | metab_348 | HMDB0036592 | C12112 | 462.1992 | 2.8151 | pos | M+H-2H2O | C20H35NO13 | 0 | 77.6 | 4.47447465 | 37248-47-8 | 0.02197347 | 5.1206 | 4.0082 | 4.7989 | 4.9844 | 4.6882 | 4.7745 | 5.1407 | 5.1471 | 5.4728 | 4.9251 | 4.8556 | 5.0214 | 4.76 | 4.7429 | 4.7443 |
| pos_1327 | Glycylglycylglycine | metab_351 | HMDB0029419 | - | 154.0615 | 2.8229 | pos | M+H-2H2O | C6H11N3O4 | 0 | 47.4 | 2.33497336 | 556-33-2 | 0.02694379 | 5.0157 | 4.3425 | 4.7493 | 4.9008 | 4.688 | 4.845 | 5.0639 | 5.2042 | 5.371 | 4.878 | 4.9646 | 4.9654 | 4.7805 | 4.8014 | 4.7823 |
| pos_1330 | N4-Acetylcytidine | metab_355 | HMDB0005923 | C22293 | 286.104 | 2.8229 | pos | M+H | C11H15N3O6 | 89.9 | 0 | 2.38165345 | 3768-18-1 | 0.00992163 | 5.144 | 4.3041 | 4.8096 | 4.9934 | 4.7182 | 4.9618 | 5.1231 | 5.3398 | 5.5079 | 5.0113 | 5.1033 | 5.0986 | 4.9024 | 4.909 | 4.9106 |
| pos_1331 | Norepinephrine | metab_356 | HMDB0000216;PW_C000142;MJDBOTE0001316 | C00547 | 339.1557 | 2.8229 | pos | 2M+H | C8H11NO3 | 0 | 57 | 2.00501913 | 51-41-2 | 0.04634829 | 4.7313 | 4.6047 | 4.7526 | 5.1811 | 4.7974 | 4.7061 | 5.0807 | 4.4473 | 4.7006 | 4.6489 | 4.6936 | 4.8668 | 4.9015 | 4.9359 | 4.9377 |
| pos_1337 | Aucubin | metab_362 | LMPR0102070006;HMDB0036562;MJDBOTE0001197 | C09771 | 369.1129 | 2.8309 | pos | M+Na | C15H22O9 | 0 | 40.2 | -7.7132295 | 479-98-1 | 0.00860139 | 5.105 | 5.2778 | 5.1875 | 5.1165 | 5.0778 | 5.1804 | 5.2651 | 5.2611 | 5.1181 | 5.2296 | 5.036 | 5.332 | 5.2259 | 5.2332 | 5.2285 |
| pos_1338 | 1-(5-Acetyl-2-hydroxyphenyl)-3-methyl-1-butanone | metab_363 | HMDB0032589 | - | 265.0802 | 2.8389 | pos | M+2Na-H | C13H16O3 | 0 | 48.3 | -4.2728749 | 62458-64-4 | 0.02973435 | 4.6847 | 4.888 | 4.4364 | 4.4651 | 4.695 | 4.6383 | 4.4957 | 4.7402 | 4.7484 | 4.5617 | 4.6554 | 4.8405 | 4.5567 | 4.558 | 4.5795 |
| pos_1341 | Butyryl-L-carnitine | metab_366 | HMDB0002013 | C02862 | 232.155 | 2.8548 | pos | M+H | C11H21NO4 | 58.9 | 0 | 2.92188489 | 25576-40-3 | 0.00619053 | 6.2457 | 6.0751 | 6.0817 | 5.9547 | 6.2688 | 6.3455 | 6.1348 | 6.3273 | 6.262 | 5.9658 | 6.3414 | 6.3889 | 6.1301 | 6.1299 | 6.1253 |
| pos_1347 | Tuliposide B | metab_371 | LMFA13010038;HMDB0303141 | C08570 | 312.1309 | 2.8787 | pos | M+NH4 | C11H18O9 | 0 | 59.8 | 6.62479816 | 19870-33-8 | 0.00841779 | 5.2559 | 5.2908 | 5.2242 | 5.1378 | 5.1134 | 5.169 | 5.1776 | 5.2828 | 5.3497 | 5.132 | 5.1721 | 5.261 | 5.0929 | 5.0982 | 5.0999 |
| pos_1350 | 1-Phenylbiguanide | metab_375 | HMDB0256437 | - | 160.0974 | 2.8867 | pos | M+H-H2O | C8H11N5 | 0 | 34.8 | -4.3666783 | - | 0.00434357 | 4.6553 | 4.6171 | 4.7056 | 4.6936 | 4.7025 | 4.8551 | 4.464 | 4.6723 | 4.6728 | 4.4332 | 4.6699 | 4.6928 | 4.9254 | 4.9251 | 4.9285 |
| pos_1351 | Muramic acid | metab_376 | HMDB0248237;HMDB0003254 | C06470 | 216.0873 | 2.8867 | pos | M+H-2H2O | C9H17NO7 | 0 | 51.6 | 2.66318778 | 1114-41-6 | 0.00523263 | 4.477 | 4.7375 | 4.5101 | 4.7018 | 4.1486 | 4.0058 | 4.889 | 4.4514 | 4.2763 | 4.7163 | 4.2777 | 4.2712 | 4.4576 | 4.4545 | 4.459 |
| pos_1354 | Glutarate semialdehyde | metab_379 | HMDB0012233 | C03273 | 158.0817 | 2.9107 | pos | M+ACN+H | C5H8O3 | 0 | 35.7 | 4.86657094 | 2/1/46 | 0.01249387 | 4.9154 | 5.2895 | 5.0679 | 5.1492 | 5.0613 | 5.294 | 5.2056 | 4.8504 | 5.0126 | 4.8478 | 5.3322 | 5.1801 | 5.0331 | 5.0431 | 5.0345 |
| pos_1355 | (2-Ethylcrotonoyl)urea | metab_380 | HMDB0242468 | - | 198.1244 | 2.9107 | pos | M+ACN+H | C7H12N2O2 | 0 | 39 | 4.3124769 | - | 0.0184368 | 5.1157 | 5.3167 | 5.2293 | 4.675 | 5.114 | 5.6373 | 5.489 | 3.9308 | 3.6336 | 4.7833 | 5.5398 | 5.1391 | 5.257 | 5.2452 | 5.2419 |
| pos_1370 | 3-Methyloxindole | metab_394 | HMDB0304943 | C02366 | 165.1029 | 2.9668 | pos | M+NH4 | C9H9NO | 0 | 51.9 | 4.21008183 | 1504-06-9 | 0.00472424 | 2.9986 | 3.2895 | 3.1126 | 3.154 | 4.3437 | 4.314 | 3.2472 | 3.1418 | 4.2229 | 3.1553 | 3.8449 | 4.2263 | 4.063 | 4.0594 | 4.0595 |
| pos_1379 | DOPA sulfate | metab_403 | HMDB0002028 | - | 242.0124 | 2.9988 | pos | M+H-2H2O | C9H11NO7S | 0 | 55.8 | 2.37001209 | 96253-55-3 | 0.02875389 | 2.8719 | 2.6416 | 2.8759 | 2.4318 | 4.3551 | 4.5588 | 2.9735 | 2.4283 | 4.3945 | 2.4315 | 4.3337 | 4.4346 | 4.0004 | 4.0232 | 4.0033 |
| pos_139 | LysoPC(20:4(8Z,11Z,14Z,17Z)/0:0) | metab_415 | HMDB0010396 | C04230 | 544.3412 | 6.6259 | pos | M+H, M+Na, M+H-H2O | C28H50NO7P | 0 | 82.6 | 2.61719852 | - | 0.0053075 | 7.9699 | 7.9612 | 7.8504 | 7.9308 | 7.9207 | 7.9099 | 8.008 | 7.8777 | 7.9 | 7.8747 | 7.9327 | 8.0426 | 7.8827 | 7.8787 | 7.8827 |
| pos_1394 | XI-Dihydro-2-methyl-3(2H)-thiophenone | metab_420 | HMDB0038556 | - | 134.0638 | 3.0788 | pos | M+NH4 | C5H8OS | 0 | 47.6 | 3.54430273 | - | 0.02762666 | 4.1226 | 4.1603 | 4.1441 | 4.2326 | 4.1795 | 4.2628 | 4.1542 | 4.1259 | 4.1106 | 4.2089 | 4.1329 | 4.364 | 4.3086 | 4.2884 | 4.31 |
| pos_1401 | O-Pimeloylcarnitine | metab_428 | HMDB0242187 | - | 304.1761 | 3.1108 | pos | M+H | C14H25NO6 | 0 | 58.8 | 2.24713778 | - | 0.03199999 | 4.7999 | 4.6043 | 4.5666 | 4.3997 | 4.7029 | 4.7801 | 4.7206 | 4.508 | 4.7067 | 4.3963 | 4.8711 | 4.9285 | 4.6197 | 4.5924 | 4.611 |
| pos_1409 | N'-nitrosonornicotine | metab_435 | HMDB0041940 | C16452 | 195.1233 | 3.1508 | pos | M+NH4 | C9H11N3O | 0 | 35.9 | -4.0789142 | 16543-55-8 | 0.00498901 | 3.9929 | 3.9199 | 3.9778 | 4.0507 | 3.8921 | 3.9878 | 4.0404 | 4.0152 | 3.8824 | 4.0689 | 3.9382 | 3.948 | 4.2519 | 4.2491 | 4.2533 |
| pos_141 | LysoPC(14:0/0:0) | metab_436 | HMDB0010379;PW_C006388 | C04230 | 468.3096 | 6.594 | pos | M+H-H2O, M+Na, M+H | C22H46NO7P | 0 | 62.2 | 2.33052844 | 20559-16-4 | 0.00604841 | 6.7037 | 7.0225 | 6.8575 | 6.992 | 6.8324 | 6.8891 | 6.8414 | 6.9399 | 7.0052 | 6.7819 | 6.8844 | 6.6971 | 6.796 | 6.7997 | 6.7946 |
| pos_1411 | N-Acetyl-DL-phenylalanine beta-naphthyl ester | metab_438 | HMDB0245596 | C11341 | 334.1405 | 3.1583 | pos | M+H | C21H19NO3 | 0 | 49.7 | -9.879489 | 20874-31-1 | 0.00991575 | 5.169 | 4.6405 | 4.8932 | 4.5698 | 4.8948 | 4.8137 | 4.8477 | 4.8515 | 4.6585 | 4.7558 | 4.7577 | 4.927 | 4.77 | 4.7619 | 4.7635 |
| pos_1426 | 3h-Indole-3-propanoic acid,a-amino- | metab_448 | HMDB0246269 | - | 187.0872 | 3.2104 | pos | M+H-H2O | C11H12N2O2 | 0 | 46.1 | 2.90819254 | - | 0.01925468 | 4.1348 | 4.5356 | 4.2866 | 4.8549 | 4.1553 | 4.2996 | 4.4067 | 4.0504 | 4.6968 | 4.2967 | 4.3128 | 4.6902 | 4.4089 | 4.4224 | 4.4243 |
| pos_1432 | (4S)-4-Amino-1,3-dioxohexane-1,2,6-tricarboxylic acid | metab_454 | HMDB0260161 | - | 279.0805 | 3.2344 | pos | M+NH4 | C9H11NO8 | 0 | 62.8 | -6.855827 | - | 0.01538513 | 4.4711 | 4.0231 | 4.305 | 4.3318 | 3.7414 | 3.9408 | 4.4802 | 4.0858 | 4.0568 | 4.0559 | 3.8319 | 4.5824 | 4.3508 | 4.363 | 4.3523 |
| pos_1434 | 1,3-Diacetoxy-4,6,12-tetradecatriene-8,10-diyne | metab_456 | HMDB0030922 | - | 265.1222 | 3.2424 | pos | M+H-2H2O | C18H20O4 | 0 | 35.2 | -0.3311794 | 29576-66-7 | 0.02023107 | 4.5048 | 4.8554 | 4.736 | 4.8287 | 4.9177 | 4.933 | 4.6593 | 4.605 | 5.0253 | 4.6605 | 4.9665 | 5.0309 | 4.746 | 4.7604 | 4.7621 |
| pos_1437 | 4-Hydroxy-4-(3-pyridyl)-butanoic acid | metab_459 | HMDB0001119 | C19579 | 223.1083 | 3.2584 | pos | M+ACN+H | C9H11NO3 | 0 | 30.1 | 3.4220129 | 15569-97-8 | 0.01792775 | 4.7575 | 4.6348 | 4.6852 | 4.5882 | 4.1397 | 4.4232 | 4.6465 | 4.6775 | 4.5284 | 4.5625 | 4.6044 | 4.623 | 4.3081 | 4.3074 | 4.2942 |
| pos_1448 | Nicousamide Pyrotinib Maleate | metab_468 | HMDB0255592 | - | 442.0849 | 3.3063 | pos | M+ACN+H | C18H12N2O9 | 0 | 54.2 | -8.0101563 | - | 0.13992034 | 4.6392 | 4.7048 | 4.7563 | 4.5628 | 4.5521 | 4.597 | 4.7776 | 4.8018 | 4.2752 | 4.6499 | 4.5021 | 4.8581 | 4.7674 | 4.6582 | 4.7694 |
| pos_1452 | Chlorobenzene | metab_473 | HMDB0041855 | C06990 | 154.0423 | 3.33 | pos | M+ACN+H | C6H5Cl | 0 | 58.1 | 4.4258567 | 108-90-7 | 0.00082373 | 7.7555 | 7.7511 | 7.7553 | 7.7531 | 7.7153 | 7.745 | 7.7287 | 7.7525 | 7.7722 | 7.756 | 7.7561 | 7.7255 | 7.7772 | 7.7769 | 7.7765 |
| pos_1454 | 9-Carboxymethoxymethylguanine | metab_475 | HMDB0060805 | - | 204.0502 | 3.33 | pos | M+H-2H2O | C8H9N5O4 | 0 | 34.4 | -5.7088512 | - | 0.01469792 | 5.3421 | 5.3727 | 5.3866 | 5.405 | 5.3824 | 5.387 | 5.3127 | 5.3415 | 5.3897 | 5.3559 | 5.3968 | 5.3434 | 5.3206 | 5.3089 | 5.3105 |
| pos_1460 | Garcinia acid | metab_481 | LMFA01050511;HMDB0031159 | - | 439.0322 | 3.3762 | pos | 2M+Na | C6H8O8 | 0 | 44.4 | -2.1183493 | 27750-10-3 | 0.00409757 | 5.4284 | 5.3546 | 5.4403 | 5.3752 | 5.2963 | 5.4821 | 5.3028 | 5.3567 | 5.5378 | 5.5359 | 5.423 | 5.3863 | 5.4813 | 5.48 | 5.4836 |
| pos_1464 | Isovalerylcarnitine | metab_485 | LMFA07070076;HMDB0000688 | C20826 | 246.1707 | 3.4157 | pos | M+H | C12H23NO4 | 0 | 35.7 | 2.73363053 | 31023-24-2 | 0.00256387 | 6.1081 | 6.1643 | 6.1133 | 6.0091 | 6.1771 | 6.2834 | 6.081 | 6.0965 | 6.1937 | 5.835 | 6.3037 | 6.3474 | 6.0916 | 6.0914 | 6.0896 |
| pos_1468 | Gamma-Glutamylisoleucine | metab_488 | HMDB0011170 | - | 261.1451 | 3.4316 | pos | M+H | C11H20N2O5 | 0 | 58.9 | 2.38831352 | 23632-83-9 | 0.01031678 | 5.0065 | 5.1475 | 5.0466 | 4.9172 | 4.9261 | 4.8611 | 4.9878 | 4.8942 | 4.8749 | 4.8082 | 4.7395 | 4.9944 | 4.9979 | 5.0047 | 5.0064 |
| pos_1471 | Tert-Butylbicyclophosphorothionate | metab_492 | HMDB0258754 | C19930 | 223.0543 | 3.4476 | pos | M+H | C8H15O3PS | 0 | 34 | -4.3114944 | 70636-86-1 | 0.09092606 | 3.9512 | 4.538 | 4.027 | 4.256 | 4.5217 | 4.4815 | 4.2844 | 3.6812 | 4.0895 | 3.6843 | 4.5813 | 4.0835 | 4.0418 | 3.9718 | 4.0426 |
| pos_1472 | Salidroside | metab_493 | HMDB0257463;MJDBOTE0000899 | C06046 | 333.1528 | 3.4476 | pos | M+CH3OH+H | C14H20O7 | 0 | 54.2 | -5.3187103 | 10338-51-9 | 0.01993564 | 4.6599 | 5.1284 | 5.0166 | 4.9046 | 5.0462 | 4.9712 | 5.0127 | 4.6264 | 5.0488 | 4.9904 | 4.8118 | 5.1058 | 5.2223 | 5.2067 | 5.2082 |
| pos_1475 | 2-Phenyl-1,3-propanediol monocarbamate | metab_496 | PW_C040528;HMDB0060351 | C16586 | 178.0869 | 3.4714 | pos | M+H-H2O | C10H13NO3 | 0 | 65.8 | 3.22734261 | 25451-53-0 | 0.00784267 | 4.6204 | 4.5257 | 4.5629 | 4.7253 | 4.3892 | 4.413 | 4.7823 | 4.5063 | 4.6897 | 4.3887 | 4.4983 | 4.6804 | 4.3602 | 4.3537 | 4.3553 |
| pos_1479 | Histidylglycine | metab_500 | HMDB0028885 | - | 177.0791 | 3.4874 | pos | M+H-2H2O | C8H12N4O3 | 0 | 55.2 | 9.60865278 | 2578-58-7 | 0.01001573 | 4.6007 | 4.5776 | 4.647 | 4.8122 | 4.4777 | 4.5736 | 4.6956 | 4.6278 | 4.6883 | 4.6359 | 4.5085 | 4.6361 | 4.4705 | 4.4638 | 4.4719 |
| pos_1480 | 6-Amino-N-[6-keto-6-(6-ketohexylamino)hexyl]hexanamide | metab_502 | HMDB0256669 | - | 386.2393 | 3.4954 | pos | M+2Na-H | C18H35N3O3 | 0 | 51.4 | 0.76781077 | - | 0.05496174 | 3.7171 | 4.0597 | 3.6945 | 3.8166 | 3.9937 | 3.7094 | 3.8579 | 3.8438 | 3.2668 | 3.2003 | 3.9722 | 4.0152 | 5.0839 | 5.1247 | 5.127 |
| pos_1486 | 1-Hydroxyisoquinoline | metab_506 | HMDB0243900 | C06324 | 187.0872 | 3.5278 | pos | M+ACN+H | C9H7NO | 50.4 | 0 | 4.2722433 | - | 0.03453215 | 3.9458 | 4.6349 | 4.3931 | 4.6819 | 4.1255 | 4.4636 | 4.3667 | 3.5694 | 4.0468 | 3.3072 | 4.4809 | 4.2369 | 4.2465 | 4.2208 | 4.2477 |
| pos_1489 | CHEBI:69439 | metab_508 | - | - | 197.1292 | 3.5438 | pos | M+H | C10H16N2O2 | 65.2 | 0 | 3.84384873 | - | 0.01276186 | 4.1289 | 4.1994 | 4.1918 | 3.9826 | 4.4423 | 4.3788 | 4.3394 | 4.2558 | 4.4452 | 4.233 | 4.4259 | 4.4388 | 4.2547 | 4.2648 | 4.2559 |
| pos_149 | LysoPC(18:1(11Z)/0:0) | metab_509 | HMDB0010385 | C04230 | 522.3567 | 6.9056 | pos | M+H-H2O, M+H, M+Na | C26H52NO7P | 0 | 89.4 | 2.51700254 | - | 0.02200512 | 8.3308 | 8.437 | 8.4144 | 8.4267 | 8.2595 | 8.303 | 8.3769 | 8.351 | 8.3361 | 8.296 | 8.2349 | 8.2997 | 8.3344 | 8.3515 | 8.3358 |
| pos_1496 | 2,6-Dimethylaniline | metab_514 | PW_C040580;HMDB0060677 | C11004 | 122.0971 | 3.5918 | pos | M+H | C8H11N | 76.5 | 0 | 5.26888865 | 87-62-7 | 0.01497194 | 4.8676 | 4.8553 | 4.876 | 4.856 | 4.8228 | 4.8773 | 4.8908 | 4.882 | 4.8991 | 4.9332 | 4.8609 | 4.8526 | 4.9105 | 4.8979 | 4.9072 |
| pos_1497 | 4-Methoxy-1H-indole-3-acetonitrile | metab_515 | HMDB0303405 | - | 228.114 | 3.5918 | pos | M+ACN+H | C11H10N2O | 0 | 60.1 | 4.62070213 | - | 0.01868356 | 2.6288 | 4.4131 | 4.0847 | 5.039 | 2.9481 | 2.3321 | 2.6098 | 3.9807 | 4.1792 | 2.6556 | 3.0373 | 3.3902 | 4.1491 | 4.1483 | 4.1627 |
| pos_151 | Alpha-Amylcinnamyl acetate | metab_527 | HMDB0036207 | - | 247.1699 | 6.3792 | pos | M+H, 2M+NH4, M+H-2H2O | C16H22O2 | 0 | 34.4 | 2.53993656 | 7493-78-9 | 0.02046173 | 4.9374 | 5.3262 | 5.3873 | 5.068 | 5.0781 | 5.2908 | 5.2804 | 5.2557 | 5.3868 | 5.1679 | 5.0423 | 5.2111 | 5.1735 | 5.1734 | 5.1888 |
| pos_1513 | Nitrotyrosine | metab_530 | HMDB0001904;HMDB0255667 | - | 191.0458 | 3.6709 | pos | M+H-2H2O | C9H10N2O5 | 0 | 58.3 | 3.05352183 | 3604-79-3 | 0.00542143 | 4.7649 | 4.407 | 4.5574 | 4.3894 | 4.4954 | 4.029 | 4.8969 | 3.8533 | 3.6119 | 4.3654 | 4.1995 | 4.5452 | 4.2846 | 4.285 | 4.2888 |
| pos_1515 | 3-methylheptanedioylcarnitine | metab_532 | HMDB0241046 | - | 318.1919 | 3.6709 | pos | M+H | C15H27NO6 | 0 | 63.5 | 2.58935651 | - | 0.00786121 | 4.7575 | 4.6725 | 4.6549 | 4.5202 | 4.5808 | 4.5344 | 4.6867 | 4.6195 | 4.7193 | 4.6478 | 4.7073 | 4.7401 | 4.5408 | 4.5358 | 4.5423 |
| pos_1517 | PC-M6 | metab_534 | HMDB0038568 | - | 463.2928 | 3.6875 | pos | M+ACN+H | C27H35NO3 | 0 | 74.7 | -6.3489944 | 133613-76-0 | 0.01516416 | 4.8651 | 4.8817 | 4.9362 | 4.828 | 5.0332 | 4.827 | 4.9359 | 4.8678 | 4.8372 | 4.8405 | 4.9064 | 4.9236 | 4.5304 | 4.5302 | 4.5188 |
| pos_152 | PC(20:1(11Z)/16:1(9Z)) | metab_536 | HMDB0008299;LMGP01011812;PW_C004347 | C00157 | 786.6022 | 7.0013 | pos | M+H, M+Na, M+2Na-H | C44H84NO8P | 0 | 91.2 | 1.921179 | - | 0.01821071 | 7.5725 | 7.2938 | 7.7368 | 7.6782 | 7.5232 | 7.6751 | 7.7043 | 7.7227 | 7.7406 | 7.7772 | 7.6027 | 7.6759 | 7.737 | 7.7225 | 7.7245 |
| pos_1520 | Helenalin | metab_537 | HMDB0253066 | C09473 | 280.1558 | 3.711 | pos | M+NH4 | C15H18O4 | 0 | 31.9 | 5.53870886 | 6754-13-8 | 0.00510327 | 5.2544 | 4.8442 | 5.4239 | 4.9166 | 4.7559 | 5.0782 | 5.2879 | 5.6009 | 4.9338 | 4.8982 | 5.2899 | 5.201 | 4.9467 | 4.9423 | 4.944 |
| pos_1528 | Aspartame | metab_543 | HMDB0001894;MJDBOTE0001640 | C11045 | 295.1296 | 3.7246 | pos | M+H | C14H18N2O5 | 0 | 44.7 | 2.56707755 | 22839-47-0 | 0.01893336 | 5.007 | 5.1699 | 5.0557 | 4.7718 | 4.9756 | 4.9325 | 5.096 | 5.0352 | 4.9106 | 4.9045 | 4.8846 | 5.0594 | 5.0923 | 5.1072 | 5.0938 |
| pos_1531 | Methionyl-Histidine | metab_547 | HMDB0028975 | - | 251.0935 | 3.7397 | pos | M+H-2H2O | C11H18N4O3S | 0 | 47.4 | -8.9558512 | - | 0.03233012 | 2.5909 | 3.8774 | 3.3683 | 2.3284 | 3.667 | 4.4077 | 4.0974 | 2.3249 | 2.3253 | 2.3281 | 4.5986 | 3.5336 | 3.5194 | 3.5441 | 3.5205 |
| pos_1547 | Coniferin | metab_561 | HMDB0013682 | C00761 | 381.0947 | 3.8091 | pos | M+K | C16H22O8 | 0 | 50.3 | 0.30947341 | 124151-33-3;531-29-3 | 0.01701744 | 4.9751 | 5.2813 | 5.0845 | 4.8781 | 5.1847 | 5.236 | 5.0736 | 4.9256 | 5.206 | 4.4844 | 5.215 | 5.197 | 5.1425 | 5.1423 | 5.1295 |
| pos_1551 | Dioscorine | metab_566 | HMDB0030305 | C10142 | 460.3128 | 3.833 | pos | 2M+NH4 | C13H19NO2 | 0 | 55.8 | -9.5029475 | 3329-91-7 | 0.01626186 | 5.2474 | 5.279 | 5.2703 | 5.2817 | 5.3963 | 5.272 | 5.2541 | 5.2425 | 5.2402 | 5.1925 | 5.2689 | 5.3061 | 5.4745 | 5.4628 | 5.4757 |
| pos_1552 | 2-Methoxyacetaminophen sulfate | metab_567 | HMDB0062550 | - | 244.0281 | 3.841 | pos | M+H-H2O | C9H11NO6S | 0 | 56.4 | 2.7505795 | 53446-13-2 | 0.00675574 | 3.3933 | 4.2123 | 3.5811 | 3.1446 | 4.8036 | 4.7562 | 3.6051 | 2.8426 | 4.6896 | 2.8458 | 4.8236 | 5.0222 | 4.7474 | 4.7472 | 4.7524 |
| pos_1563 | 2-cis-abscisate | metab_576 | HMDB0304067 | - | 246.1244 | 3.961 | pos | M+H-H2O | C15H19O4- | 0 | 41.3 | -2.4096601 | - | 0.00956789 | 4.6416 | 4.9751 | 4.8726 | 4.2695 | 4.6984 | 5.2135 | 5.1416 | 3.2896 | 2.6736 | 4.2302 | 5.1392 | 4.789 | 4.8679 | 4.86 | 4.8617 |
| pos_157 | 3,4-Dimethyl-5-pentyl-2-furanpropanoic acid | metab_581 | HMDB0112083;LMFA01150023 | - | 239.1647 | 6.2682 | pos | M+H, M+Na, M+H-H2O | C14H22O3 | 0 | 50.2 | 2.42099054 | 116627-39-5 | 0.00519174 | 4.3625 | 4.8342 | 4.5458 | 4.6557 | 4.6995 | 4.6771 | 4.8212 | 4.6781 | 4.9911 | 4.6568 | 4.8419 | 5.0527 | 5.1454 | 5.1466 | 5.1497 |
| pos_1573 | 1-Heptadecanoylglycerophosphoethanolamine | metab_583 | HMDB0061691 | - | 490.2872 | 4.0271 | pos | M+Na | C22H46NO7P | 0 | 41.5 | -6.7759177 | - | 0.00163229 | 4.725 | 4.8395 | 4.8621 | 4.7066 | 4.894 | 4.7421 | 4.8182 | 4.7537 | 4.6331 | 4.6729 | 4.8278 | 4.8344 | 5.1277 | 5.1284 | 5.1291 |
| pos_1578 | (2R,3R)-2-Aminooctadecane-1,3-diol | metab_588 | HMDB0242634 | - | 340.2603 | 4.0561 | pos | M+K | C18H39NO2 | 0 | 42.1 | -3.2262008 | - | 0.00055771 | 5.2877 | 5.2807 | 5.2886 | 5.3154 | 5.2213 | 5.3094 | 5.2508 | 5.3097 | 5.2788 | 5.3395 | 5.2608 | 5.2019 | 5.3216 | 5.3217 | 5.3212 |
| pos_1579 | 1-Naphthylamine | metab_589 | HMDB0243964 | C14790 | 144.0813 | 4.0636 | pos | M+H | C10H9N | 39.9 | 0 | 3.69787979 | 134-32-7 | 0.00707801 | 3.5261 | 3.6942 | 3.9261 | 3.6967 | 4.3817 | 4.2362 | 3.8582 | 3.4822 | 4.2199 | 3.4151 | 4.1631 | 4.2445 | 4.0998 | 4.0959 | 4.102 |
| pos_1580 | Fructosyl-lysine | metab_591 | HMDB0252495;HMDB0034879;HMDB0242138 | C16488 | 347.1246 | 4.0636 | pos | M+K | C12H24N2O7 | 0 | 35.8 | 9.95022286 | 21291-40-7 | 0.02262241 | 3.3949 | 4.1406 | 3.4067 | 3.8007 | 3.49 | 2.9346 | 3.2522 | 3.7312 | 3.445 | 2.7928 | 2.8001 | 3.386 | 3.9892 | 4.0055 | 4.007 |
| pos_159 | Homodihydrojasmone | metab_601 | HMDB0031181 | - | 198.1858 | 6.1729 | pos | M+H-H2O, M+H, M+NH4 | C12H20O | 0 | 31.4 | 3.25596269 | 4868-24-0 | 0.00389341 | 5.5087 | 5.4976 | 5.4991 | 5.4743 | 5.4838 | 5.5333 | 5.567 | 5.4884 | 5.5116 | 5.5366 | 5.4962 | 5.4889 | 5.5181 | 5.5179 | 5.5151 |
| pos_1592 | 4-Chloro-L-phenylalanine | metab_604 | HMDB0244605 | - | 241.0745 | 4.1384 | pos | M+ACN+H | C9H10ClNO2 | 0 | 70.9 | 3.29385 | - | 0.00258975 | 5.7726 | 5.8182 | 5.7586 | 5.7452 | 5.7451 | 5.7917 | 5.7486 | 5.7148 | 5.7838 | 5.7674 | 5.7976 | 5.726 | 5.8133 | 5.8112 | 5.8128 |
| pos_1593 | 6-Hydroxynorketamine | metab_605 | HMDB0243590 | - | 278.0342 | 4.1384 | pos | M+K | C12H14ClNO2 | 0 | 46.6 | -0.9144041 | - | 0.00723996 | 4.5214 | 4.5252 | 4.7041 | 4.6797 | 4.2027 | 4.2802 | 4.5799 | 4.7669 | 4.3161 | 4.3898 | 4.5629 | 4.4604 | 4.4185 | 4.4183 | 4.4238 |
| pos_1596 | (+/-)-Hexanoylcarnitine | metab_608 | - | - | 260.1863 | 4.1624 | pos | M+H | C13H25NO4 | 48.5 | 0 | 2.62548029 | - | 0.01448263 | 3.9291 | 3.8866 | 3.9305 | 3.74 | 4.1357 | 4.2867 | 4.0953 | 3.2681 | 4.3218 | 3.3306 | 4.3156 | 4.3911 | 4.7894 | 4.7856 | 4.7771 |
| pos_1597 | Deoxycholylproline | metab_609 | HMDB0242438 | - | 534.3137 | 4.1704 | pos | M+2Na-H | C29H47NO5 | 0 | 43.5 | -5.9843939 | - | 0.005967 | 4.6477 | 4.7472 | 4.6688 | 4.6275 | 4.7958 | 4.6136 | 4.6912 | 4.6361 | 4.5375 | 4.6084 | 4.711 | 4.7636 | 4.9257 | 4.9218 | 4.9208 |
| pos_1598 | Ile Ile | metab_610 | - | - | 245.1866 | 4.1784 | pos | M+H | C12H24N2O3 | 52.9 | 0 | 2.62356028 | - | 0.00601391 | 5.2232 | 5.0921 | 4.8097 | 5.1412 | 4.8454 | 4.942 | 5.0186 | 5.1655 | 4.827 | 5.0509 | 4.8963 | 5.1041 | 4.872 | 4.8754 | 4.8771 |
| pos_1606 | 2-Hydroxyquinoline | metab_620 | - | C06338 | 146.0605 | 4.2522 | pos | M+H | C9H7NO | 69.8 | 0 | 3.48027852 | 70254-42-1 | 0.02918392 | 3.8761 | 3.7712 | 3.9635 | 3.9238 | 3.6807 | 3.7322 | 3.8425 | 4.1828 | 3.6694 | 3.8932 | 3.8315 | 3.8977 | 3.7826 | 3.8049 | 3.7837 |
| pos_1611 | Indolylacryloylglycine | metab_626 | HMDB0006005 | - | 227.0821 | 4.3288 | pos | M+H-H2O | C13H12N2O3 | 0 | 77 | 2.56461635 | 3475-68-1 | 0.00330069 | 5.2515 | 5.6032 | 5.3142 | 5.3128 | 5.5556 | 5.4649 | 5.3777 | 5.2056 | 5.5293 | 5.125 | 5.4517 | 5.717 | 5.5324 | 5.5295 | 5.5312 |
| pos_1629 | 4-(pyrazine-2-carbonyl)-1,3-dihydroquinoxalin-2-one | metab_645 | - | - | 255.0901 | 4.4289 | pos | M+H | C13H10N4O2 | 48.1 | 0 | 9.57894521 | - | 0.00467365 | 3.8418 | 5.2734 | 4.8548 | 5.1859 | 4.3323 | 4.4979 | 4.4067 | 4.6017 | 4.5434 | 3.7251 | 4.8301 | 4.3083 | 4.7331 | 4.729 | 4.7307 |
| pos_1633 | Isoleucylproline | metab_650 | HMDB0011174 | - | 211.1447 | 4.4529 | pos | M+H-H2O | C11H20N2O3 | 0 | 47.8 | 2.6142491 | 37462-92-3 | 0.00446055 | 3.7475 | 4.0352 | 4.0333 | 3.5183 | 4.8584 | 4.9813 | 4.3878 | 4.1574 | 4.9281 | 4.1208 | 4.8746 | 4.7903 | 4.6735 | 4.6773 | 4.6751 |
| pos_1637 | 5'-Deoxy-5-fluorocytidine | metab_654 | HMDB0060405 | C16635 | 228.0775 | 4.4834 | pos | M+H-H2O | C9H12FN3O4 | 0 | 79.1 | -1.4723227 | - | 0.01278343 | 4.114 | 4.2562 | 4.2197 | 4.1281 | 4.016 | 4.6805 | 4.6888 | 0.083 | 0.083 | 3.0703 | 4.5111 | 4.1184 | 4.0125 | 4.0014 | 4.0073 |
| pos_164 | N-(Dimethylamino)methylene-9-((2-hydroxy-1-(hydroxymethyl)ethoxy)methyl)guanine | metab_657 | HMDB0247659 | - | 293.138 | 6.0289 | pos | M+H-2H2O, M+H-H2O, M+Na | C12H18N6O4 | 0 | 45.9 | 7.43634538 | - | 0.00482295 | 4.7964 | 5.0528 | 4.7574 | 4.8988 | 4.9708 | 4.8609 | 5.1211 | 4.5574 | 4.7933 | 4.733 | 4.9296 | 5.03 | 5.1095 | 5.1093 | 5.113 |
| pos_1643 | Paeonol | metab_661 | HMDB0256081;MJDBOTE0001333 | C10712 | 208.0975 | 4.5074 | pos | M+ACN+H | C9H10O3 | 0 | 37.7 | 3.80941881 | 552-41-0 | 0.00428103 | 3.6636 | 4.1309 | 4.0807 | 3.9814 | 4.3563 | 4.2879 | 3.8387 | 3.7619 | 4.2646 | 3.5924 | 4.1774 | 4.1513 | 4.0777 | 4.0739 | 4.0756 |
| pos_1645 | DL-2-Aminooctanoic acid | metab_663 | HMDB0000991 | - | 160.1337 | 4.5154 | pos | M+H | C8H17NO2 | 58.8 | 0 | 3.24382969 | 644-90-6 | 0.00990027 | 3.9996 | 3.6321 | 3.5983 | 3.7872 | 3.7218 | 4.1244 | 3.9269 | 4.1739 | 4.203 | 4.0038 | 4.231 | 4.0515 | 4.1846 | 4.1841 | 4.1918 |
| pos_1648 | PG(i-12:0/i-19:0) | metab_665 | HMDB0116667;PW_C076285 | C00344 | 377.2342 | 4.5474 | pos | M+2Na | C37H73O10P | 0 | 30.3 | -5.9966634 | - | 0.00373898 | 7.0574 | 7.1079 | 7.1393 | 7.1475 | 7.2225 | 7.1013 | 7.155 | 7.1059 | 7.0473 | 7.0359 | 7.1655 | 7.1931 | 6.932 | 6.9302 | 6.9334 |
| pos_1650 | Isoketocamphoric acid | metab_668 | HMDB0301824 | - | 234.1342 | 4.5714 | pos | M+NH4 | C10H16O5 | 0 | 65.8 | 2.83639442 | - | 0.02975251 | 5.008 | 4.9914 | 5.0077 | 5.0011 | 4.9237 | 5.0063 | 4.9889 | 5.0023 | 5.0096 | 5.0127 | 4.9711 | 4.9754 | 5.3955 | 5.3999 | 5.4196 |
| pos_166 | Trans-p-Menthane-7,8-diol 8-glucoside | metab_678 | HMDB0033231 | - | 357.1892 | 6.0604 | pos | M+H-H2O, M+H, M+Na | C16H30O7 | 0 | 39.7 | 2.41041256 | 217962-31-7 | 0.00442569 | 3.8003 | 3.9936 | 3.5434 | 3.468 | 3.6892 | 3.4152 | 4.4998 | 0.4433 | 3.9538 | 4.0855 | 3.7131 | 3.4019 | 5.0662 | 5.066 | 5.0694 |
| pos_1664 | KAPA | metab_683 | HMDB0240687 | C01092 | 188.1287 | 4.6434 | pos | M+H | C9H17NO3 | 61.1 | 0 | 3.16426226 | 682799-71-9 | 0.0102856 | 4.838 | 4.8125 | 4.863 | 4.8437 | 4.7732 | 4.873 | 4.8266 | 4.8475 | 4.8492 | 4.8577 | 4.8177 | 4.8056 | 4.8991 | 4.8922 | 4.9005 |
| pos_1665 | SM(d16:1/PGJ2) | metab_684 | HMDB0290269 | - | 399.2474 | 4.6434 | pos | M+2Na | C41H73N2O8P | 0 | 56.7 | 7.7262436 | - | 0.00532289 | 7.1858 | 7.2197 | 7.28 | 7.2502 | 7.3711 | 7.2582 | 7.2768 | 7.2302 | 7.1774 | 7.1503 | 7.2753 | 7.3297 | 7.0388 | 7.0357 | 7.0402 |
| pos_1672 | L-4-Chlorotryptophan | metab_692 | HMDB0030400 | - | 280.0854 | 4.6754 | pos | M+ACN+H | C11H11ClN2O2 | 0 | 46.8 | 2.88054363 | 52448-14-3 | 0.00209877 | 5.4999 | 5.8252 | 5.7478 | 5.3518 | 5.6442 | 6.121 | 5.9332 | 4.3871 | 3.8813 | 5.1541 | 6.1387 | 5.5208 | 5.7396 | 5.7407 | 5.7389 |
| pos_1676 | SM(d16:2(4E,8Z)/20:5(6E,8Z,11Z,14Z,17Z)-OH(5)) | metab_696 | HMDB0290329 | - | 379.2498 | 4.6914 | pos | M+H+Na | C41H71N2O7P | 0 | 60.5 | 4.36151823 | - | 0.00744789 | 6.7686 | 6.7933 | 6.8605 | 6.8393 | 6.93 | 6.818 | 6.8802 | 6.8178 | 6.7682 | 6.757 | 6.8577 | 6.9038 | 6.7691 | 6.7736 | 6.7754 |
| pos_1681 | 2-Pentyl-3-phenyl-2-propenal | metab_702 | HMDB0031313 | C12288 | 241.0978 | 4.7154 | pos | M+K | C14H18O | 0 | 37.8 | -5.671776 | 122-40-7 | 0.00902549 | 4.619 | 5.1215 | 4.6275 | 4.7679 | 4.7323 | 4.6362 | 4.8906 | 4.6998 | 4.7932 | 4.6896 | 4.5817 | 5.1142 | 4.8609 | 4.8668 | 4.8684 |
| pos_1682 | PI(22:2(13Z,16Z)/PGF1alpha) | metab_703 | HMDB0277787 | - | 496.3054 | 4.7154 | pos | M+2H | C51H91O16P | 0 | 66.1 | -8.355648 | - | 0.00584249 | 6.165 | 6.1844 | 6.2563 | 6.2261 | 6.3106 | 6.2146 | 6.2728 | 6.1967 | 6.1325 | 6.1255 | 6.2661 | 6.3147 | 6.1462 | 6.1495 | 6.1512 |
| pos_1698 | PGP(i-22:0/TXB2) | metab_719 | HMDB0275860 | - | 501.2921 | 4.7794 | pos | M+2H | C48H90O17P2 | 0 | 46.7 | 4.3546902 | - | 0.028351 | 5.0699 | 5.1373 | 5.2748 | 5.2031 | 5.2575 | 5.1583 | 5.231 | 5.1065 | 5.1412 | 5.0594 | 5.2578 | 5.2338 | 5.0417 | 5.0415 | 5.0628 |
| pos_1699 | PI(18:0/18:2(9Z,12Z)) | metab_720 | HMDB0009809;LMGP06010956 | C00626 | 443.2736 | 4.8034 | pos | M+H+Na | C45H83O13P | 0 | 36.6 | -7.3429989 | - | 0.00519056 | 7.1962 | 7.2144 | 7.2891 | 7.2659 | 7.3373 | 7.2213 | 7.2758 | 7.2106 | 7.1917 | 7.1573 | 7.2849 | 7.3205 | 7.0269 | 7.0307 | 7.0309 |
| pos_1708 | SM(d18:1/PGF2alpha) | metab_731 | HMDB0290519 | - | 423.2761 | 4.8434 | pos | M+2Na | C43H81N2O9P | 0 | 47.8 | 7.11428871 | - | 0.00925537 | 6.6582 | 6.7089 | 6.746 | 6.7507 | 6.8268 | 6.6832 | 6.7634 | 6.6831 | 6.6394 | 6.6514 | 6.7659 | 6.7976 | 6.6285 | 6.6341 | 6.6263 |
| pos_172 | 1-O-Isopentyl-3-O-octadec-2-enoyl glycerol | metab_742 | HMDB0302897 | - | 427.3792 | 7.3757 | pos | M+H, M+NH4, 2M+Na | C26H50O4 | 0 | 54.6 | 2.33827744 | - | 0.00217919 | 8.6185 | 8.6467 | 8.6196 | 8.5984 | 8.49 | 8.6026 | 8.6148 | 8.5659 | 8.6514 | 8.6488 | 8.5523 | 8.5715 | 8.6488 | 8.6505 | 8.6503 |
| pos_173 | Sebacic acid | metab_753 | LMFA01170006;HMDB0000792 | C08277 | 185.1175 | 7.3757 | pos | M+H-H2O, M+H, M+H-2H2O | C10H18O4 | 92.5 | 0 | 1.61111636 | 111-20-6 | 0.00899127 | 6.6391 | 6.6763 | 6.6539 | 6.5896 | 6.5066 | 6.6336 | 6.6422 | 6.5784 | 6.703 | 6.6748 | 6.5796 | 6.608 | 6.598 | 6.6038 | 6.6055 |
| pos_1733 | PI(20:0/20:3(6,8,11)-OH(5)) | metab_757 | HMDB0277061 | - | 467.3024 | 4.9786 | pos | M+2H | C49H89O14P | 0 | 34.4 | -9.3085878 | - | 0.00226507 | 6.3333 | 6.4123 | 6.4551 | 6.4081 | 6.4884 | 6.4175 | 6.4564 | 6.3819 | 6.3567 | 6.3292 | 6.4477 | 6.4667 | 6.2731 | 6.2714 | 6.273 |
| pos_1735 | Epanolol | metab_759 | HMDB0251835 | C11773 | 352.1663 | 4.9866 | pos | M+H-H2O | C20H23N3O4 | 0 | 31.5 | 1.94289448 | 86880-51-5 | 0.00081706 | 4.5578 | 4.737 | 4.3936 | 4.9534 | 4.6262 | 4.7135 | 4.9455 | 4.7235 | 4.5639 | 4.7676 | 4.7692 | 4.7831 | 5.0238 | 5.0236 | 5.0243 |
| pos_174 | 4-Hydroxynonenal | metab_764 | HMDB0004362;HMDB0245269;LMFA06000051 | C21642 | 139.1122 | 7.3757 | pos | M+H-H2O, M+H, M+H-2H2O | C9H16O2 | 0 | 39.3 | 2.75736674 | 75899-68-2 | 0.00402678 | 6.9135 | 6.9422 | 6.9186 | 6.8821 | 6.7719 | 6.8956 | 6.9048 | 6.8586 | 6.9504 | 6.9479 | 6.8489 | 6.8715 | 6.9268 | 6.9287 | 6.9303 |
| pos_1747 | Netilmicin | metab_772 | PW_C009396;HMDB0015090 | C07657 | 489.3156 | 5.0273 | pos | 2M+3H2O+2H | C21H41N5O7 | 0 | 71.8 | 7.25064997 | 56391-56-1 | 0.01416703 | 6.1124 | 6.1653 | 6.2373 | 6.1932 | 6.3072 | 6.179 | 6.1979 | 6.1499 | 6.139 | 6.1004 | 6.2116 | 6.2635 | 6.0364 | 6.0362 | 6.0469 |
| pos_1756 | Paucine | metab_782 | HMDB0029876 | C03002 | 292.1664 | 5.0738 | pos | M+ACN+H | C13H18N2O3 | 0 | 55.6 | 3.5100076 | 29554-26-5 | 0.10720793 | 3.7552 | 4.5123 | 3.9328 | 3.8233 | 3.8233 | 4.1088 | 4.2642 | 3.7655 | 3.2152 | 3.218 | 4.0092 | 3.7262 | 3.7983 | 3.8212 | 3.8863 |
| pos_1765 | 1-Hydroxy-2,2,5,5-tetramethylpyrrolidine-3-carboxamide | metab_792 | HMDB0243894 | - | 187.1447 | 5.1105 | pos | M+H | C9H18N2O2 | 0 | 41.4 | 2.93634429 | - | 0.00756759 | 5.2999 | 5.3005 | 5.3078 | 5.0463 | 5.2067 | 5.0511 | 5.5626 | 5.3115 | 5.4321 | 5.1291 | 5.2794 | 5.415 | 5.2678 | 5.2676 | 5.262 |
| pos_177 | Simonin IV | metab_796 | HMDB0029977 | - | 672.411 | 5.0939 | pos | M+2H+Na, M+2Na+H, M+H+Na | C68H120O24 | 0 | 62.8 | 6.45974277 | 151310-53-1 | 0.00528652 | 7.4343 | 7.5129 | 7.6008 | 7.5321 | 7.5966 | 7.5146 | 7.5779 | 7.5014 | 7.4692 | 7.4285 | 7.5723 | 7.5871 | 7.3359 | 7.3389 | 7.3405 |
| pos_1782 | N-Acetyl-D-tryptophan | metab_807 | HMDB0255052 | C03137 | 247.1083 | 5.1937 | pos | M+H | C13H14N2O3 | 0 | 54.6 | 2.24704944 | 1/5/80 | 0.01501955 | 3.6917 | 3.8563 | 4.0138 | 3.8925 | 3.146 | 3.587 | 3.9725 | 3.987 | 3.6114 | 3.1522 | 3.9382 | 4.2166 | 4.1678 | 4.1796 | 4.1691 |
| pos_1790 | Trp Phe | metab_814 | - | - | 352.1663 | 5.2248 | pos | M+H | C20H21N3O3 | 95.9 | 0 | 2.22578631 | - | 0.00746432 | 4.5759 | 4.831 | 4.3921 | 4.5929 | 4.5565 | 4.3863 | 4.7034 | 4.5361 | 4.6456 | 4.4039 | 4.3072 | 4.4836 | 4.3645 | 4.3598 | 4.366 |
| pos_18 | LysoPC(16:0/0:0) | metab_824 | HMDB0010382 | C04230 | 496.341 | 6.8738 | pos | M+Na, 2M+H, M+K, M+H, M+H-H2O | C24H50NO7P | 0 | 77.5 | 2.53184254 | 17364-16-8 | 0.0184787 | 8.5484 | 8.4645 | 8.4197 | 8.4458 | 8.3437 | 8.4099 | 8.4431 | 8.4576 | 8.4514 | 8.3806 | 8.3999 | 8.3798 | 8.4008 | 8.4153 | 8.4022 |
| pos_181 | Indoleacrylic acid | metab_836 | HMDB0000734 | - | 188.0712 | 3.2824 | pos | M+H, M+NH4, M+H-H2O | C11H9NO2 | 0 | 38.7 | 3.14882459 | 29953-71-7 | 0.00130999 | 7.3054 | 7.6464 | 7.495 | 7.4865 | 7.3558 | 7.3203 | 7.6361 | 7.4123 | 7.3092 | 7.3286 | 7.376 | 7.443 | 7.4924 | 7.4922 | 7.4913 |
| pos_1862 | Ghrih-A | metab_893 | HMDB0252714 | - | 819.371 | 5.4614 | pos | M+2H | C76H104N18O19S2 | 0 | 98 | 6.59339722 | - | 0.01054526 | 6.7981 | 6.3171 | 6.3305 | 6.1647 | 6.3289 | 6.2105 | 6.2412 | 6.624 | 6.4273 | 6.0848 | 6.5238 | 6.3915 | 6.139 | 6.1304 | 6.132 |
| pos_1876 | Panipenem | metab_908 | HMDB0256111 | - | 304.1087 | 5.5039 | pos | M+H-2H2O | C15H21N3O4S | 0 | 50.8 | -7.9230959 | - | 0.02863586 | 5.7572 | 6.3491 | 6.345 | 6.2498 | 6.3032 | 6.3357 | 6.1784 | 5.3888 | 5.1964 | 5.5949 | 6.3151 | 6.3881 | 5.9566 | 5.9564 | 5.9779 |
| pos_1881 | Histidylleucine | metab_914 | HMDB0253179;HMDB0028889 | C05010 | 332.1709 | 5.5171 | pos | M+ACN+Na | C12H20N4O3 | 0 | 62.8 | 6.00344557 | 7763-65-7 | 0.02478786 | 5.0307 | 4.5941 | 4.7236 | 5.3006 | 4.3779 | 3.0215 | 4.3169 | 4.5282 | 3.9846 | 4.3522 | 4.2086 | 4.4654 | 4.3246 | 4.3438 | 4.326 |
| pos_1888 | 3-[1-(4-methylphenyl)sulfonylpiperidin-4-yl]-1,2-oxazol-5-amine | metab_921 | - | - | 322.1193 | 5.5372 | pos | M+H | C15H19N3O3S | 76.3 | 0 | -8.262612 | - | 0.02041701 | 5.0499 | 5.7171 | 5.6771 | 5.4582 | 5.5729 | 5.5285 | 5.524 | 4.5995 | 4.4758 | 4.5767 | 5.4888 | 5.5325 | 5.2143 | 5.1995 | 5.2155 |
| pos_1893 | Nicotine glucuronide | metab_927 | HMDB0001272;PW_C000985 | - | 361.1401 | 5.5816 | pos | M+Na | C16H22N2O6 | 0 | 66.5 | 9.09168123 | 152306-59-7 | 0.0095089 | 4.0511 | 4.7776 | 4.316 | 4.2927 | 3.9987 | 3.8617 | 4.0228 | 4.2363 | 3.6737 | 3.6802 | 4.0419 | 3.8944 | 4.6459 | 4.6474 | 4.6396 |
| pos_19 | Taurocholic acid | metab_933 | LMST05040001;HMDB0000036;PW_C000025 | C05122 | 533.3269 | 6.1649 | pos | M+H-2H2O, M+H, M+NH4, M+2Na-H, M+H-H2O | C26H45NO7S | 92.2 | 0 | 2.79667407 | 81-24-3 | 0.00458097 | 6.6941 | 6.9161 | 6.0923 | 6.0035 | 7.1385 | 7.0277 | 6.569 | 6.1302 | 7.0363 | 7.0436 | 6.95 | 6.8377 | 6.6545 | 6.6543 | 6.6509 |
| pos_1906 | 2'-Deoxy-5'-O-thiophosphonouridine | metab_941 | HMDB0251638 | - | 307.0164 | 5.6498 | pos | M+H-H2O | C9H13N2O7PS | 0 | 30.5 | 4.76715468 | - | 0.0169441 | 3.5028 | 4.6888 | 4.3536 | 4.607 | 4.1863 | 4.4269 | 3.9863 | 4.0213 | 4.0328 | 2.688 | 4.6113 | 4.0432 | 4.2779 | 4.2659 | 4.2794 |
| pos_1911 | Domoic acid | metab_947 | HMDB0033939 | C13732 | 344.1711 | 5.6791 | pos | M+CH3OH+H | C15H21NO6 | 0 | 54.8 | 2.33557289 | 14277-97-5 | 0.01055882 | 3.9183 | 2.855 | 2.8512 | 3.9024 | 1.6253 | 0.7178 | 2.817 | 3.0398 | 0.7184 | 4.3027 | 0.4749 | 0.4716 | 4.8594 | 4.8594 | 4.8673 |
| pos_1912 | Melanettin | metab_948 | HMDB0041758 | C10504 | 302.1029 | 5.6857 | pos | M+NH4 | C16H12O5 | 0 | 72.9 | 2.24546641 | 58115-08-5 | 0.01151834 | 4.5759 | 4.0875 | 4.5588 | 4.565 | 3.5352 | 3.7591 | 4.556 | 4.5381 | 4.0287 | 4.6097 | 3.5522 | 4.054 | 4.5898 | 4.5998 | 4.5951 |
| pos_1919 | 3-Methylcrotonylglycine | metab_954 | HMDB0000459 | C20828 | 202.0449 | 5.7332 | pos | M+2Na-H | C7H11NO3 | 0 | 48.3 | -0.8459724 | 33008-07-0 | 0.00683553 | 4.3111 | 4.6664 | 4.4485 | 4.4572 | 4.3459 | 4.5048 | 4.4784 | 4.2634 | 4.2051 | 4.2054 | 4.4958 | 4.3303 | 4.4219 | 4.4218 | 4.4167 |
| pos_1920 | Fenamic acid | metab_956 | HMDB0246312;HMDB0255225 | C13697 | 214.0869 | 5.7378 | pos | M+H | C13H11NO2 | 85.8 | 0 | 2.93066135 | 91-40-7 | 0.01751152 | 4.5332 | 4.8943 | 4.541 | 4.8239 | 4.8338 | 4.6938 | 4.6435 | 4.574 | 4.8772 | 4.1203 | 4.8174 | 4.7371 | 4.4555 | 4.4419 | 4.4429 |
| pos_1921 | 2-(4-Chlorophenoxy)propionic acid | metab_957 | HMDB0244931 | C13701 | 242.0584 | 5.7378 | pos | M+ACN+H | C9H9ClO3 | 0 | 38.1 | 2.95093551 | 3307-39-9 | 0.00988583 | 4.757 | 5.3683 | 5.106 | 5.2735 | 4.887 | 5.1607 | 5.031 | 4.9012 | 4.8301 | 4.6242 | 5.2446 | 4.8891 | 5.0876 | 5.0796 | 5.0809 |
| pos_1922 | Carisbamate | metab_958 | HMDB0249677 | - | 257.0693 | 5.7378 | pos | M+ACN+H | C9H10ClNO3 | 0 | 48.7 | 2.76449789 | - | 0.00988314 | 4.3732 | 4.9163 | 4.5828 | 4.5245 | 4.4514 | 4.8515 | 4.7473 | 3.6795 | 3.3392 | 3.6193 | 4.8276 | 4.3683 | 4.5614 | 4.5589 | 4.553 |
| pos_1934 | Methenamine | metab_970 | HMDB0029598 | - | 141.114 | 5.7983 | pos | M+H | C6H12N4 | 48.1 | 0 | 3.74898496 | 100-97-0 | 0.02694869 | 3.72 | 3.7364 | 3.8456 | 3.8697 | 3.6825 | 3.8394 | 3.8676 | 3.88 | 3.7099 | 3.8611 | 3.6745 | 3.6863 | 3.8632 | 3.8838 | 3.8642 |
| pos_1970 | 3-[1-(2-methylphenyl)sulfonylpiperidin-4-yl]-1,2-oxazol-5-amine | metab_1006 | - | - | 322.1193 | 5.9408 | pos | M+H | C15H19N3O3S | 71 | 0 | -8.2609302 | - | 0.04408693 | 4.5979 | 5.1786 | 5.2254 | 5.0497 | 5.1446 | 5.1146 | 5.0733 | 4.3124 | 4.2334 | 4.1176 | 5.033 | 5.0463 | 4.7241 | 4.7235 | 4.7565 |
| pos_20 | 3,7-Dihydroxy-12-oxocholanoic acid | metab_1037 | HMDB0000400 | - | 424.3066 | 6.2364 | pos | M+Na, 2M+Na, 2M+NH4, M+NH4, M+H-2H2O | C24H38O5 | 0 | 52 | 2.06460641 | 8/4/58 | 0.01231068 | 4.1483 | 5.0766 | 4.8551 | 5.3268 | 4.9578 | 5.3093 | 5.423 | 5.5069 | 5.5005 | 4.7617 | 5.2491 | 5.4206 | 6.0121 | 6.0205 | 6.0221 |
| pos_2004 | Capsidiol | metab_1043 | HMDB0002352;LMPR0103250002 | C09627 | 219.1749 | 6.0289 | pos | M+H-H2O | C15H24O2 | 0 | 68.5 | 2.51933073 | 37208-05-2 | 0.01133587 | 4.0674 | 4.1399 | 4.0723 | 4.1664 | 4.2942 | 4.2929 | 4.3627 | 4.3212 | 4.2042 | 4.4051 | 4.2992 | 4.4622 | 4.5213 | 4.5121 | 4.5136 |
| pos_2006 | NNAL-N-glucuronide | metab_1045 | HMDB0060498 | C19606 | 369.1528 | 6.0289 | pos | M+H-H2O | C16H24N3O8+ | 0 | 33.1 | -0.5451667 | - | 0.04184895 | 3.9654 | 4.3215 | 3.8567 | 4.0461 | 4.4638 | 4.0231 | 4.2208 | 3.8814 | 4.2522 | 4.2552 | 4.2826 | 4.2328 | 4.5067 | 4.5066 | 4.4748 |
| pos_2009 | Linalool (8-hydroxydihydro-) | metab_1048 | HMDB0304700 | C06740 | 633.2569 | 6.0344 | pos | M+H-H2O | C32H42O14 | 0 | 50.4 | 4.1521401 | - | 0.02365331 | 4.5702 | 3.9201 | 4.269 | 4.1273 | 4.4251 | 4.4714 | 4.5957 | 4.2655 | 3.8852 | 4.2863 | 4.1873 | 4.3372 | 4.9348 | 4.9163 | 4.918 |
| pos_2010 | Cinnamyl acetate | metab_1050 | HMDB0029699 | C12299 | 209.1178 | 6.0385 | pos | M+CH3OH+H | C11H12O2 | 0 | 39.5 | 3.07840124 | 21040-45-9;103-54-8 | 0.04911553 | 3.1206 | 3.5255 | 3.5595 | 3.5864 | 3.5186 | 3.3617 | 3.3209 | 3.1558 | 3.413 | 3.5353 | 3.6044 | 3.6784 | 3.8197 | 3.7823 | 3.7843 |
| pos_2012 | Cordycepin | metab_1052 | HMDB0250451;MJDBOTE0001321 | C08431 | 269.1366 | 6.0385 | pos | M+NH4 | C10H13N5O3 | 0 | 43.7 | 3.58693557 | 73-03-0 | 0.0123133 | 5.0271 | 5.0611 | 4.9658 | 5.1437 | 5.0338 | 5.0414 | 5.0564 | 4.9975 | 5.0937 | 4.9677 | 5.0771 | 5.1029 | 5.0988 | 5.0889 | 5.0904 |
| pos_2022 | 4-Methyl-1H-Benzotriazole | metab_1063 | - | - | 134.0718 | 6.0764 | pos | M+H | C7H7N3 | 94.3 | 0 | 3.8136884 | - | 0.00436246 | 5.5461 | 5.52 | 5.5462 | 5.5658 | 5.5137 | 5.5813 | 5.5385 | 5.5777 | 5.5639 | 5.6168 | 5.533 | 5.5268 | 5.595 | 5.5912 | 5.5929 |
| pos_2024 | Arg Met Asp | metab_1065 | - | - | 421.1845 | 6.0764 | pos | M+H | C15H28N6O6S | 65.4 | 0 | -4.4758287 | - | 0.01997009 | 4.0887 | 4.4071 | 4.212 | 4.1685 | 4.6344 | 4.4665 | 4.1933 | 4.1216 | 4.3275 | 4.0392 | 4.6144 | 4.6716 | 4.3265 | 4.3269 | 4.3416 |
| pos_2030 | Thienamycin | metab_1072 | HMDB0258982 | C06664 | 255.0772 | 6.101 | pos | M+H-H2O | C11H16N2O4S | 0 | 75.5 | -9.6758062 | 59995-64-1 | 0.00320717 | 3.9175 | 4.4905 | 4.1156 | 4.2486 | 4.8173 | 4.7286 | 4.4387 | 4.1487 | 4.7236 | 3.9978 | 4.5647 | 4.8866 | 4.3065 | 4.3093 | 4.3079 |
| pos_2032 | 16-Oxoestrone | metab_1074 | HMDB0000372;LMST02010048 | C14441 | 323.1034 | 6.101 | pos | M+K | C18H20O3 | 0 | 72.6 | -3.5313782 | 1228-73-5 | 0.06082318 | 3.9326 | 4.9764 | 4.8308 | 4.7195 | 4.6298 | 4.6244 | 4.7296 | 3.5163 | 3.3851 | 3.388 | 4.9296 | 4.9263 | 4.1133 | 4.1101 | 4.1566 |
| pos_2040 | Ovalicin | metab_1082 | HMDB0038120;HMDB0246031 | C09674 | 338.1946 | 6.117 | pos | M+ACN+H | C16H24O5 | 0 | 66.3 | -5.4366531 | 19683-98-8 | 0.01226036 | 4.605 | 4.1335 | 4.449 | 5.3672 | 3.9265 | 4.3083 | 4.6618 | 4.6639 | 4.167 | 4.1714 | 4.1947 | 4.4654 | 4.346 | 4.3457 | 4.3366 |
| pos_2042 | 3-(L-Menthoxy)-2-methylpropane-1,2-diol | metab_1084 | HMDB0032417 | - | 262.2383 | 6.125 | pos | M+NH4 | C14H28O3 | 0 | 42.5 | 2.60188113 | 195863-84-4 | 0.01213413 | 4.7272 | 5.0067 | 4.7157 | 4.7163 | 4.7576 | 4.9534 | 5.0238 | 4.7139 | 4.6361 | 4.7515 | 4.9764 | 4.8328 | 4.9806 | 4.9903 | 4.982 |
| pos_2045 | 7,10,13,16,19-Docosapentaenoic acid | metab_1087 | HMDB0247288;HMDB0246621;LMFA01030184;HMDB0006528 | C16513 | 353.248 | 6.125 | pos | M+Na | C22H34O2 | 0 | 68.3 | 8.73800117 | 24880-45-3 | 0.05360467 | 3.2954 | 3.2634 | 3.642 | 3.2347 | 3.2292 | 3.7671 | 3.6812 | 3.5291 | 3.769 | 3.2344 | 4.1177 | 3.8764 | 4.4739 | 4.4335 | 4.475 |
| pos_2047 | BioMed 101 | metab_1089 | HMDB0257533 | - | 502.2788 | 6.125 | pos | M+NH4 | C28H36O7 | 0 | 50.1 | -2.3965231 | - | 0.01361758 | 3.4877 | 4.8196 | 3.3615 | 3.5941 | 4.9902 | 5.2222 | 3.669 | 3.3782 | 5.1974 | 4.3112 | 5.2911 | 4.541 | 4.7093 | 4.7201 | 4.7107 |
| pos_2049 | Furanodiene | metab_1091 | HMDB0036769 | C16959 | 255.1135 | 6.133 | pos | M+K | C15H20O | 0 | 38.5 | -4.8915163 | 19912-61-9 | 0.00643277 | 3.2759 | 4.7324 | 3.7242 | 4.2761 | 3.2823 | 3.2592 | 2.9348 | 3.7402 | 3.1778 | 2.7398 | 3.6739 | 3.0301 | 4.1707 | 4.1705 | 4.1754 |
| pos_2051 | Arginyllysine | metab_1094 | HMDB0028714 | - | 335.2373 | 6.133 | pos | M+CH3OH+H | C12H26N6O3 | 0 | 57.2 | -9.2980812 | 40968-46-5 | 0.00581896 | 4.0333 | 4.1259 | 3.7733 | 3.696 | 4.0666 | 4.5238 | 3.9894 | 4.0281 | 4.4808 | 3.6696 | 4.7468 | 4.2 | 4.7228 | 4.7178 | 4.7194 |
| pos_2078 | (9S,10E,12S,13S)-9,12,13-Trihydroxyoctadec-10-enoylcarnitine | metab_1121 | HMDB0241532 | - | 456.3329 | 6.181 | pos | M+H-H2O | C25H47NO7 | 0 | 40.4 | 2.00474524 | - | 0.03032858 | 5.5918 | 5.5668 | 5.5967 | 5.5392 | 5.2738 | 5.0788 | 5.536 | 5.4022 | 5.4594 | 5.4546 | 5.4311 | 5.3869 | 5.2305 | 5.2538 | 5.2319 |
| pos_2086 | Misoprostol acid | metab_1130 | HMDB0254756 | - | 386.2908 | 6.2125 | pos | M+NH4 | C21H36O5 | 0 | 36.8 | 1.96943233 | - | 0.03997858 | 5.4158 | 5.0197 | 5.1233 | 4.9155 | 4.6846 | 4.9314 | 5.052 | 5.0736 | 5.0496 | 4.8348 | 5.1475 | 5.2752 | 5.0621 | 5.0324 | 5.0635 |
| pos_2087 | (E)-N-(3-(3-(4-Fluorophenoxy)phenyl)-1-(R,S)-methylprop-2-enyl)-N-hydroxyurea | metab_1131 | HMDB0249490 | - | 633.2567 | 6.2125 | pos | 2M+H | C17H17FN2O3 | 0 | 43.6 | 7.52892089 | - | 0.02829847 | 4.872 | 4.2444 | 5.3546 | 5.0054 | 4.2494 | 4.2662 | 5.1453 | 5.1401 | 4.4634 | 4.9558 | 4.2629 | 4.5206 | 5.2233 | 5.2026 | 5.2247 |
| pos_2089 | Xi-3-Methyl-3-cyclohexen-1-ol | metab_1133 | HMDB0034372 | - | 95.0864 | 6.2285 | pos | M+H-H2O | C7H12O | 0 | 35.3 | 7.50879764 | - | 0.00204987 | 5.1785 | 5.186 | 5.1818 | 5.1867 | 5.1532 | 5.2254 | 5.1941 | 5.2043 | 5.2264 | 5.1946 | 5.1892 | 5.1893 | 5.2683 | 5.268 | 5.2666 |
| pos_2091 | 4-Hydroxy-3-(3-methyl-2-butenyl)acetophenone | metab_1135 | HMDB0030770 | C10702 | 237.1468 | 6.2285 | pos | M+CH3OH+H | C13H16O2 | 0 | 37.9 | -8.2588564 | 26932-05-8 | 0.02909265 | 4.6924 | 4.6391 | 4.669 | 4.7561 | 4.632 | 4.6564 | 4.7767 | 4.6548 | 4.6529 | 4.772 | 4.6348 | 4.7152 | 4.753 | 4.752 | 4.7742 |
| pos_2094 | Daucol | metab_1138 | HMDB0035287 | C09652 | 271.2275 | 6.2285 | pos | M+CH3OH+H | C15H26O2 | 0 | 33.5 | 3.19296563 | 887-08-1 | 0.00270335 | 5.6845 | 5.638 | 5.6614 | 5.6981 | 5.611 | 5.6828 | 5.683 | 5.7058 | 5.6851 | 5.7178 | 5.6533 | 5.6659 | 5.7445 | 5.7422 | 5.7438 |
| pos_2101 | Lauroylcarnitine | metab_1145 | HMDB0002250;LMFA07070090 | - | 344.2802 | 6.2364 | pos | M+H | C19H37NO4 | 60.8 | 0 | 1.89840587 | 25518-54-1 | 0.00439097 | 5.2946 | 4.6839 | 4.354 | 4.2782 | 3.5912 | 3.8776 | 4.6782 | 4.5733 | 4.1573 | 4.7918 | 3.9104 | 3.9528 | 4.7941 | 4.7915 | 4.7904 |
| pos_2109 | 3a,6b,7a,12a-Tetrahydroxy-5b-cholanoic acid | metab_1152 | HMDB0000399 | C01094 | 389.2689 | 6.2443 | pos | M+H-2H2O | C24H40O6 | 0 | 55.2 | 0.56457191 | 80875-93-0 | 0.0167534 | 3.0279 | 4.6826 | 4.6038 | 4.6326 | 2.754 | 4.8408 | 4.9303 | 4.9461 | 4.8137 | 3.1502 | 5.0154 | 4.3304 | 5.4869 | 5.4986 | 5.5003 |
| pos_2120 | Neocnidolide | metab_1163 | HMDB0302242 | C17002 | 159.1172 | 6.2602 | pos | M+H-2H2O | C12H18O2 | 0 | 63.9 | 2.10267971 | - | 0.01231036 | 4.2161 | 4.5861 | 4.3895 | 4.2551 | 4.6525 | 4.634 | 4.324 | 4.2276 | 4.803 | 4.3092 | 4.6226 | 4.5407 | 4.5811 | 4.5894 | 4.5911 |
| pos_2137 | Indigo Carmine | metab_1181 | HMDB0059912;HMDB0240742 | - | 263.0821 | 6.2841 | pos | M+H | C16H10N2O2 | 80 | 0 | 2.32885184 | - | 0.06579862 | 4.2771 | 3.9528 | 4.0205 | 4.1517 | 3.4677 | 4.1811 | 4.0396 | 4.0567 | 4.0532 | 4.1896 | 3.9516 | 4.1207 | 4.1436 | 4.0941 | 4.096 |
| pos_2146 | Sphinganine | metab_1189 | HMDB0000269;LMSP01020001;PW_C000184;HMDB0242634 | C00836 | 302.306 | 6.2999 | pos | M+H | C18H39NO2 | 81 | 0 | 2.04489765 | 764-22-7 | 0.00062917 | 6.693 | 6.934 | 6.753 | 6.7884 | 6.8295 | 6.9806 | 6.8925 | 6.7695 | 6.7537 | 6.7993 | 6.9561 | 6.858 | 7.0312 | 7.031 | 7.0307 |
| pos_2147 | 15-hydroxyicosanoic acid | metab_1190 | HMDB0061665 | - | 346.3321 | 6.2999 | pos | M+NH4 | C20H40O3 | 0 | 79.6 | 1.64340735 | - | 0.00274065 | 5.0736 | 5.296 | 5.1476 | 5.1235 | 5.165 | 5.2999 | 5.1498 | 5.0813 | 5.1273 | 5.1788 | 5.2439 | 5.1702 | 5.7557 | 5.7534 | 5.7552 |
| pos_2148 | LysoPG(18:1(9Z)/0:0) | metab_1191 | HMDB0240602 | - | 552.331 | 6.2999 | pos | M+ACN+H | C24H47O9P | 0 | 55.2 | 2.79543762 | - | 0.00740968 | 5.1825 | 6.3167 | 6.2351 | 6.0809 | 5.6029 | 5.7467 | 5.8825 | 5.5429 | 6.1444 | 5.6235 | 5.844 | 5.7908 | 5.8917 | 5.8915 | 5.886 |
| pos_2149 | Bisacurone epoxide | metab_1192 | HMDB0038503 | - | 269.1727 | 6.3079 | pos | M+H | C15H24O4 | 0 | 76.7 | -7.4142491 | 127214-83-9 | 0.00449512 | 5.1306 | 5.1985 | 5.187 | 5.1685 | 5.115 | 5.1606 | 5.1407 | 5.1239 | 5.1368 | 5.1524 | 5.0708 | 5.1532 | 5.1842 | 5.1867 | 5.1881 |
| pos_2151 | Prostaglandin E1 | metab_1195 | LMFA03010134;HMDB0001442;HMDB0302206 | C04741 | 387.272 | 6.3079 | pos | M+CH3OH+H | C20H34O5 | 0 | 62.1 | -6.0269374 | 745-65-3 | 0.01339703 | 4.8035 | 5.1343 | 5.1659 | 5.0774 | 5.0032 | 5.0751 | 5.0607 | 5.0644 | 4.9343 | 5.0402 | 5.0371 | 5.036 | 5.2107 | 5.2099 | 5.2002 |
| pos_2155 | Cabergoline | metab_1199 | HMDB0014393 | C08187 | 474.2849 | 6.3158 | pos | M+Na | C26H37N5O2 | 0 | 31.6 | 2.21250709 | 81409-90-7 | 0.01381987 | 5.6371 | 5.6008 | 5.4892 | 5.6314 | 5.3983 | 5.5412 | 5.6126 | 5.634 | 5.5368 | 5.6127 | 5.5224 | 5.5953 | 5.522 | 5.533 | 5.5234 |
| pos_2157 | Delta-Valerolactone | metab_1201 | HMDB0250981 | C02240 | 83.05 | 6.3237 | pos | M+H-H2O | C5H8O2 | 0 | 79.6 | 8.69119445 | 542-28-9 | 0.01120422 | 4.3288 | 4.1644 | 4.0837 | 4.2073 | 4.0923 | 4.1041 | 4.0614 | 4.1471 | 4.2056 | 4.3108 | 4.1848 | 4.1406 | 4.2172 | 4.2167 | 4.2253 |
| pos_2161 | Taurochenodesoxycholic acid | metab_1206 | PW_C000759;HMDB0000951 | C05465 | 544.2693 | 6.3396 | pos | M+2Na-H | C26H45NO6S | 0 | 54.4 | 2.7521504 | 516-35-8 | 0.03018942 | 5.7112 | 5.3736 | 4.6448 | 4.6485 | 5.5921 | 5.4923 | 5.2327 | 5.2644 | 5.271 | 5.5959 | 5.301 | 5.562 | 5.2625 | 5.2624 | 5.285 |
| pos_2163 | 2-Aminopurine | metab_1208 | HMDB0245025 | - | 177.0887 | 6.3476 | pos | M+ACN+H | C5H5N5 | 0 | 38.5 | 3.08465197 | - | 0.00481977 | 5.6432 | 5.6681 | 5.6745 | 5.6832 | 5.6052 | 5.6805 | 5.672 | 5.6628 | 5.6605 | 5.7379 | 5.6103 | 5.6754 | 5.6741 | 5.6714 | 5.6756 |
| pos_2164 | Xi-4-Hydroxy-4-methyl-2-cyclohexen-1-one | metab_1209 | HMDB0033629 | - | 253.1414 | 6.3476 | pos | 2M+H | C7H10O2 | 0 | 62.6 | -8.1063631 | - | 0.00968026 | 4.934 | 4.9277 | 4.8704 | 4.8707 | 4.7862 | 4.9963 | 4.8623 | 4.8531 | 4.9102 | 4.9399 | 4.946 | 4.8632 | 4.947 | 4.9468 | 4.9541 |
| pos_2170 | Hydroxyoctadecenoylcarnitine | metab_1215 | HMDB0253282 | - | 424.3427 | 6.3554 | pos | M+H-H2O | C25H47NO5 | 0 | 66 | 1.32415254 | - | 0.01589411 | 5.4608 | 5.4692 | 5.4274 | 5.5085 | 5.4355 | 5.3012 | 5.3799 | 5.4019 | 5.2463 | 5.513 | 5.3258 | 5.4082 | 5.3623 | 5.3618 | 5.374 |
| pos_2179 | Pc(18:0/0:0) | metab_1222 | HMDB0010384;LMGP01050026 | C04230 | 524.3724 | 6.3712 | pos | M+H | C26H54NO7P | 75 | 0 | 2.50869175 | 19420-57-6 | 0.10727016 | 7.0099 | 7.1209 | 7.0589 | 7.0639 | 6.9707 | 7.0076 | 6.9716 | 7.099 | 7.0638 | 6.9818 | 6.9648 | 7.0394 | 5.5402 | 5.5382 | 5.6176 |
| pos_2187 | Panaxytriol | metab_1230 | LMFA05000027;HMDB0031928 | C16792 | 261.1855 | 6.3792 | pos | M+H-H2O | C17H26O3 | 0 | 38 | 1.99653075 | 87005-03-6 | 0.01574956 | 4.2533 | 4.675 | 4.6581 | 4.5756 | 4.6415 | 4.8373 | 4.7659 | 4.4706 | 4.7748 | 4.3872 | 4.5842 | 4.6456 | 4.9513 | 4.9511 | 4.963 |
| pos_2194 | 3,7R,11R,15-tetramethyl-hexadecanoic acid | metab_1238 | LMPR0104010004;HMDB0062520 | C01607 | 330.3374 | 6.3869 | pos | M+NH4 | C20H40O2 | 0 | 53.3 | 2.26613112 | - | 0.00928983 | 6.1859 | 6.3713 | 6.2098 | 6.2531 | 6.2851 | 6.4518 | 6.2977 | 6.2727 | 6.2322 | 6.3424 | 6.4256 | 6.3022 | 6.5892 | 6.589 | 6.5961 |
| pos_2196 | Tetracosahexaenoic acid | metab_1240 | PW_C001344;HMDB0002007;LMFA01030804 | - | 339.2692 | 6.3949 | pos | M+H-H2O | C24H36O2 | 0 | 80.6 | 2.72611482 | 81247-23-6 | 0.02838299 | 4.6861 | 5.2617 | 5.412 | 4.808 | 5.7261 | 5.6475 | 4.7984 | 5.2798 | 5.6084 | 4.9123 | 5.437 | 5.5617 | 5.2328 | 5.2537 | 5.2549 |
| pos_2200 | Trenbolone acetate | metab_1245 | HMDB0259120;HMDB0244801 | - | 402.3562 | 6.4029 | pos | 2M+NH4 | C12H20N2 | 0 | 46.2 | -7.6372116 | - | 0.00246926 | 5.9682 | 5.9755 | 5.9978 | 6.0406 | 5.9783 | 5.9866 | 5.9926 | 5.9985 | 5.9816 | 6.0204 | 5.9951 | 5.9399 | 6.043 | 6.041 | 6.0426 |
| pos_2208 | Dihydrozeatin | metab_1251 | HMDB0012215;MJDBZSJ0000131 | C02029 | 239.162 | 6.4266 | pos | M+NH4 | C10H15N5O | 0 | 58.8 | 2.40785054 | 23599-75-9;37789-32-5 | 0.0186852 | 5.0724 | 5.1736 | 5.1602 | 5.1743 | 5.1025 | 5.1389 | 5.1951 | 5.1738 | 5.216 | 5.2131 | 5.1511 | 5.1176 | 5.1479 | 5.1486 | 5.1622 |
| pos_2212 | 4-Heptenoic acid | metab_1256 | HMDB0033793;LMFA01030014 | - | 151.0731 | 6.4346 | pos | M+Na | C7H12O2 | 0 | 40.2 | 1.52257957 | 35194-37-7 | 0.01266314 | 4.7039 | 4.7413 | 4.7721 | 4.7031 | 4.6542 | 4.7199 | 4.7512 | 4.6486 | 4.7493 | 4.7926 | 4.7803 | 4.7493 | 4.7621 | 4.7533 | 4.7634 |
| pos_2232 | Artemorin | metab_1276 | HMDB0302701 | C09345 | 281.173 | 6.4744 | pos | M+CH3OH+H | C15H20O3 | 0 | 70.5 | -7.0290804 | 64845-92-7 | 0.02563383 | 5.0708 | 5.1493 | 5.3118 | 5.2473 | 5.008 | 5.3464 | 5.189 | 5.3106 | 5.3498 | 5.2318 | 5.19 | 5.2744 | 5.2775 | 5.2961 | 5.2977 |
| pos_2237 | Salviol | metab_1279 | HMDB0302947 | C21819 | 325.2127 | 6.4824 | pos | M+Na | C20H30O2 | 0 | 80.7 | -3.7402381 | - | 0.01065844 | 4.9932 | 5.0382 | 5.025 | 5.0698 | 4.9455 | 5.0229 | 5.0393 | 5.0587 | 5.0421 | 5.081 | 5.0245 | 5.0535 | 5.027 | 5.0184 | 5.0198 |
| pos_224 | (+/-)-1-(4-Methylphenyl)ethanol | metab_1282 | HMDB0034378 | - | 119.0862 | 0.6423 | pos | M+H-H2O, M+CH3OH+H | C9H12O | 0 | 58.7 | 5.2051275 | 5788-09-0 | 0.00122477 | 6.3987 | 6.4072 | 6.4169 | 6.4143 | 6.3657 | 6.4067 | 6.4268 | 6.4264 | 6.4126 | 6.4429 | 6.389 | 6.3913 | 6.4513 | 6.4511 | 6.4503 |
| pos_2244 | PC(15:0/20:4(8Z,11Z,14Z,17Z)) | metab_1287 | HMDB0007950;PW_C004000 | C00157 | 768.559 | 6.4984 | pos | M+H | C43H78NO8P | 0 | 66.3 | 6.73833364 | - | 0.0328253 | 3.904 | 4.6457 | 4.3745 | 4.839 | 4.1379 | 4.4523 | 5.208 | 4.9622 | 4.5444 | 4.0791 | 5.1845 | 4.9464 | 5.6963 | 5.6874 | 5.6682 |
| pos_2249 | Polygonal | metab_1292 | HMDB0256674 | - | 223.1698 | 6.5143 | pos | M+H | C14H22O2 | 0 | 30.2 | 2.63994325 | - | 0.00125724 | 6.6209 | 6.6179 | 6.6307 | 6.6594 | 6.5148 | 6.6243 | 6.6332 | 6.64 | 6.6213 | 6.6728 | 6.5865 | 6.5802 | 6.6616 | 6.6614 | 6.6606 |
| pos_225 | Gentisate aldehyde | metab_1293 | HMDB0004062 | C05585 | 180.0661 | 0.6583 | pos | M+H, M+ACN+H | C7H6O3 | 0 | 32.9 | 4.2428642 | 1194-98-5 | 0.00333378 | 5.5203 | 5.5205 | 5.5216 | 5.5311 | 5.4844 | 5.5473 | 5.5366 | 5.5469 | 5.5316 | 5.5541 | 5.5003 | 5.512 | 5.5413 | 5.5426 | 5.5442 |
| pos_2258 | 1-Methyl-1,3-cyclohexadiene | metab_1300 | HMDB0031532 | - | 95.0864 | 6.53 | pos | M+H | C7H10 | 0 | 30.8 | 8.87595012 | 1489-56-1 | 0.00324214 | 4.6282 | 4.6989 | 4.7024 | 4.7301 | 4.5288 | 4.7053 | 4.693 | 4.7001 | 4.7138 | 4.7219 | 4.6252 | 4.661 | 4.8517 | 4.8521 | 4.8495 |
| pos_2260 | 3-Hydroxysebacic Acid | metab_1303 | HMDB0000350 | - | 201.1127 | 6.5379 | pos | M+H-H2O | C10H18O5 | 67.8 | 0 | 2.65825043 | 446881-43-2 | 0.01505062 | 4.4761 | 4.5337 | 4.5739 | 4.5648 | 4.3488 | 4.5274 | 4.5771 | 4.5875 | 4.6101 | 4.5885 | 4.5255 | 4.4287 | 4.5694 | 4.5798 | 4.5816 |
| pos_2263 | PE(16:1(9Z)/P-18:1(11Z)) | metab_1306 | HMDB0008984 | C00350 | 722.5067 | 6.5379 | pos | M+Na | C39H74NO7P | 0 | 38.6 | -3.953024 | - | 0.01168801 | 5.7139 | 5.7649 | 5.7528 | 5.7061 | 5.6574 | 5.7793 | 5.7178 | 5.6995 | 5.7609 | 5.7201 | 5.728 | 5.6471 | 5.8182 | 5.8276 | 5.8197 |
| pos_2265 | Caprylic acid | metab_1308 | HMDB0000482;HMDB0304443;HMDB0303345;PW_C000366;LMFA01010008 | C06423 | 109.1019 | 6.5459 | pos | M+H-2H2O | C8H16O2 | 0 | 36.9 | 4.97815493 | 124-07-2;74-81-7;124-07-2 | 0.01079769 | 4.547 | 4.5344 | 4.582 | 4.6683 | 4.4348 | 4.5359 | 4.6019 | 4.5613 | 4.6065 | 4.5875 | 4.4973 | 4.5333 | 4.705 | 4.7045 | 4.6966 |
| pos_2268 | PE(18:0/TXB2) | metab_1311 | HMDB0261128 | - | 847.5525 | 6.5459 | pos | 2M+3H2O+2H | C43H80NO12P | 0 | 81.2 | -1.0813219 | - | 0.01483199 | 5.9198 | 5.3823 | 5.6499 | 4.6102 | 6.0004 | 5.6083 | 5.3551 | 5.6426 | 5.6559 | 5.7885 | 5.6579 | 5.4755 | 5.9749 | 5.9645 | 5.9763 |
| pos_2270 | 19(S)-HETE | metab_1314 | PW_C006775;HMDB0011136 | C14749 | 303.2325 | 6.5539 | pos | M+H-H2O | C20H32O3 | 0 | 75.1 | 1.96195181 | 79551-85-2 | 0.00815974 | 5.4064 | 4.9773 | 4.7198 | 5.3963 | 4.287 | 4.8107 | 5.1421 | 5.3488 | 4.5063 | 4.6173 | 4.3006 | 4.9412 | 5.1036 | 5.0966 | 5.1011 |
| pos_2273 | PE(18:3(9Z,12Z,15Z)/16:0) | metab_1317 | PW_C005202;HMDB0009154;LMGP02010047 | C00350 | 678.4803 | 6.5539 | pos | M+H-2H2O | C39H72NO8P | 0 | 36 | -7.5689265 | - | 0.01015847 | 5.9133 | 5.9925 | 5.9765 | 6.0022 | 5.9025 | 6.009 | 5.9838 | 6.0197 | 6.0217 | 6.0169 | 5.9217 | 5.945 | 6.0464 | 6.0531 | 6.0548 |
| pos_2285 | CL(i-12:0/i-22:0/18:2(9Z,11Z)/18:2(9Z,11Z)) | metab_1329 | HMDB0089074;PW_C087674 | C05980 | 737.4866 | 6.5699 | pos | M+2Na | C79H146O17P2 | 0 | 50.7 | -6.1185841 | - | 0.0105162 | 5.595 | 5.2167 | 5.3652 | 4.9225 | 5.6626 | 5.4435 | 5.3533 | 5.409 | 5.4817 | 5.5621 | 5.5147 | 5.3356 | 5.9883 | 5.9881 | 5.9961 |
| pos_230 | Pipecolic Acid | metab_1345 | HMDB0000070;HMDB0000716;MJDBOTE0000664 | C00408 | 147.1133 | 0.7463 | pos | M+H, M+NH4 | C6H11NO2 | 64.3 | 0 | 3.46348757 | 3105-95-1;535-75-1 | 0.00556476 | 5.7476 | 5.7078 | 5.6856 | 5.6098 | 5.5582 | 5.4313 | 5.7728 | 5.5849 | 5.4569 | 5.7548 | 5.3807 | 5.5856 | 5.5119 | 5.5085 | 5.5133 |
| pos_2311 | Oryzalexin D | metab_1358 | HMDB0302102 | C21562 | 337.2743 | 6.6099 | pos | M+CH3OH+H | C20H32O2 | 0 | 55.5 | 1.886985 | 110268-98-9 | 0.00592927 | 5.4305 | 5.4822 | 5.5522 | 5.5071 | 5.296 | 5.4732 | 5.4417 | 5.4602 | 5.4523 | 5.3874 | 5.3722 | 5.3492 | 5.5485 | 5.5535 | 5.5499 |
| pos_2320 | Parasorbic acid | metab_1366 | HMDB0301801 | C08502 | 95.05 | 6.6259 | pos | M+H-H2O | C6H8O2 | 0 | 34.9 | 7.43909601 | 10048-32-5 | 0.04459411 | 4.0936 | 4.1103 | 4.1612 | 4.3146 | 3.8387 | 4.1104 | 4.2407 | 4.2891 | 4.1239 | 4.2799 | 4.1182 | 4.0535 | 4.3474 | 4.3807 | 4.3821 |
| pos_2324 | PE(15:0/PGF1alpha) | metab_1370 | HMDB0260874 | - | 791.5259 | 6.6259 | pos | 2M+3H2O+2H | C40H76NO11P | 0 | 32.7 | -1.6255872 | - | 0.05240789 | 6.0928 | 5.9027 | 5.9876 | 5.6377 | 6.2046 | 5.9749 | 5.8225 | 5.9665 | 5.9876 | 6.0559 | 6.0145 | 5.9346 | 5.9844 | 5.9839 | 5.9441 |
| pos_2333 | Pristimerin | metab_1379 | HMDB0256776;MJDBOTE0000261 | C08633 | 506.3254 | 6.6419 | pos | M+ACN+H | C30H40O4 | 0 | 41.1 | -2.3944565 | 1258-84-0 | 0.00585815 | 4.7137 | 5.2748 | 5.165 | 5.2708 | 4.8837 | 5.0729 | 5.1623 | 5.1374 | 5.2306 | 5.0694 | 4.987 | 5.0856 | 5.3924 | 5.3958 | 5.3974 |
| pos_2338 | Polidocanol | metab_1383 | HMDB0244942;HMDB0249382;HMDB0256665;HMDB0251574 | C13493 | 600.4693 | 6.6499 | pos | M+NH4 | C30H62O10 | 0 | 40.4 | 2.02197769 | 3055-99-0 | 0.01202972 | 5.3779 | 5.2117 | 5.3157 | 5.5869 | 5.4119 | 5.4004 | 5.2159 | 5.3108 | 5.3003 | 5.2168 | 5.1827 | 5.1509 | 5.3816 | 5.3722 | 5.3731 |
| pos_2341 | 20-Hydroxy-6Z,15Z-eicosadienoic acid | metab_1387 | HMDB0245564;LMFA03000010 | - | 307.2638 | 6.6579 | pos | M+H-H2O | C20H36O3 | 0 | 36.7 | 1.84271243 | - | 0.00546395 | 4.6632 | 4.2469 | 4.1939 | 3.6067 | 4.7704 | 4.5067 | 3.867 | 4.2882 | 4.4604 | 4.6083 | 4.2707 | 3.6088 | 4.9726 | 4.9694 | 4.9741 |
| pos_2351 | Heptaethylene glycol monododecyl ether | metab_1398 | HMDB0251574 | - | 512.4169 | 6.6739 | pos | M+NH4 | C26H54O8 | 0 | 33.2 | 2.37223531 | - | 0.00698587 | 5.0439 | 5.0751 | 5.3272 | 5.5634 | 5.4433 | 5.4205 | 5.1757 | 5.0223 | 5.2949 | 4.902 | 5.0447 | 5.1457 | 5.4423 | 5.4423 | 5.437 |
| pos_2352 | CL(i-12:0/i-22:0/i-16:0/i-12:0) | metab_1399 | PW_C087859;HMDB0088999 | C05980 | 685.4627 | 6.6739 | pos | M+2Na | C71H138O17P2 | 0 | 58.3 | 4.59623441 | - | 0.03420119 | 6.173 | 5.8644 | 5.822 | 5.4775 | 6.2304 | 5.9439 | 5.7648 | 5.9262 | 6.0319 | 6.1844 | 6.0597 | 5.8851 | 5.8407 | 5.8147 | 5.8157 |
| pos_2374 | 13,16,19-Docosatrienoic acid | metab_1421 | HMDB0002823;LMFA01030407;HMDB0244522 | C16534 | 352.3216 | 6.7138 | pos | M+NH4 | C22H38O2 | 0 | 80.5 | 1.77121231 | 28845-86-5 | 0.01013591 | 5.4671 | 5.4016 | 5.4793 | 5.4875 | 5.2432 | 5.4088 | 5.4845 | 5.4499 | 5.3495 | 5.4481 | 5.3627 | 5.3602 | 5.5461 | 5.5528 | 5.5545 |
| pos_2391 | PE-NMe2(20:1(11Z)/14:1(9Z)) | metab_1439 | HMDB0114211;PW_C060228 | C04308 | 744.5563 | 6.7298 | pos | M+H | C41H78NO8P | 0 | 71.6 | 3.3968935 | - | 0.0285851 | 6.006 | 6.7823 | 6.3716 | 6.8052 | 6.4542 | 6.4424 | 6.8431 | 6.8717 | 6.5629 | 6.5101 | 6.8724 | 6.5973 | 6.7213 | 6.7059 | 6.6969 |
| pos_2392 | PC(20:3(8Z,11Z,14Z)/16:1(9Z)) | metab_1440 | LMGP01011873;PW_C004446;HMDB0008398 | C00157 | 782.5708 | 6.7298 | pos | M+H | C44H80NO8P | 0 | 50.6 | 1.75597047 | - | 0.02431135 | 6.8957 | 7.7072 | 7.7312 | 7.6937 | 7.4425 | 7.6733 | 7.7905 | 7.7703 | 7.7432 | 7.8154 | 7.6892 | 7.6915 | 7.6898 | 7.6703 | 7.6733 |
| pos_2409 | PE(20:3(8Z,11Z,14Z)-2OH(5,6)/24:1(15Z)) | metab_1458 | HMDB0284589 | - | 916.6592 | 6.7618 | pos | M+CH3OH+H | C49H90NO10P | 0 | 48.5 | -5.1747442 | - | 0.01430649 | 6.2335 | 5.9164 | 6.0333 | 5.6882 | 6.4088 | 6.0827 | 5.8586 | 6.0787 | 6.2014 | 6.342 | 6.2479 | 5.9966 | 6.0987 | 6.0984 | 6.1092 |
| pos_2410 | Ethyl-4-hydroxymethyl-3(2H)-Furanone | metab_1460 | - | - | 107.0498 | 6.7698 | pos | M+H-2H2O | C7H10O3 | 0 | 49.7 | 4.74352318 | - | 0.02448065 | 4.3676 | 4.3197 | 4.3213 | 4.3613 | 4.2064 | 4.2567 | 4.2418 | 4.3223 | 4.2618 | 4.2753 | 4.2743 | 4.2292 | 4.3365 | 4.3363 | 4.3547 |
| pos_2425 | PC(18:4(6Z,9Z,12Z,15Z)/20:1(11Z)) | metab_1475 | PW_C004290;HMDB0008242;LMGP01011720 | C00157 | 808.5869 | 6.7858 | pos | M+H | C46H82NO8P | 0 | 90 | 2.21313012 | - | 0.1327186 | 5.5373 | 7.2278 | 7.2039 | 7.1602 | 6.8724 | 7.1761 | 7.3397 | 7.291 | 7.2321 | 7.293 | 7.1426 | 7.2394 | 7.0357 | 6.9338 | 7.0404 |
| pos_2426 | PE(20:3(8Z,11Z,14Z)/20:1(11Z)) | metab_1476 | PW_C005410;HMDB0009362;LMGP02010922 | C00350 | 828.6061 | 6.7858 | pos | M+CH3OH+H | C45H82NO8P | 0 | 60.1 | -6.5208732 | - | 0.00353296 | 6.481 | 6.1942 | 6.2959 | 6.019 | 6.6727 | 6.3531 | 6.1857 | 6.3114 | 6.4321 | 6.565 | 6.4316 | 6.3259 | 6.3994 | 6.4025 | 6.4008 |
| pos_2429 | LysoPE(P-18:0/0:0) | metab_1479 | HMDB0240598 | - | 488.3122 | 6.7938 | pos | M+Na | C23H48NO6P | 0 | 74.6 | 2.20565492 | 174062-73-8 | 0.02437648 | 4.5633 | 4.4372 | 4.5759 | 4.8356 | 4.2437 | 4.4742 | 4.6135 | 4.1468 | 4.6266 | 4.6453 | 4.0932 | 3.7606 | 5.7251 | 5.7427 | 5.7444 |
| pos_2435 | Choline Phosphate | metab_1485 | HMDB0001565 | C00588 | 184.0738 | 6.8178 | pos | M+H | C5H14NO4P | 56.8 | 0 | 2.35790951 | 3616-04-4;107-73-3 | 0.00684715 | 6.1544 | 6.1947 | 6.1637 | 6.1811 | 6.0464 | 6.1108 | 6.1803 | 6.1853 | 6.1718 | 6.0968 | 6.1123 | 6.1175 | 6.1319 | 6.1317 | 6.1267 |
| pos_2437 | PE-NMe(20:1(11Z)/16:1(9Z)) | metab_1487 | HMDB0113312;PW_C059436 | C01241 | 740.5579 | 6.8178 | pos | M+H-H2O | C42H80NO8P | 0 | 49 | -1.2780911 | - | 0.05952119 | 6.5294 | 6.5211 | 6.2959 | 5.9941 | 6.6536 | 6.2968 | 6.1169 | 6.2499 | 6.3257 | 6.4794 | 6.3767 | 6.2171 | 6.5979 | 6.5928 | 6.55 |
| pos_2439 | Flavidulol A | metab_1489 | HMDB0039152 | - | 534.3566 | 6.8258 | pos | 2M+NH4 | C17H22O2 | 0 | 31.3 | -2.3321435 | 117568-32-8 | 0.01254705 | 4.3214 | 5.767 | 5.6638 | 5.5259 | 5.3444 | 5.6384 | 5.4406 | 5.4409 | 5.7361 | 5.5403 | 4.9535 | 5.1062 | 5.4683 | 5.4678 | 5.4586 |
| pos_2453 | Sclareol | metab_1503 | LMPR0104030010;HMDB0036827;MJDBOTE0001479 | C09183 | 326.306 | 6.8498 | pos | M+NH4 | C20H36O2 | 0 | 40.3 | 2.02986042 | 515-03-7 | 0.02489087 | 4.3069 | 4.3782 | 4.3998 | 4.541 | 4.3973 | 4.3818 | 4.7265 | 4.4717 | 4.2454 | 4.6516 | 4.2064 | 4.1958 | 4.9668 | 4.9859 | 4.9679 |
| pos_2456 | PE-NMe2(18:3(9Z,12Z,15Z)/18:0) | metab_1506 | HMDB0114128;PW_C060145 | C04308 | 770.5715 | 6.8498 | pos | M+H | C43H80NO8P | 0 | 44.3 | 2.68170433 | - | 0.09412749 | 4.501 | 6.48 | 5.268 | 6.4865 | 5.9 | 5.9157 | 6.3592 | 6.4354 | 6.136 | 6.144 | 6.4439 | 6.1827 | 6.3456 | 6.3458 | 6.2727 |
| pos_2457 | 6-methyl-5-Hepten-2-one | metab_1507 | HMDB0035915;LMFA12000039 | C07287 | 127.1122 | 6.8578 | pos | M+H | C8H14O | 54.6 | 0 | 3.82896299 | 110-93-0 | 0.01448021 | 4.5474 | 4.5082 | 4.4676 | 4.558 | 4.3614 | 4.5122 | 4.501 | 4.5289 | 4.4905 | 4.595 | 4.5379 | 4.4691 | 4.5705 | 4.5805 | 4.5822 |
| pos_2466 | (4E,7E,10E,13E)-Hexadeca-4,7,10,13-tetraenoic acid | metab_1516 | HMDB0253125 | - | 560.3722 | 6.8738 | pos | 2M+ACN+Na | C16H24O2 | 0 | 43 | 2.39410372 | - | 0.01637711 | 4.8424 | 5.7143 | 5.3153 | 5.5885 | 5.5749 | 5.6343 | 5.553 | 5.3693 | 5.6556 | 5.4253 | 5.2966 | 5.4317 | 5.3243 | 5.3242 | 5.3365 |
| pos_2470 | 4,5-Dihydrovomifoliol | metab_1521 | HMDB0040615 | - | 244.1913 | 6.8897 | pos | M+NH4 | C13H22O3 | 0 | 38.1 | 2.43084852 | 142173-08-8 | 0.06768621 | 3.5345 | 3.5071 | 3.9144 | 3.5991 | 3.503 | 3.5469 | 4.0818 | 3.774 | 3.7205 | 4.3304 | 3.8074 | 3.6012 | 4.3991 | 4.3949 | 4.4469 |
| pos_2472 | 3-Hydroxydodecanedioic acid | metab_1523 | HMDB0000413 | - | 229.144 | 6.8976 | pos | M+H-H2O | C12H22O5 | 0 | 60.5 | 2.46375863 | 34574-69-1 | 0.04680386 | 3.9536 | 4.1576 | 3.9273 | 3.8542 | 3.6793 | 3.8744 | 3.3821 | 4.2041 | 4.1655 | 4.2449 | 3.9675 | 3.9358 | 4.1604 | 4.1588 | 4.1239 |
| pos_2476 | Contignasterol | metab_1527 | HMDB0250439 | C19902 | 1017.6903 | 6.9056 | pos | 2M+H | C29H48O7 | 0 | 39.8 | 3.00816467 | 137571-30-3 | 0.01069583 | 5.9845 | 6.146 | 6.1783 | 6.1314 | 6.0336 | 6.0185 | 6.1398 | 6.0672 | 6.1488 | 5.9148 | 6.1369 | 6.0798 | 5.9566 | 5.9566 | 5.9646 |
| pos_2482 | P-Coumaraldehyde | metab_1534 | HMDB0040986 | C05608 | 181.0864 | 6.9295 | pos | M+CH3OH+H | C9H8O2 | 0 | 52.1 | 2.99554613 | 20711-53-9;2538-87-6 | 0.00948433 | 4.4425 | 4.3903 | 4.3641 | 4.2852 | 4.2827 | 4.3389 | 4.2421 | 4.2982 | 4.4185 | 4.5373 | 4.2641 | 4.1714 | 4.4916 | 4.4985 | 4.499 |
| pos_2484 | 1,4-Ipomeadiol | metab_1536 | HMDB0030471 | - | 153.0914 | 6.9375 | pos | M+H-H2O | C9H14O3 | 0 | 64.1 | 2.5742212 | 53011-73-7 | 0.02098086 | 4.3873 | 4.3603 | 4.3219 | 4.3271 | 4.2245 | 4.3362 | 4.2997 | 4.3648 | 4.3596 | 4.497 | 4.2613 | 4.2348 | 4.4625 | 4.4778 | 4.4617 |
| pos_2492 | PC(14:1(9Z)/20:0) | metab_1545 | PW_C003961;LMGP01011397;HMDB0007911 | C00157 | 742.5716 | 6.9533 | pos | M+H-H2O | C42H82NO8P | 0 | 48.7 | -3.8381285 | - | 0.10114286 | 5.4716 | 3.913 | 4.7534 | 3.9246 | 4.7584 | 4.602 | 4.1385 | 4.384 | 4.4705 | 4.6763 | 4.3897 | 3.9266 | 5.4633 | 5.5276 | 5.4461 |
| pos_2503 | LysoPC(20:2(11Z,14Z)/0:0) | metab_1557 | HMDB0010392 | C04230 | 548.3724 | 6.9773 | pos | M+H | C28H54NO7P | 0 | 86.3 | 2.43142137 | - | 0.00365224 | 5.8433 | 6.1835 | 6.1829 | 6.1188 | 5.9624 | 6.0276 | 6.1076 | 6.0656 | 6.1716 | 6.0915 | 5.8284 | 5.9251 | 6.2216 | 6.2247 | 6.2239 |
| pos_2507 | SM(d18:0/16:1(9Z)) | metab_1561 | HMDB0013464 | C00550 | 703.5768 | 6.9853 | pos | M+H | C39H79N2O6P | 0 | 38.3 | 2.76053524 | - | 0.06052933 | 3.6102 | 6.1805 | 5.8085 | 5.6714 | 5.8051 | 5.7937 | 6.0665 | 6.0122 | 5.9512 | 6.1157 | 5.9796 | 5.8164 | 5.8974 | 5.8576 | 5.8487 |
| pos_2509 | PE(PGJ2/24:0) | metab_1563 | HMDB0284475 | - | 895.6177 | 6.9853 | pos | 2M+3H2O+2H | C49H88NO10P | 0 | 63.3 | -9.5706426 | - | 0.02796819 | 5.9858 | 5.8779 | 5.9219 | 6.0253 | 6.1889 | 6.066 | 5.9195 | 6.0979 | 5.9833 | 6.1016 | 6.001 | 6.0115 | 5.9841 | 5.984 | 5.9629 |
| pos_2513 | PC(18:0/PGF2alpha) | metab_1568 | HMDB0286237 | - | 873.604 | 6.9933 | pos | 2M+3H2O+2H | C46H86NO11P | 0 | 66.7 | -1.6627234 | - | 0.00730132 | 5.8981 | 5.7115 | 5.8187 | 5.9865 | 6.0735 | 5.9895 | 5.8197 | 5.918 | 5.8808 | 5.9977 | 5.9275 | 5.9593 | 5.9155 | 5.9205 | 5.9214 |
| pos_2517 | PC(14:1(9Z)/18:0) | metab_1570 | PW_C003954;HMDB0007904;LMGP01011389 | C00157 | 732.5545 | 7.0013 | pos | M+H | C40H78NO8P | 0 | 33.6 | 0.96658974 | - | 0.06573419 | 6.3622 | 6.6695 | 6.4607 | 6.7976 | 6.7362 | 6.8064 | 6.7016 | 6.7688 | 6.6032 | 6.7567 | 6.7359 | 6.6982 | 6.6256 | 6.5761 | 6.578 |
| pos_2550 | Janthitrem B | metab_1603 | HMDB0030528 | C20600 | 568.3388 | 7.0491 | pos | M+H-H2O | C37H47NO5 | 0 | 46.8 | -5.747504 | 73561-90-7 | 0.05243187 | 4.6475 | 4.7827 | 4.5574 | 5.0526 | 4.5229 | 4.1394 | 4.5401 | 4.6427 | 4.5302 | 4.7007 | 3.7279 | 4.4893 | 5.1418 | 5.1815 | 5.1823 |
| pos_2601 | Alpha-hydroxyalprazolam | metab_1658 | HMDB0013943 | - | 687.1157 | 7.1209 | pos | 2M+K | C17H13ClN4O | 0 | 41.9 | -4.7540545 | 37115-43-8 | 0.02248062 | 5.379 | 5.3469 | 5.411 | 5.5249 | 5.5504 | 5.5961 | 5.3121 | 5.5169 | 5.3522 | 5.4818 | 5.5325 | 5.5152 | 5.561 | 5.5446 | 5.5623 |
| pos_2612 | GNRH, (Ac-dehydro-pro(1)-4-Cl-phe(2)-trp(3,6))-N-(alpha)-meleu(7)- | metab_1670 | HMDB0252904 | - | 698.3229 | 7.137 | pos | M+2H | C70H87ClN16O13 | 0 | 40.2 | -0.9735193 | - | 0.04614355 | 5.6779 | 5.7488 | 5.7764 | 5.8219 | 5.9028 | 5.8816 | 5.7645 | 5.8931 | 5.7429 | 5.7949 | 5.9156 | 5.9193 | 5.9224 | 5.9239 | 5.888 |
| pos_2618 | 15-Octadecene-9,11,13-triynoic acid | metab_1676 | LMFA01030559;HMDB0032673 | - | 558.3542 | 7.145 | pos | 2M+NH4 | C18H22O2 | 0 | 48 | -6.6079152 | - | 0.00457156 | 5.2363 | 5.4974 | 5.2182 | 5.4614 | 4.8489 | 5.0773 | 5.3777 | 5.1028 | 5.1706 | 5.3605 | 4.7411 | 4.8995 | 5.3085 | 5.3125 | 5.31 |
| pos_2624 | PS(24:0/22:5(4Z,7Z,10Z,13Z,16Z)) | metab_1683 | HMDB0112905;PW_C061347 | C02737 | 935.6485 | 7.145 | pos | 2M+3H2O+2H | C52H92NO10P | 0 | 67.5 | -9.6867894 | - | 0.02950774 | 5.8262 | 5.8535 | 5.8918 | 5.864 | 5.9466 | 5.8523 | 5.8026 | 5.805 | 5.9681 | 5.9718 | 5.9668 | 5.8794 | 5.8995 | 5.9016 | 5.9225 |
| pos_264 | N-Methyl-D-Aspartic Acid | metab_1699 | HMDB0002393;MJDBOTE0000848 | C12269 | 148.0609 | 1.0563 | pos | M+H-H2O, M+H | C5H9NO4 | 59 | 0 | 3.06328522 | 6384-92-5 | 0.0027371 | 6.0761 | 5.9407 | 5.9266 | 5.9023 | 6.078 | 6.0398 | 5.9593 | 5.9163 | 5.9827 | 5.9337 | 6.0043 | 6.1778 | 6.0071 | 6.0078 | 6.0094 |
| pos_2650 | MG(0:0/18:3(6Z,9Z,12Z)/0:0) | metab_1711 | HMDB0011539 | - | 385.2953 | 7.184 | pos | M+CH3OH+H | C21H36O4 | 0 | 30.1 | 1.21956721 | - | 0.10313603 | 3.7959 | 3.4659 | 3.8266 | 3.6763 | 3.5037 | 3.2068 | 3.6957 | 3.1539 | 3.8529 | 3.6715 | 3.1644 | 3.1594 | 3.3963 | 3.47 | 3.3929 |
| pos_2679 | PC(20:2(11Z,14Z)/18:1(11Z)) | metab_1742 | LMGP01012190;PW_C004382;HMDB0008334 | C00157 | 812.617 | 7.2257 | pos | M+H | C46H86NO8P | 0 | 78.1 | 0.71870497 | - | 0.26661306 | 5.9534 | 5.0893 | 6.206 | 5.769 | 5.2391 | 5.5052 | 5.7842 | 5.5993 | 5.5028 | 5.7212 | 5.8027 | 5.72 | 5.9588 | 5.9576 | 5.7362 |
| pos_269 | Arabinofuranosylcytosine | metab_1753 | HMDB0248553;HMDB0015122 | C02961 | 266.0753 | 1.1355 | pos | M+H, M+Na | C9H13N3O5 | 0 | 57.9 | 2.47713644 | 147-94-4 | 0.00944152 | 5.1087 | 5.2423 | 5.0958 | 4.9752 | 5.2884 | 5.216 | 5.1955 | 5.0679 | 5.2608 | 5.0949 | 5.1121 | 5.2406 | 5.1429 | 5.1491 | 5.1507 |
| pos_2690 | CL(i-12:0/i-24:0/a-25:0/18:2(9Z,11Z))[rac] | metab_1754 | HMDB0089529 | - | 791.5919 | 7.2468 | pos | M+H+Na | C88H168O17P2 | 0 | 33.1 | 7.51404221 | - | 0.01001448 | 6.4387 | 6.4311 | 6.4933 | 6.5681 | 6.6756 | 6.5866 | 6.4784 | 6.6111 | 6.449 | 6.5419 | 6.6402 | 6.531 | 6.635 | 6.6268 | 6.6285 |
| pos_2696 | Isoetharine | metab_1760 | HMDB0014366 | C07053 | 542.3228 | 7.2549 | pos | 2M+ACN+Na | C13H21NO3 | 0 | 62.1 | 5.71815614 | 530-08-5 | 0.01451775 | 5.0945 | 5.1604 | 5.0339 | 4.9647 | 4.6667 | 4.8265 | 4.9995 | 4.7355 | 4.8837 | 4.7058 | 4.5401 | 4.7569 | 4.8748 | 4.8748 | 4.8857 |
| pos_2701 | Cer(d20:1/20:4(6E,8Z,11Z,14Z)-OH(5S)) | metab_1767 | HMDB0290100 | - | 674.5049 | 7.2628 | pos | M+2Na-H | C40H71NO4 | 0 | 31.6 | -7.3294215 | - | 0.01529378 | 5.1539 | 5.0297 | 4.9822 | 5.1435 | 5.291 | 5.2059 | 5.0315 | 5.1903 | 4.9631 | 5.1062 | 5.2582 | 5.1898 | 5.2482 | 5.2481 | 5.2596 |
| pos_272 | L-Methionine | metab_1787 | HMDB0033951;HMDB0000696;PW_C000548;MJDBOTE0001368 | C00073 | 150.0589 | 1.1753 | pos | M+H, M+Na | C5H11NO2S | 63.3 | 0 | 3.5292094 | 63-68-3;59-51-8 | 0.00650807 | 5.8918 | 6.0893 | 5.9905 | 5.8422 | 6.0045 | 5.9512 | 6.0957 | 5.8796 | 5.8883 | 5.9848 | 5.869 | 6.1136 | 6.0493 | 6.0547 | 6.0507 |
| pos_2732 | 3a,12b-Dihydroxy-5b-cholanoic acid | metab_1800 | HMDB0000411 | C17646 | 826.619 | 7.3042 | pos | 2M+ACN+H | C24H40O4 | 0 | 33.5 | -0.1774314 | 570-62-7 | 0.00846264 | 6.2996 | 6.2493 | 6.2925 | 6.3696 | 6.4665 | 6.3739 | 6.2103 | 6.4194 | 6.188 | 6.3656 | 6.3535 | 6.3091 | 6.4225 | 6.4169 | 6.4239 |
| pos_2742 | Diisobutyl phthalate | metab_1810 | HMDB0013835 | C15205 | 311.1831 | 7.3202 | pos | M+CH3OH+H | C16H22O4 | 0 | 31 | -7.7821773 | 84-69-5 | 0.04188939 | 4.7025 | 4.9742 | 4.722 | 4.4782 | 4.1229 | 4.5471 | 4.7572 | 4.3802 | 4.7563 | 4.6354 | 4.498 | 4.5055 | 4.4835 | 4.4832 | 4.5145 |
| pos_2769 | Floionolic acid | metab_1839 | HMDB0034295;LMFA02000147 | C19621 | 315.2536 | 7.3757 | pos | M+H-H2O | C18H36O5 | 0 | 83.5 | 1.9543468 | 17705-68-9 | 0.00484768 | 6.858 | 6.8884 | 6.8526 | 6.8498 | 6.7253 | 6.8486 | 6.8536 | 6.8105 | 6.8963 | 6.8953 | 6.7894 | 6.8138 | 6.8813 | 6.8838 | 6.8855 |
| pos_2777 | Octylamine | metab_1848 | HMDB0255916 | C01740 | 130.1595 | 7.3917 | pos | M+H | C8H19N | 0 | 30.4 | 4.00491054 | 111-86-4 | 0.00210628 | 7.3739 | 7.4001 | 7.3903 | 7.3614 | 7.2679 | 7.3704 | 7.3773 | 7.3429 | 7.4154 | 7.4125 | 7.3482 | 7.3538 | 7.3802 | 7.3819 | 7.3816 |
| pos_2784 | PE(18:0/18:1(9Z)-O(12,13)) | metab_1856 | HMDB0261148 | - | 773.5587 | 7.3997 | pos | 2M+3H2O+2H | C41H78NO9P | 0 | 77.7 | 7.47091815 | - | 0.0359802 | 5.4281 | 5.155 | 5.2184 | 5.4644 | 5.5756 | 5.662 | 5.704 | 5.2353 | 5.5478 | 5.8165 | 5.7062 | 5.5183 | 5.5271 | 5.5276 | 5.5 |
| pos_2790 | (11R,16S)-misoprostol | metab_1862 | HMDB0242321 | - | 415.3034 | 7.4157 | pos | M+CH3OH+H | C22H38O5 | 0 | 49.9 | -5.2302948 | - | 0.20206038 | 3.5772 | 3.8917 | 3.9374 | 3.3574 | 3.3509 | 3.7754 | 3.8301 | 3.9435 | 4.0471 | 4.0058 | 3.848 | 3.4961 | 3.6213 | 3.7695 | 3.628 |
| pos_2794 | Peonidin 3-rutinoside | metab_1865 | HMDB0303649;LMPK12010237 | - | 610.1857 | 7.4237 | pos | M+H | C28H33O15+ | 0 | 33.7 | -5.7285207 | - | 0.00963888 | 5.2076 | 4.9631 | 5.108 | 4.9437 | 4.9687 | 5.0323 | 4.9338 | 5.2 | 5.0184 | 5.16 | 5.2036 | 4.902 | 7.956 | 7.9623 | 7.9639 |
| pos_2799 | Pirimicarb | metab_1870 | HMDB0256598 | C11079 | 256.176 | 7.4316 | pos | M+NH4 | C11H18N4O2 | 93.2 | 0 | -3.2718326 | 23103-98-2 | 0.00793537 | 5.1184 | 5.138 | 5.1376 | 4.9499 | 4.8232 | 5.0326 | 5.0602 | 4.8511 | 5.1744 | 5.0959 | 4.9988 | 5.0697 | 4.9296 | 4.9299 | 4.9357 |
| pos_2822 | 3alpha,7alpha,12alpha-trihydroxy-5beta-cholestanate | metab_1896 | HMDB0062207 | C04722 | 942.7021 | 7.4714 | pos | 2M+ACN+H | C27H46O5 | 0 | 40.4 | -0.8348665 | 547-98-8 | 0.02695146 | 5.7281 | 5.4641 | 5.6569 | 5.8662 | 5.9819 | 5.6624 | 5.4338 | 5.9055 | 4.8902 | 5.7497 | 5.7258 | 5.4193 | 5.8963 | 5.9197 | 5.9072 |
| pos_2826 | Serratol | metab_1900 | HMDB0039827 | - | 323.2951 | 7.4873 | pos | M+CH3OH+H | C20H34O | 0 | 46.9 | 2.04809478 | 67814-27-1 | 0.02084783 | 5.8539 | 5.9438 | 5.9862 | 5.9409 | 5.8194 | 5.9098 | 5.9497 | 5.9284 | 5.9987 | 6.0081 | 5.9225 | 5.9233 | 5.7487 | 5.7486 | 5.7329 |
| pos_2835 | PGP(PGD2/a-17:0) | metab_1910 | HMDB0274500 | - | 954.5101 | 7.4954 | pos | M+ACN+H | C43H78O16P2 | 0 | 53.3 | -0.2080431 | - | 0.0579789 | 5.0739 | 5.2289 | 4.8666 | 5.4516 | 5.4285 | 5.0931 | 5.0626 | 5.5775 | 4.2596 | 5.0915 | 5.1761 | 5.1136 | 5.4053 | 5.4481 | 5.4052 |
| pos_2836 | LysoPC(P-18:1(9Z)/0:0) | metab_1911 | HMDB0010408 | C04230 | 506.3614 | 7.5033 | pos | M+H | C26H52NO6P | 0 | 55.9 | 1.81420864 | - | 0.02703367 | 5.3141 | 5.2236 | 5.2465 | 5.104 | 4.9616 | 5.1779 | 5.1426 | 5.195 | 5.2282 | 5.189 | 5.2738 | 5.2892 | 5.262 | 5.2814 | 5.2835 |
| pos_2863 | Morpholine-4-carboxamide | metab_1939 | HMDB0254895 | - | 113.0716 | 7.5751 | pos | M+H-H2O | C5H10N2O2 | 0 | 50.1 | 5.23243546 | - | 0.00291952 | 5.1974 | 5.2078 | 5.2153 | 5.197 | 5.1362 | 5.1968 | 5.1796 | 5.1729 | 5.2205 | 5.2352 | 5.1668 | 5.1661 | 5.2138 | 5.2147 | 5.2163 |
| pos_2890 | (3a,5b)-24-oxo-24-[(2-sulfoethyl)amino]cholan-3-yl-b-D-Glucopyranosiduronic acid | metab_1965 | HMDB0002429 | - | 682.3294 | 7.6624 | pos | M+Na | C32H53NO11S | 0 | 55.1 | 9.43192413 | 99794-82-8 | 0.01738796 | 5.8729 | 5.7778 | 5.6791 | 5.7122 | 5.622 | 5.6632 | 5.817 | 5.7184 | 5.6939 | 5.7431 | 5.7437 | 5.8035 | 5.829 | 5.8427 | 5.8305 |
| pos_2892 | PC(20:5(5Z,8Z,11Z,14Z,17Z)/20:2(11Z,14Z)) | metab_1966 | HMDB0008506;PW_C004554;LMGP01011946 | C00157 | 832.5854 | 7.6704 | pos | M+H | C48H82NO8P | 0 | 94.4 | 0.34835836 | - | 0.09361701 | 6.046 | 6.7747 | 7.054 | 6.5696 | 6.4806 | 6.814 | 6.8507 | 6.7389 | 6.852 | 6.8672 | 6.6919 | 6.8701 | 6.2522 | 6.1718 | 6.2054 |
| pos_2895 | Histidinal | metab_1969 | HMDB0012234 | C01929 | 157.1088 | 7.6943 | pos | M+NH4 | C6H9N3O | 0 | 37.5 | 3.30528543 | 23784-15-8 | 0.01389802 | 4.7591 | 4.7445 | 4.678 | 4.7459 | 4.6655 | 4.7202 | 4.6839 | 4.6986 | 4.7389 | 4.7377 | 4.7158 | 4.6326 | 4.7857 | 4.7953 | 4.797 |
| pos_2897 | PC(20:2(11Z,14Z)/14:1(9Z)) | metab_1971 | PW_C004377;HMDB0008329;LMGP01011838 | C00157 | 756.5558 | 7.6943 | pos | M+H | C42H78NO8P | 0 | 46.7 | 2.60610017 | - | 0.11983415 | 6.1672 | 5.6256 | 6.2849 | 6.1027 | 5.9632 | 6.4556 | 6.3398 | 6.169 | 6.2354 | 6.4063 | 6.0613 | 6.157 | 4.3265 | 4.2363 | 4.3331 |
| pos_2898 | PC(18:4(6Z,9Z,12Z,15Z)/20:2(11Z,14Z)) | metab_1972 | PW_C004291;LMGP01011721;HMDB0008243 | C00157 | 806.5707 | 7.6943 | pos | M+H | C46H80NO8P | 0 | 94 | 1.57285787 | - | 0.04152721 | 6.7856 | 6.784 | 6.7727 | 6.7815 | 6.772 | 6.9104 | 6.8645 | 6.8661 | 6.8787 | 6.8813 | 6.8313 | 6.891 | 6.8716 | 6.8717 | 6.84 |
| pos_2903 | PC(14:0/18:2(9Z,12Z)) | metab_1979 | PW_C003924;HMDB0007874;LMGP01010496 | C00157 | 730.5403 | 7.7102 | pos | M+H | C40H76NO8P | 0 | 35.2 | 3.03522505 | 92345-33-0 | 0.17955295 | 5.6946 | 5.0788 | 5.9381 | 5.6148 | 5.6235 | 6.1046 | 5.9485 | 5.5721 | 5.7846 | 6.1432 | 5.5337 | 5.7944 | 4.4891 | 4.4144 | 4.3318 |
| pos_2904 | Falcarindiol | metab_1980 | HMDB0244257;LMFA05000658;HMDB0033941 | C08449 | 538.3881 | 7.7182 | pos | 2M+NH4 | C17H24O2 | 0 | 43.8 | -1.9495618 | 55297-87-5 | 0.00241819 | 6.0604 | 6.0813 | 6.1675 | 6.1126 | 5.9815 | 6.1284 | 6.1031 | 6.0891 | 6.1219 | 6.0754 | 6.0752 | 6.1029 | 5.9091 | 5.9085 | 5.9106 |
| pos_2907 | 1-O-Hexadecyl-2-C-Methyl-3-Phosphatidylcholine | metab_1983 | - | - | 496.3773 | 7.7422 | pos | M+H | C25H54NO6P | 97.1 | 0 | 2.24946928 | - | 0.03785915 | 4.8506 | 4.8563 | 4.6148 | 4.5687 | 4.7812 | 4.8797 | 4.8423 | 4.8125 | 4.8591 | 4.7941 | 4.898 | 4.8706 | 4.4837 | 4.5127 | 4.4854 |
| pos_2908 | Risbitin | metab_1984 | HMDB0302980 | C09715 | 508.342 | 7.7502 | pos | 2M+ACN+Na | C14H22O2 | 0 | 30.4 | 5.16418794 | - | 0.01328555 | 4.4117 | 5.199 | 4.9249 | 4.8759 | 4.7203 | 4.5096 | 4.9321 | 4.7316 | 4.935 | 4.5783 | 4.3392 | 4.3385 | 4.8818 | 4.8807 | 4.8712 |
| pos_291 | 1-(Hydroxymethyl)-5,5-dimethyl-2,4-imidazolidinedione | metab_1986 | HMDB0031670 | - | 176.1034 | 1.0247 | pos | M+H, M+NH4 | C6H10N2O3 | 0 | 32.4 | 2.9359911 | 116-25-6 | 0.0051439 | 6.3478 | 6.4137 | 6.2625 | 6.4087 | 6.067 | 5.996 | 6.4622 | 6.287 | 6.0959 | 6.3866 | 5.9476 | 6.2022 | 6.0903 | 6.0901 | 6.094 |
| pos_2920 | Arkofix | metab_1998 | HMDB0248602 | - | 211.0934 | 7.7742 | pos | M+CH3OH+H | C5H10N2O5 | 0 | 33.7 | 5.09776064 | - | 0.01295283 | 4.7769 | 4.8023 | 4.7742 | 4.8161 | 4.744 | 4.8329 | 4.7946 | 4.8012 | 4.8198 | 4.8073 | 4.7707 | 4.8019 | 4.8061 | 4.8165 | 4.8078 |
| pos_2965 | (2E)-4-Hydroxy-3-methylbut-2-en-1-yl trihydrogen diphosphate | metab_2046 | HMDB0242600;LMPR01010009 | C11811 | 244.9987 | 7.9989 | pos | M+H-H2O | C5H12O8P2 | 0 | 32.1 | 4.88601023 | - | 0.0140481 | 5.131 | 5.1658 | 5.1167 | 5.1792 | 5.1327 | 5.1752 | 5.1564 | 5.15 | 5.17 | 5.1669 | 5.16 | 5.1479 | 5.1871 | 5.1868 | 5.1975 |
| pos_305 | Hypoxanthine | metab_2138 | HMDB0000157;PW_C000102 | C00262 | 137.0463 | 2.6791 | pos | M+H-H2O, M+H | C5H4N4O | 51.4 | 0 | 3.97270462 | 68-94-0 | 0.00514559 | 7.0634 | 6.9819 | 7.0208 | 6.6694 | 6.8831 | 6.8892 | 6.9811 | 6.9304 | 6.9639 | 6.9675 | 6.9089 | 6.9154 | 6.7443 | 6.744 | 6.7403 |
| pos_306 | 2-Amino-9-[(2R,4S,5R)-5-(hydroperoxymethyl)-4-hydroxyoxolan-2-yl]-1H-purin-6-one | metab_2148 | HMDB0257980 | - | 284.0997 | 2.6791 | pos | M+H, M+Na | C10H13N5O5 | 0 | 45 | 2.72471601 | - | 0.00400277 | 6.1303 | 6.031 | 6.0024 | 5.8728 | 5.867 | 5.7518 | 6.0257 | 5.9189 | 6.0266 | 5.9585 | 6.0095 | 5.9358 | 5.7064 | 5.7083 | 5.7099 |
| pos_3061 | 24-Oxo-1alpha,25-dihydroxyvitamin D3 | metab_2150 | HMDB0060128 | - | 494.3253 | 7.7582 | pos | M+ACN+Na | C27H42O4 | 0 | 41.3 | 2.94929087 | - | 0.00829347 | 5.1184 | 5.4776 | 5.2869 | 5.3323 | 5.2563 | 5.2304 | 5.3017 | 5.3392 | 5.3474 | 5.2512 | 5.1874 | 5.1684 | 5.2454 | 5.2401 | 5.247 |
| pos_3063 | 5-Methyl-1H-Benzotriazole | metab_2152 | - | - | 134.0717 | 7.7582 | pos | M+H | C7H7N3 | 79.5 | 0 | 3.54280935 | - | 0.00704069 | 4.6383 | 4.6757 | 4.6722 | 4.671 | 4.621 | 4.664 | 4.6763 | 4.6486 | 4.6913 | 4.6971 | 4.6375 | 4.6097 | 4.6938 | 4.6981 | 4.6998 |
| pos_3064 | Salbutamol | metab_2153 | HMDB0001937 | C11770 | 520.3416 | 7.7502 | pos | 2M+ACN+H | C13H21NO3 | 0 | 53.6 | 7.1886495 | 18559-94-9 | 0.00189802 | 6.2779 | 6.4325 | 6.3745 | 6.2559 | 6.3824 | 6.3848 | 6.3899 | 6.222 | 6.4356 | 6.2234 | 6.3159 | 6.4174 | 6.3671 | 6.3671 | 6.3686 |
| pos_3065 | 7-Hydroxyoctadecanoylcarnitine | metab_2154 | HMDB0241523 | - | 482.3256 | 7.7502 | pos | M+K | C25H49NO5 | 0 | 59.6 | 3.17000724 | - | 0.01060329 | 5.1415 | 5.4847 | 5.2821 | 5.1397 | 5.3641 | 5.3384 | 5.2872 | 5.0563 | 5.5346 | 5.2548 | 5.332 | 5.1654 | 5.3158 | 5.3156 | 5.3237 |
| pos_3072 | PC(18:2/0:0) | metab_2162 | LMGP01050035;LMGP01050034 | - | 542.323 | 7.7182 | pos | M+Na | C26H50NO7P | 82.2 | 0 | 2.50274152 | - | 0.00085989 | 5.7166 | 5.8753 | 5.8067 | 5.6525 | 5.7988 | 5.822 | 5.8649 | 5.6636 | 5.9032 | 5.6411 | 5.7757 | 5.8577 | 5.8003 | 5.8001 | 5.7996 |
| pos_3073 | (3beta,5alpha,6beta,22E,24R)-23-Methylergosta-7,22-diene-3,5,6-triol | metab_2163 | HMDB0033633 | - | 508.3774 | 7.7182 | pos | M+ACN+Na | C29H48O3 | 0 | 40 | 2.99192235 | 243449-54-9 | 0.01133957 | 6.0842 | 6.0703 | 6.1116 | 5.9848 | 6.133 | 6.1295 | 6.1745 | 6.065 | 6.132 | 6.0683 | 6.1297 | 6.1995 | 6.0959 | 6.1034 | 6.1052 |
| pos_3074 | DL-Allylglycine | metab_2164 | HMDB0251510 | - | 98.0608 | 7.7182 | pos | M+H-H2O | C5H9NO2 | 0 | 36.8 | 7.02194356 | - | 0.01683864 | 4.1535 | 4.0893 | 4.0509 | 4.125 | 4.051 | 4.1388 | 4.076 | 4.2238 | 4.1646 | 4.1665 | 4.0555 | 4.083 | 4.1508 | 4.164 | 4.1522 |
| pos_3075 | PC(18:0/18:4(6Z,9Z,12Z,15Z)) | metab_2165 | HMDB0008042;LMGP01010773;PW_C004091 | C00157 | 782.5695 | 7.7102 | pos | M+H | C44H80NO8P | 0 | 64.4 | 0.14718796 | - | 0.06584653 | 6.5001 | 6.2573 | 6.5482 | 6.5546 | 6.3185 | 6.7563 | 6.729 | 6.509 | 6.4934 | 6.7318 | 6.5069 | 6.5889 | 6.2543 | 6.2045 | 6.2558 |
| pos_3078 | PC(20:1(11Z)/18:3(9Z,12Z,15Z)) | metab_2168 | PW_C004353;LMGP01011820;HMDB0008305 | C00157 | 810.6026 | 7.7023 | pos | M+H | C46H84NO8P | 0 | 82.6 | 2.27831162 | - | 0.04721898 | 6.5049 | 7.2065 | 7.4528 | 6.9929 | 6.9276 | 7.1721 | 7.2416 | 7.135 | 7.2657 | 7.2487 | 7.101 | 7.2681 | 6.7412 | 6.7427 | 6.7059 |
| pos_3079 | LysoPC(0:0/18:0) | metab_2169 | HMDB0011128 | - | 562.3283 | 7.7023 | pos | M+K | C26H54NO7P | 0 | 74.9 | 2.61474399 | 4421-58-3 | 0.00191034 | 6.3401 | 6.2926 | 6.2804 | 6.2812 | 6.1488 | 6.1807 | 6.3522 | 6.2775 | 6.2082 | 6.2717 | 6.2821 | 6.3399 | 6.329 | 6.3284 | 6.3301 |
| pos_308 | Deoxyinosine | metab_2170 | PW_C000050;HMDB0000071;MJDBOTE0001081 | C05512 | 253.0939 | 2.7591 | pos | M+H, M+Na | C10H12N4O4 | 77.9 | 0 | 2.85376587 | 890-38-0 | 0.00318338 | 4.7403 | 4.9465 | 4.6503 | 3.9355 | 4.8814 | 4.814 | 4.6202 | 4.5711 | 4.9245 | 4.6497 | 4.9 | 4.7768 | 4.4703 | 4.4714 | 4.4731 |
| pos_3080 | LysoPE(22:0/0:0) | metab_2171 | HMDB0011520 | - | 560.3701 | 7.7023 | pos | M+Na | C27H56NO7P | 0 | 63.4 | 2.77017157 | - | 0.01448875 | 5.5018 | 5.4918 | 5.5886 | 5.543 | 5.3969 | 5.5367 | 5.5855 | 5.5109 | 5.5572 | 5.4875 | 5.4661 | 5.5642 | 5.2159 | 5.2156 | 5.2266 |
| pos_3081 | LysoPC(P-18:0/0:0) | metab_2172 | HMDB0013122 | C04230 | 530.3594 | 7.7023 | pos | M+Na | C26H54NO6P | 0 | 74.7 | 2.6629935 | - | 0.01267601 | 5.6245 | 5.5252 | 5.628 | 5.5542 | 5.639 | 5.6475 | 5.7277 | 5.6311 | 5.6276 | 5.6011 | 5.6226 | 5.7464 | 5.6024 | 5.611 | 5.6126 |
| pos_309 | NIPECOTIC ACID | metab_2181 | HMDB0255618 | - | 130.0868 | 2.7431 | pos | M+H-H2O, M+H | C6H11NO2 | 65.5 | 0 | 4.60932169 | - | 0.00095957 | 5.0118 | 5.2459 | 5.0865 | 5.0942 | 5.1507 | 5.2289 | 5.0867 | 5.0188 | 5.1683 | 5.0436 | 5.2167 | 5.1971 | 5.2543 | 5.2541 | 5.2549 |
| pos_3096 | Cyanidin 3-glucosyl-rutinoside | metab_2188 | HMDB0302725 | - | 758.2237 | 7.6624 | pos | M+H | C33H41O20+ | 0 | 38.7 | -3.5893039 | - | 0.12260217 | 7.2854 | 5.4153 | 5.1311 | 5.2551 | 5.6094 | 5.7943 | 5.6935 | 5.2693 | 5.705 | 5.2832 | 5.513 | 5.5064 | 4.316 | 4.2248 | 4.2284 |
| pos_31 | Bz-Arg-OEt | metab_2192 | HMDB0249494 | C01670 | 289.1651 | 5.4189 | pos | M+H-H2O, M+NH4, M+Na, M+H | C15H22N4O3 | 0 | 36.1 | -2.4907653 | - | 0.01292202 | 4.2985 | 3.589 | 3.9632 | 4.2312 | 3.7848 | 3.9649 | 5.0703 | 3.8976 | 4.1989 | 4.2613 | 4.3625 | 4.0574 | 5.0613 | 5.0508 | 5.0525 |
| pos_3127 | Fagomine | metab_2219 | HMDB0033453 | C10144 | 148.0972 | 7.6068 | pos | M+H | C6H13NO3 | 0 | 38 | 2.84452931 | 53185-12-9 | 0.00745961 | 5.4578 | 5.4535 | 5.44 | 5.4498 | 5.3966 | 5.445 | 5.4477 | 5.4316 | 5.4684 | 5.4845 | 5.4108 | 5.4182 | 5.4791 | 5.4837 | 5.4853 |
| pos_3135 | Furanogermenone | metab_2227 | HMDB0036766 | C17488 | 487.2807 | 7.583 | pos | 2M+Na | C15H20O2 | 0 | 31.8 | -2.5116041 | 81678-18-4 | 0.0024414 | 6.3816 | 6.2793 | 6.2969 | 6.2886 | 6.1077 | 6.1833 | 6.3268 | 6.2381 | 6.2057 | 6.2225 | 6.2675 | 6.3067 | 6.3083 | 6.3063 | 6.3079 |
| pos_3136 | Hexylamine | metab_2228 | HMDB0032323 | C08306 | 102.1285 | 7.583 | pos | M+H | C6H15N | 0 | 51.6 | 7.67332054 | 111-26-2 | 0.00239333 | 7.7894 | 7.8221 | 7.8005 | 7.7727 | 7.6712 | 7.7782 | 7.7874 | 7.7471 | 7.8348 | 7.8193 | 7.7532 | 7.7605 | 7.7991 | 7.8012 | 7.8005 |
| pos_3144 | Butylisopropylamine | metab_2235 | - | - | 116.144 | 7.5591 | pos | M+H | C7H17N | 85.9 | 0 | 5.59842267 | - | 0.00725901 | 7.2863 | 7.3129 | 7.2998 | 7.2687 | 7.1754 | 7.2772 | 7.2829 | 7.2497 | 7.3237 | 7.3192 | 7.2524 | 7.261 | 7.2974 | 7.2971 | 7.2918 |
| pos_3147 | (+/-)-Pelletierine | metab_2238 | HMDB0030325 | C06182 | 142.1231 | 7.5512 | pos | M+H | C8H15NO | 0 | 44.5 | 3.13157633 | 539-00-4 | 0.00453514 | 5.4901 | 5.5337 | 5.5145 | 5.4767 | 5.3753 | 5.4815 | 5.5203 | 5.4236 | 5.5375 | 5.5536 | 5.4567 | 5.4925 | 5.4822 | 5.4845 | 5.4862 |
| pos_316 | 1-Acetamidocyclopentanecarboxylic acid | metab_2252 | - | - | 136.0762 | 2.8151 | pos | M+H-2H2O, M+H | C8H13NO3 | 47 | 0 | 2.91357267 | - | 0.00338386 | 5.262 | 5.2235 | 5.063 | 4.9998 | 5.123 | 4.9689 | 5.1937 | 5.1222 | 5.0771 | 5.0436 | 5.0118 | 5.2477 | 5.1963 | 5.1992 | 5.1977 |
| pos_3181 | DG(20:1(11Z)/16:1(9Z)/0:0) | metab_2275 | PW_C003440;HMDB0007389 | C00165 | 643.5282 | 7.4954 | pos | M+Na | C39H72O5 | 0 | 84.5 | 1.57307948 | - | 0.04739735 | 6.277 | 6.3002 | 6.2832 | 6.3185 | 6.2789 | 6.3389 | 6.264 | 6.1983 | 6.3423 | 6.3469 | 6.241 | 6.2282 | 5.7242 | 5.7252 | 5.6885 |
| pos_3185 | Dodecyl benzenesulfonate | metab_2278 | HMDB0251568 | - | 344.2286 | 7.4873 | pos | M+NH4 | C18H30O3S | 0 | 50.4 | 9.74346237 | - | 0.02193127 | 5.2339 | 5.2161 | 5.2697 | 5.1598 | 5.1519 | 5.2182 | 5.1782 | 5.1965 | 5.2727 | 5.2387 | 5.1878 | 5.2149 | 5.1447 | 5.154 | 5.1638 |
| pos_3187 | PGP(PGF2alpha/i-16:0) | metab_2280 | HMDB0275231 | - | 942.5017 | 7.4793 | pos | M+ACN+H | C42H78O16P2 | 0 | 31.6 | -9.5631221 | - | 0.01153664 | 5.7369 | 5.4375 | 5.758 | 6.0328 | 6.1261 | 5.9081 | 5.7157 | 6.0906 | 5.2483 | 5.8036 | 5.8694 | 5.816 | 6.1065 | 6.1158 | 6.1079 |
| pos_3189 | DG(18:0/18:2(9Z,12Z)/0:0) | metab_2282 | HMDB0007161;PW_C003212 | C00165 | 638.5732 | 7.4793 | pos | M+NH4 | C39H72O5 | 0 | 60.8 | 2.25827622 | 34487-26-8 | 0.06668062 | 6.1607 | 6.2399 | 6.2082 | 6.1201 | 6.0012 | 6.1185 | 6.2067 | 6.1078 | 6.2361 | 6.2209 | 6.1011 | 6.1284 | 4.5037 | 4.5042 | 4.4527 |
| pos_319 | Porphobilinogen | metab_2283 | PW_C000166;HMDB0000245 | C00931 | 227.1032 | 2.8229 | pos | M+H-2H2O, M+H | C10H14N2O4 | 0 | 64.2 | 2.44507895 | 487-90-1 | 0.02326844 | 5.2204 | 5.3157 | 5.2135 | 5.2896 | 5.2258 | 5.2989 | 5.3011 | 5.2259 | 5.3387 | 5.1941 | 5.2669 | 5.3769 | 5.1414 | 5.1582 | 5.1597 |
| pos_32 | Acetylshikonin | metab_2293 | HMDB0247936 | C17413 | 348.1449 | 5.6594 | pos | M+H-H2O, M+NH4, M+Na, M+H-2H2O | C18H18O6 | 0 | 41.5 | 2.22695626 | 24502-78-1 | 0.01397599 | 5.6093 | 5.727 | 5.7491 | 5.6313 | 5.5636 | 5.4231 | 5.9071 | 5.9178 | 5.545 | 5.4331 | 5.6989 | 5.7306 | 5.5693 | 5.568 | 5.579 |
| pos_3206 | 3-Oxododecanoic acid | metab_2301 | PW_C006734;HMDB0010727 | C02367 | 237.1464 | 7.4395 | pos | M+Na | C12H22O3 | 0 | 42.8 | 1.50390128 | - | 0.05518394 | 4.5264 | 4.5337 | 4.6303 | 4.5396 | 4.2348 | 4.3583 | 4.4471 | 4.4164 | 4.612 | 4.5214 | 4.4241 | 4.4707 | 4.2997 | 4.3 | 4.2577 |
| pos_3210 | Methacycline | metab_2306 | PW_C009375;HMDB0015066 | C07654 | 907.2713 | 7.4316 | pos | 2M+Na | C22H22N2O8 | 0 | 44.6 | 7.71767478 | 914-00-1 | 0.13647154 | 5.8512 | 5.7365 | 5.8276 | 6.4593 | 6.0209 | 6.1838 | 5.7787 | 6.3488 | 5.4781 | 6.2172 | 5.9491 | 5.9895 | 6.4198 | 6.3154 | 6.3263 |
| pos_322 | Ethyl 2-methyl-3,4-pentadienoate | metab_2316 | HMDB0037196;LMFA07010858 | - | 105.0707 | 2.8309 | pos | M+H-2H2O, 2M+K | C8H12O2 | 0 | 37.7 | 5.6458673 | 60523-21-9 | 0.01587231 | 5.0158 | 5.0197 | 5.0223 | 5.0228 | 5.0076 | 5.0392 | 5.032 | 5.026 | 5.045 | 5.0756 | 5.02 | 5.0252 | 5.163 | 5.1754 | 5.1642 |
| pos_3224 | Prostavasin | metab_2319 | HMDB0001442;HMDB0256860 | C04741 | 355.2459 | 7.4157 | pos | M+H | C20H34O5 | 0 | 30.7 | -5.7309017 | 745-65-3 | 0.12431535 | 4.3306 | 4.4705 | 4.3294 | 3.8961 | 3.8916 | 4.0105 | 4.107 | 4.1358 | 4.5624 | 4.3844 | 4.1978 | 4.3222 | 3.8721 | 3.7827 | 3.7804 |
| pos_3234 | (-)-1-Cyclohexyl-4-tricosanol | metab_2329 | HMDB0040884 | - | 883.8573 | 7.3917 | pos | 2M+K | C29H58O | 0 | 39.1 | -3.9648845 | 151454-21-6 | 0.02151419 | 5.832 | 5.6203 | 5.7476 | 5.9498 | 5.8994 | 5.8458 | 5.7189 | 5.8058 | 5.3286 | 5.9247 | 5.768 | 5.8406 | 5.9412 | 5.9254 | 5.9422 |
| pos_3238 | Cetylmannoside | metab_2333 | HMDB0249840 | - | 872.6504 | 7.3837 | pos | 2M+ACN+Na | C22H44O6 | 0 | 43.6 | 8.72466601 | - | 0.02123829 | 6.4306 | 6.2395 | 6.382 | 6.5583 | 6.5868 | 6.4702 | 6.3527 | 6.6244 | 6.228 | 6.4785 | 6.4939 | 6.4501 | 6.5835 | 6.5829 | 6.5991 |
| pos_3242 | (1(10)E,4a,5E)-1(10),5-Germacradiene-12-acetoxy-4,11-diol | metab_2338 | HMDB0038798 | - | 297.204 | 7.3837 | pos | M+H | C17H28O4 | 0 | 69.6 | -6.8443105 | 143305-08-2 | 0.0347692 | 5.0931 | 5.2894 | 5.2036 | 5.0854 | 4.8689 | 5.1378 | 5.1475 | 4.9703 | 5.2538 | 5.176 | 5.0309 | 5.1293 | 5.0103 | 5.0357 | 5.0376 |
| pos_3249 | Toluene | metab_2345 | HMDB0034168 | C01455 | 93.0707 | 7.3757 | pos | M+H | C7H8 | 0 | 58.7 | 9.16719618 | 108-88-3 | 0.00329467 | 4.7521 | 4.7929 | 4.7671 | 4.7087 | 4.5994 | 4.7454 | 4.7299 | 4.6807 | 4.8153 | 4.8089 | 4.7165 | 4.7545 | 4.6682 | 4.671 | 4.6696 |
| pos_3250 | 2,5-Heptadien-1-ol | metab_2347 | HMDB0040339 | - | 95.0864 | 7.3757 | pos | M+H-H2O | C7H12O | 0 | 41.6 | 7.36254505 | 62237-90-5 | 0.01049738 | 5.4734 | 5.5144 | 5.4895 | 5.404 | 5.3289 | 5.4579 | 5.4846 | 5.4168 | 5.5252 | 5.5227 | 5.4098 | 5.4439 | 5.4118 | 5.4203 | 5.4132 |
| pos_3254 | 4-Deoxypyridoxine | metab_2351 | HMDB0246408 | - | 118.0657 | 7.3677 | pos | M+H-2H2O | C8H11NO2 | 0 | 50.2 | 3.87190852 | - | 0.00303744 | 5.6667 | 5.717 | 5.6948 | 5.6215 | 5.539 | 5.661 | 5.6807 | 5.6087 | 5.7392 | 5.709 | 5.613 | 5.6363 | 5.6192 | 5.6205 | 5.6178 |
| pos_326 | Carmofur | metab_2357 | HMDB0249685 | C13252 | 240.1139 | 2.9187 | pos | M+H-H2O, M+H | C11H16FN3O3 | 0 | 52.1 | -1.6387071 | 61422-45-5 | 0.0148583 | 4.8187 | 5.6299 | 5.1614 | 5.3725 | 5.4335 | 5.6068 | 5.3623 | 3.631 | 3.8409 | 4.268 | 5.5536 | 5.4313 | 4.9967 | 4.9966 | 5.0078 |
| pos_327 | Bemegride | metab_2366 | HMDB0248936 | - | 173.129 | 2.9348 | pos | M+NH4, M+ACN+H | C8H13NO2 | 0 | 44 | 3.81150608 | - | 0.01441265 | 4.529 | 4.6472 | 4.3552 | 4.5925 | 4.4531 | 4.6549 | 4.9404 | 4.3021 | 4.4736 | 4.4036 | 4.5645 | 4.6589 | 4.5727 | 4.5841 | 4.5741 |
| pos_3283 | Cer(d18:1/20:3(8Z,11Z,14Z)-2OH(5,6)) | metab_2380 | HMDB0290014 | - | 652.5523 | 7.3202 | pos | M+CH3OH+H | C38H69NO5 | 0 | 49.5 | 2.0414989 | - | 0.07961663 | 4.6474 | 4.8936 | 4.8196 | 4.666 | 3.6829 | 4.4835 | 4.612 | 4.505 | 4.8348 | 4.7703 | 4.3655 | 4.7209 | 4.4638 | 4.5237 | 4.5266 |
| pos_3290 | PC(14:0/P-18:1(9Z)) | metab_2388 | HMDB0007898 | C00157 | 716.5602 | 7.3121 | pos | M+H | C40H78NO7P | 0 | 51.7 | 1.90229202 | - | 0.05577129 | 3.3548 | 4.4671 | 3.3881 | 3.3803 | 3.5838 | 3.6032 | 3.4009 | 3.5853 | 3.4333 | 3.808 | 3.4312 | 3.5657 | 3.775 | 3.7336 | 3.7339 |
| pos_33 | Pentaethylene glycol | metab_2398 | HMDB0256256 | - | 239.1496 | 3.0548 | pos | M+H, M+K, M+Na, M+NH4 | C10H22O6 | 87.5 | 0 | 2.77617161 | - | 0.00224404 | 5.702 | 5.7334 | 5.7539 | 5.7196 | 5.815 | 5.7184 | 5.7512 | 5.7161 | 5.7117 | 5.7006 | 5.7578 | 5.7791 | 6.1504 | 6.1502 | 6.1486 |
| pos_3313 | Nigakihemiacetal B | metab_2414 | HMDB0035922 | C08771 | 803.4004 | 7.2791 | pos | 2M+Na | C22H30O6 | 0 | 51.2 | 3.49799797 | 76-77-7 | 0.01264646 | 6.2941 | 6.2379 | 6.2996 | 6.4089 | 6.4859 | 6.3905 | 6.2653 | 6.4162 | 6.2303 | 6.354 | 6.455 | 6.3832 | 6.4534 | 6.4446 | 6.4548 |
| pos_3322 | SM(d19:0/PGF2alpha) | metab_2424 | HMDB0290623 | - | 799.5944 | 7.2711 | pos | M+H-H2O | C44H85N2O9P | 0 | 32.1 | -1.9509462 | - | 0.03502082 | 5.5185 | 5.4113 | 5.5116 | 5.5647 | 5.6916 | 5.6122 | 5.4181 | 5.6487 | 5.5194 | 5.5467 | 5.5749 | 5.558 | 5.6818 | 5.656 | 5.6834 |
| pos_3324 | (13Z,16Z,19Z)-Docosa-13,16,19-trienoylcarnitine | metab_2426 | HMDB0241605 | - | 516.3436 | 7.2711 | pos | M+K | C29H51NO4 | 0 | 56.1 | -2.8588392 | - | 0.0435072 | 4.9524 | 4.8767 | 4.7956 | 4.7482 | 4.4638 | 4.3915 | 4.9069 | 4.4356 | 4.297 | 4.7904 | 4.293 | 4.4684 | 4.7759 | 4.75 | 4.7871 |
| pos_3331 | Y-27632 Dihydrochloride | metab_2434 | HMDB0259944 | - | 558.3543 | 7.2628 | pos | 2M+ACN+Na | C14H21N3O | 0 | 49.5 | 3.25558796 | - | 0.04500684 | 4.9488 | 5.3614 | 5.3163 | 5.251 | 4.7995 | 4.8508 | 5.2535 | 4.9948 | 5.0757 | 5.243 | 4.5902 | 4.7915 | 5.0163 | 5.0499 | 5.0514 |
| pos_3333 | Erucamide | metab_2436 | LMFA08010028;HMDB0244507 | - | 338.3423 | 7.2628 | pos | M+H | C22H43NO | 55 | 0 | 1.72333471 | - | 0.02456677 | 5.5826 | 5.5865 | 5.5814 | 5.4468 | 5.4769 | 5.5321 | 5.5839 | 5.4621 | 5.5074 | 5.6269 | 5.4683 | 5.5745 | 5.62 | 5.6378 | 5.6393 |
| pos_3337 | DG(20:2(11Z,14Z)/16:1(9Z)/0:0) | metab_2440 | PW_C003469;HMDB0007418 | C00165 | 641.5129 | 7.2549 | pos | M+Na | C39H70O5 | 0 | 31.3 | 2.13017228 | - | 0.02742869 | 5.3346 | 5.2314 | 5.1603 | 5.034 | 4.4932 | 4.8533 | 5.1103 | 4.5109 | 4.9546 | 5.1948 | 4.5511 | 4.8923 | 5.3888 | 5.4084 | 5.4106 |
| pos_3344 | PC(20:5(5Z,8Z,11Z,14Z,17Z)/20:1(11Z)) | metab_2448 | HMDB0008505;PW_C004553;LMGP01011945 | C00157 | 834.6018 | 7.2468 | pos | M+H | C48H84NO8P | 0 | 80.4 | 1.30686646 | - | 0.15996732 | 5.9287 | 6.2396 | 6.46 | 6.0829 | 6.1119 | 6.229 | 6.2982 | 6.1674 | 6.3603 | 6.3493 | 6.2422 | 6.2561 | 6.1282 | 5.9921 | 6.1055 |
| pos_3363 | PE(22:2(13Z,16Z)/18:1(12Z)-O(9S,10R)) | metab_2469 | HMDB0283978 | - | 825.5827 | 7.2177 | pos | 2M+3H2O+2H | C45H82NO9P | 0 | 46.9 | -1.8982065 | - | 0.01977413 | 4.694 | 4.6992 | 4.9227 | 4.8305 | 5.1657 | 4.908 | 4.962 | 4.4502 | 4.9953 | 5.0947 | 4.9398 | 4.8204 | 4.984 | 4.9799 | 4.9674 |
| pos_338 | D-Dethiobiotin | metab_2486 | HMDB0003581 | C01909 | 215.1397 | 3.0868 | pos | M+H, M+Na | C10H18N2O3 | 45.6 | 0 | 3.18302048 | 533-48-2 | 0.0168246 | 4.0616 | 4.8135 | 4.5246 | 4.8083 | 4.3947 | 4.5779 | 4.3671 | 4.1593 | 4.2051 | 3.9347 | 4.6854 | 4.6429 | 4.3576 | 4.3457 | 4.3592 |
| pos_3380 | PE(22:1(13Z)/20:3(8Z,11Z,14Z)-2OH(5,6)) | metab_2487 | HMDB0283860 | - | 869.6091 | 7.19 | pos | 2M+3H2O+2H | C47H86NO10P | 0 | 34.4 | -1.6497414 | - | 0.01722957 | 5.2723 | 5.3903 | 5.3923 | 5.3276 | 5.5637 | 5.4491 | 5.1576 | 5.2529 | 5.544 | 5.5026 | 5.3605 | 5.326 | 5.4079 | 5.3957 | 5.4094 |
| pos_339 | 1-Methyl-3-(2-oxopropylidene)indol-2-one | metab_2497 | HMDB0258628 | - | 202.0869 | 3.0868 | pos | M+H-H2O, M+H | C12H11NO2 | 0 | 49.4 | 3.15836156 | - | 0.0156319 | 4.7787 | 4.2837 | 4.0125 | 4.138 | 4.5108 | 4.2839 | 4.1237 | 4.0154 | 4.5501 | 4.122 | 4.7662 | 4.5674 | 5.7651 | 5.7526 | 5.7543 |
| pos_3398 | PS(22:0/18:1(12Z)-2OH(9,10)) | metab_2505 | HMDB0282930 | - | 891.6224 | 7.1685 | pos | 2M+3H2O+2H | C46H88NO12P | 0 | 45 | 7.30292935 | - | 0.02651783 | 5.5973 | 5.6402 | 5.6994 | 5.6526 | 5.74 | 5.706 | 5.608 | 5.6283 | 5.7214 | 5.7301 | 5.7623 | 5.6586 | 5.6782 | 5.678 | 5.6979 |
| pos_3403 | PE(24:1(15Z)/PGF2alpha) | metab_2512 | HMDB0284576 | - | 913.6357 | 7.1605 | pos | 2M+3H2O+2H | C49H90NO11P | 0 | 43 | -1.1299615 | - | 0.0166081 | 5.6805 | 5.6129 | 5.7366 | 5.6824 | 5.8235 | 5.7937 | 5.7027 | 5.7088 | 5.8621 | 5.8868 | 5.8818 | 5.7354 | 5.7838 | 5.7705 | 5.7725 |
| pos_3408 | PE(20:1(11Z)/14:0) | metab_2517 | PW_C005298;HMDB0009250;LMGP02010843 | C00350 | 718.5366 | 7.1605 | pos | M+H | C39H76NO8P | 0 | 60 | -2.0879483 | - | 0.15375809 | 4.5302 | 4.3569 | 2.896 | 5.0033 | 4.756 | 4.3262 | 3.982 | 4.9913 | 4.1148 | 4.3949 | 4.55 | 4.4712 | 4.7898 | 4.9083 | 4.9147 |
| pos_3460 | DG(8:0/a-13:0/0:0) | metab_2572 | PW_C064412;HMDB0092911 | C00165 | 459.3031 | 7.1049 | pos | M+2Na-H | C24H46O5 | 0 | 35.8 | -6.131534 | - | 0.00341681 | 5.4226 | 5.4361 | 5.3998 | 5.3164 | 5.1114 | 5.254 | 5.3505 | 5.297 | 5.3585 | 5.3565 | 5.1718 | 5.2255 | 5.3579 | 5.3575 | 5.3551 |
| pos_347 | 3-Amino-4-hydroxybenzoic acid | metab_2582 | HMDB0304941 | C12115 | 154.0504 | 3.2344 | pos | M+H-H2O, M+H | C7H7NO3 | 0 | 54.9 | 3.44248937 | 1571-72-8 | 0.00685911 | 3.7167 | 3.6217 | 3.614 | 3.7424 | 3.5739 | 3.4811 | 3.8685 | 3.6419 | 3.6763 | 3.7042 | 3.6287 | 3.7187 | 4.0566 | 4.0564 | 4.0513 |
| pos_3483 | DG(8:0/14:0/0:0) | metab_2594 | HMDB0092913;PW_C062180 | C00165 | 473.319 | 7.081 | pos | M+2Na-H | C25H48O5 | 0 | 41.7 | -5.5338378 | - | 0.00575024 | 4.8588 | 4.9525 | 4.8046 | 4.9266 | 4.8594 | 5.103 | 4.9894 | 5.0035 | 4.9491 | 5.0541 | 5.095 | 4.9019 | 5.363 | 5.3627 | 5.3585 |
| pos_3487 | SM(d17:2(4E,8Z)/16:0) | metab_2598 | HMDB0240677 | - | 750.5562 | 7.073 | pos | M+ACN+Na | C38H75N2O6P | 0 | 68.6 | 6.07589954 | - | 0.14209788 | 6.4421 | 6.5915 | 6.5251 | 6.5552 | 6.4917 | 6.689 | 6.5308 | 6.5772 | 6.481 | 6.5512 | 6.7124 | 6.8183 | 6.4811 | 6.5038 | 6.3829 |
| pos_349 | S-4-Hydroxymephenytoin | metab_2601 | HMDB0013894 | - | 235.1084 | 3.2505 | pos | M+H-H2O, M+H | C12H14N2O3 | 0 | 43.5 | 2.92024461 | - | 0.02558017 | 4.3344 | 4.3061 | 4.4646 | 4.2843 | 4.319 | 4.7647 | 4.187 | 4.3692 | 4.2143 | 4.1384 | 4.7056 | 4.4681 | 4.3254 | 4.3068 | 4.3269 |
| pos_3499 | PC(P-16:0/0:0) | metab_2609 | HMDB0010407;LMGP01070006 | - | 502.328 | 7.0571 | pos | M+Na | C24H50NO6P | 95.3 | 0 | 2.47692061 | - | 0.01865839 | 6.4369 | 6.0332 | 5.9869 | 6.2464 | 5.6745 | 5.6842 | 6.1008 | 6.0119 | 5.6282 | 6.0941 | 5.6758 | 5.8504 | 6.3807 | 6.394 | 6.3955 |
| pos_35 | LysoPC(0:0/18:2(9Z,12Z)) | metab_2610 | HMDB0061700 | - | 520.3413 | 6.6739 | pos | M+H, M+K, M+Na, M+H-H2O | C26H50NO7P | 0 | 79.5 | 2.89135269 | 27304-12-7 | 0.00217132 | 8.1941 | 8.3028 | 8.232 | 8.2135 | 8.1592 | 8.2664 | 8.2596 | 8.1283 | 8.2954 | 8.1043 | 8.1865 | 8.2638 | 8.2791 | 8.2809 | 8.2806 |
| pos_350 | Olprinone | metab_2611 | HMDB0255959 | - | 289.0502 | 3.2824 | pos | M+Na, M+K | C14H10N4O | 0 | 35.1 | 6.37134102 | - | 0.0045893 | 5.2094 | 5.414 | 5.3401 | 5.3387 | 5.212 | 5.2362 | 5.3913 | 5.2635 | 5.1827 | 5.2823 | 5.2603 | 5.3002 | 5.3376 | 5.3337 | 5.3353 |
| pos_3502 | PC(14:1(9Z)/20:1(11Z)) | metab_2614 | HMDB0007912;LMGP01011398;PW_C003962 | C00157 | 780.5533 | 7.0491 | pos | M+Na | C42H80NO8P | 0 | 91.2 | 2.5413418 | - | 0.02376219 | 6.6133 | 7.3013 | 7.2306 | 7.3086 | 6.9409 | 7.0145 | 7.3064 | 7.2869 | 7.1099 | 7.3186 | 7.1017 | 7.0181 | 7.1302 | 7.1107 | 7.1148 |
| pos_3503 | PC(18:0/14:1(9Z)) | metab_2615 | HMDB0008032;PW_C004081;LMGP01011582 | C00157 | 754.5362 | 7.0491 | pos | M+Na | C40H78NO8P | 0 | 70.5 | 0.65249566 | - | 0.14210491 | 5.0307 | 5.556 | 5.4396 | 5.7993 | 5.3091 | 5.4477 | 5.6084 | 5.3695 | 5.3659 | 5.7633 | 5.2193 | 5.3253 | 5.2857 | 5.195 | 5.1725 |
| pos_3504 | 4-Hydroxycinnamoylagmatine | metab_2616 | HMDB0033460 | C04498 | 594.3544 | 7.0491 | pos | 2M+ACN+H | C14H20N4O2 | 0 | 83.4 | 5.98831428 | 7295-86-5 | 0.01362059 | 5.4557 | 4.6887 | 3.7575 | 5.0768 | 4.3122 | 4.2303 | 4.9804 | 4.2808 | 3.6835 | 4.834 | 4.2718 | 4.546 | 4.9867 | 4.991 | 4.9984 |
| pos_3505 | LysoPE(0:0/20:2(11Z,14Z)) | metab_2617 | HMDB0011483 | - | 488.3121 | 7.0491 | pos | M+H-H2O | C25H48NO7P | 0 | 75.1 | -2.8997137 | - | 0.02342829 | 4.8396 | 3.6879 | 4.3649 | 3.7996 | 3.6929 | 4.1099 | 4.111 | 4.2593 | 3.6963 | 4.4832 | 3.7064 | 3.9857 | 5.2859 | 5.2995 | 5.3058 |
| pos_3506 | PC(16:0/0:0) | metab_2618 | LMGP01050018;HMDB0010382 | C04230 | 518.323 | 7.0491 | pos | M+Na | C24H50NO7P | 93.6 | 0 | 2.69173738 | 17364-16-8 | 0.01799171 | 7.6346 | 7.2059 | 7.0964 | 7.3737 | 6.9017 | 6.8967 | 7.1909 | 7.1472 | 6.9732 | 7.1564 | 6.9192 | 6.9217 | 7.2935 | 7.3063 | 7.3078 |
| pos_3523 | Bis(2-ethylhexyl) phthalate | metab_2635 | HMDB0249243 | C03690 | 413.2671 | 7.0332 | pos | M+Na | C24H38O4 | 0 | 33.8 | 2.1902943 | - | 0.01907563 | 5.7267 | 5.4312 | 5.3891 | 5.4927 | 5.2694 | 5.3661 | 5.3593 | 5.3086 | 5.2926 | 5.5045 | 5.1713 | 5.2511 | 5.6811 | 5.6947 | 5.6963 |
| pos_3526 | DG(18:1(11Z)/16:0/0:0) | metab_2638 | HMDB0007185;PW_C003236 | C00165 | 636.5576 | 7.0253 | pos | M+ACN+H | C37H70O5 | 0 | 60.8 | 2.37784184 | - | 0.00195938 | 5.9525 | 5.9171 | 5.877 | 5.7951 | 5.6047 | 5.6948 | 5.7846 | 5.7429 | 5.8168 | 5.8729 | 5.6406 | 5.692 | 5.1427 | 5.1425 | 5.1411 |
| pos_3529 | Oryzalexin E | metab_2641 | HMDB0039702 | C21561 | 337.2743 | 7.0253 | pos | M+CH3OH+H | C20H32O2 | 0 | 70.5 | 1.85583891 | 150943-96-7 | 0.00158473 | 5.8976 | 5.8536 | 5.8075 | 5.7754 | 5.633 | 5.7468 | 5.7853 | 5.7564 | 5.7993 | 5.8507 | 5.6488 | 5.7197 | 5.5734 | 5.5722 | 5.5721 |
| pos_3542 | 4-Dodecan-6-ylbenzenesulfonic Acid | metab_2655 | HMDB0246414 | - | 344.2286 | 7.0093 | pos | M+NH4 | C18H30O3S | 0 | 35.8 | 9.97884877 | - | 0.01026576 | 4.8645 | 4.887 | 4.7984 | 4.7785 | 4.8222 | 4.8875 | 4.8027 | 4.7762 | 4.7641 | 4.8866 | 4.8121 | 4.7989 | 4.8453 | 4.8451 | 4.8375 |
| pos_3547 | DG(18:0/18:3(9Z,12Z,15Z)/0:0) | metab_2659 | PW_C003214;HMDB0007163 | C00165 | 619.5308 | 7.0013 | pos | M+H | C39H70O5 | 0 | 43.2 | 1.92046835 | - | 0.05296778 | 5.5218 | 5.5258 | 5.425 | 5.5257 | 5.2437 | 5.3477 | 5.3838 | 5.3786 | 5.4595 | 5.5137 | 5.2793 | 5.2731 | 3.7669 | 3.7269 | 3.7284 |
| pos_3566 | PC(6 keto-PGF1alpha/20:0) | metab_2674 | HMDB0286963 | - | 917.631 | 6.9693 | pos | 2M+3H2O+2H | C48H90NO12P | 0 | 48.9 | -0.6501959 | - | 0.00552888 | 5.878 | 5.7955 | 5.8513 | 5.8877 | 6.0895 | 6.0615 | 5.8622 | 6.0229 | 5.9675 | 6.0926 | 6.001 | 5.9925 | 5.9237 | 5.9189 | 5.9206 |
| pos_3573 | PS(24:0/PGF1alpha) | metab_2682 | HMDB0283699 | - | 961.6566 | 6.9533 | pos | 2M+3H2O+2H | C50H94NO13P | 0 | 72.3 | -1.2708818 | - | 0.02899957 | 5.6804 | 5.5154 | 5.738 | 5.6936 | 6.0361 | 5.8414 | 5.7281 | 5.8772 | 5.5919 | 5.9204 | 5.7941 | 5.8556 | 5.7133 | 5.733 | 5.7099 |
| pos_3587 | 5-Hydroxyvalproic acid | metab_2694 | HMDB0013898;PW_C008640 | C16650 | 125.0966 | 6.9375 | pos | M+H-2H2O | C8H16O3 | 0 | 32.9 | 3.20178995 | 53660-23-4 | 0.04631901 | 4.4415 | 4.2409 | 4.2851 | 4.2724 | 4.2047 | 4.3155 | 4.3111 | 4.313 | 4.2774 | 4.3338 | 4.23 | 4.3109 | 4.364 | 4.3987 | 4.3999 |
| pos_3598 | 8-Methylnonenoate | metab_2705 | HMDB0012183;LMFA01030994 | C18202 | 341.2693 | 6.8976 | pos | 2M+H | C10H18O2 | 0 | 36 | 1.92712406 | 59320-77-3 | 0.01703218 | 4.7342 | 4.5932 | 4.5951 | 4.455 | 4.7219 | 4.7654 | 4.7373 | 4.724 | 4.7236 | 4.7879 | 4.7387 | 4.8216 | 4.9041 | 4.9187 | 4.9137 |
| pos_36 | Tetraethylene glycol monododecyl ether | metab_2707 | HMDB0246913 | - | 385.2955 | 6.7298 | pos | M+Na, M+2Na-H, M+NH4, M+H | C20H42O5 | 0 | 35.2 | 8.49797656 | - | 0.00576401 | 5.8938 | 5.8097 | 5.8351 | 5.9238 | 5.7482 | 5.8068 | 5.8011 | 5.8451 | 5.7843 | 5.8571 | 5.7458 | 5.77 | 5.9111 | 5.9144 | 5.916 |
| pos_3600 | (2S)-2-Hydroxyoctadecanoylcarnitine | metab_2709 | HMDB0241527 | - | 476.3955 | 6.8897 | pos | M+CH3OH+H | C25H49NO5 | 0 | 35.8 | 2.2101961 | - | 0.01535156 | 5.8078 | 5.7985 | 5.8111 | 5.811 | 5.7665 | 5.841 | 5.6876 | 5.7834 | 5.7886 | 5.9517 | 5.7964 | 5.7687 | 5.9057 | 5.9178 | 5.9071 |
| pos_3626 | PC(20:1(11Z)/18:4(6Z,9Z,12Z,15Z)) | metab_2735 | LMGP01011821;PW_C004354;HMDB0008306 | C00157 | 830.5701 | 6.8418 | pos | M+Na | C46H82NO8P | 0 | 87.3 | 3.75619831 | - | 0.16358705 | 5.4609 | 6.0581 | 6.083 | 5.996 | 5.469 | 5.9498 | 6.1976 | 6.2934 | 6.0733 | 6.316 | 5.9 | 6.0005 | 6.1383 | 6.0076 | 6.1372 |
| pos_3633 | Valproic acid | metab_2743 | LMFA01020291;HMDB0000901;PW_C001249;HMDB0001877 | C07185 | 109.1019 | 6.8418 | pos | M+H-2H2O | C8H16O2 | 0 | 31.1 | 4.81667583 | 99-66-1;60113-83-9 | 0.01427993 | 4.2324 | 4.1706 | 4.1376 | 4.1349 | 3.995 | 4.1924 | 4.1117 | 4.1359 | 4.1489 | 4.3247 | 4.1201 | 4.1973 | 4.2969 | 4.2859 | 4.2866 |
| pos_364 | 3-O-Methylniveusin A | metab_2749 | HMDB0039084 | - | 409.1879 | 3.2824 | pos | M+H, M+K | C21H28O8 | 0 | 31.3 | 5.32647069 | - | 0.00746482 | 5.4003 | 6.0277 | 5.7963 | 5.7523 | 5.5507 | 5.3738 | 6.0215 | 5.6067 | 5.4055 | 5.4347 | 5.5324 | 5.6735 | 5.7103 | 5.7165 | 5.7117 |
| pos_3640 | PE-NMe(20:1(11Z)/14:0) | metab_2750 | HMDB0113308;PW_C059432 | C01241 | 696.5272 | 6.8258 | pos | M+H-2H2O | C40H78NO8P | 0 | 66.4 | -7.4380292 | - | 0.01429578 | 6.1823 | 5.906 | 6.0746 | 5.788 | 6.4031 | 6.1319 | 5.8588 | 6.0655 | 6.1207 | 6.2775 | 6.1365 | 6.0494 | 6.1431 | 6.1544 | 6.1444 |
| pos_3648 | Estradiol cypionate | metab_2758 | HMDB0251962 | C14640 | 438.299 | 6.8178 | pos | M+ACN+H | C26H36O3 | 0 | 40.1 | -3.2550938 | 313-06-4 | 0.03631462 | 5.2934 | 4.9015 | 5.2902 | 5.2159 | 5.0467 | 5.1877 | 5.237 | 5.1287 | 5.0325 | 5.114 | 4.7831 | 5.2151 | 5.1799 | 5.2074 | 5.1809 |
| pos_3649 | Grotan | metab_2759 | HMDB0243492 | - | 283.1757 | 6.8178 | pos | M+ACN+Na | C9H21N3O3 | 0 | 32.9 | 7.55068882 | - | 0.00984601 | 4.9593 | 4.8688 | 4.9252 | 4.9142 | 4.7762 | 4.9215 | 4.9568 | 4.8646 | 4.9044 | 4.982 | 4.8931 | 4.9423 | 4.8979 | 4.9059 | 4.8993 |
| pos_365 | Gamma-Glu-leu | metab_2760 | HMDB0011171 | - | 261.1452 | 3.5918 | pos | M+H, M+Na | C11H20N2O5 | 92.1 | 0 | 2.67470316 | 2566-39-4 | 0.01045088 | 5.4515 | 5.6272 | 5.5139 | 5.2357 | 5.3554 | 5.2318 | 5.4985 | 5.3668 | 5.217 | 5.3001 | 5.1856 | 5.4147 | 5.4325 | 5.4323 | 5.4402 |
| pos_3650 | DG(11D5/9D3/0:0) | metab_2761 | PW_C063622;HMDB0116384 | C00165 | 714.5412 | 6.8098 | pos | 2M+3H2O+2H | C43H72O7 | 0 | 31 | 2.59698474 | - | 0.08159414 | 6.5123 | 6.5368 | 6.5388 | 6.6535 | 6.6415 | 6.6233 | 6.5237 | 6.6246 | 6.4837 | 6.6022 | 6.5797 | 6.6755 | 6.6348 | 6.6309 | 6.57 |
| pos_3659 | LysoPA(O-18:0/0:0) | metab_2770 | HMDB0011144 | - | 466.3302 | 6.7938 | pos | M+ACN+H | C21H45O6P | 0 | 43.5 | 2.377561 | - | 0.0028867 | 5.9007 | 6.0005 | 6.0518 | 6.0547 | 5.9224 | 5.9484 | 6.0016 | 5.7803 | 5.9683 | 5.9712 | 5.8495 | 5.8993 | 6.772 | 6.7745 | 6.7734 |
| pos_3662 | PE(20:5(5Z,8Z,11Z,14Z,17Z)/20:1(11Z)) | metab_2774 | HMDB0009461;LMGP02010981;PW_C005509 | C00350 | 824.5757 | 6.7858 | pos | M+CH3OH+H | C45H78NO8P | 0 | 64.2 | -5.4780531 | - | 0.00868389 | 6.4854 | 6.2035 | 6.331 | 6.1009 | 6.6132 | 6.3768 | 6.2022 | 6.351 | 6.439 | 6.5642 | 6.458 | 6.3558 | 6.3335 | 6.3391 | 6.3407 |
| pos_3665 | PS(20:1(11Z)/22:1(13Z)) | metab_2777 | HMDB0112559;PW_C060971 | C02737 | 872.6327 | 6.7778 | pos | M+H | C48H90NO10P | 0 | 56.3 | -5.5374673 | - | 0.02099671 | 6.3942 | 6.0784 | 6.2414 | 5.8492 | 6.5841 | 6.244 | 6.084 | 6.242 | 6.3698 | 6.4805 | 6.3709 | 6.1642 | 6.263 | 6.2627 | 6.2785 |
| pos_3673 | PS(20:1(11Z)/20:0) | metab_2785 | PW_C060962;LMGP03010547;HMDB0112551 | C02737 | 868.602 | 6.7698 | pos | M+Na | C46H88NO10P | 0 | 67.6 | -2.1594111 | - | 0.00311692 | 6.5336 | 6.2696 | 6.3449 | 6.1122 | 6.6687 | 6.4072 | 6.2496 | 6.4117 | 6.4987 | 6.612 | 6.5116 | 6.3794 | 6.3662 | 6.3652 | 6.3679 |
| pos_3681 | 3-Hydroxyphenylacetic Acid | metab_2794 | HMDB0000440 | C05593 | 153.055 | 6.7698 | pos | M+H | C8H8O3 | 95.7 | 0 | 2.66604365 | 621-37-4 | 0.01107919 | 4.637 | 4.6436 | 4.5749 | 4.6567 | 4.3908 | 4.5805 | 4.5995 | 4.6158 | 4.5889 | 4.6261 | 4.5584 | 4.5696 | 4.6792 | 4.6865 | 4.6883 |
| pos_369 | Fusaric Acid | metab_2802 | HMDB0252556 | C10146 | 197.1292 | 3.2824 | pos | M+H-2H2O, M+NH4 | C10H13NO2 | 67.6 | 0 | 4.119163 | 536-69-6 | 0.00322359 | 5.9981 | 6.1558 | 6.0643 | 6.0373 | 6.0009 | 5.9516 | 6.2088 | 6.0284 | 6.0692 | 6.0178 | 6.1435 | 6.1314 | 6.0855 | 6.0841 | 6.0869 |
| pos_3691 | PC(18:0/22:5(7Z,10Z,13Z,16Z,19Z)) | metab_2804 | PW_C004105;LMGP01010818;HMDB0008056 | C00157 | 836.6184 | 6.7538 | pos | M+H | C48H86NO8P | 0 | 42.5 | 2.42423279 | - | 0.06340856 | 6.6628 | 5.4277 | 6.724 | 6.9853 | 6.2993 | 6.5439 | 6.6269 | 6.6329 | 6.5745 | 6.7802 | 6.6997 | 6.5184 | 6.8163 | 6.7681 | 6.8172 |
| pos_37 | Corchorifatty acid F | metab_2812 | HMDB0035919 | - | 351.215 | 6.2841 | pos | M+Na, M+ACN+H, M+H-2H2O, M+H-H2O | C18H32O5 | 0 | 54 | 2.32479279 | 95341-44-9 | 0.01862775 | 4.9519 | 5.0529 | 5.0493 | 4.9491 | 4.6724 | 4.2137 | 5.263 | 4.8016 | 4.9042 | 5.0015 | 4.8851 | 4.9948 | 5.2255 | 5.2252 | 5.2393 |
| pos_3702 | PE(16:0/0:0) | metab_2816 | HMDB0011503;LMGP02050002 | - | 454.2938 | 6.7458 | pos | M+H | C21H44NO7P | 59.9 | 0 | 2.24740616 | 53862-35-4 | 0.01390277 | 5.0074 | 5.0595 | 4.9205 | 4.9473 | 5.1089 | 5.1636 | 5.0569 | 5.1003 | 5.1294 | 4.8595 | 4.972 | 5.0234 | 4.9434 | 4.9319 | 4.9409 |
| pos_3711 | PC(14:1(9Z)/20:2(11Z,14Z)) | metab_2826 | PW_C003963;LMGP01011399;HMDB0007913 | C00157 | 738.5365 | 6.7298 | pos | M+H-H2O | C42H78NO8P | 0 | 46.3 | -8.9481307 | - | 0.00905642 | 4.361 | 4.0568 | 3.8302 | 3.978 | 4.581 | 4.2116 | 4.1265 | 4.0717 | 4.2625 | 4.468 | 4.5083 | 4.1809 | 5.5409 | 5.5407 | 5.5476 |
| pos_3722 | PC(15:0/0:0) | metab_2837 | HMDB0010381;LMGP01050016;HMDB0010375 | C04230 | 504.3074 | 6.7218 | pos | M+Na | C23H48NO7P | 62.3 | 0 | 2.83544415 | - | 0.00776899 | 6.399 | 6.4599 | 6.3172 | 6.2786 | 6.2807 | 6.3407 | 6.2553 | 6.2104 | 6.4116 | 6.3288 | 6.2972 | 6.1625 | 6.3822 | 6.387 | 6.3888 |
| pos_3724 | (+)-Dehydrovomifoliol | metab_2839 | HMDB0302822 | C02533 | 255.1596 | 6.7218 | pos | M+CH3OH+H | C13H18O3 | 0 | 47.5 | 2.51966725 | - | 0.03871074 | 4.8733 | 4.7901 | 4.802 | 4.9366 | 4.6878 | 4.7726 | 4.7932 | 4.8031 | 4.7425 | 4.8151 | 4.8189 | 4.7441 | 4.8471 | 4.8472 | 4.8759 |
| pos_3729 | Anisperimus | metab_2844 | HMDB0248445 | - | 402.3222 | 6.7138 | pos | M+H | C18H39N7O3 | 0 | 49.2 | 8.68312089 | - | 0.0067674 | 5.854 | 5.7997 | 5.805 | 5.8859 | 5.6303 | 5.7825 | 5.7982 | 5.82 | 5.8081 | 5.8799 | 5.7337 | 5.7483 | 5.8948 | 5.8906 | 5.8962 |
| pos_3730 | PS(20:2(11Z,14Z)/20:0) | metab_2846 | HMDB0112578;LMGP03010577;PW_C060991 | C02737 | 826.5874 | 6.7058 | pos | M+H-H2O | C46H86NO10P | 0 | 32.2 | -9.804062 | - | 0.0309018 | 4.6305 | 4.2918 | 4.3655 | 3.998 | 4.8037 | 4.6807 | 4.2664 | 4.3288 | 4.7719 | 4.681 | 4.498 | 4.4569 | 5.3349 | 5.3123 | 5.3365 |
| pos_3737 | PS(20:2(11Z,14Z)/22:1(13Z)) | metab_2853 | PW_C061000;HMDB0112586 | C02737 | 870.6148 | 6.6978 | pos | M+H | C48H88NO10P | 0 | 46.4 | -8.1187812 | - | 0.02173955 | 4.6326 | 4.3243 | 4.4948 | 4.2523 | 4.8758 | 4.4272 | 4.5153 | 4.4227 | 4.6969 | 4.7106 | 4.5718 | 4.5428 | 5.0865 | 5.0776 | 5.0964 |
| pos_374 | Methyl cellulose | metab_2856 | HMDB0029925 | - | 472.2766 | 3.7702 | pos | M+H, M+NH4 | C20H38O11 | 0 | 45.2 | 3.01146813 | 9004-67-5 | 0.00070046 | 6.2801 | 6.2904 | 6.3762 | 6.3108 | 6.4292 | 6.2683 | 6.3386 | 6.2936 | 6.2383 | 6.2347 | 6.3508 | 6.3607 | 6.2526 | 6.2524 | 6.252 |
| pos_3751 | FAHFA(16:0/9-O-18:0) | metab_2868 | HMDB0112106;LMFA07090004 | - | 556.5311 | 6.6818 | pos | M+NH4 | C34H66O4 | 0 | 62.9 | 2.10574542 | 1481636-31-0 | 0.02423814 | 5.5448 | 5.2284 | 5.4308 | 5.5507 | 5.2589 | 5.4601 | 5.4797 | 5.3968 | 5.5188 | 5.6286 | 5.4399 | 5.3267 | 5.5663 | 5.5662 | 5.5843 |
| pos_3756 | Artemisinic acid | metab_2872 | LMPR0103190015;HMDB0248622 | C20309 | 532.3414 | 6.6739 | pos | 2M+ACN+Na | C15H22O2 | 0 | 43.8 | 3.60964411 | 80286-58-4 | 0.0074175 | 4.739 | 5.8229 | 5.473 | 5.7496 | 5.2421 | 5.7096 | 5.4449 | 5.562 | 5.7178 | 5.3974 | 5.1179 | 5.2382 | 5.4374 | 5.4432 | 5.4378 |
| pos_3781 | LysoPC(16:1(9Z)/0:0) | metab_2895 | HMDB0010383 | C04230 | 516.3076 | 6.6419 | pos | M+Na | C24H48NO7P | 0 | 89.7 | 3.0809009 | 76790-27-7 | 0.00749622 | 6.3521 | 6.5352 | 6.4443 | 6.6277 | 6.304 | 6.3744 | 6.3501 | 6.5181 | 6.4104 | 6.4109 | 6.2447 | 6.1548 | 6.4098 | 6.4145 | 6.4161 |
| pos_3782 | MG(20:3(5Z,8Z,14Z)-O(11S,12R)/0:0/0:0) | metab_2896 | HMDB0260446 | - | 427.3062 | 6.6419 | pos | M+CH3OH+H | C23H38O5 | 0 | 46.1 | 2.10820957 | - | 0.04268923 | 4.7798 | 5.0528 | 4.9368 | 5.0578 | 4.2893 | 5.0114 | 4.9719 | 4.955 | 5.0061 | 5.0554 | 4.7926 | 4.9546 | 5.1141 | 5.1151 | 5.0821 |
| pos_3785 | CL(11:0/i-18:0/18:2(9Z,11Z)/21:0) | metab_2899 | HMDB0200384 | - | 725.4859 | 6.6339 | pos | M+2Na | C77H146O17P2 | 0 | 32 | -7.2121108 | - | 0.03505403 | 5.7462 | 5.6504 | 5.7439 | 5.3813 | 5.9098 | 5.7206 | 5.4925 | 5.7053 | 5.7286 | 5.7225 | 5.7536 | 5.6375 | 5.7674 | 5.7404 | 5.7424 |
| pos_3789 | MG(0:0/20:3(6,8,11)-OH(5)/0:0) | metab_2903 | HMDB0260564 | - | 414.3223 | 6.6339 | pos | M+NH4 | C23H40O5 | 0 | 34.3 | 2.29663374 | - | 0.00498277 | 4.8713 | 4.989 | 4.9528 | 5.0284 | 4.5024 | 4.8069 | 4.978 | 5.0281 | 4.9204 | 4.9651 | 4.8758 | 4.8691 | 5.0762 | 5.0734 | 5.0776 |
| pos_38 | Citral propylene glycol acetal | metab_2915 | HMDB0037286 | - | 228.1964 | 6.2205 | pos | M+H-2H2O, M+NH4, M+H, M+H-H2O | C13H22O2 | 0 | 30.3 | 2.97715233 | 10444-50-5 | 0.00225897 | 7.0234 | 7.0282 | 7.028 | 7.0289 | 6.98 | 7.0334 | 7.0401 | 7.0401 | 7.0468 | 7.0594 | 7.0016 | 7.0228 | 7.0703 | 7.0721 | 7.0717 |
| pos_3803 | (17alpha,23S)-17,23-Epoxy-29-hydroxy-27-norlanosta-1,8-diene-3,15,24-trione | metab_2920 | HMDB0035971 | - | 507.2495 | 6.618 | pos | M+K | C29H40O5 | 0 | 45.1 | -2.7043781 | - | 0.03073025 | 5.3866 | 5.293 | 5.1134 | 5.3351 | 5.1666 | 5.2061 | 5.2574 | 5.2735 | 5.2298 | 5.2493 | 5.3182 | 5.3121 | 5.1761 | 5.1996 | 5.1774 |
| pos_3815 | N-Oleoyl Arginine | metab_2931 | HMDB0241954 | - | 502.3749 | 6.602 | pos | M+ACN+Na | C24H46N4O3 | 0 | 33.6 | 4.92153934 | - | 0.00667203 | 6.1188 | 6.1574 | 6.1585 | 6.2557 | 6.1018 | 6.2071 | 6.1708 | 6.1867 | 6.1274 | 6.1906 | 6.163 | 6.176 | 6.2402 | 6.2401 | 6.2351 |
| pos_3824 | Polypropylene | metab_2941 | HMDB0255988 | C19505 | 372.348 | 6.594 | pos | M+NH4 | C22H42O3 | 0 | 44 | 2.1070878 | 9003-07-0 | 0.00464807 | 5.4952 | 5.5765 | 5.5615 | 5.6027 | 5.3604 | 5.5704 | 5.5077 | 5.5787 | 5.4998 | 5.5332 | 5.4957 | 5.4751 | 5.5954 | 5.5994 | 5.5967 |
| pos_383 | 2,8-Dihydroxyquinoline-beta-D-glucuronide | metab_2947 | HMDB0011658 | - | 338.0878 | 3.7782 | pos | M+H, M+Na | C15H15NO8 | 0 | 59.2 | 2.28391924 | - | 0.00260137 | 4.8594 | 5.2348 | 5.3503 | 5.0023 | 6.438 | 6.4616 | 5.1569 | 4.9942 | 6.4346 | 4.4689 | 6.4532 | 6.51 | 5.9259 | 5.9237 | 5.9253 |
| pos_3856 | PE(18:0/5-iso PGF2VI) | metab_2975 | HMDB0261134 | - | 803.526 | 6.5619 | pos | 2M+3H2O+2H | C41H76NO11P | 0 | 65.1 | -1.4465733 | - | 0.00120961 | 5.8047 | 5.5685 | 5.6629 | 5.1102 | 5.9499 | 5.6502 | 5.4875 | 5.5547 | 5.8337 | 5.8368 | 5.7684 | 5.5039 | 6.1043 | 6.1053 | 6.1045 |
| pos_3863 | Linolenic Acid | metab_2983 | HMDB0001388 | C06427 | 279.2325 | 6.5619 | pos | M+H | C18H30O2 | 42.7 | 0 | 2.26620473 | 463-40-1 | 0.02375517 | 5.0849 | 5.2197 | 5.137 | 5.1219 | 4.9241 | 4.9417 | 5.238 | 5.0371 | 5.0971 | 4.9643 | 4.9019 | 5.1239 | 5.2395 | 5.2393 | 5.2571 |
| pos_3885 | Terbutaline | metab_3005 | HMDB0015009 | C07129 | 492.31 | 6.5379 | pos | 2M+ACN+H | C12H19NO3 | 0 | 41.3 | 7.03833995 | 23031-25-6 | 0.03493365 | 5.5405 | 5.9034 | 5.717 | 5.7545 | 5.8225 | 5.8044 | 5.7441 | 5.7316 | 5.8689 | 5.6554 | 5.7109 | 5.635 | 5.7093 | 5.7349 | 5.7366 |
| pos_39 | 3-Oxo-alpha-ionol | metab_3020 | HMDB0303805 | - | 226.1808 | 6.1729 | pos | M+H-2H2O, M+H, M+H-H2O, M+NH4 | C13H20O2 | 0 | 66.2 | 2.92541986 | - | 0.00111827 | 6.2719 | 6.2837 | 6.2604 | 6.2394 | 6.236 | 6.3069 | 6.3158 | 6.2455 | 6.298 | 6.3148 | 6.2746 | 6.25 | 6.2883 | 6.2892 | 6.2889 |
| pos_3900 | Cyanidin 3,5-O-diglucoside | metab_3022 | HMDB0304306;HMDB0304659 | C08639 | 742.5761 | 6.5222 | pos | M+H | C42H80NO7P | 0 | 46.3 | 2.18258285 | 20905-74-2 | 0.14124798 | 6.5056 | 6.0098 | 6.1489 | 5.0589 | 5.1274 | 5.5934 | 5.4219 | 5.5279 | 5.7107 | 5.2835 | 5.1987 | 5.3899 | 6.4536 | 6.4766 | 6.3563 |
| pos_3908 | PC(P-18:1(9Z)/14:1(9Z)) | metab_3030 | HMDB0011303 | - | 714.5408 | 6.5143 | pos | M+H | C40H76NO7P | 0 | 35.7 | -3.3496261 | - | 0.03221279 | 7.131 | 7.2373 | 7.2173 | 7.2193 | 7.2921 | 7.1982 | 7.153 | 7.1887 | 7.1108 | 7.1739 | 7.2272 | 7.2488 | 7.0984 | 7.098 | 7.0737 |
| pos_3934 | PE(15:0/18:3(9Z,12Z,15Z)) | metab_3055 | PW_C004945;HMDB0008897;LMGP02010460 | C00350 | 664.4648 | 6.4744 | pos | M+H-2H2O | C38H70NO8P | 0 | 62.6 | -7.4943896 | - | 0.01245645 | 6.4707 | 6.4556 | 6.4439 | 6.4434 | 6.372 | 6.4282 | 6.4233 | 6.4436 | 6.413 | 6.4522 | 6.4045 | 6.4093 | 6.4564 | 6.4664 | 6.458 |
| pos_3937 | Behenic acid | metab_3057 | HMDB0000944;HMDB0304269;LMFA01010022 | C08281 | 358.3687 | 6.4744 | pos | M+NH4 | C22H44O2 | 0 | 31.1 | 2.23373764 | 112-85-6 | 0.00298646 | 6.328 | 6.3595 | 6.3486 | 6.3591 | 6.3057 | 6.4157 | 6.3441 | 6.3687 | 6.3517 | 6.3731 | 6.3514 | 6.3154 | 6.4703 | 6.4725 | 6.4726 |
| pos_3940 | PE(20:4(8Z,11Z,14Z,17Z)/15:0) | metab_3060 | PW_C005465;HMDB0009417 | C00350 | 708.4912 | 6.4664 | pos | M+H-H2O | C40H72NO8P | 0 | 43.3 | -7.0363584 | - | 0.01661314 | 6.2641 | 6.2807 | 6.2625 | 6.2773 | 6.1451 | 6.2715 | 6.2901 | 6.2858 | 6.2617 | 6.239 | 6.2199 | 6.2212 | 6.265 | 6.278 | 6.2661 |
| pos_3943 | 6-Hydroxypentadecanedioic acid | metab_3063 | HMDB0031885 | - | 311.1832 | 6.4664 | pos | M+Na | C15H28O5 | 0 | 40.4 | 1.11826125 | 38076-52-7 | 0.00276142 | 5.3671 | 5.3921 | 5.3458 | 5.4187 | 5.2804 | 5.3683 | 5.3836 | 5.3653 | 5.3938 | 5.4164 | 5.3738 | 5.3285 | 5.394 | 5.3964 | 5.3954 |
| pos_3944 | N-Acetyl-b-glucosaminylamine | metab_3064 | HMDB0001104 | C01239 | 253.1414 | 6.4664 | pos | M+CH3OH+H | C8H16N2O5 | 0 | 83 | 9.22752183 | - | 0.02162918 | 5.0621 | 5.1015 | 5.1298 | 5.1335 | 4.8717 | 5.1104 | 5.0884 | 5.1012 | 5.1096 | 5.1207 | 5.0089 | 5.0238 | 5.114 | 5.0971 | 5.0988 |
| pos_3945 | PC(20:2(11Z,14Z)/18:4(6Z,9Z,12Z,15Z)) | metab_3065 | HMDB0008339;LMGP01011851;PW_C004387 | C00157 | 806.5707 | 6.4586 | pos | M+H | C46H80NO8P | 0 | 75.5 | 1.58811884 | - | 0.09268243 | 5.5829 | 6.3379 | 6.5516 | 6.3411 | 6.2107 | 6.3387 | 6.544 | 6.4841 | 6.5776 | 6.5264 | 6.263 | 6.3707 | 6.7365 | 6.663 | 6.7329 |
| pos_3946 | Taurolithocholic Acid | metab_3066 | HMDB0000722;LMST05040003;MJDBDZS0000023 | C02592 | 466.299 | 6.4586 | pos | M+H-H2O | C26H45NO5S | 33 | 0 | 0.89421114 | 516-90-5 | 0.00605244 | 5.489 | 5.2403 | 4.9112 | 4.7585 | 5.3109 | 5.4211 | 4.9681 | 4.8116 | 5.5179 | 5.2889 | 5.3253 | 5.3833 | 5.1865 | 5.1862 | 5.1909 |
| pos_395 | Alanylphenylalanine | metab_3070 | HMDB0028694 | - | 278.1506 | 4.5314 | pos | M+H, M+ACN+H | C12H16N2O3 | 0 | 43 | 2.93862236 | 3061-90-3 | 0.00111992 | 5.0092 | 5.3275 | 5.0001 | 5.2188 | 4.9756 | 5.4406 | 5.1072 | 4.833 | 4.5936 | 4.5342 | 4.9226 | 5.2097 | 5.2734 | 5.2731 | 5.2724 |
| pos_3950 | 11-Dehydro-thromboxane B2 | metab_3071 | HMDB0244347;PW_C002104;LMFA03030014;HMDB0004242;LMFA03030004 | C05964 | 351.2151 | 6.4506 | pos | M+H-H2O | C20H32O6 | 0 | 44 | -3.9662457 | 67910-12-7 | 0.01020453 | 5.4024 | 5.4911 | 5.4706 | 5.4846 | 5.4002 | 5.4636 | 5.504 | 5.4338 | 5.4338 | 5.4726 | 5.4075 | 5.4552 | 5.467 | 5.4753 | 5.4686 |
| pos_3968 | Tanacetol B | metab_3085 | HMDB0035075 | - | 297.2041 | 6.4266 | pos | M+H | C17H28O4 | 0 | 71.6 | -6.6217152 | 86787-28-2 | 0.00635573 | 5.9859 | 6.0125 | 6.0294 | 6.0383 | 5.9129 | 6.0035 | 6.0005 | 6.0275 | 6.0098 | 6.0247 | 5.9542 | 5.9698 | 6.0229 | 6.019 | 6.0243 |
| pos_3970 | Cis-5-Octenoic acid | metab_3088 | HMDB0032207;LMFA01030022 | - | 165.0887 | 6.4266 | pos | M+Na | C8H14O2 | 0 | 30.4 | 0.61946613 | 41653-97-8 | 0.00374608 | 5.5547 | 5.5949 | 5.5699 | 5.5804 | 5.5023 | 5.5831 | 5.5483 | 5.5512 | 5.5632 | 5.6114 | 5.552 | 5.5499 | 5.5862 | 5.5895 | 5.5877 |
| pos_3971 | 13-Hydroxyoctadecanoylcarnitine | metab_3089 | HMDB0241521 | - | 426.3585 | 6.4186 | pos | M+H-H2O | C25H49NO5 | 0 | 67.7 | 1.71215546 | - | 0.00249621 | 6.3387 | 5.6767 | 5.8671 | 6.0754 | 5.2124 | 5.212 | 6.0553 | 6.07 | 5.4998 | 6.1895 | 5.3612 | 5.3528 | 5.8849 | 5.8871 | 5.8858 |
| pos_3975 | 12-hydroxyicosanoic acid | metab_3091 | HMDB0061664 | - | 346.3325 | 6.4107 | pos | M+NH4 | C20H40O3 | 0 | 38.7 | 2.68110629 | - | 0.01011087 | 4.5946 | 4.9335 | 4.3898 | 4.5593 | 4.6548 | 4.7418 | 4.854 | 4.6029 | 4.5288 | 4.6669 | 4.9125 | 4.8276 | 5.2034 | 5.2032 | 5.2109 |
| pos_3976 | 9-Octadecen-1-ol, (9Z)- | metab_3092 | HMDB0247606 | - | 286.311 | 6.4107 | pos | M+NH4 | C18H36O | 0 | 59.4 | 2.16525164 | - | 0.0002807 | 4.7618 | 4.6573 | 4.7992 | 4.5911 | 4.7257 | 4.8677 | 4.6325 | 4.653 | 4.4146 | 4.9414 | 4.7972 | 4.8008 | 4.9643 | 4.964 | 4.964 |
| pos_3981 | Palmitoylcarnitine | metab_3098 | HMDB0240783;LMFA07070004;HMDB0000222 | C02990 | 400.3436 | 6.4029 | pos | M+H | C23H45NO4 | 68.7 | 0 | 3.70466839 | 2364-67-2 | 0.00955122 | 6.4663 | 5.9563 | 6.0653 | 6.186 | 5.5778 | 5.6799 | 6.1923 | 6.2201 | 5.8517 | 6.2343 | 5.7869 | 5.7419 | 6.0587 | 6.0535 | 6.0617 |
| pos_3986 | 2-Hydroxyundec-8-enoylcarnitine | metab_3103 | HMDB0241166 | - | 344.2435 | 6.3869 | pos | M+H | C18H33NO5 | 0 | 30.1 | 1.06069255 | - | 0.02364081 | 5.0125 | 5.1152 | 5.01 | 5.4177 | 4.7147 | 5.0026 | 5.0195 | 4.9453 | 4.9302 | 5.0434 | 4.8213 | 4.991 | 5.1597 | 5.1417 | 5.1424 |
| pos_3992 | VAPIPROST | metab_3110 | HMDB0259765 | - | 516.2555 | 6.3792 | pos | M+K | C30H39NO4 | 0 | 65.7 | 9.1983562 | - | 0.06556692 | 4.0728 | 5.1126 | 5.0633 | 4.401 | 4.9155 | 5.2365 | 5.3529 | 4.0949 | 5.2522 | 4.2084 | 4.8468 | 4.9958 | 5.4534 | 5.5028 | 5.5045 |
| pos_3994 | LysoPE(0:0/18:0) | metab_3112 | HMDB0011129 | - | 520.2808 | 6.3792 | pos | M+K | C23H48NO7P | 0 | 39.7 | 1.72864175 | 899443-67-5 | 0.02102583 | 5.7101 | 5.5912 | 5.6569 | 5.622 | 5.5443 | 5.5271 | 5.604 | 5.6107 | 5.4077 | 5.7496 | 5.4112 | 5.6737 | 5.4942 | 5.5093 | 5.5109 |
| pos_4 | Cholic Acid | metab_3117 | PW_C000480;HMDB0000619;LMST04010001;MJDBDZS0000046 | C00695 | 426.3222 | 6.3792 | pos | M+H-2H2O, 2M+NH4, 2M+H, M+Na, M+NH4, M+H-H2O | C24H40O5 | 78.7 | 0 | 2.07598259 | 81-25-4 | 0.00653703 | 6.4978 | 7.136 | 7.1104 | 6.6926 | 7.0197 | 7.3343 | 7.24 | 6.8285 | 7.4029 | 6.6229 | 7.0233 | 7.0799 | 7.427 | 7.4268 | 7.4318 |
| pos_40 | Ethyl beta-D-fructofuranoside | metab_3118 | HMDB0303035 | - | 231.0845 | 2.8468 | pos | M+H, M+Na, M+NH4, M+H-H2O | C8H16O6 | 0 | 50.6 | 3.03012209 | - | 0.00931977 | 0.4125 | 0.8742 | 0.4158 | 0.4284 | 3.3202 | 3.2022 | 0.5385 | 3.0498 | 2.5008 | 1.0251 | 3.2095 | 1.0807 | 5.8385 | 5.8383 | 5.8454 |
| pos_4002 | 4-hydroxysphinganine | metab_3122 | LMSP01030001;HMDB0304183 | C12144 | 282.2798 | 6.3792 | pos | M+H-2H2O | C18H39NO3 | 0 | 46.4 | 2.09317822 | - | 0.03853945 | 5.1581 | 4.6095 | 4.7263 | 5.0711 | 4.4826 | 4.5329 | 4.6375 | 4.8414 | 4.5868 | 4.9744 | 4.7225 | 4.8133 | 4.8697 | 4.8707 | 4.8409 |
| pos_4008 | Methyl (3b,11x)-3-Hydroxy-8-oxo-6-eremophilen-12-oate | metab_3128 | HMDB0041229 | - | 245.1542 | 6.3792 | pos | M+H-2H2O | C16H24O4 | 0 | 66.7 | 2.09636385 | 64964-00-7 | 0.01340805 | 4.6709 | 5.0386 | 4.9837 | 4.8401 | 4.9549 | 5.1159 | 5.119 | 4.7847 | 5.2136 | 4.814 | 4.9984 | 4.8733 | 5.2143 | 5.2172 | 5.2255 |
| pos_4011 | Dehydrocurdione | metab_3132 | HMDB0035407 | C16949 | 199.1487 | 6.3792 | pos | M+H-2H2O | C15H22O2 | 0 | 73.3 | 2.32375148 | 38230-32-9 | 0.02055576 | 3.831 | 4.4677 | 4.2518 | 3.9843 | 4.4066 | 4.6625 | 4.2947 | 3.8531 | 4.6689 | 3.8908 | 4.3269 | 4.2232 | 4.5731 | 4.589 | 4.5882 |
| pos_4013 | Cnidilide | metab_3134 | HMDB0302259 | C16937 | 159.1173 | 6.3792 | pos | M+H-2H2O | C12H18O2 | 0 | 70.8 | 2.19968572 | 3/1/74 | 0.03091753 | 4.1729 | 4.6504 | 4.5195 | 4.2553 | 4.6171 | 4.8806 | 4.7352 | 4.5073 | 4.8794 | 4.3836 | 4.6017 | 4.4895 | 4.8241 | 4.8341 | 4.8506 |
| pos_4014 | Jasmolone | metab_3135 | HMDB0030039 | - | 145.1016 | 6.3792 | pos | M+H-2H2O | C11H16O2 | 0 | 51.7 | 2.11854037 | 54383-66-3 | 0.01192877 | 4.5119 | 4.7236 | 4.6276 | 4.4542 | 4.6945 | 4.7898 | 4.7565 | 4.4672 | 4.975 | 4.6085 | 4.651 | 4.6422 | 4.9022 | 4.8939 | 4.9034 |
| pos_4023 | SM(d18:1/16:1(9Z)) | metab_3145 | HMDB0240613 | - | 701.5608 | 6.3712 | pos | M+H | C39H77N2O6P | 0 | 36.3 | 2.22948033 | 222403-66-9 | 0.05761615 | 4.8998 | 5.5776 | 4.9183 | 5.0113 | 5.217 | 5.302 | 4.9499 | 5.1088 | 5.3255 | 5.2879 | 4.9324 | 5.2191 | 6.0445 | 6.0239 | 5.9944 |
| pos_403 | Cellulose hydroxyethylate | metab_3150 | HMDB0249797 | - | 404.2336 | 4.7234 | pos | M+2H, M+H+Na | C36H70O19 | 0 | 42.7 | 3.49502068 | - | 0.00321422 | 5.9525 | 5.9775 | 6.0707 | 6.0097 | 6.0987 | 5.9593 | 6.0571 | 6.0171 | 5.9538 | 5.9184 | 6.0675 | 6.0989 | 5.8176 | 5.8162 | 5.819 |
| pos_4031 | 1-acyl-PAF | metab_3151 | HMDB0243815 | - | 538.3517 | 6.3633 | pos | M+H | C26H52NO8P | 0 | 59 | 2.49010631 | - | 0.00912355 | 3.979 | 5.631 | 5.3322 | 4.0045 | 5.0395 | 5.114 | 4.2367 | 4.001 | 5.4459 | 4.0042 | 5.4248 | 5.2614 | 5.2182 | 5.2257 | 5.2199 |
| pos_4039 | Cinncassiol D1 | metab_3158 | HMDB0034676 | C17652 | 353.2309 | 6.3554 | pos | M+H | C20H32O5 | 0 | 33.4 | -3.8069598 | 77353-84-5 | 0.02722646 | 5.08 | 5.2861 | 5.1681 | 4.7288 | 4.7975 | 4.5672 | 5.5805 | 5.0484 | 5.2997 | 4.6094 | 4.5028 | 5.3283 | 4.8852 | 4.8652 | 4.8864 |
| pos_4040 | Alpha-Bisabolol oxide A | metab_3160 | HMDB0038196 | C16773 | 256.2277 | 6.3554 | pos | M+NH4 | C15H26O2 | 0 | 56.9 | 2.30473802 | 22567-36-8 | 0.00486556 | 5.2497 | 5.349 | 5.3274 | 5.34 | 5.2793 | 5.2974 | 5.3827 | 5.3517 | 5.284 | 5.416 | 5.3181 | 5.2988 | 5.3504 | 5.3502 | 5.354 |
| pos_4043 | 4-[(2S)-2-Aminopropyl]-N,3-dimethylaniline | metab_3163 | HMDB0246317 | - | 398.3271 | 6.3476 | pos | 2M+ACN+H | C11H18N2 | 0 | 35.8 | -2.1513004 | - | 0.03103872 | 5.4738 | 5.0208 | 5.076 | 5.3136 | 4.4192 | 4.5493 | 5.3332 | 5.3534 | 4.8759 | 5.4053 | 4.6976 | 4.6931 | 5.2775 | 5.2545 | 5.2786 |
| pos_4044 | (9Z)-(13S)-12,13-epoxyoctadeca-9,11-dienoate | metab_3164 | HMDB0303983 | C04594 | 357.2255 | 6.3476 | pos | M+ACN+Na | C18H29O3- | 0 | 35 | -6.7635146 | - | 0.03051664 | 5.1199 | 5.2922 | 5.2923 | 5.1954 | 5.1892 | 5.3386 | 5.3043 | 5.2601 | 5.2168 | 5.2852 | 5.2206 | 5.3135 | 5.2909 | 5.2868 | 5.3114 |
| pos_4049 | Di-homo-gamma-linolenate | metab_3168 | HMDB0304332 | - | 674.5065 | 6.3396 | pos | 2M+ACN+Na | C20H33O2- | 0 | 44.5 | -8.8473102 | - | 0.09129929 | 5.4961 | 5.5465 | 5.5384 | 5.4764 | 5.7386 | 5.5859 | 5.5326 | 5.5657 | 5.4992 | 5.5576 | 5.5572 | 5.5986 | 5.2241 | 5.2238 | 5.1533 |
| pos_4050 | Nonadecanoic acid | metab_3170 | HMDB0000772;LMFA01010019 | C16535 | 316.3216 | 6.3396 | pos | M+NH4 | C19H38O2 | 0 | 56.6 | 2.15027561 | 646-30-0 | 0.12596047 | 4.6082 | 4.5315 | 4.4351 | 4.4532 | 4.8793 | 4.3496 | 4.0422 | 4.6335 | 4.6214 | 4.604 | 4.5083 | 4.6595 | 4.3193 | 4.3182 | 4.4105 |
| pos_4051 | Harmaline | metab_3171 | HMDB0030310;MJDBOTE0001002 | C06536 | 446.2546 | 6.3316 | pos | 2M+NH4 | C13H14N2O | 0 | 48.6 | -1.0883017 | 304-21-2 | 0.01425415 | 6.8828 | 6.7742 | 6.8033 | 6.832 | 6.727 | 6.7527 | 6.8116 | 6.8387 | 6.7731 | 6.9002 | 6.7728 | 6.7559 | 6.7702 | 6.7815 | 6.7716 |
| pos_4053 | Tetradecanoylcarnitine | metab_3172 | LMFA07070107;HMDB0005066;HMDB0254979;LMFA07070102 | - | 372.3115 | 6.3237 | pos | M+H | C21H41NO4 | 64.2 | 0 | 1.86918349 | 25597-07-3 | 0.00123552 | 5.4877 | 5.2346 | 5.1599 | 5.1563 | 3.7633 | 4.7525 | 5.2859 | 5.1756 | 5.0011 | 5.2259 | 4.8604 | 4.9547 | 5.3309 | 5.3299 | 5.33 |
| pos_4057 | Cucurbitacin S | metab_3176 | LMST01010116;HMDB0250583 | C08806 | 516.3332 | 6.3158 | pos | M+NH4 | C30H42O6 | 0 | 64.6 | 2.50382645 | 60137-06-6 | 0.01084333 | 6.6781 | 6.3566 | 6.3124 | 6.346 | 6.4748 | 6.3774 | 6.3399 | 6.4248 | 6.4612 | 6.5042 | 6.4232 | 6.4683 | 6.3058 | 6.3056 | 6.2975 |
| pos_4067 | Dopamine | metab_3185 | HMDB0000073;PW_C000052;MJDBSJD0000070 | C03758 | 118.0657 | 6.3158 | pos | M+H-2H2O | C8H11NO2 | 52.8 | 0 | 4.01566567 | 62-31-7;51-61-6 | 0.00176995 | 5.3423 | 5.2597 | 5.1989 | 5.2337 | 5.2283 | 5.2515 | 5.2642 | 5.287 | 5.2632 | 5.2943 | 5.2481 | 5.2789 | 5.2884 | 5.2893 | 5.2899 |
| pos_4068 | 4-Propylphenol | metab_3186 | HMDB0032625 | C14311 | 119.0861 | 6.3158 | pos | M+H-H2O | C9H12O | 0 | 58.6 | 4.36910048 | 645-56-7 | 0.00287014 | 7.0042 | 6.7741 | 6.7768 | 6.8002 | 6.7477 | 6.7646 | 6.8136 | 6.8521 | 6.8373 | 6.8933 | 6.8182 | 6.789 | 6.7497 | 6.7505 | 6.7522 |
| pos_4073 | Phytuberin | metab_3191 | HMDB0035754 | C09709 | 327.2147 | 6.3079 | pos | M+CH3OH+H | C17H26O4 | 0 | 49.8 | -6.4558873 | 37209-50-0 | 0.00992816 | 5.1884 | 5.2907 | 5.2825 | 5.3266 | 5.2371 | 5.2512 | 5.323 | 5.2996 | 5.2514 | 5.2273 | 5.158 | 5.2939 | 5.3342 | 5.3275 | 5.3357 |
| pos_4074 | Sphingosine | metab_3192 | HMDB0000252;LMSP01010001;PW_C000172 | C00319 | 300.2903 | 6.3079 | pos | M+H | C18H37NO2 | 90.6 | 0 | 2.0046447 | 123-78-4 | 0.00523578 | 4.6869 | 4.8631 | 4.6759 | 4.906 | 4.8337 | 4.9756 | 4.8679 | 4.7545 | 4.7856 | 4.92 | 4.9124 | 4.9659 | 5.0745 | 5.079 | 5.0758 |
| pos_4077 | Gibberellin A14 | metab_3195 | LMPR0104170015;HMDB0303454 | C11858 | 366.2258 | 6.2999 | pos | M+NH4 | C20H28O5 | 0 | 41.8 | -4.7406862 | 4955-22-0 | 0.0079655 | 4.7809 | 4.0639 | 5.0435 | 6.0683 | 3.9381 | 4.7338 | 5.2311 | 5.0174 | 4.4577 | 4.6761 | 3.9517 | 4.2249 | 5.0255 | 5.0306 | 5.0322 |
| pos_4078 | N-Myristoyl Aspartic acid | metab_3196 | HMDB0242048 | - | 344.2436 | 6.2999 | pos | M+H | C18H33NO5 | 0 | 47.6 | 1.41242154 | - | 0.13150347 | 4.6514 | 4.1999 | 4.7117 | 5.8278 | 3.9683 | 4.4227 | 5.0977 | 4.6406 | 3.9717 | 4.3954 | 4.0864 | 4.1118 | 4.8484 | 4.8484 | 4.7451 |
| pos_4086 | Isoalantolactone | metab_3205 | HMDB0035934 | C09484 | 233.1541 | 6.292 | pos | M+H | C15H20O2 | 68.7 | 0 | 2.31677393 | 470-17-7 | 0.02319731 | 3.8594 | 4.1464 | 3.6539 | 3.6657 | 4.1207 | 3.827 | 3.8476 | 3.9359 | 4.3167 | 3.6014 | 4.105 | 4.4547 | 4.5242 | 4.542 | 4.5252 |
| pos_4098 | PA(8:0/13:0) | metab_3218 | PW_C062910;HMDB0115484 | C00416 | 512.3362 | 6.2761 | pos | M+NH4 | C24H47O8P | 0 | 35.3 | 3.05333589 | - | 0.01916166 | 5.5336 | 5.6141 | 5.3604 | 5.2207 | 5.8052 | 5.6085 | 5.3456 | 5.1575 | 5.6945 | 5.012 | 5.6646 | 5.638 | 5.466 | 5.4822 | 5.4778 |
| pos_41 | Isoline | metab_3220 | HMDB0253654 | - | 418.1846 | 6.117 | pos | M+Na, M+K, M+H, M+H-H2O | C20H29NO7 | 0 | 62 | 2.49552167 | - | 0.00812438 | 5.7153 | 5.8214 | 5.7704 | 5.8152 | 5.8189 | 5.5709 | 5.8974 | 5.8089 | 5.8176 | 5.4577 | 5.8161 | 5.7747 | 5.3369 | 5.3436 | 5.3383 |
| pos_4110 | Triterpenoids | metab_3232 | HMDB0259282 | - | 536.3361 | 6.2682 | pos | M+ACN+Na | C29H44O5 | 0 | 36.1 | 3.08398435 | - | 0.01469448 | 4.12 | 5.5013 | 5.1218 | 4.6111 | 5.5005 | 5.1026 | 4.699 | 4.1378 | 5.5333 | 4.0112 | 5.4539 | 5.3175 | 5.07 | 5.0801 | 5.0819 |
| pos_4119 | Senkyunolide | metab_3241 | HMDB0258235 | C17853 | 225.1491 | 6.2682 | pos | M+CH3OH+H | C12H16O2 | 0 | 32.7 | 3.17096391 | 63038-10-8 | 0.01757181 | 4.8799 | 4.9376 | 4.8584 | 4.9151 | 4.8054 | 4.8159 | 4.9347 | 4.8569 | 4.8314 | 4.8444 | 4.8233 | 4.8843 | 4.8962 | 4.9085 | 4.9103 |
| pos_4127 | Tridecanoylcarnitine | metab_3250 | HMDB0241308 | - | 358.2959 | 6.2602 | pos | M+H | C20H39NO4 | 0 | 39.7 | 1.87412387 | - | 0.00742648 | 5.2606 | 4.7945 | 4.6491 | 4.7278 | 4.47 | 4.5668 | 4.9545 | 4.6247 | 4.5877 | 4.7638 | 4.6016 | 4.9191 | 5.1842 | 5.1841 | 5.1785 |
| pos_4131 | N-Palmitoyl Cysteine | metab_3254 | HMDB0241923 | - | 255.1597 | 6.2602 | pos | M+H | C14H22O4 | 0 | 33.6 | 2.33462864 | - | 0.00934045 | 4.8008 | 4.7514 | 4.7839 | 4.7226 | 4.6807 | 4.6871 | 4.6797 | 4.6642 | 4.7872 | 4.6887 | 4.719 | 4.7738 | 4.8172 | 4.8169 | 4.824 |
| pos_4141 | Prostaglandin G2 | metab_3264 | PW_C001814;HMDB0003235;LMFA03010009 | C05956 | 410.2524 | 6.2523 | pos | M+ACN+H | C20H32O6 | 0 | 83.4 | -3.4485922 | 51982-36-6 | 0.01458345 | 4.9856 | 5.3431 | 5.6163 | 5.5954 | 4.9057 | 4.8803 | 5.5328 | 5.0487 | 5.061 | 4.2298 | 4.8957 | 4.7663 | 5.0624 | 5.0622 | 5.0513 |
| pos_4147 | Atraton | metab_3269 | HMDB0248713 | C19098 | 445.2782 | 6.2443 | pos | 2M+Na | C9H17N5O | 0 | 45 | 5.53774733 | 1610-17-9 | 0.04289533 | 4.1451 | 5.3764 | 4.9792 | 4.4233 | 5.1094 | 5.1111 | 5.0154 | 4.5485 | 5.2149 | 4.7571 | 5.1116 | 5.1105 | 5.1938 | 5.2258 | 5.2271 |
| pos_4150 | 12S-HHT | metab_3273 | HMDB0012535 | C20388 | 313.2379 | 6.2443 | pos | M+CH3OH+H | C17H28O3 | 0 | 35 | 2.12302819 | 54397-84-1 | 0.01842415 | 4.5586 | 5.027 | 4.7012 | 4.217 | 4.8191 | 4.8241 | 4.7454 | 4.3773 | 4.9031 | 4.5239 | 4.7712 | 4.7826 | 4.951 | 4.9508 | 4.9369 |
| pos_4158 | Prostaglandin D1 | metab_3280 | HMDB0005102;LMFA03010049 | C06438 | 377.2308 | 6.2364 | pos | M+Na | C20H34O5 | 0 | 39.7 | 2.58774072 | 17968-82-0 | 0.03659873 | 4.0317 | 3.9359 | 4.0162 | 4.3786 | 3.6793 | 3.6965 | 4.3163 | 4.5005 | 3.7056 | 3.8549 | 3.6928 | 4.6896 | 4.6957 | 4.6686 | 4.697 |
| pos_4164 | O-(17-Carboxyheptadecanoyl)carnitine | metab_3286 | LMFA07070085;HMDB0240777 | - | 458.3486 | 6.2285 | pos | M+H | C25H47NO6 | 0 | 67.3 | 2.10438214 | - | 0.01311002 | 5.8903 | 5.723 | 5.602 | 5.5856 | 5.4913 | 5.3214 | 5.6774 | 5.5676 | 5.7459 | 5.6833 | 5.5759 | 5.5266 | 5.6413 | 5.6313 | 5.641 |
| pos_4167 | 12-oxo-PDA | metab_3289 | LMFA02010001;HMDB0301804;HMDB0302724 | C01226 | 310.2361 | 6.2285 | pos | M+NH4 | C18H28O3 | 0 | 52.4 | -5.5382289 | 85551-10-6 | 0.00355329 | 5.5501 | 5.4074 | 5.5061 | 5.5905 | 5.3281 | 5.3542 | 5.552 | 5.5915 | 5.3851 | 5.5333 | 5.3991 | 5.421 | 5.4904 | 5.4935 | 5.4919 |
| pos_4168 | 16-Hydroxyhexadecanoic acid | metab_3290 | HMDB0006294 | C18218 | 290.2695 | 6.2285 | pos | M+NH4 | C16H32O3 | 0 | 75.4 | 2.01100288 | 506-13-8 | 0.00350799 | 6.0838 | 6.233 | 5.9693 | 6.0423 | 6.0344 | 6.2407 | 6.2035 | 5.9573 | 6.0175 | 6.0953 | 6.2015 | 6.0807 | 6.1787 | 6.1784 | 6.1812 |
| pos_417 | 4-Hydroxyquinoline | metab_3292 | HMDB0246466 | C06343 | 146.0605 | 4.8665 | pos | M+H-H2O, M+H | C9H7NO | 56.9 | 0 | 3.3908102 | 611-36-9 | 0.00547296 | 4.5906 | 4.9973 | 4.8055 | 4.756 | 5.9978 | 5.9979 | 4.9096 | 4.7664 | 6.0091 | 4.5962 | 6.1432 | 6.0276 | 5.6528 | 5.6481 | 5.6498 |
| pos_4170 | 2',3'-Dideoxyadenosine | metab_3293 | HMDB0245544 | - | 253.1415 | 6.2285 | pos | M+NH4 | C10H13N5O2 | 0 | 65.7 | 3.16863254 | - | 0.01662503 | 5.0908 | 5.072 | 5.1301 | 5.1887 | 5.0149 | 5.0662 | 5.1479 | 5.1447 | 5.1579 | 5.1124 | 5.0647 | 5.1427 | 5.1983 | 5.1851 | 5.1867 |
| pos_4172 | 11-Hydroxy-9-tridecenoic acid | metab_3295 | LMFA01050437;HMDB0035881 | - | 229.1806 | 6.2285 | pos | M+H | C13H24O3 | 0 | 52.9 | 3.58593229 | 105798-56-9 | 0.00682853 | 5.0068 | 5.0115 | 5.0276 | 5.0392 | 4.9177 | 5.0275 | 5.0233 | 4.9874 | 5.0023 | 5.0216 | 5 | 5.026 | 5.0505 | 5.0466 | 5.0447 |
| pos_4174 | 4-Vinylcyclohexene | metab_3297 | HMDB0246592 | C19310 | 109.1019 | 6.2285 | pos | M+H | C8H12 | 0 | 40.6 | 6.45220122 | 100-40-3 | 0.00573535 | 4.9709 | 5.0523 | 5.0224 | 5.0318 | 4.9793 | 5.0716 | 5.0767 | 5.0162 | 5.0501 | 5.0351 | 4.9935 | 5.0354 | 5.1075 | 5.1123 | 5.1087 |
| pos_4182 | (4S,8R)-8,9-Dihydroxy-p-menth-1(6)-en-2-one | metab_3306 | HMDB0039052 | - | 207.0994 | 6.2205 | pos | M+Na | C10H16O3 | 0 | 60.8 | 1.13649279 | 402593-42-4 | 0.00915751 | 5.2474 | 5.237 | 5.2388 | 5.2525 | 5.1154 | 5.2636 | 5.2219 | 5.2189 | 5.2385 | 5.27 | 5.1625 | 5.1955 | 5.2566 | 5.2564 | 5.2634 |
| pos_4183 | Adenine | metab_3307 | PW_C000024;HMDB0000034;MJDBOTE0000759 | C00147 | 177.0888 | 6.2205 | pos | M+ACN+H | C5H5N5 | 0 | 39.1 | 3.3492579 | 73-24-5 | 0.00759011 | 5.8211 | 5.8094 | 5.8271 | 5.8282 | 5.7332 | 5.8154 | 5.8062 | 5.8241 | 5.8091 | 5.8416 | 5.7927 | 5.7986 | 5.8436 | 5.8484 | 5.85 |
| pos_4195 | 12-Hydroxydodecanoic acid | metab_3318 | HMDB0304547;HMDB0002059 | C08317 | 450.3787 | 6.1889 | pos | 2M+NH4 | C12H24O3 | 0 | 57.8 | -0.3969213 | 505-95-3 | 0.00425445 | 5.3309 | 5.1846 | 5.2054 | 5.2548 | 5.2172 | 5.2422 | 5.1798 | 5.2425 | 5.2637 | 5.3589 | 5.1356 | 5.116 | 5.2304 | 5.234 | 5.2318 |
| pos_4196 | Anabasine | metab_3319 | HMDB0004350 | C06180 | 342.2646 | 6.1889 | pos | 2M+NH4 | C10H14N2 | 0 | 47.1 | -2.0553236 | 13078-04-1 | 0.00483935 | 6.2251 | 5.0292 | 5.5403 | 5.4936 | 4.7938 | 5.1526 | 5.4518 | 5.6474 | 5.3849 | 5.7462 | 4.9911 | 5.527 | 5.5598 | 5.5627 | 5.5639 |
| pos_42 | 1-Octen-3-yl glucoside | metab_3322 | HMDB0032959 | - | 313.1628 | 6.0524 | pos | M+H-H2O, M+NH4, M+Na, M+H | C14H26O6 | 0 | 33.3 | 2.23771402 | 209863-00-3 | 0.01329193 | 4.7462 | 4.6049 | 4.6603 | 4.7254 | 4.6891 | 4.6377 | 4.5698 | 4.5576 | 4.5416 | 4.6126 | 4.6622 | 4.6199 | 5.6889 | 5.6797 | 5.6903 |
| pos_420 | Ganglioside GM3 (d18:0/20:0) | metab_3323 | HMDB0011919 | - | 628.3845 | 5.0193 | pos | M+2Na+H, M+2Na | C61H114N2O21 | 0 | 31.6 | -0.6211959 | - | 0.0094359 | 7.3482 | 7.4236 | 7.4768 | 7.4697 | 7.5135 | 7.4334 | 7.4966 | 7.3978 | 7.3933 | 7.3355 | 7.4573 | 7.5003 | 7.2702 | 7.2764 | 7.278 |
| pos_4209 | 20-Hydroxyeicosatetraenoic acid | metab_3333 | HMDB0005998;PW_C002614 | C14748 | 303.2323 | 6.1729 | pos | M+H-H2O | C20H32O3 | 0 | 80.5 | 1.23473771 | 79551-86-3 | 0.04162068 | 3.589 | 4.5522 | 3.8048 | 4.091 | 4.1384 | 4.1209 | 4.5352 | 3.9775 | 3.9372 | 3.7531 | 3.9565 | 3.7021 | 4.2867 | 4.3181 | 4.2876 |
| pos_4215 | Rimantadine | metab_3339 | HMDB0014621;MJDBOTE0001766 | C07236 | 180.1752 | 6.1729 | pos | M+H | C12H21N | 75.9 | 0 | 2.72329596 | 13392-28-4 | 0.0029054 | 5.025 | 5.01 | 4.9601 | 4.9683 | 4.979 | 5.0212 | 5.0459 | 5.0199 | 5.0368 | 5.0598 | 4.9938 | 4.9919 | 5.0341 | 5.0316 | 5.0332 |
| pos_4224 | N-Linoleoyl Histidine | metab_3347 | HMDB0241977 | - | 462.2683 | 6.1649 | pos | M+2Na-H | C24H39N3O3 | 0 | 61.8 | -4.8956826 | - | 0.00131842 | 6.4669 | 6.6739 | 5.8576 | 5.7622 | 6.9126 | 6.782 | 6.3214 | 5.9059 | 6.8041 | 6.7944 | 6.7285 | 6.5963 | 6.4142 | 6.414 | 6.4131 |
| pos_4227 | 16-B1-phytoprostane | metab_3350 | HMDB0304634 | - | 291.196 | 6.1649 | pos | M+H-H2O | C18H28O4 | 0 | 44.4 | 1.74263247 | - | 0.00592328 | 4.2411 | 4.2925 | 4.1902 | 3.9243 | 4.2165 | 4.2247 | 4.3715 | 4.2584 | 4.4529 | 3.9844 | 4.2122 | 4.5992 | 4.6155 | 4.6118 | 4.6168 |
| pos_4228 | Cedazuridine | metab_3351 | HMDB0304872 | - | 301.1189 | 6.1649 | pos | M+CH3OH+H | C9H14F2N2O5 | 0 | 55.9 | -6.3309567 | - | 0.05099885 | 4.3261 | 3.5131 | 4.249 | 4.2291 | 3.3726 | 3.0839 | 4.3156 | 4.3316 | 4.1226 | 4.4166 | 3.0806 | 3.6864 | 4.368 | 4.3666 | 4.4051 |
| pos_4246 | Micheliolide | metab_3370 | HMDB0254704 | C09507 | 249.1491 | 6.149 | pos | M+H | C15H20O3 | 0 | 69.9 | 2.52335528 | 68370-47-8 | 0.0561453 | 3.2794 | 3.7774 | 3.6138 | 3.5847 | 3.9774 | 3.9357 | 3.8214 | 3.1455 | 4.1447 | 3.7478 | 4.1142 | 4.2591 | 4.2834 | 4.2505 | 4.2987 |
| pos_4259 | Piliformic Acid | metab_3383 | - | - | 197.1179 | 6.141 | pos | M+H-H2O | C11H18O4 | 35.2 | 0 | 3.045277 | - | 0.03178951 | 3.6226 | 3.8265 | 3.8038 | 4.9113 | 3.5104 | 3.5869 | 3.6892 | 3.5288 | 3.9246 | 2.988 | 3.4882 | 3.6243 | 3.9352 | 3.9492 | 3.9216 |
| pos_4260 | Lovastatin acid | metab_3385 | HMDB0254177 | C21130 | 464.3015 | 6.133 | pos | M+ACN+H | C24H38O6 | 0 | 51.6 | 1.97323613 | 75225-51-3 | 0.00645683 | 4.8061 | 5.0479 | 4.3371 | 4.158 | 4.9825 | 5.4495 | 4.407 | 4.3481 | 5.3154 | 4.018 | 5.5027 | 4.9088 | 5.5806 | 5.5804 | 5.5756 |
| pos_4267 | Piperdial | metab_3390 | HMDB0035798 | C09711 | 233.1543 | 6.133 | pos | M+H-H2O | C15H22O3 | 0 | 66.7 | 2.86074062 | 100288-36-6 | 0.02802094 | 4.4201 | 4.7645 | 4.5022 | 4.597 | 4.9121 | 4.8409 | 4.6752 | 4.3947 | 4.7886 | 4.3429 | 4.9773 | 5.0629 | 4.7327 | 4.7121 | 4.734 |
| pos_4273 | Dihydroconiferyl alcohol | metab_3397 | HMDB0303757 | C10448 | 365.1943 | 6.125 | pos | 2M+H | C10H14O3 | 0 | 96.8 | -4.3459501 | 2305-13-7 | 0.0028141 | 4.4111 | 4.6236 | 4.5589 | 4.4223 | 4.5998 | 4.5328 | 4.6744 | 4.356 | 4.7004 | 4.4363 | 4.6669 | 4.5768 | 4.8481 | 4.8457 | 4.8474 |
| pos_4275 | Sterebin A | metab_3399 | HMDB0035337 | - | 328.2489 | 6.125 | pos | M+NH4 | C18H30O4 | 0 | 34.5 | 2.12621162 | 107647-14-3 | 0.01184261 | 5.5067 | 4.7587 | 4.7209 | 4.5951 | 4.2635 | 4.4154 | 4.9964 | 4.8143 | 4.5793 | 5.0245 | 4.5241 | 4.6128 | 4.9833 | 4.9738 | 4.9753 |
| pos_4277 | Batatasin IV | metab_3401 | LMPK13090032;HMDB0032641 | C10247 | 277.1439 | 6.125 | pos | M+CH3OH+H | C15H16O3 | 0 | 37.5 | 2.10071296 | 60347-67-3 | 0.02136711 | 3.5176 | 4.201 | 3.6901 | 4.0254 | 3.4348 | 3.8503 | 3.9757 | 3.9118 | 3.9795 | 3.4496 | 3.7875 | 3.8896 | 4.1628 | 4.1456 | 4.1483 |
| pos_4279 | RHODAMINE 6G | metab_3403 | HMDB0257199 | C11177 | 443.234 | 6.117 | pos | M+H | C28H30N2O3 | 0 | 64.1 | 2.35786671 | 989-38-8 | 0.01533946 | 5.1607 | 4.434 | 4.4674 | 4.4655 | 4.593 | 4.8262 | 4.4564 | 4.4264 | 4.388 | 4.3285 | 4.8999 | 4.6663 | 5.0829 | 5.0712 | 5.0828 |
| pos_4283 | Decylamine Oxide | metab_3408 | - | - | 202.2172 | 6.117 | pos | M+H | C12H27NO | 56.2 | 0 | 3.08877876 | - | 0.0059975 | 4.7639 | 4.8314 | 4.7932 | 4.7794 | 4.7669 | 4.8281 | 4.8474 | 4.7262 | 4.8016 | 4.8017 | 4.7811 | 4.8786 | 4.9279 | 4.9329 | 4.9292 |
| pos_4287 | 9-Tetradecenoic acid | metab_3412 | HMDB0002000;HMDB0258881 | C08322 | 244.2278 | 6.109 | pos | M+NH4 | C14H26O2 | 0 | 59.9 | 3.13471353 | 544-64-9 | 0.01519343 | 4.3352 | 4.507 | 4.3691 | 4.349 | 4.4584 | 4.524 | 4.4742 | 4.4014 | 4.3803 | 4.4508 | 4.5856 | 4.4506 | 4.6158 | 4.6182 | 4.6057 |
| pos_4294 | Flumazenil | metab_3419 | HMDB0015336 | C07825 | 321.1352 | 6.101 | pos | M+NH4 | C15H14FN3O3 | 0 | 45.3 | -1.8687311 | 78755-81-4 | 0.01134239 | 3.9662 | 4.2506 | 3.8977 | 3.7484 | 4.2503 | 4.3308 | 4.3768 | 3.2758 | 4.0364 | 3.4639 | 4.3331 | 4.3303 | 4.479 | 4.4698 | 4.4714 |
| pos_4305 | (1S,2S,4S,5S)-2,4-Thujanediol 4-O-beta-D-Glucopyranoside | metab_3432 | HMDB0033644 | - | 297.1681 | 6.0844 | pos | M+H-2H2O | C16H28O7 | 0 | 42.6 | -4.5198869 | 240495-81-2 | 0.01084233 | 5.0318 | 5.0153 | 5.1005 | 5.0507 | 5.1583 | 5.2691 | 5.0871 | 5.035 | 5.3803 | 5.0171 | 5.2279 | 5.241 | 5.1022 | 5.1021 | 5.094 |
| pos_4308 | METALDEHYDE | metab_3435 | HMDB0254499 | C18744 | 221.075 | 6.0844 | pos | M+2Na-H | C8H16O4 | 0 | 31.9 | -6.0054582 | 108-62-3 | 0.00626285 | 5.0498 | 5.1484 | 5.1053 | 5.2156 | 5.1779 | 5.0505 | 5.0006 | 5.1222 | 5.0132 | 5.0753 | 5.2093 | 5.3772 | 5.0016 | 5.0068 | 5.0029 |
| pos_4309 | 1-Carboxycyclohexaneacetic Acid | metab_3436 | - | - | 169.0865 | 6.0844 | pos | M+H-H2O | C9H14O4 | 58.7 | 0 | 2.86147874 | - | 0.0076369 | 4.6409 | 4.0474 | 4.0602 | 4.6255 | 4.3349 | 4.4612 | 4.4943 | 4.2123 | 4.0926 | 3.9304 | 4.0872 | 4.2597 | 4.199 | 4.1986 | 4.193 |
| pos_432 | Fluvoxamino acid | metab_3447 | HMDB0060950 | - | 319.1295 | 5.2876 | pos | M+H-H2O, M+H | C14H17F3N2O3 | 0 | 70.5 | 9.71946027 | 88699-91-6 | 0.0055869 | 4.7497 | 4.1172 | 4.5219 | 4.53 | 2.9675 | 2.9843 | 4.529 | 4.6752 | 3.5521 | 4.6863 | 2.9811 | 3.9764 | 4.664 | 4.6688 | 4.6656 |
| pos_4324 | Cromakalim | metab_3451 | HMDB0250542 | C11819 | 287.1397 | 6.0684 | pos | M+H | C16H18N2O3 | 0 | 79.2 | 2.26868857 | 94470-67-4 | 0.01061692 | 4.7031 | 4.6461 | 4.7077 | 4.5698 | 4.7486 | 4.7167 | 4.7215 | 4.7308 | 4.7157 | 4.5321 | 4.68 | 4.8203 | 4.9128 | 4.9199 | 4.9215 |
| pos_4326 | Lauroyl diethanolamide | metab_3453 | HMDB0032358;LMFA08040058 | - | 288.2539 | 6.0684 | pos | M+H | C16H33NO3 | 0 | 39.9 | 1.99001478 | 120-40-1 | 0.03537463 | 4.5684 | 4.0484 | 3.9306 | 4.2891 | 3.6647 | 2.889 | 4.2502 | 4.0812 | 3.4082 | 4.6607 | 3.2588 | 3.7496 | 4.2289 | 4.2547 | 4.2568 |
| pos_4327 | Glucosyl (E)-2,6-Dimethyl-2,5-heptadienoate | metab_3454 | HMDB0035136 | - | 281.1366 | 6.0684 | pos | M+H-2H2O | C15H24O7 | 0 | 31.9 | -5.5519295 | 261949-43-3 | 0.01068505 | 4.4341 | 4.4835 | 4.4019 | 4.4388 | 4.4727 | 4.4027 | 4.52 | 4.4074 | 4.3632 | 4.4539 | 4.4044 | 4.4547 | 4.6176 | 4.6175 | 4.6095 |
| pos_4333 | 9,10-Epoxy-18-hydroxy-octadecanoic acid | metab_3461 | HMDB0302107 | C19620 | 332.2801 | 6.0604 | pos | M+NH4 | C18H34O4 | 0 | 47.3 | 1.91307147 | - | 0.00558435 | 4.2547 | 4.3962 | 4.0758 | 4.2824 | 3.6464 | 3.5934 | 4.2313 | 3.9679 | 4.1567 | 4.3588 | 4.1887 | 4.2107 | 4.7819 | 4.7867 | 4.7834 |
| pos_4334 | 3,7-Dimethylquercetin | metab_3462 | HMDB0029263 | C01265 | 331.082 | 6.0604 | pos | M+H | C17H14O7 | 0 | 95.5 | 2.18855715 | 2/2/68 | 0.02366386 | 4.5686 | 4.6129 | 4.4358 | 4.237 | 4.5655 | 4.1963 | 4.481 | 4.7957 | 4.6443 | 4.2951 | 3.651 | 4.0921 | 4.1727 | 4.1726 | 4.1903 |
| pos_4335 | Arbutin | metab_3463 | HMDB0029943;MJDBOTE0001645 | C06186 | 237.0764 | 6.0604 | pos | M+H-2H2O | C12H16O7 | 0 | 59.1 | 2.29550481 | 497-76-7 | 0.00652652 | 5.0559 | 5.0968 | 5.0942 | 5.1052 | 5.0413 | 5.1216 | 5.0787 | 5.0844 | 5.065 | 5.1079 | 5.0559 | 5.0465 | 5.0725 | 5.0722 | 5.0674 |
| pos_4336 | Tetrapeptide | metab_3464 | HMDB0258927 | C01960 | 585.2722 | 6.0524 | pos | M+H-2H2O | C27H40N8O9 | 0 | 40.8 | -9.3153169 | - | 0.01306576 | 5.6871 | 3.8671 | 5.1608 | 5.1101 | 4.365 | 4.3149 | 5.0162 | 5.49 | 4.5574 | 5.9536 | 3.9985 | 4.7672 | 5.5478 | 5.5475 | 5.5575 |
| pos_4338 | 10-Hydroxycarbazepine | metab_3466 | HMDB0060676;PW_C040604 | C07493 | 255.1136 | 6.0524 | pos | M+H | C15H14N2O2 | 0 | 47.8 | 3.02368069 | 29331-92-8 | 0.07683451 | 3.5696 | 4.4794 | 3.765 | 4.063 | 3.2793 | 3.1333 | 3.4889 | 3.8362 | 3.7443 | 3.5646 | 3.6155 | 3.4964 | 4.0394 | 4.0395 | 4.0961 |
| pos_4341 | Abscisic alcohol | metab_3470 | HMDB0039635 | C13456 | 283.1907 | 6.0445 | pos | M+CH3OH+H | C15H22O3 | 0 | 50.2 | 1.35895664 | 113472-20-1 | 0.01198338 | 4.2093 | 4.1746 | 4.0342 | 3.8984 | 4.1743 | 4.0434 | 4.3832 | 3.6406 | 4.0723 | 4.2438 | 4.2194 | 4.2539 | 4.4141 | 4.4044 | 4.406 |
| pos_4348 | Trinexapac-ethyl | metab_3477 | HMDB0259248 | C18541 | 235.0971 | 6.0385 | pos | M+H-H2O | C13H16O5 | 0 | 57.3 | 2.28605139 | 95266-40-3 | 0.0086326 | 3.5167 | 3.6954 | 2.9031 | 3.2365 | 3.4376 | 3.6467 | 3.2094 | 2.827 | 3.6422 | 3.2404 | 3.2698 | 3.6047 | 4.8414 | 4.8357 | 4.8428 |
| pos_4356 | Cortisol | metab_3485 | HMDB0000063;LMST02030001;PW_C000045;MJDBOTE0000771 | C00735 | 363.2172 | 6.0289 | pos | M+H | C21H30O5 | 75.7 | 0 | 1.56861277 | 50-23-7 | 0.01577389 | 3.9669 | 5.0972 | 4.8806 | 5.227 | 4.3122 | 4.0266 | 4.3171 | 4.9314 | 5.0239 | 4.7781 | 4.3671 | 4.2145 | 5.0998 | 5.0996 | 5.0878 |
| pos_4383 | N-Acetyl-L-phenylalanine | metab_3515 | HMDB0000512 | C03519 | 190.0868 | 5.9981 | pos | M+H-H2O | C11H13NO3 | 0 | 45.1 | 2.56891181 | 2018-61-3 | 0.03344628 | 3.847 | 3.926 | 4.1343 | 4.0048 | 3.7891 | 3.6888 | 3.9506 | 4.0968 | 3.9271 | 3.3925 | 3.9991 | 4.215 | 3.9547 | 3.9803 | 3.9561 |
| pos_4385 | N-(3-Methyl-2-pyridyl)-3-phenylsuccinimide | metab_3517 | HMDB0253391 | - | 299.1396 | 5.9911 | pos | M+CH3OH+H | C16H14N2O2 | 0 | 49.6 | 2.30188238 | - | 0.0020249 | 5.4056 | 3.9788 | 5.0132 | 4.8315 | 4.1872 | 4.5054 | 5.2384 | 4.8813 | 4.0872 | 5.2896 | 3.9592 | 4.4764 | 5.2051 | 5.205 | 5.2035 |
| pos_4392 | Fuberidazole | metab_3525 | - | C18737 | 185.0715 | 5.9831 | pos | M+H | C11H8N2O | 53.7 | 0 | 3.26014495 | 3878-19-1 | 0.00861136 | 4.1103 | 3.7701 | 3.7754 | 3.7743 | 3.9967 | 4.1335 | 4.1115 | 3.7044 | 3.8445 | 3.6329 | 4.012 | 3.9229 | 4.3163 | 4.3161 | 4.3097 |
| pos_4396 | L-Octanoylcarnitine | metab_3529 | HMDB0000791;LMFA07070002 | C02838 | 288.2175 | 5.9766 | pos | M+H | C15H29NO4 | 84.1 | 0 | 2.00957654 | 25243-95-2 | 0.0112318 | 5.4894 | 4.8461 | 4.7624 | 4.6702 | 4.4144 | 4.5941 | 5.0679 | 4.9445 | 4.495 | 5.0052 | 4.4646 | 4.5587 | 4.9661 | 4.9659 | 4.9744 |
| pos_4401 | Indolophenanthridine | metab_3536 | HMDB0253475 | - | 292.1816 | 5.9716 | pos | M+NH4 | C19H18N2 | 0 | 37.9 | 2.67338273 | - | 0.03595266 | 4.9934 | 4.1594 | 4.4743 | 4.6725 | 4.0727 | 4.5012 | 3.4508 | 3.0347 | 4.2714 | 4.2637 | 4.4461 | 3.7327 | 4.8216 | 4.8219 | 4.8485 |
| pos_4406 | Vomifoliol | metab_3540 | HMDB0303570 | C04166 | 242.1757 | 5.96 | pos | M+NH4 | C13H20O3 | 0 | 62.9 | 2.71362341 | - | 0.00222644 | 5.0656 | 5.0427 | 5.098 | 5.1074 | 5.0814 | 5.121 | 5.046 | 5.1032 | 5.082 | 5.1106 | 5.0649 | 5.0482 | 5.1052 | 5.1071 | 5.1066 |
| pos_4412 | Menadione bisulfite | metab_3546 | HMDB0254432;HMDB0001892 | C05377 | 277.0123 | 5.9408 | pos | M+Na | C11H10O5S | 0 | 53.1 | -7.1814412 | 58-27-5 | 0.03176412 | 3.6101 | 4.0362 | 3.5446 | 4.0458 | 4.4686 | 4.5069 | 3.8317 | 3.3664 | 4.6926 | 3.7257 | 4.371 | 4.2608 | 4.2992 | 4.2746 | 4.2765 |
| pos_4413 | Isopentyl beta-D-glucoside | metab_3547 | HMDB0034750 | - | 215.1284 | 5.9408 | pos | M+H-2H2O | C11H22O6 | 0 | 49.5 | 2.4555811 | - | 0.00209936 | 4.1612 | 4.0857 | 4.1525 | 4.1072 | 4.1306 | 4.1533 | 4.0367 | 4.1811 | 4.1519 | 4.1852 | 4.1299 | 4.0511 | 5.039 | 5.0388 | 5.0373 |
| pos_4415 | Embelin | metab_3549 | HMDB0251767;LMPK15050006 | C10342 | 333.145 | 5.9317 | pos | M+K | C17H26O4 | 0 | 42.4 | -4.1508254 | 550-24-3 | 0.01414786 | 5.3753 | 4.9036 | 5.5028 | 5.4112 | 4.9158 | 5.2004 | 5.5338 | 5.5021 | 5.0943 | 5.4326 | 5.0594 | 5.3091 | 5.5637 | 5.5757 | 5.5676 |
| pos_4431 | 5-Nitro-2-(3-phenylpropylamino)benzoic acid | metab_3567 | HMDB0255766 | C13705 | 301.1189 | 5.8812 | pos | M+H | C16H16N2O4 | 0 | 43 | 2.10996883 | 107254-86-4 | 0.01782071 | 2.7924 | 4.6494 | 4.0032 | 4.4512 | 2.7474 | 2.7642 | 3.4918 | 3.9668 | 3.1211 | 3.2971 | 3.3235 | 2.7559 | 4.3143 | 4.3051 | 4.3206 |
| pos_4436 | Ampicillin | metab_3572 | HMDB0014559;MJDBOTE0001553 | C06574 | 367.1408 | 5.8528 | pos | M+NH4 | C16H19N3O4S | 0 | 72.9 | -7.5838235 | 69-53-4 | 0.0243366 | 4.7278 | 3.9935 | 4.7155 | 4.0741 | 4.0883 | 4.7203 | 5.2008 | 4.0015 | 4.0019 | 4.501 | 4.5454 | 4.4466 | 4.7585 | 4.7408 | 4.76 |
| pos_4452 | Ethyl 3-hydroxydodecanoate | metab_3589 | HMDB0059850 | - | 262.2383 | 5.8083 | pos | M+NH4 | C14H28O3 | 0 | 68.5 | 2.41629525 | - | 0.0154554 | 3.9477 | 4.1308 | 4.1175 | 3.9888 | 3.9798 | 4.0973 | 4.0894 | 3.9365 | 3.9591 | 4.0457 | 4.0443 | 3.9282 | 5.1983 | 5.1873 | 5.1995 |
| pos_4453 | Quinmerac | metab_3590 | HMDB0257043 | C18891 | 254.0584 | 5.8038 | pos | M+CH3OH+H | C11H8ClNO2 | 0 | 37 | 2.56457085 | 90717-03-6 | 0.00540104 | 4.4798 | 4.4574 | 4.2092 | 4.491 | 4.2386 | 4.4533 | 4.43 | 4.2474 | 4.269 | 4.1401 | 4.4184 | 4.9349 | 4.5358 | 4.5404 | 4.5373 |
| pos_4458 | Nafenopin | metab_3594 | HMDB0255421 | C11371 | 333.1451 | 5.7821 | pos | M+Na | C20H22O3 | 0 | 37 | -3.2760781 | 3771-19-5 | 0.01589096 | 5.213 | 4.9245 | 5.3102 | 5.2247 | 4.9163 | 5.2005 | 5.44 | 5.1613 | 4.8352 | 5.3194 | 5.0859 | 5.3207 | 5.4269 | 5.4157 | 5.4284 |
| pos_4461 | N-Phenethylacetamide | metab_3598 | HMDB0255222 | C06746 | 164.1075 | 5.7654 | pos | M+H | C10H13NO | 39.5 | 0 | 3.27052913 | - | 0.00807846 | 4.642 | 4.6224 | 4.5964 | 4.6377 | 4.5821 | 4.6573 | 4.6536 | 4.6553 | 4.6398 | 4.7122 | 4.5893 | 4.5982 | 4.6611 | 4.6609 | 4.6549 |
| pos_4467 | 5-Hydroxyindoleacetylglycine | metab_3603 | HMDB0004185 | C05832 | 231.0771 | 5.7378 | pos | M+H-H2O | C12H12N2O4 | 0 | 82.5 | 2.73240041 | - | 0.02363324 | 4.9754 | 5.3821 | 5.0633 | 5.021 | 5.7011 | 5.744 | 5.0823 | 5.1499 | 5.659 | 4.7071 | 5.6973 | 5.8896 | 5.2451 | 5.2268 | 5.2282 |
| pos_4472 | (+/-)-6-Chlorotryptophan | metab_3608 | HMDB0033557 | - | 239.0588 | 5.7109 | pos | M+H | C11H11ClN2O2 | 0 | 59.2 | 2.70333587 | 17808-21-8 | 0.02028917 | 3.6697 | 4.8318 | 4.4972 | 4.7333 | 4.1344 | 4.3373 | 4.1266 | 3.9385 | 3.9258 | 3.2663 | 4.6251 | 4.0259 | 4.457 | 4.4425 | 4.4585 |
| pos_4488 | N-Chlorophenylalanine | metab_3623 | HMDB0255100 | - | 182.0373 | 5.6443 | pos | M+H-H2O | C9H10ClNO2 | 0 | 35.5 | 2.94800747 | - | 0.04020337 | 3.4957 | 4.5794 | 4.2391 | 4.5094 | 4.0108 | 4.2691 | 3.949 | 3.8266 | 3.9907 | 3.0078 | 4.4646 | 3.8978 | 4.1586 | 4.1281 | 4.1293 |
| pos_4491 | Glutathione episulfonium ion | metab_3627 | HMDB0060479 | C14874 | 367.1409 | 5.6357 | pos | M+CH3OH+H | C12H20N3O6S+ | 0 | 84.7 | 0.36386692 | - | 0.01583515 | 4.8792 | 4.2498 | 4.8295 | 4.1791 | 4.3298 | 4.8762 | 5.369 | 4.1755 | 4.1759 | 4.6633 | 4.6953 | 4.6502 | 4.9522 | 4.9521 | 4.964 |
| pos_4500 | N-Nervonoyl Asparagine | metab_3636 | HMDB0242086 | - | 241.2043 | 5.5816 | pos | M+2H | C28H52N2O4 | 0 | 47.5 | 2.58646168 | - | 0.01105846 | 5.2772 | 4.4182 | 3.613 | 3.6494 | 3.6076 | 5.1534 | 4.213 | 3.1725 | 3.9466 | 3.3189 | 3.506 | 4.8733 | 4.9962 | 4.996 | 4.9878 |
| pos_4501 | Indole-3-acetic Acid | metab_3637 | HMDB0302181;HMDB0000197;MJDBZSJ0000111 | C00954 | 176.0712 | 5.5771 | pos | M+H | C10H9NO2 | 91.8 | 0 | 3.42929848 | 87-51-4 | 0.02168523 | 4.1407 | 4.3223 | 4.0582 | 4.2834 | 4.0389 | 4.4862 | 4.083 | 3.988 | 4.0841 | 3.9706 | 4.293 | 4.3067 | 4.1047 | 4.0878 | 4.0893 |
| pos_4505 | Harmalol | metab_3641 | HMDB0029838 | C06537 | 201.1029 | 5.5473 | pos | M+H | C12H12N2O | 0 | 61.9 | 3.38200548 | 525-57-5 | 0.01011957 | 3.7444 | 3.8115 | 3.8832 | 3.764 | 4.5368 | 3.9915 | 3.8141 | 3.9212 | 3.9338 | 3.8621 | 4.195 | 4.1948 | 4.5862 | 4.5857 | 4.5783 |
| pos_4508 | N-Carbamimidoyliminomethanethioamide | metab_3644 | HMDB0260382 | - | 158.0494 | 5.5372 | pos | M+ACN+H | C2H4N4S | 0 | 31.2 | -1.001822 | - | 0.03378351 | 3.8519 | 3.8987 | 3.9845 | 3.8429 | 3.8791 | 3.9457 | 4.0051 | 4.0486 | 3.9314 | 4.0079 | 3.9092 | 3.8805 | 3.9796 | 3.9506 | 3.9698 |
| pos_453 | A-L-Arabinofuranosyl-(1->2)-[a-D-mannopyranosyl-(1->6)]-D-mannose | metab_3665 | HMDB0039818 | - | 492.1877 | 5.5771 | pos | M+NH4, M+Na | C17H30O15 | 0 | 59.8 | -9.6367416 | - | 0.00787563 | 4.5072 | 5.1811 | 4.5902 | 4.4817 | 5.1616 | 5.1863 | 4.863 | 4.6072 | 5.0177 | 3.9296 | 4.9378 | 5.0411 | 5.1707 | 5.1641 | 5.1658 |
| pos_454 | (+/-)-Enterolactone | metab_3676 | HMDB0006101 | C18165 | 299.1285 | 5.5711 | pos | M+H-H2O, M+H | C18H18O4 | 52.6 | 0 | 2.30368614 | 185254-87-9 | 0.00492856 | 4.1542 | 4.8977 | 4.3403 | 4.1426 | 4.8386 | 4.8844 | 4.6191 | 4.3098 | 4.7151 | 3.3286 | 4.6385 | 4.6946 | 4.8101 | 4.807 | 4.8111 |
| pos_4559 | Lumichrome | metab_3696 | HMDB0254199 | C01727 | 243.0883 | 5.4189 | pos | M+H | C12H10N4O2 | 62.5 | 0 | 2.70612647 | 1086-80-2 | 0.01838837 | 3.9603 | 3.9045 | 3.8268 | 3.9874 | 4.0745 | 3.9448 | 4.3814 | 4.0336 | 4.0099 | 4.2714 | 3.9006 | 4.128 | 4.1546 | 4.1543 | 4.1682 |
| pos_4568 | L-beta-aspartyl-L-serine | metab_3706 | HMDB0011168 | - | 504.1583 | 5.4053 | pos | 2M+ACN+Na | C7H12N2O6 | 0 | 56.6 | 7.92788416 | - | 0.05383406 | 5.0692 | 4.8983 | 4.6367 | 4.533 | 4.8238 | 4.6644 | 4.7709 | 5.0428 | 4.9061 | 4.4688 | 4.7494 | 4.6961 | 4.4399 | 4.3989 | 4.4403 |
| pos_4577 | Heptyl 4-hydroxybenzoate | metab_3716 | HMDB0034462 | C14718 | 219.1385 | 5.3896 | pos | M+H-H2O | C14H20O3 | 0 | 58.5 | 2.3614943 | 1085-12-7 | 0.00562509 | 5.0862 | 5.0673 | 5.0567 | 5.0862 | 5.0419 | 5.1137 | 5.0542 | 5.1382 | 5.1357 | 5.1666 | 5.0977 | 5.0436 | 5.204 | 5.1992 | 5.2009 |
| pos_4589 | 1-(2-Furyl)butan-3-one | metab_3728 | HMDB0032290 | - | 103.055 | 5.3649 | pos | M+H-2H2O | C8H10O2 | 0 | 41.2 | 5.40676777 | 699-17-2 | 0.0094837 | 4.7161 | 4.5673 | 4.5542 | 4.5466 | 4.4801 | 4.4543 | 4.5375 | 4.4191 | 4.7646 | 4.551 | 4.3793 | 4.4836 | 4.5207 | 4.5129 | 4.5144 |
| pos_4601 | Nonate | metab_3742 | HMDB0011717 | - | 153.0915 | 5.3368 | pos | M+H-2H2O | C9H16O4 | 0 | 38.1 | 2.82671831 | - | 0.01085468 | 4.3127 | 4.5541 | 4.2532 | 4.3302 | 4.3587 | 4.4873 | 4.4122 | 4.3244 | 4.534 | 4.2925 | 4.4901 | 4.4744 | 4.5009 | 4.4936 | 4.5024 |
| pos_4614 | Alpha-Ionone | metab_3756 | HMDB0059883 | C12286 | 175.1487 | 5.2931 | pos | M+H-H2O | C13H20O | 0 | 52.6 | 2.91188959 | 127-41-3 | 0.00729138 | 2.723 | 3.6406 | 3.0624 | 2.657 | 4.1868 | 4.322 | 3.1331 | 2.9592 | 4.322 | 2.6566 | 4.2712 | 4.2172 | 4.584 | 4.579 | 4.5848 |
| pos_4617 | 1,5-Isoquinolinediol | metab_3759 | HMDB0244237 | - | 162.0554 | 5.2876 | pos | M+H | C9H7NO2 | 0 | 50.9 | 2.88058494 | - | 0.03042488 | 3.3447 | 3.05 | 3.3787 | 2.7862 | 4.5768 | 4.5728 | 3.2877 | 3.5093 | 4.5595 | 2.7859 | 4.5287 | 4.4489 | 4.384 | 4.3606 | 4.3622 |
| pos_4622 | N-Nitrosomethylethylamine | metab_3764 | - | C19281 | 89.0718 | 5.2736 | pos | M+H | C3H8N2O | 39.7 | 0 | 9.97137748 | 10595-95-6 | 0.01095602 | 4.3324 | 4.2889 | 4.255 | 4.3352 | 4.2549 | 4.3326 | 4.282 | 4.3367 | 4.3673 | 4.3925 | 4.2389 | 4.2227 | 4.3941 | 4.4014 | 4.4031 |
| pos_4674 | Risdiplam | metab_3811 | HMDB0304853 | - | 419.2296 | 5.1185 | pos | M+NH4 | C22H23N7O | 0 | 49.4 | -1.5025252 | - | 0.03063594 | 3.8402 | 4.2346 | 3.4541 | 3.9784 | 3.9519 | 3.7261 | 4.0722 | 3.3463 | 3.9516 | 3.339 | 3.8635 | 3.8628 | 4.7303 | 4.754 | 4.732 |
| pos_4675 | Kainic acid | metab_3812 | HMDB0253757 | C12819 | 178.0868 | 5.1185 | pos | M+H-2H2O | C10H15NO4 | 0 | 72.3 | 2.75076768 | 487-79-6 | 0.02117122 | 3.2255 | 3.4226 | 3.1816 | 3.1202 | 4.0565 | 3.6327 | 3.1594 | 3.5084 | 4.1146 | 3.3284 | 4.0095 | 4.0535 | 4.2221 | 4.207 | 4.2238 |
| pos_4681 | Phenylpropionylglycine | metab_3819 | HMDB0000860 | - | 208.0974 | 5.0939 | pos | M+H | C11H13NO3 | 0 | 32.2 | 3.00109504 | 56613-60-6 | 0.01350962 | 4.4194 | 4.6579 | 4.5047 | 4.5345 | 4.4441 | 4.5776 | 4.5253 | 4.4189 | 4.5842 | 4.417 | 4.49 | 4.6759 | 4.6438 | 4.6436 | 4.6335 |
| pos_4683 | Cholylmethionine | metab_3821 | HMDB0242382 | - | 553.3395 | 5.0864 | pos | 2M+3H2O+2H | C29H49NO6S | 0 | 35 | -0.1914828 | - | 0.00678779 | 6.3113 | 6.3694 | 6.4625 | 6.3621 | 6.4717 | 6.3653 | 6.3827 | 6.3606 | 6.3005 | 6.284 | 6.4349 | 6.432 | 6.1128 | 6.118 | 6.113 |
| pos_4700 | Deoxycholylarginine | metab_3840 | HMDB0242418 | - | 566.4291 | 5.0353 | pos | M+NH4 | C30H52N4O5 | 0 | 49.4 | 2.68019584 | - | 0.01258727 | 5.399 | 5.3945 | 5.361 | 5.3541 | 5.2826 | 5.3774 | 5.4003 | 5.3548 | 5.4381 | 5.4388 | 5.3147 | 5.2977 | 5.3994 | 5.3992 | 5.4087 |
| pos_4713 | PGP(a-25:0/22:5(4Z,7Z,10Z,13Z,19Z)-O(16,17)) | metab_3854 | HMDB0274767 | - | 509.3132 | 4.9946 | pos | M+2H | C53H94O14P2 | 0 | 51.3 | 0.026604 | - | 0.00443192 | 6.7357 | 6.803 | 6.8554 | 6.823 | 6.9126 | 6.8094 | 6.8498 | 6.79 | 6.7867 | 6.7176 | 6.8312 | 6.8787 | 6.5766 | 6.5793 | 6.5756 |
| pos_4719 | 5-Phenyl-1,3-oxazinane-2,4-dione | metab_3860 | HMDB0060400;PW_C040533 | C16596 | 233.0927 | 4.9866 | pos | M+ACN+H | C10H9NO3 | 0 | 47.9 | 3.17606575 | - | 0.00316082 | 4.1633 | 5.2985 | 4.7443 | 4.9271 | 4.5574 | 4.8496 | 4.9505 | 4.0929 | 3.9946 | 3.5869 | 5.0111 | 4.4979 | 4.7961 | 4.7989 | 4.7975 |
| pos_4722 | (+/-)-Tryptophan | metab_3864 | HMDB0030396 | C00806 | 187.0872 | 4.9786 | pos | M+H-H2O | C11H12N2O2 | 0 | 61.4 | 2.88474477 | 54-12-6 | 0.02165883 | 3.1415 | 4.5274 | 3.9716 | 4.1824 | 3.6726 | 4.0353 | 4.1344 | 3.1216 | 2.7573 | 2.5594 | 4.2327 | 3.5694 | 3.9751 | 3.9581 | 3.9598 |
| pos_4723 | Agavoside A | metab_3865 | LMST01080006;HMDB0034391 | C08885 | 606.3715 | 4.9706 | pos | 2M+3H2O+2H | C33H52O9 | 0 | 45.9 | -2.0814023 | 56857-65-9 | 0.00831093 | 7.2524 | 7.3427 | 7.3953 | 7.3795 | 7.4075 | 7.3476 | 7.4155 | 7.3152 | 7.2857 | 7.2419 | 7.3779 | 7.4204 | 7.1938 | 7.1991 | 7.2007 |
| pos_4738 | SM(d20:1/TXB2) | metab_3880 | HMDB0290731 | - | 445.2893 | 4.9145 | pos | M+2Na | C45H85N2O10P | 0 | 37.8 | 6.95931094 | - | 0.00690292 | 6.518 | 6.5657 | 6.6287 | 6.6338 | 6.6855 | 6.5755 | 6.6388 | 6.5863 | 6.5233 | 6.483 | 6.6428 | 6.6478 | 6.4641 | 6.4639 | 6.4692 |
| pos_4741 | Permetin A | metab_3884 | HMDB0030527 | - | 562.3451 | 4.8905 | pos | M+H+Na | C54H92N12O12 | 0 | 95.6 | -1.8929902 | 71888-70-5 | 0.00644055 | 6.9345 | 7.0031 | 7.0499 | 7.0605 | 7.0927 | 6.9967 | 7.0798 | 6.985 | 6.9355 | 6.9221 | 7.0621 | 7.1006 | 6.8978 | 6.9016 | 6.9032 |
| pos_4747 | PGP(a-25:0/22:6(5Z,7Z,10Z,13Z,16Z,19Z)-OH(4)) | metab_3890 | HMDB0274757 | - | 509.3133 | 4.8665 | pos | M+2H | C53H94O14P2 | 0 | 55.1 | 0.16464958 | - | 0.01153026 | 5.8953 | 5.9468 | 5.9975 | 5.9714 | 6.0391 | 5.9266 | 5.9969 | 5.9466 | 5.8768 | 5.8825 | 6.0267 | 6.056 | 5.6737 | 5.683 | 5.6752 |
| pos_4749 | PG(TXB2/i-21:0) | metab_3892 | HMDB0272022 | - | 465.2869 | 4.8665 | pos | M+H+Na | C47H87O14P | 0 | 61.1 | -6.7644115 | - | 0.0063975 | 7.0886 | 7.1435 | 7.1979 | 7.1878 | 7.2511 | 7.1341 | 7.2046 | 7.1372 | 7.0872 | 7.0705 | 7.2076 | 7.242 | 6.929 | 6.9287 | 6.9337 |
| pos_4775 | SM(d16:1/22:6(5Z,8E,10Z,13Z,15E,19Z)-2OH(7S, 17S)) | metab_3921 | HMDB0290284 | - | 401.263 | 4.7714 | pos | M+H+Na | C43H75N2O8P | 0 | 51.5 | 4.34455573 | - | 0.0113664 | 6.7627 | 6.787 | 6.8553 | 6.8357 | 6.9277 | 6.811 | 6.8566 | 6.7938 | 6.766 | 6.7444 | 6.8573 | 6.8779 | 6.7308 | 6.7384 | 6.74 |
| pos_4780 | Hydroxy Tyrosol -Acetate | metab_3926 | HMDB0253254 | - | 208.0974 | 4.7554 | pos | M+H-H2O | C11H15NO4 | 0 | 42.4 | 2.74634993 | - | 0.05358945 | 4.1068 | 4.2894 | 4.4408 | 4.3847 | 4.0143 | 4.187 | 4.156 | 4.6663 | 4.249 | 3.8849 | 4.4589 | 4.1913 | 4.2283 | 4.2281 | 4.2679 |
| pos_4787 | Sulfoquinovosyl diglyceride | metab_3933 | HMDB0258600 | - | 421.2605 | 4.7234 | pos | M+2H | C45H76O12S | 0 | 49.1 | 0.78225414 | - | 0.00380227 | 7.2122 | 7.2374 | 7.3237 | 7.2884 | 7.3677 | 7.2425 | 7.3225 | 7.2755 | 7.2126 | 7.1892 | 7.3276 | 7.3579 | 7.0751 | 7.0768 | 7.0784 |
| pos_4789 | Ile Trp | metab_3935 | - | - | 318.182 | 4.7234 | pos | M+H | C17H23N3O3 | 83.7 | 0 | 2.48302616 | - | 0.01496539 | 4.7468 | 5.1078 | 4.7855 | 5.2729 | 4.5367 | 4.3636 | 4.5488 | 4.2745 | 4.6149 | 4.8723 | 4.8145 | 4.8627 | 4.8961 | 4.908 | 4.8977 |
| pos_4807 | 4-Piperidinone, 1-hydroxy-2,2,6,6-tetramethyl- | metab_3954 | - | - | 172.1337 | 4.6514 | pos | M+H | C9H17NO2 | 34.2 | 0 | 2.87509352 | - | 0.00216324 | 4.8872 | 4.8812 | 4.9131 | 4.9007 | 4.834 | 4.9375 | 4.8946 | 4.9304 | 4.9307 | 4.965 | 4.8726 | 4.8645 | 4.9683 | 4.9667 | 4.9683 |
| pos_4816 | Cyclopamine | metab_3963 | HMDB0250664 | C10798 | 453.3446 | 4.6274 | pos | M+ACN+H | C27H41NO2 | 0 | 77.4 | -7.2254181 | 4449-51-8 | 0.01349396 | 5.318 | 5.2969 | 5.3039 | 5.368 | 5.2817 | 5.3589 | 5.3321 | 5.3167 | 5.3367 | 5.36 | 5.2687 | 5.3071 | 5.3383 | 5.3476 | 5.3492 |
| pos_4825 | L-Hexanoylcarnitine | metab_3973 | HMDB0000756 | - | 260.1863 | 4.6034 | pos | M+H | C13H25NO4 | 83.3 | 0 | 2.5708109 | 22671-29-0 | 0.00450775 | 5.0426 | 4.6455 | 4.5714 | 4.5141 | 4.3055 | 4.4179 | 4.8827 | 4.7827 | 4.2961 | 4.6798 | 4.3033 | 4.4926 | 4.6253 | 4.6214 | 4.623 |
| pos_4833 | Metribuzin | metab_3982 | HMDB0254685 | C14332 | 237.0777 | 4.5714 | pos | M+Na | C8H14N4OS | 0 | 49.5 | -1.5571065 | 21087-64-9 | 0.00311225 | 4.6547 | 5.7036 | 5.2919 | 5.0519 | 5.2902 | 5.862 | 5.6386 | 3.5737 | 3.574 | 3.5769 | 5.9381 | 5.3051 | 5.1901 | 5.1874 | 5.189 |
| pos_4846 | Indole-3-Carbinol | metab_3996 | HMDB0005785 | - | 130.0657 | 4.5234 | pos | M+H-H2O | C9H9NO | 39 | 0 | 3.77895979 | 700-06-1 | 0.00431932 | 4.7767 | 5.0375 | 4.7829 | 4.9154 | 4.7276 | 5.1932 | 4.8863 | 4.63 | 4.5183 | 4.4503 | 4.7094 | 4.9548 | 5.0359 | 5.0336 | 5.0373 |
| pos_4849 | N,N-Didemethyl orphenadrine | metab_3999 | HMDB0061165 | - | 242.1546 | 4.5154 | pos | M+H | C16H19NO | 0 | 48.6 | 2.64784801 | - | 0.01685038 | 3.2741 | 3.3447 | 3.3422 | 3.1599 | 5.8895 | 5.5907 | 3.19 | 3.2696 | 5.8396 | 3.0032 | 5.6022 | 5.835 | 5.574 | 5.562 | 5.5754 |
| pos_4857 | Indole-3-carboxylic acid | metab_4008 | HMDB0003320 | C19837 | 162.0555 | 4.4994 | pos | M+H | C9H7NO2 | 0 | 49.6 | 3.09505247 | 771-50-6 | 0.01541257 | 3.1177 | 3.1999 | 3.3725 | 3.2088 | 4.2816 | 4.5095 | 3.3036 | 3.5057 | 4.4154 | 3.1028 | 4.4104 | 4.6282 | 4.5924 | 4.6054 | 4.6018 |
| pos_4860 | Tropolone | metab_4012 | HMDB0259299 | C15474 | 105.0343 | 4.4914 | pos | M+H-H2O | C7H6O2 | 0 | 47.9 | 6.44567906 | 533-75-5 | 0.01939416 | 3.6321 | 3.7796 | 3.6096 | 3.687 | 3.8052 | 3.8983 | 3.6264 | 3.5996 | 3.9546 | 3.4781 | 3.9296 | 4.0351 | 3.7115 | 3.6999 | 3.7164 |
| pos_4863 | 6-Hydroxyheptanoylcarnitine | metab_4015 | HMDB0241680 | - | 272.1862 | 4.4754 | pos | M+H-H2O | C14H27NO5 | 0 | 47.4 | 2.02284899 | - | 0.0099111 | 3.6478 | 3.7225 | 2.9986 | 2.7889 | 3.8324 | 3.6222 | 3.7668 | 2.7853 | 3.573 | 3.1214 | 3.7928 | 3.7171 | 5.0296 | 5.0362 | 5.0377 |
| pos_4866 | VASICINONE | metab_4018 | HMDB0259770 | C10744 | 244.1087 | 4.4529 | pos | M+ACN+H | C11H10N2O2 | 0 | 39.3 | 3.17434865 | 486-64-6 | 0.02503842 | 2.796 | 4.8501 | 4.1846 | 4.5138 | 3.5047 | 3.8535 | 4.0546 | 3.4004 | 2.8183 | 2.8212 | 4.2462 | 3.5641 | 4.1378 | 4.1574 | 4.1561 |
| pos_4867 | 5-Methoxyindoleacetate | metab_4019 | HMDB0004096;PW_C002060 | C05660 | 188.0713 | 4.4529 | pos | M+H-H2O | C11H11NO3 | 0 | 68.2 | 3.38382142 | 3471-31-6 | 0.01162755 | 2.467 | 4.5694 | 3.9884 | 4.2159 | 3.4587 | 3.7735 | 3.9719 | 3.3029 | 2.5629 | 1.8686 | 4.0774 | 3.3689 | 3.8967 | 3.8962 | 3.8877 |
| pos_4893 | PA(10:0/PGF1alpha) | metab_4047 | HMDB0262739 | - | 333.2078 | 4.3362 | pos | M+2H | C33H61O11P | 0 | 40.8 | 8.80064609 | - | 0.00489722 | 6.1846 | 6.219 | 6.2522 | 6.2272 | 6.3302 | 6.2056 | 6.2562 | 6.1805 | 6.1839 | 6.1531 | 6.2637 | 6.3006 | 6.08 | 6.0757 | 6.0774 |
| pos_4894 | Dehydrocrotonin | metab_4048 | HMDB0250947 | - | 279.1381 | 4.3362 | pos | M+H-2H2O | C19H22O4 | 0 | 54.2 | 0.40892522 | - | 0.01220351 | 6.0764 | 6.1615 | 6.13 | 6.2149 | 5.9943 | 6.0088 | 6.1899 | 6.1886 | 6.1431 | 6.0744 | 6.1754 | 6.1397 | 6.1043 | 6.1041 | 6.1133 |
| pos_4895 | Leucylproline | metab_4049 | HMDB0011175 | - | 211.1447 | 4.3362 | pos | M+H-H2O | C11H20N2O3 | 0 | 59 | 2.65245651 | 6403-35-6 | 0.0137688 | 3.7203 | 3.9608 | 3.9764 | 3.306 | 4.1845 | 4.2895 | 4.0493 | 4.0294 | 4.3698 | 3.9632 | 4.3464 | 4.3269 | 4.1601 | 4.1489 | 4.1509 |
| pos_4908 | Butyl 4-aminobenzoate | metab_4064 | HMDB0249473 | C07875 | 158.097 | 4.2827 | pos | M+H-2H2O | C11H15NO2 | 0 | 43.2 | 2.82250195 | - | 0.003057 | 4.523 | 4.5456 | 4.5067 | 4.5034 | 4.5506 | 4.5554 | 4.5313 | 4.4614 | 4.5275 | 4.5104 | 4.5299 | 4.4949 | 4.816 | 4.8159 | 4.8137 |
| pos_4909 | Arg-Thr-Lys-Arg | metab_4065 | HMDB0304796 | - | 592.3922 | 4.2757 | pos | M+CH3OH+H | C22H45N11O6 | 0 | 42.8 | 5.78711598 | - | 0.01284636 | 5.5473 | 5.5151 | 5.5695 | 5.4477 | 5.675 | 5.5427 | 5.602 | 5.4619 | 5.5364 | 5.4927 | 5.5928 | 5.6381 | 5.6441 | 5.6338 | 5.6354 |
| pos_4916 | Gluten exorphin C | metab_4073 | HMDB0059796 | - | 632.3873 | 4.2166 | pos | M+H-2H2O | C29H57N5O12 | 0 | 69.2 | 1.10250684 | - | 0.0119352 | 5.9599 | 5.9403 | 6.0148 | 5.9408 | 6.1133 | 5.9581 | 6.0191 | 5.981 | 5.9607 | 5.9511 | 5.9987 | 6.0883 | 5.8802 | 5.88 | 5.8711 |
| pos_4925 | DL-p-Chlorophenylalanine methyl ester hydrochloride | metab_4083 | - | - | 214.0635 | 4.1544 | pos | M+H | C10H12ClNO2 | 91.3 | 0 | 2.85708145 | - | 0.01269453 | 4.5204 | 4.6316 | 4.5856 | 4.5069 | 4.5005 | 4.5747 | 4.497 | 4.5415 | 4.5944 | 4.5363 | 4.5672 | 4.4631 | 4.6304 | 4.6201 | 4.6218 |
| pos_4927 | 4-Hydroxy-alprenolol | metab_4085 | HMDB0061122 | - | 548.3657 | 4.1384 | pos | 2M+NH4 | C15H23NO3 | 0 | 55.2 | -6.9847408 | - | 0.01051512 | 5.5744 | 5.5791 | 5.667 | 5.6045 | 5.6808 | 5.631 | 5.5902 | 5.5679 | 5.5633 | 5.4816 | 5.6269 | 5.6826 | 5.7258 | 5.7186 | 5.727 |
| pos_4941 | Anamorelin | metab_4099 | HMDB0248383 | - | 588.3608 | 4.0797 | pos | M+ACN+H | C31H42N6O3 | 0 | 63.9 | -8.8585099 | - | 0.00454581 | 5.9896 | 6.0054 | 6.0619 | 6.0005 | 6.118 | 6.0206 | 6.0174 | 5.9992 | 5.9926 | 5.9371 | 6.0408 | 6.0732 | 5.9199 | 5.9222 | 5.9239 |
| pos_4961 | Phenylalanylhistidine | metab_4121 | HMDB0028997;HMDB0028892 | - | 285.1353 | 3.969 | pos | M+H-H2O | C15H18N4O3 | 0 | 36.5 | 2.35255734 | 16874-81-0;33367-37-2 | 0.01414907 | 4.3682 | 4.9199 | 4.7704 | 4.1717 | 4.5602 | 4.9906 | 5.1323 | 2.7792 | 2.7796 | 3.9945 | 5.0678 | 4.5245 | 4.7601 | 4.7699 | 4.7714 |
| pos_4962 | N-[(3a,5b,7a)-3-hydroxy-24-oxo-7-(sulfooxy)cholan-24-yl]-Glycine | metab_4122 | HMDB0002496 | C01324 | 530.2823 | 3.961 | pos | M+H | C26H43NO8S | 0 | 38.8 | 7.77187565 | 67030-55-1 | 0.01353262 | 3.0865 | 3.551 | 3.8537 | 3.6704 | 3.0906 | 3.4764 | 3.7064 | 3.4822 | 3.688 | 3.4766 | 3.7647 | 3.6453 | 4.5189 | 4.5185 | 4.5288 |
| pos_4966 | 6-(Pyridin-3-YL)hexanoic acid | metab_4126 | HMDB0245979 | - | 211.1447 | 3.945 | pos | M+NH4 | C11H15NO2 | 0 | 43.8 | 3.08354503 | - | 0.00330461 | 3.6754 | 3.994 | 3.8399 | 3.9824 | 4.0777 | 3.9989 | 4.098 | 3.5799 | 4.1738 | 3.8714 | 4.1146 | 4.116 | 4.6608 | 4.6579 | 4.6595 |
| pos_4974 | Isoleucyl-thiazolidine | metab_4134 | HMDB0253651 | - | 446.2607 | 3.865 | pos | 2M+ACN+H | C9H18N2OS | 0 | 52.5 | -2.6230691 | - | 0.00525326 | 4.7332 | 4.8709 | 4.761 | 4.6939 | 4.8914 | 4.7856 | 4.8621 | 4.7339 | 4.6933 | 4.662 | 4.8585 | 4.8395 | 5.3094 | 5.3092 | 5.3054 |
| pos_4977 | 4-Oxo-1-(3-pyridyl)-1-butanone | metab_4137 | HMDB0062406 | C19567 | 164.0711 | 3.841 | pos | M+H | C9H9NO2 | 0 | 37.4 | 3.08350512 | 76014-80-7 | 0.00282443 | 4.1343 | 4.4715 | 4.1035 | 3.9879 | 4.9939 | 4.9176 | 4.1524 | 3.9269 | 4.9155 | 3.955 | 5.0268 | 5.1841 | 4.9228 | 4.9227 | 4.9206 |
| pos_4979 | Benzamide | metab_4139 | HMDB0004461 | C09815 | 122.0607 | 3.833 | pos | M+H | C7H7NO | 87.9 | 0 | 5.05505616 | 55-21-0 | 0.00598132 | 4.6648 | 4.7648 | 4.692 | 4.6703 | 4.6624 | 4.7589 | 4.7038 | 4.6713 | 4.7607 | 4.6539 | 4.7449 | 4.7458 | 4.7126 | 4.7124 | 4.717 |
| pos_4994 | N-Palmitoyl Asparagine | metab_4153 | HMDB0241921 | - | 278.0706 | 3.7932 | pos | M+NH4 | C10H12O6S | 0 | 40.1 | 4.99744366 | - | 0.01874358 | 4.8735 | 4.9706 | 4.8814 | 4.8302 | 4.8964 | 4.9497 | 4.927 | 4.8408 | 4.9442 | 4.7149 | 4.9099 | 4.9226 | 4.9589 | 4.9455 | 4.9604 |
| pos_5 | Phenylacetylglycine | metab_4158 | HMDB0000821;MJDBOTE0000576 | C05598 | 194.0819 | 4.1384 | pos | M+H, 2M+K, M+2Na-H, M+Na, M+K, M+H-H2O | C10H11NO3 | 77.7 | 0 | 3.58738992 | 500-98-1 | 0.00748784 | 6.5378 | 6.5748 | 6.7713 | 6.7014 | 6.2826 | 6.3403 | 6.6205 | 6.777 | 6.3923 | 6.4073 | 6.582 | 6.5238 | 6.4789 | 6.4727 | 6.4743 |
| pos_500 | N-Undecanoylglycine | metab_4160 | HMDB0013286 | - | 244.1914 | 6.1729 | pos | M+H, M+Na | C13H25NO3 | 0 | 48.9 | 2.5039151 | - | 0.00110178 | 6.4409 | 6.434 | 6.4691 | 6.4448 | 6.3386 | 6.4228 | 6.5067 | 6.4674 | 6.4218 | 6.4442 | 6.4046 | 6.4068 | 6.4523 | 6.4531 | 6.4531 |
| pos_5001 | Etimicin | metab_4162 | HMDB0252101 | - | 500.308 | 3.7622 | pos | M+Na | C21H43N5O7 | 0 | 79.6 | 5.38369539 | - | 0.00849395 | 5.639 | 5.6506 | 5.7393 | 5.6586 | 5.7815 | 5.5997 | 5.684 | 5.6582 | 5.5844 | 5.5875 | 5.7027 | 5.7161 | 5.587 | 5.5815 | 5.5885 |
| pos_5003 | Mecarbam | metab_4163 | HMDB0031800 | C18661 | 362.0854 | 3.7622 | pos | M+CH3OH+H | C10H20NO5PS2 | 0 | 55.9 | -0.3043165 | 2595-54-2 | 0.01952844 | 4.0714 | 3.2097 | 4.4783 | 4.4562 | 2.792 | 3.0792 | 4.3266 | 4.6901 | 2.8737 | 3.8522 | 4.062 | 4.1605 | 3.7421 | 3.7564 | 3.7573 |
| pos_501 | Oct-5-enedioylcarnitine | metab_4169 | HMDB0241709 | - | 316.1763 | 6.1729 | pos | M+H, M+Na | C15H25NO6 | 0 | 39.1 | 2.49683727 | - | 0.00391647 | 3.9387 | 5.1739 | 5.0537 | 4.9929 | 5.271 | 4.9297 | 4.7614 | 4.6981 | 4.941 | 5.3363 | 5.0244 | 4.9153 | 5.8934 | 5.8951 | 5.8968 |
| pos_5024 | 4-(2-Aminophenyl)-2,4-dioxobutanoic acid | metab_4183 | HMDB0000978;PW_C000778 | C01252 | 208.0611 | 3.703 | pos | M+H | C10H9NO4 | 0 | 60.1 | 3.31398906 | 90924-76-8 | 0.00738376 | 3.974 | 3.981 | 3.7106 | 3.8157 | 4.8008 | 4.7247 | 3.9683 | 3.9274 | 4.5115 | 3.2957 | 4.6116 | 4.6123 | 4.3788 | 4.3784 | 4.3731 |
| pos_5031 | N-Methylphenylalanine | metab_4191 | HMDB0029224 | - | 197.1292 | 3.6709 | pos | M+NH4 | C10H13NO2 | 50.6 | 0 | 3.93238432 | - | 0.00826192 | 3.8417 | 4.2574 | 4.0764 | 4.1352 | 4.6499 | 4.6868 | 4.1998 | 4.0805 | 4.7677 | 4.2189 | 4.6837 | 4.5786 | 4.4717 | 4.4769 | 4.4786 |
| pos_5032 | Quinoline-4,8-diol | metab_4192 | HMDB0060289 | C05637 | 162.0555 | 3.6709 | pos | M+H | C9H7NO2 | 0 | 61.3 | 3.60594946 | - | 0.00369747 | 4.2088 | 4.2134 | 4.2646 | 4.4183 | 4.9587 | 5.0245 | 4.1783 | 4.147 | 5.057 | 4.0081 | 4.9605 | 5.0518 | 4.9936 | 4.9934 | 4.9963 |
| pos_5037 | 3-Hydroxybutyrylcarnitine | metab_4197 | LMFA07070037;HMDB0013127;HMDB0062735 | - | 248.1498 | 3.6553 | pos | M+H | C11H21NO5 | 0 | 46.2 | 2.41711757 | 1469900-92-2 | 0.01683802 | 3.9086 | 3.9328 | 3.9557 | 3.7429 | 3.7896 | 3.8705 | 4.0546 | 3.801 | 3.9412 | 3.8017 | 3.9494 | 3.8984 | 4.292 | 4.28 | 4.2934 |
| pos_5039 | Kynurenic Acid | metab_4199 | HMDB0000715;PW_C000566;MJDBOTE0000367 | C01717 | 190.0505 | 3.6398 | pos | M+H | C10H7NO3 | 84.4 | 0 | 3.34891178 | 492-27-3 | 0.07558692 | 3.854 | 3.8055 | 3.7314 | 3.8229 | 3.7188 | 3.6392 | 3.8011 | 3.7792 | 3.7791 | 3.6589 | 3.7797 | 3.9492 | 3.8846 | 3.8844 | 3.8263 |
| pos_5042 | Estriol | metab_4201 | HMDB0000153;HMDB0000347;PW_C000100;MJDBOTE0000808 | C05141 | 333.1452 | 3.6318 | pos | M+2Na-H | C18H24O3 | 0 | 62.8 | 5.05236155 | 50-27-1;547-81-9 | 0.01393009 | 4.0259 | 3.4573 | 4.1597 | 3.5767 | 3.3324 | 3.6328 | 4.3627 | 3.9943 | 3.5359 | 4.0483 | 3.6107 | 3.774 | 4.2126 | 4.2124 | 4.2229 |
| pos_5051 | Arterolane | metab_4206 | HMDB0248627 | - | 456.2816 | 3.5918 | pos | M+ACN+Na | C22H36N2O4 | 0 | 76.7 | -4.3332188 | - | 0.01895657 | 5.3352 | 5.3339 | 5.4386 | 5.3488 | 5.5049 | 5.3211 | 5.4039 | 5.3973 | 5.2962 | 5.287 | 5.4628 | 5.4442 | 5.3924 | 5.4072 | 5.3938 |
| pos_5052 | Hept-4-enedioylcarnitine | metab_4207 | HMDB0241688 | - | 266.1393 | 3.5918 | pos | M+H-2H2O | C14H23NO6 | 0 | 35.4 | 2.1409575 | - | 0.00874837 | 5.063 | 5.3232 | 5.099 | 4.9831 | 5.2071 | 5.3461 | 5.2925 | 4.8551 | 5.1539 | 5.1411 | 5.3499 | 5.2762 | 5.2194 | 5.2266 | 5.2209 |
| pos_5054 | 5-Hydroxyindoleacetate | metab_4209 | HMDB0000763 | C05635 | 192.0661 | 3.5918 | pos | M+H | C10H9NO3 | 93.8 | 0 | 3.24912623 | 54-16-0 | 0.00756201 | 4.3458 | 4.4147 | 4.4031 | 4.545 | 4.1106 | 4.2724 | 4.4989 | 4.3509 | 4.5167 | 4.285 | 4.4432 | 4.5309 | 4.4172 | 4.4235 | 4.4186 |
| pos_5056 | 3-Hydroxybenzaldehyde | metab_4211 | - | C03067 | 105.0343 | 3.5758 | pos | M+H-H2O | C7H6O2 | 58 | 0 | 6.46581747 | 100-83-4 | 0.0219521 | 3.9918 | 4.2021 | 3.939 | 3.8009 | 4.1482 | 4.2066 | 4.0075 | 4.0082 | 4.1939 | 3.7931 | 4.3616 | 4.0473 | 3.9713 | 3.987 | 3.9887 |
| pos_5057 | Alpha-Methyl-4-carboxyphenylglycine | metab_4212 | HMDB0243703 | - | 251.1033 | 3.5678 | pos | M+ACN+H | C10H11NO4 | 0 | 44.7 | 3.18215705 | - | 0.00476159 | 5.1809 | 5.47 | 5.162 | 4.8724 | 5.4122 | 5.478 | 5.1424 | 5.1719 | 5.4506 | 4.8 | 5.6834 | 5.3141 | 5.2217 | 5.2258 | 5.2231 |
| pos_5060 | (9Z)-12-oxo-dodec-9-enoate | metab_4216 | HMDB0303984 | - | 212.14 | 3.5598 | pos | M+H | C12H19O3- | 0 | 52.3 | -3.2016102 | - | 0.00639185 | 5.3006 | 5.5317 | 5.4144 | 4.8092 | 5.3281 | 5.7289 | 5.7003 | 3.9391 | 3.3047 | 4.8879 | 5.7255 | 5.3627 | 5.412 | 5.412 | 5.4072 |
| pos_5066 | Racemetirosine | metab_4222 | HMDB0247174;HMDB0014903 | C07921 | 160.0762 | 3.5278 | pos | M+H-2H2O | C10H13NO3 | 0 | 33.3 | 2.68143855 | 672-87-7 | 0.00413273 | 4.5857 | 5.0058 | 4.7897 | 5.0626 | 4.6102 | 4.8217 | 4.8391 | 4.3468 | 4.6258 | 4.2596 | 4.9043 | 4.7009 | 4.6719 | 4.6705 | 4.674 |
| pos_5067 | Beta-Tyrosine | metab_4223 | HMDB0003831 | C04368 | 164.0711 | 3.5198 | pos | M+H-H2O | C9H11NO3 | 0 | 77.9 | 2.87082018 | - | 0.01775413 | 4.1351 | 4.2829 | 4.2266 | 4.1907 | 4.6327 | 4.7266 | 4.2546 | 4.0404 | 4.635 | 3.9231 | 4.7477 | 4.7418 | 4.3462 | 4.3335 | 4.3476 |
| pos_5071 | Ritalinic Acid | metab_4227 | HMDB0042008 | - | 220.1339 | 3.5033 | pos | M+H | C13H17NO2 | 44.9 | 0 | 3.39264286 | 19395-41-6 | 0.0134661 | 4.0117 | 4.3358 | 4.5258 | 4.2205 | 4.2531 | 4.1023 | 4.5927 | 3.9874 | 4.3756 | 4.374 | 4.2371 | 4.571 | 4.1437 | 4.1329 | 4.1346 |
| pos_5072 | N-(3-Amino-3-oxopropyl)-L-valine | metab_4228 | HMDB0246894 | - | 171.1133 | 3.4954 | pos | M+H-H2O | C8H16N2O3 | 0 | 32.9 | 2.69115639 | - | 0.02373157 | 3.7797 | 4.8936 | 4.402 | 4.5388 | 3.927 | 4.2098 | 4.2579 | 3.6456 | 3.2576 | 2.9205 | 4.3888 | 4.0907 | 4.2881 | 4.3005 | 4.2801 |
| pos_5077 | Glutamylisoleucine | metab_4233 | HMDB0028822 | - | 225.1239 | 3.4714 | pos | M+H-2H2O | C11H20N2O5 | 0 | 39.9 | 2.16831213 | 5879-22-1 | 0.01228767 | 4.0137 | 4.3662 | 4.0825 | 3.9172 | 4.8579 | 4.558 | 4.307 | 3.9389 | 4.628 | 3.9884 | 4.5958 | 4.7676 | 4.4582 | 4.4482 | 4.4501 |
| pos_5097 | Lamivudine-monophosphate | metab_4248 | PW_C040659;HMDB0060641 | - | 636.0757 | 3.3762 | pos | 2M+NH4 | C8H12N3O6PS | 0 | 47.9 | 8.01225864 | - | 0.15565504 | 5.3203 | 5.3047 | 5.1281 | 5.3043 | 4.8146 | 5.2527 | 5.2767 | 5.146 | 5.3729 | 5.4306 | 5.2491 | 5.1064 | 5.1777 | 5.041 | 5.1197 |
| pos_510 | 12a-Hydroxy-3-oxocholadienic acid | metab_4251 | HMDB0000385 | - | 428.2804 | 6.2364 | pos | M+H-H2O, M+ACN+H | C24H34O4 | 0 | 48 | 2.11801075 | 13535-96-1 | 0.01376045 | 0.8473 | 4.1043 | 3.637 | 3.5083 | 4.4901 | 5.0047 | 0.8604 | 0.8663 | 5.0774 | 0.869 | 4.7264 | 4.0195 | 4.7229 | 4.7338 | 4.7242 |
| pos_5107 | (4-Chloro-2-methylphenoxy)acetic acid | metab_4259 | HMDB0254366 | C18528 | 183.0213 | 3.33 | pos | M+H-H2O | C9H9ClO3 | 0 | 52.7 | 2.58529695 | - | 0.05665726 | 5.0372 | 5.0446 | 5.0621 | 5.056 | 4.8276 | 5.1399 | 4.9479 | 5.0752 | 5.1335 | 5.1195 | 5.1426 | 5.0651 | 5.089 | 5.0469 | 5.0913 |
| pos_5109 | 4-Chlorobenzaldehyde | metab_4261 | HMDB0246392 | C06648 | 141.0106 | 3.33 | pos | M+H | C7H5ClO | 0 | 32.6 | 2.98164106 | 104-88-1 | 0.00547911 | 5.9088 | 5.9145 | 5.9218 | 5.9016 | 5.8822 | 5.9062 | 5.8684 | 5.9149 | 5.9441 | 5.9114 | 5.9243 | 5.8966 | 5.9403 | 5.9449 | 5.9417 |
| pos_5110 | 2-Phenylacetamide | metab_4263 | HMDB0010715 | C02505 | 118.0658 | 3.33 | pos | M+H-H2O | C8H9NO | 64.4 | 0 | 4.86265628 | 103-81-1 | 0.00383132 | 6.6646 | 6.6748 | 6.6686 | 6.6635 | 6.6144 | 6.6386 | 6.6582 | 6.6582 | 6.6603 | 6.6534 | 6.6531 | 6.6312 | 6.6902 | 6.6883 | 6.6916 |
| pos_5119 | D-Cathine | metab_4270 | - | C08300 | 134.0969 | 3.2904 | pos | M+H-H2O | C9H13NO | 43.8 | 0 | 3.26032004 | 492-39-7 | 0.00948529 | 4.5566 | 4.5751 | 4.534 | 4.4369 | 4.5524 | 4.5288 | 4.7336 | 4.5526 | 4.7132 | 4.5934 | 4.7703 | 4.6521 | 4.5668 | 4.5664 | 4.5595 |
| pos_5120 | L-Prolinamide, 5-oxo-L-prolyl-L-norvalyl- | metab_4272 | HMDB0253911 | - | 342.2129 | 3.2824 | pos | M+NH4 | C15H24N4O4 | 0 | 60.4 | -2.0398658 | - | 0.01443376 | 3.2898 | 3.2675 | 4.026 | 3.5328 | 4.0058 | 3.6042 | 3.5153 | 3.2755 | 3.7342 | 3.7967 | 3.8239 | 4.1218 | 5.0989 | 5.109 | 5.1105 |
| pos_5121 | (6Z)-Oct-6-enedioylcarnitine | metab_4273 | HMDB0241707 | - | 316.1763 | 3.2824 | pos | M+H | C15H25NO6 | 0 | 57.8 | 2.55191379 | - | 0.01057722 | 5.6144 | 4.4535 | 5.0094 | 4.8176 | 4.6915 | 4.6494 | 4.5255 | 5.3725 | 4.9544 | 5.1933 | 4.7605 | 5.3235 | 5.0299 | 5.0384 | 5.0312 |
| pos_5124 | Serotonin | metab_4275 | HMDB0000259;PW_C000178;MJDBSJD0000073 | C00780 | 159.0922 | 3.2824 | pos | M+H-H2O | C10H12N2O | 0 | 64.4 | 2.72076362 | 50-67-9 | 0.0120096 | 5.3707 | 5.698 | 5.5615 | 5.5587 | 5.3897 | 5.3278 | 5.686 | 5.4726 | 5.3554 | 5.3974 | 5.3934 | 5.477 | 5.5401 | 5.5398 | 5.5309 |
| pos_5128 | Isoquinoline | metab_4279 | HMDB0034244 | C06323 | 130.0656 | 3.2824 | pos | M+H | C9H7N | 41.8 | 0 | 4.03125441 | 119-65-3 | 0.00174237 | 4.7511 | 5.0041 | 4.9199 | 4.9035 | 4.7397 | 4.7514 | 5.0111 | 4.8557 | 4.7344 | 4.769 | 4.8224 | 4.8454 | 4.8784 | 4.8782 | 4.877 |
| pos_5139 | Candicine | metab_4289 | HMDB0303388 | C10575 | 244.1551 | 3.2424 | pos | M+ACN+Na | C11H18NO+ | 0 | 36.3 | 2.54018154 | 6656-13-9 | 0.00580975 | 4.5145 | 4.5543 | 4.6388 | 4.4111 | 4.4491 | 4.6252 | 4.6284 | 4.407 | 4.4947 | 4.4414 | 4.5437 | 4.5921 | 4.4946 | 4.4911 | 4.496 |
| pos_5142 | Coniferaldehyde | metab_4293 | HMDB0141782 | C02666 | 196.0975 | 3.2344 | pos | M+NH4 | C10H10O3 | 0 | 51.3 | 3.63241369 | 20649-42-7;458-36-6 | 0.03234975 | 4.2763 | 4.1377 | 4.3282 | 4.158 | 4.2754 | 4.3519 | 4.2852 | 4.3202 | 4.4089 | 4.2323 | 4.3959 | 4.5274 | 4.2667 | 4.2429 | 4.2681 |
| pos_5145 | Dimboa glucoside | metab_4296 | HMDB0301843 | - | 338.0878 | 3.2264 | pos | M+H-2H2O | C15H19NO10 | 0 | 70.3 | 2.1529357 | - | 0.00163035 | 2.7807 | 3.0109 | 3.4767 | 2.8062 | 4.6209 | 4.6519 | 3.3402 | 2.8027 | 4.5425 | 2.8059 | 4.6561 | 4.7917 | 4.146 | 4.145 | 4.1464 |
| pos_5151 | 6-(2-Hydroxyethoxy)-6-oxohexanoic acid | metab_4303 | HMDB0061681 | - | 173.0816 | 3.2104 | pos | M+H-H2O | C8H14O5 | 0 | 40.2 | 4.12739089 | - | 0.01795779 | 3.9863 | 4.0904 | 4.0284 | 3.972 | 3.9661 | 4.0086 | 4.0559 | 3.8672 | 4.0703 | 4.0665 | 3.9487 | 4.0901 | 4.1826 | 4.197 | 4.1847 |
| pos_516 | Curcumenol | metab_4310 | HMDB0033960;MJDBOTE0000956 | C16942 | 510.358 | 6.2602 | pos | M+H-2H2O, 2M+ACN+H | C15H22O2 | 0 | 66.6 | 0.54526761 | 19431-84-6 | 0.02938133 | 4.7914 | 5.2602 | 5.0461 | 4.9284 | 5.228 | 5.2319 | 4.6966 | 4.8988 | 5.256 | 4.9447 | 5.2258 | 5.1069 | 4.6109 | 4.613 | 4.6338 |
| pos_5161 | (1R,2R,4S)-p-Menthane-1,2,8-triol 8-glucoside | metab_4311 | HMDB0039894 | - | 368.2288 | 3.1784 | pos | M+NH4 | C16H30O8 | 0 | 59.7 | 2.60770166 | 378254-09-2 | 0.00958342 | 4.4578 | 4.4773 | 4.543 | 4.4857 | 4.6887 | 4.4263 | 4.4961 | 4.4884 | 4.4099 | 4.4006 | 4.5478 | 4.6287 | 5.163 | 5.1693 | 5.1709 |
| pos_5165 | 3-Hydroxy-2-oxoindole | metab_4313 | HMDB0240741 | C11130 | 132.045 | 3.1784 | pos | M+H-H2O | C8H7NO2 | 0 | 34.4 | 3.79523849 | - | 0.02334292 | 4.4438 | 4.1823 | 4.5211 | 3.9822 | 4.233 | 4.1059 | 4.3352 | 4.519 | 4.4931 | 4.4405 | 3.9868 | 3.9735 | 4.3598 | 4.3417 | 4.3432 |
| pos_517 | Hydroxytetradecadienyl-l-carnitine | metab_4317 | HMDB0253295 | - | 370.2959 | 6.2682 | pos | M+H, M+ACN+Na | C21H39NO4 | 0 | 70.1 | 2.00668325 | - | 0.02807437 | 5.61 | 4.9965 | 4.8866 | 4.9764 | 4.5922 | 4.4875 | 5.1534 | 5.1759 | 4.9693 | 5.3397 | 4.7558 | 4.6054 | 5.3221 | 5.3219 | 5.3429 |
| pos_5175 | 8-Amino-7-oxononanoic acid | metab_4323 | LMFA01060168;HMDB0240687 | C01092 | 229.1553 | 3.1348 | pos | M+ACN+H | C9H17NO3 | 0 | 66.3 | 3.40111414 | 682799-71-9 | 0.01303989 | 4.6702 | 4.5243 | 4.5461 | 4.2593 | 4.2459 | 4.4928 | 4.5816 | 4.7789 | 4.4137 | 4.1944 | 4.1975 | 4.6191 | 4.4012 | 4.3902 | 4.3936 |
| pos_5186 | Leucyl-Glutamine | metab_4335 | HMDB0028927 | - | 242.1508 | 3.0788 | pos | M+H-H2O | C11H21N3O4 | 0 | 75.1 | 3.53100461 | - | 0.04446492 | 2.3048 | 4.4023 | 4.1997 | 4.506 | 3.7365 | 3.8815 | 3.8217 | 3.6386 | 2.8531 | 2.0454 | 4.261 | 4.1362 | 3.807 | 3.7739 | 3.8085 |
| pos_5194 | Glycyl-leucine | metab_4344 | HMDB0000759;MJDBOTE0000578 | C02155 | 189.1239 | 3.0628 | pos | M+H | C8H16N2O3 | 81.8 | 0 | 3.02076681 | 869-19-2 | 0.00123953 | 3.7845 | 3.6228 | 3.7858 | 3.6311 | 3.4673 | 3.5046 | 3.5835 | 3.4882 | 3.4669 | 3.5356 | 3.6337 | 3.5813 | 4.8714 | 4.8712 | 4.8723 |
| pos_520 | PC(16:0/20:3(5Z,8Z,11Z)) | metab_4350 | LMGP01010622;HMDB0007980;PW_C004029 | C00157 | 784.5853 | 6.2761 | pos | M+H, M+Na | C44H82NO8P | 0 | 78.7 | 0.24846134 | - | 0.01864241 | 5.8631 | 5.8489 | 5.8123 | 5.5839 | 5.7538 | 5.8528 | 5.795 | 5.7746 | 5.9323 | 5.7899 | 5.8242 | 5.802 | 5.9707 | 5.9707 | 5.9566 |
| pos_5213 | 2-Aminobenzoic Acid | metab_4364 | HMDB0001123;PW_C000877 | C00108 | 120.0451 | 3.0068 | pos | M+H-H2O | C7H7NO2 | 63.1 | 0 | 5.02909892 | 118-92-3 | 0.01213377 | 4.5877 | 4.7079 | 4.302 | 4.3128 | 4.4119 | 4.1907 | 4.5523 | 4.5993 | 4.5214 | 4.4659 | 4.1076 | 4.3082 | 4.3074 | 4.3172 | 4.3089 |
| pos_5219 | Felbamate | metab_4370 | PW_C009391;HMDB0015084 | C07501 | 221.0928 | 2.9828 | pos | M+H-H2O | C11H14N2O4 | 0 | 33.4 | 2.89992657 | 25451-15-4 | 0.02999888 | 3.4346 | 4.6258 | 3.9565 | 4.2534 | 3.7742 | 3.9239 | 4.0079 | 3.2963 | 3.4549 | 2.8809 | 4.1675 | 3.8639 | 3.969 | 3.9688 | 3.9913 |
| pos_5225 | Dopamine quinone | metab_4377 | HMDB0012219 | C17755 | 193.0979 | 2.9668 | pos | M+ACN+H | C8H9NO2 | 0 | 63.3 | 4.8449432 | 50673-96-6 | 0.02792452 | 3.4016 | 3.8499 | 3.5466 | 3.5712 | 4.5177 | 4.5343 | 3.6676 | 3.2135 | 4.4061 | 3.5248 | 4.0813 | 4.4659 | 4.2369 | 4.2583 | 4.238 |
| pos_5227 | 1,2,5-Trihydroxypyrrole-3-sulfonic acid | metab_4379 | HMDB0255342 | - | 159.9698 | 2.9668 | pos | M+H-2H2O | C4H5NO6S | 0 | 41.4 | -0.7131421 | - | 0.00434328 | 3.6964 | 3.6533 | 4.2245 | 3.6387 | 3.4627 | 3.5746 | 3.5866 | 3.7487 | 3.6149 | 3.7232 | 3.6507 | 3.8396 | 3.7297 | 3.7263 | 3.7295 |
| pos_5233 | 2,3,4,5-Tetrahydro-2-pyridinecarboxylic acid | metab_4385 | HMDB0012130 | C00450 | 169.0977 | 2.9508 | pos | M+ACN+H | C6H9NO2 | 0 | 41.8 | 4.49008865 | 73980-78-6 | 0.00347938 | 3.8619 | 4.543 | 4.1866 | 4.4005 | 4.3497 | 4.4487 | 4.1402 | 3.9668 | 4.292 | 3.7742 | 4.3825 | 4.2793 | 4.3802 | 4.3772 | 4.3782 |
| pos_5238 | O-Toluidine | metab_4388 | HMDB0041965 | C14403 | 108.0815 | 2.9348 | pos | M+H | C7H9N | 69.7 | 0 | 7.22664667 | 95-53-4 | 0.00606347 | 5.0484 | 5.0395 | 5.0421 | 5.0679 | 4.9942 | 5.0566 | 5.0509 | 5.0585 | 5.0428 | 5.104 | 5.0373 | 5.0205 | 5.0798 | 5.0796 | 5.0842 |
| pos_5246 | Pyridoxine | metab_4396 | HMDB0301760;PW_C000161;HMDB0000239;MJDBOTE0000424 | C00314 | 170.0817 | 2.9107 | pos | M+H | C8H11NO3 | 35.3 | 0 | 3.27501368 | 65-23-6;58-56-0 | 0.00330854 | 4.5765 | 4.8566 | 4.6127 | 4.6372 | 4.5183 | 4.6971 | 4.8232 | 4.4111 | 4.4052 | 4.5789 | 4.687 | 4.7734 | 4.6133 | 4.6104 | 4.6121 |
| pos_5248 | Gamma-Glutamyltyrosine | metab_4398 | HMDB0011741 | - | 311.1245 | 2.9027 | pos | M+H | C14H18N2O6 | 86.1 | 0 | 2.35319296 | 7432-23-7 | 0.00361783 | 4.6642 | 5.0316 | 4.7916 | 4.5796 | 4.731 | 4.6982 | 4.8668 | 4.6534 | 4.5991 | 4.5835 | 4.6899 | 4.7938 | 4.8004 | 4.8015 | 4.8035 |
| pos_525 | Styrene Oxide | metab_4400 | HMDB0062765 | C02083 | 103.0549 | 6.3158 | pos | M+H-H2O, M+H | C8H8O | 0 | 33.7 | 5.79918295 | 96-09-3 | 0.0027942 | 5.0987 | 4.8655 | 4.8328 | 4.8634 | 4.8652 | 4.8741 | 4.8548 | 4.9043 | 4.932 | 4.9919 | 4.9089 | 4.8972 | 4.8544 | 4.8551 | 4.8568 |
| pos_5250 | Pivagabine | metab_4401 | HMDB0246575 | - | 205.1552 | 2.8867 | pos | M+NH4 | C9H17NO3 | 0 | 34.1 | 2.83428246 | - | 0.0375828 | 3.9479 | 3.5771 | 3.9383 | 3.9672 | 3.9802 | 4.2457 | 3.1075 | 3.805 | 3.6602 | 3.2214 | 3.9374 | 3.996 | 4.3045 | 4.3332 | 4.3059 |
| pos_5251 | Valeric acid | metab_4402 | HMDB0000892;LMFA01010005 | C00803 | 144.1024 | 2.8867 | pos | M+ACN+H | C5H10O2 | 0 | 36.9 | 4.86051693 | 109-52-4 | 0.01058389 | 4.4062 | 4.5474 | 4.52 | 4.3669 | 4.7088 | 4.7921 | 4.5462 | 4.455 | 4.7866 | 4.4774 | 4.7345 | 4.7973 | 4.7446 | 4.7517 | 4.7533 |
| pos_5252 | DL-Norvaline | metab_4403 | HMDB0013716;HMDB0251527 | C01799 | 100.0765 | 2.8867 | pos | M+H-H2O | C5H11NO2 | 0 | 33.8 | 7.17073444 | 760-78-1 | 0.00272694 | 5.268 | 5.3492 | 5.3307 | 5.3419 | 5.327 | 5.4801 | 5.2542 | 5.3098 | 5.3424 | 5.2551 | 5.3968 | 5.3391 | 5.4261 | 5.4259 | 5.424 |
| pos_5254 | Coformycin | metab_4405 | HMDB0250398 | C01677 | 267.1094 | 2.8787 | pos | M+H-H2O | C11H16N4O5 | 0 | 34.4 | 2.06102118 | 11033-22-0 | 0.03149821 | 3.5246 | 4.2766 | 3.9909 | 3.7528 | 4.1986 | 4.0953 | 4.0599 | 4.1832 | 4.0493 | 4.1672 | 4.3442 | 4.2162 | 4.5285 | 4.5527 | 4.53 |
| pos_5255 | 7-Aminomethyl-7-carbaguanine | metab_4406 | HMDB0011690 | C16675 | 180.0883 | 2.8787 | pos | M+H | C7H9N5O | 0 | 44.6 | 1.78075111 | - | 0.02007239 | 4.8548 | 4.9013 | 4.8497 | 4.8078 | 4.756 | 4.7995 | 4.8136 | 4.8979 | 4.9563 | 4.7985 | 4.8043 | 4.8819 | 4.7329 | 4.7328 | 4.7479 |
| pos_5257 | 3-Hydroxy-L-proline | metab_4408 | HMDB0002113 | C04397 | 173.0927 | 2.8628 | pos | M+ACN+H | C5H9NO3 | 0 | 39 | 4.9660912 | 8/2/98 | 0.02808276 | 4.1881 | 4.7278 | 4.4374 | 4.3609 | 4.4044 | 4.7617 | 4.6663 | 2.6193 | 2.5567 | 3.6647 | 4.7732 | 4.5121 | 4.3122 | 4.3325 | 4.3343 |
| pos_5258 | 8'-hydroxyabscisate | metab_4409 | HMDB0304246 | C15514 | 262.1194 | 2.8548 | pos | M+H-H2O | C15H19O5- | 0 | 38 | -2.1079016 | - | 0.01550583 | 4.082 | 4.7451 | 4.4303 | 4.0352 | 4.38 | 4.907 | 4.8715 | 3.7103 | 3.7107 | 3.7135 | 5.0324 | 4.4104 | 4.4418 | 4.4539 | 4.4429 |
| pos_5260 | Tenuazonic acid | metab_4411 | HMDB0036074 | C08511 | 180.1025 | 2.8548 | pos | M+H-H2O | C10H15NO3 | 0 | 46.6 | 3.06189049 | 27778-66-1;610-88-8 | 0.00209284 | 4.5528 | 4.5948 | 4.6329 | 4.5873 | 4.3613 | 4.5148 | 4.5404 | 4.5029 | 4.3788 | 4.386 | 4.5162 | 4.6729 | 4.545 | 4.5464 | 4.5447 |
| pos_5263 | Cotinine | metab_4413 | PW_C000824;HMDB0001046;MJDBOTE0001185 | - | 209.129 | 2.8468 | pos | M+CH3OH+H | C10H12N2O | 32.6 | 0 | 2.99611745 | 486-56-6 | 0.00490609 | 4.545 | 4.8938 | 4.8183 | 4.6208 | 4.8126 | 4.8171 | 4.9216 | 4.213 | 4.9669 | 4.7608 | 4.7609 | 4.9704 | 4.7613 | 4.7646 | 4.7653 |
| pos_5272 | Cyclohexa-2,4-dienylmethanol | metab_4423 | HMDB0250648 | - | 93.0707 | 2.8309 | pos | M+H-H2O | C7H10O | 0 | 43.9 | 7.89342197 | - | 0.01345365 | 4.3249 | 4.3977 | 4.315 | 4.2633 | 4.2505 | 4.3392 | 4.4151 | 4.3578 | 4.3292 | 4.3399 | 4.2781 | 4.4273 | 4.3807 | 4.39 | 4.3916 |
| pos_5273 | Benzaldehyde | metab_4424 | HMDB0006115 | C00261 | 107.0499 | 2.8309 | pos | M+H | C7H6O | 0 | 47.9 | 7.18353768 | 100-52-7 | 0.00754153 | 4.9872 | 5.0766 | 4.9959 | 4.8999 | 4.8758 | 4.9736 | 5.0509 | 5.0145 | 4.9467 | 4.9399 | 4.9042 | 5.0647 | 5.0236 | 5.0283 | 5.0299 |
| pos_5274 | Adipate semialdehyde | metab_4425 | HMDB0012882 | C06102 | 95.05 | 2.8309 | pos | M+H-2H2O | C6H10O3 | 0 | 36.8 | 6.64953248 | 928-81-4 | 0.00366121 | 5.1414 | 5.2179 | 5.1495 | 5.0595 | 5.0518 | 5.1199 | 5.2021 | 5.1394 | 5.1296 | 5.114 | 5.0575 | 5.2103 | 5.1849 | 5.1881 | 5.1863 |
| pos_528 | TAURODEOXYCHOLIC ACID | metab_4429 | PW_C000715;LMST05040013;HMDB0000896 | C05463 | 500.305 | 6.3237 | pos | M+H-H2O, M+H | C26H45NO6S | 85.1 | 0 | 1.94195021 | 516-50-7 | 0.01494418 | 6.4941 | 6.2657 | 5.8344 | 5.6698 | 6.7296 | 6.5141 | 5.9968 | 5.9701 | 6.4141 | 6.4577 | 6.2791 | 6.5951 | 6.1501 | 6.1605 | 6.1621 |
| pos_5281 | L-Formylkynurenine | metab_4431 | HMDB0060485 | C02700 | 237.0876 | 2.8229 | pos | M+H | C11H12N2O4 | 0 | 70.1 | 2.54012357 | 1022-31-7 | 0.00568664 | 4.935 | 4.8997 | 4.8614 | 4.7051 | 4.7199 | 4.3714 | 4.9295 | 4.6 | 4.4865 | 4.4014 | 4.7974 | 4.5732 | 4.8268 | 4.83 | 4.8317 |
| pos_5282 | L-Kynurenine | metab_4432 | HMDB0000684;PW_C000538 | C00328 | 231.0747 | 2.8229 | pos | M+Na | C10H12N2O3 | 0 | 62.5 | 3.40766849 | 2922-83-0 | 0.02548385 | 5.3773 | 5.3387 | 5.057 | 4.9066 | 5.1365 | 4.8293 | 5.2111 | 5.1433 | 5.0466 | 5.0506 | 4.9206 | 5.3238 | 5.2401 | 5.2569 | 5.2612 |
| pos_5283 | (2R,2'S)-Isobuteine | metab_4433 | HMDB0030411 | - | 190.0539 | 2.8229 | pos | M+H-H2O | C7H13NO4S | 0 | 45 | 3.08548872 | 66512-75-2 | 0.0160575 | 4.8248 | 4.2699 | 4.029 | 3.8538 | 4.5 | 4.2504 | 4.7503 | 4.355 | 4.115 | 4.4787 | 4.0703 | 4.4995 | 4.6569 | 4.6682 | 4.6698 |
| pos_5289 | 5-Methyldeoxycytidine | metab_4437 | HMDB0002224 | C03592 | 305.1213 | 2.8151 | pos | M+ACN+Na | C10H15N3O4 | 0 | 57.3 | -2.8827609 | 838-07-3 | 0.02396187 | 3.9274 | 4.9927 | 4.6293 | 4.5571 | 4.7836 | 4.7968 | 4.5977 | 4.2709 | 4.7112 | 4.4822 | 4.706 | 4.8245 | 4.9337 | 4.9338 | 4.9516 |
| pos_529 | Tauroursodeoxycholic acid | metab_4438 | LMST05040015;HMDB0000874 | C16868 | 464.284 | 6.3237 | pos | M+H-2H2O, M+Na | C26H45NO6S | 0 | 59.6 | 2.15360151 | 14605-22-2 | 0.01110127 | 7.2102 | 6.8531 | 6.4246 | 6.338 | 7.1541 | 6.9776 | 6.5688 | 6.6263 | 6.9185 | 6.9411 | 6.9266 | 7.0865 | 6.7027 | 6.7117 | 6.7042 |
| pos_5292 | Threonylproline | metab_4440 | HMDB0029069 | - | 199.1083 | 2.8151 | pos | M+H-H2O | C9H16N2O4 | 0 | 62.9 | 2.59282082 | 46398-79-2 | 0.02686171 | 4.4454 | 4.7112 | 4.639 | 4.5297 | 4.854 | 4.8622 | 4.5656 | 4.5045 | 4.8082 | 4.4173 | 4.6675 | 4.7589 | 4.5218 | 4.5414 | 4.5429 |
| pos_5295 | L-Theanine | metab_4443 | HMDB0034365 | C01047 | 157.0978 | 2.8151 | pos | M+H-H2O | C7H14N2O3 | 0 | 33.7 | 3.70616763 | 3081-61-6 | 0.03666556 | 3.8202 | 4.8742 | 4.4261 | 4.5747 | 4.324 | 4.2765 | 4.3188 | 4.0603 | 4.1054 | 3.8339 | 4.4429 | 4.234 | 4.3276 | 4.3275 | 4.3548 |
| pos_5296 | Imexon | metab_4444 | HMDB0253411 | - | 112.0513 | 2.8151 | pos | M+H | C4H5N3O | 0 | 44.4 | 6.50051435 | - | 0.01950276 | 4.8016 | 4.1026 | 4.5274 | 4.6589 | 4.4688 | 4.5978 | 4.8341 | 4.94 | 5.1119 | 4.6505 | 4.6954 | 4.7285 | 4.553 | 4.5691 | 4.5659 |
| pos_5299 | Aniline | metab_4447 | HMDB0003012 | C00292 | 94.066 | 2.8151 | pos | M+H | C6H7N | 54.4 | 0 | 9.45481446 | 62-53-3 | 0.00431484 | 5.0035 | 4.9391 | 4.7971 | 4.6938 | 4.8516 | 4.6246 | 4.9011 | 4.8282 | 4.7911 | 4.7704 | 4.6688 | 4.9563 | 4.9363 | 4.94 | 4.9377 |
| pos_5304 | 1-Methylguanosine | metab_4453 | HMDB0001563 | C04545 | 298.1152 | 2.8071 | pos | M+H | C11H15N5O5 | 91.1 | 0 | 2.08912247 | 2140-65-0 | 0.02540446 | 5.1978 | 5.2138 | 5.2234 | 5.1192 | 5.145 | 5.0226 | 5.1279 | 5.226 | 5.287 | 5.1594 | 5.2093 | 5.2519 | 5.0161 | 5.0343 | 5.0362 |
| pos_531 | Prehumulinic acid | metab_4458 | HMDB0030148 | - | 281.173 | 6.3554 | pos | M+H-H2O, M+H | C16H24O4 | 0 | 49.5 | -6.2780214 | - | 0.01884211 | 4.6931 | 4.7112 | 5.1649 | 5.1575 | 4.6558 | 4.9435 | 5.025 | 5.1693 | 5.1027 | 5.0096 | 4.9191 | 4.9569 | 5.0504 | 5.0353 | 5.0376 |
| pos_5313 | Thymine | metab_4462 | PW_C000179;HMDB0000262;MJDBOTE0000713 | C00178 | 127.0508 | 2.8071 | pos | M+H | C5H6N2O2 | 87.2 | 0 | 4.52597598 | 65-71-4 | 0.04171598 | 5.0294 | 5.0671 | 4.7573 | 4.779 | 5.0218 | 4.9779 | 4.8214 | 4.9856 | 5.0847 | 4.9003 | 4.9979 | 5.1227 | 4.8207 | 4.848 | 4.8555 |
| pos_5328 | 2-Isopropylmalic acid | metab_4477 | HMDB0000402 | C02504 | 159.0666 | 2.7831 | pos | M+H-H2O | C7H12O5 | 0 | 55.4 | 7.96785615 | 49601-06-1 | 0.07794976 | 4.3074 | 4.258 | 4.3379 | 4.3886 | 4.123 | 4.2531 | 4.2407 | 3.9501 | 4.2279 | 4.1775 | 4.1478 | 4.2982 | 4.2409 | 4.1825 | 4.1846 |
| pos_5330 | Seryllysine | metab_4480 | HMDB0029044 | - | 198.1244 | 2.7751 | pos | M+H-2H2O | C9H19N3O4 | 0 | 59.8 | 3.09096041 | 22677-61-8 | 0.01005889 | 3.7073 | 4.3457 | 3.9509 | 3.688 | 4.0086 | 4.5234 | 4.3546 | 0.7068 | 0.7071 | 2.8445 | 4.4977 | 4.1006 | 4.0151 | 4.0131 | 4.0214 |
| pos_5333 | S-adenosyl-L-methioninamine | metab_4483 | HMDB0304476;HMDB0000988 | C01137 | 379.1508 | 2.7671 | pos | M+Na | C14H24N6O3S+2 | 0 | 44.8 | -4.1488343 | 22365-13-5 | 0.00349836 | 4.5647 | 5.0032 | 4.7054 | 4.5514 | 5.31 | 5.3276 | 4.6884 | 4.628 | 5.3799 | 4.7071 | 5.2231 | 5.3164 | 5.0706 | 5.072 | 5.0736 |
| pos_5334 | 4-Hydroxyvalerylcarnitine | metab_4484 | HMDB0241653 | - | 262.1655 | 2.7671 | pos | M+H | C12H23NO5 | 0 | 31.5 | 2.49147838 | - | 0.00804951 | 5.9465 | 5.9131 | 5.9713 | 5.8136 | 6.026 | 5.9948 | 6.1351 | 5.874 | 6.0423 | 5.7086 | 6.0374 | 6.219 | 5.7399 | 5.745 | 5.7466 |
| pos_5335 | 2-(3-Carboxy-3-aminopropyl)-L-histidine | metab_4485 | HMDB0011655 | C04441 | 298.1518 | 2.7591 | pos | M+ACN+H | C10H16N4O4 | 0 | 86.9 | 3.06864581 | - | 0.02638409 | 4.4605 | 4.6497 | 4.4072 | 3.5844 | 4.6397 | 4.5909 | 4.3594 | 4.3356 | 4.6387 | 4.4938 | 4.612 | 4.4921 | 4.23 | 4.2522 | 4.2367 |
| pos_5339 | 1-(beta-D-Ribofuranosyl)-1,4-dihydronicotinamide | metab_4489 | HMDB0011648;HMDB0011643 | C15497 | 257.1138 | 2.7351 | pos | M+H | C11H16N2O5 | 0 | 54.6 | 2.41836754 | - | 0.00320334 | 5.0131 | 5.0773 | 5.0421 | 5.1792 | 4.9018 | 5.0156 | 5.0305 | 5.1626 | 4.9337 | 5.0055 | 4.9448 | 5.0837 | 5.1222 | 5.1194 | 5.121 |
| pos_5341 | 4-Pyridoxic Acid | metab_4492 | HMDB0000017;PW_C000011 | C00847 | 184.0611 | 2.7191 | pos | M+H | C8H9NO4 | 88.7 | 0 | 3.42611936 | 82-82-6 | 0.0072697 | 4.2439 | 4.6325 | 4.4591 | 4.6135 | 4.6149 | 4.5725 | 4.5024 | 4.258 | 4.6338 | 4.2873 | 4.6014 | 4.6853 | 4.5754 | 4.5693 | 4.5709 |
| pos_5348 | Guanine | metab_4498 | PW_C000086;HMDB0000132;MJDBOTE0000457 | C00242 | 152.0572 | 2.6791 | pos | M+H | C5H5N5O | 94.5 | 0 | 3.70513767 | 73-40-5 | 0.00401122 | 5.9277 | 5.8223 | 5.7929 | 5.6469 | 5.6631 | 5.5476 | 5.8165 | 5.7348 | 5.8126 | 5.748 | 5.8229 | 5.7341 | 5.4942 | 5.4919 | 5.4954 |
| pos_5351 | Acrylamide-sodium acrylate resin | metab_4502 | HMDB0303186 | - | 215.1398 | 2.6711 | pos | M+ACN+H | C8H15NO3 | 0 | 63.2 | 4.5601556 | - | 0.04755297 | 2.6253 | 4.6025 | 4.0269 | 4.3735 | 3.3115 | 3.4074 | 3.415 | 2.6472 | 2.6476 | 2.6504 | 4.0801 | 3.508 | 3.4157 | 3.4093 | 3.4474 |
| pos_5353 | 2-Ethylpropanedioylcarnitine | metab_4504 | HMDB0241868 | - | 276.1449 | 2.6391 | pos | M+H | C12H21NO6 | 0 | 36.5 | 2.55089189 | - | 0.00287198 | 4.9928 | 4.9178 | 4.8574 | 4.8009 | 4.8332 | 4.8729 | 5.1062 | 4.9595 | 4.7996 | 4.866 | 4.9298 | 5.0373 | 4.9105 | 4.913 | 4.9119 |
| pos_5355 | Didanosine | metab_4506 | PW_C009350;HMDB0015037 | C06953 | 278.1255 | 2.6071 | pos | M+ACN+H | C10H12N4O3 | 0 | 45.5 | 3.03665467 | 69655-05-6 | 0.01580222 | 4.7348 | 4.8013 | 4.8981 | 4.7771 | 4.8154 | 4.789 | 4.9132 | 4.8104 | 4.5785 | 4.6161 | 4.7889 | 4.9619 | 4.6213 | 4.6338 | 4.6227 |
| pos_5356 | Ethosuximide | metab_4507 | HMDB0014731 | C07505 | 82.538 | 2.6071 | pos | M+H+Na | C7H11NO2 | 0 | 30.5 | 3.9033214 | 77-67-8 | 0.00504175 | 4.679 | 4.7054 | 4.7462 | 4.7504 | 4.7042 | 4.7453 | 4.7216 | 4.7287 | 4.7336 | 4.7789 | 4.7329 | 4.6888 | 4.7764 | 4.7791 | 4.7807 |
| pos_5359 | 5-Acetamidovalerate | metab_4510 | HMDB0012175;LMFA01030987 | C03087 | 160.0974 | 2.5911 | pos | M+H | C7H13NO3 | 0 | 37.4 | 3.48876084 | - | 0.00086622 | 4.2768 | 4.3351 | 4.3502 | 4.3667 | 4.9717 | 5.041 | 4.3558 | 4.3145 | 4.9561 | 4.3122 | 4.8083 | 4.7769 | 4.7875 | 4.7873 | 4.788 |
| pos_5363 | (S)-beta-Aminoisobutyric acid | metab_4515 | HMDB0002166 | C03284 | 86.061 | 2.5831 | pos | M+H-H2O | C4H9NO2 | 0 | 36.7 | 8.95553931 | 4249-19-8 | 0.00355326 | 5.4241 | 5.4553 | 5.4414 | 5.4353 | 5.5026 | 5.4801 | 5.4755 | 5.4312 | 5.5018 | 5.4488 | 5.5019 | 5.5662 | 5.4178 | 5.4147 | 5.4163 |
| pos_5366 | Cerulenin | metab_4518 | HMDB0015168;LMFA08010013 | C12058 | 241.1553 | 2.5511 | pos | M+NH4 | C12H17NO3 | 0 | 39.2 | 2.76223056 | 17397-89-6 | 0.00560237 | 5.7173 | 5.79 | 5.8002 | 5.7672 | 5.6052 | 5.7206 | 5.8572 | 5.9074 | 5.6789 | 5.9086 | 5.7083 | 5.824 | 5.8105 | 5.8103 | 5.8146 |
| pos_5367 | 3-Aminopyrazin-2-ol | metab_4519 | HMDB0245823 | - | 112.0513 | 2.5511 | pos | M+H | C4H5N3O | 0 | 34.9 | 6.66999321 | - | 0.01455885 | 4.4737 | 4.5399 | 4.515 | 4.3942 | 4.4792 | 4.3662 | 4.5326 | 4.4205 | 4.4651 | 4.5312 | 4.383 | 4.3767 | 4.5584 | 4.5468 | 4.5484 |
| pos_5397 | Nicotinuric Acid | metab_4551 | HMDB0003269 | C05380 | 198.0856 | 2.1191 | pos | M+NH4 | C8H8N2O3 | 48.5 | 0 | -9.6971419 | 583-08-4 | 0.00670699 | 4.521 | 4.7131 | 4.3996 | 4.576 | 4.1211 | 4.0797 | 4.5805 | 4.4418 | 4.295 | 4.4163 | 4.0856 | 4.3677 | 4.2075 | 4.2018 | 4.2034 |
| pos_5398 | 3-Methyl-L-Histidine | metab_4552 | HMDB0000479 | C01152 | 170.093 | 2.1111 | pos | M+H | C7H11N3O2 | 70.9 | 0 | 3.60176456 | 368-16-1 | 0.01709123 | 5.6174 | 5.3207 | 5.5185 | 5.3093 | 5.2831 | 5.4007 | 5.6905 | 5.5383 | 5.2916 | 5.5442 | 5.3389 | 5.5127 | 5.2684 | 5.2612 | 5.276 |
| pos_5418 | Dihydroorotic Acid | metab_4575 | HMDB0000528;MJDBOTE0000601 | C00337 | 176.0663 | 1.9992 | pos | M+NH4 | C5H6N2O4 | 71.7 | 0 | -1.5688085 | 155-54-4 | 0.02601127 | 6.0451 | 6.1957 | 5.9893 | 5.978 | 5.8744 | 5.9114 | 6.0023 | 5.8725 | 5.932 | 5.8512 | 5.8951 | 6.036 | 5.9763 | 5.976 | 5.9956 |
| pos_5419 | Fluroxene | metab_4576 | HMDB0252413 | - | 275.0484 | 1.9833 | pos | 2M+Na | C4H5F3O | 0 | 53.8 | 2.77690345 | - | 0.17797887 | 4.1679 | 4.3416 | 2.9658 | 3.9035 | 3.9565 | 4.5118 | 2.9842 | 3.7086 | 4.6526 | 3.0452 | 4.4562 | 4.1565 | 3.9754 | 3.9748 | 4.1034 |
| pos_5431 | LL-2,6-Diaminopimelic Acid | metab_4590 | HMDB0001370 | C00666 | 155.082 | 1.8875 | pos | M+H-2H2O | C7H14N2O4 | 72.3 | 0 | 2.82251342 | 583-93-7 | 0.00663378 | 5.7355 | 6.6169 | 6.2296 | 6.4286 | 6.0694 | 6.1693 | 6.0677 | 5.7916 | 5.5445 | 5.3618 | 6.2543 | 6.2171 | 6.1913 | 6.1954 | 6.1969 |
| pos_5434 | Coniferyl Alcohol | metab_4593 | HMDB0012915;MJDBOTE0001211 | C00590 | 163.0759 | 1.8715 | pos | M+H-H2O | C10H12O3 | 43.2 | 0 | 2.99060915 | 32811-40-8;458-35-5;32811-40-8 | 0.00193085 | 5.9515 | 5.942 | 5.9536 | 5.964 | 5.9208 | 5.9564 | 5.9549 | 5.9526 | 5.9569 | 5.9854 | 5.9259 | 5.9223 | 5.9889 | 5.9897 | 5.988 |
| pos_5435 | 4-Hydroxybenzaldehyde | metab_4594 | HMDB0011718;MJDBOTE0000554 | C00633 | 123.0447 | 1.8715 | pos | M+H | C7H6O2 | 81.7 | 0 | 5.16278684 | 123-08-0 | 0.00568545 | 5.4849 | 5.5681 | 5.5121 | 5.4884 | 5.473 | 5.4636 | 5.6398 | 5.4609 | 5.4527 | 5.501 | 5.4975 | 5.5701 | 5.5347 | 5.5345 | 5.5304 |
| pos_5437 | (S)-5-Amino-3-oxohexanoate | metab_4596 | HMDB0012131 | C03656 | 128.0712 | 1.8555 | pos | M+H-H2O | C6H11NO3 | 0 | 61.1 | 4.16563095 | 19355-90-9 | 0.0050826 | 5.8232 | 6.2214 | 6.0098 | 6.0969 | 5.9191 | 5.9678 | 5.9569 | 5.889 | 5.8156 | 5.8488 | 5.9824 | 5.9978 | 5.9778 | 5.9776 | 5.9815 |
| pos_5438 | Furfural | metab_4597 | HMDB0032914 | C14279 | 97.0293 | 1.8555 | pos | M+H | C5H4O2 | 38.6 | 0 | 9.07038846 | 98-01-1 | 0.00919321 | 4.2659 | 4.1817 | 4.2699 | 4.3304 | 4.2163 | 4.291 | 4.2314 | 4.2303 | 4.3057 | 4.2924 | 4.165 | 4.1889 | 4.3312 | 4.3309 | 4.3241 |
| pos_5440 | 4-Amino-1-piperidinecarboxylic acid | metab_4600 | HMDB0060385 | C16837 | 109.0768 | 1.8235 | pos | M+H-2H2O | C6H12N2O2 | 0 | 32.4 | 5.37912504 | 959317-82-9 | 0.00447044 | 4.7449 | 5.571 | 5.1992 | 5.403 | 5.0277 | 5.1365 | 5.0823 | 4.7879 | 4.5298 | 4.4208 | 5.2239 | 5.1781 | 5.167 | 5.1684 | 5.1708 |
| pos_5441 | 1-Butanol | metab_4601 | HMDB0004327 | C06142 | 116.1077 | 1.8155 | pos | M+ACN+H | C4H10O | 0 | 41.3 | 9.86161797 | 71-36-3 | 0.01161606 | 4.738 | 4.9262 | 4.7899 | 4.8875 | 5.1945 | 5.2485 | 4.5538 | 4.7498 | 5.082 | 4.4552 | 5.317 | 5.0972 | 4.7824 | 4.7822 | 4.791 |
| pos_5453 | 4-Amino-5-oxopentanamide | metab_4614 | HMDB0258161 | - | 169.0363 | 1.7595 | pos | M+K | C5H10N2O2 | 0 | 52.3 | -8.4917771 | - | 0.01118518 | 4.4077 | 4.4694 | 4.5202 | 4.6738 | 4.56 | 4.766 | 4.5535 | 4.7092 | 4.9334 | 4.8476 | 4.7599 | 4.5906 | 5.3599 | 5.3608 | 5.3687 |
| pos_547 | 7-[3-Hydroxy-2-(3-hydroxyoctyl)-5-oxocyclopentyl]heptanoic acid | metab_4632 | HMDB0244517 | - | 730.5406 | 6.4984 | pos | M+H-H2O, 2M+NH4 | C20H36O5 | 0 | 55.7 | -8.1169848 | - | 0.16706283 | 4.2328 | 5.4366 | 4.7553 | 5.7046 | 4.9961 | 5.2159 | 5.8581 | 5.4915 | 5.5227 | 5.3655 | 5.9489 | 5.3279 | 5.71 | 5.5863 | 5.7263 |
| pos_548 | PC(20:5/0:0) | metab_4643 | LMGP01050050 | - | 542.3256 | 6.5063 | pos | M+H, M+Na | C28H48NO7P | 42.6 | 0 | 2.70778694 | - | 0.00952522 | 6.3458 | 6.6368 | 6.4279 | 6.3542 | 6.4978 | 6.5246 | 6.5791 | 6.4299 | 6.568 | 6.3675 | 6.3051 | 6.5127 | 6.4977 | 6.4899 | 6.4915 |
| pos_5480 | Niacinamide | metab_4644 | PW_C001086;HMDB0001406 | C00153 | 123.0559 | 1.3032 | pos | M+H | C6H6N2O | 53.5 | 0 | 5.05046299 | 98-92-0 | 0.00413024 | 5.9346 | 5.9368 | 5.6981 | 5.9647 | 5.3983 | 5.956 | 5.7899 | 5.8857 | 5.9077 | 5.9714 | 5.9382 | 6.0388 | 5.8277 | 5.8256 | 5.8291 |
| pos_5488 | D-Glucaro-1,4-lactone | metab_4652 | HMDB0041862 | C21095 | 236.9988 | 1.2232 | pos | M+2Na-H | C6H8O7 | 0 | 37.9 | 3.46115229 | 5027-63-4 | 0.078121 | 3.3578 | 4.8494 | 4.9792 | 4.8754 | 4.7756 | 4.9392 | 4.8604 | 3.3799 | 4.2472 | 3.383 | 5.0686 | 4.6975 | 4.9065 | 4.9063 | 4.964 |
| pos_5509 | O-Acetylcarnitine | metab_4676 | HMDB0000201;HMDB0240773 | C02571 | 204.1237 | 1.1355 | pos | M+H | C9H17NO4 | 75.5 | 0 | 3.27640011 | 3040-38-8 | 0.00045253 | 7.3824 | 7.1143 | 7.19 | 7.0638 | 7.0898 | 7.4433 | 7.2192 | 7.1512 | 7.344 | 7.1955 | 7.3552 | 7.223 | 7.1536 | 7.1534 | 7.1538 |
| pos_5513 | Cytosine | metab_4681 | HMDB0000630;MJDBOTE0000458 | C00380 | 112.0512 | 1.1355 | pos | M+H | C4H5N3O | 89.8 | 0 | 6.37489101 | 71-30-7 | 0.01125661 | 4.6171 | 4.7972 | 4.6581 | 4.5851 | 4.7715 | 4.7471 | 4.705 | 4.6283 | 4.7985 | 4.674 | 4.7055 | 4.7587 | 4.7502 | 4.7499 | 4.7585 |
| pos_5518 | 4-(1-Hydroxy-2-methoxyethyl)-5-(hydroxymethyl)-2-methylpyridin-3-ol | metab_4686 | HMDB0260272 | - | 178.0868 | 1.1275 | pos | M+H-2H2O | C10H15NO4 | 0 | 41.4 | 2.43323519 | - | 0.00448404 | 4.7667 | 4.844 | 4.652 | 4.6384 | 4.7922 | 5.0398 | 4.8347 | 4.3792 | 5.0449 | 4.6744 | 4.9402 | 4.9527 | 4.7701 | 4.7662 | 4.7678 |
| pos_5519 | 5-Aminopentanoic acid | metab_4687 | HMDB0003355;LMFA01100040 | C00431 | 162.0507 | 1.1275 | pos | M+2Na-H | C5H11NO2 | 0 | 42.2 | 4.50778705 | 660-88-8 | 0.00929206 | 5.7401 | 5.8146 | 5.7113 | 5.6222 | 5.6022 | 5.638 | 5.7912 | 5.6228 | 5.5999 | 5.6856 | 5.5119 | 5.7265 | 5.702 | 5.6959 | 5.7035 |
| pos_5521 | Norvaline | metab_4690 | HMDB0013716;MJDBOTE0000679 | C01799 | 118.0869 | 1.1275 | pos | M+H | C5H11NO2 | 44.9 | 0 | 5.84904684 | 760-78-1 | 0.00216655 | 7.6029 | 7.6939 | 7.6428 | 7.6716 | 7.6964 | 7.6476 | 7.7183 | 7.682 | 7.5371 | 7.6837 | 7.5593 | 7.6289 | 7.6804 | 7.6788 | 7.6804 |
| pos_5524 | METHACHOLINE | metab_4693 | HMDB0015654 | C07471 | 160.1338 | 1.1118 | pos | M+H | C8H17NO2 | 62 | 0 | 3.46988475 | 55-92-5 | 0.00805916 | 6.5393 | 6.8533 | 6.9648 | 6.7774 | 6.8189 | 6.6793 | 6.9325 | 6.3882 | 6.7929 | 6.9946 | 6.6102 | 6.9164 | 6.598 | 6.5928 | 6.5994 |
| pos_5528 | Alpha-Methylene-Gamma-Butyrolactone | metab_4697 | HMDB0246945 | C20578 | 99.0449 | 1.0959 | pos | M+H | C5H6O2 | 69.9 | 0 | 8.41500308 | - | 0.00187919 | 4.8914 | 4.9026 | 4.919 | 4.997 | 4.9369 | 4.9571 | 4.8621 | 4.9764 | 4.9199 | 4.9621 | 4.9408 | 4.8704 | 4.9876 | 4.9868 | 4.9884 |
| pos_553 | PC(18:3(9Z,12Z,15Z)/20:1(11Z)) | metab_4699 | LMGP01011689;HMDB0008209;PW_C004257 | C00157 | 810.6022 | 6.53 | pos | M+H, M+Na | C46H84NO8P | 0 | 86.3 | 1.80893819 | - | 0.17899722 | 7.5623 | 6.7297 | 7.6349 | 7.8559 | 7.4365 | 7.5753 | 7.5986 | 7.7101 | 7.6737 | 7.6812 | 7.6873 | 7.6084 | 7.7408 | 7.5962 | 7.7381 |
| pos_5532 | N-Acetyl-beta-alanine | metab_4702 | HMDB0061880 | C01073 | 132.0663 | 1.0642 | pos | M+H | C5H9NO3 | 0 | 48.2 | 5.69811745 | 3025-95-4 | 0.01027077 | 4.8704 | 5.1463 | 4.6602 | 5.0781 | 5.2918 | 4.9619 | 4.9191 | 5.0019 | 5.1425 | 5.1372 | 4.9943 | 5.0171 | 5.2359 | 5.2276 | 5.2292 |
| pos_5550 | Pyroglutamic Acid | metab_4720 | PW_C000182;HMDB0000267;MJDBOTE0000549 | C01879 | 147.0769 | 1.0247 | pos | M+NH4 | C5H7NO3 | 73.2 | 0 | 3.73333247 | 98-79-3 | 0.00272884 | 6.2123 | 6.1499 | 6.073 | 6.0505 | 6.1715 | 6.2139 | 6.101 | 6.1204 | 6.0967 | 6.1537 | 6.262 | 6.2363 | 6.2111 | 6.2102 | 6.2126 |
| pos_5551 | L-Threonine | metab_4721 | PW_C000109;HMDB0000167;MJDBOTE0000464 | C00188 | 120.0661 | 1.0247 | pos | M+H | C4H9NO3 | 72.2 | 0 | 5.0445017 | 72-19-5 | 0.01367396 | 4.9766 | 5.3425 | 5.1576 | 5.2634 | 5.0447 | 5.2196 | 5.1703 | 4.919 | 5.1316 | 5.1571 | 5.1683 | 5.1087 | 5.1742 | 5.1646 | 5.1756 |
| pos_556 | PC(20:2(11Z,14Z)/15:0) | metab_4730 | PW_C004378;HMDB0008330;LMGP01011839 | C00157 | 772.5839 | 6.5459 | pos | M+H, M+Na | C43H82NO8P | 0 | 35.9 | -1.4880446 | - | 0.03651678 | 7.2113 | 7.0384 | 7.201 | 7.1697 | 7.2409 | 7.1964 | 7.1222 | 7.2034 | 7.1768 | 7.1645 | 7.1655 | 7.2544 | 7.2537 | 7.2583 | 7.2285 |
| pos_5561 | 4-Trimethylammoniobutanoic Acid | metab_4732 | HMDB0001161;PW_C000899 | C01181 | 146.118 | 1.0167 | pos | M+H | C7H15NO2 | 55.3 | 0 | 3.14286125 | 407-64-7 | 0.00193969 | 5.5212 | 5.6423 | 5.7342 | 5.6842 | 5.5484 | 5.6451 | 5.5302 | 5.6016 | 5.6469 | 5.613 | 5.7194 | 5.5597 | 5.7009 | 5.6997 | 5.7013 |
| pos_5563 | N(6)-Methyllysine | metab_4734 | HMDB0002038 | C02728 | 193.1552 | 1.0087 | pos | M+CH3OH+H | C7H16N2O2 | 0 | 42.2 | 3.41939337 | 1188-07-4 | 0.00743502 | 3.4959 | 4.6171 | 4.4138 | 4.3534 | 5.7296 | 5.7309 | 4.3915 | 4.6699 | 5.2267 | 4.3599 | 5.3352 | 5.0731 | 5.3797 | 5.3795 | 5.3852 |
| pos_5565 | N-Methyl-L-Histidine | metab_4736 | HMDB0255176 | C03298 | 170.093 | 1.0087 | pos | M+H | C7H11N3O2 | 84 | 0 | 3.29163549 | 24886-03-1 | 0.00337401 | 6.5257 | 6.132 | 6.4379 | 6.1761 | 6.2382 | 6.3096 | 6.625 | 6.4674 | 6.1816 | 6.5094 | 6.2167 | 6.4501 | 6.1889 | 6.186 | 6.1876 |
| pos_5566 | L-Proline | metab_4737 | HMDB0251528;PW_C000106;HMDB0000162;MJDBOTE0000527 | C00148 | 116.0713 | 1.0087 | pos | M+H | C5H9NO2 | 87 | 0 | 6.28640528 | 147-85-3 | 0.00343019 | 6.5496 | 6.4751 | 6.4343 | 6.4417 | 6.5067 | 6.4544 | 6.5704 | 6.4881 | 6.3655 | 6.4894 | 6.3471 | 6.5648 | 6.454 | 6.4524 | 6.4554 |
| pos_5589 | Zalcitabine | metab_4760 | PW_C009386;HMDB0015078;HMDB0037019;HMDB0255802 | C07207 | 253.1277 | 0.9454 | pos | M+ACN+H | C9H13N3O3 | 0 | 47.8 | -8.4942332 | 7481-89-2;5718-77-4 | 0.01630145 | 5.4263 | 5.4573 | 5.3145 | 5.3113 | 5.2304 | 5.2638 | 5.4938 | 5.3914 | 5.2956 | 5.4404 | 5.3325 | 5.4893 | 5.5524 | 5.5522 | 5.5645 |
| pos_5610 | Isochorismate | metab_4783 | HMDB0304395 | C00885 | 191.0323 | 0.8897 | pos | M+H-2H2O | C10H10O6 | 0 | 59.2 | -7.1474332 | 22642-82-6 | 0.00336391 | 6.019 | 5.9761 | 5.9789 | 5.9997 | 5.9012 | 5.9976 | 6.0058 | 5.9211 | 6.036 | 6.1234 | 6.0332 | 5.9264 | 6.0228 | 6.0241 | 6.0257 |
| pos_562 | PC(22:6/0:0) | metab_4792 | LMGP01050056 | - | 568.3413 | 6.5779 | pos | M+H, M+Na | C30H50NO7P | 71 | 0 | 2.70717426 | - | 0.00748198 | 6.7431 | 6.779 | 6.7242 | 6.6399 | 6.785 | 6.7341 | 6.8388 | 6.6862 | 6.6843 | 6.5503 | 6.9014 | 6.9751 | 6.6641 | 6.6578 | 6.6595 |
| pos_5644 | Glycerophosphocholine | metab_4818 | HMDB0008685;HMDB0008820;HMDB0008753;HMDB0008424;HMDB0008718;HMDB0008228;HMDB0008491;HMDB0008225;HMDB0007895;HMDB0008060;HMDB0008819;HMDB0008358;HMDB0008455;HMDB0007929;HMDB0008687;HMDB0008258;HMDB0008161;HMDB0008786;HMDB0007962;HMDB0008261;HMDB0008327;HMD | C00670 | 296.0667 | 0.8262 | pos | M+K | C8H20NO6P | 0 | 53.9 | 2.66813629 | 28319-77-9 | 0.00535154 | 4.9988 | 4.9232 | 4.9602 | 5.1055 | 4.6758 | 4.9447 | 4.9082 | 4.7795 | 5.0048 | 4.8538 | 4.9746 | 5.0135 | 4.8593 | 4.8636 | 4.863 |
| pos_5655 | Glycerylphosphorylcholine | metab_4830 | HMDB0252858;HMDB0000086 | C00670 | 280.0927 | 0.8183 | pos | M+Na | C8H20NO6P | 0 | 68.3 | 2.51659573 | 28319-77-9 | 0.01235 | 5.9067 | 5.9042 | 5.8834 | 5.9556 | 5.8207 | 5.8698 | 5.8885 | 5.808 | 5.9119 | 5.7764 | 5.8799 | 5.9638 | 5.7451 | 5.7535 | 5.7551 |
| pos_566 | LysoPE(20:4(5Z,8Z,11Z,14Z)/0:0) | metab_4835 | HMDB0011517 | - | 502.2942 | 6.594 | pos | M+H, M+Na | C25H44NO7P | 0 | 39 | 2.76177233 | 652149-09-2 | 0.01327865 | 5.8507 | 5.9824 | 5.9228 | 5.8155 | 5.8965 | 5.9653 | 5.8409 | 5.8179 | 5.9524 | 5.9152 | 5.7832 | 5.8174 | 5.9139 | 5.9246 | 5.9154 |
| pos_5670 | 1-Methylhistidine | metab_4847 | PW_C000001;HMDB0000001 | C01152 | 170.0929 | 0.8103 | pos | M+H | C7H11N3O2 | 92.2 | 0 | 2.82666297 | 368-16-1;332-80-9 | 0.0117123 | 5.7601 | 5.4046 | 5.6864 | 5.442 | 5.5001 | 5.589 | 5.8904 | 5.7361 | 5.4328 | 5.8001 | 5.4309 | 5.6913 | 5.4372 | 5.4292 | 5.4386 |
| pos_5682 | N2-Methyl-L-lysine | metab_4860 | HMDB0240689 | - | 205.093 | 0.8023 | pos | M+2Na-H | C7H16N2O2 | 0 | 34.1 | 4.1076875 | - | 0.01086668 | 5.108 | 5.0151 | 5.1336 | 4.9705 | 4.8395 | 4.7938 | 4.9976 | 4.8602 | 5.2446 | 5.137 | 4.9283 | 4.9947 | 4.7834 | 4.7745 | 4.7762 |
| pos_569 | LysoPE(18:2(9Z,12Z)/0:0) | metab_4868 | HMDB0011507 | - | 478.2939 | 6.6259 | pos | M+H, M+Na | C23H44NO7P | 0 | 75.3 | 2.35542487 | 85046-18-0 | 0.0159556 | 5.894 | 6.2709 | 6.199 | 5.9962 | 6.0363 | 6.2376 | 5.978 | 5.8707 | 6.1804 | 5.9065 | 5.9956 | 5.98 | 6.1498 | 6.1371 | 6.1387 |
| pos_574 | PC(22:5/0:0) | metab_4923 | HMDB0010403 | C04230 | 570.3569 | 6.6499 | pos | M+H, M+Na | C30H52NO7P | 83.5 | 0 | 2.58194988 | - | 0.00673322 | 7.3943 | 7.3594 | 7.3426 | 7.3222 | 7.3403 | 7.2608 | 7.45 | 7.2396 | 7.295 | 7.2288 | 7.432 | 7.4797 | 7.2966 | 7.2963 | 7.3015 |
| pos_5747 | Inosinic acid | metab_4931 | HMDB0000175;HMDB0257686;PW_C000114 | C00130 | 313.0365 | 0.7703 | pos | M+H-2H2O | C10H13N4O8P | 0 | 51.4 | 9.25348442 | 131-99-7 | 0.01363435 | 4.7143 | 4.8671 | 4.6973 | 4.6677 | 4.5681 | 4.6398 | 4.9616 | 4.58 | 4.4618 | 4.553 | 4.4038 | 4.7473 | 4.6926 | 4.702 | 4.7037 |
| pos_5778 | 2-Hydroxyadipic acid | metab_4965 | LMFA01170049;HMDB0000321 | C02360 | 145.0501 | 0.6824 | pos | M+H-H2O | C6H10O5 | 0 | 59.4 | 3.21862405 | 18294-85-4 | 0.00412768 | 6.4241 | 6.4237 | 6.4348 | 6.4376 | 6.3939 | 6.4373 | 6.4304 | 6.4362 | 6.4414 | 6.449 | 6.4028 | 6.4013 | 6.4605 | 6.457 | 6.4585 |
| pos_5779 | Uracil | metab_4966 | PW_C000204;HMDB0000300;MJDBOTE0000838 | C00106 | 113.0353 | 0.6824 | pos | M+H | C4H4N2O2 | 75.5 | 0 | 6.65016492 | 66-22-8 | 0.0093751 | 5.5649 | 5.5637 | 5.571 | 5.5724 | 5.5321 | 5.5673 | 5.5833 | 5.5895 | 5.5682 | 5.5991 | 5.5467 | 5.5508 | 5.6131 | 5.6054 | 5.607 |
| pos_5789 | 5-Hydroxypentanoic acid | metab_4977 | HMDB0061927 | C02804 | 83.0501 | 0.6343 | pos | M+H-2H2O | C5H10O3 | 0 | 58.8 | 7.99034912 | 13392-69-3 | 0.00880929 | 4.7 | 4.6804 | 4.7116 | 4.6962 | 4.653 | 4.6897 | 4.7079 | 4.703 | 4.6975 | 4.7251 | 4.673 | 4.6638 | 4.747 | 4.7398 | 4.7414 |
| pos_5791 | Cyclopentanecarboxylic acid | metab_4980 | HMDB0250667 | - | 97.0657 | 0.6263 | pos | M+H-H2O | C6H10O2 | 0 | 30.2 | 7.60117941 | - | 0.00497231 | 5.7287 | 5.7326 | 5.7333 | 5.7412 | 5.6929 | 5.7359 | 5.7461 | 5.7392 | 5.7377 | 5.7726 | 5.7164 | 5.72 | 5.7726 | 5.7724 | 5.7687 |
| pos_5795 | Maltol | metab_4984 | HMDB0030776 | C11918 | 127.0396 | 0.6023 | pos | M+H | C6H6O3 | 79.6 | 0 | 4.83100443 | 118-71-8 | 0.00540401 | 6.1832 | 6.1935 | 6.1901 | 6.2032 | 6.1362 | 6.1877 | 6.1934 | 6.2042 | 6.1838 | 6.1981 | 6.1527 | 6.1519 | 6.2132 | 6.2086 | 6.2101 |
| pos_580 | LysoPC(18:3(6Z,9Z,12Z)/0:0) | metab_4990 | HMDB0010387 | C04230 | 518.3254 | 6.5379 | pos | M+H, M+Na | C26H48NO7P | 0 | 89.1 | 2.51768143 | - | 0.00732335 | 6.6919 | 7.1447 | 6.9588 | 6.9603 | 6.8682 | 6.9568 | 6.9677 | 6.7992 | 7.0786 | 6.7921 | 6.823 | 6.9 | 7.0287 | 7.0285 | 7.0341 |
| pos_5812 | Trans-2-Hexenal | metab_5004 | HMDB0031496 | C08497 | 99.0813 | 0.4183 | pos | M+H | C6H10O | 42.2 | 0 | 8.60525714 | 6728-26-3 | 0.00466809 | 5.0496 | 5.0563 | 5.0504 | 5.0519 | 5.0046 | 5.0543 | 5.0649 | 5.0671 | 5.0612 | 5.0964 | 5.0517 | 5.0552 | 5.0923 | 5.092 | 5.0957 |
| pos_5813 | 2-Hydroxyphenylacetic Acid | metab_5005 | HMDB0000669;MJDBOTE0000611 | C05852 | 135.0446 | 0.3943 | pos | M+H-H2O | C8H8O3 | 68.6 | 0 | 3.5880188 | 614-75-5 | 0.00378 | 4.2075 | 4.2672 | 4.2141 | 4.2157 | 4.172 | 4.2373 | 4.2457 | 4.2383 | 4.2449 | 4.263 | 4.2244 | 4.222 | 4.2697 | 4.2696 | 4.2668 |
| pos_586 | 1-Pentadecanoyl-sn-Glycero-3-Phosphocholine | metab_5055 | LMGP01050016 | - | 482.3254 | 6.7218 | pos | M+H-H2O, M+H | C23H48NO7P | 74.2 | 0 | 2.61847821 | - | 0.00133302 | 7.1361 | 7.3868 | 7.2325 | 7.1573 | 7.1525 | 7.2519 | 7.2299 | 7.1032 | 7.3702 | 7.1732 | 7.2124 | 7.1218 | 7.2523 | 7.2523 | 7.2533 |
| pos_591 | LysoPE(0:0/20:1(11Z)) | metab_5110 | HMDB0011482 | - | 508.3412 | 6.7538 | pos | M+H, M+Na | C25H50NO7P | 0 | 68.7 | 2.82133992 | - | 0.00506195 | 6.926 | 7.2295 | 7.0594 | 7.0757 | 6.9844 | 6.959 | 7.0713 | 7.0068 | 7.0763 | 7.0429 | 6.8714 | 6.8944 | 7.0906 | 7.0933 | 7.0949 |
| pos_592 | PE(18:1(9Z)/0:0) | metab_5120 | LMGP02050004 | - | 480.3097 | 6.7538 | pos | M+H, M+Na | C23H46NO7P | 90.7 | 0 | 2.57670203 | - | 0.0179268 | 5.9747 | 6.1812 | 6.1832 | 6.0712 | 5.9704 | 5.9955 | 5.995 | 6.0746 | 6.1746 | 6.0187 | 5.9103 | 5.8613 | 5.9794 | 5.9792 | 5.9927 |
| pos_599 | LysoPC(20:3(8Z,11Z,14Z)/0:0) | metab_5139 | HMDB0010394 | - | 546.3566 | 6.7378 | pos | M+H, M+Na | C28H52NO7P | 0 | 90.3 | 2.130574 | 1006703-11-2 | 0.00589376 | 7.0879 | 7.4138 | 7.3355 | 7.3787 | 7.2172 | 7.3486 | 7.284 | 7.3498 | 7.3622 | 7.2503 | 7.0324 | 7.2361 | 7.5012 | 7.5046 | 7.5063 |
| pos_602 | LysoPC(22:4(7Z,10Z,13Z,16Z)/0:0) | metab_5144 | HMDB0010401 | C04230 | 572.372 | 6.8018 | pos | M+H, M+Na | C30H54NO7P | 0 | 88.8 | 1.60225298 | - | 0.01302123 | 7.378 | 7.3236 | 7.2516 | 7.287 | 7.32 | 7.2234 | 7.4444 | 7.1686 | 7.2338 | 7.2404 | 7.3366 | 7.4349 | 7.2183 | 7.2272 | 7.2288 |
| pos_616 | Glycylprolylarginine | metab_5159 | HMDB0252828 | - | 311.1833 | 6.8418 | pos | M+H-H2O, M+H | C13H24N6O4 | 0 | 31.6 | 2.07562443 | - | 0.01439547 | 5.0415 | 5.0373 | 4.9624 | 5.033 | 4.9046 | 5.0131 | 4.9398 | 5.015 | 5.0555 | 5.1198 | 4.9985 | 5.0454 | 5.064 | 5.0739 | 5.0756 |
| pos_622 | 9-Oxohexadecanoic acid | metab_5166 | HMDB0030973 | - | 288.2539 | 6.4904 | pos | M+NH4, M+Na | C16H30O3 | 0 | 41 | 2.00628112 | 54527-30-9 | 0.0147774 | 5.1901 | 5.3299 | 5.3367 | 5.3268 | 5.2517 | 5.3358 | 5.2979 | 5.1499 | 5.2742 | 5.2469 | 5.2613 | 5.257 | 5.394 | 5.3935 | 5.4048 |
| pos_636 | LysoPC(17:0/0:0) | metab_5181 | HMDB0012108 | C04230 | 532.3386 | 7.0491 | pos | M+NH4, M+Na | C25H52NO7P | 0 | 93.2 | 2.34878901 | 50930-23-9 | 0.01619776 | 7.1679 | 6.8744 | 6.7126 | 6.8194 | 6.5164 | 6.5337 | 6.8197 | 6.6481 | 6.6259 | 6.7671 | 6.5302 | 6.6291 | 6.8849 | 6.8963 | 6.8978 |
| pos_644 | 1-(11Z-docosenoyl)-glycero-3-phosphate | metab_5190 | HMDB0062304;LMGP10050029 | - | 510.3567 | 7.089 | pos | M+H-H2O, M+NH4 | C25H49O7P | 0 | 56.2 | 2.65164868 | - | 0.00365278 | 7.3457 | 7.3052 | 7.196 | 7.1079 | 7.1011 | 7.1301 | 7.2742 | 7.1493 | 7.2217 | 7.1607 | 7.1662 | 7.2354 | 7.2547 | 7.2545 | 7.2518 |
| pos_647 | LysoPC(0:0/20:4(5Z,8Z,11Z,14Z)) | metab_5193 | HMDB0061699 | - | 544.3387 | 7.0411 | pos | M+H, M+Na | C28H50NO7P | 0 | 80.5 | -1.870339 | 67341-29-1 | 0.01204585 | 7.5228 | 7.2924 | 7.2137 | 7.4536 | 6.982 | 6.9565 | 7.2449 | 7.1521 | 7.008 | 7.1982 | 6.9161 | 6.996 | 7.34 | 7.3482 | 7.3498 |
| pos_648 | Alpha-Linolenic acid | metab_5194 | HMDB0001388;LMFA01030152;PW_C001073 | C06427 | 279.2325 | 6.3792 | pos | M+H, M+Na | C18H30O2 | 0 | 31.8 | 2.20340583 | 463-40-1 | 0.05941749 | 3.6793 | 4.3928 | 4.1733 | 4.3977 | 4.4705 | 4.4529 | 4.6376 | 3.7014 | 4.5305 | 3.7295 | 3.7119 | 4.493 | 4.9407 | 4.9403 | 4.895 |
| pos_665 | Phaseic acid | metab_5213 | HMDB0302844 | C09707 | 281.139 | 6.3158 | pos | M+H-H2O, M+H | C15H20O5 | 0 | 36.6 | 2.19718336 | 24394-14-7 | 0.00293639 | 6.8819 | 6.6624 | 6.6748 | 6.6954 | 6.649 | 6.6557 | 6.6969 | 6.7523 | 6.7217 | 6.7887 | 6.7125 | 6.6741 | 6.6537 | 6.6562 | 6.6551 |
| pos_669 | Chavicol | metab_5217 | HMDB0034107 | C16930 | 135.0809 | 6.3158 | pos | M+H-H2O, M+H | C9H10O | 34.6 | 0 | 3.29216269 | 501-92-8 | 0.00468622 | 6.4001 | 6.2082 | 6.1988 | 6.2243 | 6.195 | 6.1946 | 6.2515 | 6.2686 | 6.2589 | 6.3267 | 6.2366 | 6.2268 | 6.2092 | 6.2096 | 6.2129 |
| pos_670 | Cinnamaldehyde | metab_5219 | HMDB0245299;HMDB0258628;HMDB0003441;MJDBOTE0001083 | C00903 | 133.0651 | 6.3158 | pos | M+H-H2O, M+H | C9H8O | 68.7 | 0 | 2.20882972 | 104-55-2 | 0.03735239 | 5.488 | 5.3043 | 5.3034 | 5.2955 | 5.2534 | 5.2833 | 5.321 | 5.3146 | 5.3245 | 5.3907 | 5.3171 | 5.3306 | 5.265 | 5.257 | 5.288 |
| pos_671 | (E)-4-Octenoic acid | metab_5220 | HMDB0039793;LMFA01030951 | - | 107.0862 | 6.3158 | pos | M+H-2H2O, M+Na | C8H14O2 | 0 | 34.1 | 4.98342601 | 18776-92-6 | 0.00495299 | 5.8412 | 5.6839 | 5.7108 | 5.6946 | 5.6563 | 5.6966 | 5.7229 | 5.7547 | 5.7309 | 5.7726 | 5.7024 | 5.6941 | 5.673 | 5.6688 | 5.6704 |
| pos_677 | PE(P-18:1(11Z)/22:6(4Z,7Z,10Z,13Z,16Z,19Z)) | metab_5226 | HMDB0011427 | - | 806.5697 | 7.2549 | pos | M+H-2H2O, M+CH3OH+H | C45H76NO7P | 0 | 51 | 0.40112985 | - | 0.10317293 | 5.7035 | 6.3737 | 6.0966 | 6.1134 | 5.9102 | 6.217 | 6.3702 | 6.2507 | 6.1676 | 6.3214 | 6.1268 | 6.1205 | 6.0458 | 5.9673 | 6.049 |
| pos_686 | Xi-7-Hydroxyhexadecanedioic acid | metab_5236 | HMDB0037830 | - | 325.1994 | 6.2682 | pos | M+H-2H2O, M+Na | C16H30O5 | 0 | 40.1 | 2.96078086 | - | 0.01679046 | 5.0055 | 4.7505 | 5.4237 | 5.1752 | 5.1438 | 4.8379 | 5.2099 | 5.4707 | 5.5533 | 5.156 | 5.2652 | 5.2203 | 5.1032 | 5.0913 | 5.1046 |
| pos_698 | (+/-)-1,4-Nonanediol diacetate | metab_5249 | HMDB0037184 | - | 245.1753 | 7.3757 | pos | M+H-H2O, M+H | C13H24O4 | 0 | 53.3 | 2.40199381 | 67715-81-5 | 0.00283888 | 6.9074 | 6.9311 | 6.9071 | 6.8808 | 6.7742 | 6.8917 | 6.9008 | 6.8588 | 6.9432 | 6.9411 | 6.8462 | 6.8631 | 6.9347 | 6.9371 | 6.9361 |
| pos_700 | 2-Hydroxy-p-mentha-1,8-dien-6-one | metab_5253 | HMDB0037012 | - | 149.0965 | 7.3757 | pos | M+H-H2O, M+H-2H2O | C10H14O2 | 0 | 35.7 | 2.31106795 | 51200-86-3 | 0.00317794 | 5.5779 | 5.6252 | 5.6022 | 5.536 | 5.453 | 5.6016 | 5.5833 | 5.5249 | 5.6597 | 5.6277 | 5.5256 | 5.5579 | 5.5243 | 5.524 | 5.5265 |
| pos_701 | 13(S)-HODE | metab_5254 | - | C14762 | 297.2431 | 7.3677 | pos | M+H-H2O, M+H | C18H32O3 | 40.8 | 0 | 2.24874522 | - | 0.01547232 | 5.224 | 5.2476 | 5.1929 | 5.1052 | 5.0311 | 5.2444 | 5.1564 | 5.0421 | 5.2694 | 5.176 | 5.0398 | 5.1584 | 5.1337 | 5.1443 | 5.1462 |
| pos_710 | Lauryldiethanolamine | metab_5264 | - | - | 274.2746 | 6.2047 | pos | M+H-H2O, M+H | C16H35NO2 | 69.9 | 0 | 2.12813636 | - | 0.00141911 | 7.6341 | 7.7145 | 7.6096 | 7.6185 | 7.5981 | 7.7345 | 7.6637 | 7.6281 | 7.6519 | 7.6734 | 7.6833 | 7.6592 | 7.6941 | 7.6931 | 7.693 |
| pos_711 | Sorbitan palmitate | metab_5265 | HMDB0029887;LMFA07011019 | - | 444.3328 | 6.181 | pos | M+H-H2O, M+ACN+H | C22H42O6 | 0 | 51.8 | 2.00830593 | 26266-57-9 | 0.01834873 | 4.5671 | 4.0398 | 4.3581 | 3.5727 | 4.2217 | 4.0828 | 4.2383 | 3.9475 | 4.8419 | 4.0998 | 4.3499 | 3.8625 | 4.3126 | 4.3121 | 4.3261 |
| pos_714 | PC(14:0/P-18:1(11Z)) | metab_5268 | HMDB0007897 | C00157 | 738.5446 | 7.4873 | pos | M+H, M+Na | C40H78NO7P | 0 | 67.5 | 5.27332904 | - | 0.17046997 | 5.968 | 5.5402 | 4.9871 | 4.7162 | 5.1401 | 5.4224 | 4.9449 | 5.0447 | 4.7856 | 4.8927 | 5.1419 | 4.8558 | 5.7881 | 5.7947 | 5.6557 |
| pos_716 | Cyclobutyl-[4-[2-(3,5-dimethylpyrazol-1-yl)ethyl]piperazin-1-yl]methanone | metab_5270 | - | - | 291.2171 | 6.181 | pos | M+H, M+NH4 | C16H26N4O | 30.6 | 0 | -3.0171364 | - | 0.02358507 | 4.6413 | 4.7283 | 4.8995 | 4.6871 | 4.7482 | 4.7552 | 5.1164 | 4.734 | 4.8062 | 4.7714 | 4.7635 | 4.8219 | 4.6769 | 4.6769 | 4.6945 |
| pos_719 | 2-Lysophosphatidylcholine | metab_5273 | HMDB0258493 | C04230 | 524.3725 | 7.5192 | pos | M+H, M+Na | C26H54NO7P | 0 | 92.1 | 2.71444665 | - | 0.0034377 | 8.3928 | 8.3102 | 8.3351 | 8.3047 | 8.1404 | 8.2008 | 8.3725 | 8.2454 | 8.2564 | 8.2615 | 8.2966 | 8.3297 | 8.3275 | 8.3305 | 8.3289 |
| pos_723 | PC(20:1(11Z)/14:1(9Z)) | metab_5278 | LMGP01011808;PW_C004344;HMDB0008296 | C00157 | 780.5546 | 7.5672 | pos | M+NH4, M+Na | C42H80NO8P | 0 | 89.5 | 4.22961458 | - | 0.02677429 | 6.3527 | 6.6845 | 6.7431 | 6.6422 | 6.5295 | 6.6751 | 6.7576 | 6.7571 | 6.8303 | 6.7604 | 6.7285 | 6.5693 | 6.7796 | 6.7597 | 6.7804 |
| pos_726 | Neocnidilide | metab_5281 | HMDB0034450 | C17002 | 159.1173 | 6.1729 | pos | M+H-2H2O, M+H | C12H18O2 | 0 | 66.3 | 2.38077563 | 4567-33-3 | 0.01080642 | 4.7368 | 4.5935 | 4.4948 | 4.5022 | 4.5451 | 4.5951 | 4.5669 | 4.5189 | 4.6182 | 4.7344 | 4.481 | 4.5645 | 4.6346 | 4.6273 | 4.6361 |
| pos_730 | Triethanolamine | metab_5286 | HMDB0032538 | C06771 | 150.1129 | 7.6306 | pos | M+H, M+Na | C6H15NO3 | 70.3 | 0 | 3.0906577 | 102-71-6 | 0.00399709 | 5.9522 | 5.9382 | 5.9073 | 5.9434 | 5.8851 | 5.9298 | 5.9056 | 5.9203 | 5.9525 | 5.968 | 5.9061 | 5.8776 | 5.9741 | 5.9759 | 5.9776 |
| pos_738 | PE-NMe2(18:0/18:4(6Z,9Z,12Z,15Z)) | metab_5294 | PW_C060010;HMDB0113993 | C04308 | 768.5573 | 7.6784 | pos | M+H, M+Na | C43H78NO8P | 0 | 85.7 | 4.58303311 | - | 0.29757116 | 6.3319 | 6.4636 | 6.4694 | 6.3323 | 6.2899 | 6.6876 | 6.5984 | 6.4633 | 6.494 | 6.622 | 6.5774 | 6.5047 | 5.6734 | 5.6725 | 5.4213 |
| pos_74 | L-Methionine S-oxide | metab_5295 | HMDB0002005 | C02989 | 148.0432 | 1.1673 | pos | M+H, M+Na, M+H-H2O | C5H11NO3S | 68.5 | 0 | 2.99375893 | 3226-65-1 | 0.00562218 | 6.1862 | 6.2642 | 6.2133 | 6.1991 | 6.1618 | 6.1551 | 6.1598 | 6.1638 | 6.1984 | 6.2283 | 6.1455 | 6.1997 | 6.2637 | 6.2604 | 6.2652 |
| pos_777 | 1-O-Hexadecyl-sn-glycero-3-phosphocholine | metab_5336 | HMDB0243890;LMGP01060010 | C13903 | 482.3616 | 7.7262 | pos | M+H, M+Na | C24H52NO6P | 0 | 71.8 | 2.18484712 | - | 0.0042 | 6.3858 | 6.4171 | 6.453 | 6.4796 | 6.2599 | 6.4636 | 6.5152 | 6.4955 | 6.403 | 6.3622 | 6.4059 | 6.5064 | 6.3547 | 6.3583 | 6.3562 |
| pos_780 | PC(15:0/20:2(11Z,14Z)) | metab_5340 | HMDB0007946;PW_C003996;LMGP01011422 | C00157 | 772.587 | 7.7023 | pos | M+H, M+Na | C43H82NO8P | 0 | 71.3 | 2.54222503 | - | 0.15288008 | 5.5975 | 6.6906 | 6.5747 | 6.6632 | 6.5263 | 6.533 | 6.7317 | 6.6805 | 6.6873 | 6.6855 | 6.728 | 6.6885 | 6.4674 | 6.471 | 6.3481 |
| pos_790 | 13(S)-HpODE | metab_5351 | HMDB0003871 | C04717 | 295.2273 | 6.1569 | pos | M+H-H2O, M+H | C18H32O4 | 59.6 | 0 | 1.78110432 | 33964-75-9 | 0.00637433 | 3.973 | 4.2913 | 3.7683 | 3.8122 | 4.2969 | 4.1491 | 4.1511 | 3.8667 | 4.0584 | 3.9169 | 4.1682 | 4.2801 | 4.6567 | 4.662 | 4.6581 |
| pos_805 | Tridecanedioylcarnitine | metab_5367 | HMDB0241347 | - | 410.2522 | 6.0844 | pos | M+H, M+Na | C20H37NO6 | 0 | 31.7 | 2.41856823 | - | 0.00403253 | 4.2316 | 4.663 | 4.988 | 4.8009 | 4.0696 | 4.3018 | 4.8557 | 4.2747 | 4.4054 | 3.7932 | 4.3859 | 4.0732 | 5.158 | 5.1615 | 5.1593 |
| pos_806 | Lactapiperanol D | metab_5368 | HMDB0033631 | - | 325.1995 | 6.0844 | pos | M+H, M+CH3OH+H | C18H28O5 | 0 | 34.4 | -4.5691864 | 243447-94-1 | 0.01325338 | 5.1439 | 5.1256 | 4.9647 | 4.9367 | 5.1363 | 5.0687 | 5.3996 | 4.9414 | 5.419 | 5.2423 | 5.1609 | 5.1931 | 5.2396 | 5.2394 | 5.2295 |
| pos_812 | Scopoletin | metab_5374 | HMDB0034344;MJDBOTE0001128 | C01752 | 385.0926 | 6.0684 | pos | M+H, 2M+H | C10H8O4 | 0 | 58.5 | 1.97898162 | 92-61-5 | 0.00731298 | 5.2388 | 5.2599 | 5.2389 | 5.2519 | 5.1963 | 5.2626 | 5.2555 | 5.2764 | 5.2331 | 5.3041 | 5.2046 | 5.2273 | 5.2293 | 5.2232 | 5.2248 |
| pos_817 | Loliolide | metab_5379 | HMDB0302428 | - | 229.144 | 6.0604 | pos | M+CH3OH+H, M+ACN+Na | C11H16O3 | 0 | 30.3 | 3.1176837 | - | 0.00573877 | 4.8495 | 4.8769 | 4.8148 | 4.8287 | 4.8626 | 4.9078 | 4.8576 | 4.8498 | 4.8241 | 4.8778 | 4.8666 | 4.8905 | 5.3263 | 5.3229 | 5.3277 |
| pos_825 | Alongside | metab_5388 | HMDB0248179 | - | 207.0995 | 6.0157 | pos | M+H-H2O, M+H | C12H16O4 | 0 | 53.2 | -9.1532436 | - | 0.00408804 | 5.2147 | 5.2234 | 5.2152 | 5.2731 | 5.1797 | 5.2596 | 5.2538 | 5.2381 | 5.2576 | 5.248 | 5.2184 | 5.2455 | 5.241 | 5.2374 | 5.239 |
| pos_834 | Trans-Stilbene | metab_5398 | - | - | 198.1284 | 4.4994 | pos | M+H, M+NH4 | C14H12 | 83.6 | 0 | 3.58619353 | - | 0.00407667 | 5.2747 | 5.248 | 5.3056 | 4.8773 | 6.1755 | 5.926 | 5.1863 | 5.5137 | 6.1434 | 5.2454 | 5.814 | 6.0169 | 5.7506 | 5.7525 | 5.7541 |
| pos_838 | Chakanoside I | metab_5402 | HMDB0302044 | - | 263.0934 | 4.2982 | pos | M+H-2H2O, M+NH4 | C14H18O7 | 0 | 41.9 | 6.64002149 | - | 0.00622554 | 5.1818 | 5.2094 | 4.9977 | 4.9448 | 4.8584 | 5.1764 | 5.1347 | 4.7637 | 4.6702 | 4.9542 | 5.2288 | 4.9767 | 4.909 | 4.9037 | 4.9053 |
| pos_839 | 2-Aminobiphenyl | metab_5403 | HMDB0245011 | - | 170.097 | 4.2827 | pos | M+H, M+NH4 | C12H11N | 34.5 | 0 | 3.40222587 | - | 0.00881357 | 3.9469 | 4.0126 | 3.9261 | 3.9287 | 4.2991 | 4.19 | 4.0975 | 3.886 | 4.0895 | 4.0103 | 4.0463 | 4.1482 | 4.8088 | 4.8016 | 4.8031 |
| pos_846 | 5-Methoxyindole | metab_5410 | HMDB0246822 | - | 189.1028 | 2.8151 | pos | M+H, M+ACN+H | C9H9NO | 0 | 42.8 | 3.8014753 | - | 0.00413586 | 4.8083 | 5.1832 | 5.0815 | 5.731 | 4.7062 | 4.7158 | 4.7109 | 5.2603 | 5.5782 | 4.734 | 4.7711 | 4.9322 | 5.0467 | 5.0465 | 5.0435 |
| pos_848 | Indoleacetaldehyde | metab_5412 | PW_C000926;HMDB0001190 | C00637 | 160.0762 | 2.8151 | pos | M+H, M+NH4 | C10H9NO | 51.8 | 0 | 3.40619331 | 2591-98-2 | 0.00426155 | 6.8813 | 6.7243 | 6.7987 | 6.8433 | 6.6746 | 6.6542 | 6.8632 | 6.6168 | 6.8838 | 6.8247 | 6.5771 | 6.8056 | 6.749 | 6.7527 | 6.7505 |
| pos_849 | OXYQUINOLINE | metab_5413 | - | C19434 | 146.0605 | 2.8151 | pos | M+H, M+NH4 | C9H7NO | 61.6 | 0 | 3.31461612 | 148-24-3 | 0.02029337 | 5.2368 | 5.1735 | 5.0275 | 4.9535 | 5.0918 | 4.872 | 5.1185 | 5.0403 | 5.0676 | 4.9705 | 4.8969 | 5.196 | 5.1472 | 5.1617 | 5.1634 |
| pos_85 | 5-Hydroxyindoleacetic acid | metab_5414 | PW_C000609;HMDB0000763 | C05635 | 209.0927 | 2.8151 | pos | M+H, M+NH4, M+H-H2O | C10H9NO3 | 77.8 | 0 | 3.4562417 | 54-16-0 | 0.01138432 | 6.4719 | 6.3742 | 6.1751 | 6.0563 | 6.2676 | 6.0044 | 6.3295 | 6.2463 | 6.2069 | 6.1723 | 6.0494 | 6.4076 | 6.3597 | 6.3689 | 6.3611 |
| pos_852 | (R)-3-hydroxybutyrylcarnitine | metab_5417 | HMDB0062735 | - | 230.1393 | 2.8071 | pos | M+H-H2O, M+CH3OH+H | C11H21NO5 | 0 | 47.5 | 2.50866234 | - | 0.07645876 | 3.9374 | 4.1195 | 3.8628 | 3.7348 | 4.1399 | 4.3513 | 4.2639 | 4.03 | 4.3313 | 3.9838 | 4.5277 | 4.4274 | 4.3604 | 4.361 | 4.4171 |
| pos_86 | Cinnamic acid | metab_5425 | HMDB0000567;HMDB0000930 | C10438 | 166.0868 | 2.8309 | pos | M+H-H2O, M+NH4, M+H | C9H8O2 | 0 | 36.1 | 3.63841591 | 102-94-3;140-10-3;621-82-9 | 0.0036993 | 7.2356 | 7.324 | 7.2173 | 7.1445 | 7.1285 | 7.2117 | 7.3058 | 7.2561 | 7.2052 | 7.2187 | 7.1508 | 7.3209 | 7.2723 | 7.2737 | 7.2755 |
[truncated: 8,859 more chars]
